# Enantio‐ and Diastereoselective, Complete Hydrogenation of Benzofurans by Cascade Catalysis

**DOI:** 10.1002/anie.202103910

**Published:** 2021-05-05

**Authors:** Daniel Moock, Tobias Wagener, Tianjiao Hu, Timothy Gallagher, Frank Glorius

**Affiliations:** ^1^ Organisch-Chemisches Institut Westfälische Wilhelms-Universität Münster Corrensstrasse 40 48149 Münster Germany

**Keywords:** benzofuran, cascade catalysis, enantioselective hydrogenation, heterogeneous catalysis, homogeneous catalysis

## Abstract

We report an enantio‐ and diastereoselective, complete hydrogenation of multiply substituted benzofurans in a one‐pot cascade catalysis. The developed protocol facilitates the controlled installation of up to six new defined stereocenters and produces architecturally complex octahydrobenzofurans, prevalent in many bioactive molecules. A unique match of a chiral homogeneous ruthenium‐N‐heterocyclic carbene complex and an in situ activated rhodium catalyst from a complex precursor act in sequence to enable the presented process.

## Graphical abstract

A one‐pot cascade catalytic hydrogenation is developed producing enantioenriched octahydrobenzofurans with up to six new stereocenters. The dual catalytic, sequential process is enabled by a unique match of a chiral ruthenium carbene catalyst and an achiral rhodium catalyst, which is generated in situ upon adjustment of the reaction conditions.

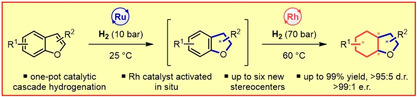

Saturated, (hetero)cyclic structures still represent an area of underexplored chemical space, especially in the pharmaceutical industry.^[1]^ One explanation for this shortcoming is a lack of suitable methods permitting synthetic access to the desired motifs. Arene hydrogenation can be a powerful tool for this purpose,^[2]^ since the user can exploit the broad availability of (hetero)aromatic structures as easy‐to‐modify starting materials. However, in practice the utility of this synthetic tool is often limited by the choice of catalyst for one of two reasons: Either the employed catalyst is only capable of performing a partial hydrogenation, leaving an aromatic core in bicyclic structures; or it fails to deliver the product in an asymmetric fashion (Figure 1 A). The latter especially impedes the exploration of chiral cyclohexane motifs.[^2d^, ^3^] Therefore, a transformation capable of fully reducing abundant arenes to the corresponding saturated (hetero)cycles under full stereocontrol is highly desired. In an effort to develop this space further, we herein report a highly stereoselective one‐pot, cascade hydrogenation of benzofurans (Figure 1 C).

**Figure 1:**
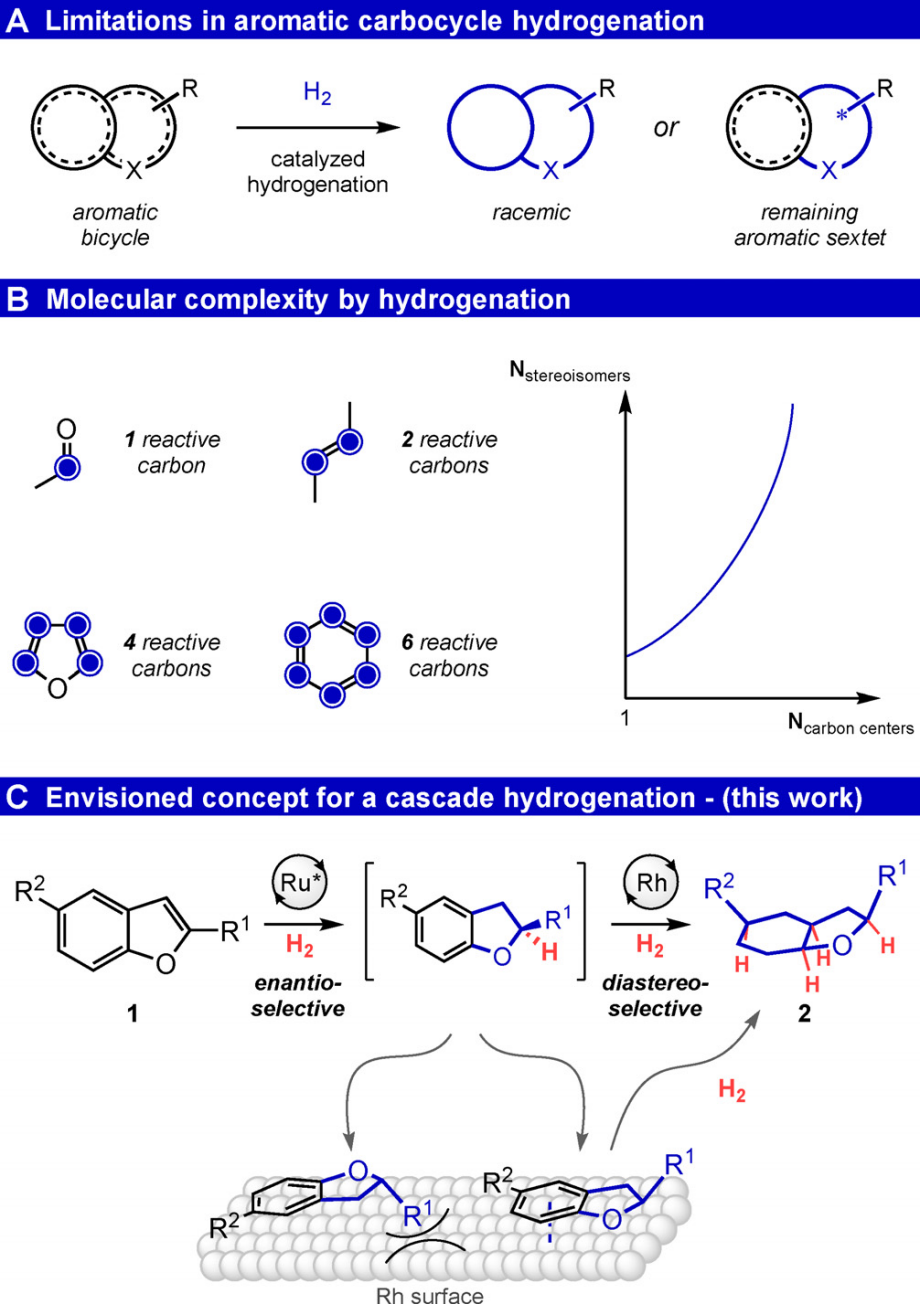
A) Catalytic arene hydrogenation is currently limited by the inability to produce enantioenriched, fully saturated, carbocyclic compounds. B) Hydrogenation is a powerful tool to convert sp^2^‐ to sp^3^‐centers and introduce complexity into molecules at multiple carbon centers in a single operation. C) The aim of this work is to develop a cascade hydrogenation protocol with two distinct catalysts to obtain enantioenriched octahydrobenzofurans.

Benzofurans **1** and their respective 2,3‐saturated derivatives are ubiquitous structural units in natural products and pharmaceutical compounds.^[4]^ Despite this prevalence, there are few reports on the asymmetric partial hydrogenation of benzofurans published today,^[5]^ and to the best of our knowledge, fully saturated octahydro derivatives **2** have never been accessed by enantioselective catalysis.^[6]^ We value this shortcoming detrimental, since the more complex octahydrobenzofuran scaffold can hold promising properties for drugs and other applications to explore.^[7]^ Reasons for this underexploration can be the problem of facile C−O bond cleavage during the hydrogenation of the furanyl ring,^[4e]^ but foremost the lack of an asymmetric catalyst capable of full reduction of both aromatic cores.^[8]^

We approached this problem by using two different catalytic systems fulfilling distinct tasks in the sought‐after transformation to the desired fused bicycle. The first catalyst would partially reduce the heterocyclic core yielding a 2,3‐dihydrobenzofuran, rendering this compound as a chiral intermediate, while the second catalyst would perform a full hydrogenation of the remaining six‐membered all‐carbon ring. We envisioned that the second catalyst would be able to utilize the installed stereocenter to guide a downstream diastereoselective reduction,^[9]^ for example, on a solid surface under a Horiuti–Polanyi mechanism.^[10]^

To catalyze the first step we chose the established Ru((*R*,*R*)‐SINpEt)_2_ catalyst **3**,^[11]^ which features two *N*,*N*′‐bis(naphthylethyl)imidazolidinium‐2‐ylidene (NHC) ligands and was previously shown to catalyze the partial hydrogenation of benzofurans with high enantiomeric excess under mild conditions (TOF 1092 h^−1^).[^5c^, ^11a^] To prevent a racemic background reaction in a one‐pot approach, the second (heterogeneous) catalyst in our envisioned process would need to be formed or activated in situ after the transformation of the starting material to the intermediate 2,3‐dihydrobenzofuran is complete. In case of in situ formation, a suitable precursor needs to be chosen, and reaction conditions need to be adjusted such that they match the induction periods of the two catalysts. We found the Rh‐CAAC (cyclic alkyl amino carbene) precursor **4**, which was studied in depth by Zeng, Bullock, us, and others to be a perfect match.^[12]^

We started our investigation on 2‐methyl‐5‐fluorobenzofuran **1 x** as the test substrate to be able to study the preservation of the fluoro substituent in addition to yield and stereochemical outcome of the reaction. Early screening attempts confirmed our hypothesis, as the proposed and subsequently optimized system successfully transformed **1 x** to the fully saturated analog (see Table 1). Several controls show how unique the achieved match of catalysts is, since all other tested common catalysts for arene hydrogenation failed to yield the desired product in an asymmetric fashion (Table 1, entries 2–6). Although the high chemoselectivity of catalyst **4** could be leveraged to preserve the sensitive fluoro moiety, defluorination levels were still comparatively high. The beneficial effect of silica gel as supporting material in these terms, which we observed in earlier studies,^[13]^ could not be exploited in the dual catalytic system, since it deactivated catalyst **3** and yielded a racemic product mixture (similarly for acidic alumina, Table 1, entries 7 and 8). In the course of our optimization we observed that an elevated reaction temperature of 60 °C was necessary to activate the rhodium catalyst in the presence of **3**, lower temperatures were not sufficient to achieve full conversion (Table 1, entry 10). An increased amount of **4** was necessary to overcome a disadvantageous, deactivating interaction of both catalytic systems and deliver complete conversion of the 2,3‐dihydro intermediate (Table 1, entry 11). However, this remarkable activity was imperative in the development of a one‐pot protocol for the complete enantioselective hydrogenation of benzofurans. In essence, this procedure resembles a type of sequential catalysis in which precursors for both catalysts are present in the reaction mixture from the beginning and their activation is solely controlled by changing temperature and hydrogen pressure as external stimuli. Hence, it can be described as an assisted cascade catalysis.[^14^, ^15^]

**Table 1:** Selected deviations from optimized conditions. 
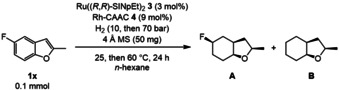

Entry	Deviation	Yield **A** [%]	Yield **B** [%]	d.r.	e.r.
1	–	64	26	94:6	95:5
2	Ru/C instead of **4**	14	76	95:5	50:50
3	Rh/C	49	56	90:10	51:49
4	Pd/C	55	48	92:8	51:49
5	Pt/C	–	2	–	–
6	[Rh(COD)Cl]_2_	73	25	94:6	50:50
7	SiO_2_ instead of molecular sieves	62	36	98:2	50:50
8	Alumina A	63	29	94:6	50:50
9	Alumina N	64	33	94:6	95:5
10	45 °C instead of 60 °C	12	4	–	–
11	5 mol % 4	33	13	94:6	93:7

The reaction was started with 10 bar H_2_ pressure and 25 °C. After 3 h reaction time the initial conditions were adjusted to the final indicated values. Yield of product A and by‐product B, d.r., and e.r. values were determined by GC‐FID. COD=1,5‐cyclooctadiene, MS=molecular sieves.

With the optimized conditions in hand, we continued our investigation into the scope of the reaction (Figure 2). The method tolerates various substitutions on the six‐membered ring. Next to 2‐methylbenzofuran **1 a**, the influence of primary, secondary, and tertiary alkyl substituents was investigated systematically for the 5‐position, all giving high yield and very good d.r.‐ and e.r.‐values (**2 a**–**e**). Furthermore, the synthetically accessible 7‐position (**2 f,g**) as well as multiple substituents (**2 h**) were tolerated well with excellent selectivity.

**Figure 2:**
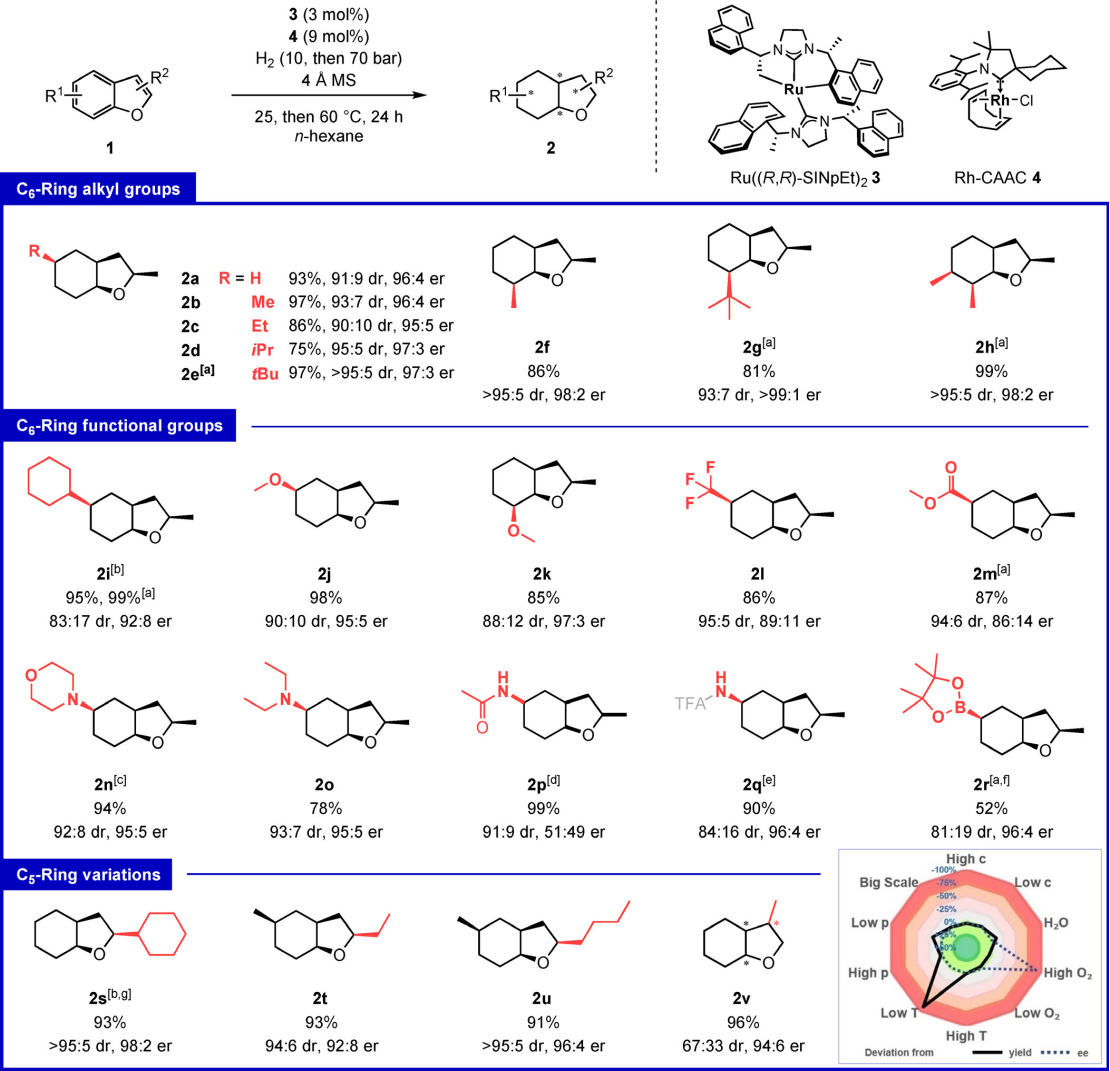
Optimized reaction conditions, scope, and reaction‐condition based sensitivity assessment. The reactions were performed on 0.30 mmol scale. After 3 h reaction time hydrogen pressure and temperature were increased as indicated. d.r. values were determined by GC–MS of the crude product mixture. All minor diastereomers are combined in a single number. [a] Extended reaction time of 48 h. [b] Phenyl substrate was used. [c] Crystal structure obtained, CCDC 2071064. [d] d.r. was determined by NMR. [e] Free amine substrate was used, protected before isolation, d.r. determined after protection. [f] Full conversion was not observed after 48 h, unreacted dihydro intermediate was fully recovered. [g] Fluoro protocol was used as described in Figure 3. TFA=trifluoroacetyl.

This method mainly yields only two of all possible diastereomers and the observed e.r. was identical for major and minor species. Next, we investigated functional group tolerance. A phenyl group was reduced under the reaction conditions, giving the fully saturated product (**2 i**). Concomitantly, a methyl ester group was preserved without reduction (**2 m**). Tertiary amino functions were tolerated well (**2 n, o**). A crystal of the hydrochloride salt of **2 n** could be used to determine the absolute configuration of the hydrogenated products to be 2*R*,3a*S*,5*R*,7a*S* (see Figure S2, remaining scope entries were assigned in analogy). When using acetamide‐protected primary amine **1 p** as the starting material, excellent yield and diastereoselectivity were observed. Yet, the product was obtained as racemic mixture (**2 p**).^[16]^ However, this limitation could be overcome by using the free primary amine as starting material for hydrogenation yielding the enantioenriched product **2 q** after subsequent protection with trifluoroacetic anhydride to ease isolation efforts. We were very pleased when discovering that a boryl ester was tolerated giving the corresponding product **2 r** with 96:4 e.r. Both primary amines as well as protected boryl esters constitute synthetically valuable and widely applied functional handles rendering these entries highlights of our work. Variation of the 2‐substituent on the five‐membered ring was well tolerated (**2 s**–**u**). A methyl group in 3‐position did not reduce the yield or e.r., but a significant drop in the diastereomeric ratio was observed (**2 v**). Presumably, the differentiation of both sides of the 2,3‐dihydro intermediate at the heterogeneous rhodium catalyst surface is hampered with this substitution pattern. The evaluation of the reaction‐condition‐based sensitivity of the developed protocol revealed a mostly robust reaction (Figure 2, see SI for details).^[17]^

Fluorinated, saturated carbocycles are emerging motifs in drug discovery and the design of functional materials.^[18]^ This class of compounds attracts increasing attention, since incorporating fluoro moieties into target molecules allows for the modulation of their physicochemical properties with little influence on steric demand.^[19]^ Although hydrogenation of fluoro arenes could give a straightforward synthetic access to these motifs, this is known to be highly challenging, as substrates and reaction intermediates are prone to hydrodefunctionalization.^[20]^ As expected, we observed increased levels of defluorination when focusing on fluorinated starting materials as the elevated temperature of 60 °C was necessary to activate the rhodium species in our developed methodology. Thus, we decided to alter the protocol to a sequential approach to allow access to this highly desirable class of enantioenriched molecules. By performing a short silica‐plug filtration after the first three hours of reaction time, we were able to perform the second hydrogenation at room temperature with lower catalyst loading (Figure 3). This procedure gave us access to mono‐, di‐, and trifluoro‐octahydrobenzofurans (**2 w**–**ac**). To the best of our knowledge, this is the first time the multifluorinated cyclohexane motif is accessed by asymmetric catalysis.^[21]^ We observed no significant defluorination for mono‐ and difluoro compounds. **2 w** could be isolated in 99 % yield, while losses during isolation accounted for the diminished yield of **2 x**–**aa**. Solely for trifluoro‐octahydrobenzofurans **2 ab** and **2 ac** significant defluorination was observed. A crystal structure of **2 ab** verified the assigned absolute configuration (see Figure S3).

**Figure 3:**
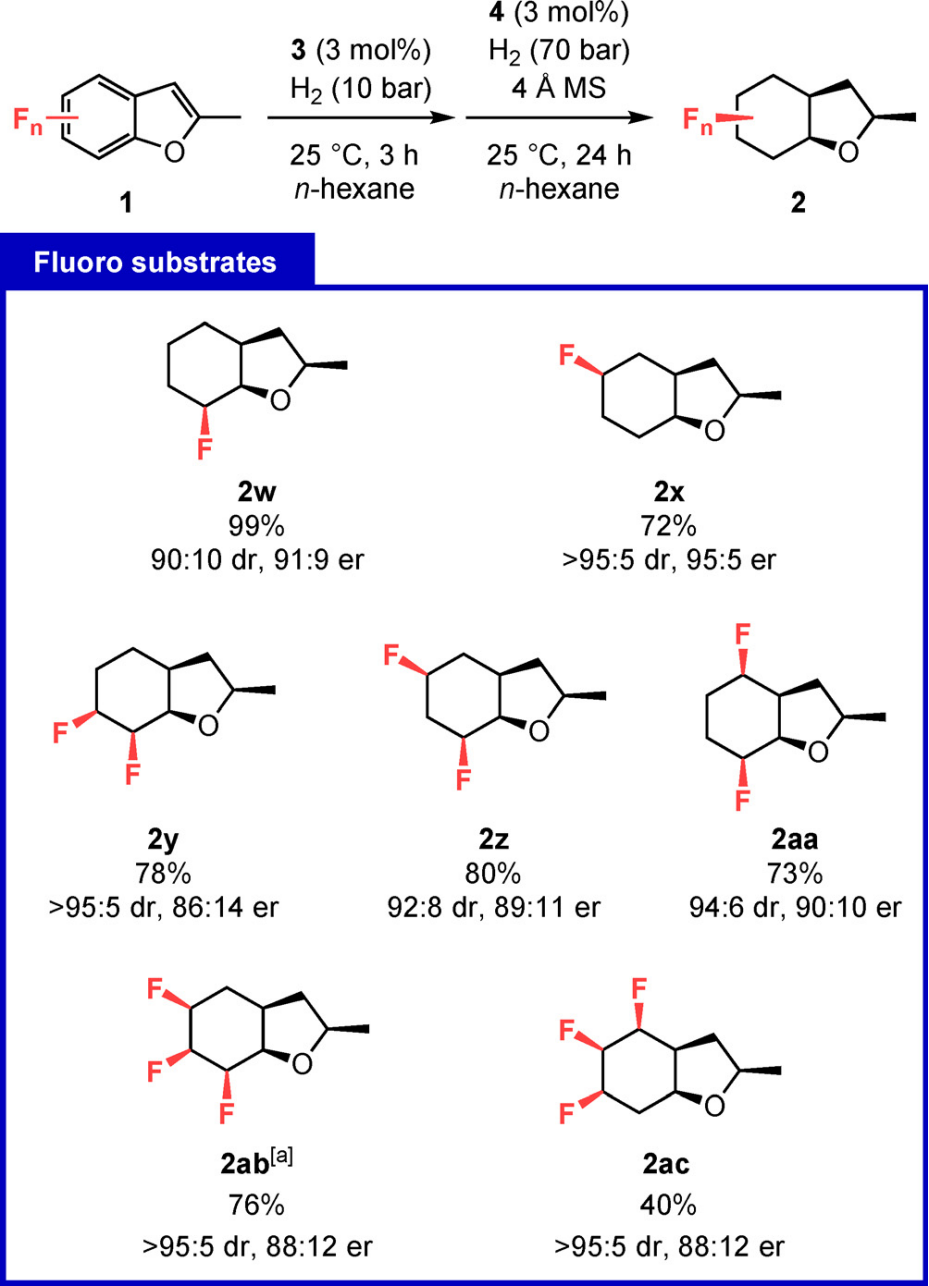
Developed protocol and scope of fluorinated substrates. The reactions were performed on 0.30 mmol scale. After 3 h reaction time at the initial hydrogen pressure the reaction mixture was filtered through a short silica plug, **4** and molecular sieves were added and the resulting mixture was again reacted at an increased hydrogen pressure. The high volatility of mono‐ and difluoro products reduced their isolated yields. d.r. was determined by GC–MS of the crude reaction mixture. All minor diastereomers are combined in a single number. [a] Crystal structure obtained, CCDC 2071065.

In conclusion, we have discovered and developed a one‐pot cascade catalysis protocol enabling a simple access to yet underexplored enantioenriched octahydrobenzofurans with a scope of >25 examples. The method tolerates various functional groups, including synthetically valuable free amines and boryl esters. Complex, three‐dimensional products from flat precursors are obtained in high yields and very good diastereo‐ and enantioselectivities. Exploiting the enhanced chemoselectivity of rhodium cyclic alkyl amino carbene **4**, highly challenging fluoro substituents can be tolerated and up to six asymmetric centers can be installed under substantial stereocontrol. Thus, the procedure gives selective access to one stereoisomer from 64 possible structures. The general strategy of controlling catalyst activation at different reaction stages presented herein is envisioned to be expanded to further substrate classes in upcoming research.

## Conflict of interest

The authors declare no conflict of interest.

## Supporting information

As a service to our authors and readers, this journal provides supporting information supplied by the authors. Such materials are peer reviewed and may be re‐organized for online delivery, but are not copy‐edited or typeset. Technical support issues arising from supporting information (other than missing files) should be addressed to the authors.

Supplementary
