# Supplementary material for: Enantio‐ and Diastereoselective, Complete Hydrogenation of Benzofurans by Cascade Catalysis
Source: Angew Chem Int Ed Engl. 2021 May 5;60(24):13677–81. doi: 10.1002/anie.202103910 (PMC8251578; doi:10.1002/anie.202103910)
Supplement: Supplementary file 1 — Supplementary [file ANIE-60-13677-s001.pdf]

Angewandte Chemie
Eine Zeitschrift der Gesellschaft Deutscher Chemiker

Supporting Information

Enantio- and Diastereoselective, Complete Hydrogenation of Benzofurans by Cascade Catalysis

*Daniel Moock, Tobias Wagener, Tianjiao Hu, Timothy Gallagher, and Frank Glorius**

anie_202103910_sm_miscellaneous_information.pdf

Supporting Information

Table of Contents

1. General Information S2
2. Synthesis of catalysts.. S3
3. Synthesis of Starting Materials... S4
4. Sensitivity Screen... S26
5. Hydrogenation Reactions.. S28
6. Mechanistic Considerations S71
7. X-Ray Analysis... S76
8. Literature S79
9. NMR Spectra.. S80

1. General Information

Unless otherwise noted, all reactions were carried out under an atmosphere of argon in oven-dried glassware. The employed solvents were either dried by distillation over standard drying agents and stored under argon over molecular sieves (diethyl ether (Na-benzophenone), toluene (CaH_2), THF (Na-benzophenone)) or directly used from a solvent purification system (HPLC grade, dried via an alumina/molecular sieves column under positive argon pressure; *n*-hexane, dichloromethane). Methanol (3 Å) and dimethylformamide (3 Å) were purchased as dry solvents from commercial suppliers and stored over molecular sieves.

Catalytic hydrogenation reactions were prepared under argon and carried out in Berghof High Pressure Reactors using hydrogen gas. Reaction temperatures are reported as the temperature of the bath surrounding the vessel unless otherwise stated.

Commercially available chemicals were obtained from Acros Organics, Aldrich Chemical Co., Strem Chemicals, Alfa Aesar, ABCR, Combi-Blocks, Chempur and TCI Europe and used as received. (*R*)- and (*S*)-1-(1-naphthyl)ethylamine were obtained from BASF (ChiPros®). Unless otherwise noted, no optimizations of the yields were performed for substrate syntheses.

Analytical thin layer chromatography (TLC) was performed on silica gel 60 F254 aluminum plates (Merck). TLC plates were visualized by exposure to short wave ultraviolet light (254nm, 366nm) and were dipped into a solution of $KMnO_4$. Flash chromatography was performed on Acros Organics silica gel (35-70 mesh) under a positive pressure of argon, eluting with the specified solvent system.

GC-MS spectra were recorded on an Agilent Technologies 7890A GC-system with an Agilent 5975C VL MSD or an Agilent 5975 inert Mass Selective Detector (EI) and a HP-5MS column (0.25 mm x 30 m, film: 0.25 µm). Enantiomeric ratios of isolated products were determined with an Agilent Technologies 7890B GC-system and a Supelco β-Dex column or on an Astec Chiraldex G-TA column using methods starting at 50 °C, always holding this temperature for 5 min and heating with a defined gradient, e.g. 4 °C/min up to a defined temperature, e.g. 70 °C, followed by continued gradient heating, e.g. 20 °C/min up to a final temperature, e.g. 220 °C. A standardized notation is used for all samples, which would result in 50_4_70_20_220 for this mentioned example. Alternatively, enantiomeric ratios were determined using an Agilent Technologies 1200 Series HPLC with a Daicel Chemical Industries LTD Chiralpak AD-H or OD-H columns (0.46 cm x 25 cm). The signals were detected by UV-absorption spectroscopy (at 210, 230 or 254 nm). Exact ESI mass spectra were recorded on a Bruker Daltonics MicroTof spectrometer. Exact GC-EI mass spectra were recorded on a Thermo Fisher Scientific Trace 1310 GC Exactive Orbitrap, equipped with a Thermo Gold TG-5SILMS (30 m, ID 0.25 mm, 0.25 µm) column. ^{1}H, ^{13}C, ^{19}F, and ^{11}B NMR

spectra were recorded on a Bruker Avance II300 or Avance II400, AgilentDD2 500 or AgilentDD2 600 in the indicated solvents. Chemical shifts (*δ*) are given in ppm relative to TMS. The residual solvent signals were used as references and the chemical shifts converted to the TMS scale ($CDCl_3$: δ_H = 7.26 ppm, δ_C = 77.16 ppm; CD_2Cl_2: δ_H = 5.32 ppm, δ_C = 53.84 ppm). ^{19}F NMR spectra are referenced according to the proton resonance of TMS as the primary reference for the unified chemical shift scale (IUPAC recommendation 2001). Broadband ^{19}F-decoupled ^{13}C spectra were recorded using the WURST decoupling method.

2. Synthesis of catalysts

Synthesis of Ru-((*R*,*R*)SINpEt)$_2$ catalyst **1**

The synthesis was performed according to a literature procedure.[1] Synthesis of achiral Ru-(ICy)$_2$ was prepared via an analogous procedure.

Synthesis of Rh-CAAC catalyst **2**

The synthesis was performed according to a literature procedure.[2]

3. Synthesis of Starting Materials

General procedure 1 for the synthesis of (2,2-diethoxyethoxy)benzene derivatives (GP1):

According to a modified literature procedure,[3] the phenol derivative (30 mmol, 1.0 equiv.), bromoacetaldehyde diethyl acetal (30 mmol, 1.0 equiv.), and K_2CO_3 (60 mmol, 2.0 equiv.) were dissolved in dimethylformamide (1.8 M) in a Schlenk tube under argon atmosphere and the resulting mixture was stirred for 16 h at 130 °C. The reaction was quenched with water and extracted with *n*-pentane (3x). The combined organic layers were washed with aqueous NaOH solution (2.0 M), water, and brine and dried over $MgSO_4$. The volatiles were removed under reduced pressure and the crude product was purified by column chromatography on silica gel.

General procedure 2 for the synthesis of benzofurans by acid promoted cyclisation (GP2):

According to a modified literature procedure,[3] the 1-(2,2-diethoxyethoxy)benzene derivative (20 mmol, 1.0 equiv.) was added to a round bottom flask containing polyphosphoric acid (0.2 g/mmol substrate) and toluene (0.2 M). The resulting mixture was stirred for 16 h at 110 °C. A continuous vigorous stirring was crucial for the reaction process. After decantation, the remainder was rinsed with pentane (1x), the organic fractions combined, and the volatiles were removed under reduced pressure. The crude product was purified by column chromatography on silica gel.

General procedure 3 for the synthesis of 2-methylbenzofurans by methylation (GP3):

According to a modified literature procedure,[4] in a dry Schlenk tube, the benzofuran derivative (5.0 mmol, 1.0 equiv.) was dissolved in THF (0.5 M) at –78 °C and *n*-butyl lithium (2.0 M in *n*-hexane, 6.5 mmol, 1.3 equiv.) was added dropwise. The resulting mixture was stirred for 2 h at low temperature to become a pale suspension. After addition of iodomethane (20 mmol, 4.0 equiv.) the reaction mixture was allowed to slowly warm to rt and stirred overnight. Water was added and the mixture was extracted with Et_2O or EtOAc (depending on the volatility of the product; 3x) and the combined organic layers were dried over $MgSO_4$. The volatiles were removed under reduced pressure and the crude product was purified by column chromatography on silica gel.

General procedure 4 for the synthesis of propargyloxy benzene derivatives (GP4):

According to a modified literature procedure,[5] to a solution of the phenol derivative (20 mmol, 1.0 equiv.) and propargyl bromide (23 mmol, 1.2 equiv.) in acetone (0.5 M), K_2CO_3 (23 mmol, 1.2 equiv.) was added and the mixture was stirred at 60 °C for 24 h. The reaction mixture was

cooled down to rt, filtered to remove the solid and the volatiles removed under reduced pressure. The crude product was purified by column chromatography on silica gel.

General procedure 5 for the synthesis of benzofurans by sigmatropic rearrangement (GP5):

According to a modified literature procedure,[5] to a solution of the propargyloxy benzene starting material (15 mmol. 1.0 equiv.) in *N*,*N*-diethylaniline (0.7 M), cesium fluoride (7.5 mmol, 50 mol%) was added and the mixture was stirred for 24 h at 210 °C. After cooling down to rt, the mixture was diluted with EtOAc and washed with aqueous HCl (1.0 M 3x), brine (1x) and dried over $MgSO_4$. The volatiles were removed under reduced pressure and the crude product was purified by column chromatography on silica gel.

General procedure 6 for the synthesis of salicyladehyde *para*-tosylhydrazones (GP6):

According to a modified literature procedure,[6] the salicyl aldehyde derivative (20 mmol, 1.0 equiv.) was added dropwise to a solution of tosylhydrazine (20 mmol, 1.0 equiv.) in methanol (1.0 M) at 60 °C. The mixture was stirred for 30–60 min until full consumption of the starting material was observed via TLC analysis. The obtained precipitant was washed with *n*-pentane, filtered and dried in vacuo to afford the desired product which was used without further purification.

General procedure 7 for the synthesis of 2-methylbenzofurans from *para*-tosylhydrazones (GP7):

According to a modified literature procedure,[7] calcium carbide (30 mmol, 3.0 equiv.) was added to a mixture of the salicylaldehyde *para*-tosylhydrazone (10 mmol, 1.0 equiv.), KOtBu (20 mmol, 2.0 equiv.), cuprous chloride (1.0 mmol, 10 mol%), and water (40 mmol, 4.0 equiv.) in DMF (0.25 M) and the resulting mixture stirred for 6 h at 90 °C. After the complete consumption of the starting material the reaction mixture was filtered, the filtrate extracted with EtOAc (3x) and the combined organic fractions washed with brine (3x). After drying over $MgSO_4$ the volatiles were removed under reduced pressure and the crude product purified by column chromatography on silica gel.

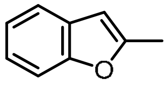

2-methylbenzofuran (1a): The title compound was synthesized on a 20.0 mmol scale according to **GP3** using *t*-butyl lithium (1.9 M in *n*-hexane). The product eluted using pure *n*-pentane and was obtained as pale yellow oil (2.31 g, 17.4 mmol, 87%). Analytical data is in accordance with the literature.[9]

^{1}H NMR (300 MHz, $CDCl_3$) δ 7.51–7.37 (m, 2H), 7.24–7.14 (m, 2H), 6.37 (s, 1H), 2.46 (d, *J* = 1.1 Hz, 3H). **GC-MS** (EI): 132 (63) $[M]^+$, 131 (100) $[M\text{-}H]^+$, 103 (24), 77 (33).

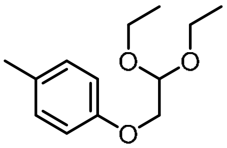

1-(2,2-diethoxyethoxy)-4-methylbenzene: The title compound was synthesized on a 100 mmol scale according to **GP1**. The product was filtered through a short silica plug using pure *n*-pentane (12.2 g, 54.4 mmol, 54%) and was used without further purification.

^{1}H NMR (300 MHz, $CDCl_3$) δ 7.07 (d, *J* = 8.1 Hz, 2H), 6.82 (d, *J* = 8.6 Hz, 1H), 4.83 (t, *J* = 5.3 Hz, 1H), 3.98 (d, *J* = 5.2 Hz, 2H), 3.82–3.52 (m, 4H), 2.28 (s, 3H), 1.27–1.23 (m, 6H).

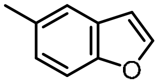

5-methylbenzofuran: The title compound was synthesized on a 54.0 mmol scale according to **GP2**. The product eluted using pure *n*-pentane and was obtained as colorless oil (1.19 g, 8.99 mmol, 30%).

^{1}H NMR (500 MHz, $CDCl_3$) δ 7.58 (d, *J* = 2.2 Hz, 1H), 7.41–7.36 (m, 2H), 7.11 (dd, *J* = 8.5, 1.7 Hz, 1H), 6.70 (dd, *J* = 2.2, 0.9 Hz, 1H), 2.45 (s, 3H). **^{13}C{^{1}H} NMR** (126 MHz, $CDCl_3$) δ 153.5, 145.1, 132.3, 127.6, 125.6, 121.1, 111.0, 106.4, 21.4.

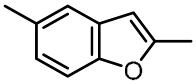

2,5-dimethylbenzofuran (1b): The title compound was synthesized on a 2.00 mmol scale according to **GP3**. The product eluted using pure *n*-pentane and was obtained as colorless oil (241 mg, 1.65 mmol, 82%).

^{1}H NMR (400 MHz, $CDCl_3$) δ 7.31–7.22 (m, 2H), 7.00 (dd, *J* = 8.3, 1.8 Hz, 1H), 6.32–6.27 (m, 1H), 2.43 (d, *J* = 1.1 Hz, 3H), 2.42 (s, 3H). **^{13}C{^{1}H} NMR** (151 MHz, $CDCl_3$) δ 155.6, 153.3, 131.9, 129.4, 124.3, 120.1, 110.2, 102.5, 21.4, 14.2. **HRMS** (EI) m/z calculated for $[C_{10}H_{10}O]$ ($[M]^{+\cdot}$) 146.0726, found 146.0716; m/z calculated for $[C_{10}H_9O]$ ($[M\text{-}H]^{+\cdot}$) 145.0648, found 145.0648.

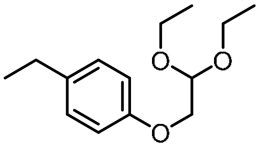

1-(2,2-diethoxyethoxy)-4-ethylbenzene: The title compound was synthesized on a 40.0 mmol scale according to **GP1**. The product was filtered through a short silica plug using pure *n*-pentane (5.49 g, 23.0 mmol, 58%) and was used without further purification.

^{1}H NMR (300 MHz, $CDCl_3$) *δ* 7.12–7.08 (m, 2H), 6.89–6.80 (m, 2H), 4.83 (t, *J* = 5.2 Hz, 1H), 3.99 (d, *J* = 5.2 Hz, 2H), 3.82–3.55 (m, 4H), 2.58 (q, *J* = 7.6 Hz, 2H), 1.28–1.17 (m, 9H).

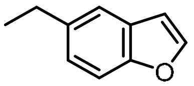

5-ethylbenzofuran: The title compound was synthesized on a 23.0 mmol scale according to **GP2**. The product eluted using a gradient of *n*-pentane/EtOAc (100:0 to 95:5, *v*:*v*) and was obtained as colorless oil (1.36 g, 9.28 mmol, 40%).

^{1}H NMR (400 MHz, $CDCl_3$) *δ* 7.59 (d, *J* = 2.2 Hz, 1H), 7.45–7.40 (m, 2H), 7.14 (dd, *J* = 8.4, 1.7 Hz, 1H), 6.72 (dd, *J* = 2.2, 1.0 Hz, 1H), 2.75 (q, *J* = 7.6 Hz, 2H), 1.29 (t, *J* = 7.6 Hz, 3H). **$^{13}C\{^1H\}$ NMR** (101 MHz, $CDCl_3$) *δ* 153.6, 145.2, 138.9, 127.6, 124.6, 119.9, 111.1, 106.5, 29.0, 16.5.

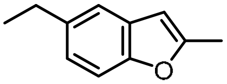

5-ethyl-2-methylbenzofuran (1c): The title compound was synthesized on a 4.00 mmol scale according to **GP3**. The product eluted using pure *n*-pentane and was obtained as colorless oil (569 mg, 3.55 mmol, 89%).

^{1}H NMR (600 MHz, $CDCl_3$) δ 7.32–7.27 (m, 2H), 7.04 (dd, *J* = 8.3, 1.9 Hz, 1H), 6.31 (p, *J* = 1.1 Hz, 1H), 2.72 (q, *J* = 7.6 Hz, 2H), 2.44 (d, *J* = 1.1 Hz, 3H), 1.27 (t, *J* = 7.6 Hz, 3H). **$^{13}C\{^1H\}$ NMR** (151 MHz, $CDCl_3$) δ 155.6, 153.4, 138.6, 129.4, 123.3, 118.9, 110.3, 102.6, 29.0, 16.5, 14.2. **HRMS** (EI) m/z calculated for [$C_{11}H_{12}O$] ($[M]^{+\cdot}$) 160.0883, found 160.0883.

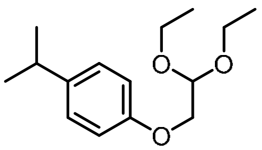

1-(2,2-diethoxyethoxy)-4-*iso*propylbenzene: The title compound was synthesized on a 20.0 mmol scale according to **GP1**. The product was filtered through a short silica plug using *n*-pentane/EtOAc (95:5, *v*:*v*), was obtained as colorless oil (2.93 g, 11.6 mmol, 58%) and used without further purification.

^{1}H NMR (400 MHz, $CDCl_3$) δ 7.18–7.07 (m, 2H), 6.91–6.81 (m, 2H), 4.83 (t, *J* = 5.2 Hz, 1H), 3.99 (d, *J* = 5.2 Hz, 2H), 3.80–3.56 (m, 4H), 2.85 (hept, *J* = 7.0 Hz, 1H), 1.28–1.18 (m, 12H).

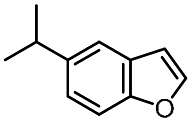

5-*iso*propylbenzofuran: The title compound was synthesized on a 11.6 mmol scale according to **GP2**. The product eluted using *n*-pentane/EtOAc (95:5, *v*:*v*) and was obtained as colorless oil (1.17 g, 7.31 mmol, 63%).

^{1}H NMR (400 MHz, $CDCl_3$) δ 7.59 (d, *J* = 2.2 Hz, 1H), 7.46–7.39 (m, 2H), 7.18 (dd, *J* = 8.5, 1.9 Hz, 1H), 6.73 (dd, *J* = 2.2, 1.0 Hz, 1H), 3.02 (hept, *J* = 6.9 Hz, 1H), 1.30 (d, *J* = 6.9 Hz, 6H). **^{13}C{^{1}H} NMR** (101 MHz, $CDCl_3$) δ 153.6, 145.2, 143.7, 127.5, 123.3, 118.4, 111.1, 106.6, 34.2, 24.7.

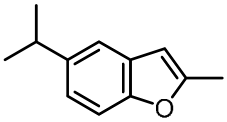

5-*iso*propyl-2-methylbenzofuran (1d): The title compound was synthesized on a 7.30 mmol scale according to **GP3** using *t*-butyl lithium (1.9 M in *n*-hexane). The product eluted using *n*-pentane/EtOAc (99.5:0.5, *v*:*v*) and was obtained as colorless oil (1.10 g, 6.34 mmol, 88%).

^{1}H NMR (400 MHz, $CDCl_3$) δ 7.34–7.28 (m, 2H), 7.07 (dd, *J* = 8.5, 1.8 Hz, 1H), 6.32 (s, 1H), 2.98 (hept, *J* = 6.9 Hz, 1H), 2.44 (d, *J* = 1.1 Hz, 3H), 1.28 (d, *J* = 6.9 Hz, 6H). **^{13}C{^{1}H} NMR** (101 MHz, $CDCl_3$) δ 155.7, 153.4, 143.3, 129.3, 122.0, 117.4, 110.3, 102.6, 34.2, 24.7, 14.2. **HRMS** (EI) m/z calculated for [$C_{12}H_{14}O$] ($[M]^{+\cdot}$) 174.1039, found 174.1039.

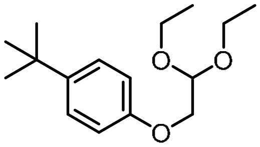

4-*tert*butyl-1-(2,2-diethoxyethoxy)benzene: The title compound was synthesized on a 20.0 mmol scale according to **GP1**. The product was filtered through a short silica plug using *n*-pentane/EtOAc (95:5, *v*:*v*), was obtained as colorless oil (3.52 g, 13.2 mmol, 66%) and used without further purification.

^{1}H NMR (400 MHz, $CDCl_3$) δ 7.32–7.27 (m, 2H), 6.89–6.84 (m, 2H), 4.83 (t, *J* = 5.2 Hz, 1H), 3.99 (d, *J* = 5.2 Hz, 2H), 3.81–3.59 (m, 4H), 1.29 (s, 9H), 1.25 (t, *J* = 7.0 Hz, 6H).

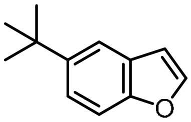

5-*tert*butylbenzofuran: The title compound was synthesized on a 13.2 mmol scale according to **GP2**. The product eluted using *n*-pentane/EtOAc (95:5, *v*:*v*) and was obtained as colorless oil (1.88 g, 10.8 mmol, 82%).

^{1}H NMR (400 MHz, $CDCl_3$) δ 7.62–7.57 (m, 2H), 7.46–7.41 (m, 1H), 7.40–7.34 (m, 1H), 6.76–6.72 (m, 1H), 1.38 (s, 9H). **^{13}C{^{1}H} NMR** (101 MHz, $CDCl_3$) δ 153.3, 145.9, 145.2, 127.2, 122.3, 117.4, 110.8, 106.8, 34.8, 32.0.

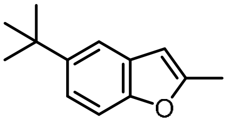

5-(*tert*butyl)-2-methylbenzofuran (1e): The title compound was synthesized on a 9.40 mmol scale according to **GP3** using *t*-butyl lithium (1.9 M in *n*-hexane). The product eluted using *n*-pentane/EtOAc (99:1, *v*:*v*) and was obtained as colorless oil (1.25 g, 6.64 mmol, 71%).

^{1}H NMR (400 MHz, $CDCl_3$) δ 7.50–7.45 (m, 1H), 7.34–7.30 (m, 1H), 7.29–7.22 (m, 1H), 6.34–6.32 (m, 1H), 2.44 (d, J = 1.1 Hz, 3H), 1.37 (s, 9H). **^{13}C{^{1}H} NMR** (101 MHz, $CDCl_3$) δ 155.6, 153.1, 145.6, 129.0, 121.0, 116.5, 110.0, 102.8, 34.8, 32.0, 14.2. **HRMS** (EI) m/z calculated for [$C_{13}H_{16}O$] ([M]$^{+\cdot}$) 188.1196, found 188.1194.

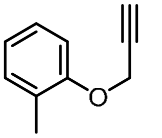

2-methyl-1-(prop-2-ynyloxy)benzene: The title compound was synthesized on a 10.0 mmol scale according to **GP4**. The product eluted using a gradient of *n*-pentane/EtOAc (100:0 to 95:5, *v*:*v*) and was obtained as colorless oil (1.17 g, 8.03 mmol, 80%).

^{1}H NMR (600 MHz, $CDCl_3$) δ 7.19–7.14 (m, 2H), 6.95 (dd, J = 8.0, 1.1 Hz, 1H), 6.91 (td, J = 7.4, 1.1 Hz, 1H), 4.72–4.71 (m, 2H), 2.50 (t, J = 2.4 Hz, 1H), 2.25 (s, 3H). **^{13}C{^{1}H} NMR** (151 MHz, $CDCl_3$) δ 155.9, 131.0, 127.4, 126.8, 121.4, 111.9, 79.1, 75.3, 56.1, 16.4.

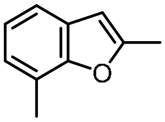

2,7-dimethylbenzofuran (1f): The title compound was synthesized on a 8.00 mmol scale according to **GP5**. The product eluted using a gradient of *n*-pentane/EtOAc (100:0 to 99:1, *v*:*v*) and was obtained as colorless oil (625 mg, 4.28 mmol, 53%).

^{1}H NMR (400 MHz, $CDCl_3$) δ 7.32–7.27 (m, 1H), 7.07 (t, J = 7.5 Hz, 1H), 7.01–6.98 (m, 1H), 6.36–6.34 (m, 1H), 2.50 (d, J = 0.8 Hz, 3H), 2.47 (d, J = 1.1 Hz, 3H). **^{13}C{^{1}H} NMR** (101 MHz,

CDCl3) δ 155.2, 153.9, 128.8, 124.2, 122.5, 121.0, 117.7, 102.9, 15.2, 14.3. **HRMS** (EI) m/z calculated for [$C_{10}H_{10}O$] ($[M]^{+\cdot}$) 146.0726, found 146.0718.

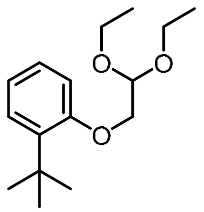

2-*tert*butyl-1-(2,2-diethoxyethoxy)benzene: The title compound was synthesized on a 20.0 mmol scale according to **GP1**. The product obtained after filtration through a short silica plug using pure EtOAc as eluent. After removing the volatiles under educed pressure the product was directly used without further purification.

^{1}H NMR (300 MHz, $CDCl_3$) δ 7.32–7.27 (m, 1H), 7.20–7.12 (m, 1H), 6.94–6.81 (m, 2H), 4.95 (t, *J* = 5.4 Hz, 1H), 4.02 (d, *J* = 5.4 Hz, 2H), 3.84–3.58 (m, 4H), 1.40 (s, 9H), 1.25 (t, *J* = 7.0 Hz, 6H).

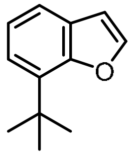

7-*tert*butylbenzofuran: The title compound was synthesized on a 7.00 mmol scale according to **GP2**. The product eluted using *n*-pentane/EtOAc (95:5, *v*:*v*) and was obtained as colorless oil (854 mg, 4.90 mmol, 70%).

^{1}H NMR (400 MHz, $CDCl_3$) δ 7.64 (d, *J* = 2.2 Hz, 1H), 7.46 (dd, *J* = 7.0, 1.9 Hz, 1H), 7.22–7.14 (m, 2H), 6.76 (d, *J* = 2.2 Hz, 1H), 1.51 (s, 9H). **^{13}C{^{1}H} NMR** (101 MHz, $CDCl_3$) δ 153.4, 144.1, 135.1, 128.0, 122.8, 120.9, 119.3, 106.5, 34.5, 30.0.

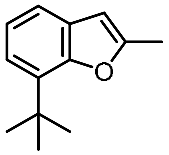

7-(*tert*butyl)-2-methylbenzofuran (1g): The title compound was synthesized on a 4.50 mmol scale according to **GP3** using *t*-butyl lithium (1.9 M in *n*-hexane). The product eluted using *n*-pentane/EtOAc (99.5:0.5, *v*:*v*) and was obtained as colorless oil (593 mg, 3.15 mmol, 70%).

^{1}H NMR (400 MHz, $CDCl_3$) δ 7.34–7.30 (m, 1H), 7.11–7.09 (m, 2H), 6.34 (d, *J* = 1.1 Hz, 1H), 2.47 (d, *J* = 1.1 Hz, 3H), 1.50 (s, 9H). **^{13}C{^{1}H} NMR** (101 MHz, $CDCl_3$) δ 154.5, 134.4, 129.8,

122.4, 119.8, 118.2, 102.5, 34.4, 30.0, 14.4, one carbon missing. **HRMS** (EI) m/z calculated for [$C_{13}H_{16}O$] ([M]$^{+\cdot}$) 188.1196, found 188.1196.

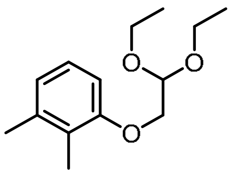

1-(2,2-diethoxyethoxy)-2,3-dimethylbenzene: The title compound was synthesized on a 20.0 mmol scale according to **GP1**. The product eluted using *n*-pentane/EtOAc (95:5, *v*:*v*) and was obtained as colorless oil (3.10 g, 13.0 mmol, 65%).

^{1}H NMR (400 MHz, $CDCl_3$) δ 7.03 (t, *J* = 7.9 Hz, 1H), 6.79 (d, *J* = 7.5 Hz, 1H), 6.70 (d, *J* = 8.1 Hz, 1H), 4.86 (t, *J* = 5.3 Hz, 1H), 3.99 (d, *J* = 5.3 Hz, 2H), 3.78 (dq, *J* = 9.3, 7.0 Hz, 2H), 3.66 (dq, *J* = 9.3, 7.0 Hz, 2H), 2.27 (s, 3H), 2.16 (s, 3H), 1.26 (t, *J* = 7.0 Hz, 6H). **^{13}C{^{1}H} NMR** (101 MHz, $CDCl_3$) δ 156.7, 138.1, 125.9, 125.5, 122.7, 109.3, 100.9, 69.3, 62.8, 20.2, 15.5, 11.8.

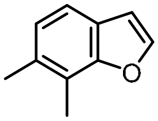

6,7-dimethylbenzofuran: The title compound was synthesized on a 19.5 mmol scale according to **GP2**. The product eluted using *n*-pentane/EtOAc (95:5, *v*:*v*) and was obtained as colorless oil (2.00 g, 13.7 mmol, 70%).

^{1}H NMR (400 MHz, $CDCl_3$) δ 7.57 (d, *J* = 2.2 Hz, 1H), 7.32 (d, *J* = 7.8 Hz, 1H), 7.06 (d, *J* = 7.8 Hz, 1H), 6.71 (d, *J* = 2.2 Hz, 1H), 2.45 (s, 3H), 2.39 (s, 3H). **^{13}C{^{1}H} NMR** (101 MHz, $CDCl_3$) δ 154.7, 144.3, 132.6, 125.0, 124.8, 120.1, 117.7, 106.8, 19.3, 11.8.

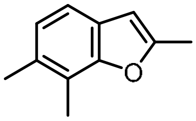

2,6,7-trimethylbenzofuran (1h): The title compound was synthesized on a 6.00 mmol scale according to **GP3** using *t*-butyl lithium (1.9 M in *n*-hexane). The product eluted using *n*-pentane/EtOAc (99.5:0.5, *v*:*v*) and was obtained as colorless oil (673 mg, 4.20 mmol, 70%).

^{1}H NMR (400 MHz, $CDCl_3$) δ 7.18 (d, *J* = 7.8 Hz, 1H), 6.98 (d, *J* = 7.8 Hz, 1H), 6.30 (q, *J* = 1.1 Hz, 1H), 2.44 (d, *J* = 1.1 Hz, 3H), 2.41 (s, 3H), 2.36 (s, 3H). **^{13}C{^{1}H} NMR** (101 MHz, $CDCl_3$) δ 154.7, 154.4, 131.4, 126.6, 124.5, 119.4, 116.7, 102.8, 19.3, 14.3, 11.8. **HRMS** (EI) m/z calculated for [$C_{11}H_{11}O$] ([M-H]$^{+\cdot}$) 159.0804, found 159.0805.

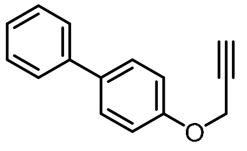

4-(prop-2-ynyloxy)-1,1'-biphenyl: The title compound was synthesized on a 10.0 mmol scale according to **GP4**. The product eluted using a gradient of *n*-pentane/EtOAc (20:1 to 4:1, *v*:*v*) and was obtained as off-white solid (1.95 g, 9.36 mmol, 94%).

^{1}H NMR (400 MHz, $CDCl_3$) δ 7.60–7.51 (m, 4H), 7.43 (t, *J* = 7.6 Hz, 2H), 7.36–7.28 (m, 1H), 7.06 (d, *J* = 8.7 Hz, 2H), 4.74 (d, *J* = 2.4 Hz, 2H), 2.55 (t, *J* = 2.4 Hz, 1H). **^{13}C{^{1}H} NMR** (101 MHz, $CDCl_3$) δ 157.2, 140.8, 134.8, 128.9, 128.3, 127.0, 126.9, 115.3, 78.7, 75.7, 56.0.

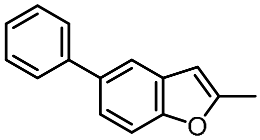

2-methyl-5-phenylbenzofuran (1i): The title compound was synthesized on a 4.00 mmol scale according to **GP5**. The product eluted using a mixture of *n*-pentane/EtOAc (50:1, *v*:*v*) and was obtained as off-white solid (552 mg, 2.65 mmol, 66%).

^{1}H NMR (400 MHz, $CDCl_3$) δ 7.69–7.66 (m, 1H), 7.64–7.60 (m, 2H), 7.48–7.41 (m, 4H), 7.38–7.31 (m, 1H), 6.42 (s, 1H), 2.49 (d, *J* = 1.1 Hz, 3H). **^{13}C{^{1}H} NMR** (101 MHz, $CDCl_3$) δ 156.3, 154.5, 142.0, 136.3, 129.9, 128.8, 127.6, 126.9, 122.9, 118.8, 110.8, 102.9, 14.3. **HRMS** (EI) m/z calculated for [$C_{15}H_{12}O$] ($[M]^{+\cdot}$) 208.0883, found 208.0871.

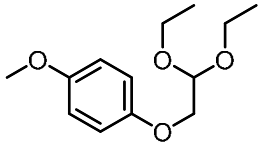

1-(2,2-diethoxyethoxy)-4-methoxybenzene: The title compound was synthesized on a 20.0 mmol scale according to **GP1**. The product eluted using a gradient of *n*-pentane/EtOAc (95:5 to 90:10, *v*:*v*) and was obtained as colorless oil (2.97 g, 12.4 mmol, 62%).

^{1}H NMR (300 MHz, $CDCl_3$) δ 6.91–6.78 (m, 4H), 4.81 (t, *J* = 5.2 Hz, 1H), 3.96 (d, *J* = 5.2 Hz, 2H), 3.82–3.70 (m, 2H), 3.76 (s, 3H), 3.69–3.57 (m, 2H), 1.25 (t, *J* = 7.1 Hz, 6H). **^{13}C{^{1}H} NMR** (101 MHz, $CDCl_3$) δ 154.1, 152.9, 115.8, 114.7, 100.7, 69.4, 62.6, 55.8, 15.5.

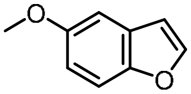

5-methoxybenzofuran: The title compound was synthesized on a 12.3 mmol scale according to **GP2**. The product eluted using a gradient of *n*-pentane/EtOAc (99:1 to 97:3, *v*:*v*) and was obtained as colorless oil (433 mg, 2.92 mmol, 24%).

^{1}H NMR (400 MHz, $CDCl_3$) δ 7.60 (d, *J* = 2.2 Hz, 1H), 7.39 (dd, *J* = 8.9, 0.7 Hz, 1H), 7.06 (d, *J* = 2.6 Hz, 1H), 6.91 (dd, *J* = 8.9, 2.6 Hz, 1H), 6.71 (dd, *J* = 2.2, 1.0 Hz, 1H), 3.85 (s, 3H). **^{13}C{^{1}H} NMR** (101 MHz, $CDCl_3$) δ 156.1, 150.1, 145.9, 128.1, 113.2, 111.9, 106.8, 103.6, 56.1.

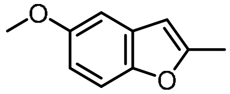

5-methoxy-2-methylbenzofuran (1j): The title compound was synthesized on a 2.90 mmol scale according to **GP3** using *t*-butyl lithium (1.9 M in *n*-hexane). The product eluted using *n*-pentane/EtOAc (99:1 to 95:5, *v*:*v*) and was obtained as colorless oil (413 mg, 2.55 mmol, 88%).

^{1}H NMR (300 MHz, $CDCl_3$) δ 7.30–7.26 (m, 1H), 6.95 (d, *J* = 2.6 Hz, 1H), 6.80 (dd, *J* = 8.9, 2.6 Hz, 1H), 6.32–6.29 (m, 1H), 3.83 (s, 3H), 2.43 (d, *J* = 1.1 Hz, 3H). **^{13}C{^{1}H} NMR** (101 MHz, $CDCl_3$) δ 156.4, 155.9, 149.8, 129.9, 111.4, 111.1, 103.2, 102.9, 56.1, 14.3. **HRMS** (ESI) m/z calculated for $[C_{10}H_{10}O_2Na]$ ($[M+Na]^+$) 185.057, found 185.060.

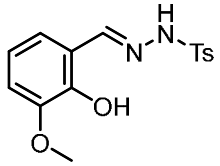

2-((2-(*para*-tolyl)hydrazinylidene)methyl)-6-methoxyphenol: The title compound was synthesized on a 7.00 mmol scale according to **GP6**. The crude product was purified by washing with *n*-pentane and was obtained as white, crystalline solid (1.90 g, 5.94 mmol, 85%).

^{1}H NMR (300 MHz, DMSO) δ 11.44 (s, 1H), 9.67 (s, 1H), 8.17 (s, 1H), 7.73 (d, *J* = 8.3 Hz, 2H), 7.42 (d, *J* = 8.1 Hz, 2H), 7.05 (dd, *J* = 7.9, 1.5 Hz, 1H), 6.97 (dd, *J* = 8.1, 1.5 Hz, 1H), 6.77 (t, *J* = 7.9 Hz, 1H), 3.77 (s, 3H), 2.36 (s, 3H).

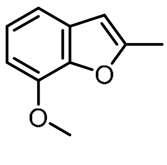

7-methoxy-2-methylbenzofuran (1k): The title compound was synthesized on a 5.50 mmol scale according to **GP3**. The product eluted using *n*-pentane/EtOAc (98:2, *v*:*v*) and was obtained as colorless oil (370 mg, 2.30 mmol, 42%).

^{1}H NMR (300 MHz, $CDCl_3$) *δ* 7.14–7.05 (m, 2H), 6.74 (dd, *J* = 7.0, 2.0 Hz, 1H), 6.37 (q, *J* = 1.1 Hz, 1H), 4.00 (s, 3H), 2.47 (d, *J* = 1.1 Hz, 3H). **^{13}C{^{1}H} NMR** (75 MHz, $CDCl_3$) *δ* 155.7, 145.0, 143.9, 130.9, 123.2, 112.8, 105.4, 103.1, 56.0, 14.2. **HRMS** (EI) m/z calculated for [$C_{10}H_{10}O_2$] ($[M]^{+\cdot}$) 162.0675, found 162.0674.

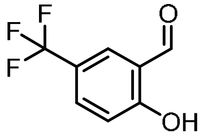

2-hydroxy-5-(trifluoromethyl)benzaldehyde: According to a modified literature procedure,[8] hexamethylenetetramine (2.15 g, 15.5 mmol, 1.03 equiv.) was added to a solution of 4-trifluoromethylphenol (2.43 g, 15.0 mmol, 1.00 equiv.) in trifluoroacetic acid (0.2 M) and the resulting solution was stirred for 16 h at 80 °C. After cooling down to rt the mixture was diluted two-fold with water and subsequently cooled with an ice bath to rt again. After extraction with DCM (4x) the combined organic fractions were dried over Na_2SO_4 and the volatiles removed under reduced pressure. The crude product was purified by column chromatography on silica gel (eluent: *n*-pentane/EtOAc, 99:1, *v*:*v*) and the desired product obtained as white, crystalline solid (1.25 g, 6.56 mmol, 44%).

^{1}H NMR (500 MHz, $CDCl_3$) *δ* 9.95 (d, *J* = 0.6 Hz, 1H), 7.86 (ddd, *J* = 2.0, 0.9, 0.4 Hz, 1H), 7.76 (dd, *J* = 8.8, 2.2 Hz, 1H), 7.11 (dd, *J* = 8.8, 0.7 Hz, 1H), O*H* missing. **^{13}C{^{1}H} NMR** (126 MHz, $CDCl_3$) *δ* 196.0, 164.1, 133.6 (q, *J* = 3.3 Hz), 131.2 (q, *J* = 3.9 Hz), 125.1, 123.0–122.2 (m), 120.1, 118.8. **^{13}C{^{1}H,^{19}F} NMR** (126 MHz, $CDCl_3$) *δ* 195.9, 164.1, 133.5, 131.2, 123.7, 122.7, 120.1, 118.8. **^{19}F{^{1}H} NMR** (470 MHz, $CDCl_3$) *δ* −62.1.

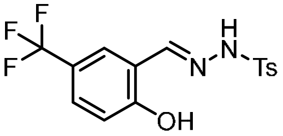

2-((2-(*para*-tolyl)hydrazinylidene)methyl)-4-(trifluoromethyl)phenol: The title compound was synthesized on a 6.00 mmol scale according to **GP6**. The crude product was purified by washing with *n*-pentane and was obtained as white, crystalline solid (1.24 g, 3.46 mmol, 58%).

^{1}H NMR (500 MHz, $CDCl_3$) *δ* 10.52 (s, 1H), 7.96 (s, 1H), 7.87–7.82 (m, 3H), 7.52 (dd, *J* = 8.7, 2.3 Hz, 1H), 7.41 (d, *J* = 2.0 Hz, 1H), 7.36 (dd, *J* = 8.6, 0.8 Hz, 2H), 7.03 (d, *J* = 8.7 Hz, 1H), 2.43 (s, 3H). **^{13}C{^{1}H,^{19}F} NMR** (126 MHz, $CDCl_3$) *δ* 169.2, 160.6, 150.5, 145.4, 134.4, 130.3, 129.1, 128.4, 128.0, 124.0, 118.0, 116.9, 21.8. **^{19}F{^{1}H} NMR** (470 MHz, $CDCl_3$) *δ* −61.8.

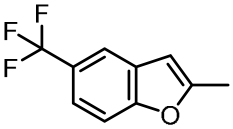

2-methyl-5-(trifluoromethyl)benzofuran (1l): The title compound was synthesized on a 3.40 mmol scale according to **GP7**. The product eluted using pure *n*-pentane and was obtained as colorless oil (99.8 mg, 0.499 mmol, 15%).

^{1}H NMR (300 MHz, $CDCl_3$) δ 7.76 (s, 1H), 7.49–7.43 (m, 2H), 6.44 (s, 1H), 2.49 (d, J = 1.1 Hz, 3H). **^{13}C{^{1}H} NMR** (101 MHz, $CDCl_3$) δ 157.7, 156.2, 129.4, 125.3 (d, J = 31.9 Hz), 124.9 (d, J = 271.8 Hz), 120.4 (q, J = 3.7 Hz), 117.8 (q, J = 4.1 Hz), 111.0, 103.0, 14.3. **^{13}C{^{1}H,^{19}F} NMR** (126 MHz, $CDCl_3$) δ 157.7, 156.2, 129.4, 125.3, 124.9, 120.4, 117.8, 111.0, 103.0, 14.3. **^{19}F{^{1}H} NMR** (470 MHz, $CDCl_3$) δ −60.8. **HRMS** (EI) m/z calculated for [$C_{10}H_6OF_3$] ([M-H]$^{+\cdot}$) 199.0365, found 199.0362.

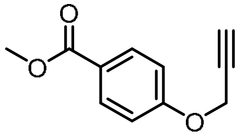

methyl 4-(prop-2-yn-1-yloxy)benzoate: The title compound was synthesized on a 10.0 mmol scale according to **GP4**. The product eluted using a mixture of *n*-pentane/EtOAc (9:1, *v*:*v*) and was obtained as off-white solid (1.90 g, 9.99 mmol, quant.).

^{1}H NMR (300 MHz, $CDCl_3$) δ 8.02–7.95 (m, 2H), 7.04–6.93 (m, 2H), 4.73 (d, J = 2.5 Hz, 2H), 3.87 (s, 3H), 2.55 (t, J = 2.4 Hz, 1H). **^{13}C{^{1}H} NMR** (75 MHz, $CDCl_3$) δ 166.7, 161.2, 131.6, 123.5, 114.5, 77.9, 76.2, 55.9, 52.0.

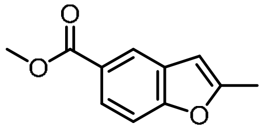

methyl 2-methylbenzofuran-5-carboxylate (1m): The title compound was synthesized on a 10.0 mmol scale according to **GP5**. The product eluted using a gradient of *n*-pentane/EtOAc (30:1 to 20:1, *v*:*v*) and was obtained as colorless oil (568 mg, 3.00 mmol, 30%).

^{1}H NMR (400 MHz, $CDCl_3$) δ 8.20 (d, J = 1.8 Hz, 1H), 7.93 (dd, J = 8.6, 1.8 Hz, 1H), 7.43–7.38 (m, 1H), 6.44–6.41 (m, 1H), 3.92 (s, 3H), 2.47 (d, J = 1.1 Hz, 3H). **^{13}C{^{1}H} NMR** (101 MHz, $CDCl_3$) δ 167.6, 157.5, 157.1, 129.3, 125.2, 124.9, 122.6, 110.6, 103.2, 52.2, 14.2. **HRMS** (ESI) m/z calculated for [$C_{11}H_{10}O_3Na$] ([M+Na]$^{+}$) 213.0522, found 213.0521.

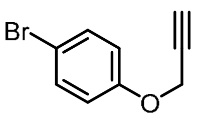

1-bromo-4-(prop-2-ynyloxy)-benzene: The title compound was synthesized on a 30.0 mmol scale according to **GP4**. The product eluted using a mixture of *n*-pentane/EtOAc (9:1, *v*:*v*) and was obtained as pale yellow oil (6.32 g, 29.9 mmol, quant.).

^{1}H NMR (400 MHz, $CDCl_3$) δ 7.44–7.36 (m, 2H), 6.90–6.83 (m, 2H), 4.67 (d, *J* = 2.4 Hz, 2H), 2.53 (t, *J* = 2.4 Hz, 1H). **^{13}C{^{1}H} NMR** (101 MHz, $CDCl_3$) δ 156.7, 132.5, 116.9, 114.0, 78.2, 76.0, 56.1.

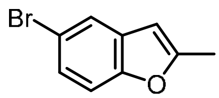

5-bromo-2-methylbenzofuran: The title compound was synthesized on a 30.0 mmol scale according to **GP5**. The product was filtered through a short silica plug (*n*-pentane/EtOAc, 9:1, *v*:*v*), obtained as colorless oil and used without further purification (2.88 g, 13.6 mmol, 45%).

^{1}H NMR (400 MHz, $CDCl_3$) δ 7.57 (d, *J* = 1.8 Hz, 1H), 7.30–7.22 (m, 3H), 6.31 (d, *J* = 1.1 Hz, 1H), 2.44 (s, 3H). **^{13}C{^{1}H} NMR** (101 MHz, $CDCl_3$) δ 157.1, 153.6, 131.4, 126.0, 122.9, 115.6, 112.2, 102.3, 14.2.

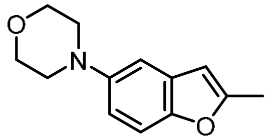

4-(2-methylbenzofuran-5-yl)morpholine (1n): Morpholine (326 µL, 329 mg, 3.78 mmol, 1.20 equiv.) was added to a solution of 5-bromo-2-methylbenzofuran (665 mg, 3.15 mmol, 1.00 equiv.), palladium acetate (14.1 mg, 63.0 µmol, 2 mol%), JohnPhos (38.0 mg, 0.127 mmol, 4 mol%), sodium *tert*-butanolate (424 mg, 4.41 mmol, 1.40 equiv.) in toluene (0.1 M) in a dry Schlenk tube. The resulting mixture was stirred for 21 h at 40 °C. After cooling to rt, 40 mL Et_2O were added and the formation of a precipitant observed. The suspension was filtered through diatomite, the volatiles removed under reduced pressure and the crude product purified by column chromatography on silica gel (eluent: *n*-pentane/EtOAc, 6:1 to 4:1, *v*:*v*) and the title compound was obtained as white, crystalline solid (359 mg, 1.67 mmol, 53%).

^{1}H NMR (400 MHz, $CDCl_3$) δ 7.30 (d, *J* = 8.9 Hz, 1H), 7.02–6.97 (m, 1H), 6.88 (dd, *J* = 8.9, 2.5 Hz, 1H), 6.33–6.28 (m, 1H), 3.92–3.86 (m, 5H), 3.16–3.09 (m, 4H), 2.43 (d, *J* = 1.1 Hz, 3H). **^{13}C{^{1}H} NMR** (101 MHz, $CDCl_3$) δ 156.2, 150.3, 148.0, 129.9, 114.4, 110.9, 107.4, 102.8, 67.3, 51.7, 14.3. **HRMS** (ESI) m/z calculated for [$C_{13}H_{15}NO_2H$] ([M+H]$^+$) 218.1176, found 218.1173.

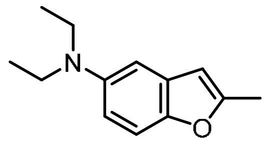

5-diethylamino-2-methylbenzofuran (1o): The title compound was synthesized on a 5.00 mmol scale according to **GP7**. The product eluted using *n*-pentane/EtOAc (95:5 to 90:10, *v*:*v*) and was obtained as white solid (156 mg, 0.767 mmol, 15%).

^{1}H NMR (300 MHz, DMSO) *δ* 7.24 (d, *J* = 8.6 Hz, 1H), 6.71 (d, *J* = 2.2 Hz, 1H), 6.60 (dd, *J* = 8.6, 2.3 Hz, 1H), 6.34–6.31 (m, 1H), 3.32 (q, *J* = 7.0 Hz, 7H), 2.35 (d, *J* = 1.2 Hz, 3H), 1.08 (t, *J* = 7.0 Hz, 6H). **^{13}C{^{1}H} NMR** (101 MHz, DMSO) *δ* 156.3, 151.9, 145.3, 120.1, 117.8, 109.1, 102.1, 94.1, 44.2, 13.7, 12.3. **HRMS** (ESI) m/z calculated for [$C_{13}H_{17}NOH$] ([M+H]$^+$) 204.1383, found 204.1380.

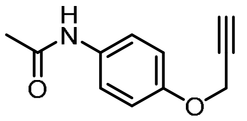

***N*-(4-(prop-2-yn-1-yloxy)phenyl)acetamide**: The title compound was synthesized on a 10.0 mmol scale according to **GP4**. The product eluted using a mixture of *n*-pentane/EtOAc (2:8, *v*:*v*) and was obtained as off-white solid (1.84 g, 9.74 mmol, 97%).

^{1}H NMR (400 MHz, $CDCl_3$) *δ* 7.55 (s, 1H), 7.43–7.37 (m, 2H), 6.94–6.88 (m, 2H), 4.65 (d, *J* = 2.5 Hz, 2H), 2.51 (t, *J* = 2.4 Hz, 1H), 2.13 (s, 3H). **^{13}C{^{1}H} NMR** (101 MHz, $CDCl_3$) *δ* 168.6, 154.4, 132.0, 122.0, 115.4, 78.6, 75.7, 56.2, 24.4.

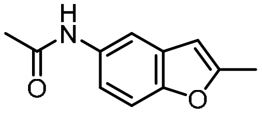

***N*-(2-methylbenzofuran-5-yl)acetamide (1p)**: The title compound was synthesized on a 8.0 mmol scale according to **GP5**. The product eluted using *n*-pentane/EtOAc (1:1, *v*:*v*) and was obtained as white solid (573 mg, 3.03 mmol, 38%).

^{1}H NMR (400 MHz, $CDCl_3$) *δ* 7.73 (d, *J* = 2.2 Hz, 1H), 7.56–7.47 (m, 1H), 7.29 (d, *J* = 8.7 Hz, 1H), 7.12 (dd, *J* = 8.7, 2.2 Hz, 1H), 6.33–6.28 (m, 1H), 2.42 (d, *J* = 1.1 Hz, 3H), 2.16 (s, 3H). **^{13}C{^{1}H} NMR** (101 MHz, $CDCl_3$) *δ* 168.6, 156.6, 151.9, 132.9, 129.7, 116.5, 112.4, 110.7, 103.0, 24.5, 14.2. **HRMS** (ESI) m/z calculated for [$C_{11}H_{11}NO_2Na$] ([M+Na]$^+$) 212.0682, found 212.0678.

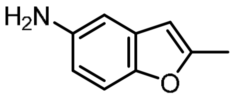

5-amino-2-methylbenzofuran (1q): *N*-(2-methylbenzofuran-5-yl)acetamide **1p** (189 mg, 1.00 mmol, 1.00 equiv.) was dissolved in MeOH (20 mL, 0.05 M). Then 1 mL of a 2 N aqueous HCl solution was added and the mixture was refluxed at 80 °C for 20 h. After cooling down,

the volatiles were removed under reduced pressure. 2 N NaOH was used to neutralize the reaction mixture and it was extracted with DCM (3x). The combined organic fractions were dried over anhydrous $MgSO_4$ and concentrated under reduced pressure. The crude product was purified by column chromatography on silica gel (*n*-pentane/EtOAc 1:1, *v*:*v*). The title compound was obtained as a dark yellow oil (105 mg, 0.713 mmol, 71%).

^{1}H NMR (400 MHz, $CDCl_3$) δ 7.18 (d, *J* = 8.6 Hz, 1H), 6.77 (d, *J* = 2.4 Hz, 1H), 6.59 (dd, *J* = 8.6, 2.3 Hz, 1H), 6.24–6.19 (m, 1H), 3.72 (s, 2H), 2.40 (d, *J* = 1.1 Hz, 3H). **^{13}C{^{1}H} NMR** (101 MHz, $CDCl_3$) δ 156.1, 149.5, 141.7, 130.2, 112.3, 110.9, 105.7, 102.4, 14.3. **HRMS** (ESI) m/z calculated for [C_9H_9NOH] ([M+H]$^+$) 148.0757, found 148.0755.

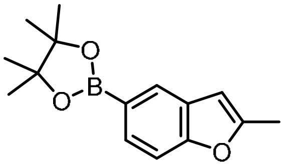

2-methyl-5-(pinacolato)borylbenzofuran (2r): 5-Bromo-2-methylbenzofuran (1.69 g, 8.00 mmol, 1.00 equiv.) was added to a mixture of $Pd(PPh_3)_4$ (462 mg, 5 mol%), potassium acetate (1.57 g, 16.0 mmol, 2.00 equiv.) and bis(pinacolato)diboron (3.11 mL, 3.05 g, 12.0 mmol, 1.50 equiv.) in dioxane (0.5 M) and stirred for 24 h at 80 °C. After full consumption of the starting material the reaction mixture was cooled to rt and water was added. The mixture was extracted with EtOAc (3x), the combined organic layers washed with brine (1x) and dried over $MgSO_4$. The volatiles were removed under reduced pressure and the crude product was purified by column chromatography on silica gel (eluent: *n*-pentane/EtOAc, 100:0 to 30:1 *v*:*v*), followed by three consecutive short-path distillations (Kugelrohr, 0.2 mbar, 190–200 °C) to remove the (pinacolato)boryl benzene by-product to afford the title compound as colorless oil (665 mg, 2.57 mmol, 32%).

^{1}H NMR (400 MHz, $CDCl_3$) δ 7.96 (s, 1H), 7.66 (dd, *J* = 8.2, 1.3 Hz, 1H), 7.39 (d, *J* = 8.2 Hz, 1H), 6.38–6.34 (m, 1H), 2.45 (s, 3H), 1.36 (s, 12H). **^{13}C{^{1}H} NMR** (101 MHz, $CDCl_3$) δ 157.0, 155.6, 129.9, 129.0, 127.5, 110.3, 102.8, 83.8, 25.0, 14.2. C—B signal not detected. **^{11}B{^{1}H} NMR** (128 MHz, $CDCl_3$) δ 31.2. **HRMS** (ESI) m/z calculated for [$C_{15}H_{19}BO_3Na$] ([M+Na]$^+$) 281.1319, found 281.1318.

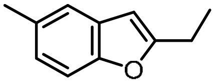

2-ethyl-5-methylbenzofuran (1t): The title compound was synthesized on a 2.00 mmol according to a modified **GP3**. As the haloalkane component bromoethane (872 mg, 8 mmol, 4.00 equiv.) was used. The crude product was purified by column chromatography on silica

gel (pure pentane). The title compound was obtained as a colorless oil (272 mg, 1.70 mmol 87%).

^{1}H NMR (400 MHz, $CDCl_3$) δ 7.33–7.20 (m, 2H), 7.01 (dd, J = 8.6, 1.6 Hz, 1H), 6.30 (td, J = 1.6, 1.1 Hz, 1H), 2.78 (qd, J = 7.5, 1.1 Hz, 2H), 2.42 (s, 3H), 1.33 (t, J = 7.5 Hz, 3H). **^{13}C{^{1}H} NMR** (101 MHz, $CDCl_3$) δ 161.2, 153.2, 131.8, 129.2, 124.3, 120.3, 110.3, 100.9, 22.0, 21.5, 12.0. **GC-MS** (EI): 160 () $[M]^+$, 145 (100) $[M-CH_3]^+$, 115 (19).

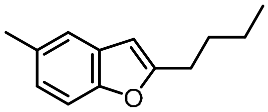

2-butyl-5-methylbenzofuran (1u): The title compound was synthesized on a 2.00 mmol according to a modified **GP3**. 1-Iodobutane (1.47 g, 8.00 mmol, 4.00 equiv.) was used as haloalkane. The crude product was purified by column chromatography on silica gel (pure pentane). The title compound was obtained as a colorless oil (292 mg, 1.55 mmol 78%).

^{1}H NMR (400 MHz, $CDCl_3$) δ 7.31–7.23 (m, 2H), 7.00 (dd, J = 8.4, 1.8 Hz, 1H), 6.29 (td, J = 1.0, 1.0 Hz, 1H), 2.79–2.70 (m, 2H), 2.41 (s, 3H), 1.72 (p, J = 7.5 Hz, 2H), 1.41 (h, J = 7.4 Hz, 2H), 0.95 (t, J = 7.4 Hz, 3H). **^{13}C{^{1}H} NMR** (101 MHz, $CDCl_3$) δ 160.0, 153.1, 131.8, 129.3, 124.3, 120.2, 110.3, 101.6, 29.9, 28.3, 22.4, 21.5, 14.0. **HRMS** (EI) m/z calculated for $[C_{13}H_{16}O]$ ($[M]^+$) 188.1196, found 188.1196.

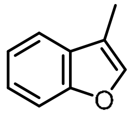

3-methylbenzofuran (1v): The title compound was synthesized following a literature procedure.[10] 2-Hydroxyacetophenone (1.36 g, 10.0 mmol, 1.00 equiv.), ethylbromoacetate (2.34 g, 14 mmol, 1.40 equiv.) and K_2CO_3 (5.53 g, 40 mmol, 4.00 equiv.) were added to 10 mL of dry acetone in a round-bottom flask. The mixture was refluxed for 60 h. After cooling down to rt, the reaction mixture was filtered, the filter cake was washed with EtOAc and the filtrate was concentrated under reduced pressure to afford the crude alkylated intermediate. To the solution of this compound in EtOH (4 mL), 6 mL of an aqueous 3 N NaOH solution was added and the resulting mixture was stirred for 2 h at rt. The solvent was removed under reduced pressure and 2 N aqueous HCl solution was added to adjust the pH to 1–2. The resulting precipitate was collected by filtration and washed with water. The obtained crude product was used without further purification.

A solution of the obtained carboxylic acid (472 mg, 2.40 mmol, 1.00 equiv.) and NaOAc (1.20 g, 14.6 mmol, 6.08 equiv.) in Ac_2O (1.37 mL, 14.6 mmol, 6.08 equiv.) was refluxed for 4 h. After cooling down to rt, the reaction mixture was poured onto ice and extracted with

EtOAc. The combined organic layers were dried over anhydrous $MgSO_4$ and the volatiles removed under reduced pressure. The crude product was purified by column chromatography on silica gel (pure pentane). The title compound was obtained as colorless oil (254 mg, 1.92 mmol, 19% over 3 steps). Analytical data is in accordance with the literature.[9]

^{1}H NMR (400 MHz, $CDCl_3$) δ 7.53 (dd, J = 6.9, 1.4 Hz, 1H), 7.48–7.43 (m, 1H), 7.44–7.37 (m, 1H), 7.33–7.20 (m, 1H), 2.26 (d, J = 1.4 Hz, 3H). **^{13}C{^{1}H} NMR** (101 MHz, $CDCl_3$) δ 155.4, 141.5, 129.1, 124.2, 122.3, 119.5, 115.8, 111.4, 8.0. **GC-MS** (EI): 132 (68) $[M]^+$, 131 (100) $[M\text{-}H]^+$, 103 (29), 77 (19).

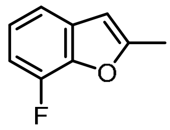

7-fluoro-2-methylbenzofuran (1w): The title compound was synthesized on a 3.00 mmol scale according to **GP3** using *t*-butyl lithium (1.9 M in *n*-hexane). The product eluted using pure *n*-pentane and was obtained as colorless oil (333 mg, 2.22 mmol, 74%).

^{1}H NMR (300 MHz, $CDCl_3$) δ 7.22 (dd, J = 7.7, 1.1 Hz, 1H), 7.09 (td, J = 7.9, 4.4 Hz, 1H), 6.94 (ddd, J = 10.8, 8.0, 1.1 Hz, 1H), 6.41 (dq, J = 3.2, 1.1 Hz, 1H), 2.48 (d, J = 1.1 Hz, 3H). **^{1}H{^{19}F} NMR** (600 MHz, $CDCl_3$) δ 7.22 (dd, J = 7.8, 1.0 Hz, 1H), 7.09 (t, J = 7.9 Hz, 1H), 6.94 (dd, J = 8.1, 1.0 Hz, 1H), 6.41 (q, J = 1.1 Hz, 1H), 2.48 (d, J = 1.1 Hz, 3H). **^{13}C{^{1}H} NMR** (151 MHz, $CDCl_3$) δ 156.7 (d, J = 1.1 Hz), 147.8 (d, J = 248.1 Hz), 141.7 (d, J = 10.9 Hz), 132.9 (d, J = 3.6 Hz), 123.1 (d, J = 6.0 Hz), 115.8 (d, J = 3.7 Hz), 109.6 (d, J = 16.2 Hz), 103.2 (d, J = 2.3 Hz), 14.1. **^{13}C{^{1}H,^{19}F} NMR** (151 MHz, $CDCl_3$) δ 156.7, 147.8, 141.7, 132.9, 123.1, 115.8, 109.6, 103.2, 14.1. **^{19}F{^{1}H} NMR** (282 MHz, $CDCl_3$) δ -138.2. **HRMS** (EI) m/z calculated for $[C_9H_7OF]$ ($[M]^{+\cdot}$) 150.0475, found 150.0472.

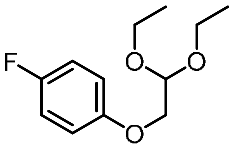

1-(2,2-diethoxyethoxy)-4-fluorobenzene: The title compound was synthesized on a 140 mmol scale according to **GP1**. The product eluted using a gradient of *n*-pentane/EtOAc (29:1 to 19:1, *v*:*v*) and was obtained as colorless oil (24.9 g, 109 mmol, 78%).

^{1}H NMR (300 MHz, $CDCl_3$) δ 7.00–6.91 (m, 2H), 6.89–6.82 (m, 2H), 4.82 (t, J = 5.2 Hz, 1H), 3.97 (d, J = 5.2 Hz, 2H), 3.82–3.57 (m, 4H), 1.25 (t, J = 7.1 Hz, 6H).

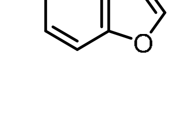

5-fluorobenzofuran: The title compound was synthesized on a 60.0 mmol scale according to **GP2**. The product was filtered through a short silica plug using pure *n*-pentane, obtained as colorless oil (4.90 g, 36.0 mmol, 60%) and was used without further purification.

^{1}H NMR (300 MHz, $CDCl_3$) *δ* 7.66 (d, *J* = 2.2 Hz, 1H), 7.43 (dd, *J* = 8.9, 4.1 Hz, 1H), 7.28–7.22 (m, 1H), 7.02 (td, *J* = 9.1, 2.7 Hz, 1H), 6.75 (dd, *J* = 2.2, 0.9 Hz, 1H).

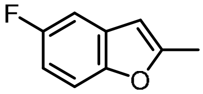

5-fluoro-2-methylbenzofuran (1x): The title compound was synthesized on a 20.0 mmol scale according to **GP3** using *t*-butyl lithium (1.9 M in *n*-hexane). The product eluted using pure *n*-pentane and was obtained as colorless oil (2.41 g, 16.1 mmol, 80%).

^{1}H NMR (500 MHz, $CDCl_3$) *δ* 7.31 (dd, *J* = 8.9, 0.7 Hz, 1H), 7.12 (dd, *J* = 8.7, 2.7 Hz, 1H), 6.91 (td, *J* = 9.1, 2.7 Hz, 1H), 6.35–6.33 (m, 1H), 2.45 (d, *J* = 1.1 Hz, 3H). **^{1}H{^{19}F}NMR** (500 MHz, $CDCl_3$) *δ* 7.31 (d, *J* = 8.9 Hz, 1H), 7.12 (d, *J* = 2.6 Hz, 1H), 6.91 (dd, *J* = 8.8, 2.7 Hz, 1H), 6.35–6.33 (m, 1H), 2.45 (d, *J* = 1.1 Hz, 3H). **^{13}C{^{1}H} NMR** (126 MHz, $CDCl_3$) *δ* 159.3, 157.5, 151.1, 130.1, 111.2, 110.6, 105.8, 103.0, 14.3. **^{13}C{^{1}H,^{19}F } NMR** (126 MHz, $CDCl_3$) *δ* 160.2, 158.3, 157.5, 151.1 (d, *J* = 0.6 Hz), 130.1 (d, *J* = 10.7 Hz), 111.2 (d, *J* = 9.8 Hz), 110.6 (d, *J* = 26.2 Hz), 105.8 (d, *J* = 25.0 Hz), 103.0 (d, *J* = 3.8 Hz), 14.3. **^{19}F{^{1}H} NMR** (470 MHz, $CDCl_3$) *δ* -121.9. **^{19}F NMR** (470 MHz, $CDCl_3$) *δ* -121.9 (td, *J* = 9.0, 4.1 Hz). **HRMS** (EI) m/z calculated for [C_9H_7OF] ($[M]^{+\cdot}$) 150.0475, found 150.0473.

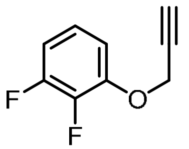

1,2-difluoro-3-(prop-2-yn-1-yloxy)benzene: The title compound was synthesized on a 10.0 mmol scale according to **GP4**. The product eluted using a gradient of *n*-pentane/EtOAc (100:0 to 97:3, *v*:*v*) and was obtained as yellow oil (1.21 g, 7.21 mmol, 72%).

^{1}H NMR (400 MHz, $CDCl_3$) *δ* 7.04–6.96 (m, 1H), 6.92–6.79 (m, 2H), 4.78 (d, *J* = 2.5 Hz, 2H), 2.56 (t, *J* = 2.4 Hz, 1H). **^{13}C{^{1}H} NMR** (101 MHz, $CDCl_3$) *δ* 151.6 (dd, *J* = 247.3, 10.5 Hz), 147.2 (dd, *J* = 7.9, 3.2 Hz), 141.9 (dd, *J* = 248.2, 14.4 Hz), 123.2 (dd, *J* = 8.5, 5.2 Hz), 111.1 (d, *J* = 3.1 Hz), 110.4 (d, *J* = 17.6 Hz), 77.8, 76.6, 57.6. **^{19}F{^{1}H} NMR** (377 MHz, $CDCl_3$) *δ* -137.0 (d, *J* = 19.9 Hz), -158.5 (d, *J* = 19.9 Hz).

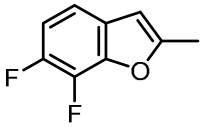

6,7-difluoro-2-methylbenzofuran (1y): The title compound was synthesized on a 5.95 mmol scale according to **GP5**. The product eluted using *n*-pentane and was obtained as colorless liquid (557 mg, 3.31 mmol, 56%), traces of mono-defluorinated by-product could not be removed.

^{1}H NMR (400 MHz, $CDCl_3$) δ 7.10 (ddd, *J* = 8.5, 4.4, 1.5 Hz, 1H), 7.00 (ddd, *J* = 11.0, 8.6, 6.6 Hz, 1H), 6.37–6.34 (m, 1H), 2.47 (s, 2H). **^{13}C{^{1}H} NMR** (101 MHz, $CDCl_3$) δ 157.6 (dd, *J* = 4.0, 1.2 Hz), 147.7 (dd, *J* = 240.8, 9.5 Hz), 142.4 (dd, *J* = 7.8, 4.8 Hz), 136.9 (dd, *J* = 251.6, 16.6 Hz), 128.0 (dd, *J* = 1.8, 1.8 Hz), 114.3 (dd, *J* = 8.1, 4.4 Hz), 112.0 (dd, *J* = 19.9, 1.0 Hz), 102.9 (dd, *J* = 1.9, 1.9 Hz), 14.1. **^{19}F{^{1}H} NMR** (377 MHz, $CDCl_3$) δ -146.1 (d, *J* = 20.4 Hz), -162.1 (d, *J* = 20.6 Hz). **HRMS** (EI) m/z calculated for [$C_9H_6F_2O$] ([M]$^{+\cdot}$) 168.0381, found 168.0372.

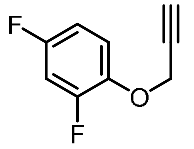

2,4-difluoro-1-(prop-2-yn-1-yloxy)benzene: The title compound was synthesized on a 10.0 mmol scale according to **GP4**. The product eluted using a gradient of *n*-pentane/EtOAc (100:0 to 97:3, *v*:*v*) and was obtained as yellow oil (889 mg, 5.29 mmol, 53%).

^{1}H NMR (400 MHz, $CDCl_3$) δ 7.11–7.03 (m, 1H), 6.87 (ddd, *J* = 11.1, 8.4, 3.0 Hz, 1H), 6.83–6.76 (m, 1H), 4.72 (d, *J* = 2.5 Hz, 3H), 2.54 (t, *J* = 2.4 Hz, 1H). **^{13}C{^{1}H} NMR** (101 MHz, $CDCl_3$) δ 157.5 (dd, *J* = 243.1, 10.4 Hz), 153.2 (dd, *J* = 249.7, 12.1 Hz), 142.0 (dd, *J* = 10.8, 3.6 Hz), 117.7 (dd, *J* = 9.5, 2.7 Hz), 110.6 (dd, *J* = 22.6, 4.0 Hz), 105.2 (dd, *J* = 26.8, 22.2 Hz), 78.1, 76.4, 58.2 (d, *J* = 1.5 Hz). **^{19}F{^{1}H} NMR** (377 MHz, $CDCl_3$) δ -118.3 (d, *J* = 3.7 Hz), -128.5 (d, *J* = 3.5 Hz).

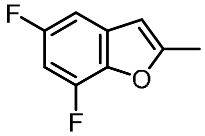

5,7-difluoro-2-methylbenzofuran (1z): The title compound was synthesized on a 4.76 mmol scale according to **GP5**. The product eluted using *n*-pentane and was obtained as colorless liquid (523 mg, 3.11 mmol, 65%), traces of mono-defluorinated by-product could not be removed.

^{1}H NMR (400 MHz, $CDCl_3$) δ 6.92 (ddd, *J* = 8.3, 2.4, 1.0 Hz, 1H), 6.73 (ddd, *J* = 10.6, 9.4, 2.4 Hz, 1H), 6.38–6.35 (m, 1H), 2.46 (d, *J* = 1.4 Hz, 3H). **^{13}C{^{1}H} NMR** (101 MHz, $CDCl_3$) δ 158.5 (dd, *J* = 239.9, 9.3 Hz), 158.4 (d, *J* = 1.0 Hz), 146.8 (dd, *J* = 250.9, 14.1 Hz), 138.3 (dd, *J* = 10.9, 1.9 Hz), 132.4 (dd, *J* = 12.1, 4.5 Hz), 103.5 (dd, *J* = 4.2, 2.5 Hz), 101.6 (dd, *J* = 24.8, 4.2

Hz), 98.9 (dd, *J* = 29.7, 20.3 Hz), 14.0. **^{19}F{^{1}H} NMR** (377 MHz, $CDCl_3$) *δ* -118.4 (d, *J* = 2.8 Hz), -134.2 (d, *J* = 2.8 Hz). **HRMS** (EI) m/z calculated for [$C_9H_6F_2O$] ([M]$^{+\cdot}$) 168.0381, found 168.0371.

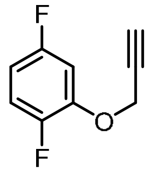

2,5-difluoro-1-(prop-2-yn-1-yloxy)benzene: The title compound was synthesized on a 10.0 mmol scale according to **GP4**. The product eluted using a gradient of *n*-pentane/EtOAc (100:0 to 97:3, *v*:*v*) and was obtained as yellow liquid (1.47 g, 8.77 mmol, 88%).

^{1}H NMR (400 MHz, $CDCl_3$) *δ* 7.03 (ddd, *J* = 10.6, 8.9, 5.3 Hz, 1H), 6.86 (ddd, *J* = 9.6, 6.6, 3.0 Hz, 1H), 6.68–6.61 (m, 1H), 4.75 (d, *J* = 2.4 Hz, 2H), 2.57 (t, *J* = 2.4 Hz, 1H). **^{13}C{^{1}H} NMR** (101 MHz, $CDCl_3$) *δ* 158.6 (dd, *J* = 242.5, 2.6 Hz), 149.3 (dd, *J* = 242.0, 3.4 Hz), 146.1 (dd, *J* = 12.5, 10.5 Hz), 116.6 (dd, *J* = 20.8, 10.1 Hz), 108.0 (dd, *J* = 23.8, 7.0 Hz), 104.0 (dd, *J* = 27.7, 1.8 Hz), 77.5, 76.8, 57.4 (d, *J* = 0.9 Hz). **^{19}F{^{1}H} NMR** (377 MHz, $CDCl_3$) *δ* -116.5 (d, *J* = 15.0 Hz), -139.4 (d, *J* = 15.1 Hz).

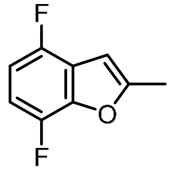

4,7-difluoro-2-methylbenzofuran (1aa): The title compound was synthesized on a 5.95 mmol scale according to **GP5**. The product eluted using *n*-pentane and was obtained as white solid (583 mg, 3.47 mmol, 58%), traces of mono-defluorinated by-product could not be removed.

^{1}H NMR (400 MHz, $CDCl_3$) *δ* 6.88 (ddd, *J* = 10.0, 8.9, 3.9 Hz, 1H), 6.83–6.75 (m, 1H), 6.51–6.49 (m, 1H), 2.50 (d, *J* = 1.2 Hz, 3H). **^{13}C{^{1}H} NMR** (101 MHz, $CDCl_3$) *δ* 156.8, 151.0 (dd, *J* = 244.1, 2.6 Hz), 144.4 (dd, *J* = 243.8, 3.8 Hz), 142.9 (dd, *J* = 13.4, 10.4 Hz), 120.9 (dd, *J* = 24.1, 3.4 Hz), 109.4 (dd, *J* = 19.1, 8.1 Hz), 108.0 (dd, *J* = 22.1, 6.5 Hz), 99.7 (t, *J* = 1.9 Hz), 14.0. **^{19}F{^{1}H} NMR** (377 MHz, $CDCl_3$) *δ* -126.2 (d, *J* = 21.7 Hz), -143.0 (d, *J* = 21.7 Hz). **HRMS** (EI) m/z calculated for [$C_9H_6F_2O$] ([M]$^{+\cdot}$) 168.0381, found 168.0372.

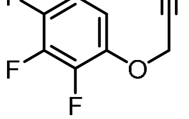

1,2,3-trifluoro-4-(prop-2-yn-1-yloxy)benzene: The title compound was synthesized on a 10.0 mmol scale according to **GP4**. The product eluted using a gradient of *n*-pentane/EtOAc (100:0 to 97:3, *v*:*v*) and was obtained as yellow liquid (720 mg, 3.89 mmol, 39%).

^{1}H NMR (400 MHz, $CDCl_3$) δ 6.95–6.79 (m, 2H), 4.74 (d, *J* = 2.5 Hz, 2H), 2.55 (t, *J* = 2.4 Hz, 1H). **^{13}C{^{1}H} NMR** (101 MHz, $CDCl_3$) δ 146.6 (ddd, *J* = 244.3, 10.3, 1.9 Hz), 143.1 (ddd, *J* = 250.8, 11.4, 3.5 Hz), 143.0 (ddd, *J* = 8.5, 3.4, 1.3 Hz), 140.9 (ddd, *J* = 251.0, 16.4, 13.1 Hz), 110.5 (dd, *J* = 18.5, 4.2 Hz), 110.3–110.2 (m), 77.6, 76.8, 58.2. **^{19}F{^{1}H} NMR** (377 MHz, $CDCl_3$) δ -142.5– -142.7 (m), -152.7– -152.9 (m), -158.0– -158.2 (m).

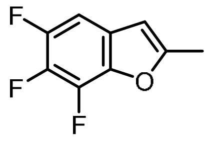

5,6,7-trifluoro-2-methylbenzofuran (1ab): The title compound was synthesized on a 3.76 mmol scale according to **GP5**. The product eluted using *n*-pentane and was obtained as white solid (382 mg, 2.05 mmol, 55%), traces of mono-defluorinated by-product could not be removed.

^{1}H NMR (400 MHz, $CDCl_3$) δ 7.00 (ddd, *J* = 9.4, 6.7, 2.1 Hz, 1H), 6.36–6.34 (m, 1H), 2.46 (s, 3H). **^{13}C{^{1}H} NMR** (101 MHz, $CDCl_3$) δ 158.6 (dd, *J* = 4.1, 1.2 Hz), 149.7–147.0 (m), 139.0–138.4 (m), 138.3–138.1 (m), 136.6–135.9 (m), 125.2 – 125.0 (m), 103.1–102.9 (m), 101.5 (dd, *J* = 20.6, 4.0 Hz), 14.1. **^{19}F{^{1}H} NMR** (377 MHz, $CDCl_3$) δ -141.6 (dd, *J* = 19.8, 1.8 Hz), -157.6 (dd, *J* = 19.5, 1.9 Hz), -166.5 (t, *J* = 19.8 Hz). **HRMS** (EI) m/z calculated for [$C_9H_5F_3O$] ($[M]^{+\cdot}$) 186.0287, found 186.0278.

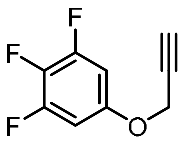

1,2,3-trifluoro-5-(prop-2-yn-1-yloxy)benzene: The title compound was synthesized on a 10.0 mmol scale according to **GP4**. The product eluted using a gradient of *n*-pentane/EtOAc (100:0 to 95:5, *v*:*v*) and was obtained as colorless oil (1.76 g, 9.44 mmol, 94%).

^{1}H NMR (400 MHz, $CDCl_3$) δ 6.67–6.56 (m, 2H), 4.64 (d, *J* = 2.5 Hz, 2H), 2.57 (t, *J* = 2.4 Hz, 1H). **^{13}C{^{1}H} NMR** (101 MHz, $CDCl_3$) δ 153.1–152.8 (m), 151.6 (ddd, *J* = 248.3, 10.8, 5.9 Hz), 135.4 (dt, *J* = 244.5, 15.6 Hz), 100.3–99.8 (m), 77.4, 76.7, 56.7. **^{19}F{^{1}H} NMR** (377 MHz, $CDCl_3$) δ -133.4 (d, *J* = 20.8 Hz), -170.0 (t, *J* = 21.2 Hz).

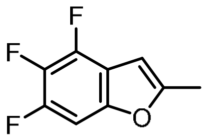

4,5,6-trifluoro-2-methylbenzofuran (1ac): The title compound was synthesized on a 8.00 mmol scale according to **GP5**. The product eluted using *n*-pentane and was obtained as white solid (1.09 g, 5.84 mmol, 73%).

^{1}H NMR (400 MHz, $CDCl_3$) δ 7.06–6.98 (m, 1H), 6.44–6.42 (m, 1H), 2.43 (s, 3H). **^{13}C{^{1}H} NMR** (101 MHz, $CDCl_3$) δ 157.3 (d, *J* = 3.9 Hz), 149.3 (dd, *J* = 25.0, 2.0 Hz), 150.4–147.3 (m), 142.9 (ddd, *J* = 251.4, 12.2, 4.9 Hz), 137.0 (ddd, *J* = 242.3, 17.0, 14.1 Hz), 114.9 (dd, *J* = 18.1, 1.8 Hz), 99.1 (dt, *J* = 3.8, 1.9 Hz), 95.7 (dd, *J* = 22.8, 4.5 Hz), 14.0. **^{19}F{^{1}H} NMR** (377 MHz, $CDCl_3$) δ -140.7 (dd, *J* = 19.9, 2.0 Hz), -143.1 (dd, *J* = 20.3, 2.3 Hz), -169.3 (t, *J* = 20.1 Hz). **HRMS** (EI) m/z calculated for [$C_9H_5F_3O$] ($[M]^{+\cdot}$) 186.0287, found 186.0279.

4. Sensitivity Screen

Recently, our group has established a reaction-condition based assignment to evaluate the sensitivity of a chemical reaction.[11] The sensitivity screen conducted in this study is based on a modification of the literature procedure.

Preparation of stock solution A:

To a 25 mL oven-dried Schlenk flask was added *n*-hexane (8.0 mL, 0.5 M) and 2,5-dimethylbenzofuran **1b** (617 mg, 4.00 mmol).

Standard conditions:

To a 4 ml glass vial (screw-cap) equipped with a stir bar Rh-CAAC **4** (7.7 mg, 14 µmol, 9 mol%) and pulverized 4 Å molecular sieve (50 mg) were added and the vial carefully evacuated. Under argon atmosphere, stock solution **A** (0.30 mL) additional *n*-hexane (0.05 mL) and the preformed Ru((*R*,*R*)-SINpEt)$_2$ **3** catalyst as stock suspension (0.15 mL, 3 mol%) in *n*-hexane were added. The glass vial was placed in a 150 ml stainless steel autoclave under an argon atmosphere. The autoclave was pressurized and depressurized four times with hydrogen gas before the hydrogen pressure was set to 10 bar. The reaction mixture was stirred at 25 °C for 3 h. After this time period, the hydrogen pressure was increased to 70 bar, the temperature increased to 60 °C and the reaction mixture continued to stir for 21 h. After this reaction time the autoclave was carefully depressurized, mesitylene (21 µl) was added and the mixture was analyzed by GC FID analysis.

Table S1: Reaction-condition based assignment for the evaluation of the developed hydrogenation.

Entry	Modification	Deviation from standard condition	Yield	Deviation	ee	Deviation of ee
**1**	High *c*	without additional 50 µl *n*-hexane	99%	+3%	94%	+2%
**2**	Low *c*	+ 100 µl *n*-hexane	95%	0	92%	0
**3**	H_2O	+ 5 µl H_2O	85%	-10%	92%	0
**4**	High O_2	+ 5 ml air, autoclave not purged	99%	+4%	2%	-90%
**5**	Low O_2	degassed solvent	99%	+4%	93%	0
**6**	High *T*	35 °C and 70 °C	95%	0	93%	0
**7**	Low *T*	15 °C and 50 °C	4%	-91%	94%	+2%
**8**	High *p*	15 bar and 90 bar H_2	94%	-2%	93%	0
**9**	Low *p*	5 bar and 50 bar H_2	77%	-18%	92%	0
**10**	Big scale	Standard scale x10	96%	+1%	93%	0

Analysis:

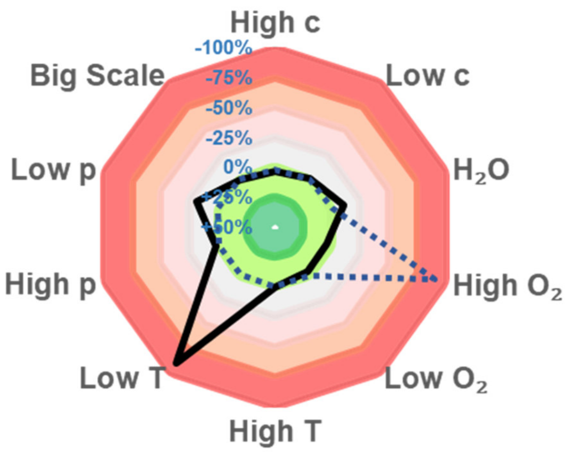

Figure S1: Radar diagram for the reaction-condition based sensitivity assessment. *c* = concentration. *T* = temperature. *p* = pressure. The solid line indicates the yield, the dashed line indicates the enantiomeric excess.

Small deviations of the reaction concentration did not have a significant impact on the reaction outcome. While the addition of H_2O led to a slight decrease of the reaction yield, small deviations of the oxygen level had no impact. Furthermore, the developed reaction is insensitive towards increased temperature and hydrogen pressure. A decreased hydrogen pressure resulted in incomplete conversion and slightly diminished yield, partially hydrogenated intermediate was observed. Negative temperature deviation resulted in a severely reduced yield (due to the unsuccessful formation of the active catalytic rhodium species, see mechanistic investigation below). To our delight, the enantiomeric excess of the obtained product was not sensitive to deviations except to the increased oxygen level, which decomposes the chiral ruthenium catalyst. Scaling up the reaction did not impact yield or enantiomeric excess, which underlines the usefulness of our developed protocol.

5. Hydrogenation Reactions

General one-pot procedure A for the synthesis of octahydrobenzofurans (GP-A):

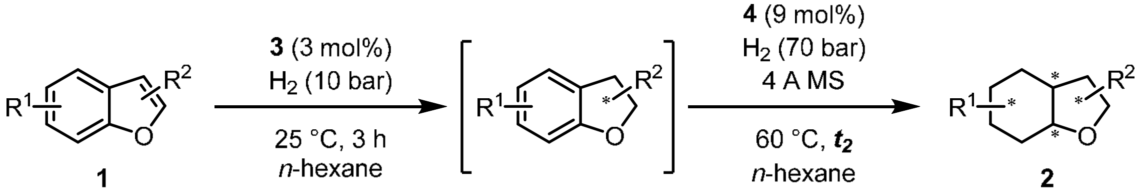

To a 4 mL glass vial (screw-cap with septum) equipped with a stir bar the substrate (300 μmol, 1.00 equiv.), pulverized 4 Å molecular sieve (100 mg), and Rh-CAAC **4** (15.4 mg, 27.0 μmol, 9 mol%) were added and the vial carefully evacuated. Under argon atmosphere, *n*-hexane (0.70 mL) and the preformed Ru((*R*,*R*)-SINpEt)$_2$ catalyst **3** as stock suspension (0.30 mL, 3 mol%) in *n*-hexane were added. The glass vial was paced in a 150 mL stainless steel autoclave under argon atmosphere. The autoclave was pressurized and depressurized with hydrogen gas three times before the pressure was set to 10 bar. The reaction mixture was stirred at 25 °C for 3–5 h. After this time period, the hydrogen pressure was increased to 70 bar and the temperature increased to 60 °C and the reaction mixture continued to stir for 19–21 h (or 43–45 h). After the autoclave was cooled down to rt, it was carefully depressurized and the crude reaction mixture was purified by column chromatography on silica gel eluting with the indicated solvent (differing purification procedures are indicated). Diastereoselectivity was determined by GC-MS analysis of the crude mixture. Racemic mixtures were prepared without the Ru((*R*,*R*)-SINpEt)$_2$ suspension in 1.0 mL solvent on a 0.1 mmol scale at 60 °C.

General procedure B for the step-wise synthesis of fluorinated octahydrobenzofurans (GP-B):

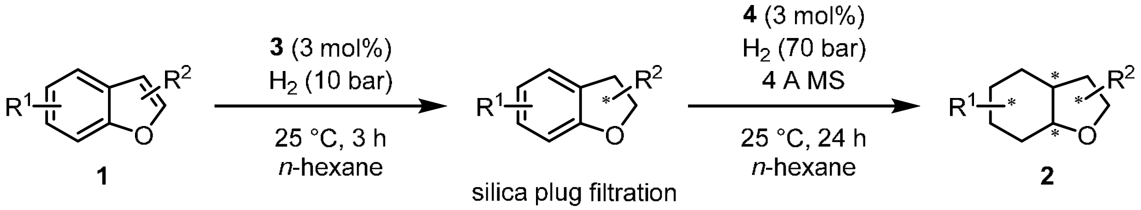

To a 4 mL glass vial (screw-cap with septum) equipped with a stir bar the substrate (300 μmol, 1.00 equiv.) was added and the vial carefully evacuated. Under argon atmosphere, *n*-hexane (0.70 mL) and the preformed Ru((*R*,*R*)-SINpEt)$_2$ catalyst **3** as stock suspension (0.30 mL, 3 mol%) in *n*-hexane were added. The glass vial was paced in a 150 mL stainless steel autoclave under argon atmosphere. The autoclave was pressurized and depressurized with hydrogen gas three times before the pressure was set to 10 bar. The reaction mixture was stirred at 25 °C for 3–5 h. The autoclave was carefully depressurized and the crude reaction

mixture was filtered through a short silica gel plug eluting with a mixture of *n*-pentane/Et2O (9:1 to 1:1, *v*:*v*). The solvent was carefully removed under reduced pressure paying special attention to avoid loss of volatile compounds. The resulting crude mixture was transferred without further purification with a total of 1 mL *n*-hexane into a 4 mL glass vial (screw-cap with septum) equipped with a stir bar, pulverized 4 Å molecular sieve (100 mg), and Rh-CAAC **4** (5.1 mg, 9.0 µmol, 3 mol%) under air. The glass vial was paced in a 150 mL stainless steel autoclave. The autoclave was pressurized and depressurized with hydrogen gas three times before the pressure was set to 70 bar. The reaction mixture was stirred at 25 °C for 24 h. After this time period the autoclave was carefully depressurized and the crude reaction mixture was purified by column chromatography on silica gel eluting with the indicated solvent (differing purification procedures are indicated). Diastereoselectivity was determined by GC-MS analysis of the crude mixture. Racemic mixtures were prepared in a one-pot procedure according to **GP-A** without the Ru((*R*,*R*)-SINpEt)$_2$ suspension in 1.0 mL solvent on a 0.1 mmol scale at 25 °C.

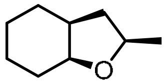

(2*R*,3a*S*,7a*S*)-2-methyloctahydrobenzofuran (2a): The title compound was synthesized according to **GP-A**. The product was isolated by column chromatography using a gradient of *n*-pentane/Et$_2$O (100:0 to 98:2, *v*:*v*) and obtained as a colorless oil (39.7 mg, 0.279 mmol, 93%, 91:9 d.r., 96:4 e.r.).

^{1}H NMR (300 MHz, CDCl$_3$) δ 4.29 (dq, J = 13.3, 6.4 Hz, 0.15H), 4.07–3.94 (m, 1H), 3.78 (dq, J = 4.4, 4.4 Hz, 0.85H), 2.17–1.98 (m, 2H), 1.90–1.78 (m, 1H), 1.69–1.48 (m, 4H), 1.43–1.35 (m, 1.5H), 1.31 (d, J = 6.2 Hz, 3H), 1.29–1.20 (m, 2.5H); signals of both diastereomers are listed. **^{13}C{^{1}H} NMR** (101 MHz, CDCl$_3$) δ 77.6, 74.3, 39.6, 38.3, 29.4, 29.0, 24.2, 23.0, 21.4; signals of the major diastereomer are listed. **Chiral GC-FID** (50_5_100_2_170_20_220, *β*-Dex): t_1 = 23.4 min (minor diastereomer, major enantiomer), t_2 = 23.7 min (major diastereomer, minor enantiomer), t_3 = 24.2 min (major diastereomer, major enantiomer), t_4 = 25.1 min (minor diastereomer, minor enantiomer).

Racemic sample:

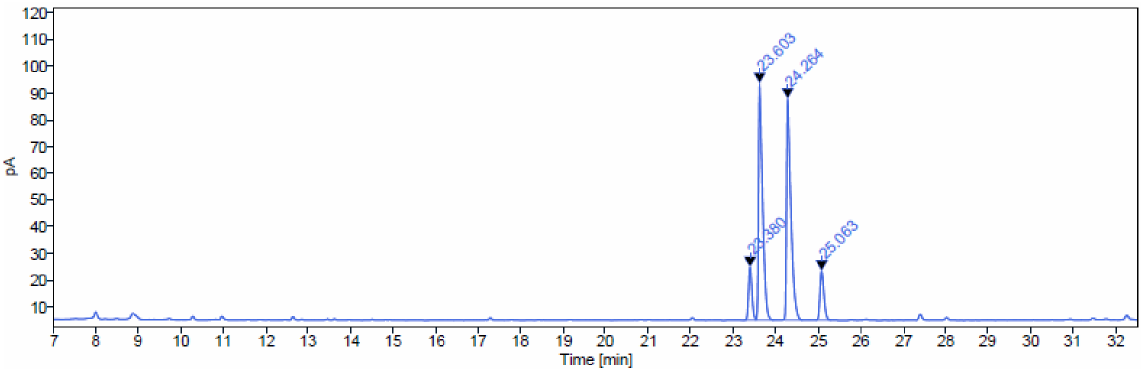

RT [min]	Type	Width [min]	Area	Height	Area%
23.380	MM m	0.09	107.07	19.78	7.37
23.603	MM m	0.10	610.43	88.68	42.04
24.264	MM m	0.10	619.81	82.83	42.69
25.063	MM m	0.10	114.70	18.33	7.90
		Sum	1452.00		

Enantioenriched sample:

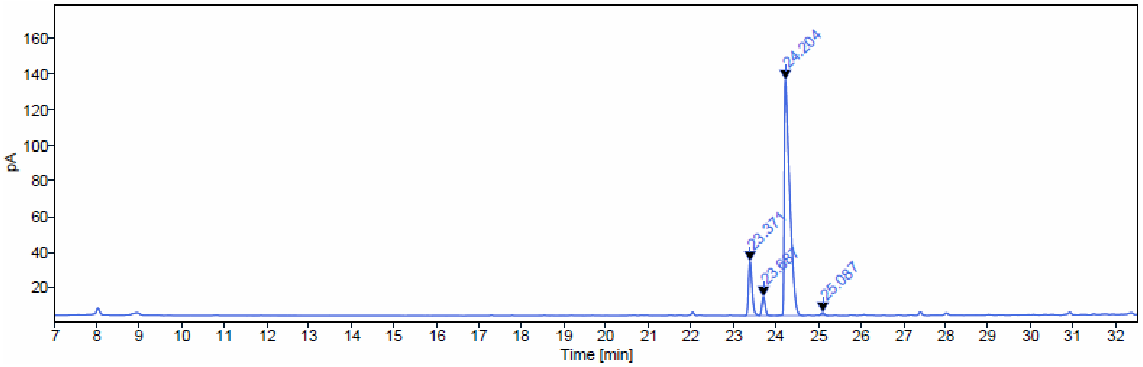

RT [min]	Type	Width [min]	Area	Height	Area%
23.371	MM m	0.09	172.79	30.12	12.34
23.687	MM m	0.08	53.88	10.26	3.85
24.204	MM m	0.12	1166.48	131.91	83.28
25.087	MM m	0.09	7.56	1.36	0.54
		Sum	1400.71		

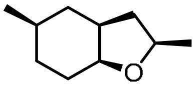

(2*R*,3a*S*,5*R*,7a*S*)-2,5-dimethyloctahydrobenzofuran (2b): The title compound was synthesized according to **GP-A**. The product was isolated by column chromatography using a

gradient of *n*-pentane/Et_2O (100:0 to 98:2, *v*:*v*) and obtained as a colorless oil (45.1 mg, 0.292 mmol, 97%, 93:7 d.r., 96:4 e.r.).

^{1}H NMR (400 MHz, CD_2Cl_2) δ 4.27–4.18 (m, 0.09H), 3.98–3.88 (m, 1H), 3.67 (td, J = 3.8, 2.4 Hz, 0.91H), 2.20 (ddd, J = 12.5, 9.3, 7.8 Hz, 0.91H), 2.03–1.90 (m, 2H), 1.76 (dd, J = 12.2, 6.4 Hz, 0.09H), 1.62–1.52 (m, 1H), 1.51–1.44 (m, 1H), 1.43–1.36 (m, 1H), 1.35–1.28 (m, 1H), 1.26 (d, J = 6.3 Hz, 3H), 1.18–1.05 (m, 2H), 0.92–0.82 (m, 4H); signals of both diastereomers are listed. **^{13}C{^{1}H} NMR** (101 MHz, CD_2Cl_2) δ 77.3, 74.0, 40.7, 39.7, 38.9, 32.0, 29.7, 28.4, 23.0, 22.8; signals of the major diastereomer are listed. **HRMS** (ESI) m/z calculated for [$C_{10}H_{18}ONa$] ([M+Na]$^+$) 177.1250, found 177.1248. **Chiral GC-FID** (50_4_75_0.05_88_20_220, *β*-Dex): t_1 = 49.8 min (minor diastereomer, major enantiomer), t_2 = 56.4 min (major diastereomer, major enantiomer), t_3 = 59.1 min (major diastereomer, minor enantiomer), t_4 = 66.1 min (minor diastereomer, minor enantiomer).

Racemic sample:

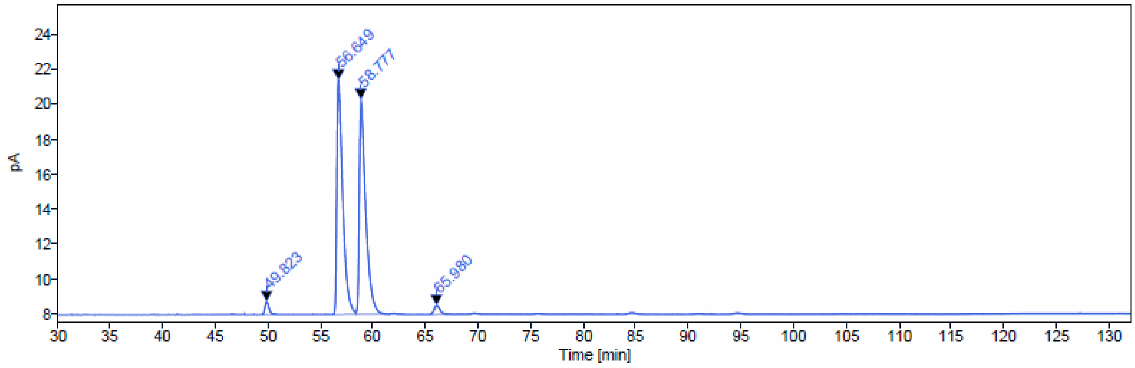

RT [min]	Type	Width [min]	Area	Height	Area%
49.823	MM m	0.33	20.47	0.74	1.92
56.649	MM m	0.64	513.25	13.40	48.08
58.777	MM m	0.70	514.03	12.28	48.15
65.980	MM m	0.64	19.83	0.52	1.86
		Sum	1067.57		

Enantioenriched sample:

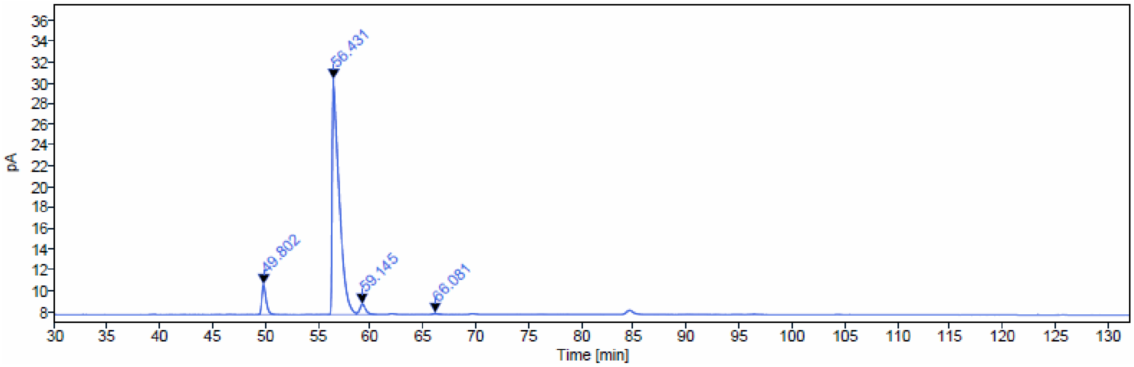

RT [min]	Type	Width [min]	Area	Height	Area%
49.802	MM m	0.33	80.72	2.85	7.07
56.431	MM m	0.53	1019.28	22.59	89.27
59.145	MM m	0.66	38.84	0.97	3.40
66.081	MM m	0.35	2.99	0.10	0.26
		Sum	1141.84		

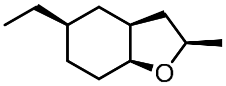

(2*R*,3a*S*,5*R*,7a*S*)-5-ethyl-2-methyloctahydrobenzofuran (2c): The title compound was synthesized according to **GP-A**. The product was isolated by column chromatography using a gradient of *n*-pentane/Et_2O (100:0 to 98:2, *v*:*v*) and obtained as a colorless oil (41.3 mg, 0.245 mmol, 82%, 90:10 d.r., 95:5 e.r.).

^{1}H NMR (400 MHz, $CDCl_3$) δ 4.32–4.24 (m, 0.09H), 4.03–3.93 (m, 1H), 3.73 (dt, J = 3.5, 3.5 Hz, 0.91H), 2.23 (ddd, J = 12.5, 9.2, 7.9 Hz, 1H), 2.11–2.02 (m, 1H), 1.99–1.90 (m, 1H), 1.63–1.44 (m, 3H), 1.31 (d, J = 6.3 Hz, 3H), 1.27–1.05 (m, 5H), 0.90–0.85 (m, 4H); signals of both diastereomers are listed. **^{13}C{^{1}H} NMR** (126 MHz, $CDCl_3$) δ 77.7, 74.0, 40.6, 38.6, 38.5, 37.1, 29.9, 28.1, 27.0, 23.0, 11.5; signals of the major diastereomer are listed. **HRMS** (ESI) m/z calculated for [$C_{11}H_{20}ONa$] ([M+Na]$^+$) 191.1406, found 191.1406. **Chiral GC-FID** (50_5_90_0.05_110_10_180, γ-TA): t_1 = 20.6 min (major diastereomer, major enantiomer), t_2 = 23.7 min (major diastereomer, minor enantiomer), t_3 = 28.2 min (minor diastereomer, major enantiomer), t_4 = 34.3 min (minor diastereomer, minor enantiomer).

Racemic sample: compound is volatile and the used column only gives low intensity

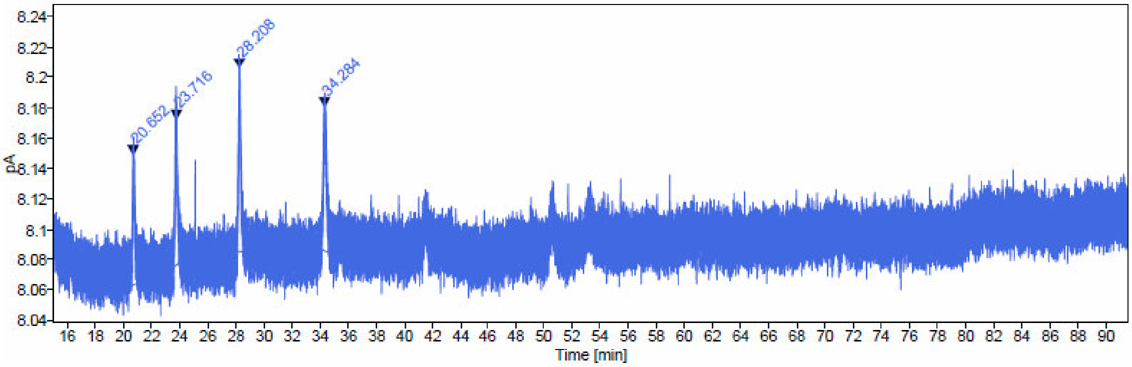

RT [min]	Type	Width [min]	Area	Height	Area%
20.652	MM m	0.16	1.17	0.09	22.08
23.716	MM m	0.21	1.18	0.10	22.30
28.208	MM m	0.15	1.49	0.12	28.07
34.284	MM m	0.18	1.46	0.09	27.54
		Sum	5.31		

Enantioenriched sample:

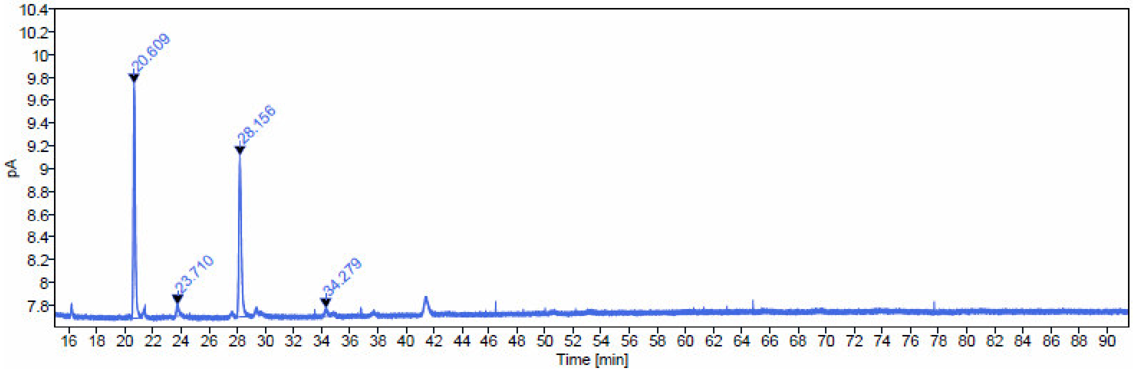

RT [min]	Type	Width [min]	Area	Height	Area%
20.609	MM m	0.15	18.79	2.06	47.53
23.710	MM m	0.13	1.01	0.09	2.57
28.156	MM m	0.17	18.77	1.41	47.49
34.279	MM m	0.18	0.95	0.06	2.41
		Sum	39.52		

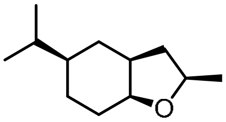

(2*R*,3a*S*,5*R*,7a*S*)-5-*iso*propyl-2-methyloctahydrobenzofuran (2d): The title compound was synthesized according to **GP-A**. The product was isolated by column chromatography using a

gradient of *n*-pentane/EtOAc (100:0 to 99:1, *v*:*v*) and obtained as a colorless oil (40.9 mg, 0.224 mmol, 75%, 95:5 d.r., 97:3 e.r.).

^{1}H NMR (400 MHz, $CDCl_3$) δ 4.31–4.26 (m, 0.05H), 4.03–3.92 (m, 1H), 3.72 (dt, J = 3.6, 3.6 Hz, 0.95H), 2.23 (ddd, J = 12.5, 9.2, 7.8 Hz, 1H), 2.14–2.04 (m, 1H), 1.99–1.89 (m, 1H), 1.62–1.38 (m, 4H), 1.31 (d, J = 6.3 Hz, 3H), 1.27–1.18 (m, 1H), 1.17–1.12 (m, 1H), 1.07–1.00 (m, 1H), 1.00–0.93 (m, 1H), 0.85 (d, J = 6.8 Hz, 6H); signals of both diastereomers are listed. **^{13}C{^{1}H} NMR** (101 MHz, $CDCl_3$) δ 77.6, 73.9, 43.1, 40.6, 38.9, 33.9, 32.8, 28.4, 24.0, 23.0, 19.8; signals of major diastereomer are listed. **HRMS** (ESI) m/z calculated for [$C_{12}H_{22}ONa$] ([M+Na]$^{+}$) 205.1563, found 205.1567. **Chiral GC-FID** (50_5_100_2_170_20_220, β-Dex): t_1 = 31.9 min (major diastereomer, minor enantiomer), t_2 = 32.2 min (major diastereomer, major enantiomer), t_3 = 32.9 min (minor diastereomer, major enantiomer), t_4 = 33.7 min (minor diastereomer, minor enantiomer).

Racemic sample:

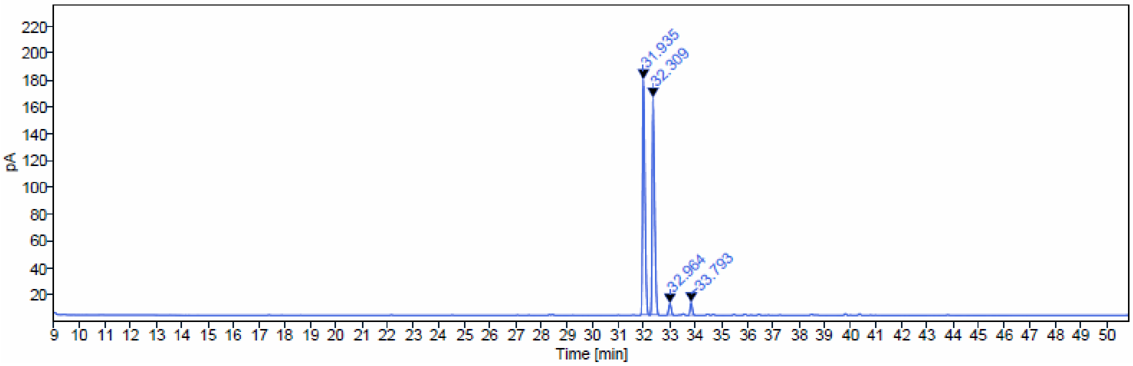

RT [min]	Type	Width [min]	Area	Height	Area%
31.935	MM m	0.11	1120.07	174.65	47.84
32.309	MM m	0.11	1117.26	160.92	47.72
32.964	MM m	0.11	52.00	8.09	2.22
33.793	MM m	0.09	51.73	8.92	2.21
		Sum	2341.05		

Enantioenriched sample:

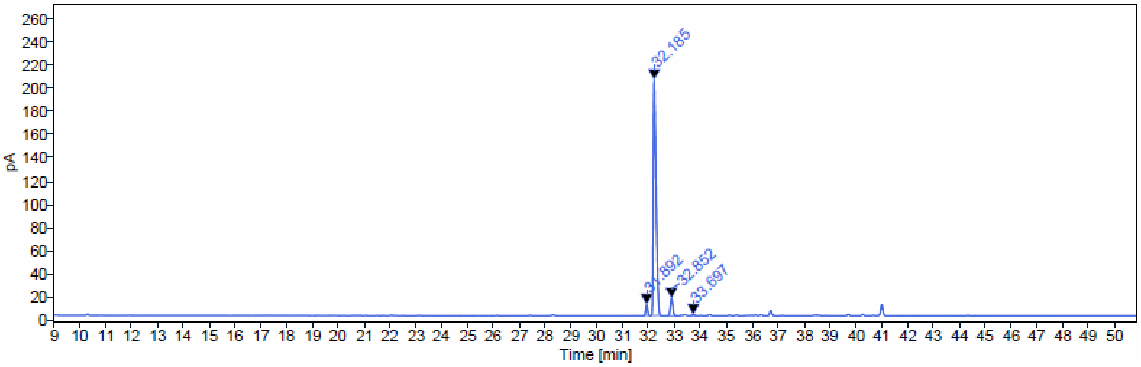

RT [min]	Type	Width [min]	Area	Height	Area%
31.892	MM m	0.08	49.65	9.36	3.07
32.185	MM m	0.11	1465.61	203.02	90.68
32.852	MM m	0.10	97.49	14.75	6.03
33.697	MM m	0.08	3.55	0.69	0.22
		Sum	1616.29		

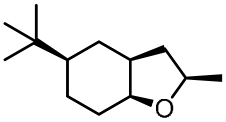

(2*R*,3a*S*,5*R*,7a*S*)-5-*tert*butyl-2-methyloctahydrobenzofuran (2e): The title compound was synthesized according to **GP-A** with a reaction time of 45 h at the elevated temperature of 60 °C. The product was isolated by column chromatography using a gradient of *n*-pentane/EtOAc (99:1 to 95:5, *v*:*v*) and obtained as a colorless oil (50.5 mg, 0.257 mmol, 86%, >95:5 d.r., 97:3 e.r.).

^{1}H NMR (300 MHz, $CDCl_3$) δ 4.04–3.92 (m, 1H), 3.71 (dt, J = 3.6, 3.6 Hz, 1H), 2.24 (ddd, J = 12.5, 9.1, 7.8 Hz, 1H), 2.14–2.04 (m, 1H), 2.00–1.88 (m, 1H), 1.63–1.55 (m, 1H), 1.52–1.47 (m, 1H), 1.31 (d, J = 6.2 Hz, 3H), 1.27–1.11 (m, 3H), 0.98–0.90 (m, 2H), 0.84 (s, 9H). **^{13}C{^{1}H} NMR** (75 MHz, $CDCl_3$) δ 77.4, 73.9, 47.1, 40.7, 39.4, 32.5, 31.7, 28.7, 27.6, 22.9, 21.8. **HRMS** (EI) m/z calculated for [$C_{13}H_{24}O$] ([M]$^{+\cdot}$) 196.1822, found 196.1819. **Chiral GC-FID** (50_5_100_2_170_20_220, *β*-Dex): t_1 = 34.5 min (major diastereomer, minor enantiomer), t_2 = 35.2 min (major diastereomer, major enantiomer).

Racemic sample:

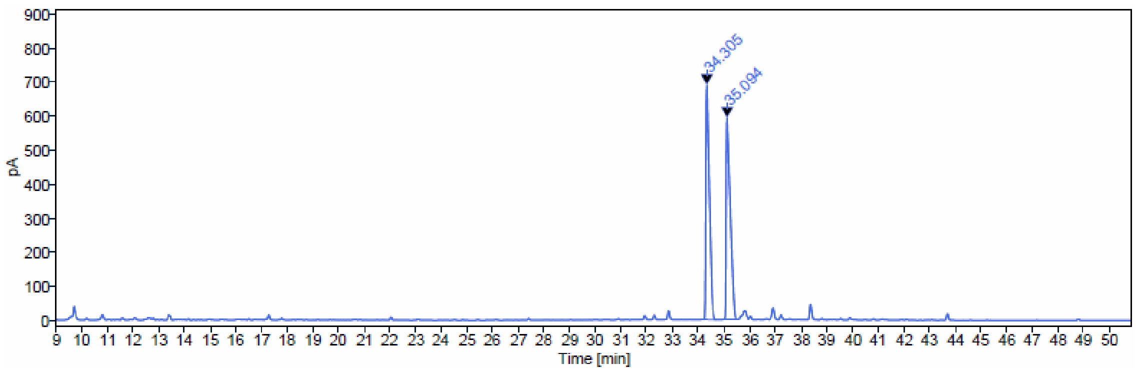

RT [min]	Type	Width [min]	Area	Height	Area%
34.305	MM m	0.12	6295.52	688.46	50.06
35.094	MM m	0.14	6279.78	591.59	49.94
		Sum	12575.29		

Enantioenriched sample:

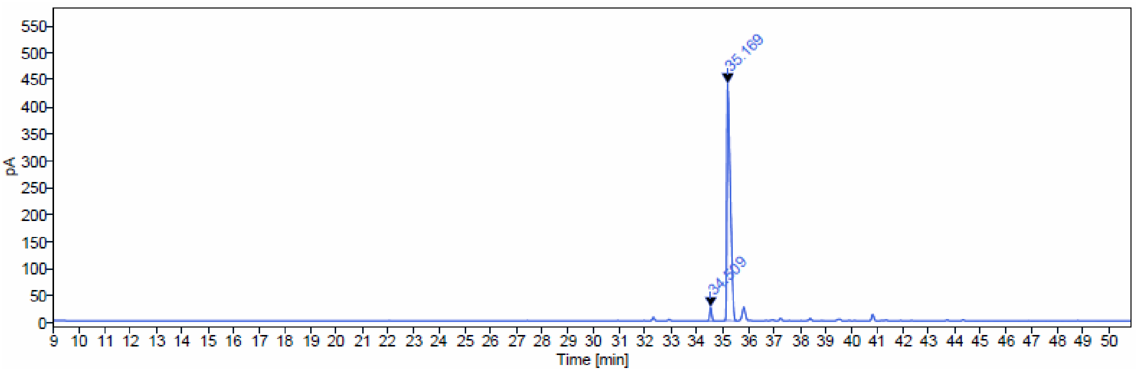

RT [min]	Type	Width [min]	Area	Height	Area%
34.509	MM m	0.09	134.45	24.10	3.26
35.169	MM m	0.12	3986.67	438.45	96.74
		Sum	4121.12		

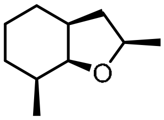

(2*R*,3a*S*,7*S*,7a*S*)- 2,7-dimethyloctahydrobenzofuran (2f): The title compound was synthesized according to **GP-A**. The product was isolated by column chromatography using pure *n*-pentane and obtained as a colorless oil (39.9 mg, 0.259 mmol, 86%, >95:5 d.r., 98:2 e.r.).

^{1}H NMR (400 MHz, $CDCl_3$) δ 4.29–4.22 (m, 0.07H), 4.07–4.02 (m, 0.03H), 4.00–3.91 (m, 0.90H), 3.81–3.79 (m, 0.07H), 3.60–3.56 (m, 0.90H), 3.42–3.38 (m, 0.03H), 2.19 (ddd, J = 12.5, 9.3, 7.7 Hz, 0.90H), 1.96–1.87 (m, 1H), 1.80–1.77 (m, 0.03H), 1.76–1.74 (m, 0.07H), 1.69–1.59 (m, 2H), 1.53–1.45 (m, 1H), 1.43–1.36 (m, 1H), 1.31 (d, J = 6.3 Hz, 3H), 1.28–1.24 (m, 1H), 1.23–1.16 (m, 2H), 1.15–1.09 (m, 1H), 1.05 (d, J = 7.0 Hz, 3H); signals of both diastereomers are listed. **^{13}C{^{1}H} NMR** (101 MHz, $CDCl_3$) δ 82.3, 73.7, 39.9, 39.0, 34.1, 30.1, 28.7, 25.7, 23.2, 19.3; signals of major diastereomer are listed. **HRMS** (EI) m/z calculated for [$C_{10}H_{18}O$] ([M]$^{+\cdot}$) 154.1352, found 154.1352. **Chiral GC-FID** (50_5_100_2_170_20_220, β-Dex): t_1 = 22.0 min (major diastereomer, minor enantiomer), t_2 = 22.4 min (minor diastereomer, major enantiomer), t_3 = 23.0 min (major diastereomer, major enantiomer), t_4 = 25.2 min (minor diastereomer, minor enantiomer).

Racemic sample:

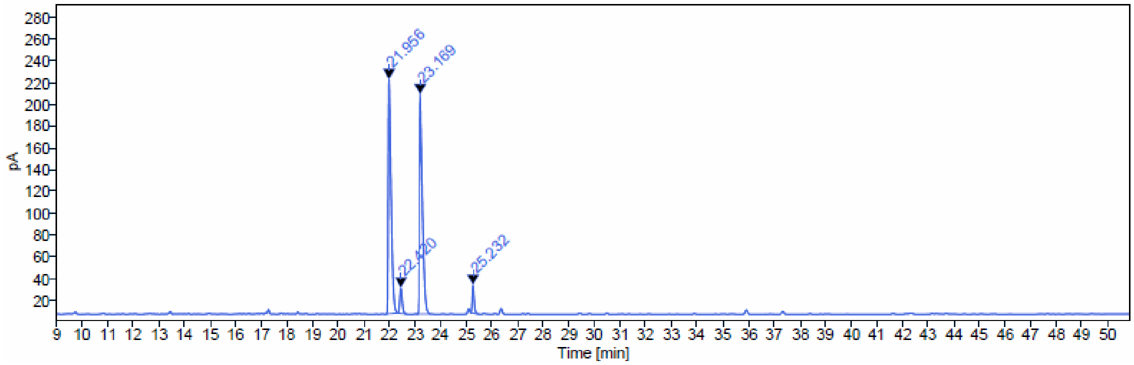

RT [min]	Type	Width [min]	Area	Height	Area%
21.956	MM m	0.12	1589.69	215.07	45.85
22.420	MM m	0.10	145.52	23.34	4.20
23.169	MM m	0.13	1587.80	201.37	45.80
25.232	MM m	0.09	143.81	25.60	4.15
		Sum	3466.82		

Enantioenriched sample:

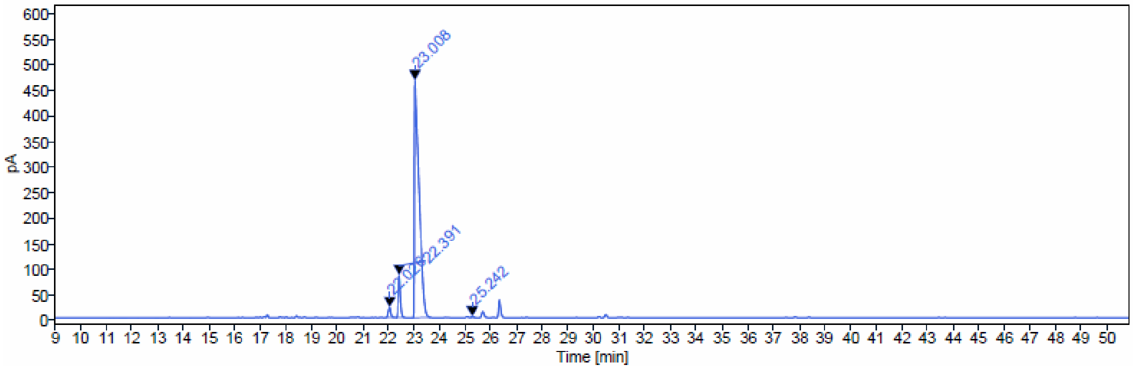

RT [min]	Type	Width [min]	Area	Height	Area%
22.026	MM m	0.11	123.13	19.27	1.95
22.391	MM m	0.08	462.40	82.16	7.31
23.008	MM m	0.15	5729.25	463.30	90.61
25.242	MM m	0.08	7.89	1.58	0.12
		Sum	6322.66		

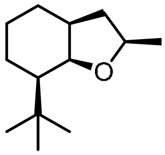

(2*R*,3a*S*,7*R*,7a*R*)-7-*tert*butyl-2-methyloctahydrobenzofuran (2g): The title compound was synthesized according to **GP-A** with a reaction time of 45 h at the elevated temperature of 60 °C. The product was isolated by column chromatography using pure *n*-pentane and obtained as a colorless oil (47.7 mg, 0.243 mmol, 81%, 93:7 d.r., >99:1 e.r.).

^{1}H NMR (400 MHz, $CDCl_3$) δ 3.95–3.85 (m, 1H), 3.80–3.77 (m, 1H), 2.09 (ddd, J = 12.3, 9.3, 7.7 Hz, 1H), 1.90–1.82 (m, 1H), 1.74–1.67 (m, 1H), 1.54–1.45 (m, 2H), 1.39–1.30 (m, 1H), 1.27 (d, J = 6.2 Hz, 3H), 1.23–1.12 (m, 3H), 1.05–0.99 (m, 1H), 0.95 (s, 9H). **^{13}C{^{1}H} NMR** (101 MHz, $CDCl_3$) δ 79.1, 73.9, 49.0, 40.2, 39.3, 33.1, 30.6, 28.7, 26.5, 23.3, 21.8. **HRMS** (EI) m/z calculated for [$C_{13}H_{24}O$] ($[M]^{+\cdot}$) 196.1822, found 196.1821. **Chiral GC-FID** (50_5_100_0.1_120_20_220, β-Dex): t_1 = 43.0 min (major diastereomer, major enantiomer), t_2 = 43.9 min (major diastereomer, minor enantiomer).

Racemic sample:

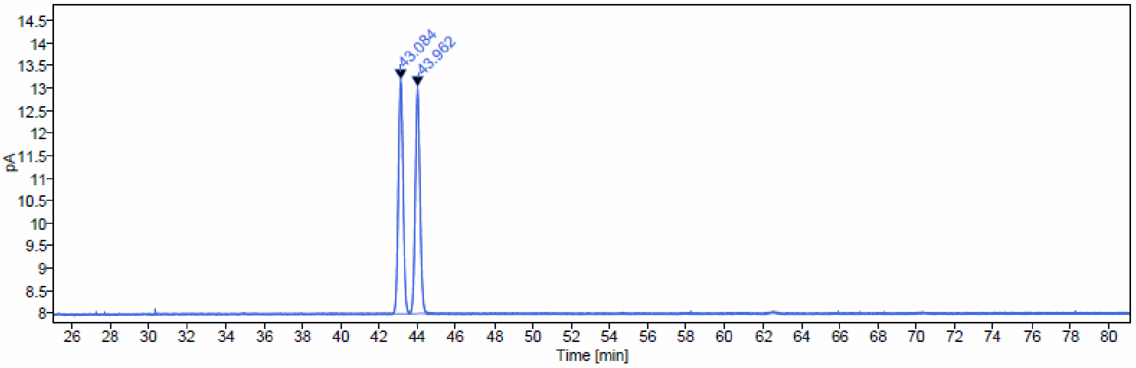

RT [min]	Type	Width [min]	Area	Height	Area%
43.084	MM m	0.28	88.49	5.21	50.03
43.962	MM m	0.22	88.39	5.04	49.97
		Sum	176.88		

Enantioenriched sample:

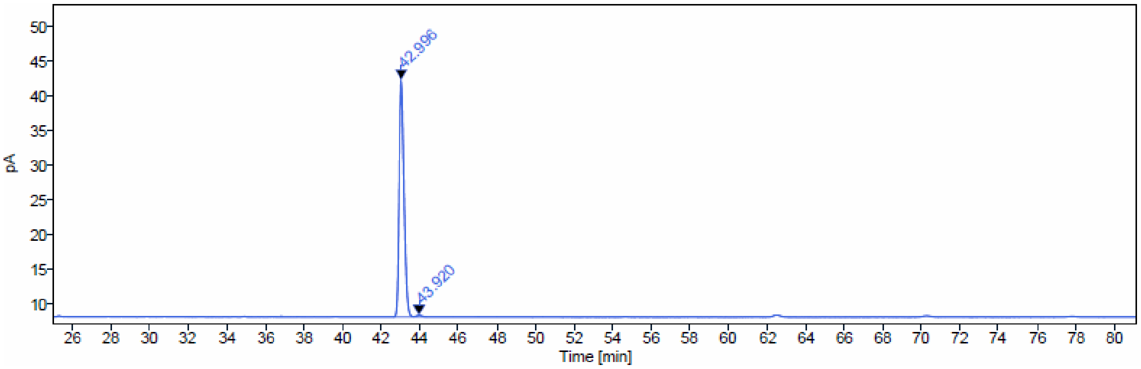

RT [min]	Type	Width [min]	Area	Height	Area%
42.996	MM m	0.22	603.51	34.15	99.31
43.920	MM m	0.21	4.18	0.23	0.69
		Sum	607.69		

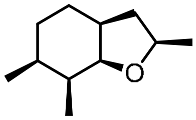

(2*R*,3a*S*,6*S*,7*S*,7a*R*)-2,6,7-trimethyloctahydrobenzofuran (2h): The title compound was synthesized according to **GP-A** with a reaction time of 45 h at 60 °C. The product was isolated by column chromatography using pure *n*-pentane and obtained as a colorless oil (50.1 mg, 0.298 mmol, 99%, >95:5 d.r., 98:2 e.r.).

^{1}H NMR (400 MHz, $CDCl_3$) δ 4.26–4.17 (m, 0.08H), 3.93–3.83 (m, 1H), 3.60 (dd, J = 3.9, 3.9 Hz, 0.92H), 2.23–2.10 (m, 1H), 1.96–1.87 (m, 1H), 1.80–1.66 (m, 2H), 1.55–1.50 (m, 1H), 1.49–1.44 (m, 1H), 1.43–1.34 (m, 2H), 1.29 (d, J = 6.2 Hz, 3H), 1.20–1.19 (m, 0.24H), 1.14 (ddd, J = 12.4, 5.7, 1.7 Hz, 1H), 1.07 (d, J = 7.1 Hz, 2.76H), 1.02 (d, J = 7.1 Hz, 2.76H), 0.96 (d, J = 7.4 Hz, 0.24H); signals of both diastereomers are listed. **^{13}C{^{1}H} NMR** (101 MHz, $CDCl_3$) δ 82.8, 73.9, 39.9, 39.0, 36.0, 32.5, 31.7, 24.4, 23.2, 16.9, 14.9; signals of the major diastereomer are listed. **HRMS** (EI) m/z calculated for [$C_{11}H_{20}O$] ($[M]^{+\cdot}$) 168.1509, found 168.1509. **Chiral GC-FID** (50_5_100_0.1_120_20_220, β-Dex): t_1 = 29.2 min (major diastereomer, minor enantiomer), t_2 = 31.2 min (major diastereomer, major enantiomer).

Racemic sample:

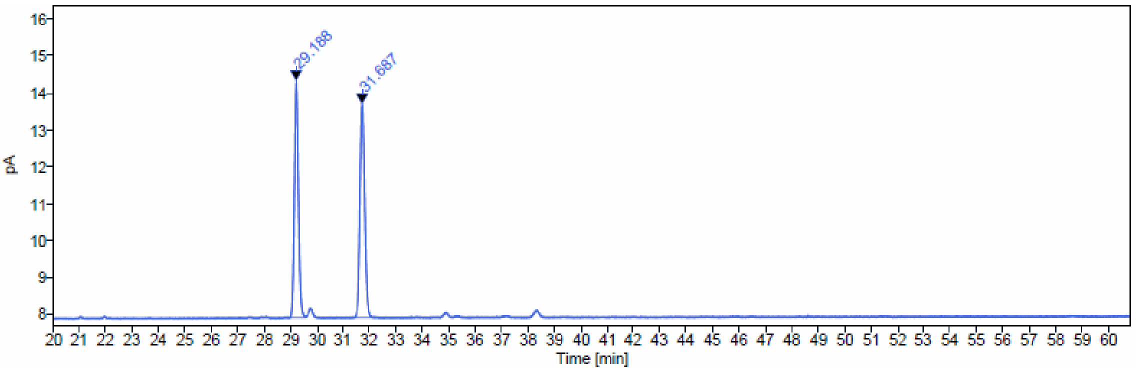

RT [min]	Type	Width [min]	Area	Height	Area%
29.188	MM m	0.18	70.53	6.39	49.86
31.687	MM m	0.21	70.94	5.76	50.14
		Sum	141.47		

Enantioenriched sample:

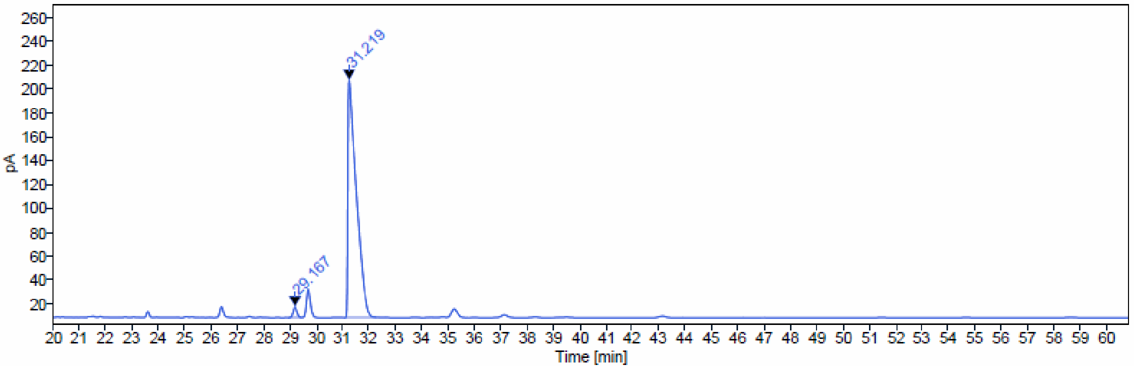

RT [min]	Type	Width [min]	Area	Height	Area%
29.167	MM m	0.17	90.96	8.88	2.01
31.219	MM m	0.28	4436.22	199.45	97.99
		Sum	4527.18		

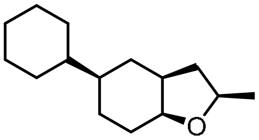

(2*R*,3a*S*,5*R*,7a*S*)-5-cyclohexyl-2-methyloctahydrobenzofuran (2i): The title compound was synthesized according to **GP-A** with a reaction time of 44 h at the elevated temperature of 60 °C. The product was isolated by column chromatography using a gradient of *n*-pentane/EtOAc (99:1 to 97:3, *v*:*v*) and obtained as a colorless oil (49.1 mg, 0.221 mmol, 99%, 83:17 d.r., 92:8 e.r.).

^{1}H NMR (400 MHz, $CDCl_3$) δ 4.32–4.23 (m, 0.12H), 4.02–3.93 (m, 1H), 3.74–3.70 (m, 0.88H), 2.23 (ddd, J = 12.5, 9.2, 7.8 Hz, 0.88H), 2.12–2.03 (m, 1H), 2.03–1.99 (m, 0.12H), 1.98–1.89 (m, 1H), 1.75–1.60 (m, 5H), 1.60–1.54 (m, 1H), 1.54–1.40 (m, 2H), 1.31 (d, J = 6.2 Hz, 3H), 1.25–1.11 (m, 5H), 1.10–0.89 (m, 5H); signals of both diastereomers are listed. **^{13}C{^{1}H} NMR** (101 MHz, $CDCl_3$) δ 77.7, 76.0, 73.9, 43.3, 42.4, 40.6, 39.0, 34.2, 30.2, 30.2, 28.5, 27.0, 27.0, 24.2, 23.0; signals of the major diastereomer are listed. **HRMS** (EI) m/z calculated for [$C_{15}H_{26}O$] ($[M]^{+\cdot}$) 222.1978, found 222.1985; m/z calculated for [$C_{15}H_{25}O$] ($[M-H]^{+\cdot}$) 221.1900, found 221.1901. **Chiral GC-FID** (50_5_90_0.05_110_10_180, γ-TA): t_1 = 356.2 min (major diastereomer, major enantiomer), t_2 = 360.7 min (major diastereomer, minor enantiomer).

Racemic sample:

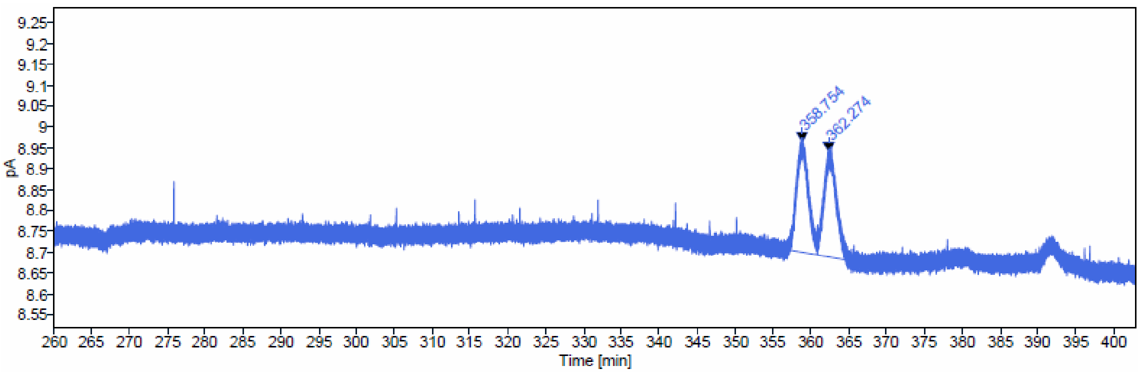

RT [min]	Type	Width [min]	Area	Height	Area%
358.754	MM m	1.28	28.63	0.26	50.15
362.274	MM m	1.33	28.45	0.25	49.85
		Sum	57.08		

Enantioenriched sample: all tested conditions did not yield perfect separation

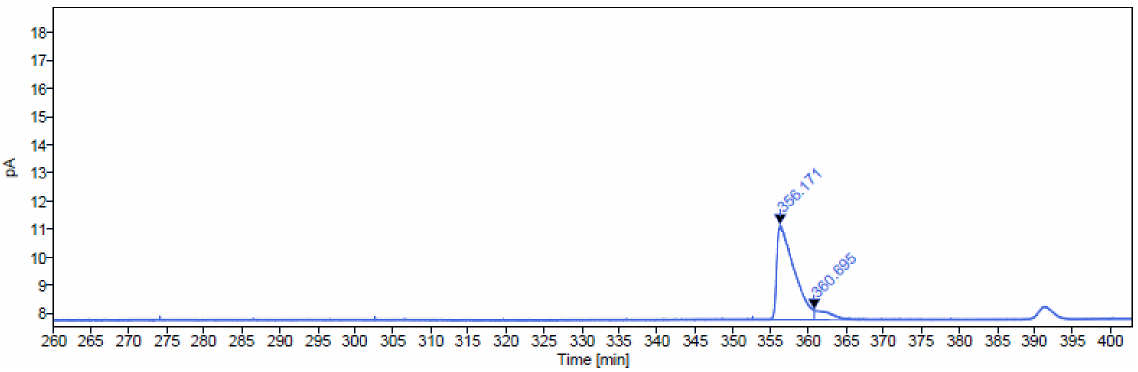

RT [min]	Type	Width [min]	Area	Height	Area%
356.171	MM m	1.86	533.19	3.35	91.80
360.695	MM m	2.36	47.65	0.34	8.20
		Sum	580.84		

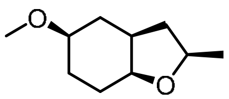

(2*R*,3a*R*,5*R*,7a*S*)-5-methoxy-2-methyloctahydrobenzofuran (2j): The title compound was synthesized according to **GP-A**. The product was isolated by column chromatography using a mixture of *n*-pentane/EtOAc (90:10, *v*:*v*) and obtained as a colorless oil (50.2 mg, 0.295 mmol, 98%, 90:10 d.r., 95:5 e.r.).

^{1}H NMR (400 MHz, $CDCl_3$) δ 4.05–3.94 (m, 1H), 3.82–3.77 (m, 0.06H), 3.69 (dt, J = 3.6, 3.6 Hz, 0.94H), 3.46–3.41 (m, 0.06H), 3.33 (s, 2.82H), 3.30 (s, 0.18H), 3.14–3.05 (m, 0.94H), 2.24 (ddd, J = 12.5, 9.0, 7.7 Hz, 1H), 2.14–2.00 (m, 2H), 1.90–1.83 (m, 1H), 1.82–1.75 (m, 1H), 1.64–1.54 (m, 1H), 1.53–1.42 (m, 1H), 1.31 (d, J = 6.2 Hz, 3H), 1.28–1.22 (m, 2H); signals of both diastereomers are listed. **^{13}C{^{1}H} NMR** (101 MHz, $CDCl_3$) δ 78.5, 76.6, 74.1, 55.7, 40.2, 38.0, 35.5, 26.3, 26.2, 22.9; signals of the major diastereomer are listed. **HRMS** (ESI) m/z calculated for [$C_{10}H_{18}O_2Na$] ([M+Na]$^+$) 193.1199, found 193.1195. **Chiral GC-FID** (50_5_100_2_170_20_220, β-Dex): t_1 = 32.4 min (major diastereomer, minor enantiomer), t_2 = 33.1 min (major diastereomer, major enantiomer).

Racemic sample:

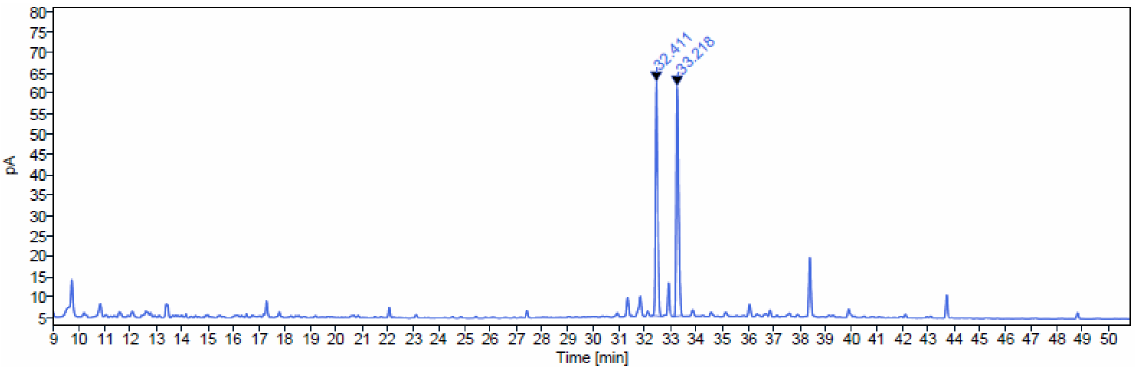

RT [min]	Type	Width [min]	Area	Height	Area%
32.411	MM m	0.09	348.57	57.32	49.61
33.218	MM m	0.10	354.11	56.37	50.39
		Sum	702.69		

Enantioenriched sample:

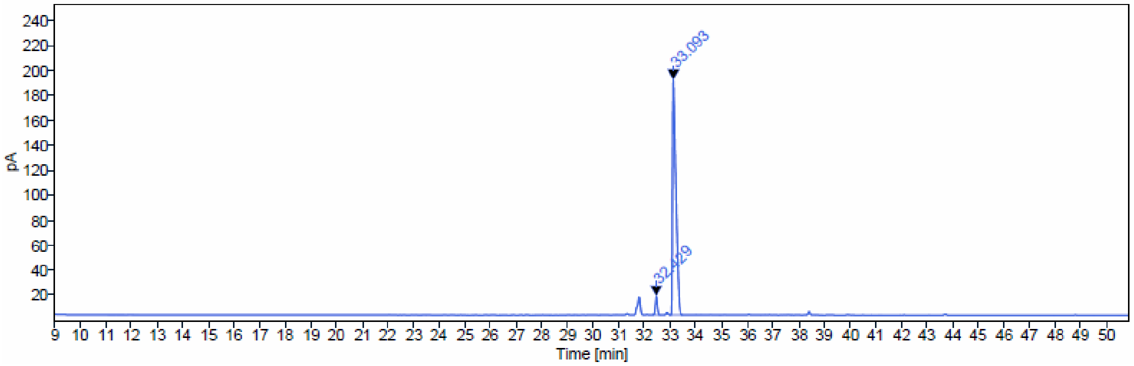

RT [min]	Type	Width [min]	Area	Height	Area%
32.429	MM m	0.09	82.30	14.71	4.74
33.093	MM m	0.12	1654.63	187.82	95.26
		Sum	1736.93		

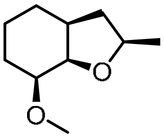

(2*R*,3a*S*,7*S*,7a*R*)-7-methoxy-2-methyloctahydrobenzofuran (2k): The title compound was synthesized according to **GP-A**. The product was isolated by column chromatography using a mixture of *n*-pentane/EtOAc (90:10, *v*:*v*) and obtained as a colorless oil (43.5 mg, 0.256 mmol, 85%, 88:12 d.r., 97:3 e.r.).

^{1}H NMR (400 MHz, $CDCl_3$) δ 4.36–4.30 (m, 0.10H), 4.16–4.13 (m, 0.10H), 4.09–3.99 (m, 0.90H), 3.94–3.90 (m, 0.90H), 3.41 (s, 3H), 3.32 (ddd, *J* = 11.6, 4.7, 3.5 Hz, 1H), 2.18 (ddd, *J* = 12.3, 9.2, 7.5 Hz, 1H), 2.08–1.98 (m, 1H), 1.86–1.78 (m, 1H), 1.75–1.67 (m, 1H), 1.64–1.46 (m, 2H), 1.33 (d, *J* = 6.3 Hz, 3H), 1.27–1.23 (m, 1H), 1.22–1.15 (m, 2H); signals of both diastereomers are listed. **^{13}C{^{1}H} NMR** (101 MHz, $CDCl_3$) δ 79.7, 78.4, 74.5, 56.5, 39.5, 39.3, 29.5, 25.3, 23.5, 22.9; signals of the major diastereomer are listed. **HRMS** (ESI) m/z calculated for [$C_{10}H_{18}O_2Na$] ([M+Na]$^+$) 193.1199, found 193.1197. **Chiral GC-FID** (50_5_90_0.05_110_20_220, *β*-Dex): t_1 = 86.9 min (major diastereomer, minor enantiomer), t_2 = 90.9 min (minor diastereomer, major enantiomer), t_3 = 95.5 min (major diastereomer, major enantiomer), t_4 = 131.7 min (minor diastereomer, minor enantiomer).

Racemic sample:

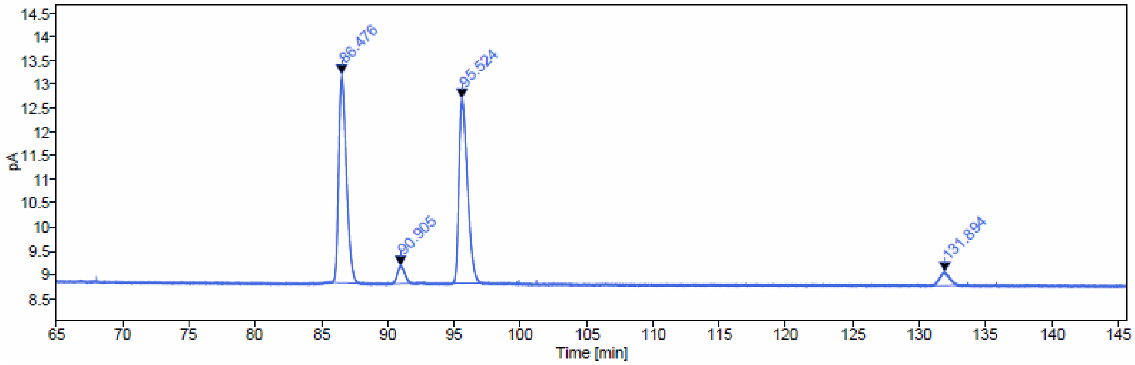

RT [min]	Type	Width [min]	Area	Height	Area%
86.476	MM m	0.47	172.52	4.36	46.19
90.905	MM m	0.47	14.58	0.37	3.90
95.524	MM m	0.74	171.68	3.86	45.96
131.894	MM m	0.63	14.73	0.27	3.94
		Sum	373.51		

Enantioenriched sample:

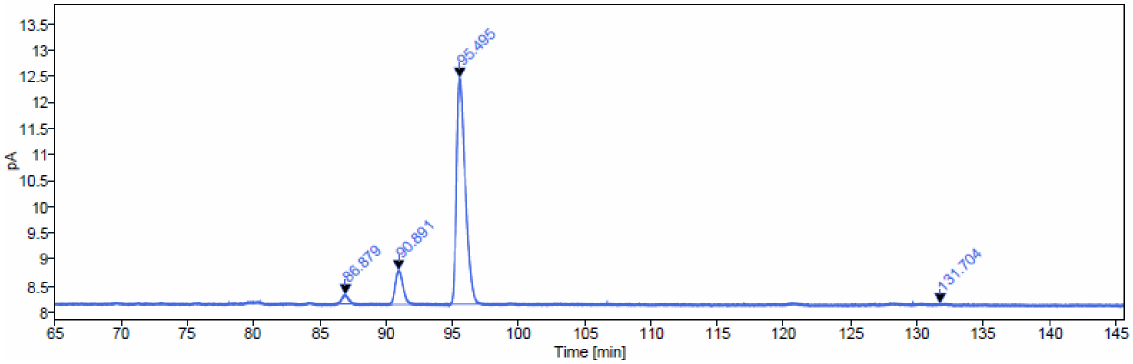

RT [min]	Type	Width [min]	Area	Height	Area%
86.879	MM m	0.37	5.15	0.17	2.25
90.891	MM m	0.48	26.79	0.65	11.72
95.495	MM m	0.53	196.21	4.31	85.86
131.704	MM m	0.21	0.38	0.02	0.17
		Sum	228.54		

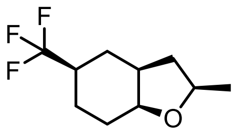

(2*R*,3a*S*,5*R*,7a*S*)-2-methyl-5-(trifluoromethyl)octahydrobenzofuran (2l): The title compound was synthesized according to **GP-A**. The product was isolated by column

chromatography using a gradient of *n*-pentane/EtOAc (99:1 to 95:5, *v*:*v*) and obtained as a colorless oil (53.8 mg, 0.258 mmol, 86%, 95:5 d.r., 89:11 e.r.).

^{1}H NMR (500 MHz, $CDCl_3$) δ 4.35–4.20 (m, 0.08H), 4.06–3.97 (m, 1H), 3.77–3.73 (m, 0.92H), 2.28 (ddd, J = 12.7, 9.1, 7.8 Hz, 1H), 2.20–2.15 (m, 0.92H), 2.06–1.91 (m, 2H), 1.89–1.84 (m, 0.08H), 1.80–1.74 (m, 1H), 1.73–1.67 (m, 1H), 1.66–1.54 (m, 2H), 1.33 (d, J = 6.3 Hz, 3H), 1.26–1.18 (m, 2H); signals of both diastereomers are listed. **^{1}H{^{19}F} NMR** (500 MHz, $CDCl_3$) δ 4.36–4.21 (m, 0.08H), 4.05–3.98 (m, 1H), 3.76–3.73 (m, 0.92H), 2.28 (ddd, J = 12.7, 9.2, 7.8 Hz, 1H), 2.21–2.15 (m, 0.92H), 2.06–1.93 (m, 2H), 1.89–1.84 (m, 0.08H), 1.80–1.74 (m, 1H), 1.73–1.67 (m, 1H), 1.66–1.54 (m, 2H), 1.33 (d, J = 6.3 Hz, 3H), 1.27–1.18 (m, 2H); signals of both diastereomers are listed. **^{13}C{^{1}H} NMR** (126 MHz, $CDCl_3$) δ 127.8 (q, J = 278.4 Hz), 76.3, 74.0, 41.2 (q, J = 26.7 Hz), 40.1, 37.4, 28.5 (q, J = 2.5 Hz), 26.8, 22.8, 19.6 (q, J = 2.6 Hz); signals of the major diastereomer are listed. **^{13}C{^{1}H,^{19}F} NMR** (126 MHz, $CDCl_3$) δ 127.7, 76.3, 74.0, 41.2, 40.1, 37.4, 28.5, 26.8, 22.8, 19.6; signals of the major diastereomer are listed. **^{19}F NMR** (470 MHz, $CDCl_3$) δ -73.8 (d, J = 8.5 Hz, major). **^{19}F{^{1}H} NMR** (470 MHz, $CDCl_3$) δ -73.8, major. **HRMS** (EI) m/z calculated for [$C_{10}H_{15}OF_3$] ($[M]^{+\cdot}$) 208.1070, found 208.1066. **Chiral GC-FID** (50_5_100_1_180_20_220, β-Dex): t_1 = 25.2 min (major diastereomer, minor enantiomer), t_2 = 25.8 min (major diastereomer, major enantiomer).

Racemic sample:

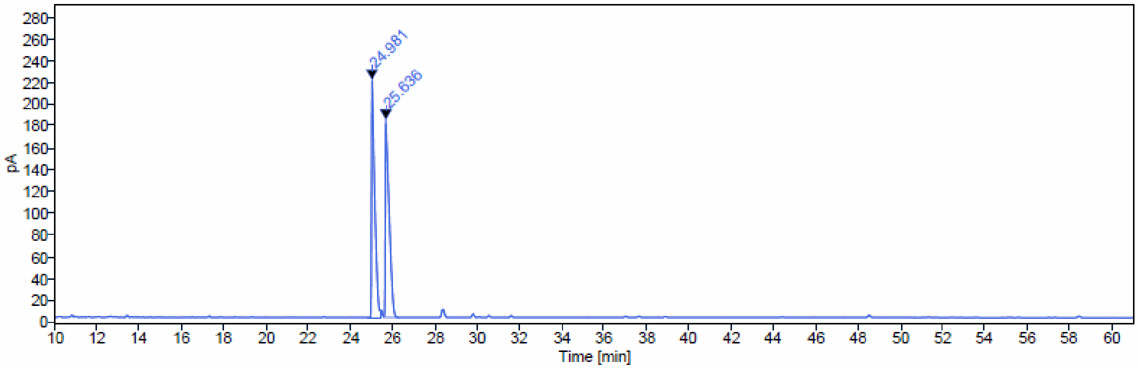

RT [min]	Type	Width [min]	Area	Height	Area%
24.981	MM m	0.14	2384.53	217.82	49.96
25.636	MM m	0.22	2388.74	181.11	50.04
		Sum	4773.27		

Enantioenriched sample:

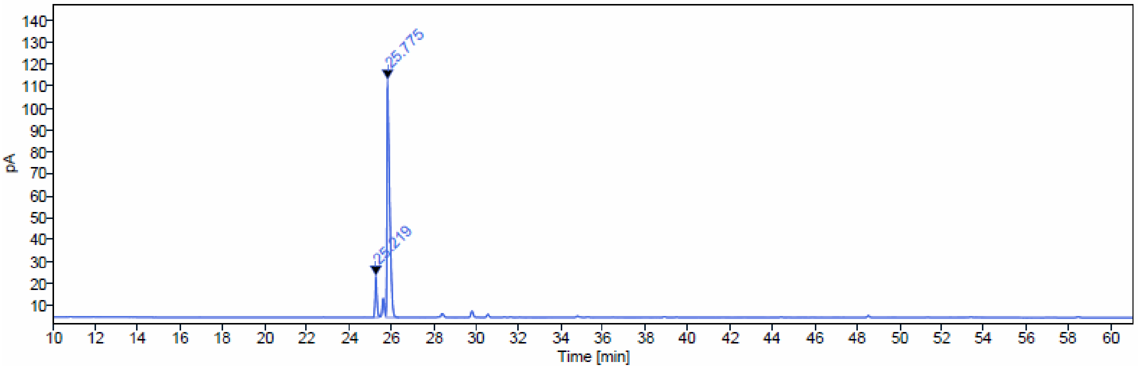

RT [min]	Type	Width [min]	Area	Height	Area%
25.219	MM m	0.10	124.25	18.61	10.93
25.775	MM m	0.13	1013.04	108.17	89.07
		Sum	1137.29		

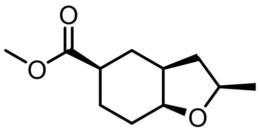

Methyl (2*R*,3a*S*,5*R*,7a*S*)-2-methyloctahydrobenzofuran-5-carboxylate (2m): The title compound was synthesized according to **GP-A** with a reaction time of 45 h at the elevated temperature of 60 °C. The product was isolated by column chromatography using a gradient of *n*-pentane/EtOAc (95:5 to 85:15, *v*:*v*) and obtained as a colorless oil (51.7 mg, 0.261 mmol, 87%, 94:6 d.r., 86:14 e.r.).

^{1}H NMR (400 MHz, $CDCl_3$) δ 4.34–4.27 (m, 0.06H), 4.04–3.94 (m, 0.94H), 3.86–3.83 (m, 0.06H), 3.75–3.71 (m, 0.94H), 3.67 (s, 0.18H), 3.66 (s, 2.82H), 2.42–2.33 (m, 0.12H), 2.29–2.19 (m, 1.88H), 2.17–2.06 (m, 1H), 2.05–1.96 (m, 1H), 1.84–1.71 (m, 2H), 1.71–1.56 (m, 2H), 1.45–1.34 (m, 1H), 1.31 (d, J = 6.3 Hz, 3H), 1.24–1.16 (m, 1H); signals of both diastereomers are listed. **^{13}C{^{1}H} NMR** (101 MHz, $CDCl_3$) δ 176.3, 76.5, 74.0, 51.7, 42.4, 40.1, 37.8, 32.4, 27.4, 23.5, 22.8; signals of the major diastereomer are listed. **HRMS** (ESI) m/z calculated for [$C_{11}H_{18}O_3Na$] ([M+Na]$^+$) 221.1148, found 221.1146. **Chiral GC-FID** (50_5_100_0.1_120_20_220, β-Dex): t_1 = 120.3 min (major diastereomer, minor enantiomer), t_2 = 123.6 min (major diastereomer, major enantiomer), t_3 = 126.1 min (minor diastereomer, major enantiomer), t_4 = 143.7 min (minor diastereomer, minor enantiomer).

Racemic sample:

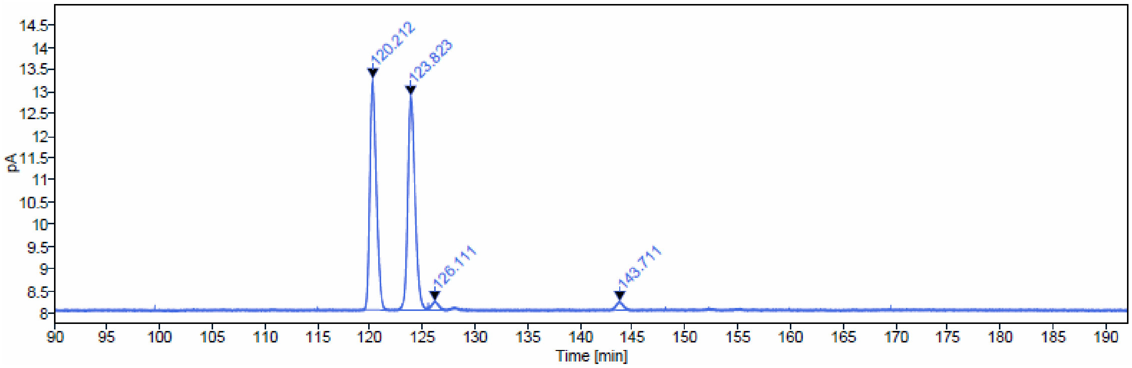

RT [min]	Type	Width [min]	Area	Height	Area%
120.212	MM m	0.51	225.61	5.21	47.79
123.823	MM m	0.79	228.64	4.82	48.43
126.111	MM m	0.79	8.99	0.19	1.90
143.711	MM m	0.78	8.84	0.19	1.87
		Sum	472.09		

Enantioenriched sample:

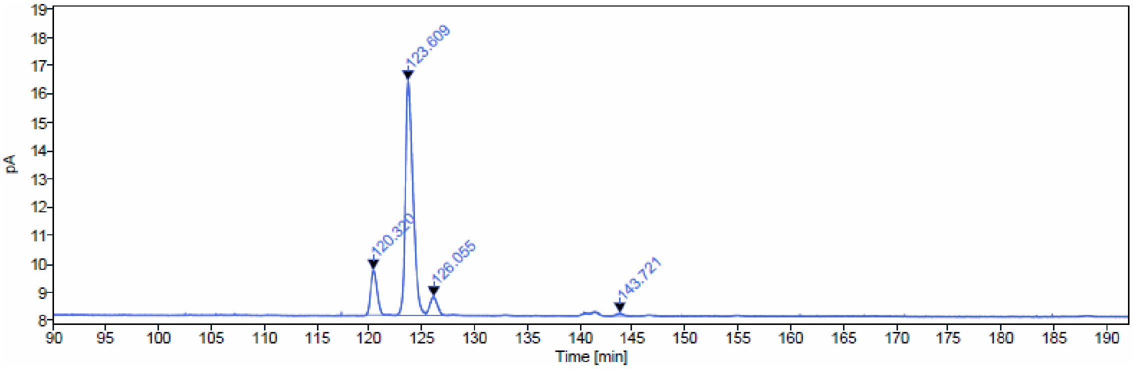

RT [min]	Type	Width [min]	Area	Height	Area%
120.320	MM m	0.49	66.28	1.57	12.80
123.609	MM m	0.59	414.84	8.27	80.12
126.055	MM m	0.58	32.43	0.65	6.26
143.721	MM m	0.51	4.24	0.10	0.82
		Sum	517.79		

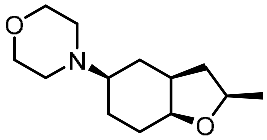

4-((2*R*,3a*S*,5*R*,7a*S*)-2-methyloctahydrobenzofuran-5-yl)morpholine (2n): The title compound was synthesized according to **GP-A**. The product was isolated by column chromatography using a gradient of DCM/MeOH (100:0 to 95:5, *v*:*v*) and obtained as a off-white solid (62.3 mg, 0.276 mmol, 92%, 92:8 d.r., 95:5 e.r.).

^{1}H NMR (400 MHz, $CDCl_3$) δ 4.33–4.26 (m, 0.07H), 4.03–3.92 (m, 0.93H), 3.76–3.67 (m, 5H), 2.60–2.47 (m, 4H), 2.31–2.20 (m, 2H), 2.19–2.12 (m, 1H), 2.08–1.98 (m, 1H), 1.78–1.70 (m, 1H), 1.69–1.64 (m, 1H), 1.63–1.56 (m, 1H), 1.56–1.45 (m, 1H), 1.31 (d, J = 6.3 Hz, 3H), 1.25–1.15 (m, 2H); signals of both diastereomers are listed. **^{13}C{^{1}H} NMR** (101 MHz, $CDCl_3$) δ 76.9, 74.0, 67.4, 63.0, 49.6, 40.4, 38.7, 32.5, 27.5, 22.9, 22.7; signals of major diastereomer are listed. **HRMS** (ESI) m/z calculated for $[C_{13}H_{23}NO_2H]$ ($[M+H]^+$) 226.1802, found 226.1794. **Chiral GC-FID** (50_5_90_0.05_110_20_220, β-Dex): t_1 = 307.2 min (major diastereomer, major enantiomer), t_2 = 312.0 min (major diastereomer, minor enantiomer).

Racemic sample:

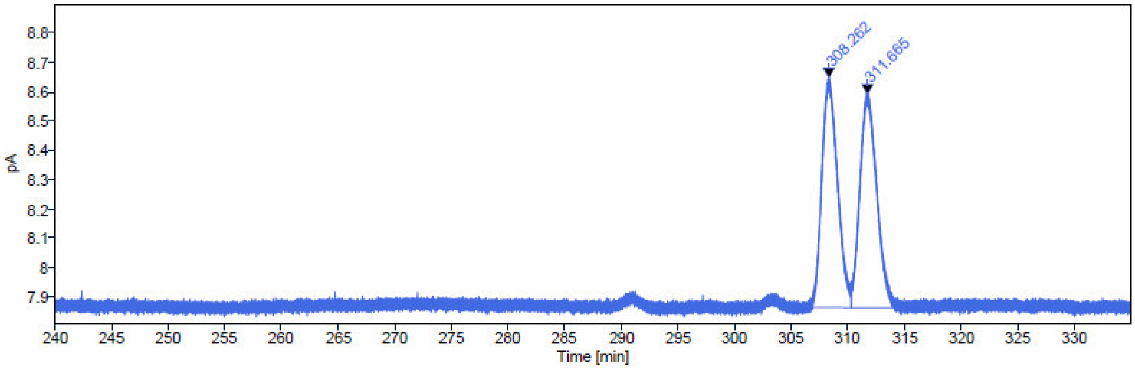

RT [min]	Type	Width [min]	Area	Height	Area%
308.262	MM m	1.12	74.65	0.78	49.79
311.665	MM m	1.22	75.28	0.72	50.21
		Sum	149.93		

Enantioenriched sample:

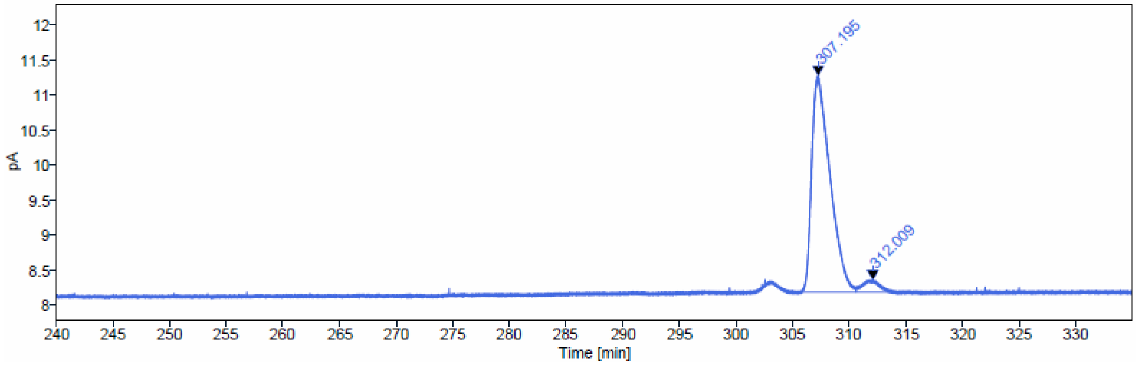

RT [min]	Type	Width [min]	Area	Height	Area%
307.195	MM m	1.32	345.85	3.07	95.36
312.009	MM m	1.71	16.83	0.16	4.64
		Sum	362.68		

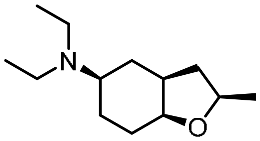

(2*R*,3a*S*,5*R*,7a*S*)-*N*,*N*-diethyl-2-methyloctahydrobenzofuran-5-amine (2o): The title compound was synthesized according to **GP-A**. The product was isolated by column chromatography using a mixture of DCM/MeOH (1:1, *v*:*v*) and obtained as a pale yellow oil (49.5 mg, 0.234 mmol, 78%, 95:5 d.r., 95:5 e.r.).

^{1}H NMR (400 MHz, $CDCl_3$) δ 4.10–3.94 (m, 2H), 2.53 (qd, J = 7.1, 1.9 Hz, 4H), 2.49–2.43 (m, 1H), 2.42–2.31 (m, 1H), 2.05–1.98 (m, 1H), 1.88–1.76 (m, 2H), 1.67–1.56 (m, 2H), 1.55–1.44 (m, 1H), 1.41–1.33 (m, 1H), 1.29 (d, J = 6.1 Hz, 3H), 1.22–1.16 (m, 1H), 1.02 (t, J = 7.2 Hz, 6H); signals of both diastereomers are listed. **^{13}C{^{1}H} NMR** (101 MHz, $CDCl_3$) δ 78.1, 75.5, 56.5, 43.7, 38.4, 37.2, 33.8, 24.8, 24.4, 22.8, 13.8; signals of major diastereomer are listed. **HRMS** (ESI) m/z calculated for [$C_{13}H_{25}NOH$] ([M+H]$^+$) 212.2009, found 212.2006. **Chiral GC-FID** (50_5_100_0.1_120_20_220, *β*-Dex): t_1 = 126.0 min (major diastereomer, minor enantiomer), t_2 = 127.3 min (major diastereomer, major enantiomer), t_3 = 136.8 min (minor diastereomer, major enantiomer), t_4 = 145.2 min (minor diastereomer, minor enantiomer).

Racemic sample:

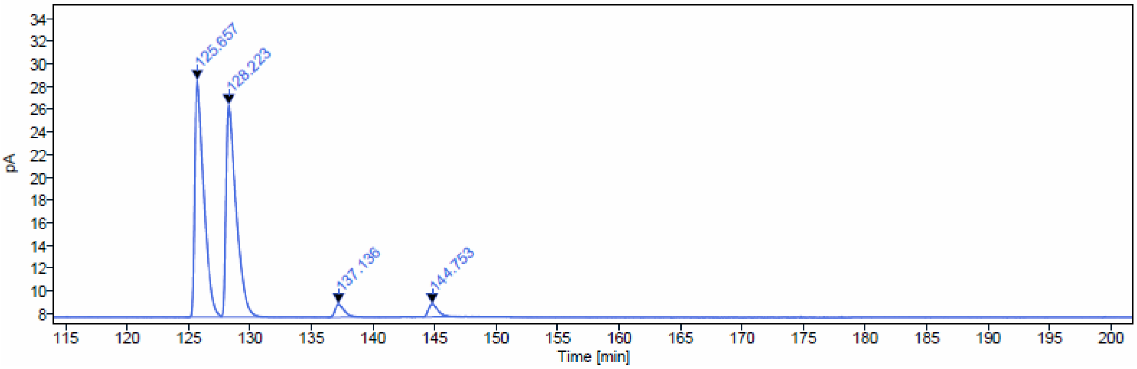

RT [min]	Type	Width [min]	Area	Height	Area%
125.657	MM m	0.86	1070.51	20.87	47.39
128.223	MM m	0.67	1068.62	18.70	47.31
137.136	MM m	0.61	60.21	1.16	2.67
144.753	MM m	0.87	59.53	1.14	2.64
		Sum	2258.87		

Enantioenriched sample:

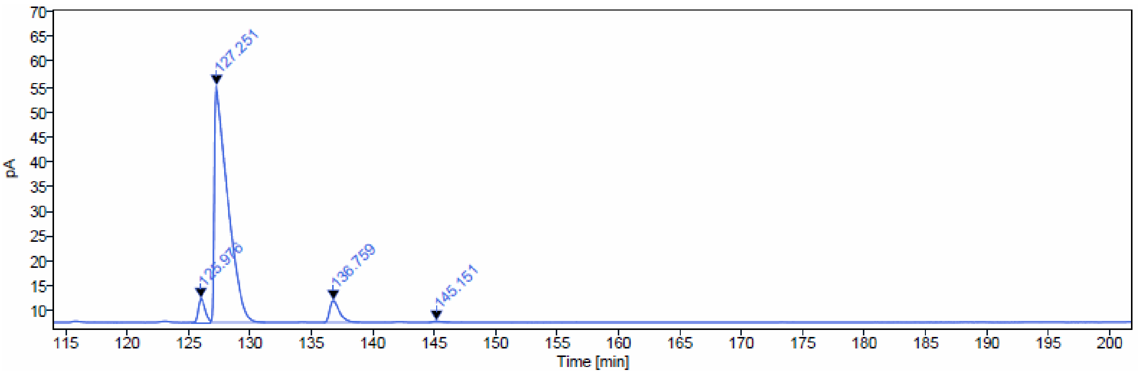

RT [min]	Type	Width [min]	Area	Height	Area%
125.976	MM m	0.44	180.28	4.79	4.46
127.251	MM m	0.89	3617.82	47.64	89.45
136.759	MM m	0.63	236.48	4.40	5.85
145.151	MM m	0.79	10.03	0.21	0.25
		Sum	4044.61		

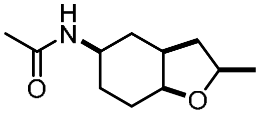

***N*-(2-methyloctahydrobenzofuran-5-yl)acetamide (2p):** The title compound was synthesized according to **GP-A**. The product was isolated by column chromatography using a gradient of DCM/MeOH (98:2 to 95:5, *v*:*v*) and obtained as a pale yellow oil (59.0 mg, 0.299 mmol, 99%, 91:9 d.r., 51:49 e.r.).

^{1}H NMR (400 MHz, $CDCl_3$) δ 5.57–5.51 (m, 1H), 4.31–4.21 (m, 0.09H), 4.02–3.93 (m, 1H), 3.73–3.61 (m, 1.91H), 2.24 (ddd, J = 12.6, 9.2, 7.8 Hz, 1H), 2.13–2.04 (m, 2H), 1.93 (s, 3H), 1.84–1.75 (m, 1H), 1.73–1.63 (m, 2H), 1.45–1.32 (m, 1H), 1.28 (d, J = 6.3 Hz, 3H), 1.21–1.02 (m, 2H); signals of both diastereomers are listed. **^{13}C{^{1}H} NMR** (101 MHz, $CDCl_3$) δ 169.3, 76.2, 73.9, 47.6, 40.0, 37.9, 36.9, 27.3, 26.8, 23.6, 22.9; signals of the major diastereomer are listed. **HRMS** (ESI) m/z calculated for $[C_{11}H_{19}NO_2Na]$ ($[M+Na]^+$) 220.1308, found 220.1304. **Chiral HPLC-UV** (AD-H, hexane:*i*PrOH = 95:5, 1.0 mL/min, 360 nm): t_1 = 15.8 min, t_2 = 25.8 min.

Racemic sample:

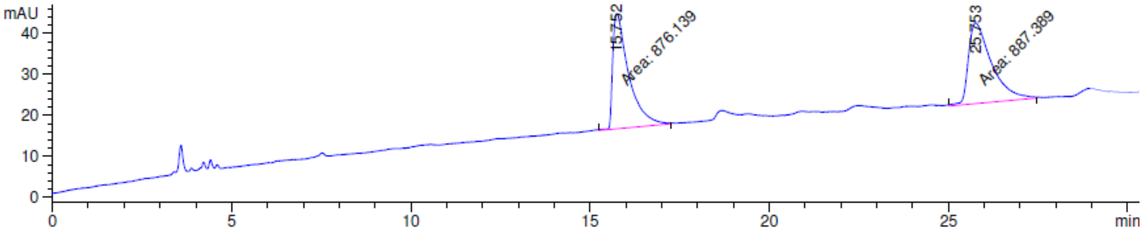

Peak #	RetTime [min]	Type	Width [min]	Area [mAU*s]	Height [mAU]	Area %
1	15.752	MM	0.5198	876.13904	28.09312	49.6810
2	25.753	MM	0.7497	887.38934	19.72780	50.3190
Totals :				1763.52838	47.82092	

Enantioenriched sample:

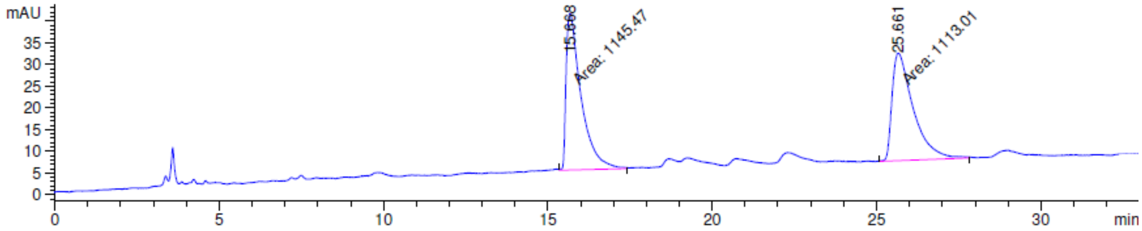

Peak #	RetTime [min]	Type	Width [min]	Area [mAU*s]	Height [mAU]	Area %
1	15.668	MM	0.5286	1145.47058	36.11370	50.7186
2	25.661	MM	0.7497	1113.01343	24.74246	49.2814
Totals :				2258.48401	60.85616	

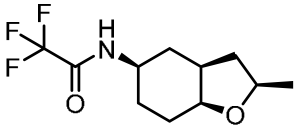

2,2,2-trifluoro-*N*-((2*R*,3a*S*,5*R*,7a*S*)-2-methyloctahydrobenzofuran-5-yl)acetamide (2q): The title compound was synthesized according to **GP-A** starting from 5-amino-2-methylbenzofuran **1q**. After the autoclave was depressurized the diastereomeric ratio was determined by GC-MS analysis. To the crude reaction mixture triethylamine (125 µL, 3.00 equiv.) and trifluoroacetic anhydride (125 µL, 3.00 equiv.) were added and the resulting mixture stirred for 1 h at rt. The resulting reaction mixture was separated by column chromatography on silica gel using a gradient of DCM/MeOH (98:2 to 96:4, *v*:*v*). The desired

compound was obtained as a pale yellow oil (67.9 mg, 0.270 mmol, 90%, 84:16 d.r., 96:4 e.r.) as a mixture of diastereo- and rotamers.

^{1}H NMR (600 MHz, $CDCl_3$) δ 6.36 (bs, 1H), 4.31–4.26 (m, 0.16H), 4.03–3.97 (m, 0.84H), 3.96–3.93 (m, 0.16H), 3.88–3.85 (m, 0.16H), 3.78–3.72 (m, 0.84H), 3.71–3.66 (m, 0.84H), 2.27 (ddd, *J* = 12.8, 9.3, 7.8 Hz, 1H), 2.19–2.09 (m, 2H), 1.86–1.81 (m, 1H), 1.77–1.67 (m, 2H), 1.57–1.49 (m, 1H), 1.35–1.33 (m, 0.48H), 1.32–1.28 (m, 2.52H), 1.23–1.19 (m, 1H), 1.18–1.14 (m, 1H); signals of diastereo- and rotamers are listed. **^{13}C{^{1}H} NMR** (151 MHz, $CDCl_3$) δ 156.5 (q, *J* = 36.7 Hz), 116.0 (q, *J* = 288.0 Hz), 75.8, 74.0, 48.7, 40.0, 37.8, 35.9, 26.7, 26.6, 22.9; signals of the major diastereo- and rotamer are listed. **^{13}C{^{1}H,^{19}F} NMR** (151 MHz, $CDCl_3$) δ 156.4, 115.9, 75.8, 74.0, 48.7, 40.0, 37.7, 35.9, 26.7, 26.6, 22.9; signals of the major diastereo- and rotamer are listed. **^{19}F{^{1}H} NMR** (564 MHz, $CDCl_3$) δ -76.0. **HRMS** (ESI) m/z calculated for $[C_{11}H_{16}NO_2F_3Na]$ ($[M+Na]^+$) 274.1025, found 274.1028. **Chiral GC-FID** (50_5_90_0.05_110_20_220, β-Dex): t_1 = 392.3 min (major diastereomer, major enantiomer), t_2 = 406.9 min (major diastereomer, minor enantiomer).

Racemic sample:

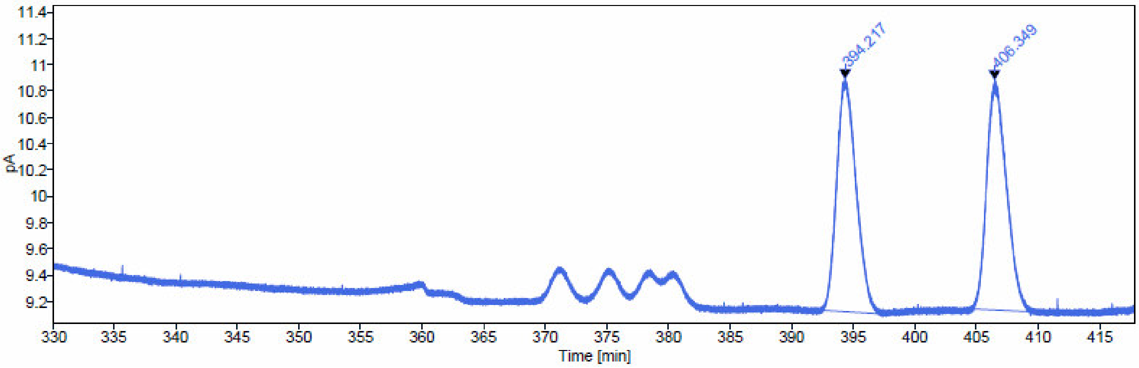

RT [min]	Type	Width [min]	Area	Height	Area%
394.217	MM m	1.83	193.44	1.76	50.03
406.349	MM m	1.30	193.23	1.74	49.97
		Sum	386.67		

Enantioenriched sample:

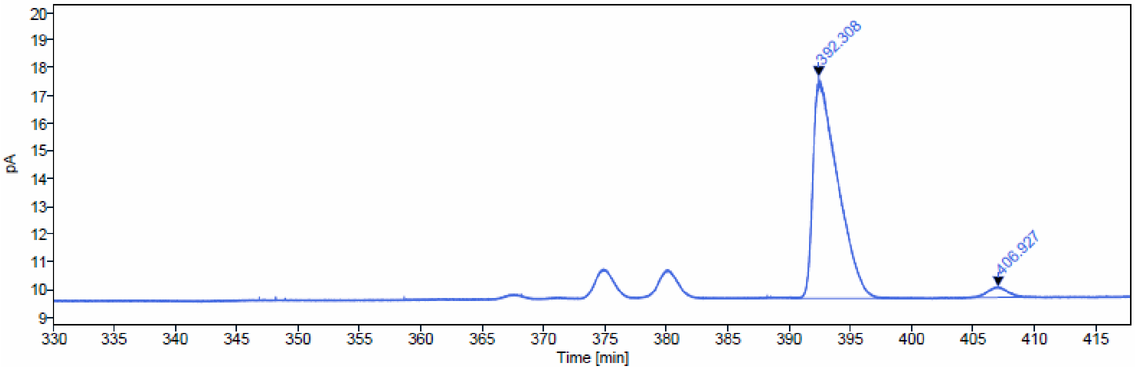

RT [min]	Type	Width [min]	Area	Height	Area%
392.308	MM m	1.62	1106.08	7.99	96.33
406.927	MM m	1.31	42.16	0.38	3.67
		Sum	1148.24		

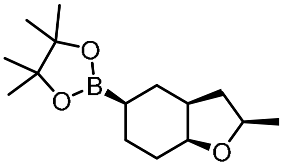

4,4,5,5-tetramethyl-2-((2*R*,3a*S*,5*R*,7a*S*)-2-methyloctahydrobenzofuran-5-yl)-1,3,2-dioxaborolane (2r): The title compound was synthesized according to **GP-A** with a reaction time of 45 h at the elevated temperature of 60 °C. The product was isolated by column chromatography using a mixture of *n*-pentane/EtOAc (90:10, *v*:*v*) and obtained as a colorless oil (41.7 mg, 0.157 mmol, 52%, 81:19 d.r., 96:4 e.r.).

^{1}H NMR (400 MHz, $CDCl_3$) δ 4.33–4.24 (m, 0.09H), 4.06–4.00 (m, 0.09H), 4.00–3.91 (m, 0.91H), 3.83–3.78 (m, 0.09H), 3.76–3.72 (m, 0.82H), 2.28–2.21 (m, 0.09H), 2.17 (ddd, J = 12.5, 9.0, 7.8 Hz, 0.91H), 2.06–1.97 (m, 1H), 1.97–1.87 (m, 0.91H), 1.8– 1.76 (m, 0.09H), 1.67–1.50 (m, 3H), 1.50–1.43 (m, 1H), 1.31 (d, J = 6.3 Hz, 3H), 1.22 (s, 12H), 1.18–1.12 (m, 2H), 0.88–0.78 (m, 1H); signals of all diastereomers are listed. **^{13}C{^{1}H} NMR** (101 MHz, $CDCl_3$) δ 83.0, 77.5, 75.8, 74.7, 73.9, 40.2, 38.8, 31.4, 29.0, 24.9, 24.9, 22.8, 22.2; signals of the major diastereomer are listed, one carbon missing. **^{11}B NMR** (128 MHz, $CDCl_3$) δ 33.82. **HRMS** (ESI) m/z calculated for [$C_{15}H_{27}BO_3Na$] ([M+Na]$^+$) 289.1948, found 289.1944. **Chiral GC-FID** (50_5_90_0.05_110_10_180, γ-TA): t_1 = 369.3 min (major diastereomer, major enantiomer), t_2 = 385.2 min (major diastereomer, minor enantiomer).

Racemic sample:

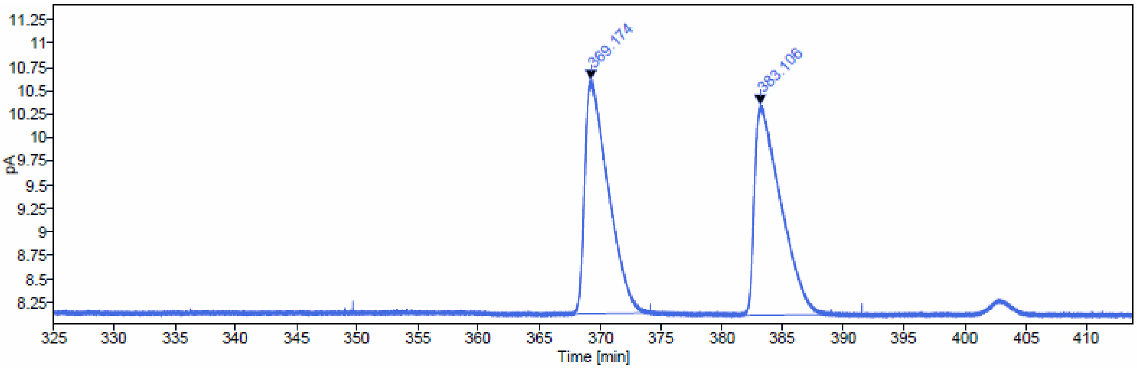

RT [min]	Type	Width [min]	Area	Height	Area%
369.174	MM m	1.57	332.83	2.48	49.82
383.106	MM m	1.76	335.29	2.23	50.18
		Sum	668.12		

Enantioenriched sample:

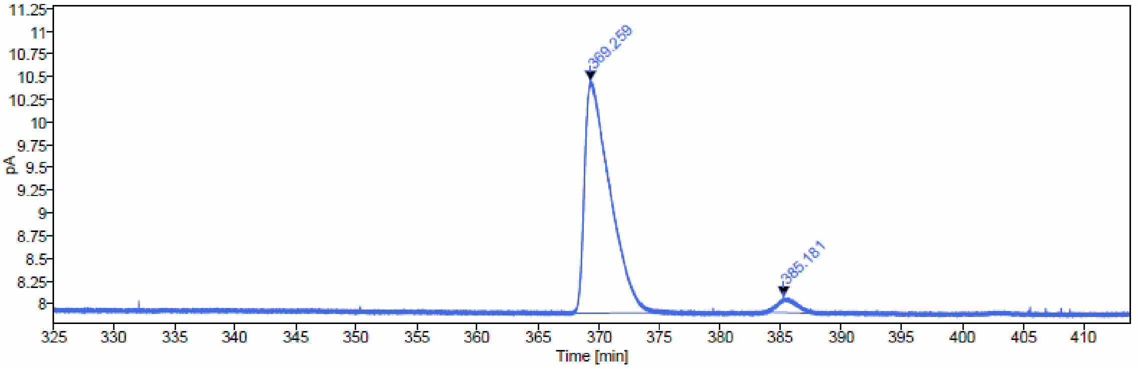

RT [min]	Type	Width [min]	Area	Height	Area%
369.259	MM m	1.70	371.66	2.56	95.79
385.181	MM m	1.11	16.32	0.17	4.21
		Sum	387.98		

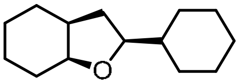

(2*S*,3a*S*,7a*S*)-2-cyclohexyloctahydrobenzofuran (2s): The title compound was synthesized according to **GP-B** from a commercial starting material. The product was isolated by column chromatography using a mixture of *n*-pentane/EtOAc (98:2, *v*:*v*) and obtained as a colorless oil (58.2 mg, 0.279 mmol, 93%, >95:5 d.r., 98:2 e.r.).

^{1}H NMR (400 MHz, $CDCl_3$) δ 3.88–3.84 (m, 0.06H), 3.83–3.80 (m, 0.06H), 3.76 (ddd, J = 5.1, 5.1, 5.1 Hz, 0.94H), 3.55 (ddd, J = 7.7, 7.7, 7.7 Hz, 0.94H), 2.12–2.03 (m, 1H), 1.99–1.86 (m,

2H), 1.79–1.69 (m, 3H), 1.69–1.60 (m, 3H), 1.59–1.45 (m, 3H), 1.44–1.34 (m, 3H), 1.34–1.12 (m, 5H), 1.03–0.89 (m, 2H); signals of both diastereomers are listed. **^{13}C{^{1}H} NMR** (101 MHz, $CDCl_3$) δ 83.3, 77.0, 44.1, 37.6, 35.0, 30.2, 29.3, 29.3, 28.8, 26.8, 26.3, 26.2, 23.8, 21.8; signals of the major diastereomer are listed. **HRMS** (EI) m/z calculated for [$C_{14}H_{24}O$] ([M]$^{+\cdot}$) 208.1827, found 208.1827. **Chiral GC-FID** (50_5_100_0.1_120_20_220, β-Dex): t_1 = 225.1 min (major diastereomer, minor enantiomer), t_2 = 226.4 min (major diastereomer, major enantiomer), t_3 = 236.0 min (minor diastereomer, major enantiomer), t_4 = 243.1 min (minor diastereomer, minor enantiomer).

Racemic sample:

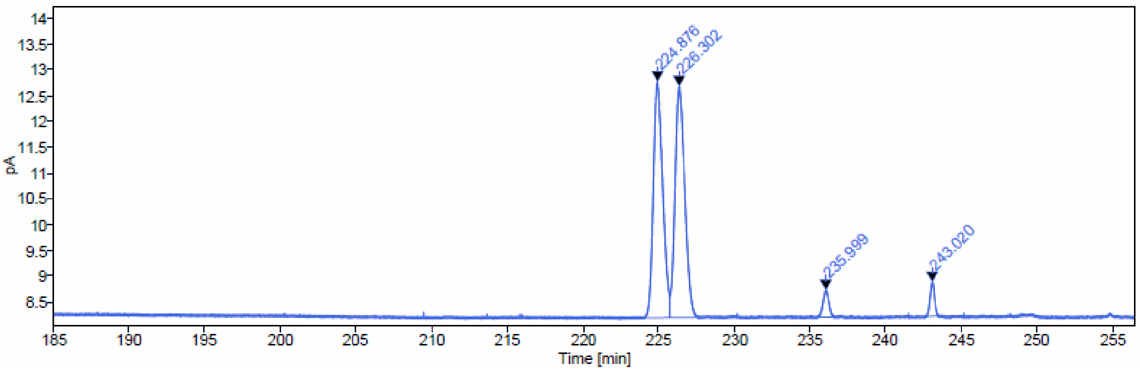

RT [min]	Type	Width [min]	Area	Height	Area%
224.876	MM m	0.51	197.37	4.56	46.56
226.302	MM m	0.52	199.60	4.47	47.09
235.999	MM m	0.45	13.88	0.52	3.28
243.020	MM m	0.24	13.01	0.66	3.07
		Sum	423.87		

Enantioenriched sample:

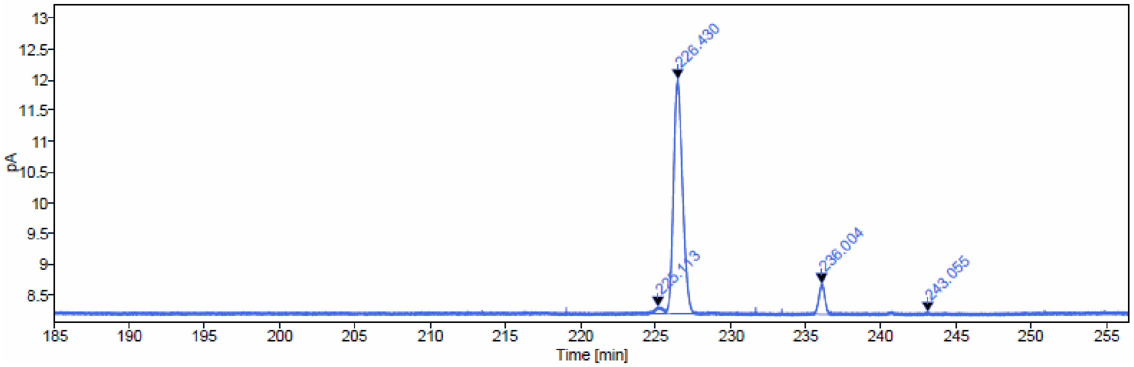

RT [min]	Type	Width [min]	Area	Height	Area%
225.113	MM m	0.61	3.70	0.10	2.12
226.430	MM m	0.49	157.75	3.80	90.09
236.004	MM m	0.45	13.29	0.49	7.59
243.055	MM m	0.23	0.37	0.03	0.21
		Sum	175.12		

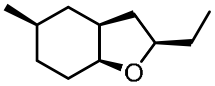

(2*R*,3a*S*,5*R*,7a*S*)-2-ethyl-5-methyloctahydrobenzofuran (2t): The title compound was synthesized according to **GP-A**. The product was isolated by column chromatography using a gradient of *n*-pentane/EtOAc (100:0 to 98:2, *v*:*v*) and obtained as a colorless oil (46.8 mg, 0.278 mmol, 93%, 94:6 d.r., 92:8 e.r.).

^{1}H NMR (400 MHz, $CDCl_3$) δ 4.08–4.00 (m, 0.09H), 3.92–3.89 (m, 0.09H), 3.78–3.68 (m, 1.82H), 2.15 (ddd, J = 12.5, 9.2, 7.9 Hz, 1H), 2.10–2.03 (m, 1H), 2.00–1.91 (m, 1H), 1.79–1.67 (m, 1H), 1.65–1.56 (m, 1H), 1.55–1.49 (m, 1H), 1.49–1.36 (m, 2H), 1.34–1.23 (m, 1H), 1.21–1.08 (m, 2H), 0.93 (t, J = 7.4 Hz, 3H), 0.90–0.85 (m, 1H), 0.86 (d, J = 6.4 Hz, 3H); signals of both diastereomers are listed. **^{13}C{^{1}H} NMR** (101 MHz, $CDCl_3$) δ 79.7, 76.8, 39.4, 38.3, 38.0, 31.7, 30.1, 29.4, 28.1, 22.8, 10.8; signals of the major diastereomer are listed. **HRMS** (EI) m/z calculated for [$C_{11}H_{19}O$] ([M-H]$^{+\cdot}$) 167.1430, found 167.1430. **Chiral GC-FID** (50_5_90_0.05_110_20_220, β-Dex): t_1 = 43.7 min (major diastereomer, minor enantiomer), t_2 = 44.5 min (minor diastereomer, major enantiomer), t_3 = 45.3 min (major diastereomer, major enantiomer), t_4 = 55.8 min (minor diastereomer, minor enantiomer).

Racemic sample:

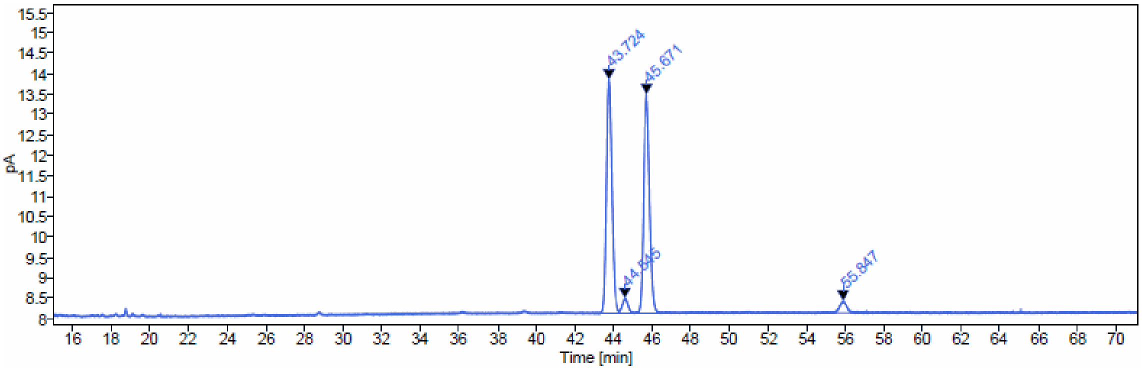

RT [min]	Type	Width [min]	Area	Height	Area%
43.724	MM m	0.23	111.42	5.72	46.82
44.545	MM m	0.34	7.26	0.36	3.05
45.671	MM m	0.35	111.88	5.35	47.02
55.847	MM m	0.43	7.39	0.28	3.10
		Sum	237.96		

Enantioenriched sample:

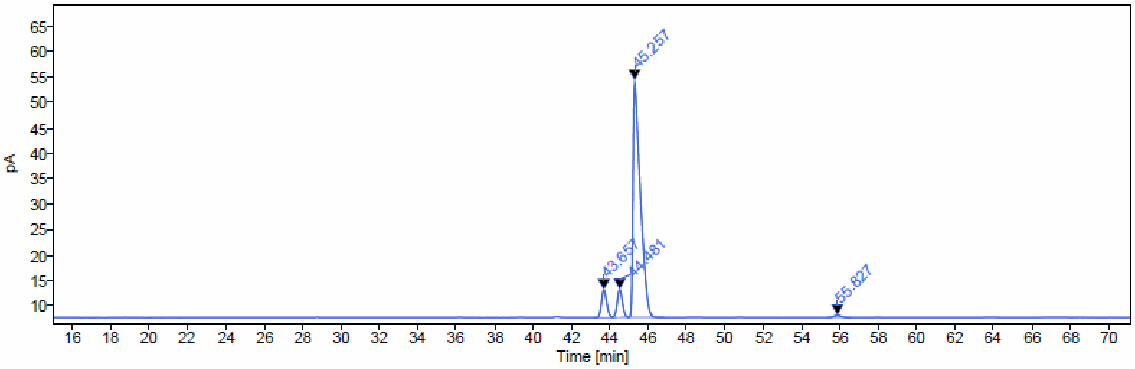

RT [min]	Type	Width [min]	Area	Height	Area%
43.657	MM m	0.23	101.77	5.44	7.02
44.481	MM m	0.31	103.97	5.51	7.17
45.257	MM m	0.31	1233.80	46.59	85.09
55.827	MM m	0.29	10.49	0.43	0.72
		Sum	1450.04		

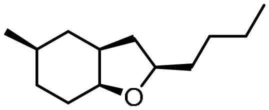

(2*R*,3a*S*,5*R*,7a*S*)-2-butyl-5-methyloctahydrobenzofuran (2u): The title compound was synthesized according to **GP-A**. The product was isolated by column chromatography using a gradient of *n*-pentane/EtOAc (100:0 to 98:2, *v*:*v*) and obtained as a colorless oil (53.5 mg, 0.273 mmol, 91%, >95:5 d.r., 96:4 e.r.).

^{1}H NMR (400 MHz, $CDCl_3$) *δ* 4.13–4.06 (m, 0.08H), 3.93–3.88 (m, 0.08H), 3.84–3.75 (m, 0.92H), 3.71–3.67 (m, 0.92H), 2.16 (ddd, *J* = 12.5, 9.2, 7.9 Hz, 1H), 2.10–2.02 (m, 1H), 2.00–1.90 (m, 1H), 1.77–1.67 (m, 1H), 1.63–1.52 (m, 1H), 1.52–1.41 (m, 2H), 1.40–1.24 (m, 6H), 1.21–1.09 (m, 2H), 0.92–0.88 (m, 4H), 0.86 (d, *J* = 6.4 Hz, 3H); signals of both diastereomers are listed. **^{13}C{^{1}H} NMR** (101 MHz, $CDCl_3$) *δ* 78.3, 76.7, 39.4, 38.8, 38.1, 37.2, 31.7, 29.4, 28.8, 28.1, 23.0, 22.8, 14.2; signals of the major diastereomer are listed. **HRMS** (EI) m/z calculated for [$C_{13}H_{23}O$] ($[M-H]^{+\cdot}$) 195.1743, found 195.1743. **Chiral GC-FID**

(50_5_90_0.05_110_20_220, *β*-Dex): t_1 = 117.9 min (major diastereomer, major enantiomer), t_2 = 119.5 min (major diastereomer, minor enantiomer).

Racemic sample:

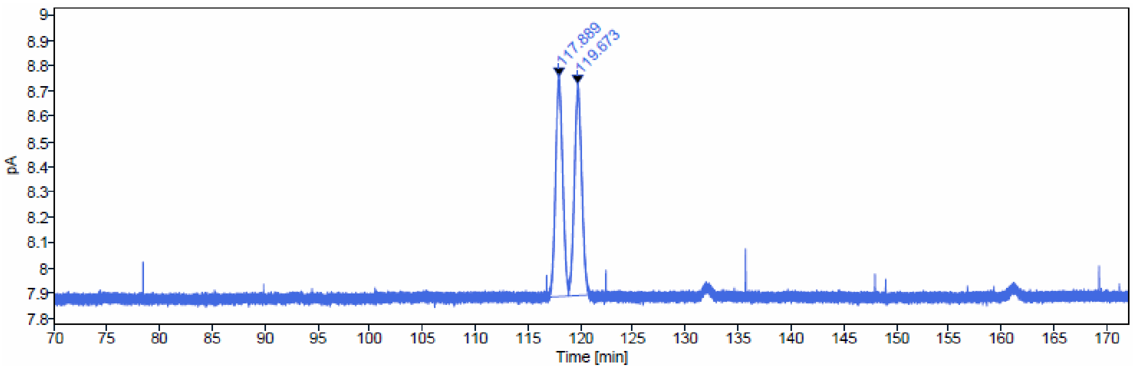

RT [min]	Type	Width [min]	Area	Height	Area%
117.889	MM m	0.79	41.07	0.86	50.17
119.673	MM m	0.82	40.79	0.83	49.83
		Sum	81.86		

Enantioenriched sample:

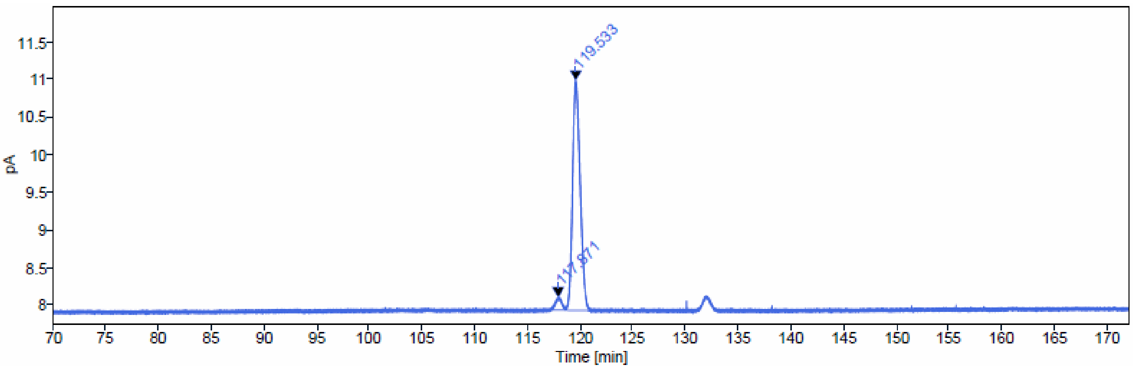

RT [min]	Type	Width [min]	Area	Height	Area%
117.871	MM m	0.49	6.73	0.16	4.18
119.533	MM m	0.59	154.13	3.05	95.82
		Sum	160.86		

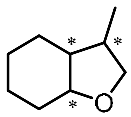

3-methyloctahydrobenzofuran (2v): The title compound was synthesized according to **GP-A**. The product was isolated by column chromatography using a mixture of *n*-pentane/Et_2O (90:10, *v*:*v*) and obtained as a colorless oil (40.5 mg, 0.289 mmol, 96%, 67:33 d.r., 94:6 e.r.).

^{1}H NMR (400 MHz, CDCl$_3$) δ 4.16–4.10 (m, 0.33H), 3.99–3.90 (m, 1.67H), 3.46 (dd, J = 10.2, 7.8 Hz, 0.67H), 3.34 (dd, J = 8.4, 5.6 Hz, 0.33H), 2.50–2.38 (m, 0.67H), 2.12–2.01 (m, 0.33H), 2.00–1.92 (m, 0.67H), 1.82–1.68 (m, 1.67H), 1.68–1.55 (m, 1H), 1.55–1.38 (m, 3H), 1.38–1.30 (m, 0.67H), 1.29–1.13 (m, 1H), 1.13–1.03 (m, 1H), 1.00 (d, J = 6.9 Hz, 1H), 0.94 (d, J = 6.9 Hz, 2H); signals of both diastereomers are listed. **^{13}C{^{1}H} NMR** (101 MHz, CDCl$_3$) δ 78.5, 72.5, 41.5, 38.2, 28.8, 24.7, 22.1, 20.7, 11.7; major diastereomer; 76.5, 74.1, 45.4, 37.8, 28.8, 26.9, 23.4, 21.7, 18.5; minor diastereomer. **HRMS** (EI) m/z calculated for [$C_9H_{16}O$] ([M]$^{+\cdot}$) 140.1201, found 140.1196. **Chiral GC-FID** (50_3_70_0.04_80_20_220, β-Dex): t_1 = 90.0 min (minor diastereomer, minor enantiomer), t_2 = 97.0 min (minor diastereomer, major enantiomer), t_3 = 108.4 min (major diastereomer, major enantiomer), t_4 = 112.4 min (major diastereomer, minor enantiomer).

Racemic sample:

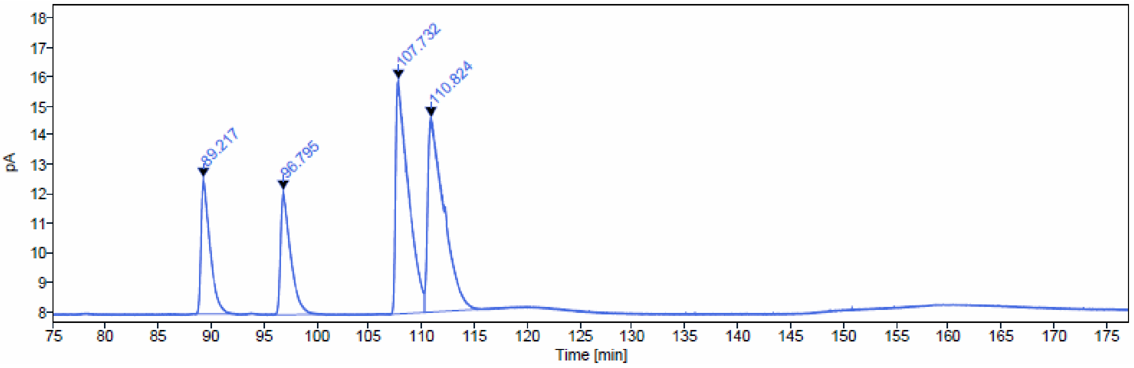

RT [min]	Type	Width [min]	Area	Height	Area%
89.217	MM m	0.69	270.38	4.62	14.08
96.795	MM m	0.76	274.04	4.21	14.27
107.732	MM m	0.99	671.51	7.97	34.97
110.824	MM m	1.25	704.41	6.62	36.68
		Sum	1920.33		

Enantioenriched sample:

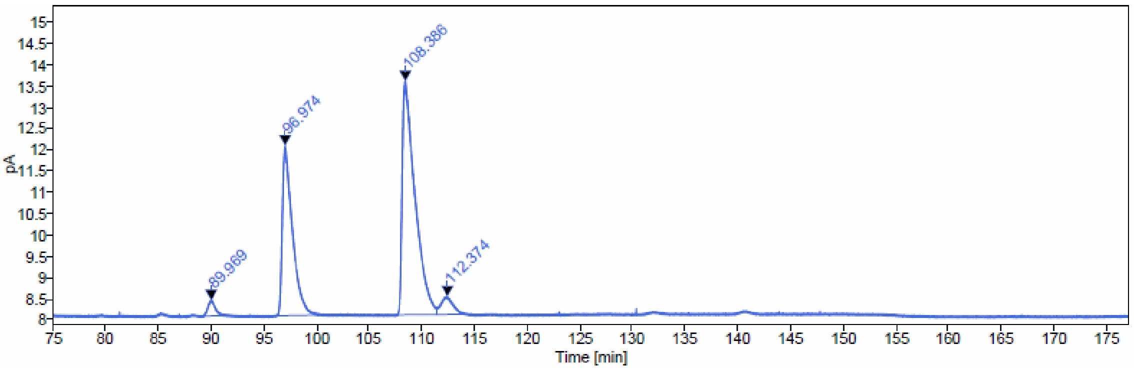

RT [min]	Type	Width [min]	Area	Height	Area%
89.969	MM m	0.78	17.10	0.37	2.20
96.974	MM m	0.79	268.35	3.99	34.51
108.386	MM m	0.98	458.89	5.50	59.02
112.374	MM m	0.92	33.23	0.42	4.27
		Sum	777.57		

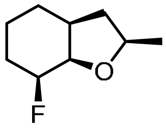

(2*R*,3a*S*,5*S*,7a*R*)-7-fluoro-2-methyloctahydrobenzofuran (2w): The title compound was synthesized according to **GP-B**. The product was isolated by column chromatography using a gradient of *n*-pentane/Et_2O (90:10 to 80:20, *v*:*v*) and obtained as a colorless oil (47.5 mg, 0.299 mmol, 99%, 90:10 d.r., 91:9 e.r.).

^{1}H NMR (500 MHz, $CDCl_3$) δ 4.72–4.55 (m, 1H), 4.43–4.35 (m, 0.15H), 4.16–4.07 (m, 1H), 3.95–3.89 (m, 0.85H), 2.27–2.19 (m, 1H), 2.07 (dddd, J = 12.2, 7.6, 7.2, 1.0 Hz, 1H), 1.98–1.87 (m, 1H), 1.78–1.64 (m, 2H), 1.60–1.51 (m, 1H), 1.43–1.36 (m, 1H), 1.34 (d, J = 6.2 Hz, 3H), 1.30–1.20 (m, 2H); signals of both diastereomers are listed. **^{1}H{^{19}F} NMR** (500 MHz, $CDCl_3$) δ 4.65 (ddd, J = 9.2, 3.7, 3.7 Hz, 0.85H), 4.63–4.59 (m, 0.15H), 4.43–4.36 (m, 0.15H), 4.15–4.07 (m, 1H), 3.92 (dd, J = 5.0, 3.6 Hz, 0.85H), 2.23 (dddd, J = 11.5, 8.7, 6.8, 5.1 Hz, 1H), 2.07 (ddd, J = 12.1, 7.7, 7.7 Hz, 1H), 1.97–1.87 (m, 1H), 1.76–1.67 (m, 2H), 1.60–1.51 (m, 1H), 1.43–1.36 (m, 1H), 1.34 (d, J = 6.1 Hz, 3H), 1.30–1.21 (m, 2H); signals of both diastereoers are listed. **^{13}C{^{1}H} NMR** (126 MHz, $CDCl_3$) δ 91.3 (d, J = 177.2 Hz), 78.2 (d, J = 15.2 Hz), 75.4, 39.2 (d, J = 3.7 Hz), 38.9 (d, J = 1.9 Hz), 27.6, 27.0 (d, J = 19.5 Hz), 22.1, 20.3 (d, J = 8.5 Hz); signals of the major diastereomer are listed. **^{13}C{^{1}H,^{19}F} NMR** (126 MHz, $CDCl_3$) δ 91.2, 78.2, 75.4, 39.2, 38.9, 27.6, 27.0, 22.1, 20.3; signals of the major diastereomer are listed. **^{19}F NMR** (470 MHz, $CDCl_3$) δ -183.3 (d, J = 46.5 Hz, minor), -184.1 (d, J = 47.0 Hz, major). **^{19}F{^{1}H} NMR** (376 MHz, CD_2Cl_2) δ -182.7 (minor), -183.5 (major). **HRMS** (ESI) m/z calculated for [$C_9H_{15}OFNa$] ([M+Na]$^+$) 181.1000, found 181.1008. **Chiral GC-FID** (50_5_90_0.05_110_20_220, *β*-Dex): t_1 = 66.2 min (minor diastereomer, major enantiomer), t_2 = 67.0 min (major diastereomer, minor enantiomer), t_3 = 69.4 min (major diastereomer, major enantiomer), t_4 = 79.4 min (minor diastereomer, minor enantiomer).

Racemic sample:

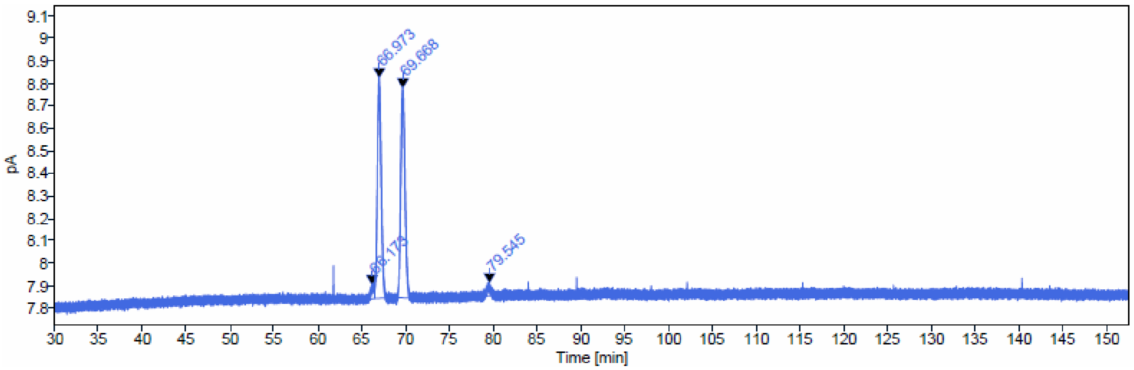

RT [min]	Type	Width [min]	Area	Height	Area%
66.173	MM m	0.29	1.39	0.06	2.22
66.973	MM m	0.37	30.05	0.97	47.96
69.668	MM m	0.38	29.90	0.92	47.73
79.545	MM m	0.30	1.31	0.05	2.09
		Sum	62.65		

Enantioenriched sample:

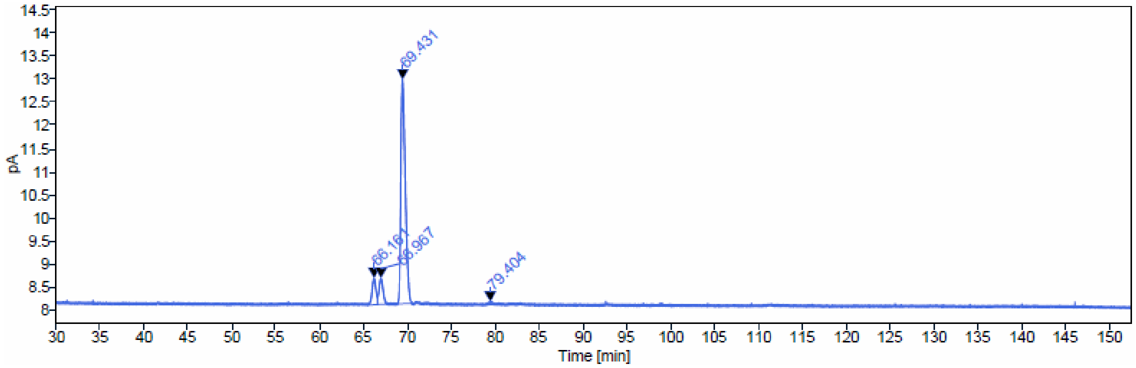

RT [min]	Type	Width [min]	Area	Height	Area%
66.161	MM m	0.35	17.21	0.57	8.44
66.967	MM m	0.36	17.27	0.56	8.46
69.431	MM m	0.41	167.82	4.87	82.26
79.404	MM m	0.36	1.71	0.06	0.84
		Sum	204.00		

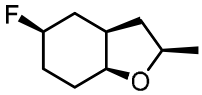

(2*R*,3a*R*,5*R*,7a*S*)-5-fluoro-2-methyloctahydrobenzofuran (2x): The title compound was synthesized according to **GP-B**. The product was isolated by column chromatography using a

mixture of *n*-pentane/EtOAc (95:5, *v*:*v*) and obtained as a colorless oil (34.2 mg, 0.216 mmol, 72%, >95:5 d.r., 95:5 e.r.).

^{1}H NMR (300 MHz, $CDCl_3$) δ 4.63–4.35 (m, 1H), 4.08–3.96 (m, 1H), 3.92–3.89 (m, 0.05H), 3.74–3.66 (m, 0.95H), 2.31–2.19 (m, 1H), 2.18–2.05 (m, 2H), 2.00–1.87 (m, 1H), 1.86–1.72 (m, 2H), 1.71–1.50 (m, 2H), 1.33 (d, *J* = 6.3 Hz, 3H), 1.36–1.29 (m, 1H); signals of both diastereomers are listed. **^{1}H{^{19}F} NMR** (500 MHz, $CDCl_3$) δ 4.52–4.43 (m, 1H), 4.10–4.07 (m, 0.10H), 4.06–3.97 (m, 0.90H), 3.92–3.88 (m, 0.10H), 3.72–3.67 (m, 0.10H), 2.24 (ddd, *J* = 12.5, 8.7, 7.7 Hz, 1H), 2.18–2.05 (m, 2H), 1.97–1.91 (m, 1H), 1.84–1.74 (m, 2H), 1.68–1.51 (m, 3H), 1.33 (d, *J* = 6.3 Hz, 3H); signals of both diastereomers are listed. **^{13}C{^{1}H} NMR** (126 MHz, $CDCl_3$) δ 91.3 (d, *J* = 171.3 Hz), 76.0 (d, *J* = 2.2 Hz), 74.3, 39.9, 37.6 (d, *J* = 9.6 Hz), 35.6 (d, *J* = 18.1 Hz), 27.3 (d, *J* = 19.2 Hz), 25.6 (d, *J* = 10.3 Hz), 22.9; signals of the major diastereomer are listed. **^{13}C{^{1}H,^{19}F} NMR** (126 MHz, $CDCl_3$) δ 91.3, 76.0, 74.3, 39.9, 37.6, 35.6, 27.3, 25.6, 22.; signals of both diastereomers are listed. **^{19}F NMR** (470 MHz, $CDCl_3$) δ -170.1– -170.3 (m, major), -170.5– -170.7 (m, minor). **^{19}F{^{1}H} NMR** (470 MHz, $CDCl_3$) δ -170.2 (major), -170.6 (minor). **HRMS** (ESI) m/z calculated for [$C_9H_{15}OFNa$] ([M+Na]$^+$) 181.1000, found 181.1005. **Chiral GC-FID** (50_5_100_2_170_20_220, *β*-Dex): t_1 = 28.8 min (major diastereomer, minor enantiomer), t_2 = 29.9 min (major diastereomer, major enantiomer).

Racemic sample:

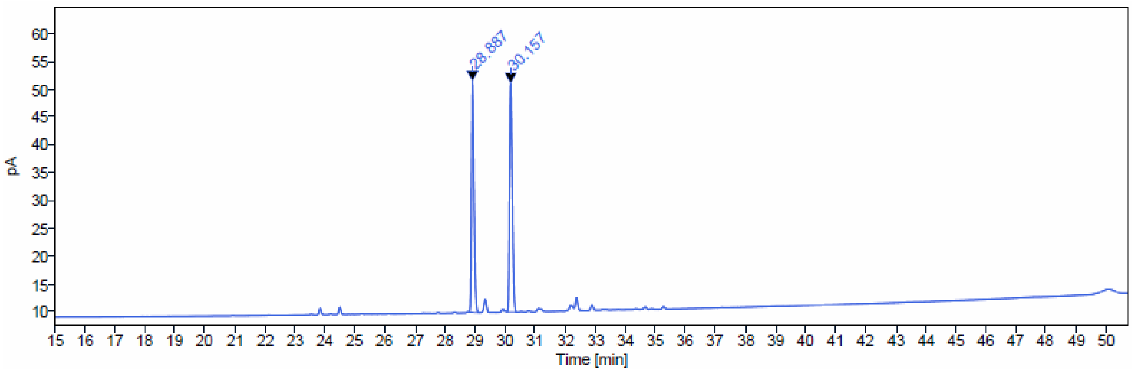

RT [min]	Type	Width [min]	Area	Height	Area%
28.887	BB	0.34	245.59	41.50	49.86
30.157	BB	0.34	246.99	41.05	50.14
		Sum	492.58		

Enantioenriched sample:

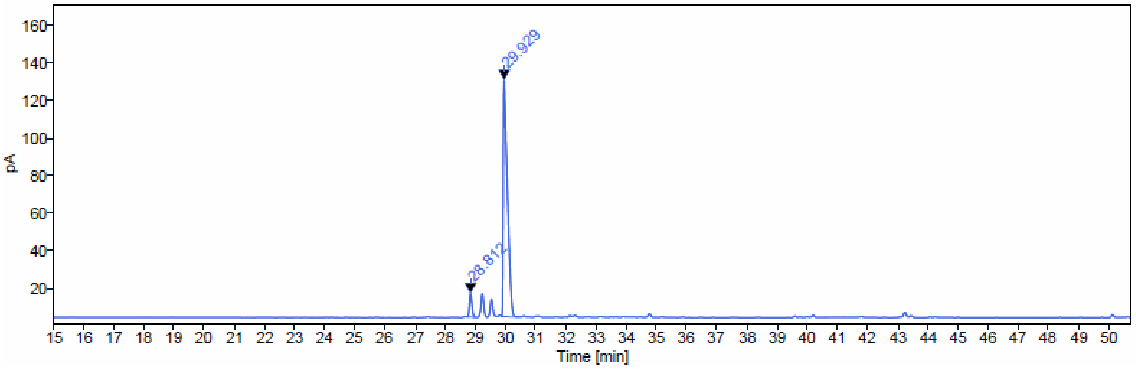

RT [min]	Type	Width [min]	Area	Height	Area%
28.812	MM m	0.09	68.60	12.48	5.49
29.929	MM m	0.12	1182.13	125.73	94.51
		Sum	1250.73		

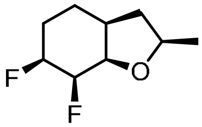

(2*R*,3a*S*,6*S*,7*R*,7a*R*)-6,7-difluoro-2-methyloctahydrobenzofuran (2y): The title compound was synthesized according to **GP-B**. The product was isolated by column chromatography using a gradient of *n*-pentane/Et_2O (90:10 to 80:20, *v*:*v*) and obtained as a colorless oil (41.2 mg, 0.234 mmol, 78%, >95:5 d.r., 86:14 e.r.).

^{1}H NMR (600 MHz, CD_2Cl_2) δ 4.78–4.64 (m, 1H), 4.60–4.57 (m, 0.08H), 4.56–4.41 (m, 1H), 4.39–4.33 (m, 0.08H), 4.16–4.09 (m, 0.92H), 3.97–3.89 (m, 0.92H), 2.43–2.36 (m, 1H), 2.05–1.97 (m, 1H), 1.90–1.82 (m, 2H), 1.73–1.64 (m, 1H), 1.62–1.55 (m, 1H), 1.53–1.46 (m, 1H), 1.29–1.26 (m, 3H); signals of both diastereomers are listed. **^{1}H{^{19}F} NMR** (600 MHz, CD_2Cl_2) δ 4.72–4.70 (m, 1H), 4.54 (dd, *J* = 6.4, 3.3 Hz, 0.08H), 4.51–4.47 (m, 1H), 4.38–4.34 (m, 0.08H), 4.16–4.09 (m, 0.92H), 3.93 (dd, *J* = 6.9, 3.7 Hz, 0.92H), 2.43–2.36 (m, 1H), 2.05–1.96 (m, 1H), 1.89–1.82 (m, 2H), 1.72–1.66 (m, 1H), 1.62–1.55 (m, 1H), 1.54–1.46 (m, 1H), 1.28 (d, *J* = 6.0 Hz, 3H); signals of both diastereomers are listed. **^{13}C{^{1}H} NMR** (151 MHz, CD_2Cl_2) δ 90.2 (dd, *J* = 183.1, 15.8 Hz), 90.0 (dd, *J* = 182.4, 18.5 Hz), 77.5 (dd, *J* = 16.4, 6.7 Hz), 76.8, 38.1 (d, *J* = 2.9 Hz), 37.9, 22.4 (dd, *J* = 19.5, 5.7 Hz), 21.3 (d, *J* = 10.2 Hz), 20.9 (d, *J* = 1.0 Hz); signals of the major diastereomer are listed. **^{13}C{^{1}H,^{19}F} NMR** (151 MHz, CD_2Cl_2) δ 90.1, 90.0, 77.5, 76.8, 38.1, 37.9, 22.4, 21.3, 20.9; signals of the major diastereomer are listed. **^{19}F{^{1}H} NMR** (564 MHz, CD_2Cl_2) δ -189.2 (major), -205.9 (major). **HRMS** (EI) m/z calculated for [$C_9H_{14}OF_2$] ([M]$^{+\cdot}$) 176.1007, found 176.1008. **Chiral GC-FID** (50_5_100_0.1_120_20_220,

β-Dex): t_1 = 75.4 min (major diastereomer, minor enantiomer), t_2 = 80.3 min (major diastereomer, major enantiomer).

Racemic sample:

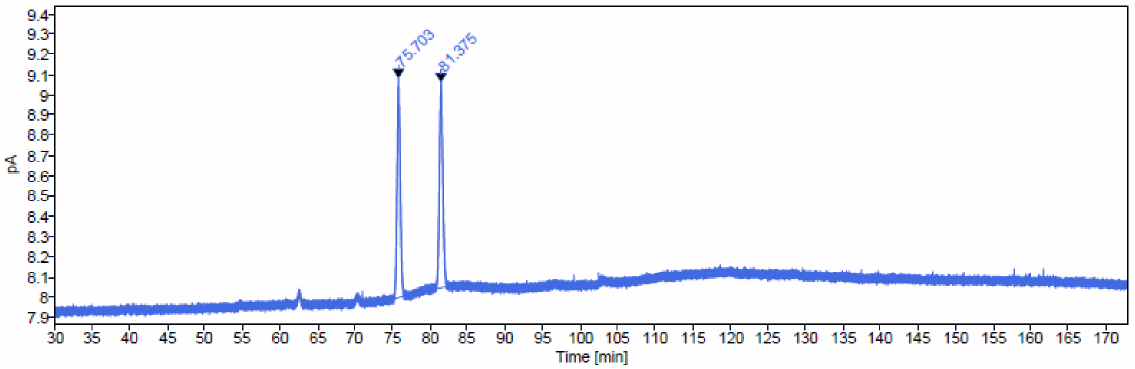

RT [min]	Type	Width [min]	Area	Height	Area%
75.703	MM m	0.52	33.36	1.08	49.67
81.375	MM m	0.39	33.80	1.01	50.33
		Sum	67.16		

Enantioenriched sample:

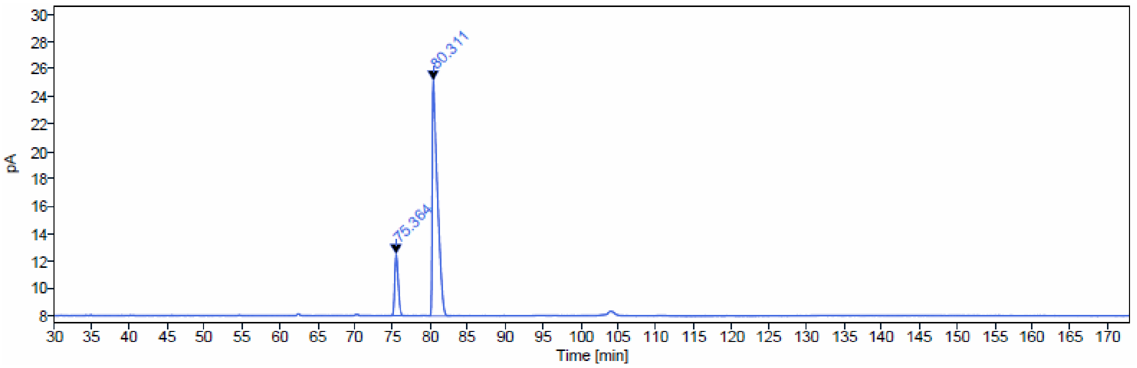

RT [min]	Type	Width [min]	Area	Height	Area%
75.364	MM m	0.52	138.50	4.44	14.32
80.311	MM m	0.81	828.74	17.11	85.68
		Sum	967.25		

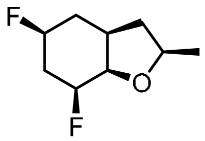

(2*R*,3a*R*,5*S*,7*S*,7a*R*)-5,7-difluoro-2-methyloctahydrobenzofuran (2z): The title compound was synthesized according to **GP-B**. The product was isolated by column chromatography

using a gradient of *n*-pentane/Et_2O (90:10 to 80:20, *v*:*v*) and obtained as a colorless oil (42.1 mg, 0.239 mmol, 80%, 92:8 d.r., 89:11 e.r.).

^{1}H NMR (600 MHz, CD_2Cl_2) δ 4.72–4.60 (m, 1H), 4.57–4.43 (m, 1H), 4.12–4.05 (m, 1H), 3.90–3.84 (m, 1H), 2.33–2.24 (m, 2H), 2.18–2.05 (m, 2H), 2.00–1.93 (m, 1H), 1.57–1.48 (m, 1H), 1.37–1.32 (m, 4H); signals of both diastereomers are listed. **^{1}H{^{19}F} NMR** (600 MHz, CD_2Cl_2) δ 4.66 (ddd, *J* = 10.7, 4.7, 3.5 Hz, 1H), 4.53–4.47 (m, 1H), 4.12–4.05 (m, 1H), 3.87 (dd, *J* = 4.0, 4.0 Hz, 1H), 2.32–2.24 (m, 2H), 2.19–2.13 (m, 1H), 2.13–2.07 (m, 1H), 1.97 (dddd, *J* = 12.7, 6.3, 4.5, 1.9 Hz, 1H), 1.56–1.49 (m, 1H), 1.34 (d, *J* = 6.3 Hz, 3H), 1.38–1.31 (m, 1H); signals of both diastereomers are listed. **^{13}C{^{1}H} NMR** (151 MHz, CD_2Cl_2) δ 89.3 (dd, *J* = 180.4, 13.6 Hz), 88.7 (dd, *J* = 174.3, 12.7 Hz), 77.4 (dd, *J* = 15.1, 1.7 Hz), 75.4, 39.5 (dd, *J* = 1.4, 1.4 Hz), 35.7 (dd, *J* = 11.3, 5.8 Hz), 35.0 (d, *J* = 18.5 Hz), 33.5 (d, *J* = 20.5 Hz), 22.4; signals of the major diastereomer are listed. **^{13}C{^{1}H,^{19}F} NMR** (151 MHz, CD_2Cl_2) δ 89.3, 88.7, 77.3, 75.4, 39.5, 35.7, 35.0, 33.5, 22.4; signals of the major diastereomer are listed. **^{19}F NMR** (564 MHz, CD_2Cl_2) δ -173.6– -173.9 (m, major), -174.4– -174.6 (m, minor), -186.52– -186.73 (m, major), -186.87– -187.02 (m, minor). **^{19}F{^{1}H} NMR** (564 MHz, CD_2Cl_2) δ -173.8 (d, *J* = 4.5 Hz, major), -174.5 (d, *J* = 4.2 Hz, minor), -186.6 (d, *J* = 4.5 Hz, major), -187.0 (d, *J* = 4.3 Hz, minor). **HRMS** (EI) m/z calculated for [$C_9H_{14}OF_2$] ([M]$^{+\cdot}$) 176.1007, found 176.1007. **Chiral GC-FID** (50_5_100_0.1_120_20_220, *β*-Dex): t_1 = 62.8 min (major diastereomer, minor enantiomer), t_2 = 68.0 min (major diastereomer, major enantiomer).

Racemic sample:

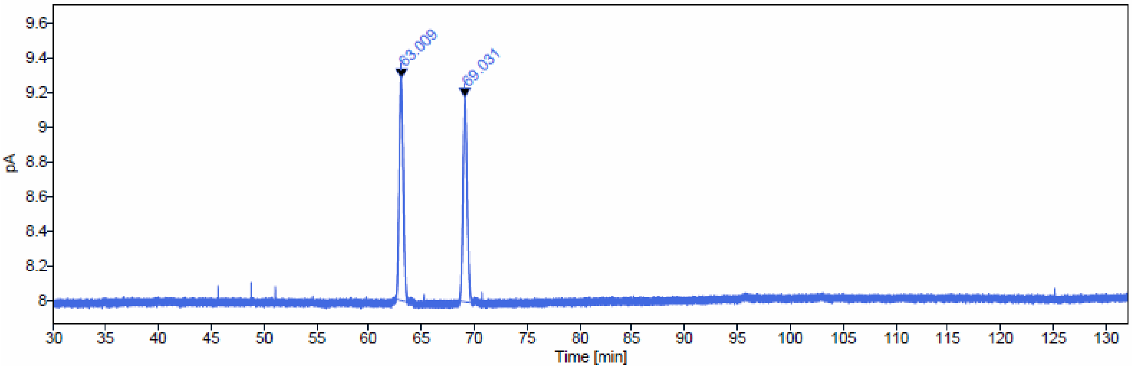

RT [min]	Type	Width [min]	Area	Height	Area%
63.009	MM m	0.42	32.33	1.28	49.69
69.031	MM m	0.33	32.73	1.17	50.31
		Sum	65.06		

Enantioenriched sample:

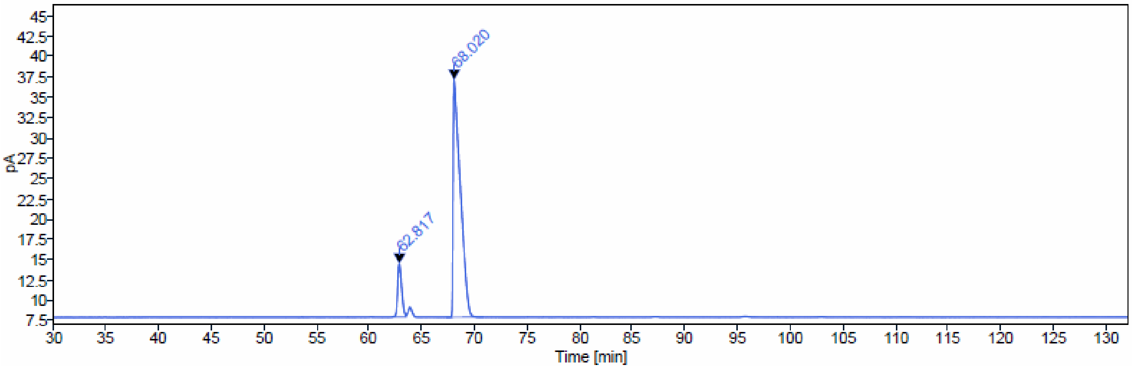

RT [min]	Type	Width [min]	Area	Height	Area%
62.817	MM m	0.31	174.12	6.52	11.19
68.020	MM m	0.79	1382.31	29.14	88.81
		Sum	1556.43		

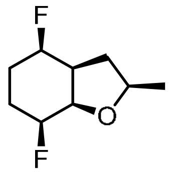

(2*R*,3a*S*,4*R*,7*S*,7a*R*)-4,7-difluoro-2-methyloctahydrobenzofuran (2aa): The title compound was synthesized according to **GP-B**. The product was isolated by column chromatography using a gradient of *n*-pentane/Et_2O (90:10 to 80:20, *v*:*v*) and obtained as a colorless oil (38.5 mg, 0.218 mmol, 73%, 94:6 d.r., 90:10 e.r.).

^{1}H NMR (600 MHz, CD_2Cl_2) δ 4.77–4.64 (m, 1H), 4.64–4.52 (m, 1H), 4.43–4.36 (m, 0.15H), 4.16–4.10 (m, 0.85H), 4.09–4.05 (m, 0.15H), 3.96–3.91 (m, 0.85H), 2.74–2.65 (m, 0.85H), 2.65–2.58 (m, 0.15H), 2.29–2.23 (m, 0.15H), 2.12 (dddd, *J* = 17.8, 11.3, 7.6, 3.7 Hz, 1H), 2.07–1.97 (m, 1.85H), 1.72–1.57 (m, 2H), 1.56–1.44 (m, 1H), 1.29 (d, *J* = 6.1 Hz, 2.55H), 1.19 (d, *J* = 6.3 Hz, 0.45H); signals of both diastereomers are listed. **^{1}H{^{19}F} NMR** (600 MHz, CD_2Cl_2) δ 4.73–4.68 (m, 1H), 4.64–4.61 (m, 0.15H), 4.60–4.56 (m, 0.85H), 4.42–4.36 (m, 0.15H), 4.16–4.10 (m, 0.85H), 4.09–4.06 (m, 0.15H), 3.94 (dd, *J* = 6.3, 3.9 Hz, 0.85H), 2.72–2.67 (m, 0.85H), 2.64–2.59 (m, 0.15H), 2.26 (ddd, *J* = 12.4, 8.0, 6.5 Hz, 0.15H), 2.12 (ddd, *J* = 14.4, 7.4, 3.6 Hz, 0.85H), 2.09–1.99 (m, 2H), 1.71–1.63 (m, 1.70H), 1.63–1.57 (m, 0.30H), 1.54–1.47 (m, 1H), 1.29 (d, *J* = 6.1 Hz, 2.55H), 1.19 (d, *J* = 6.3 Hz, 0.45H); signals of both diastereomers are listed. **^{13}C{^{1}H} NMR** (151 MHz, CD_2Cl_2) δ 89.5 (dd, *J* = 175.1, 0.9 Hz), 89.0 (dd, *J* = 177.7, 1.4 Hz), 78.0 (dd, *J* = 15.9, 4.9 Hz), 76.4, 43.9 (dd, *J* = 19.2, 1.8 Hz), 33.7 (dd, *J* = 4.3, 2.4 Hz), 23.9 (dd, *J* = 21.2, 7.0 Hz), 23.3 (dd, *J* = 21.0, 8.3 Hz), 20.1; signals of the major diastereomer are listed. **^{13}C{^{1}H,^{19}F} NMR** (151 MHz, CD_2Cl_2) δ 89.0, 89.0, 78.0, 76.4, 43.9, 33.7, 23.9, 23.2,

20.1; signals of the major diastereomer are listed. **^{19}F NMR** (564 MHz, CD_2Cl_2) δ -180.8– -181.1 (m, major), -184.9 (m, minor), -187.9– -188.2 (m, major), -188.76 (m, minor). **^{19}F{^{1}H} NMR** (564 MHz, CD_2Cl_2) δ -180.9 (major), -184.9 (minor), -188.1 (major), -188.8 (minor). **HRMS** (EI) m/z calculated for $[C_9H_{14}OF_2]$ ($[M]^{+\cdot}$) 176.1007, found 176.1007. **Chiral GC-FID** (50_5_100_0.1_120_20_220, β-Dex): t_1 = 67.7 min (major diastereomer, minor enantiomer), t_2 = 78.4 min (major diastereomer, major enantiomer).

Racemic sample:

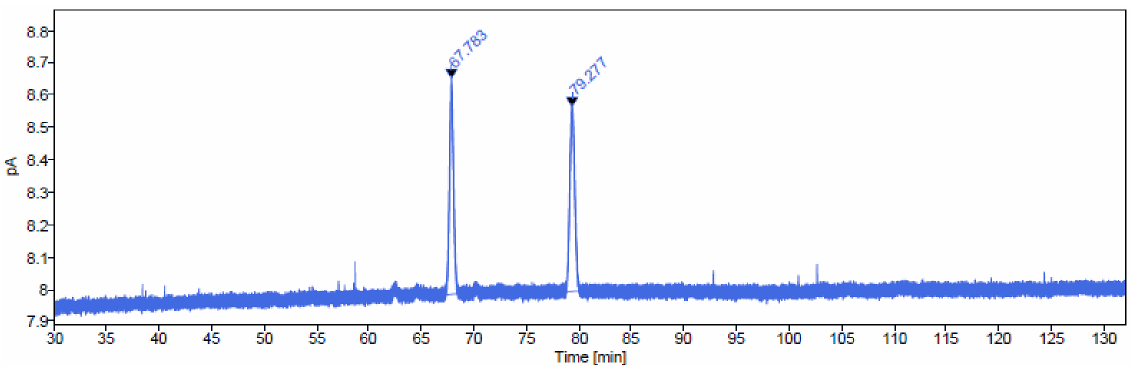

RT [min]	Type	Width [min]	Area	Height	Area%
67.783	MM m	0.46	18.46	0.66	50.49
79.277	MM m	0.37	18.10	0.57	49.51
		Sum	36.56		

Enantioenriched sample:

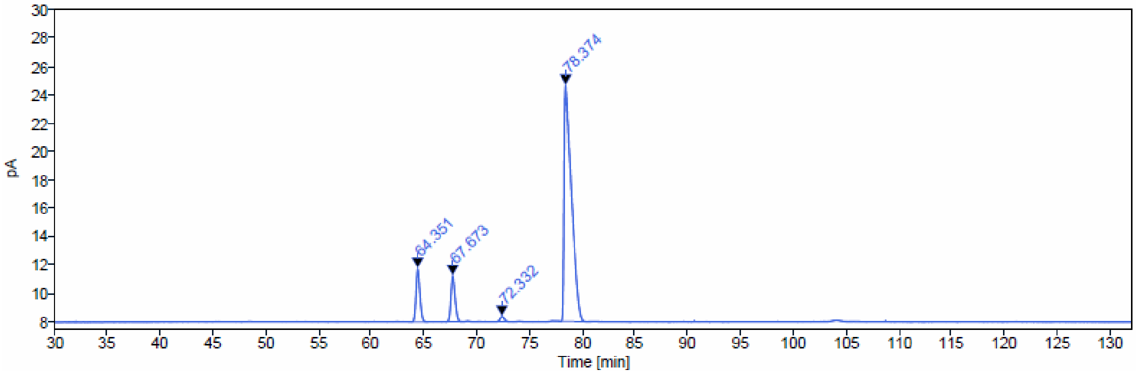

RT [min]	Type	Width [min]	Area	Height	Area%
64.351	MM m	0.45	101.96	3.79	10.24
67.673	MM m	0.33	91.84	3.26	9.22
72.332	MM m	0.32	10.41	0.38	1.04
78.374	MM m	0.56	791.65	16.69	79.49
		Sum	995.86		

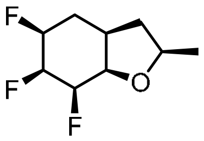

(2*R*,3a*R*,5*S*,6*S*,7*R*,7a*R*)-5,6,7-trifluoro-2-methyloctahydrobenzofuran (2ab): The title compound was synthesized according to **GP-B**. The product was isolated by column chromatography using a gradient of *n*-pentane/Et_2O (90:10 to 30:70, *v*:*v*) and obtained as a white solid (44.2 mg, 0.228 mmol, 76%, >95:5 d.r., 88:12 e.r.).

^{1}H NMR (600 MHz, CD_2Cl_2) δ 5.03–4.88 (m, 1H), 4.69–4.50 (m, 2H), 4.10–4.03 (m, 1H), 4.02–3.97 (m, 1H), 2.29–2.18 (m, 2H), 2.07–1.98 (m, 1H), 1.90–1.82 (m, 1H), 1.50–1.44 (m, 1H), 1.33 (d, *J* = 6.2 Hz, 3H). **^{1}H{^{19}F} NMR** (600 MHz, CD_2Cl_2) δ 4.96–4.94 (m, 1H), 4.61 (dd, *J* = 4.3, 2.6 Hz, 1H), 4.58 (ddd, *J* = 10.5, 4.4, 2.1 Hz, 1H), 4.09–4.03 (m, 1H), 4.00 (dd, *J* = 4.3, 4.3 Hz, 1H), 2.28–2.18 (m, 2H), 2.06–1.99 (m, 1H), 1.88–1.83 (m, 1H), 1.49–1.44 (m, 1H), 1.33 (d, *J* = 6.2 Hz, 3H). **^{13}C{^{1}H} NMR** (151 MHz, CD_2Cl_2) δ 89.1–87.4 (m, 3C), 76.9 (ddd, *J* = 15.6, 1.8, 0.8 Hz), 75.8, 38.8 (dd, *J* = 1.8, 1.8 Hz), 35.3 (dd, *J* = 9.1, 4.8 Hz), 28.4 (dd, *J* = 18.9, 4.8 Hz), 22.2. **^{13}C{^{1}H,^{19}F} NMR** (151 MHz, CD_2Cl_2) δ 88.3, 88.3, 88.1, 76.9, 75.8, 38.9, 35.3, 28.4, 22.2. **^{19}F NMR** (564 MHz, CD_2Cl_2) δ -189.9– -190.4 (m), -201.7– -202.0 (m), -212.0– -212.5 (m). **^{19}F{^{1}H} NMR** (564 MHz, CD_2Cl_2) δ -190.1 (dd, *J* = 13.4, 10.3 Hz), -201.9 (dd, *J* = 13.9, 10.2 Hz), -212.2– -212.3 (m). **HRMS** (EI) m/z calculated for [$C_9H_{13}OF_3$] ([M]$^{+\cdot}$) 194.0913, found 194.0912. **Chiral GC-FID** (50_5_90_0.05_110_20_220, *β*-Dex): t_1 = 254.1 min (major diastereomer, minor enantiomer), t_2 = 282.6 min (major diastereomer, major enantiomer).

Racemic sample:

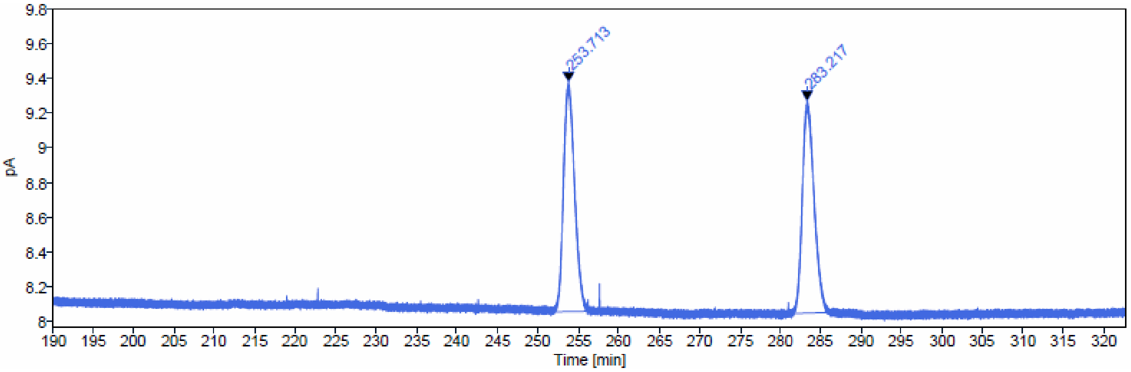

RT [min]	Type	Width [min]	Area	Height	Area%
253.713	MM m	1.11	124.29	1.31	50.35
283.217	MM m	1.18	122.54	1.21	49.65
		Sum	246.83		

Enantioenriched sample:

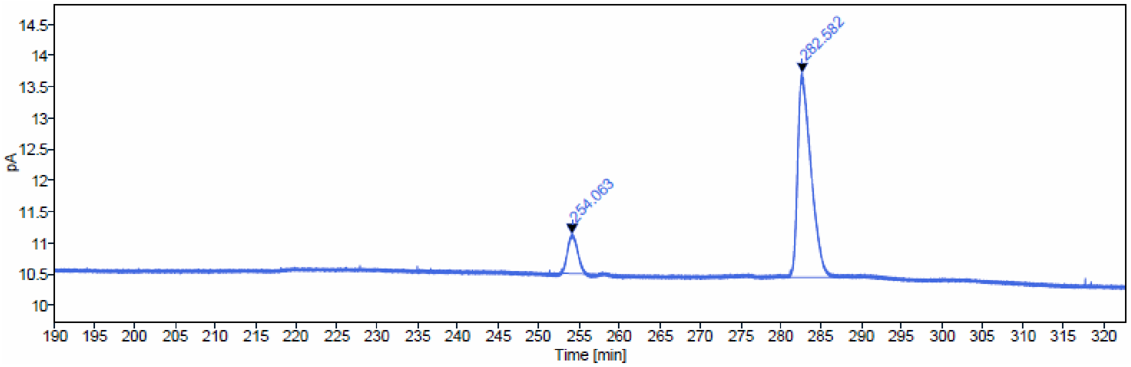

RT [min]	Type	Width [min]	Area	Height	Area%
254.063	MM m	1.33	50.27	0.63	11.76
282.582	MM m	1.35	377.18	3.27	88.24
		Sum	427.45		

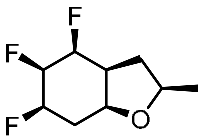

(2*R*,3a*S*,4*S*,5*R*,6*R*,7a*S*)-4,5,6-trifluoro-2-methyloctahydrobenzofuran (2ac): The title compound was synthesized according to **GP-B**. The product was isolated by column chromatography using a gradient of *n*-pentane/Et_2O (80:20 to 30:70, *v*:*v*) and obtained as a colorless oil (23.1 mg, 0.189 mmol, 40%, >95:5 d.r., 88:12 e.r.).

^{1}H NMR (500 MHz, CD_2Cl_2) δ 5.06–4.86 (m, 1H), 4.80–4.63 (m, 1H), 4.63–4.45 (m, 1H), 4.09–3.98 (m, 2H), 2.81–2.71 (m, 1H), 2.22–2.05 (m, 3H), 1.92–1.83 (m, 1H), 1.28 (d, *J* = 6.1 Hz, 3H). **^{1}H{^{19}F} NMR** (500 MHz, CD_2Cl_2) δ 4.97–4.95 (m, 1H), 4.71 (dd, *J* = 6.4, 2.6 Hz, 1H), 4.54 (dddd, *J* = 10.1, 4.8, 2.1, 0.8 Hz, 1H), 4.08–3.98 (m, 2H), 2.80–2.72 (m, 1H), 2.22–2.06 (m, 3H), 1.91–1.84 (m, 1H), 1.28 (d, *J* = 6.1 Hz, 3H). **^{13}C{^{1}H} NMR** (126 MHz, CD_2Cl_2) δ 89.6 (ddd, *J* = 188.3, 17.7, 17.7 Hz), 87.7 (ddd, *J* = 185.7, 16.6, 8.6 Hz), 86.3 (ddd, *J* = 184.8, 18.1, 7.6 Hz), 76.3, 73.1 (dd, *J* = 11.6, 7.3 Hz), 42.6 (dd, *J* = 19.8, 1.2 Hz), 34.6 (dd, *J* = 4.5, 3.2 Hz), 31.3 (dd, *J* = 18.1, 4.9 Hz), 22.0. **^{13}C{^{1}H,^{19}F} NMR** (126 MHz, CD_2Cl_2) δ 89.6, 87.7, 86.3, 76.3, 73.1, 42.6, 34.6, 31.3, 22.0. **^{19}F NMR** (470 MHz, CD_2Cl_2) δ -194.2– -194.5 (m), -199.0– -199.3 (m), -209.5– -209.9 (m). **^{19}F{^{1}H} NMR** (470 MHz, CD_2Cl_2) δ -194.3 (dd, *J* = 13.3, 9.4 Hz), -199.1 (dd, *J* = 14.0, 9.4 Hz), -209.7 (dd, *J* = 13.7, 13.7 Hz). **HRMS** (EI) m/z calculated for [$C_9H_{13}OF_3$] ([M]$^{+\cdot}$) 194.0913, found 194.0913. **Chiral GC-FID** (50_5_90_0.05_110_20_220, *β*-Dex): t_1 = 163.7 min (major diastereomer, minor enantiomer), t_2 = 194.8 min (major diastereomer, major enantiomer).

Racemic sample:

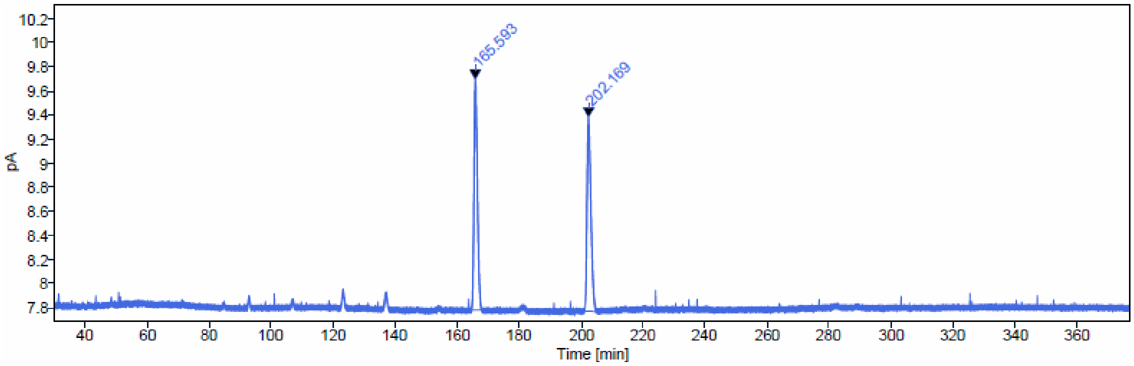

RT [min]	Type	Width [min]	Area	Height	Area%
165.593	MM m	0.84	136.96	1.91	50.24
202.169	MM m	0.99	135.66	1.61	49.76
		Sum	272.61		

Enantioenriched sample:

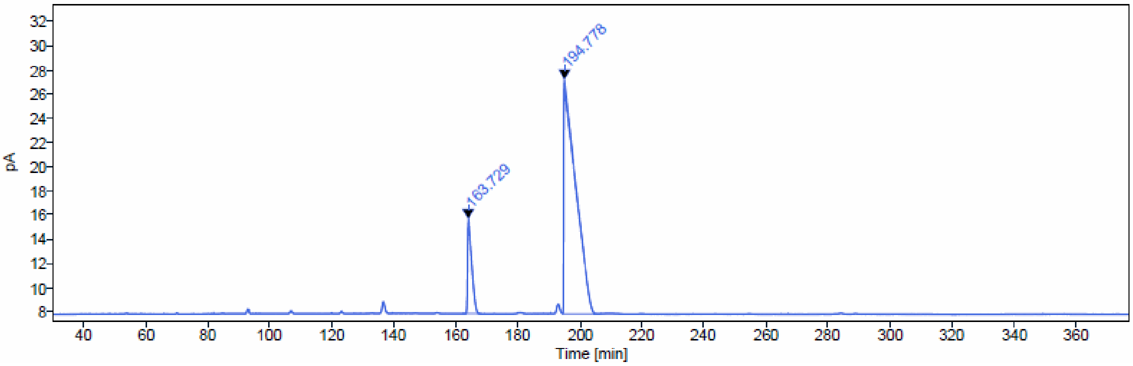

RT [min]	Type	Width [min]	Area	Height	Area%
163.729	MM m	1.09	721.32	7.75	12.46
194.778	MM m	3.07	5066.78	19.30	87.54
		Sum	5788.10		

6. Mechanistic Considerations

6.1 Control experiments to investigate the role of both used catalysts:

Control experiments were conducted with 0.10 mmol of 2,5-dimethylbenzofuran **2b** according to **GP-A** with modified reaction conditions in each case (**Scheme S1**). Yields, diastereomeric and enantiomeric ratios were determined by GC-FID analysis.

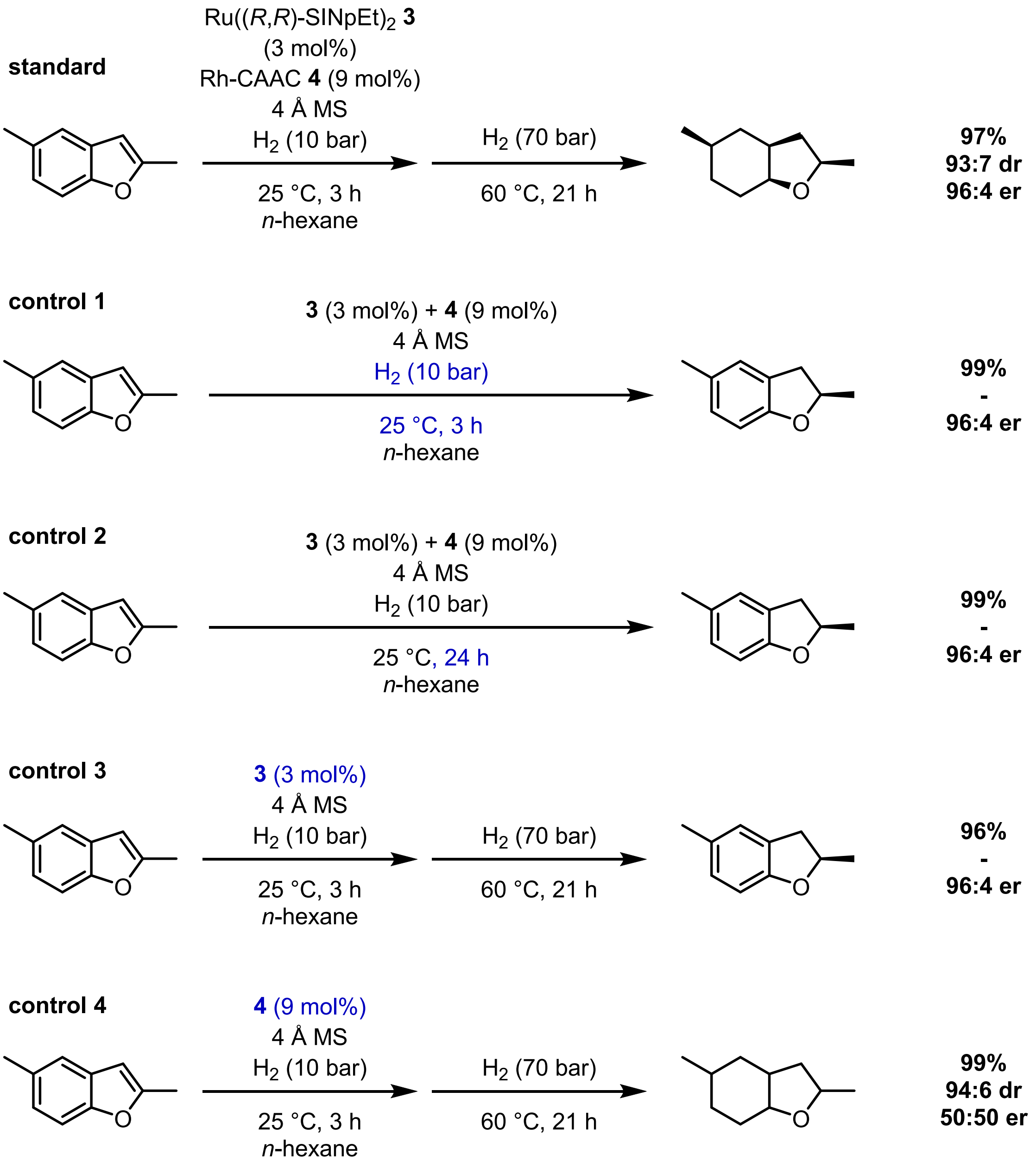

Scheme S1: Control reactions to study the role of employed catalysts. Yield, d.r., and e.r. were determined by GC-FID.

For **control 1** the reaction mixture was stirred at a hydrogen pressure of 10 bar and a temperature of 25 °C for 3 h after which the reaction was stopped and analyzed. As result a

quantitative yield of the dihydro-intermediate was obtained with an enantiomeric ratio of 96:4, which is identical to the enantiomeric ratio obtained for the octahydro-product under standard reaction conditions. This indicates that the first partial hydrogenation of the furanyl ring is completed before the reaction conditions are altered to activate the rhodium catalyst precursor.

In **control 2** the initial reaction conditions of 10 bar hydrogen pressure and 25 °C are kept for the full reaction time of 24 h. An identical outcome to the first control reaction is obtained. This shows, that the elevated reaction temperature and pressure are needed to activate the rhodium catalyst.

In **control 3** no Rh-CAAC complex **4** was added but the other standard conditions were applied. This led to the exclusive formation of the dihydro-intermediate in an unaltered enantiomeric ratio when compared to the product of the standard reaction. The obtained reaction mixture still showed the characteristic color of complex **3**. Thus, it can be excluded that the ruthenium catalyst is reducing the six membered ring at elevated temperature and hydrogen pressure.

Lastly, in **control 4** solely Rh-CAAC **4** (9 mol%) was used under otherwise unchanged standard reaction conditions. The octahydrobenzofuran was obtained in quantitative yield with essentially unchanged diastereomeric ratio, but as racemic mixture.

The results of the conducted control experiments indicate that the used ruthenium precursor complex is activated and catalyzes the partial hydrogenation of the furanyl ring within the first 3 h of reaction time. This observation is consistent with the findings of previous studies in which the authors found an incubation time of ~1 h with a subsequent very fast hydrogenation of 2-methylbenzofuran under similar reaction conditions.[12] Since the obtained enantiomeric ratios of **controls 1** and **3** are identical, a racemic background reaction catalyzed by a rhodium species can be excluded. To obtain a fully saturated product a change in the reaction conditions is needed.

6.2 Analysis of the stereochemical outcome in stepwise procedures

To verify that no enantioinformation is lost during the hydrogenation of dihydro-intermediates to the fully saturated products, hydrogenation of **1a** and **1x** has been performed in a stepwise manner with analysis of intermediates and products (**Scheme S2**).

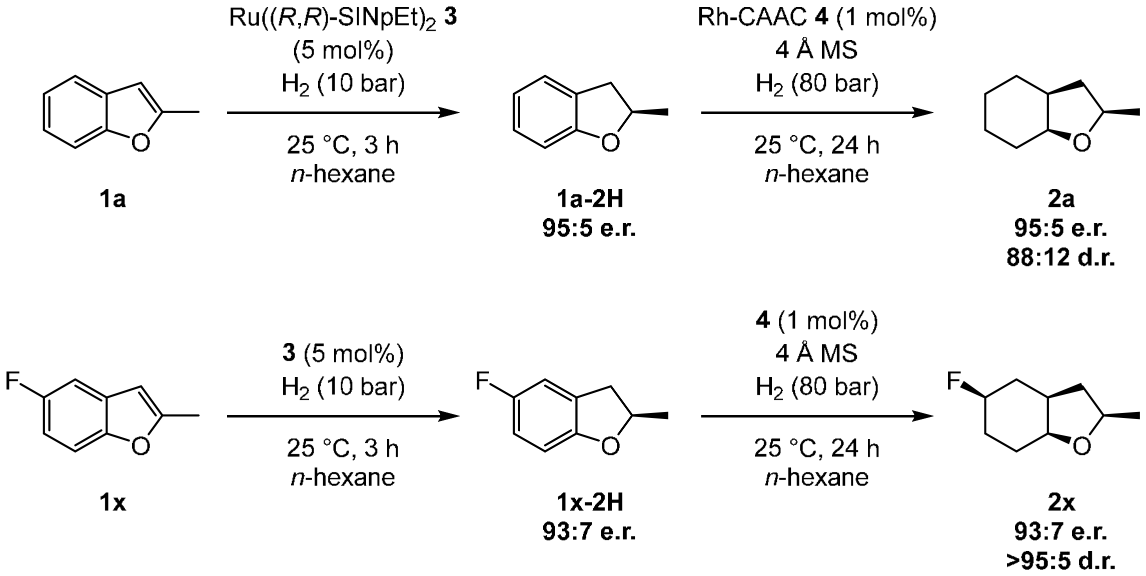

Scheme S2: Control reactions to study the stereochemical outcome of a stepwise hydrogenation.

The hydrogenation of 2-methylbenzofuran (**1a**) to 2-methyl-2,3-dihydrobenzofuran (**1a-2HB**) with Ru-((*R*,*R*)SINpEt)$_2$ **3** produced the intermediate product in 95:5 e.r. After isolation, the dihydrobenzofuran was submitted to hydrogenation conditions catalyzed by Rh-CAAC **4**. The resulting product was obtained in an unaltered 95:5 e.r. The obtained major diastereomer was identical to the major diastereomer obtained under optimized one-pot conditions for compound **2a**. The observed enantiomeric excess of 95:5 in this stepwise process is comparable to the observed 96:4 e.r. for **2a** in the optimized one-pot protocol.

Similar results were observed for the stepwise hydrogenation of fluoro substituted **1x** with an enantiomeric ratio of 93:7 observed after both single steps.

These results show that no enantioinformation of the dihydro-intermediates set by the chiral ruthenium catalyst is lost during the hydrogenation of the six-membered ring. Furthermore, the obtained major diastereomer of a step-wise hydrogenation is identical to the major diastereomer obtained in the developed one-pot procedure.

2-Methyl-2,3-dihydrobenzofuran **1a-2H**

The obtained characterization data was identical to the literature data.[4]

Chiral HPLC-UV (OD-H, hexane:*i*PrOH = 99:1, 0.5 mL/min, 360 nm): t_1 = 11.1 min, t_2 = 12.0 min.

Racemic sample:

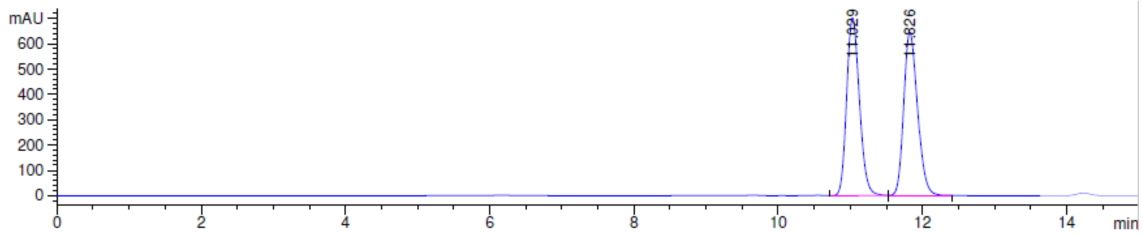

Peak #	RetTime [min]	Type	Width [min]	Area [mAU*s]	Height [mAU]	Area %
1	11.029	VV	0.1897	8585.84375	701.73840	49.9039
2	11.826	VB	0.2080	8618.90625	644.75403	50.0961

Totals : 1.72048e4 1346.49243

Enantioenriched sample:

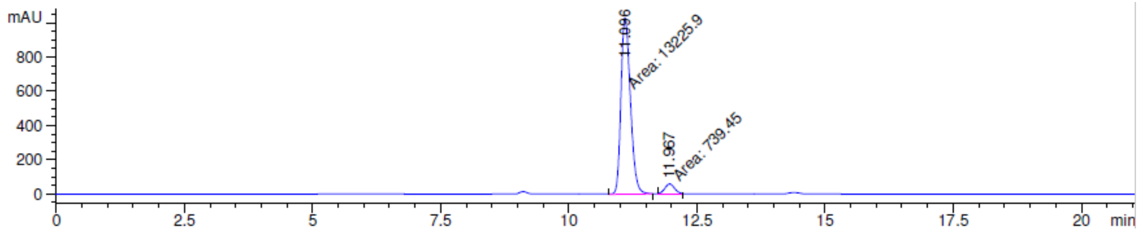

Peak #	RetTime [min]	Type	Width [min]	Area [mAU*s]	Height [mAU]	Area %
1	11.096	MM	0.2139	1.32259e4	1030.36060	94.7051
2	11.967	MM	0.2186	739.44983	56.38134	5.2949

Totals : 1.39653e4 1086.74193

5-Fluoro-2-methyl-2,3-dihydrobenzofuran **1x-2H**

^{1}H NMR (300 MHz, $CDCl_3$) δ 6.86 (ddd, *J* = 8.1, 2.8, 1.3 Hz, 1H), 6.78 (td, *J* = 8.9, 2.8 Hz, 1H), 6.64 (dd, *J* = 8.6, 4.2 Hz, 1H), 5.01–4.83 (m, 1H), 3.29 (dd, *J* = 15.7, 8.7 Hz, 1H), 2.87–2.72 (m, 1H), 1.46 (d, *J* = 6.2 Hz, 3H). **^{13}C{^{1}H} NMR** (101 MHz, $CDCl_3$) δ 157.4 (d, *J* = 236.2 Hz),

155.6 (d, J = 1.5 Hz), 128.5 (d, J = 8.8 Hz), 114.0 (d, J = 24.0 Hz), 112.2 (d, J = 24.6 Hz), 109.4 (d, J = 8.6 Hz), 80.3, 37.5 (d, J = 1.7 Hz), 21.8. **^{19}F NMR** (376 MHz, $CDCl_3$) δ -125.0. **GC-MS** (EI): 152 (100) $[M]^+$, 137 (52) $[M-CH_3]^+$, 133 (29) $[M-F]^+$, 109 (95). **Chiral HPLC-UV** (OD-H, hexane:*i*PrOH = 99:1, 0.5 mL/min, 360 nm): t_1 = 9.8 min, t_2 = 10.5 min.

Racemic sample:

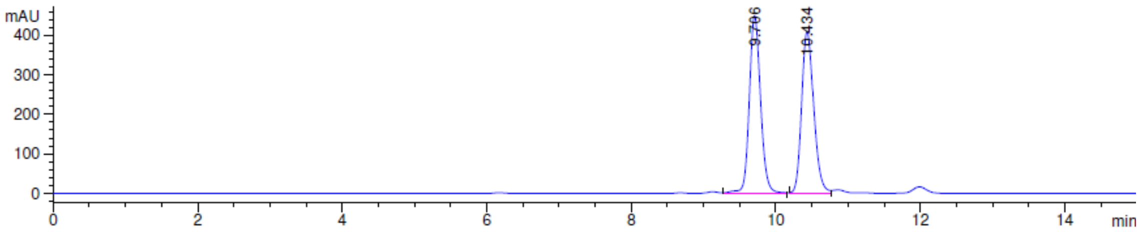

Peak #	RetTime [min]	Type	Width [min]	Area [mAU*s]	Height [mAU]	Area %
1	9.706	VB	0.1675	4915.22217	449.64163	50.7556
2	10.434	BV	0.1807	4768.88525	409.90628	49.2444
Totals :				9684.10742	859.54791	

Enantioenriched sample:

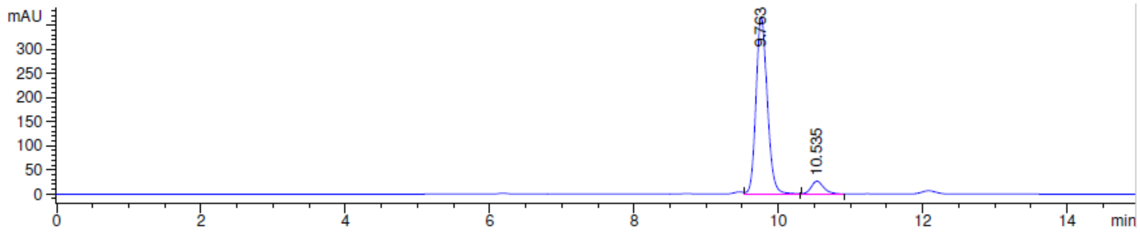

Peak #	RetTime [min]	Type	Width [min]	Area [mAU*s]	Height [mAU]	Area %
1	9.763	VB	0.1665	3949.47388	367.09430	92.7272
2	10.535	BB	0.1806	309.76639	26.27183	7.2728
Totals :				4259.24026	393.36613	

7. X-Ray Analysis

X-Ray diffraction: For compound **2n** data sets were collected with a Bruker APEX II CCD diffractometer. Data sets for compounds **2ab** were collected with a Bruker D8 Venture PHOTON III diffractometer. Programs used: data collection: APEX3 V2019.1-0[13] (Bruker AXS Inc., **2019**); cell refinement: SAINT V8.40A (Bruker AXS Inc., **2019**); data reduction: SAINT V8.40A (Bruker AXS Inc., **2019**); absorption correction, SADABS V2016/2 (Bruker AXS Inc., **2019**); structure solution *SHELXT-2015*[14] (Sheldrick, G. M. *Acta Cryst.,* **2015**, *A71*, 3-8); structure refinement *SHELXL-2015*[15] (Sheldrick, G. M. *Acta Cryst.,* **2015**, *C71* (1), 3-8) and graphics, *XP*[16] (Version 5.1, Bruker AXS Inc., Madison, Wisconsin, USA, **1998**). *R*-values are given for observed reflections, and wR^2 values are given for all reflections.

X-ray crystal structure analysis of 2n: A colorless plate-like specimen of $C_{13}H_{24}ClNO_2$, approximate dimensions 0.030 mm x 0.180 mm x 0.360 mm, was used for the X-ray crystallographic analysis. The X-ray intensity data were measured on a Bruker APEX II diffractometer system equipped with a fine-focus sealed tube Cu sealed tube (CuK_α, λ = 1.54178 Å) and a graphite monochromator. A total of 1342 frames were collected. The total exposure time was 21.11 hours. The frames were integrated with the Bruker SAINT software package using a wide-frame algorithm. The integration of the data using a monoclinic unit cell yielded a total of 7619 reflections to a maximum θ angle of 66.55° (0.84 Å resolution), of which 2387 were independent (average redundancy 3.192, completeness = 97.9%, R_{int} = 6.16%, R_{sig} = 6.56%) and 2146 (89.90%) were greater than $2\sigma(F^2)$. The final cell constants of a = 5.7308(3) Å, b = 11.5043(6) Å, c = 10.5714(6) Å, β = 91.202(3)°, volume = 696.81(6) $Å^3$, are based upon the refinement of the XYZ-centroids of 3262 reflections above 20 σ(I) with 8.365° < 2θ < 132.7°. Data were corrected for absorption effects using the Multi-Scan method (SADABS). The ratio of minimum to maximum apparent transmission was 0.791. The calculated minimum and maximum transmission coefficients (based on crystal size) are 0.4840 and 0.9330. The structure was solved and refined using the Bruker SHELXTL Software Package, using the space group $P2_1$, with Z = 2 for the formula unit, $C_{13}H_{24}ClNO_2$. The final anisotropic full-matrix least-squares refinement on F^2 with 159 variables converged at R1 = 3.76%, for the observed data and wR2 = 8.77% for all data. The goodness-of-fit was 1.036. The largest peak in the final difference electron density synthesis was 0.222 $e^-/Å^3$ and the largest hole was -0.232 $e^-/Å^3$ with an RMS deviation of 0.046 $e^-/Å^3$. On the basis of the final model, the calculated density was 1.248 g/cm^3 and F(000), 284 e^-. Flack parameter was refined to 0.035(14). The hydrogen at N1 atom was refined freely. CCDC Nr.: 2071064.

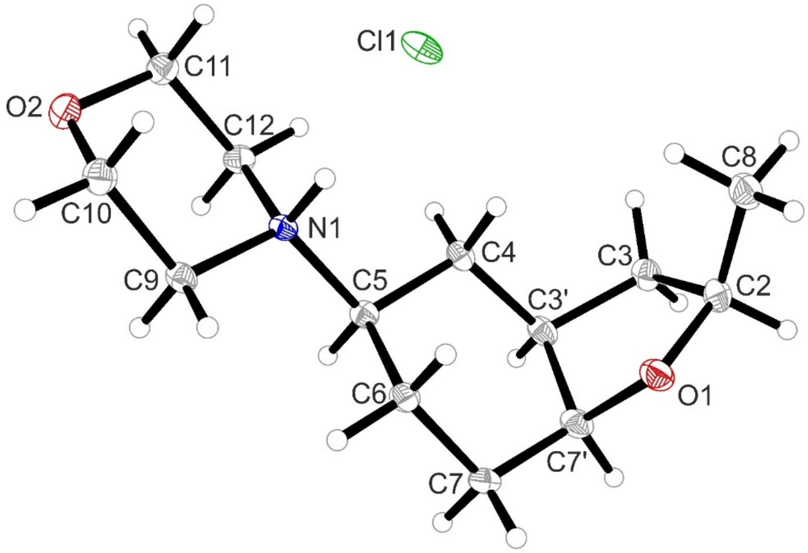

Figure S2: Crystal structure of compound **2n**. Thermal ellipsoids are shown at 30% probability.

X-ray crystal structure analysis of 2ab: A colorless plate-like specimen of $C_9H_{13}F_3O$, approximate dimensions 0.081 mm x 0.099 mm x 0.191 mm, was used for the X-ray crystallographic analysis. The X-ray intensity data were measured on a Bruker D8 Venture PHOTON III Diffractometer system equipped with a micro focus tube Cu Ims (CuK_α, λ = 1.54178 Å) and a MX mirror monochromator. A total of 991 frames were collected. The total exposure time was 12.40 hours. The frames were integrated with the Bruker SAINT software package using a narrow-frame algorithm. The integration of the data using an orthorhombic unit cell yielded a total of 7125 reflections to a maximum θ angle of 68.17° (0.83 Å resolution), of which 1596 were independent (average redundancy 4.464, completeness = 96.8%, R_{int} = 9.74%, R_{sig} = 4.62%) and 1584 (99.25%) were greater than $2\sigma(F^2)$. The final cell constants of a = 9.2242(2) Å, b = 9.2984(2) Å, c = 10.4901(2) Å, volume = 899.74(3) $Å^3$, are based upon the refinement of the XYZ-centroids of 6384 reflections above 20 σ(I) with 9.588° < 2θ < 136.3°. The calculated minimum and maximum transmission coefficients (based on crystal size) are 0.8080 and 0.9120.

The structure was solved and refined using the Bruker SHELXTL Software Package, using the space group $P2_12_12_1$, with Z = 4 for the formula unit, $C_9H_{13}F_3O$. The final anisotropic full-matrix least-squares refinement on F^2 with 119 variables converged at R1 = 2.93%, for the observed data and wR2 = 8.06% for all data. The goodness-of-fit was 1.114. The largest peak in the final difference electron density synthesis was 0.240 $e^-/Å^3$ and the largest hole was -0.170 $e^-/Å^3$ with an RMS deviation of 0.043 $e^-/Å^3$. On the basis of the final model, the calculated density was 1.434 g/cm^3 and F(000), 408 e^-. Flack parameter was refined to 0.03(5). CCDC Nr.: 2071065.

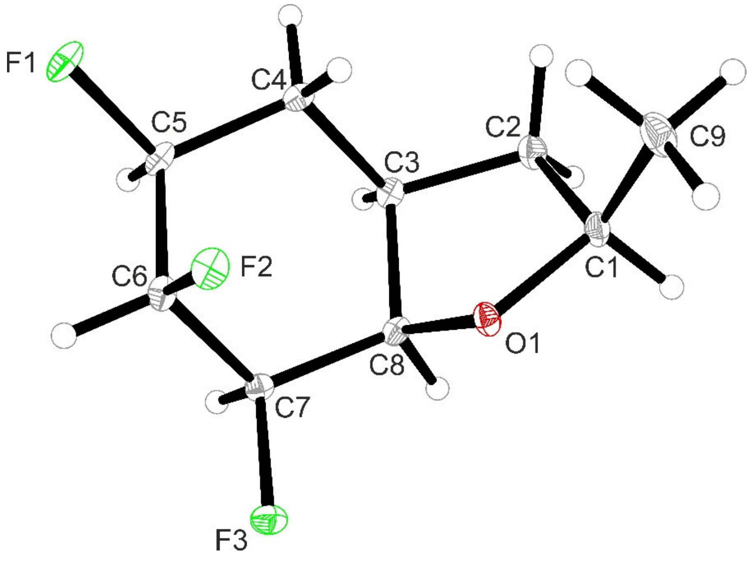

Figure S3: Crystal structure of compound **2ab**. Thermal ellipsoids are shown at 30% probability.

8. Literature

[1] M. P. Wiesenfeldt, D. Moock, D. Paul, F. Glorius, *Chem. Sci.* **2021**, DOI 10.1039/D0SC07099H.

[2] M. P. Wiesenfeldt, Z. Nairoukh, W. Li, F. Glorius, *Science* **2017**, *357*, 908–912.

[3] P. Barker, P. Finke, K. Thompson, *Synth. Commun.* **1989**, *19*, 257–265.

[4] N. Ortega, S. Urban, B. Beiring, F. Glorius, *Angew. Chem. Int. Ed.* **2012**, *51*, 1710–1713; *Angew. Chem.* **2012**, *124*, 1742–1745.

[5] W.-G. Lee, R. Gallardo-Macias, K. M. Frey, K. A. Spasov, M. Bollini, K. S. Anderson, W. L. Jorgensen, *J. Am. Chem. Soc.* **2013**, *135*, 16705–16713.

[6] F. Ye, C. Wang, X. Ma, M. L. Hossain, Y. Xia, Y. Zhang, J. Wang, *J. Org. Chem.* **2015**, *80*, 647–652.

[7] R. Fu, Z. Li, *Org. Lett.* **2018**, *20*, 2342–2345.

[8] H. Brandenburg, J. Krahmer, K. Fischer, B. Schwager, B. Flöser, C. Näther, F. Tuczek, *Eur. J. Inorg. Chem.* **2018**, *2018*, 576–585.

[9] S. Kaiser, S. P. Smidt, A. Pfaltz, *Angew. Chem. Int. Ed.* **2006**, *45*, 5194–5197; *Angew. Chem.* **2006**, *118*, 5318–5321.

[10] S. Nielek, T. Lesiak, *Chem. Ber.* **1982**, *115*, 1247–1251.

[11] L. Pitzer, F. Schäfers, F. Glorius, *Angew. Chem. Int. Ed.* **2019**, *58*, 8572–8576; *Angew. Chem.* **2019**, *131*, 8660–8664.

[12] D. Paul, B. Beiring, M. Plois, N. Ortega, S. Kock, D. Schlüns, J. Neugebauer, R. Wolf, F. Glorius, *Organometallics* **2016**, *35*, 3641–3646.

[13] Bruker AXS (**2019**) *APEX3 Version 2019.1-0, SAINT Version 8.40A* and *SADABS Bruker AXS area detector scaling and absorption correction Version 2016/2*, Bruker AXS Inc., Madison, Wisconsin, USA.

[14] Sheldrick, G. M. SHELXT – *Integrated space-group and crystal-structure determination*. *Acta. Cryst.* **2015**, *A71*, 3–8.

[15] Sheldrick, G. M. *Crystal structure refinement with SHELXL. Acta. Cryst.* **2015**, *C71*, 3–8.

[17] Bruker AXS (**1998**) XP – *Interactive molecular graphics, Version 5.1*, Bruker AXS Inc., Madison, Wisconsin, USA, **1998**.

9. NMR Spectra

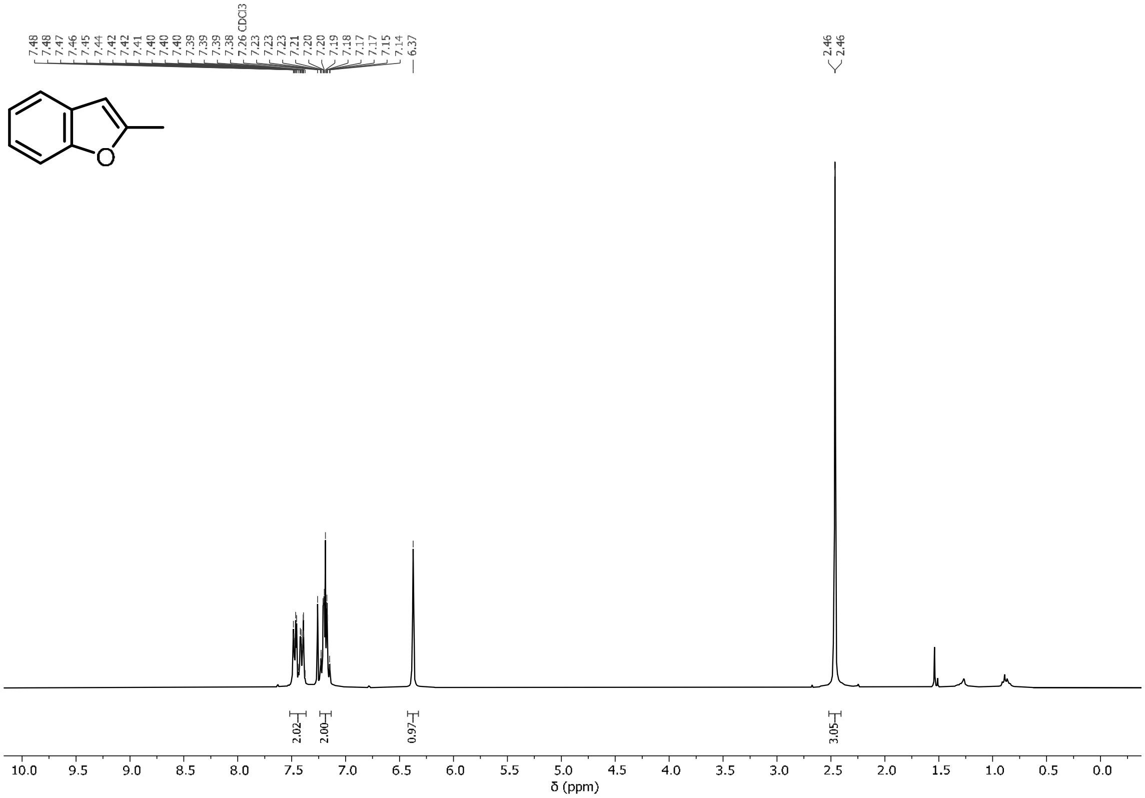

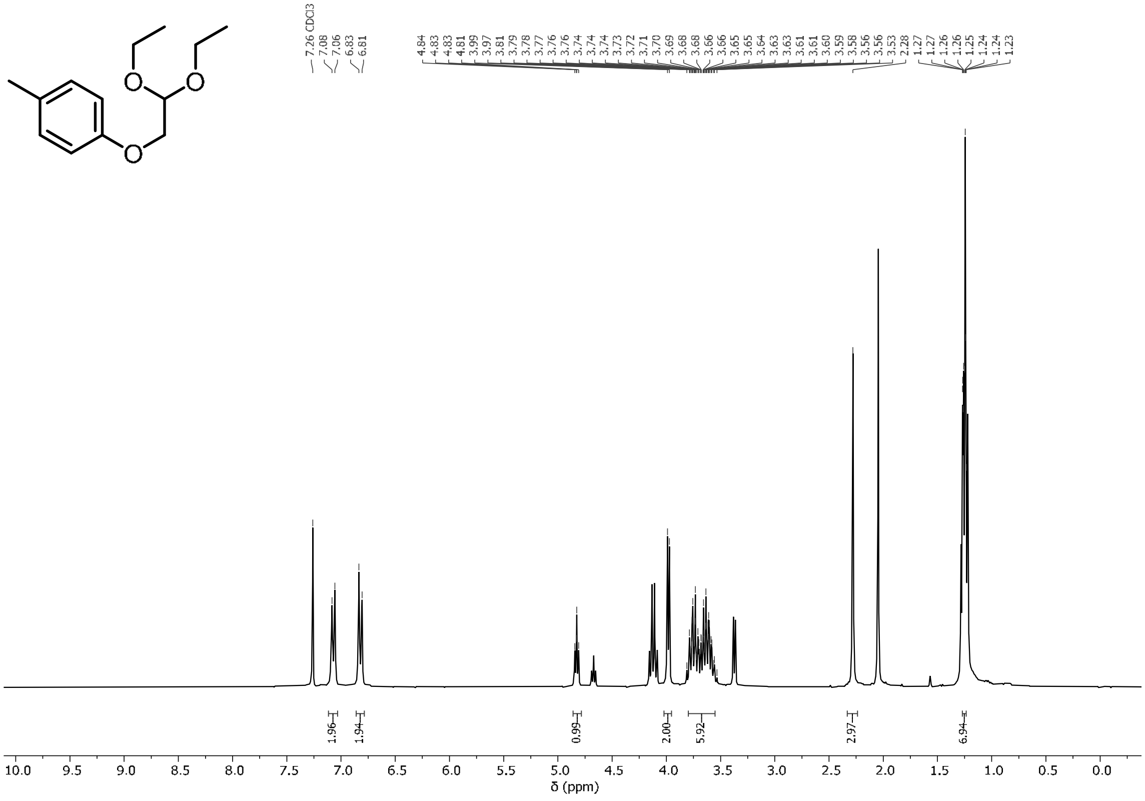

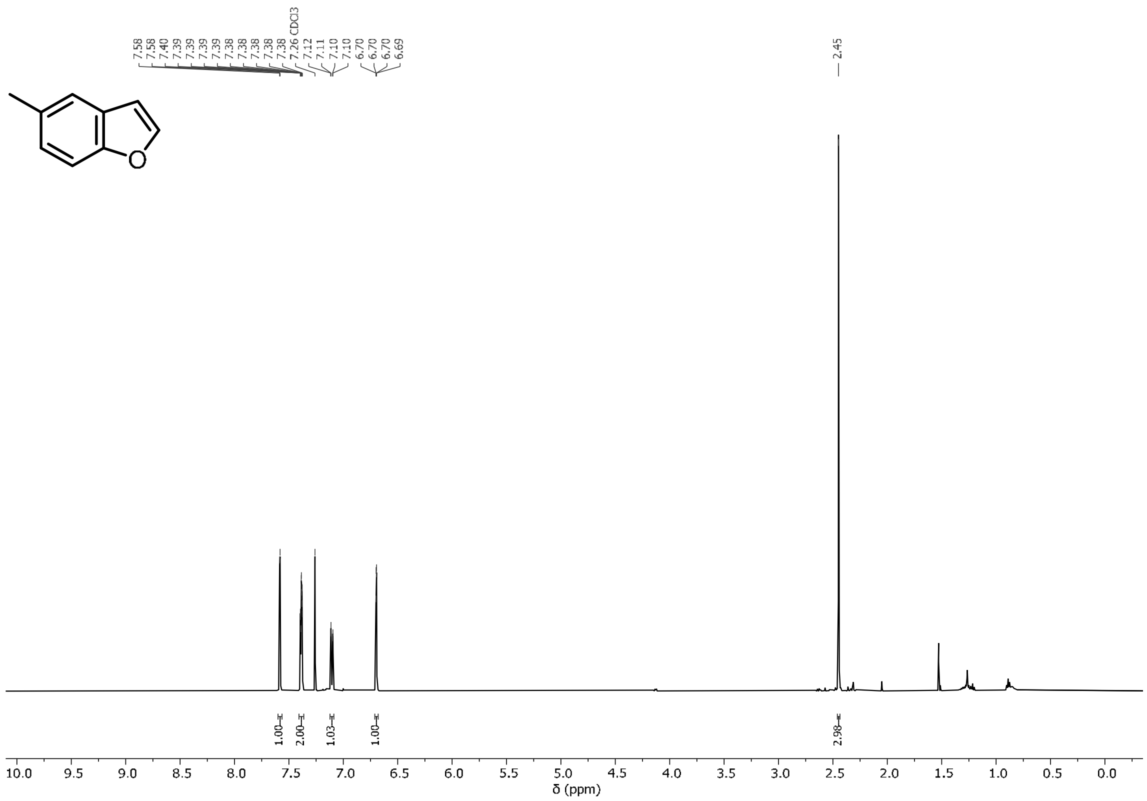
7.58
7.58
7.40
7.39
7.39
7.39
7.39
7.38
7.38
7.38
7.38
7.38
7.26 CDCl3
7.12
7.11
7.10
7.10
6.70
6.70
6.70
6.69
2.45
1.00
2.00
1.03
1.00
2.98
δ (ppm)

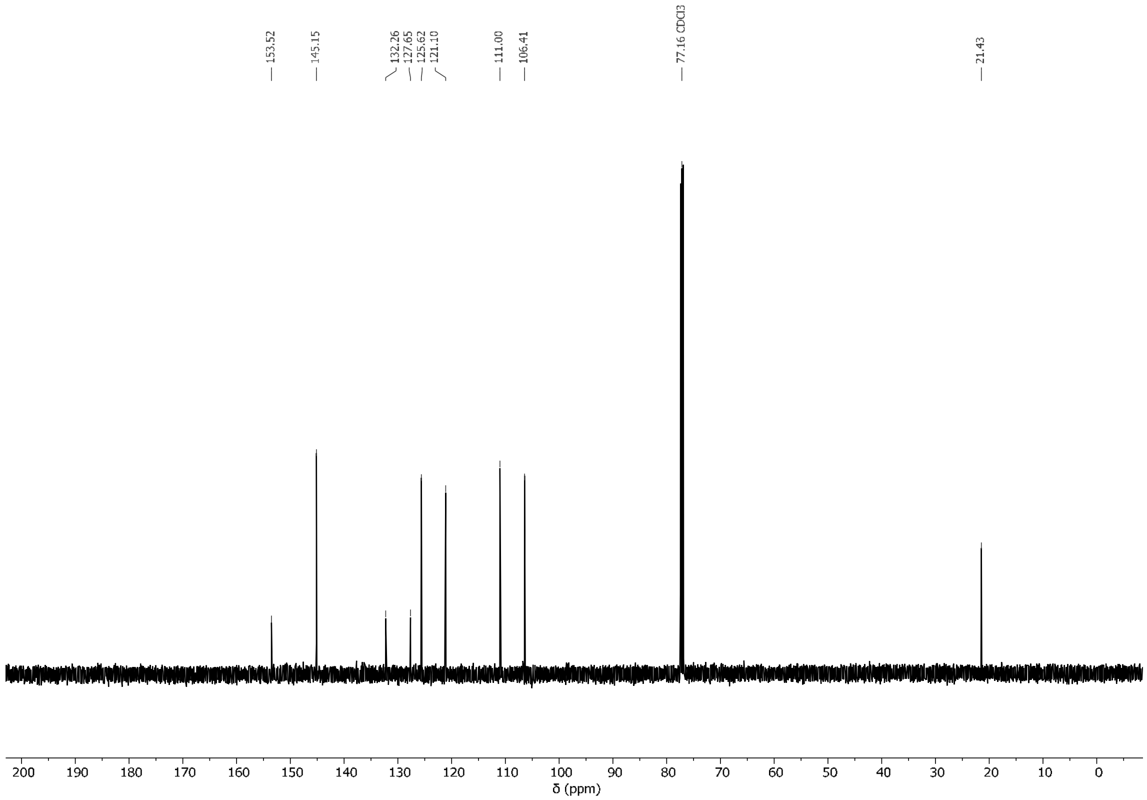
153.52
145.15
132.26
127.65
125.62
121.10
111.00
106.41
77.16 CDCl3
21.43
δ (ppm)

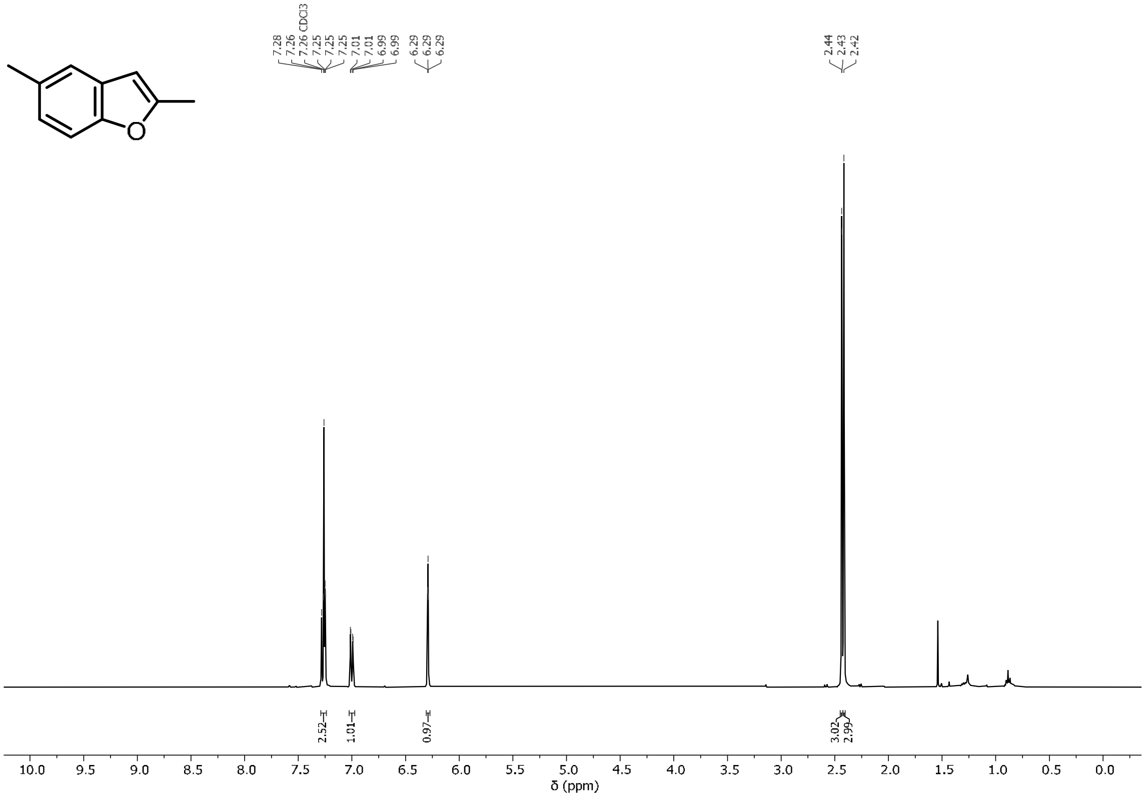
7.28
7.26
7.26 CDCl3
7.25
7.25
7.25
7.01
7.01
6.99
6.99
6.29
6.29
6.29
2.44
2.43
2.42
O
2.52
1.01
0.97
3.02
2.99
10.0 9.5 9.0 8.5 8.0 7.5 7.0 6.5 6.0 5.5 5.0 4.5 4.0 3.5 3.0 2.5 2.0 1.5 1.0 0.5 0.0
δ (ppm)

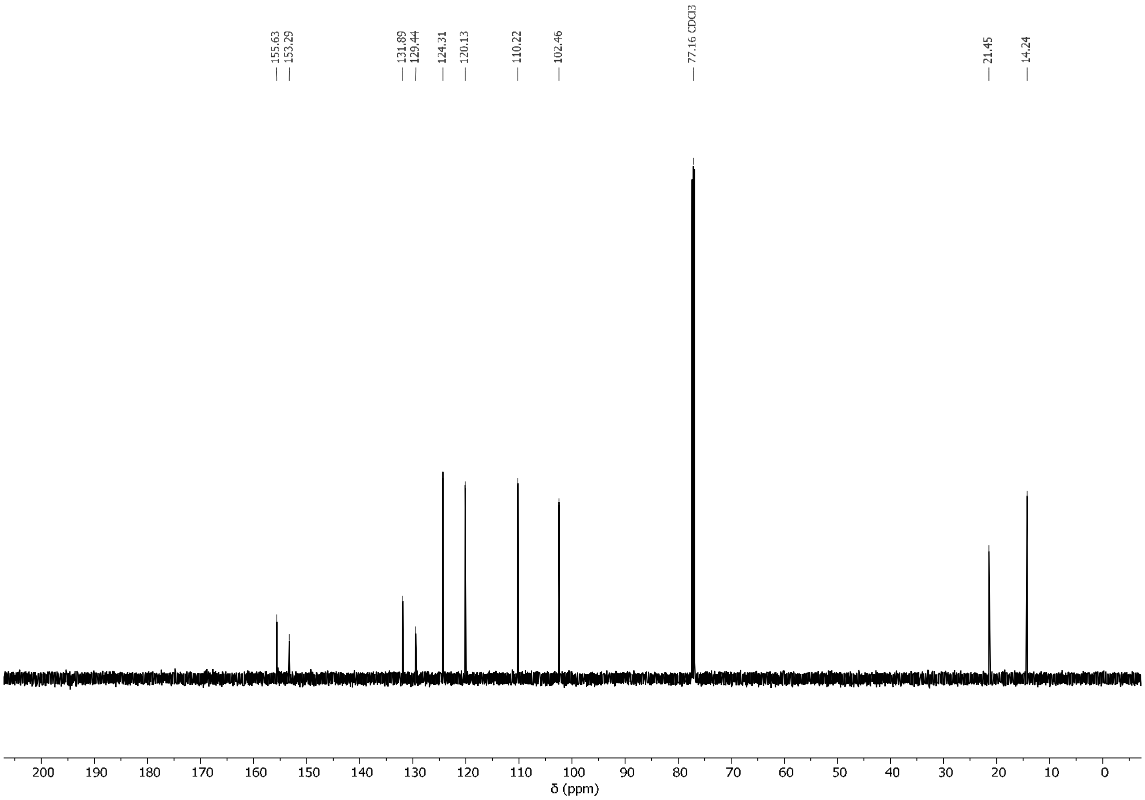
155.63
153.29
131.89
129.44
124.31
120.13
110.22
102.46
77.16 CDCl3
21.45
14.24
200 190 180 170 160 150 140 130 120 110 100 90 80 70 60 50 40 30 20 10 0
δ (ppm)

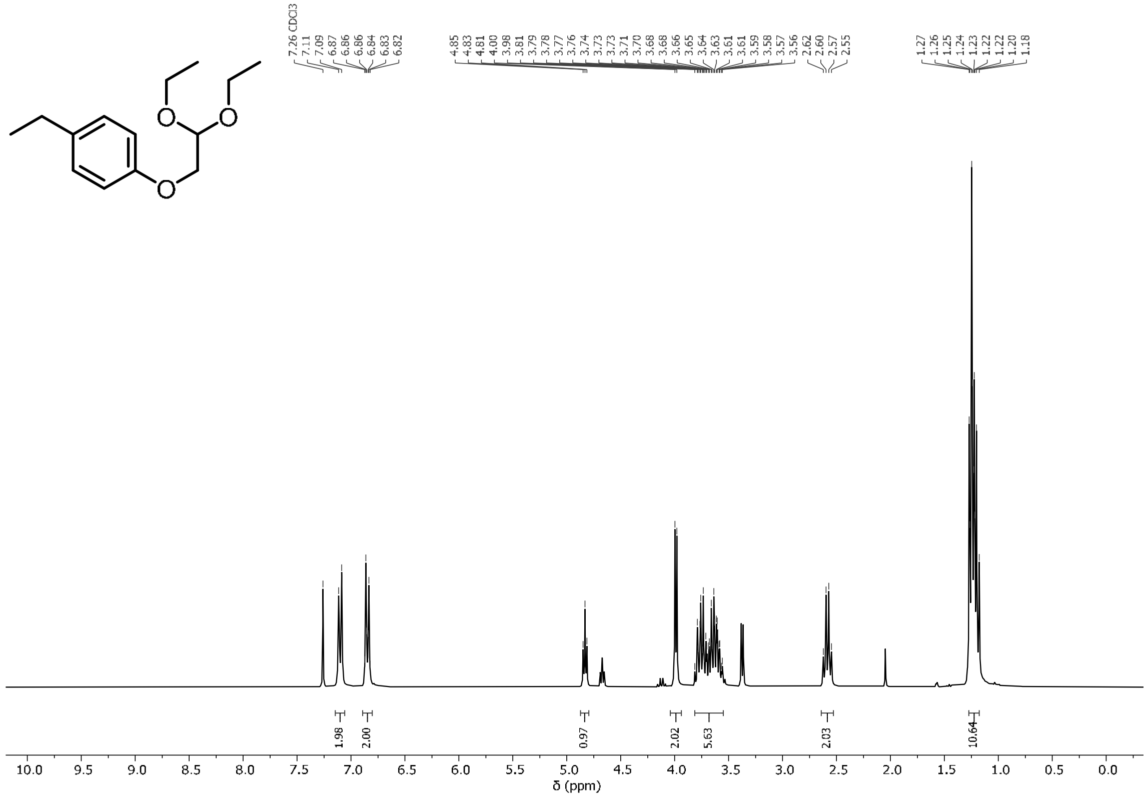

7.26 CDCl3
7.11
7.09
6.87
6.86
6.86
6.84
6.83
6.82
4.85
4.83
4.81
4.00
3.98
3.81
3.79
3.78
3.77
3.76
3.74
3.73
3.73
3.71
3.70
3.68
3.68
3.66
3.65
3.64
3.63
3.61
3.61
3.59
3.58
3.57
3.56
2.62
2.60
2.57
2.55
1.27
1.26
1.25
1.24
1.23
1.22
1.22
1.20
1.18
1.98
2.00
0.97
2.02
5.63
2.03
10.64
δ (ppm)

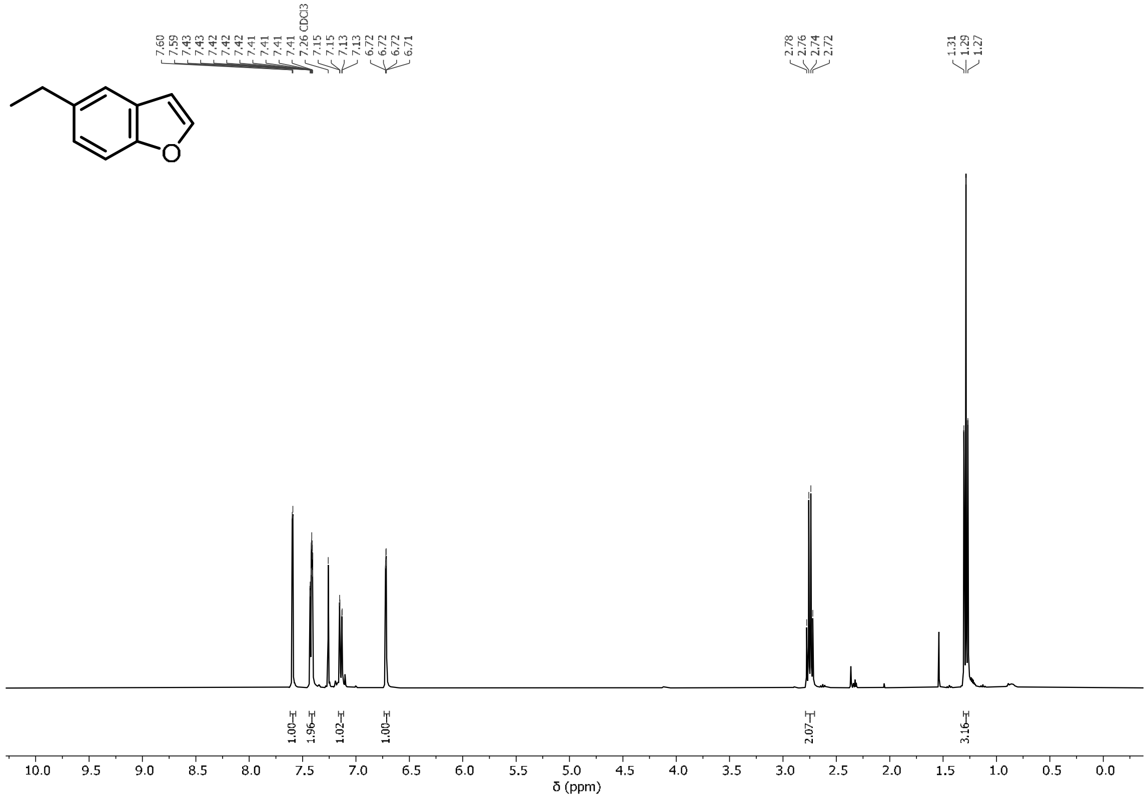

7.60
7.59
7.43
7.43
7.42
7.42
7.42
7.41
7.41
7.41
7.41
7.26 CDCl3
7.15
7.15
7.13
7.13
6.72
6.72
6.72
6.71
2.78
2.76
2.74
2.72
1.31
1.29
1.27
1.00
1.96
1.02
1.00
2.07
3.16
δ (ppm)

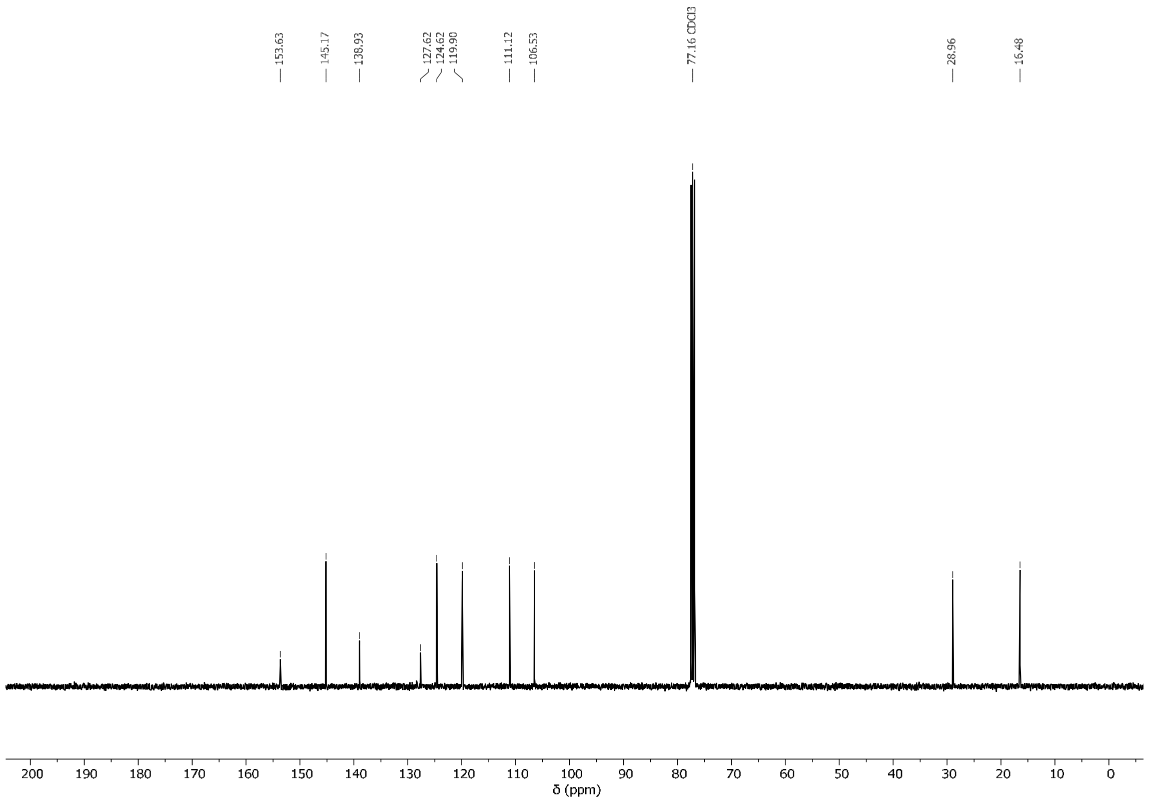
153.63
145.17
138.93
127.62
124.62
119.90
111.12
106.53
77.16 CDCl3
28.96
16.48
200 190 180 170 160 150 140 130 120 110 100 90 80 70 60 50 40 30 20 10 0
δ (ppm)

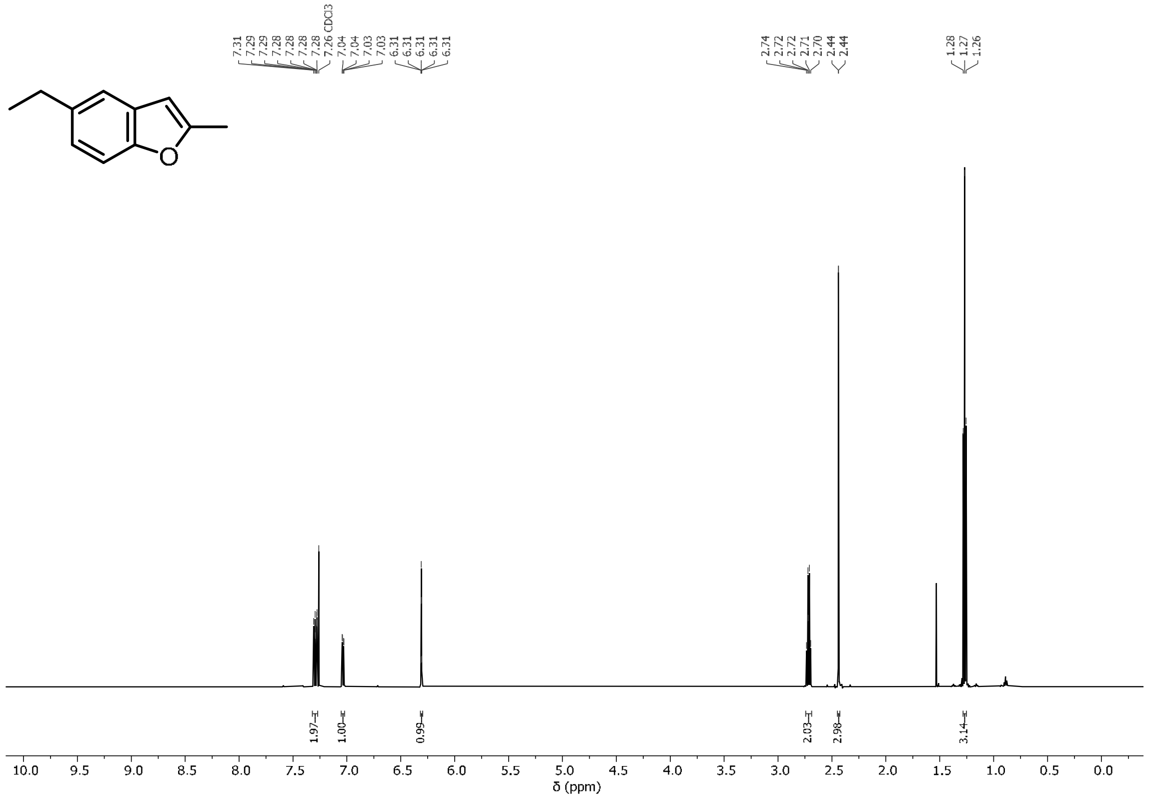
7.31
7.29
7.29
7.28
7.28
7.28
7.28
7.26 CDCl3
7.04
7.04
7.03
7.03
6.31
6.31
6.31
6.31
6.31
2.74
2.72
2.72
2.71
2.70
2.44
2.44
1.28
1.27
1.26
O
1.97
1.00
0.99
2.03
2.98
3.14
10.0 9.5 9.0 8.5 8.0 7.5 7.0 6.5 6.0 5.5 5.0 4.5 4.0 3.5 3.0 2.5 2.0 1.5 1.0 0.5 0.0
δ (ppm)

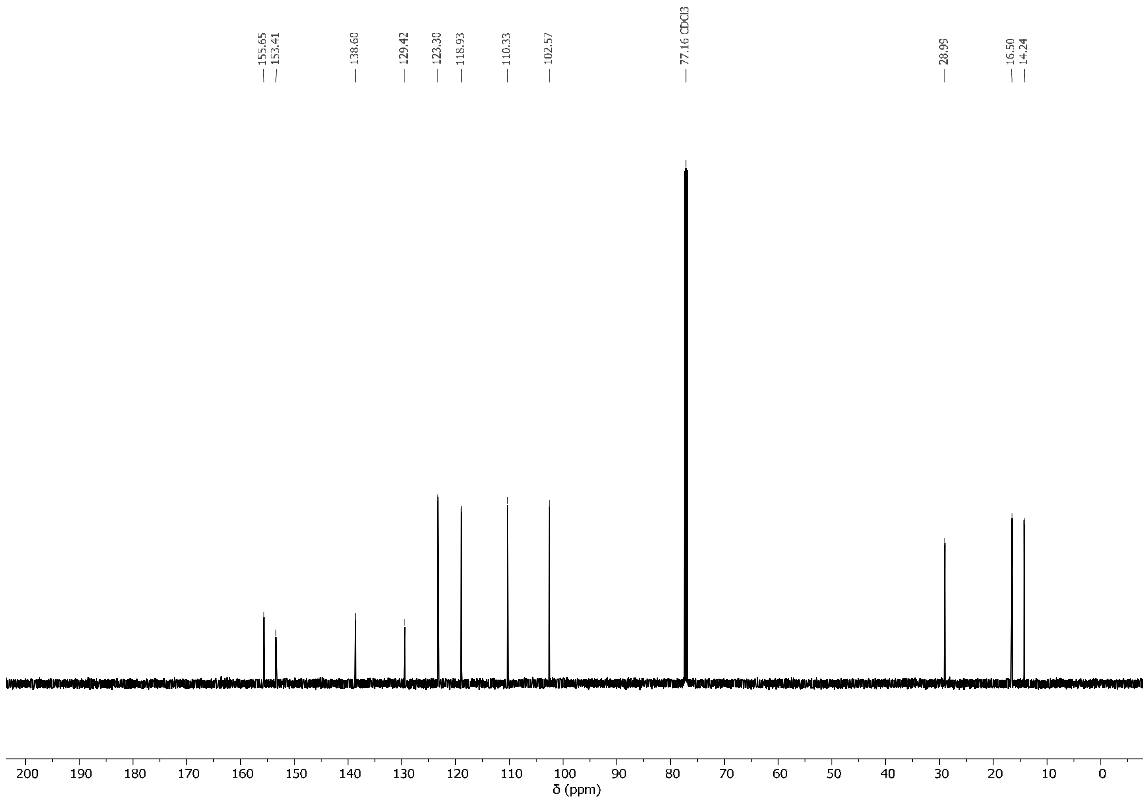
155.65
153.41
138.60
129.42
123.30
118.93
110.33
102.57
77.16 CDCl3
28.99
16.50
14.24
200 190 180 170 160 150 140 130 120 110 100 90 80 70 60 50 40 30 20 10 0
δ (ppm)

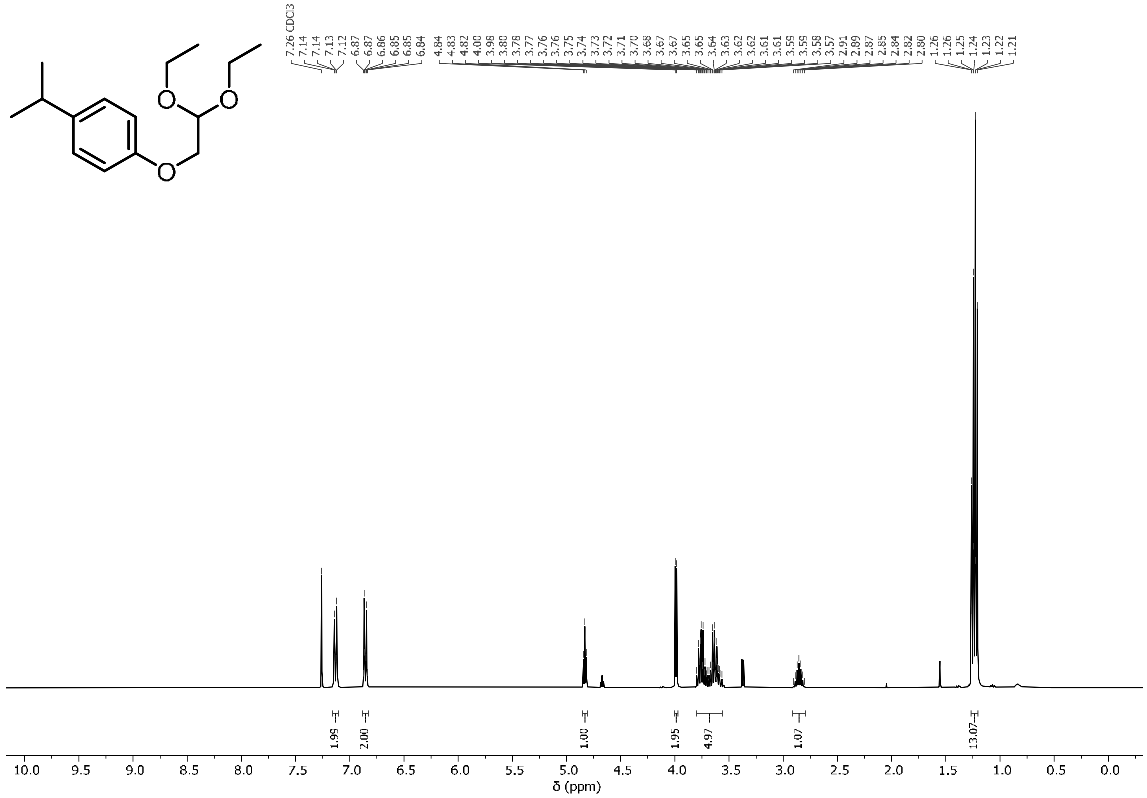
7.26 CDCl3
7.14
7.14
7.13
7.12
6.87
6.87
6.86
6.85
6.85
6.84
4.84
4.83
4.82
4.00
3.98
3.80
3.78
3.77
3.76
3.76
3.75
3.74
3.73
3.72
3.71
3.70
3.68
3.67
3.67
3.65
3.65
3.64
3.63
3.62
3.62
3.61
3.61
3.59
3.59
3.58
3.57
2.91
2.89
2.87
2.87
2.85
2.84
2.82
2.80
1.26
1.26
1.25
1.24
1.23
1.22
1.21
1.99
2.00
1.00
1.95
4.97
1.07
13.07
10.0 9.5 9.0 8.5 8.0 7.5 7.0 6.5 6.0 5.5 5.0 4.5 4.0 3.5 3.0 2.5 2.0 1.5 1.0 0.5 0.0
δ (ppm)

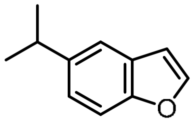

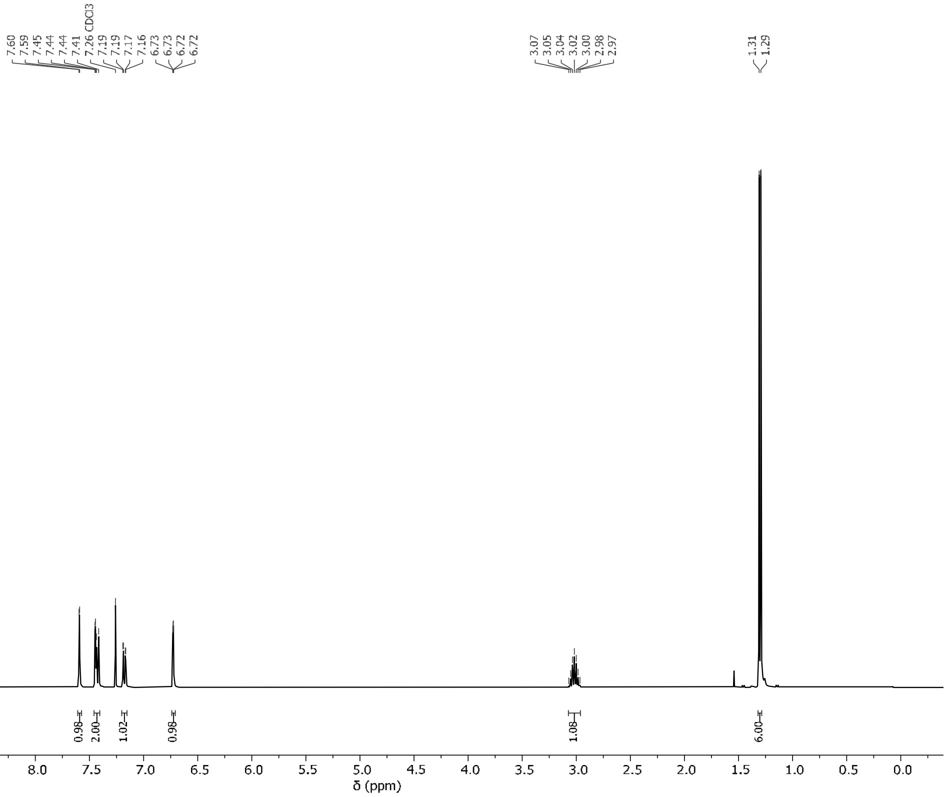

7.60
7.59
7.45
7.44
7.44
7.41
7.26 CDCl3
7.19
7.19
7.17
7.16
6.73
6.73
6.72
6.72
3.07
3.05
3.04
3.02
3.00
2.98
2.97
1.31
1.29
0.98
2.00
1.02
0.98
1.08
6.00
10.0 9.5 9.0 8.5 8.0 7.5 7.0 6.5 6.0 5.5 5.0 4.5 4.0 3.5 3.0 2.5 2.0 1.5 1.0 0.5 0.0
δ (ppm)

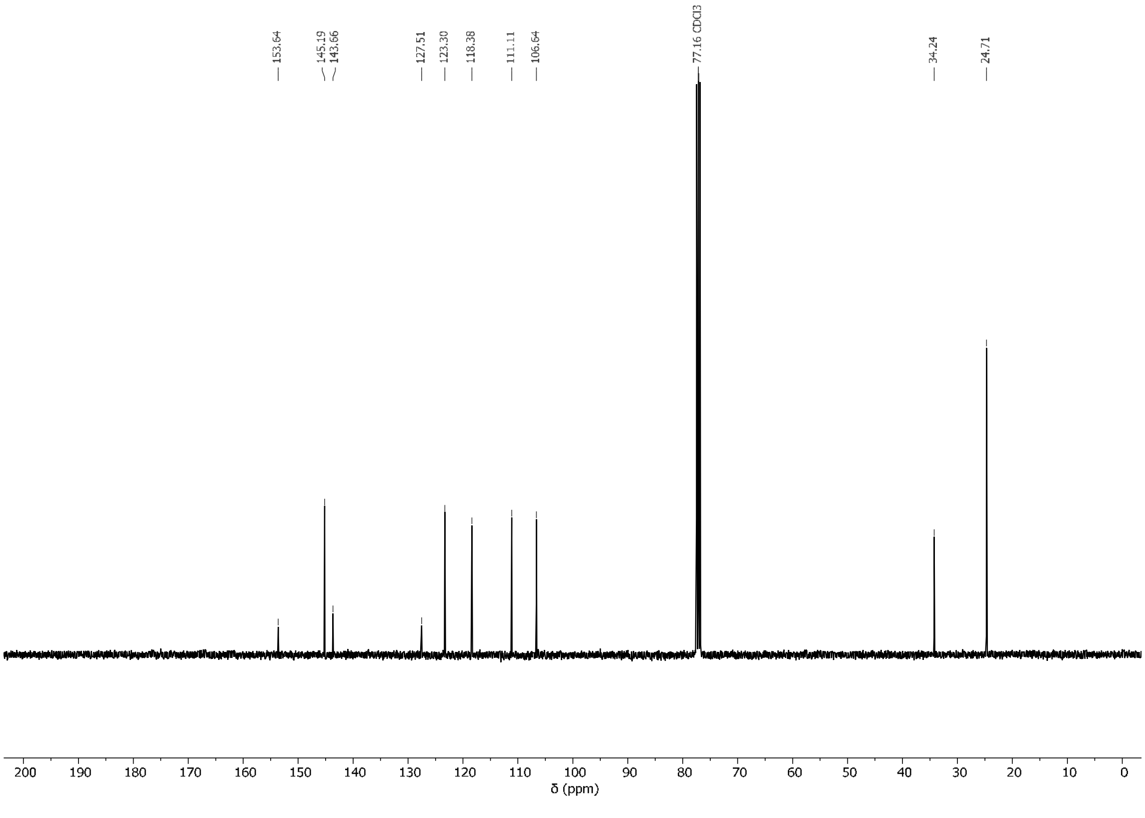

153.64
145.19
143.66
127.51
123.30
118.38
111.11
106.64
77.16 CDCl3
34.24
24.71
200 190 180 170 160 150 140 130 120 110 100 90 80 70 60 50 40 30 20 10 0
δ (ppm)

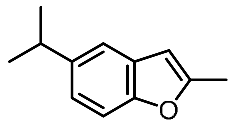

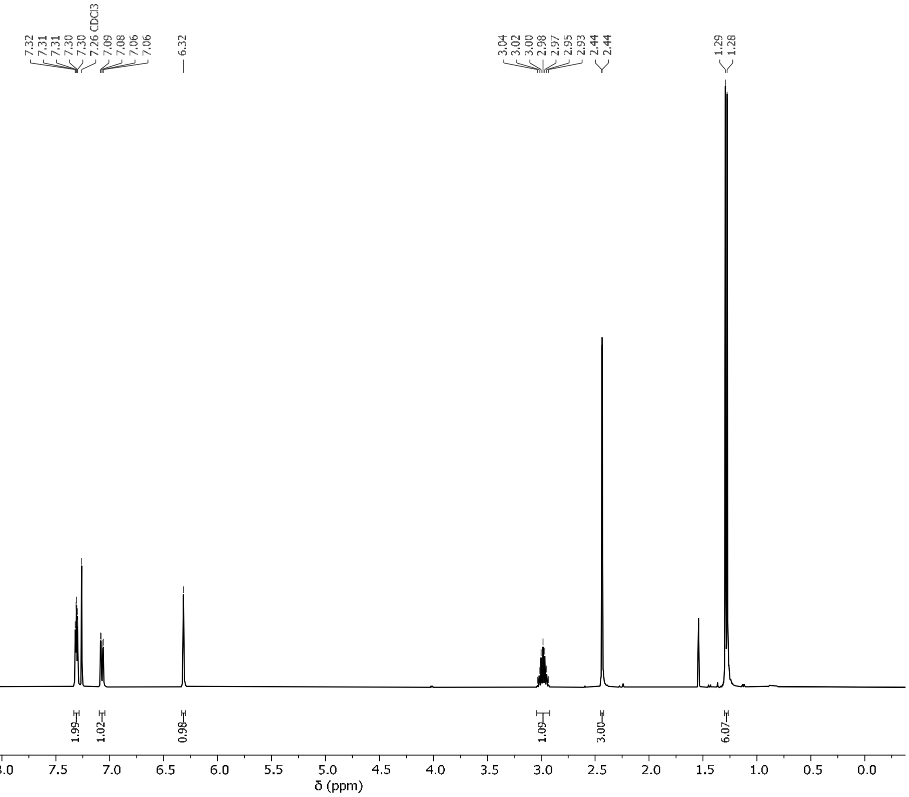
7.32
7.31
7.31
7.30
7.30
7.26 CDCl3
7.09
7.08
7.06
7.06
6.32
3.04
3.02
3.00
2.98
2.97
2.95
2.93
2.44
2.44
1.29
1.28
1.99
1.02
0.98
1.09
3.00
6.07
10.0 9.5 9.0 8.5 8.0 7.5 7.0 6.5 6.0 5.5 5.0 4.5 4.0 3.5 3.0 2.5 2.0 1.5 1.0 0.5 0.0
δ (ppm)

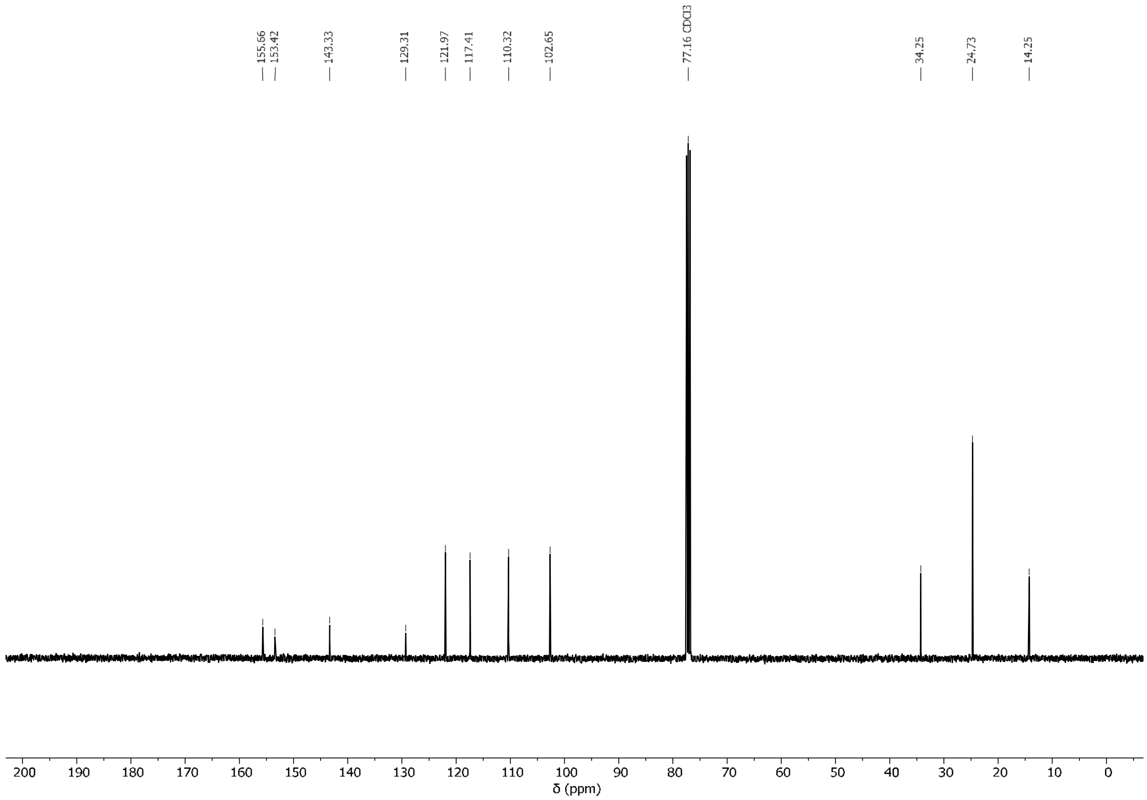
155.66
153.42
143.33
129.31
121.97
117.41
110.32
102.65
77.16 CDCl3
34.25
24.73
14.25
200 190 180 170 160 150 140 130 120 110 100 90 80 70 60 50 40 30 20 10 0
δ (ppm)

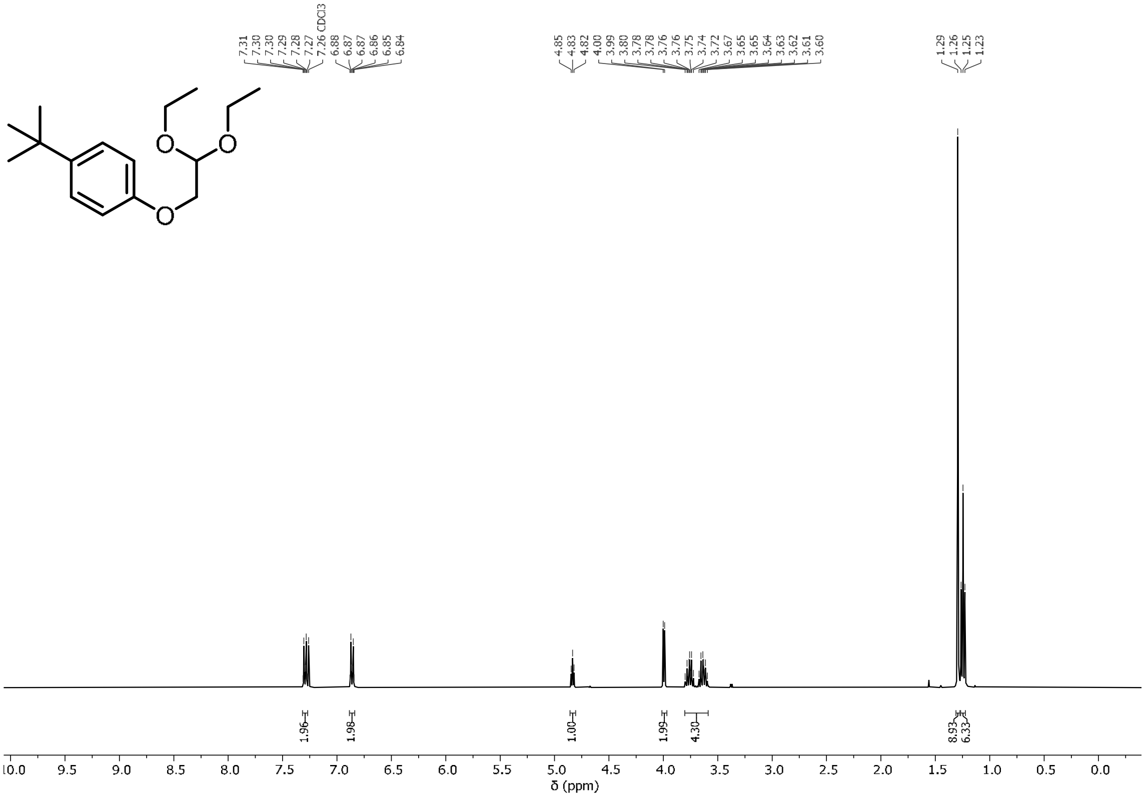
7.31 7.30 7.30 7.29 7.28 7.27 7.26 CDCl3 6.88 6.87 6.87 6.86 6.85 6.84
4.85 4.83 4.82 4.00 3.99 3.80 3.78 3.78 3.76 3.76 3.75 3.74 3.72 3.67 3.65 3.65 3.64 3.63 3.62 3.61 3.60
1.29 1.26 1.25 1.23
1.96 1.98 1.00 1.99 4.30 8.93 6.33
10.0 9.5 9.0 8.5 8.0 7.5 7.0 6.5 6.0 5.5 5.0 4.5 4.0 3.5 3.0 2.5 2.0 1.5 1.0 0.5 0.0
δ (ppm)

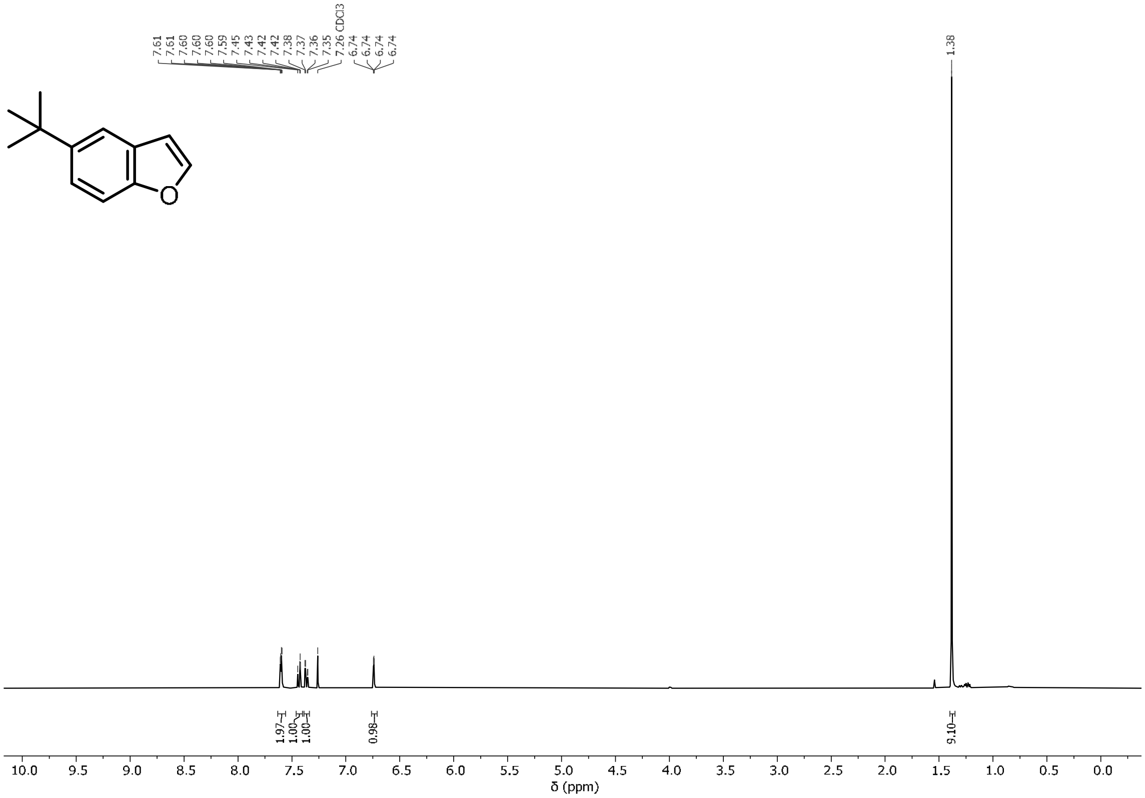
7.61 7.61 7.60 7.60 7.60 7.59 7.45 7.43 7.42 7.42 7.38 7.37 7.36 7.35 7.26 CDCl3 6.74 6.74 6.74 6.74
1.38
1.97 1.00 1.00 0.98 9.10
10.0 9.5 9.0 8.5 8.0 7.5 7.0 6.5 6.0 5.5 5.0 4.5 4.0 3.5 3.0 2.5 2.0 1.5 1.0 0.5 0.0
δ (ppm)

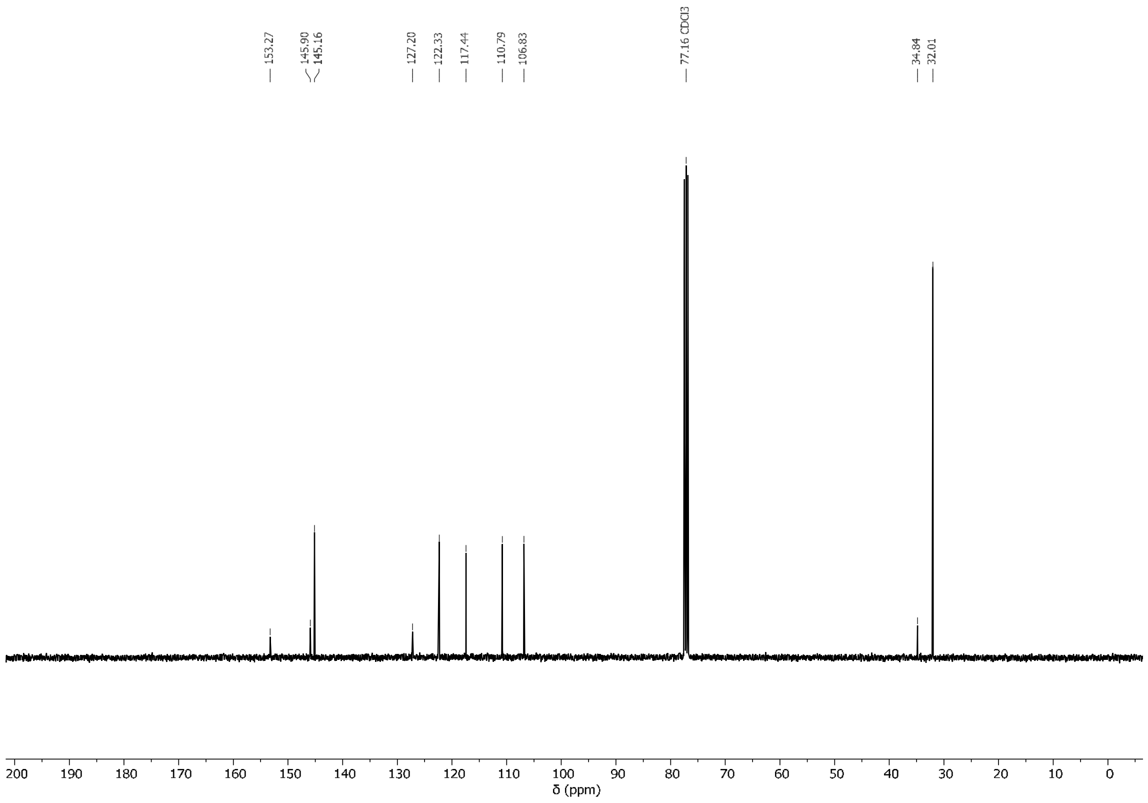
153.27
145.90
145.16
127.20
122.33
117.44
110.79
106.83
77.16 CDCl3
34.84
32.01
200 190 180 170 160 150 140 130 120 110 100 90 80 70 60 50 40 30 20 10 0
δ (ppm)

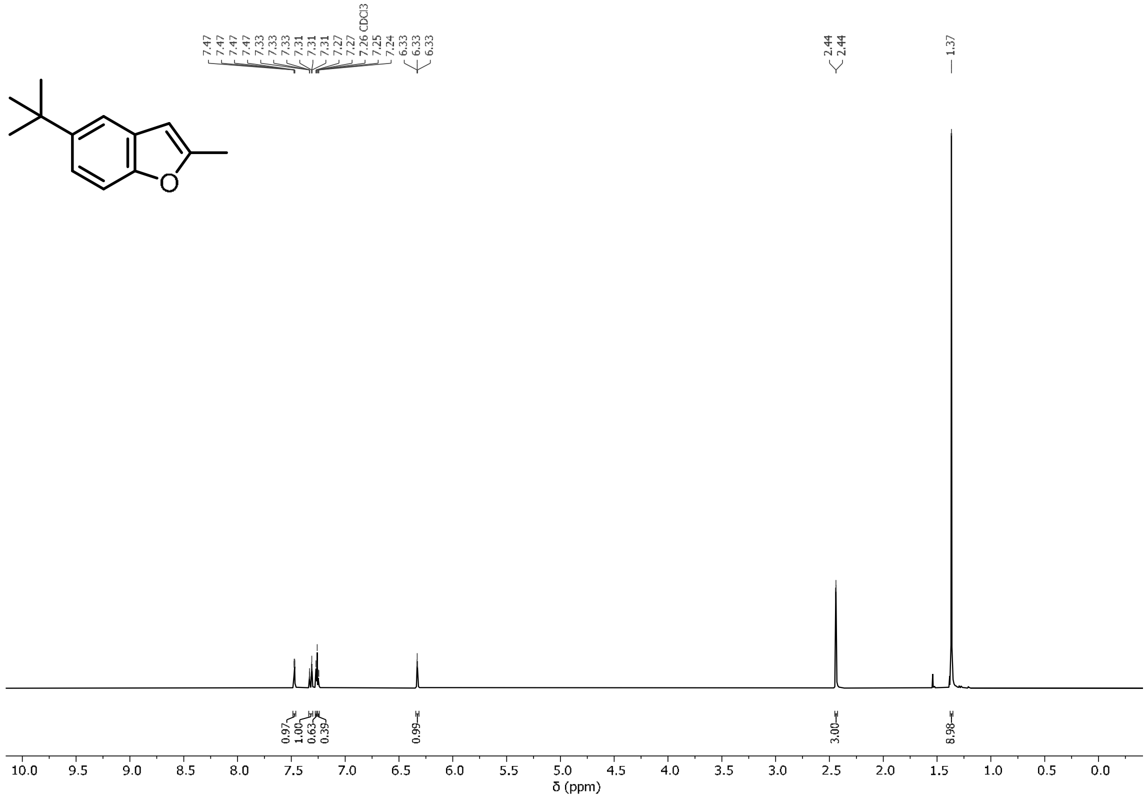
7.47
7.47
7.47
7.47
7.33
7.33
7.33
7.31
7.31
7.31
7.27
7.27
7.26 CDCl3
7.25
7.24
6.33
6.33
6.33
2.44
2.44
1.37
0.97
1.00
0.63
0.39
0.99
3.00
8.98
10.0 9.5 9.0 8.5 8.0 7.5 7.0 6.5 6.0 5.5 5.0 4.5 4.0 3.5 3.0 2.5 2.0 1.5 1.0 0.5 0.0
δ (ppm)

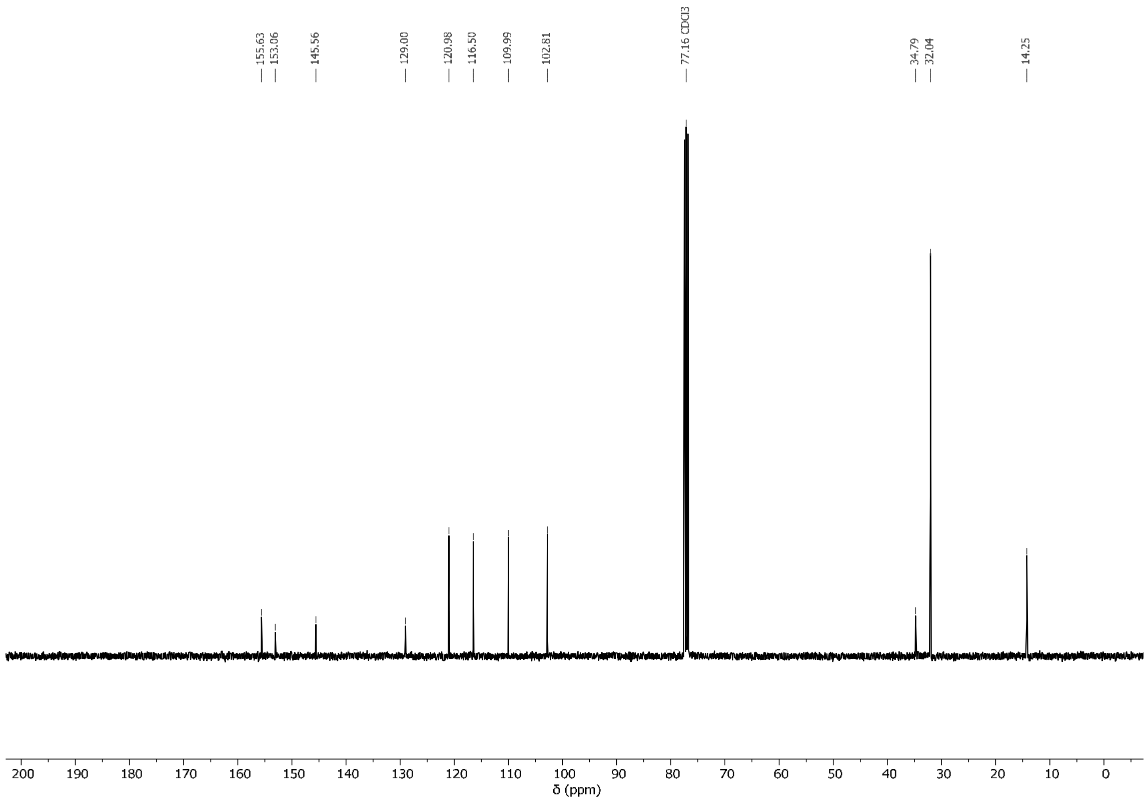
155.63
153.06
145.56
129.00
120.98
116.50
109.99
102.81
77.16 CDCl3
34.79
32.04
14.25
200 190 180 170 160 150 140 130 120 110 100 90 80 70 60 50 40 30 20 10 0
δ (ppm)

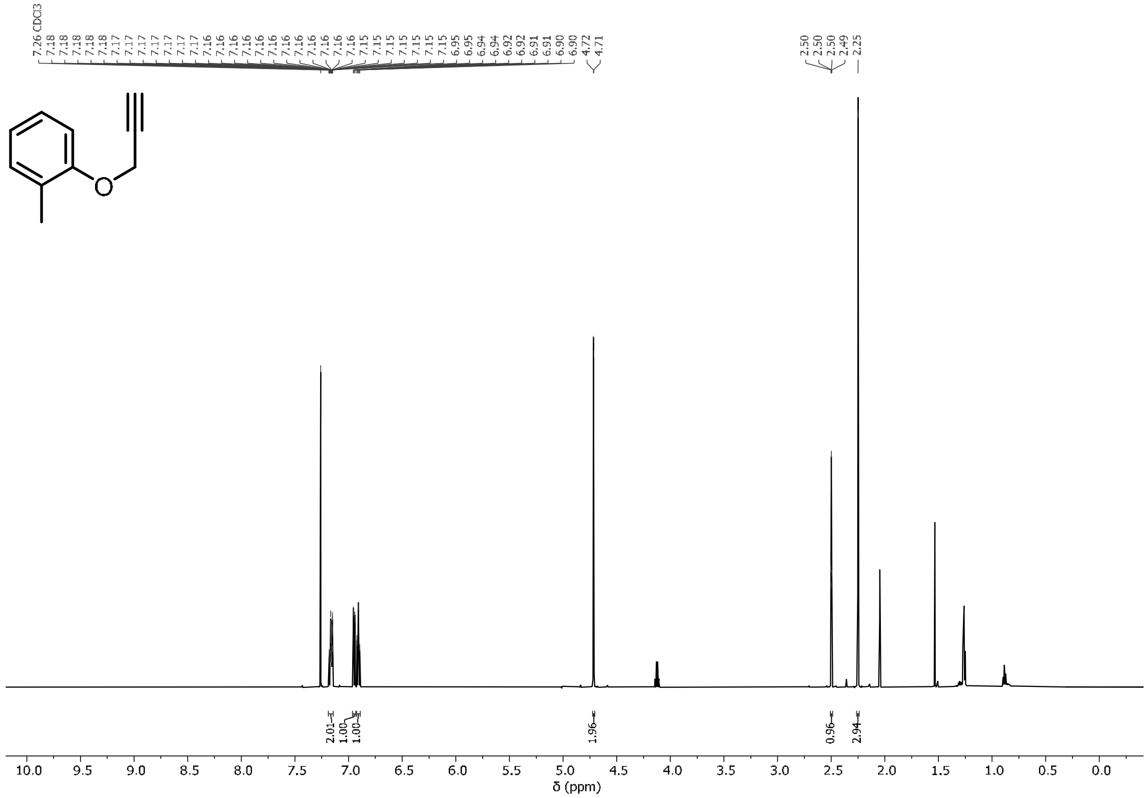
7.26 CDCl3
7.18 7.18 7.18 7.18 7.18 7.17 7.17 7.17 7.17 7.17 7.17 7.16 7.16 7.16 7.16 7.16 7.16 7.16 7.16 7.16 7.15 7.15 7.15 7.15 7.15 7.15 7.15 6.95 6.95 6.94 6.94 6.92 6.92 6.91 6.91 6.90 6.90
4.72
4.71
2.50
2.50
2.50
2.49
2.25
O
2.01
1.00
1.00
1.96
0.96
2.94
10.0 9.5 9.0 8.5 8.0 7.5 7.0 6.5 6.0 5.5 5.0 4.5 4.0 3.5 3.0 2.5 2.0 1.5 1.0 0.5 0.0
δ (ppm)

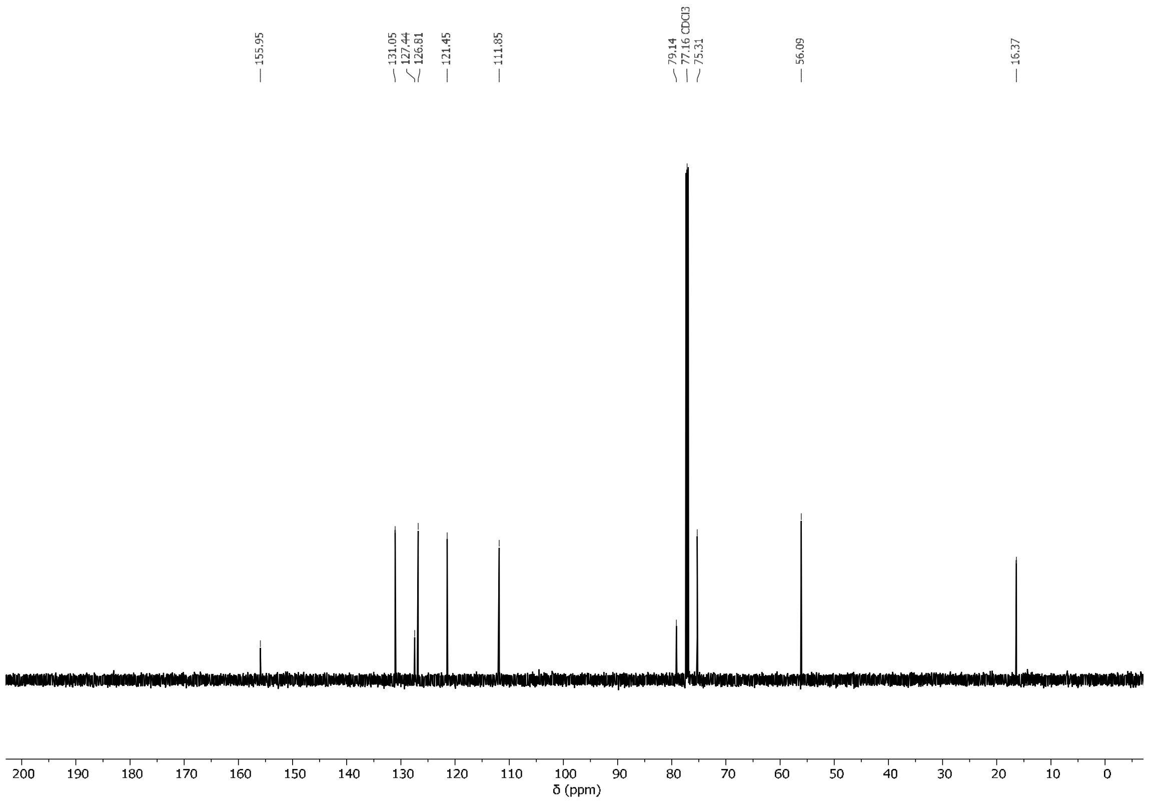
155.95
131.05
127.44
126.81
121.45
111.85
79.14
77.16 CDCl3
75.31
56.09
16.37
200 190 180 170 160 150 140 130 120 110 100 90 80 70 60 50 40 30 20 10 0
δ (ppm)

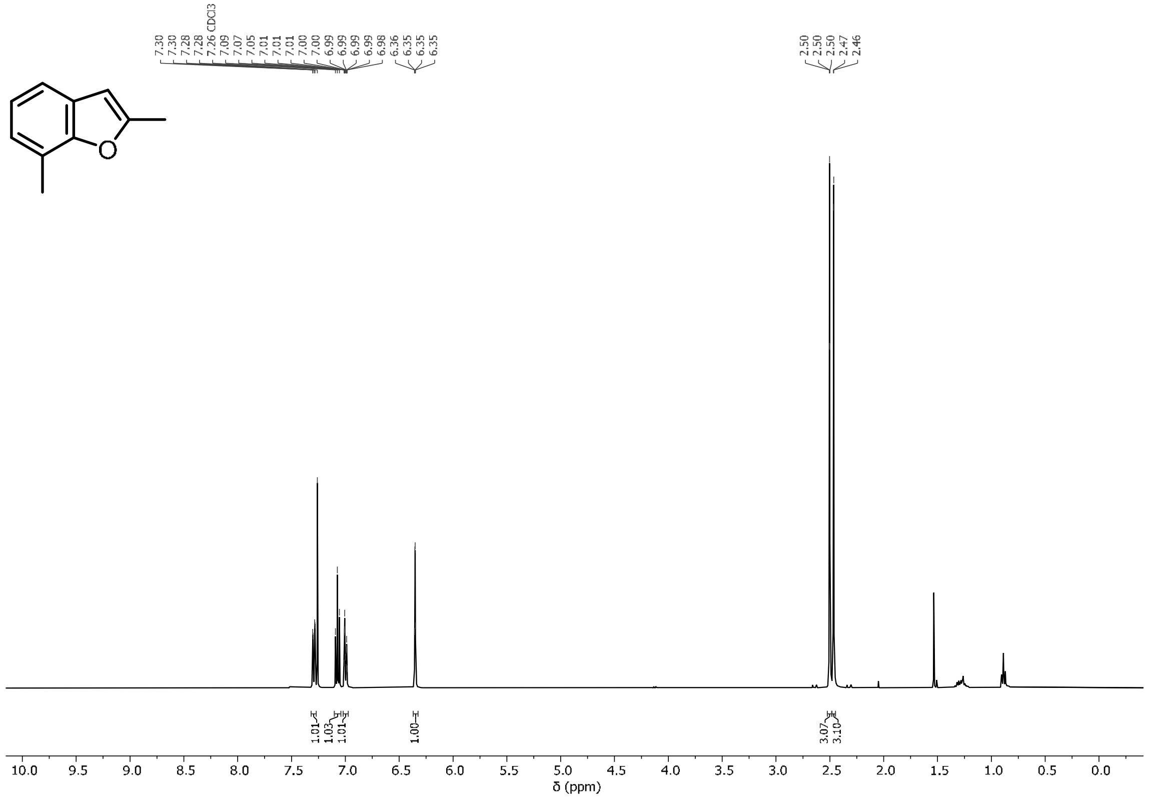
7.30
7.30
7.28
7.26 CDCl3
7.09
7.07
7.05
7.01
7.01
7.00
7.00
6.99
6.99
6.99
6.99
6.98
6.36
6.35
6.35
6.35
2.50
2.50
2.50
2.47
2.46
1.01
1.03
1.01
1.00
3.07
3.10
10.0 9.5 9.0 8.5 8.0 7.5 7.0 6.5 6.0 5.5 5.0 4.5 4.0 3.5 3.0 2.5 2.0 1.5 1.0 0.5 0.0
δ (ppm)

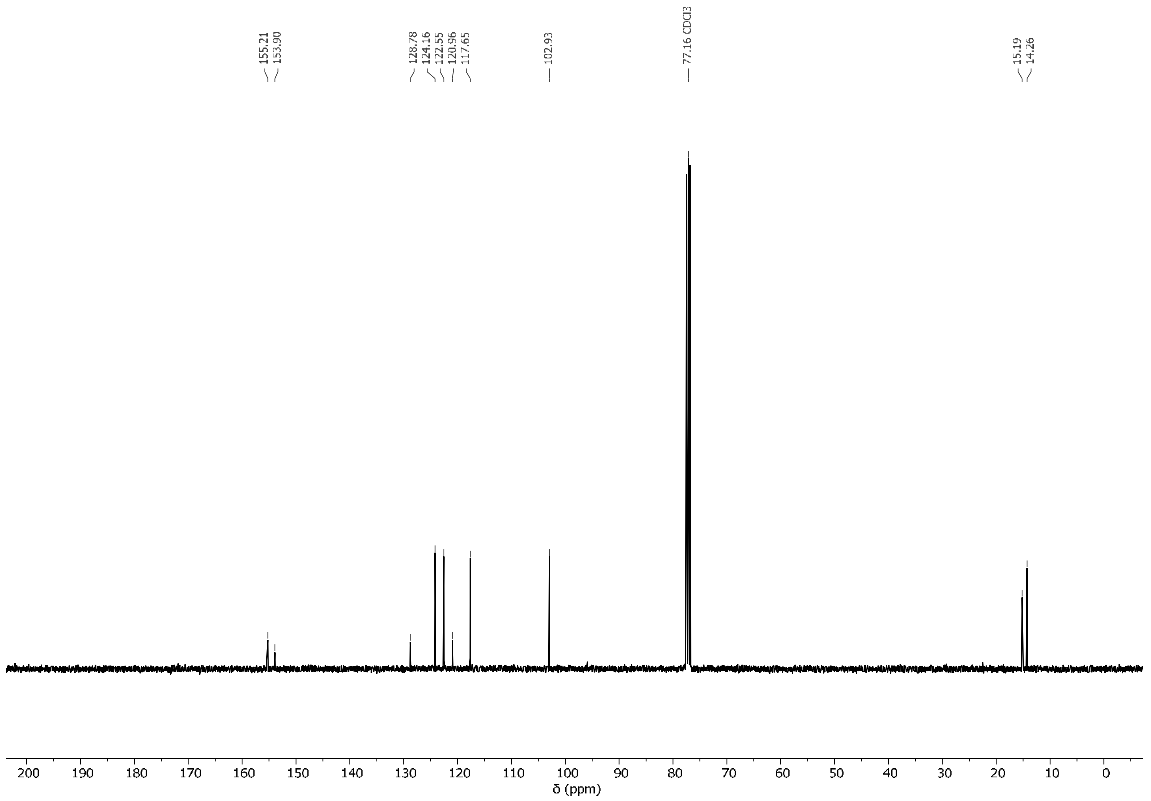
155.21
153.90
128.78
124.16
122.55
120.96
117.65
102.93
77.16 CDCl3
15.19
14.26
200 190 180 170 160 150 140 130 120 110 100 90 80 70 60 50 40 30 20 10 0
δ (ppm)

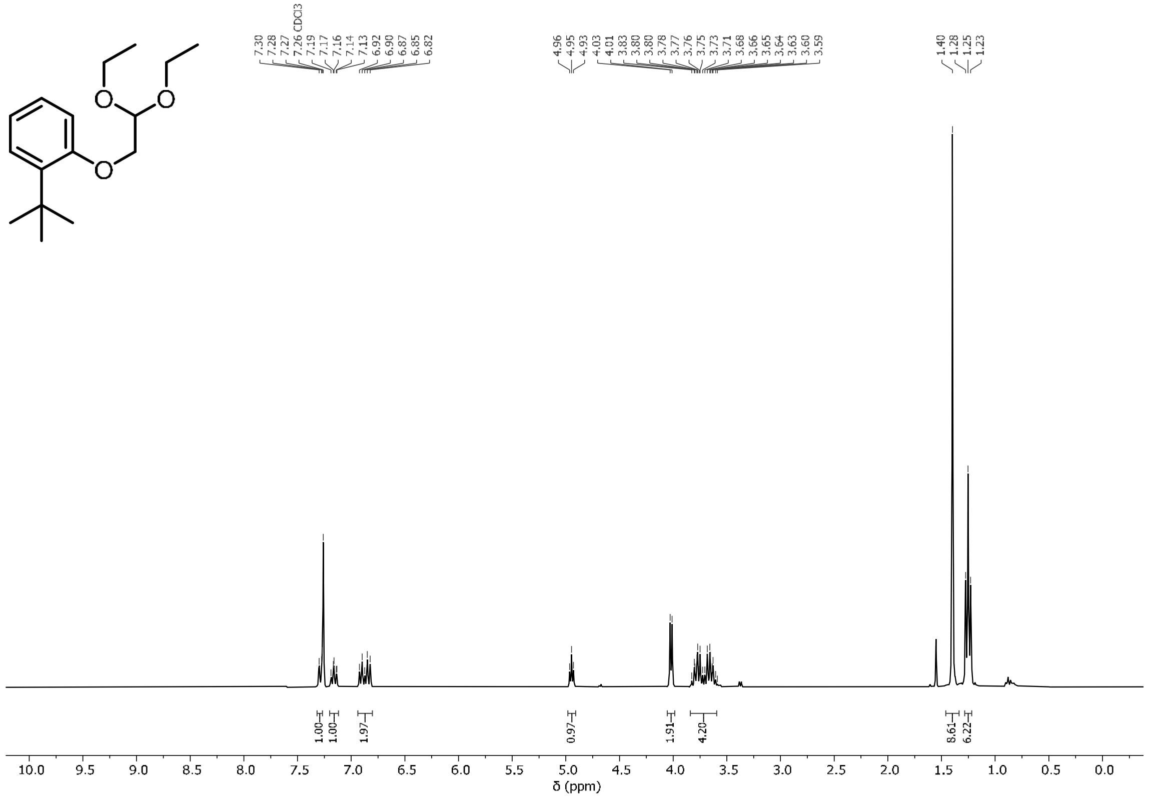
7.30
7.28
7.27
7.26 CDCl3
7.19
7.17
7.16
7.14
7.13
6.92
6.90
6.87
6.85
6.82
4.96
4.95
4.93
4.03
4.01
3.83
3.80
3.80
3.78
3.77
3.76
3.75
3.73
3.71
3.68
3.66
3.65
3.64
3.63
3.60
3.59
1.40
1.28
1.25
1.23
1.00
1.00
1.97
0.97
1.91
4.20
8.61
6.22
10.0 9.5 9.0 8.5 8.0 7.5 7.0 6.5 6.0 5.5 5.0 4.5 4.0 3.5 3.0 2.5 2.0 1.5 1.0 0.5 0.0
δ (ppm)

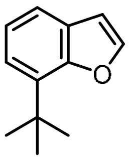

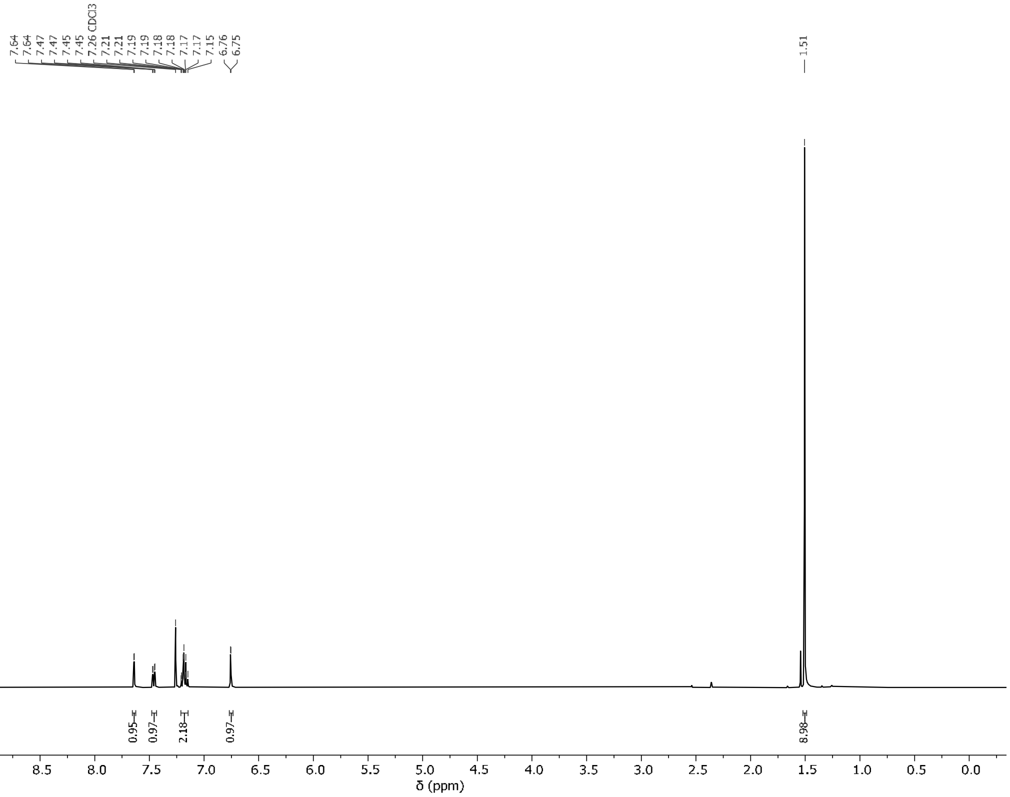
7.64
7.64
7.47
7.47
7.45
7.45
7.26 CDCl3
7.21
7.21
7.19
7.19
7.18
7.18
7.17
7.17
7.15
6.76
6.75
1.51
0.95
0.97
2.18
0.97
8.98
δ (ppm)

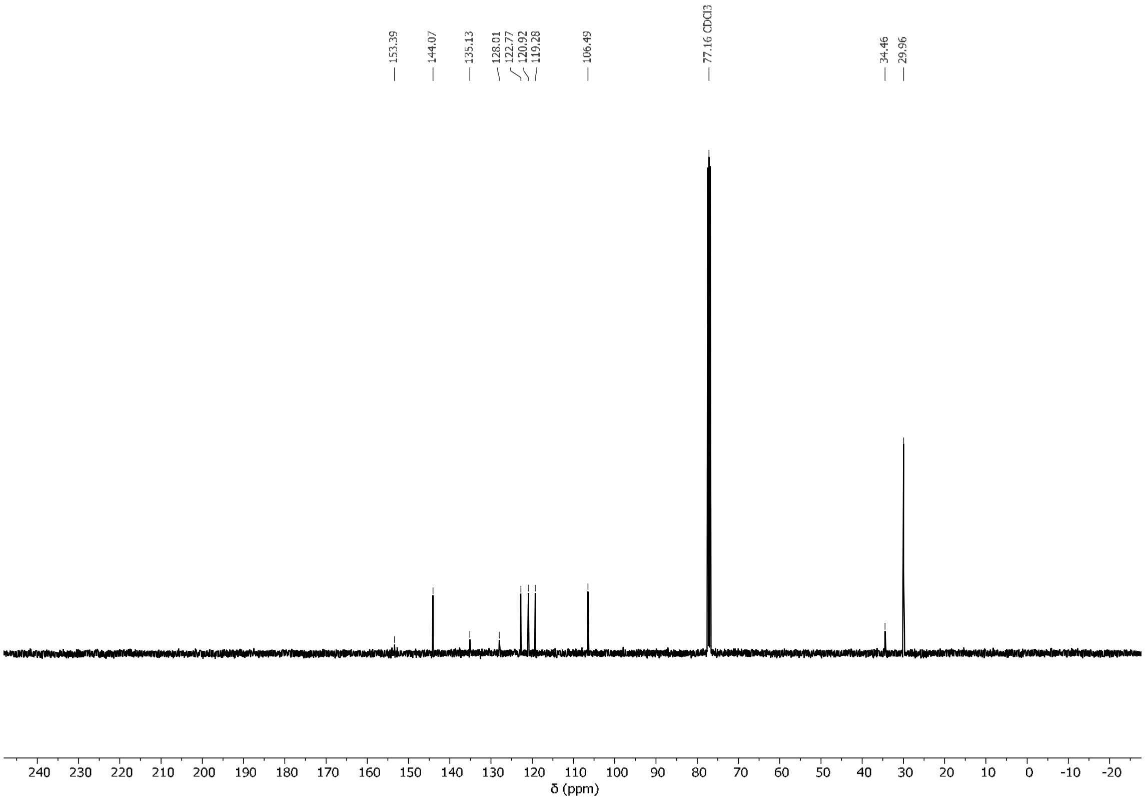
153.39
144.07
135.13
128.01
122.77
120.92
119.28
106.49
77.16 CDCl3
34.46
29.96
δ (ppm)

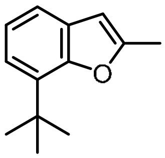

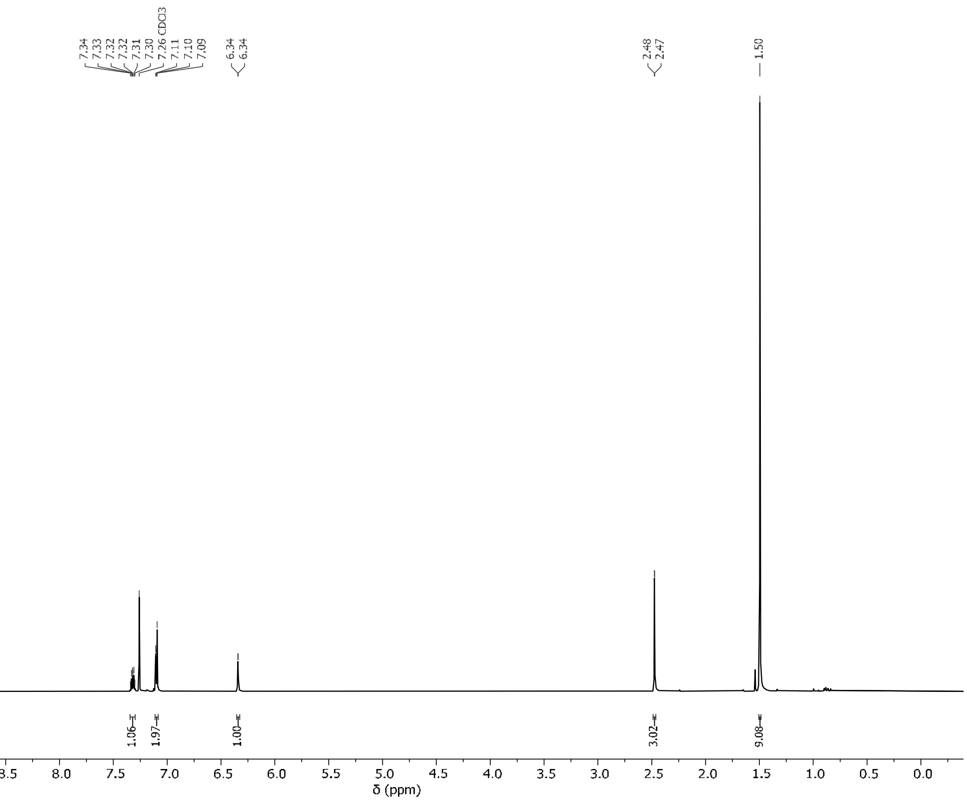
7.34
7.33
7.32
7.32
7.31
7.30
7.26 CDCl3
7.11
7.10
7.09
6.34
6.34
2.48
2.47
1.50
1.06
1.97
1.00
3.02
9.08
δ (ppm)

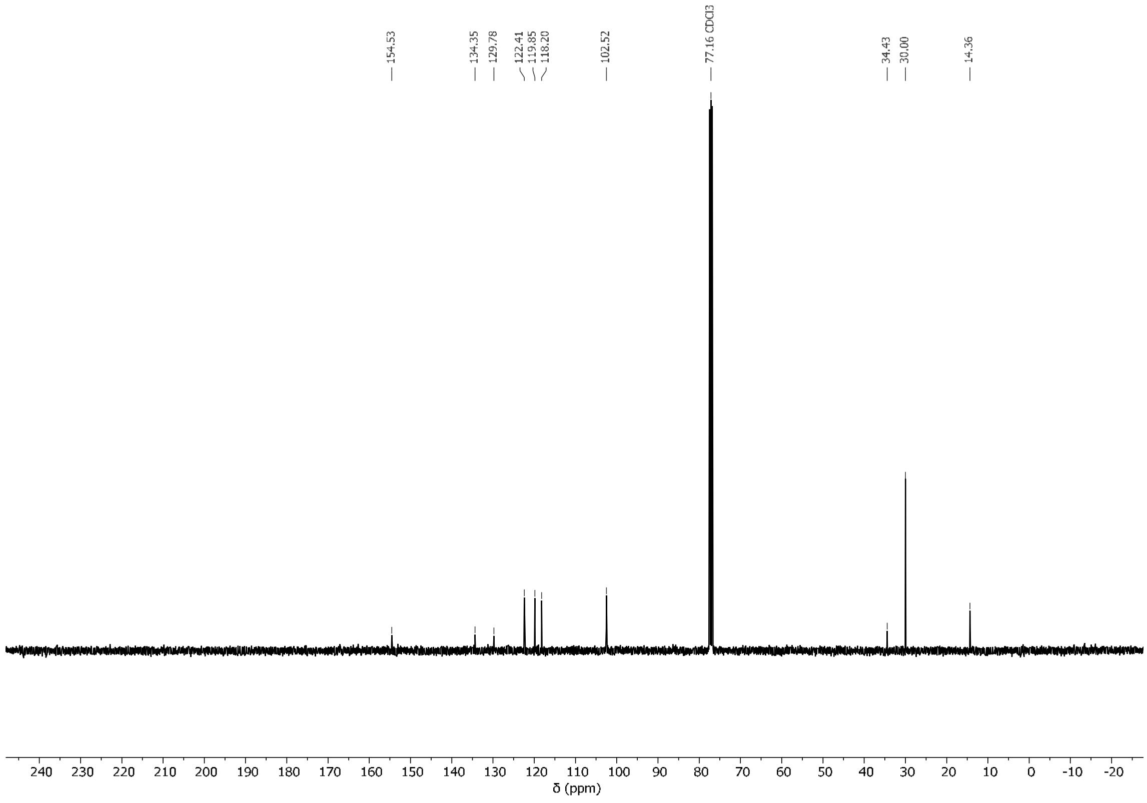
154.53
134.35
129.78
122.41
119.85
118.20
102.52
77.16 CDCl3
34.43
30.00
14.36
δ (ppm)

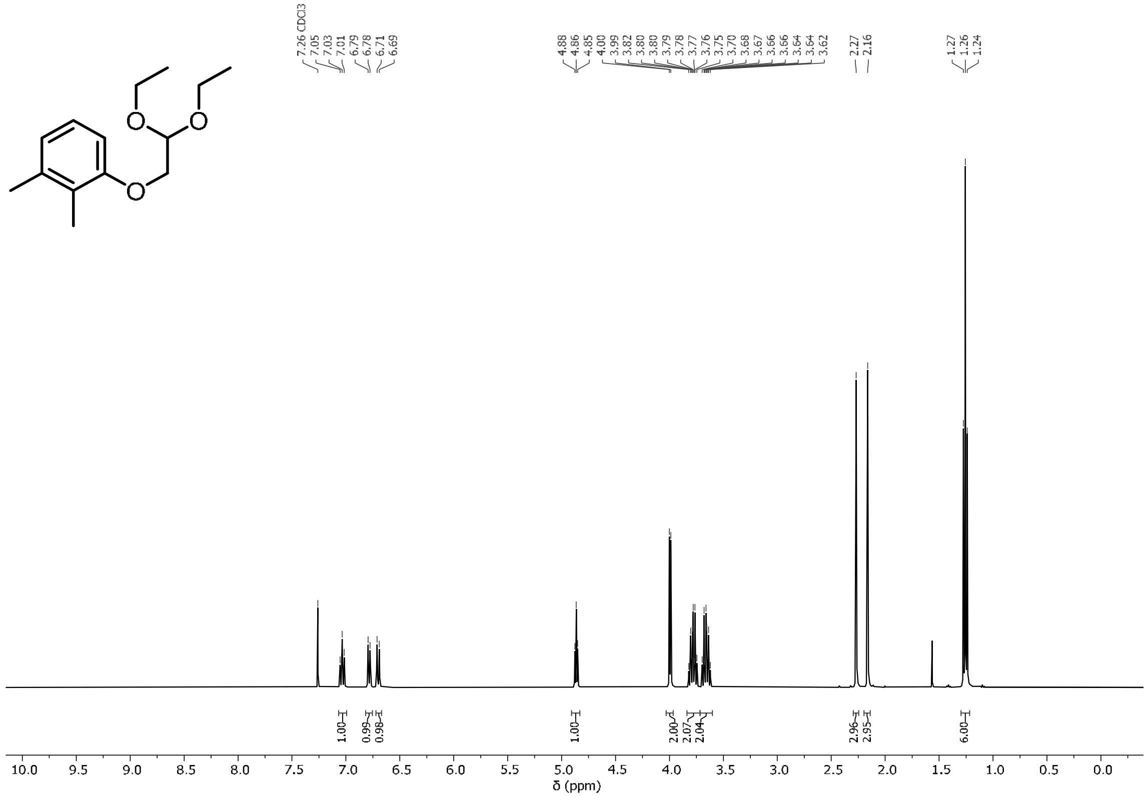
7.26 CDCl3
7.05
7.03
7.01
6.79
6.78
6.71
6.69
4.88
4.86
4.85
4.00
3.99
3.82
3.80
3.80
3.79
3.78
3.77
3.76
3.75
3.70
3.68
3.67
3.66
3.66
3.64
3.64
3.62
2.27
2.16
1.27
1.26
1.24
1.00
0.99
0.98
1.00
2.00
2.07
2.04
2.96
2.95
6.00
δ (ppm)

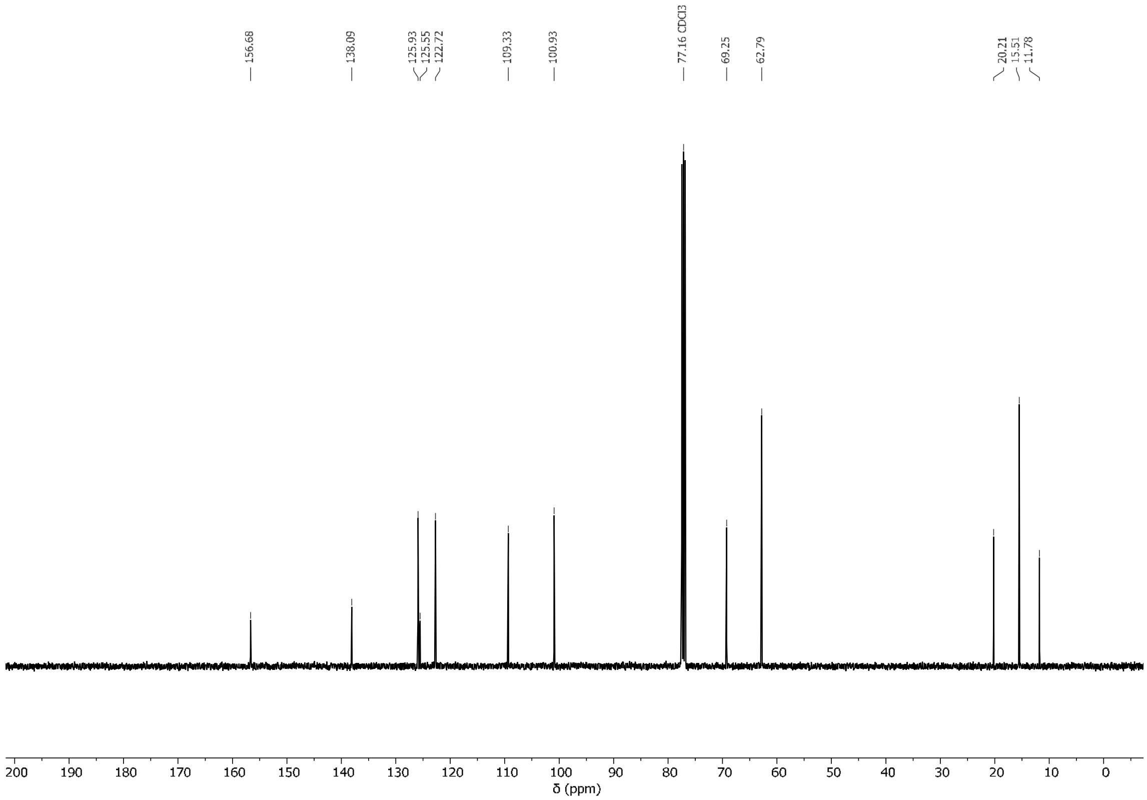
156.68
138.09
125.93
125.55
122.72
109.33
100.93
77.16 CDCl3
69.25
62.79
20.21
15.51
11.78
δ (ppm)

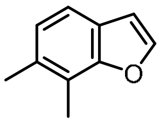
O

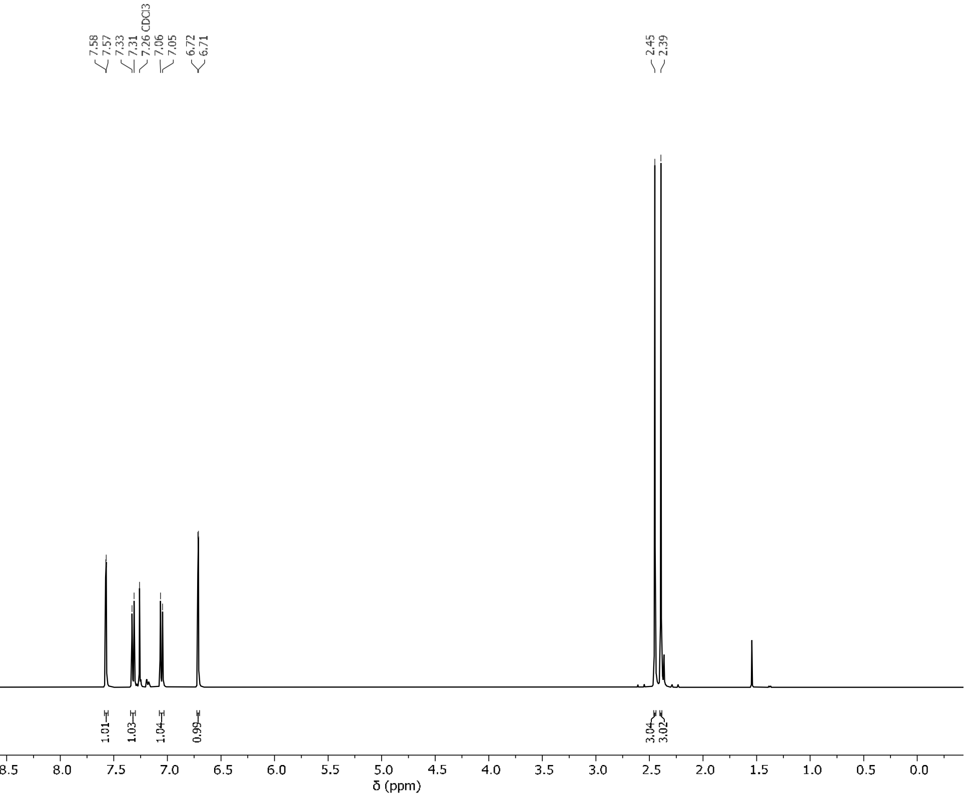
7.58
7.57
7.33
7.31
7.26 CDCl3
7.06
7.05
6.72
6.71
2.45
2.39
1.01
1.03
1.04
0.99
3.04
3.02
10.0 9.5 9.0 8.5 8.0 7.5 7.0 6.5 6.0 5.5 5.0 4.5 4.0 3.5 3.0 2.5 2.0 1.5 1.0 0.5 0.0
δ (ppm)

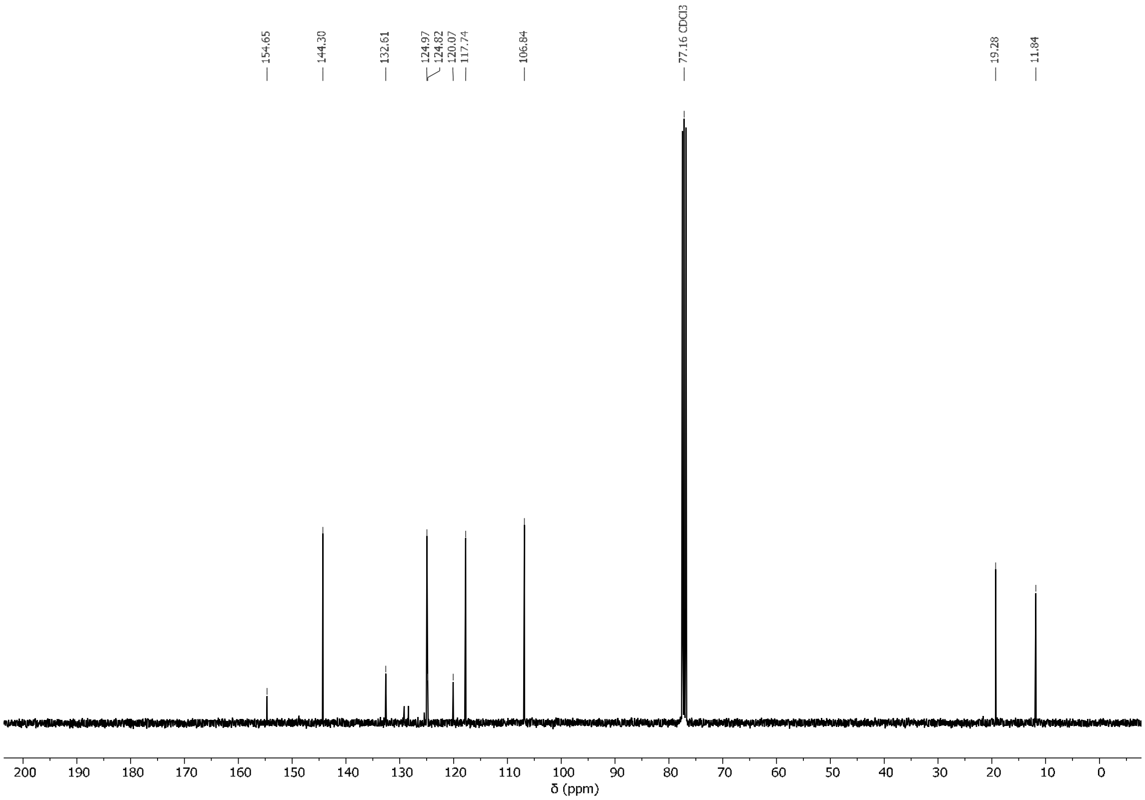
154.65
144.30
132.61
124.97
124.82
120.07
117.74
106.84
77.16 CDCl3
19.28
11.84
200 190 180 170 160 150 140 130 120 110 100 90 80 70 60 50 40 30 20 10 0
δ (ppm)

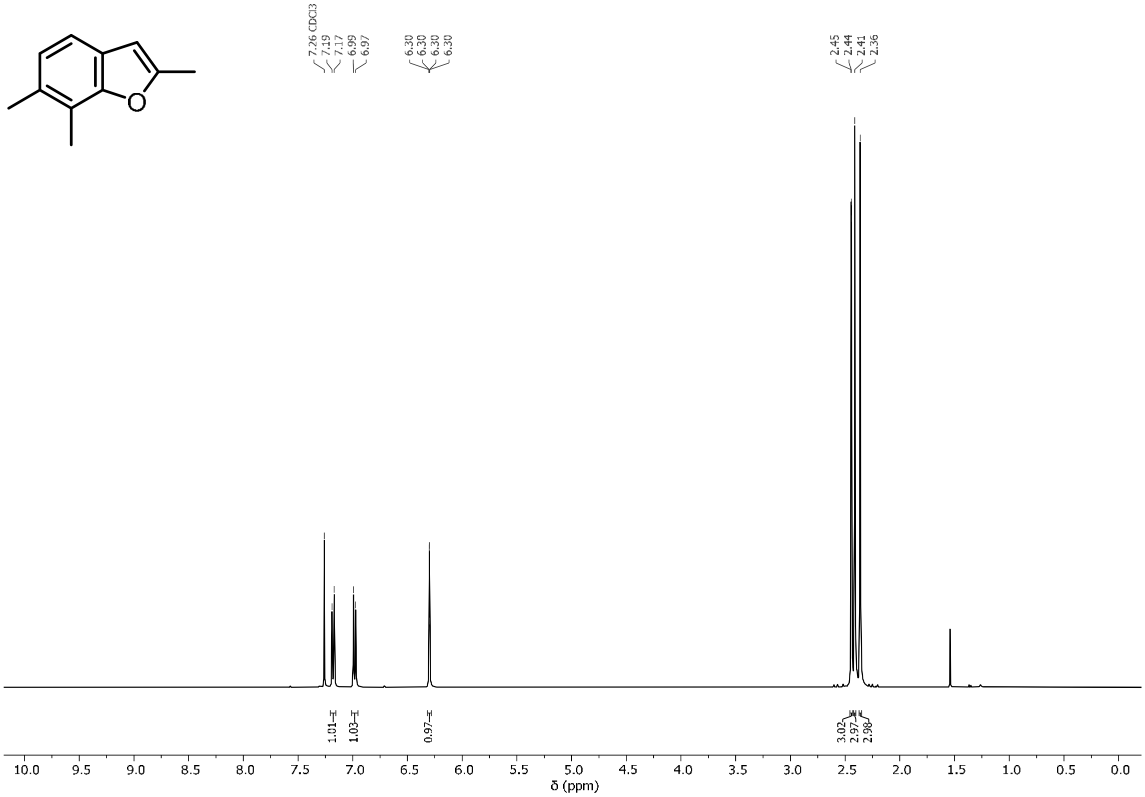
7.26 CDCl3
7.19
7.17
6.99
6.97
6.30
6.30
6.30
6.30
2.45
2.44
2.41
2.36
1.01
1.03
0.97
3.02
2.97
2.98
δ (ppm)

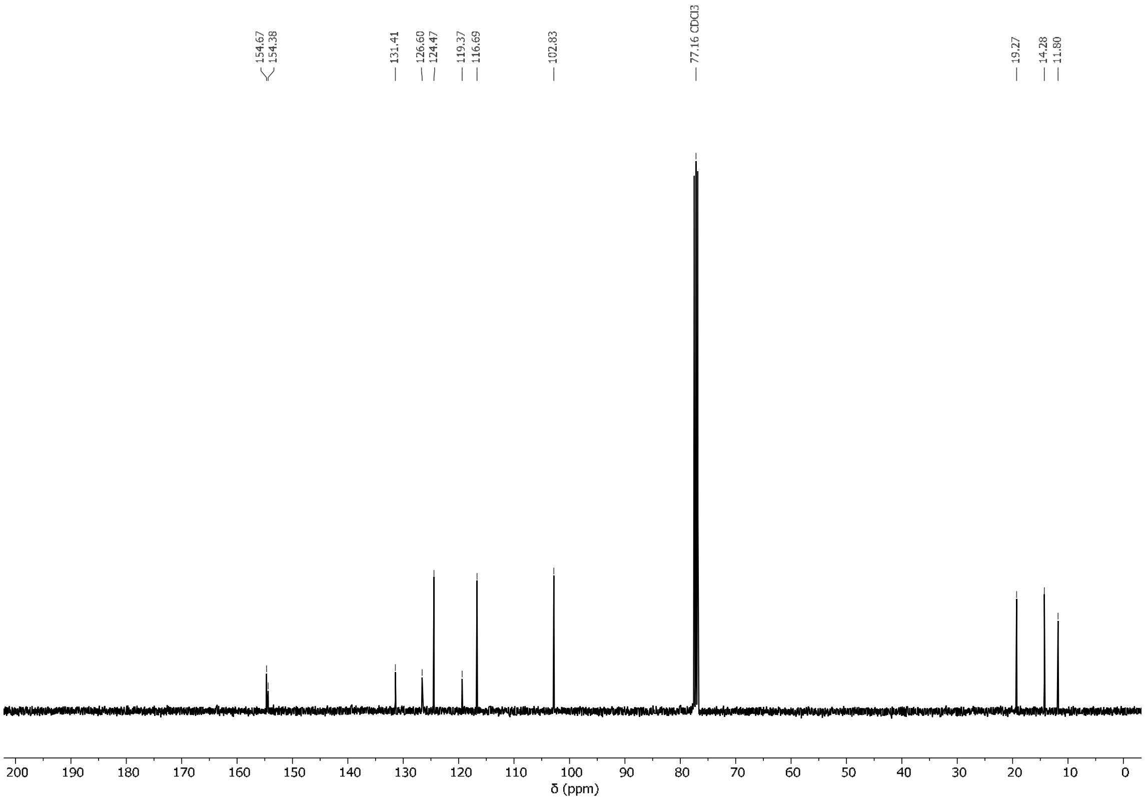
154.67
154.38
131.41
126.60
124.47
119.37
116.69
102.83
77.16 CDCl3
19.27
14.28
11.80
δ (ppm)

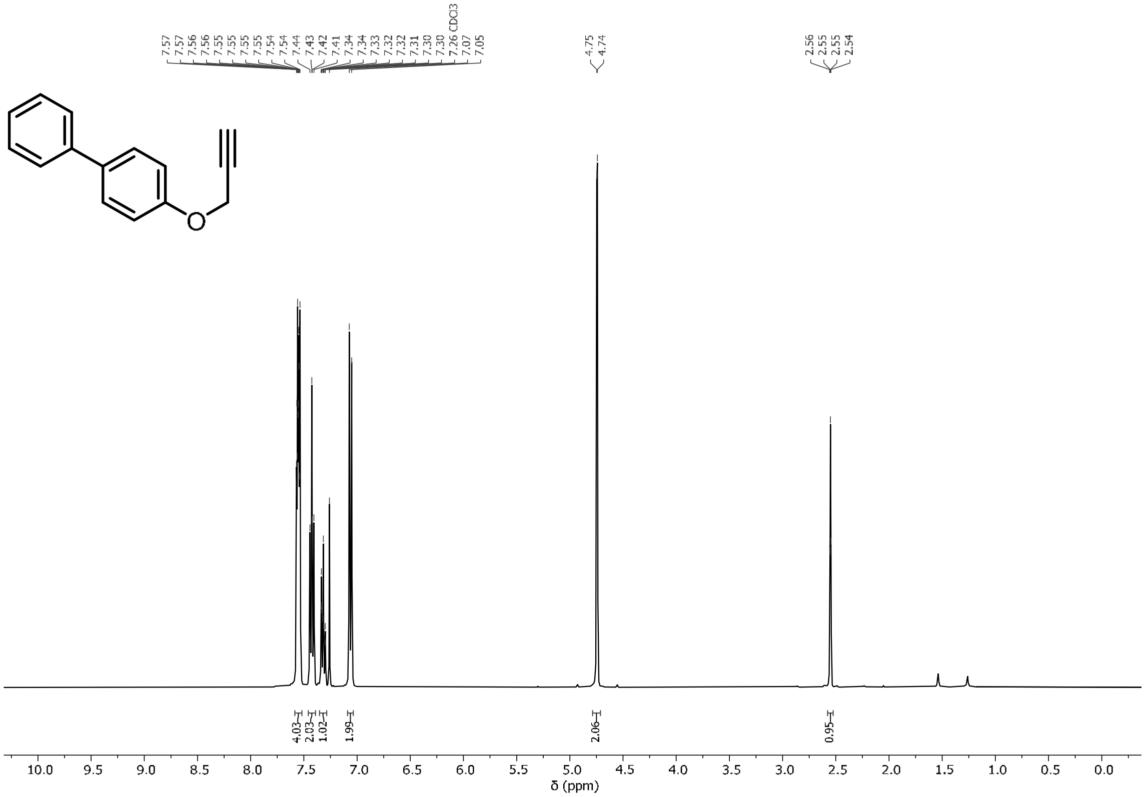
7.57
7.57
7.56
7.56
7.55
7.55
7.55
7.55
7.54
7.54
7.44
7.43
7.42
7.41
7.34
7.34
7.33
7.32
7.32
7.31
7.30
7.26 CDCl3
7.07
7.05
4.75
4.74
2.56
2.55
2.55
2.54
O
4.03
2.03
1.02
1.99
2.06
0.95
10.0
9.5
9.0
8.5
8.0
7.5
7.0
6.5
6.0
5.5
5.0
4.5
4.0
3.5
3.0
2.5
2.0
1.5
1.0
0.5
0.0
δ (ppm)

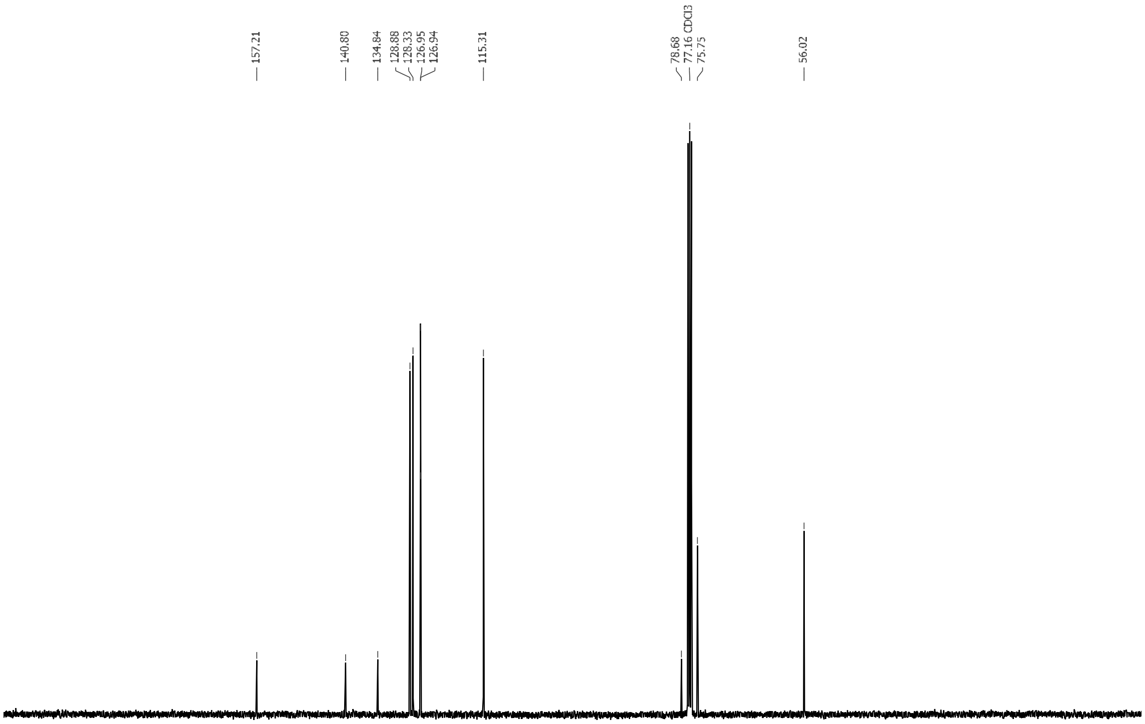
157.21
140.80
134.84
128.88
128.33
126.95
126.94
115.31
78.68
77.16 CDCl3
75.75
56.02
200
190
180
170
160
150
140
130
120
110
100
90
80
70
60
50
40
30
20
10
0
δ (ppm)

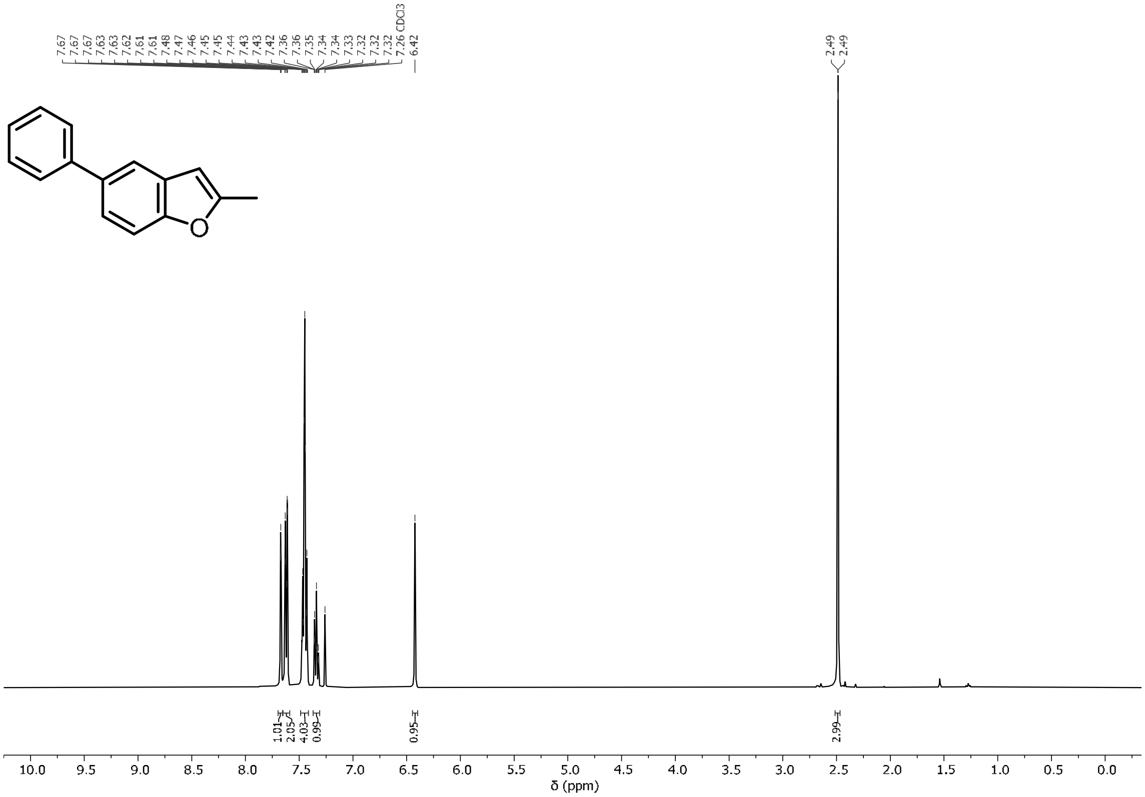
7.67
7.67
7.67
7.63
7.63
7.62
7.61
7.61
7.48
7.47
7.46
7.45
7.45
7.44
7.43
7.43
7.42
7.36
7.36
7.35
7.34
7.34
7.33
7.32
7.32
7.26 CDCl3
6.42
2.49
2.49
O
1.01
2.05
4.03
0.99
0.95
2.99
10.0 9.5 9.0 8.5 8.0 7.5 7.0 6.5 6.0 5.5 5.0 4.5 4.0 3.5 3.0 2.5 2.0 1.5 1.0 0.5 0.0
δ (ppm)

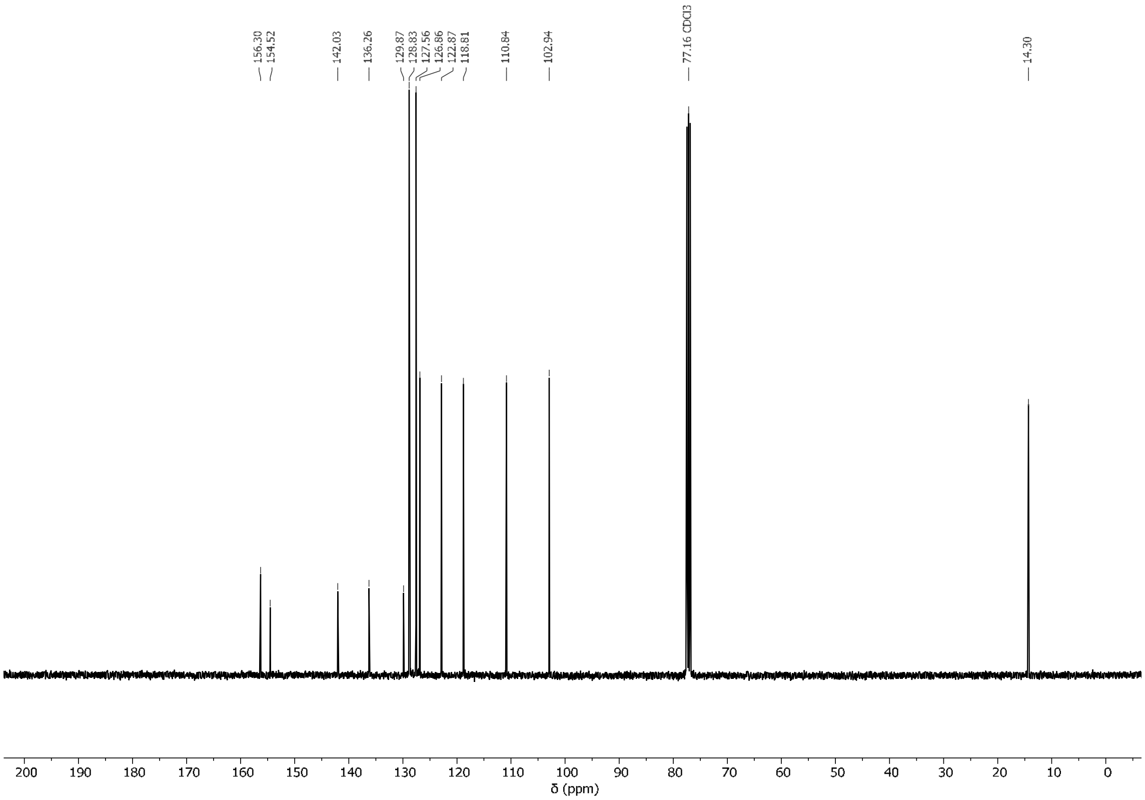
156.30
154.52
142.03
136.26
129.87
128.83
127.56
126.86
122.87
118.81
110.84
102.94
77.16 CDCl3
14.30
200 190 180 170 160 150 140 130 120 110 100 90 80 70 60 50 40 30 20 10 0
δ (ppm)

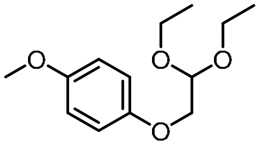

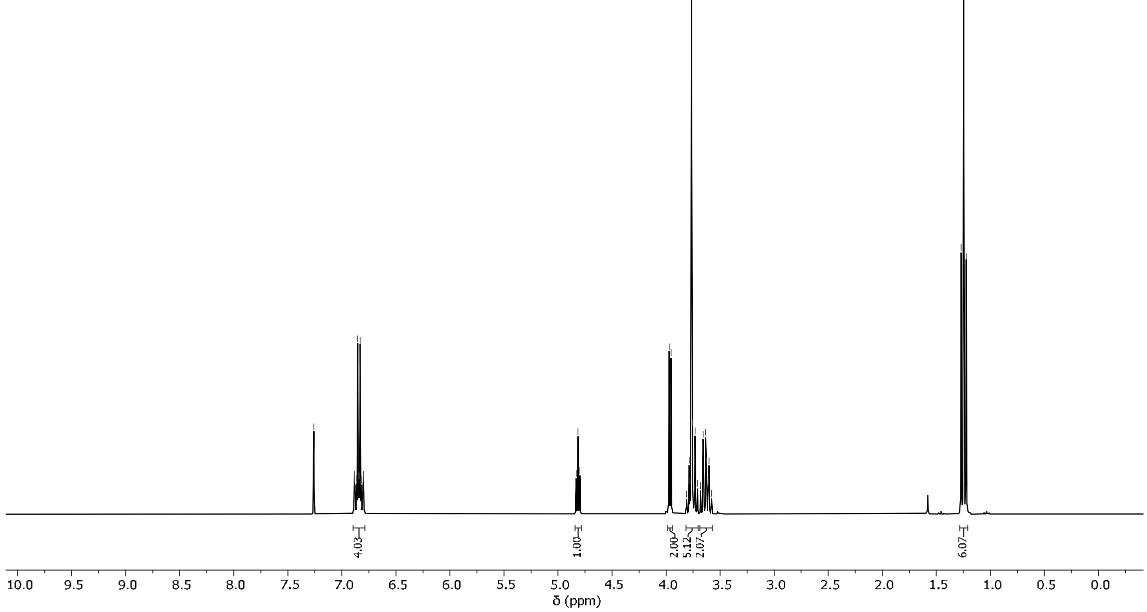

7.26 CDCl3
6.89 6.89 6.88 6.88 6.86 6.85 6.84 6.84 6.83 6.82 6.82 6.81 6.80 6.80 6.79
4.83 4.81 4.80
3.97 3.95 3.81 3.79 3.78 3.76 3.76 3.74 3.73 3.71 3.68 3.66 3.65 3.63 3.63 3.61 3.60 3.58
1.27 1.25 1.22
4.03
1.00
2.00
5.12
2.07
6.07
10.0 9.5 9.0 8.5 8.0 7.5 7.0 6.5 6.0 5.5 5.0 4.5 4.0 3.5 3.0 2.5 2.0 1.5 1.0 0.5 0.0
δ (ppm)

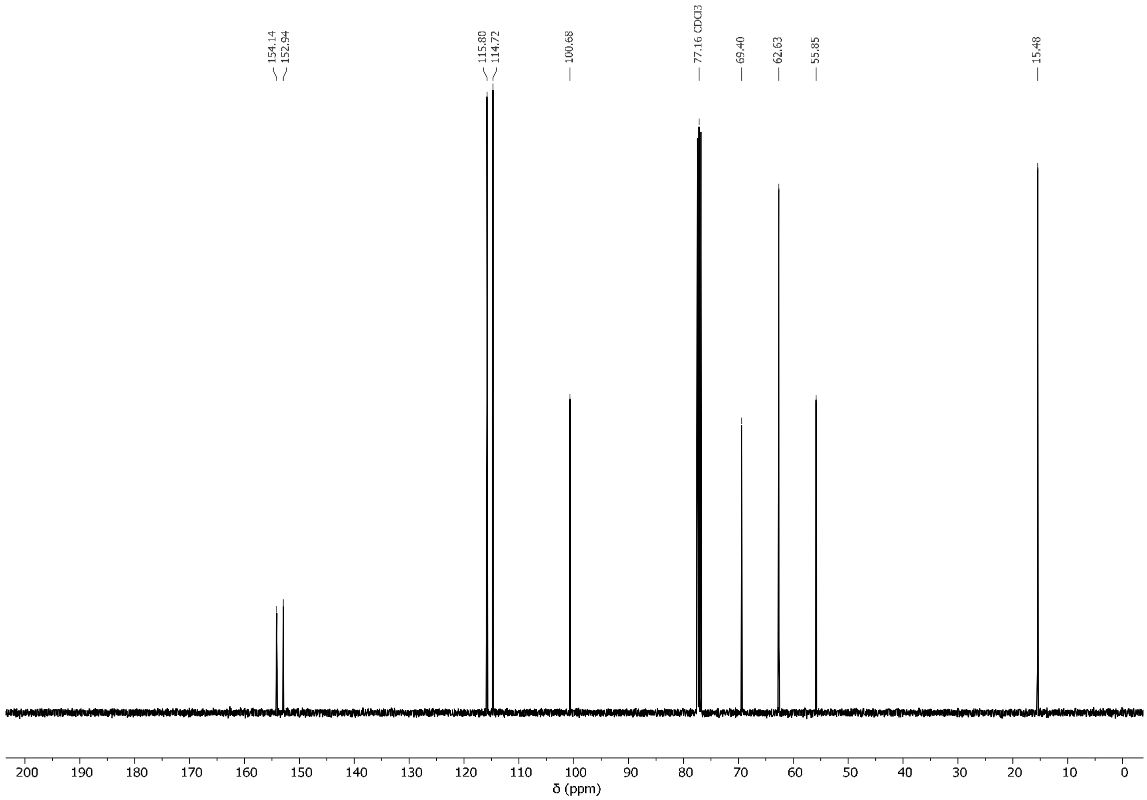

154.14 152.94
115.80 114.72
100.68
77.16 CDCl3
69.40
62.63
55.85
15.48
200 190 180 170 160 150 140 130 120 110 100 90 80 70 60 50 40 30 20 10 0
δ (ppm)

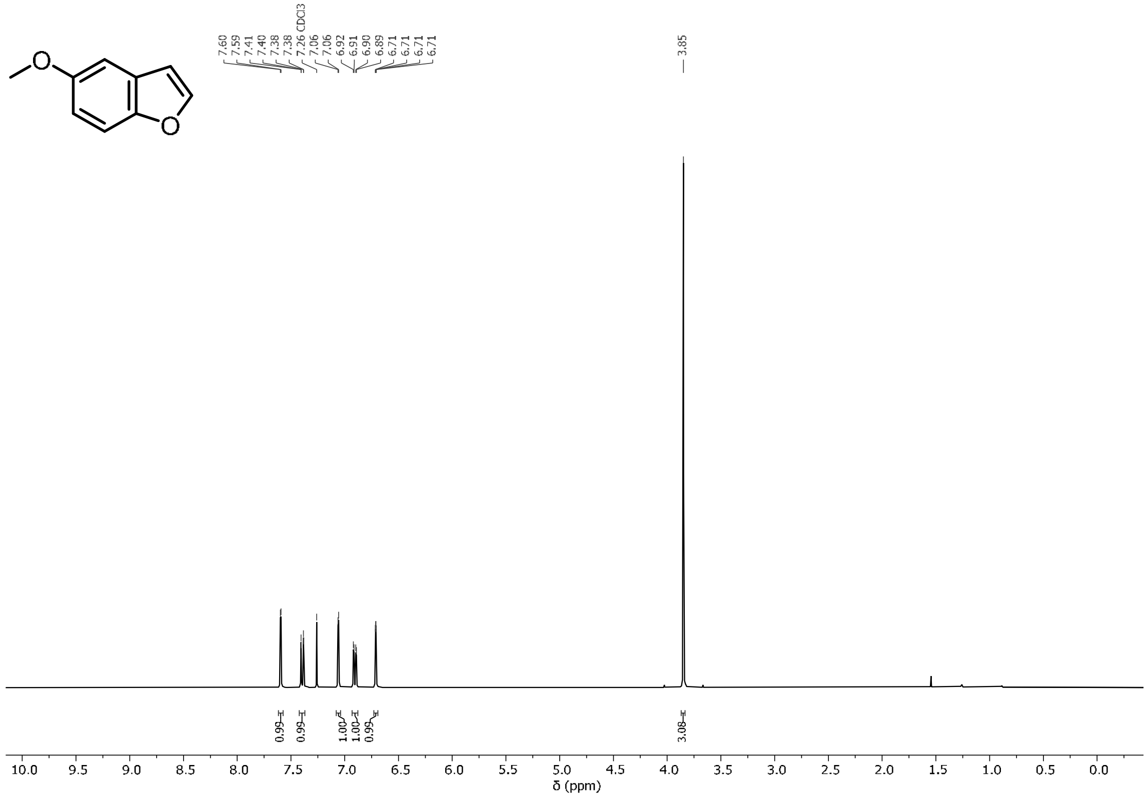

7.60
7.59
7.41
7.40
7.38
7.38
7.26 CDCl3
7.06
7.06
6.92
6.91
6.90
6.89
6.71
6.71
6.71
6.71
3.85
0.99
0.99
1.00
1.00
0.99
3.08
10.0 9.5 9.0 8.5 8.0 7.5 7.0 6.5 6.0 5.5 5.0 4.5 4.0 3.5 3.0 2.5 2.0 1.5 1.0 0.5 0.0
δ (ppm)

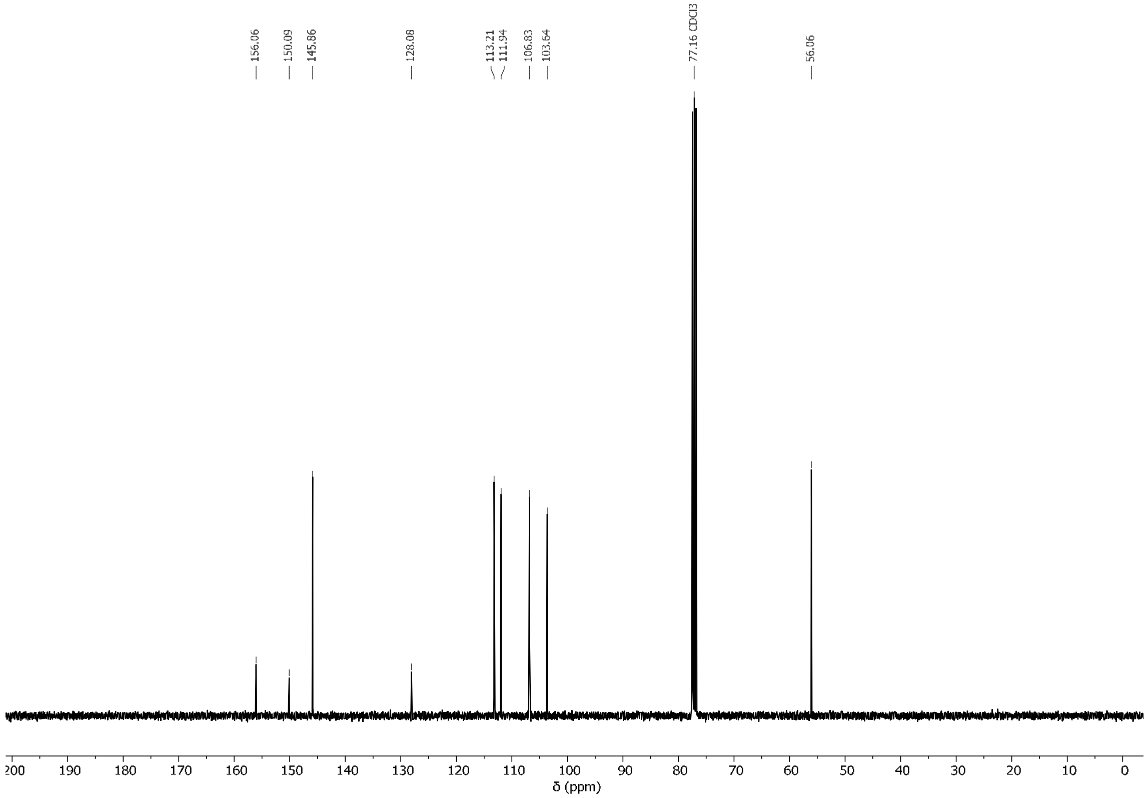

156.06
150.09
145.86
128.08
113.21
111.94
106.83
103.64
77.16 CDCl3
56.06
200 190 180 170 160 150 140 130 120 110 100 90 80 70 60 50 40 30 20 10 0
δ (ppm)

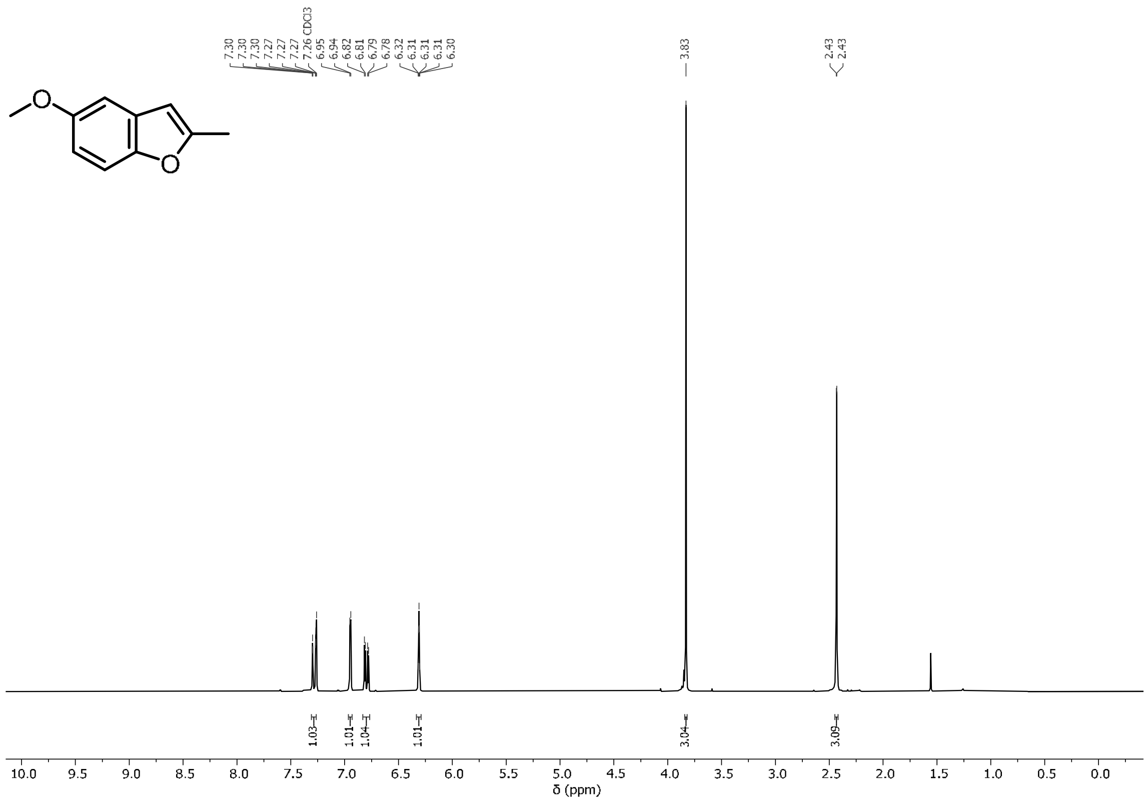
7.30
7.30
7.30
7.27
7.27
7.27
7.26 CDCl3
6.95
6.94
6.82
6.81
6.79
6.78
6.32
6.31
6.31
6.31
6.30
3.83
2.43
2.43
O
O
1.03
1.01
1.04
1.01
3.04
3.09
10.0 9.5 9.0 8.5 8.0 7.5 7.0 6.5 6.0 5.5 5.0 4.5 4.0 3.5 3.0 2.5 2.0 1.5 1.0 0.5 0.0
δ (ppm)

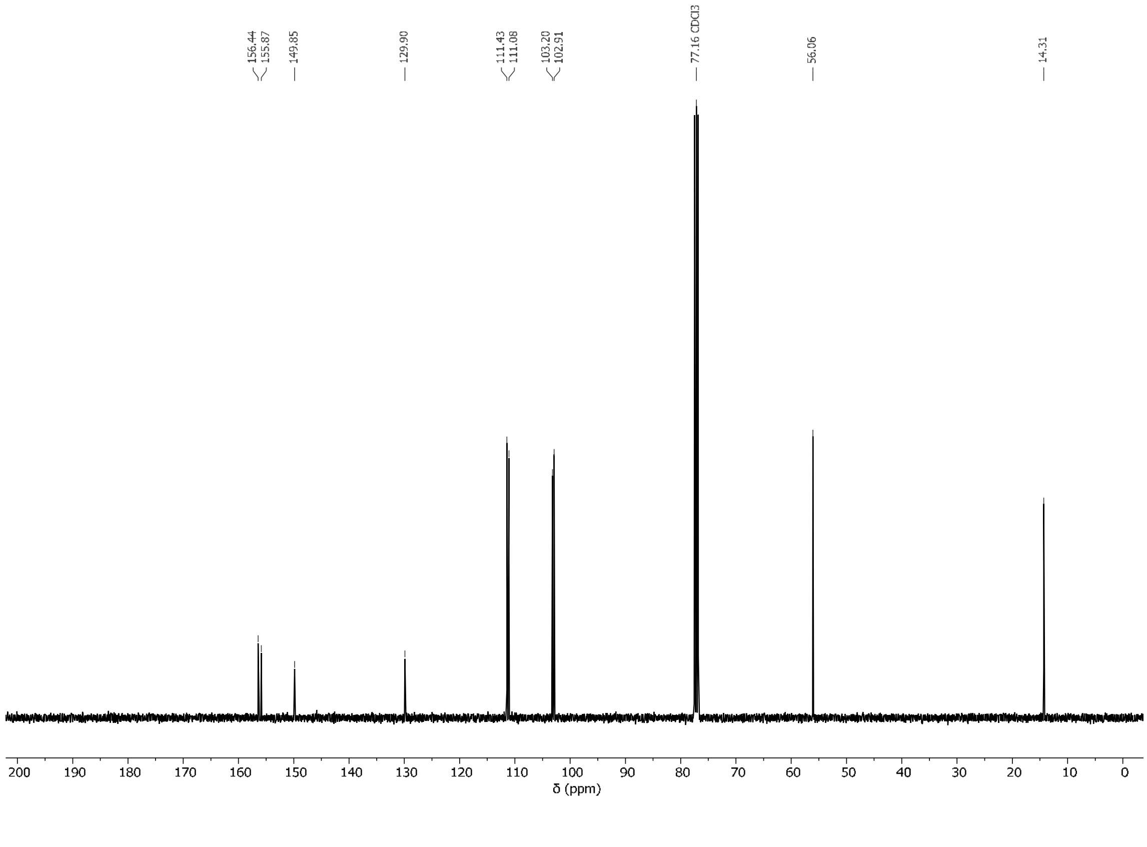
156.44
155.87
149.85
129.90
111.43
111.08
103.20
102.91
77.16 CDCl3
56.06
14.31
200 190 180 170 160 150 140 130 120 110 100 90 80 70 60 50 40 30 20 10 0
δ (ppm)

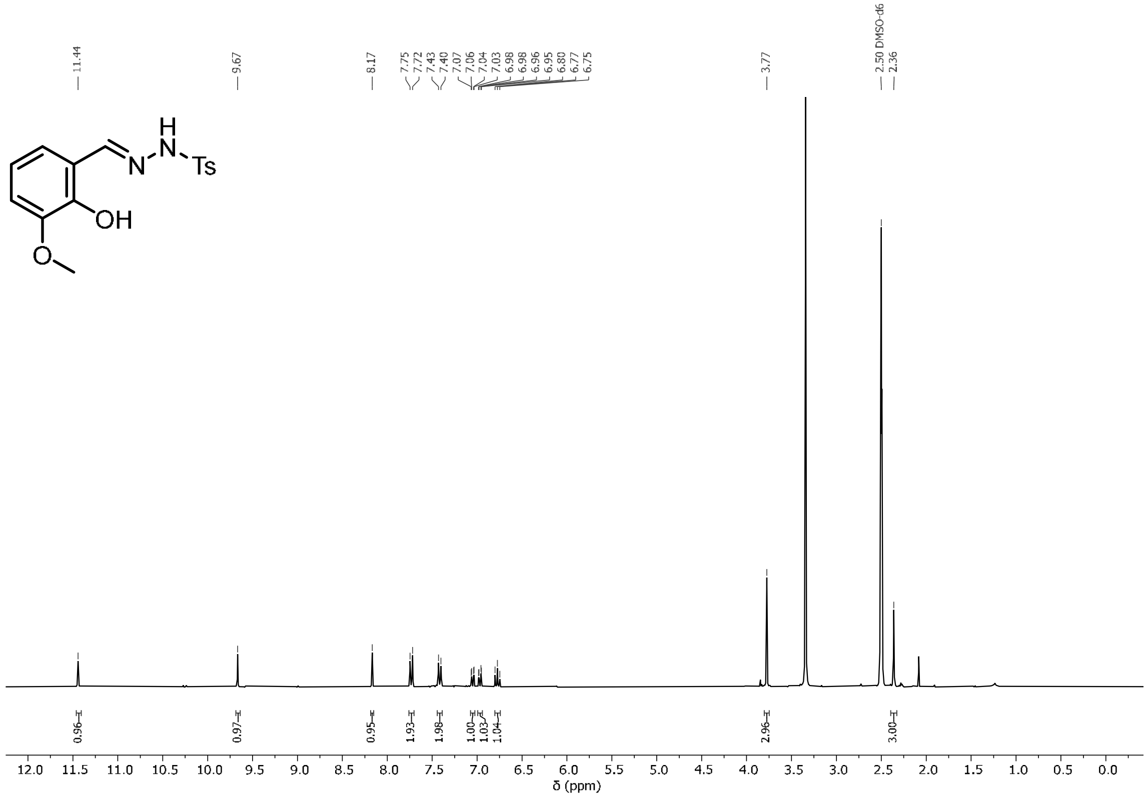

11.44
9.67
8.17
7.75
7.72
7.43
7.40
7.07
7.06
7.04
7.03
6.98
6.98
6.96
6.95
6.80
6.77
6.75
3.77
2.50 DMSO-d6
2.36
H
N
N
Ts
OH
O
0.96
0.97
0.95
1.93
1.98
1.00
1.03
1.04
2.96
3.00
δ (ppm)

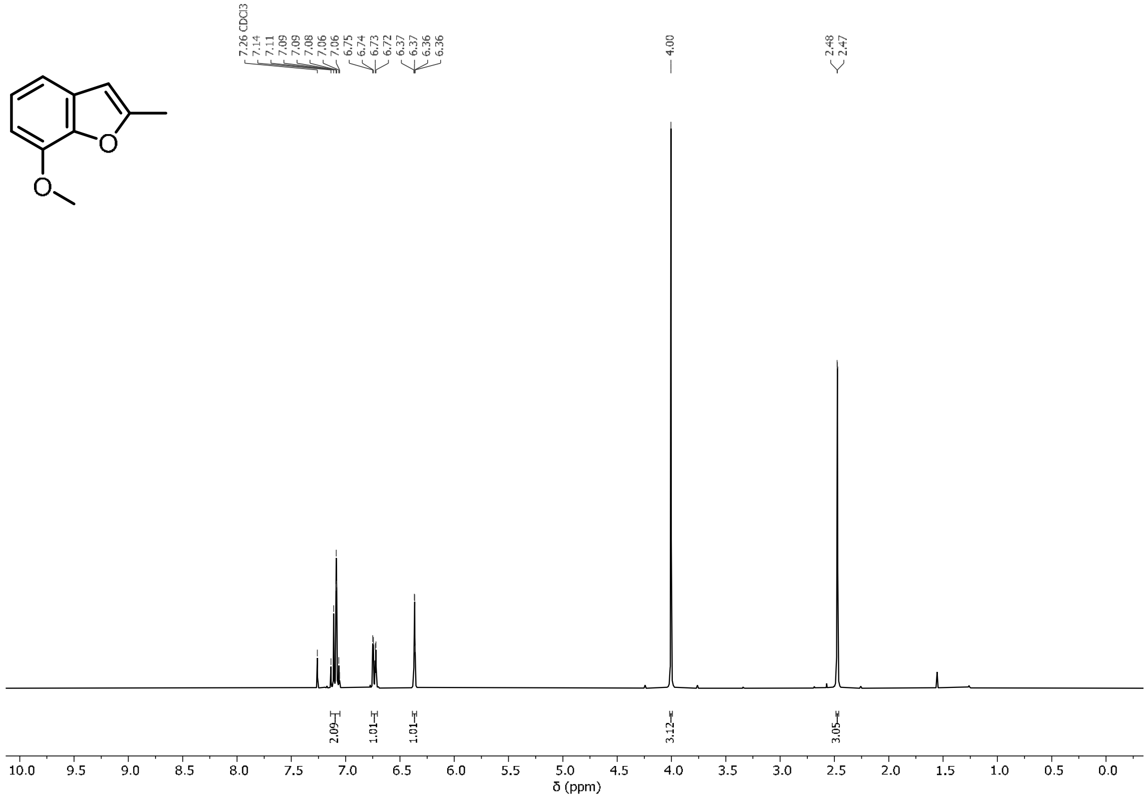

7.26 CDCl3
7.14
7.11
7.09
7.09
7.08
7.06
7.06
6.75
6.74
6.73
6.72
6.37
6.37
6.36
6.36
4.00
2.48
2.47
O
O
2.09
1.01
1.01
3.12
3.05
δ (ppm)

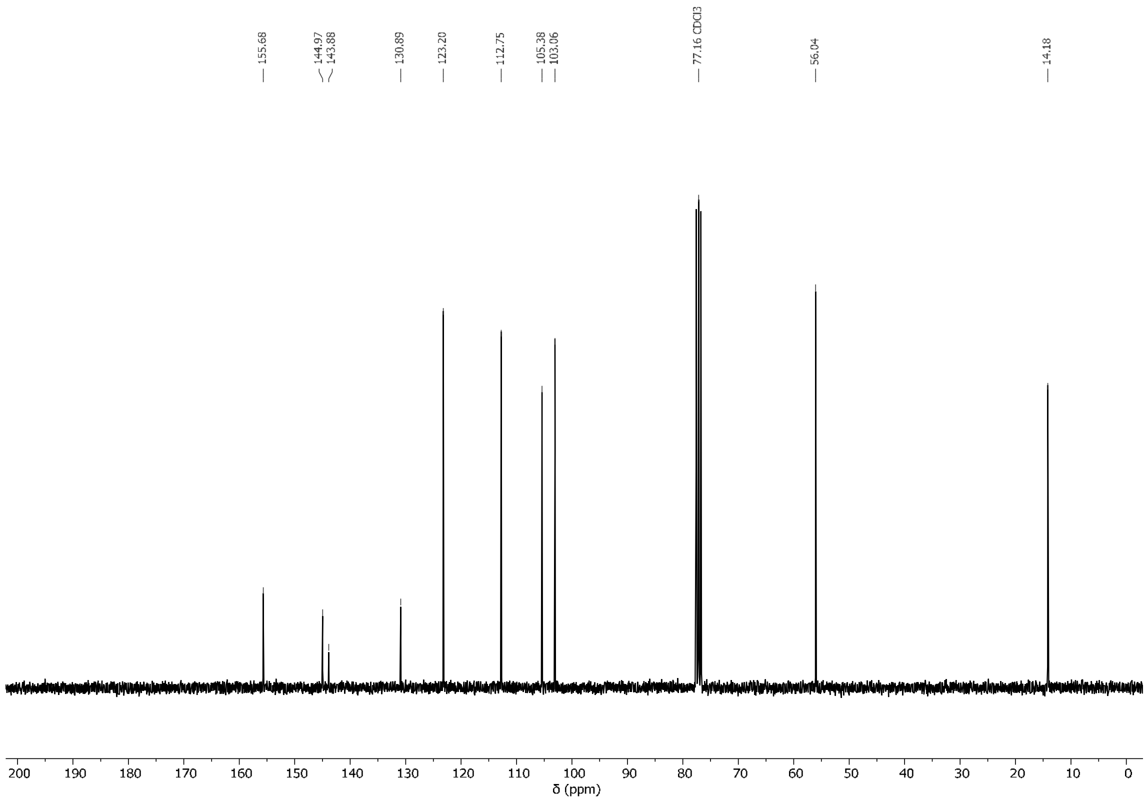
155.68
144.97
143.88
130.89
123.20
112.75
105.38
103.06
77.16 CDCl3
56.04
14.18
200 190 180 170 160 150 140 130 120 110 100 90 80 70 60 50 40 30 20 10 0
δ (ppm)

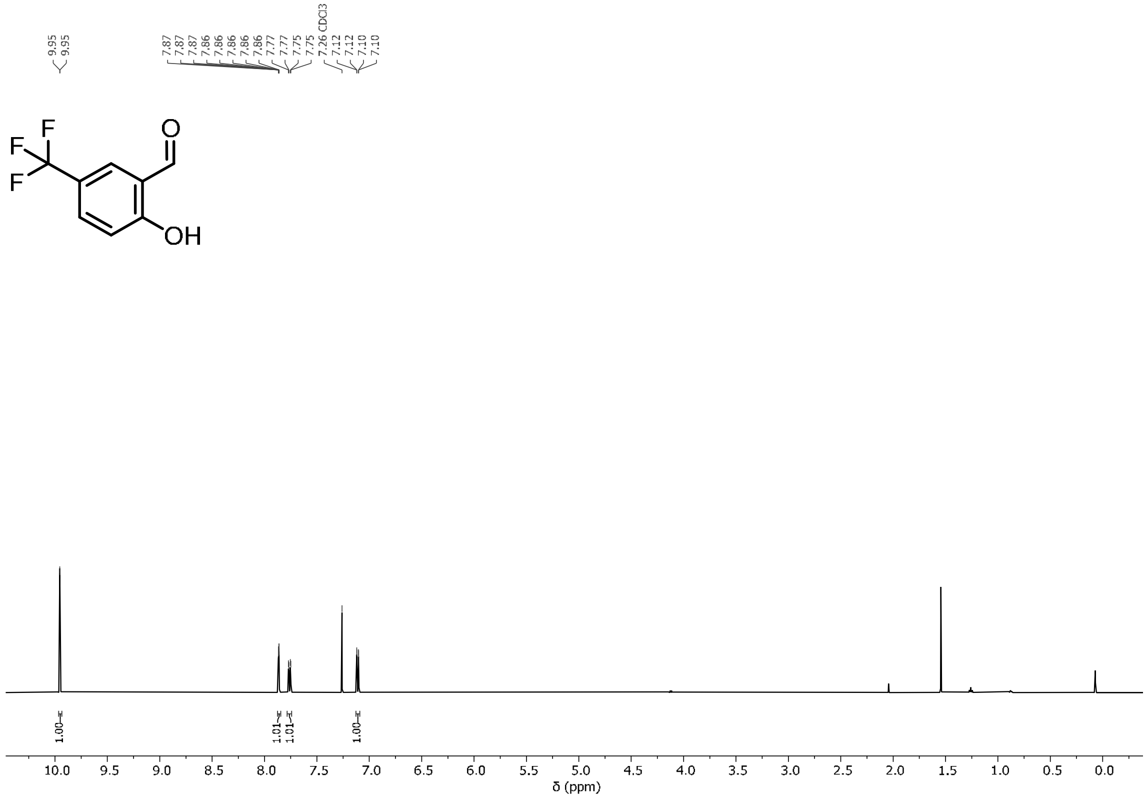
9.95
9.95
7.87
7.87
7.87
7.86
7.86
7.86
7.86
7.86
7.77
7.77
7.75
7.75
7.26 CDCl3
7.12
7.12
7.10
7.10
F
F
F
O
OH
1.00
1.01
1.01
1.00
10.0 9.5 9.0 8.5 8.0 7.5 7.0 6.5 6.0 5.5 5.0 4.5 4.0 3.5 3.0 2.5 2.0 1.5 1.0 0.5 0.0
δ (ppm)

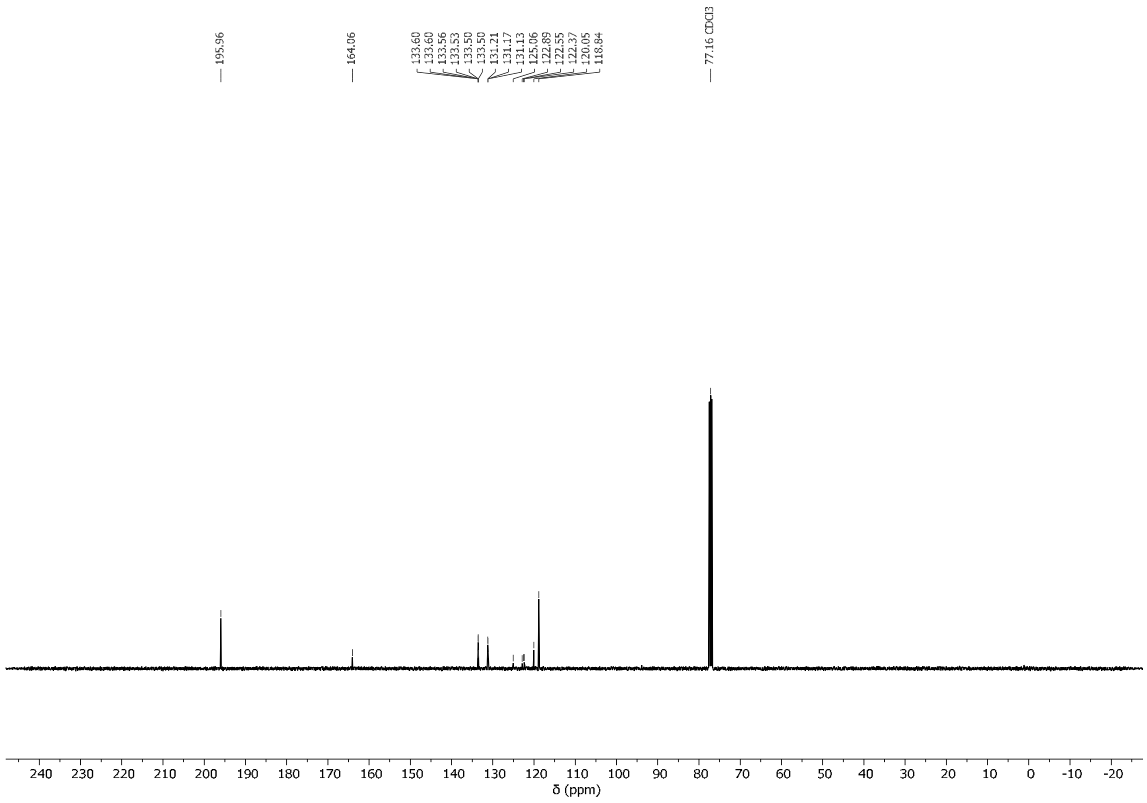
195.96
164.06
133.60
133.60
133.56
133.53
133.50
133.50
131.21
131.17
131.13
125.06
122.89
122.55
122.37
120.05
118.84
77.16 CDCl3
δ (ppm)

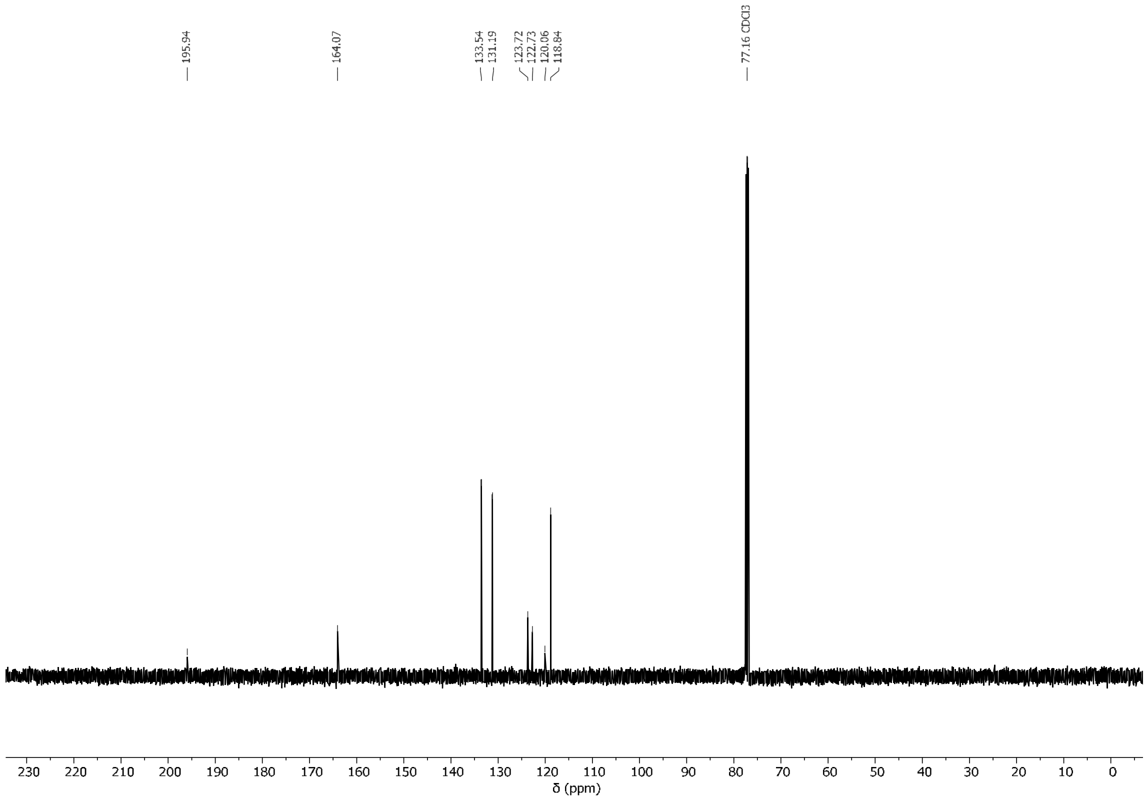
195.94
164.07
133.54
131.19
123.72
122.73
120.06
118.84
77.16 CDCl3
δ (ppm)

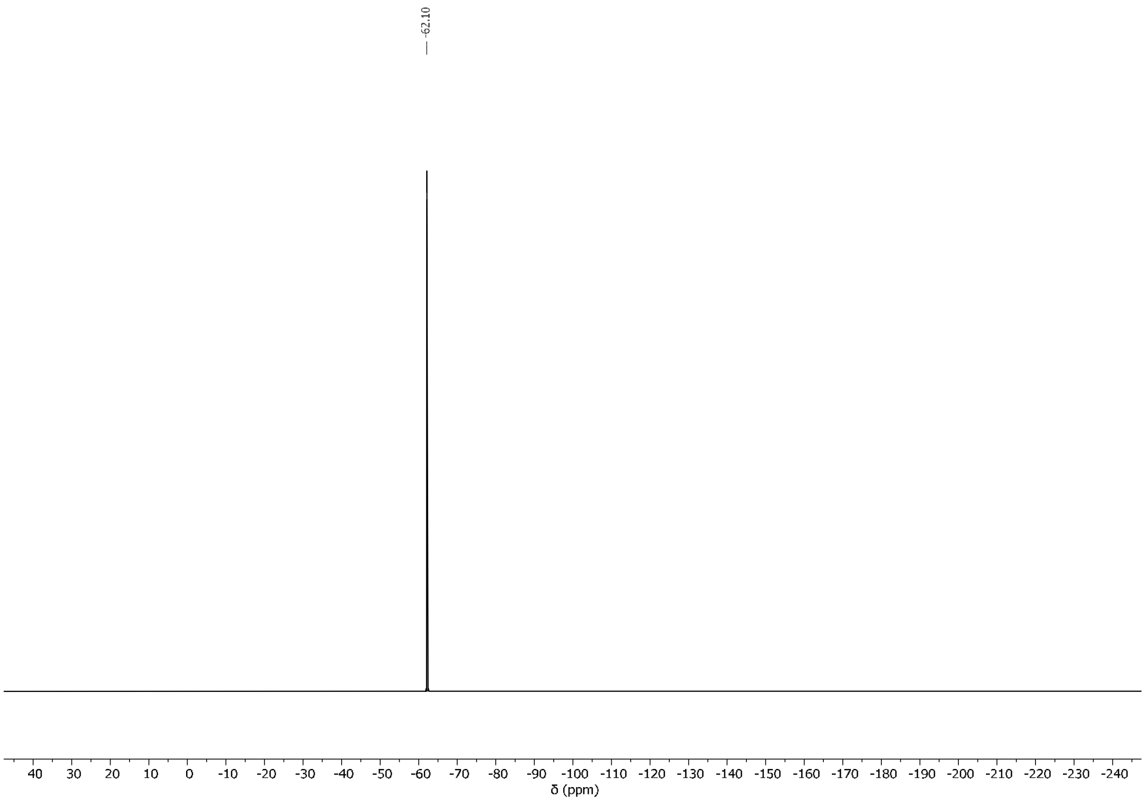
-62.10
40 30 20 10 0 -10 -20 -30 -40 -50 -60 -70 -80 -90 -100 -110 -120 -130 -140 -150 -160 -170 -180 -190 -200 -210 -220 -230 -240
δ (ppm)

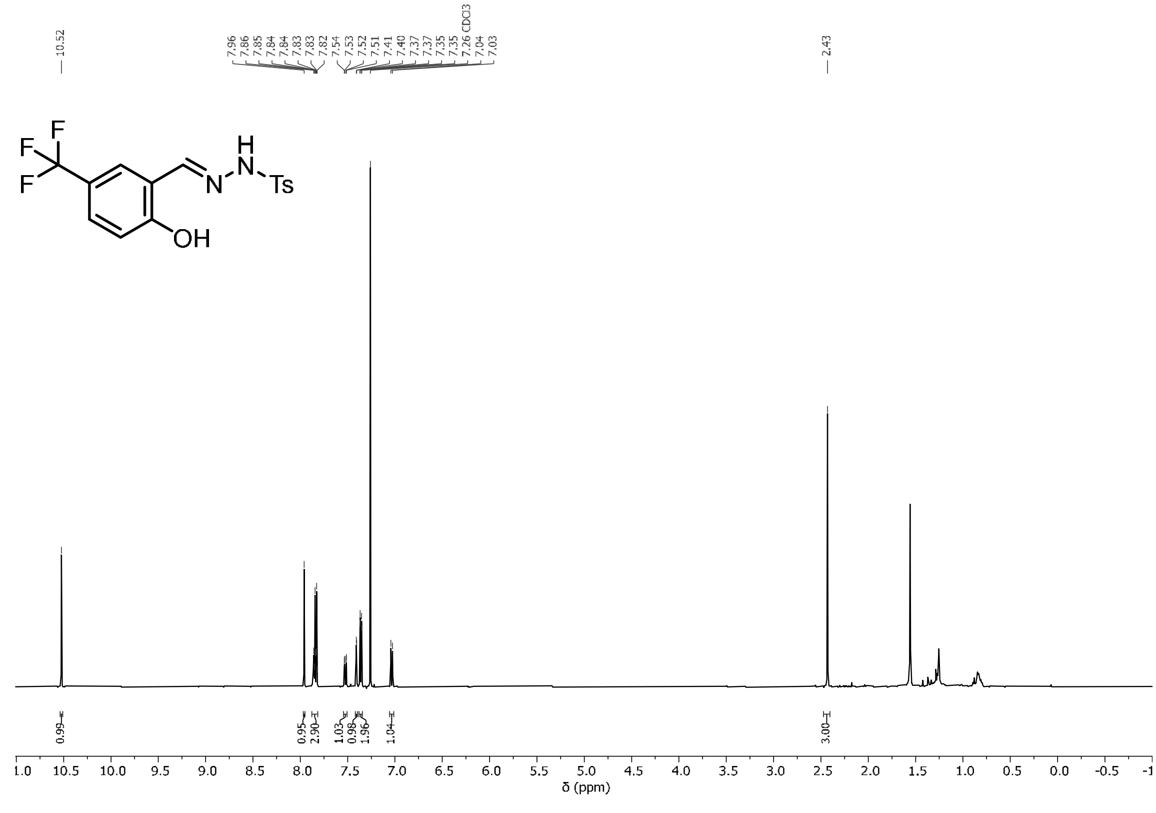
10.52
7.96 7.86 7.85 7.84 7.84 7.83 7.83 7.82 7.54 7.53 7.52 7.51 7.41 7.40 7.37 7.37 7.35 7.35 7.26 CDCl3 7.04 7.03
2.43
F F F H N N Ts OH
0.99 0.95 2.90 1.03 0.98 1.96 1.04 3.00
11.0 10.5 10.0 9.5 9.0 8.5 8.0 7.5 7.0 6.5 6.0 5.5 5.0 4.5 4.0 3.5 3.0 2.5 2.0 1.5 1.0 0.5 0.0 -0.5 -1
δ (ppm)

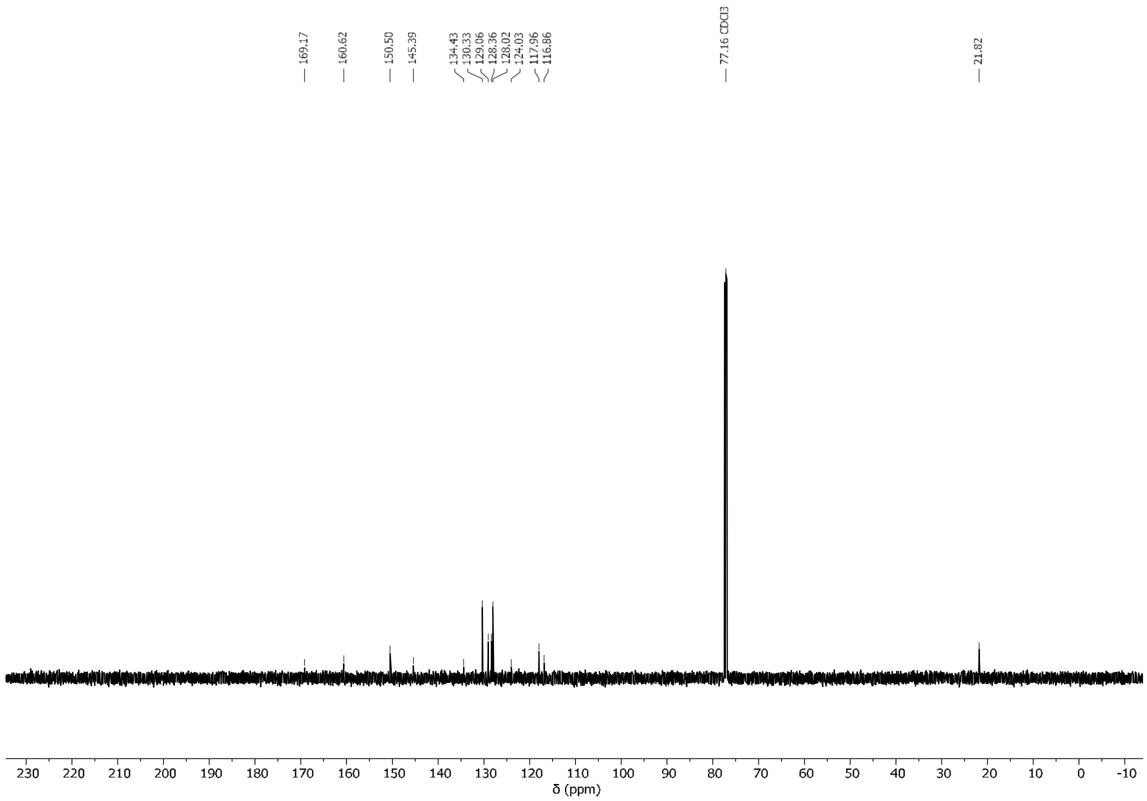
169.17
160.62
150.50
145.39
134.43
130.33
129.06
128.36
128.02
124.03
117.96
116.86
77.16 CDCl3
21.82
δ (ppm)

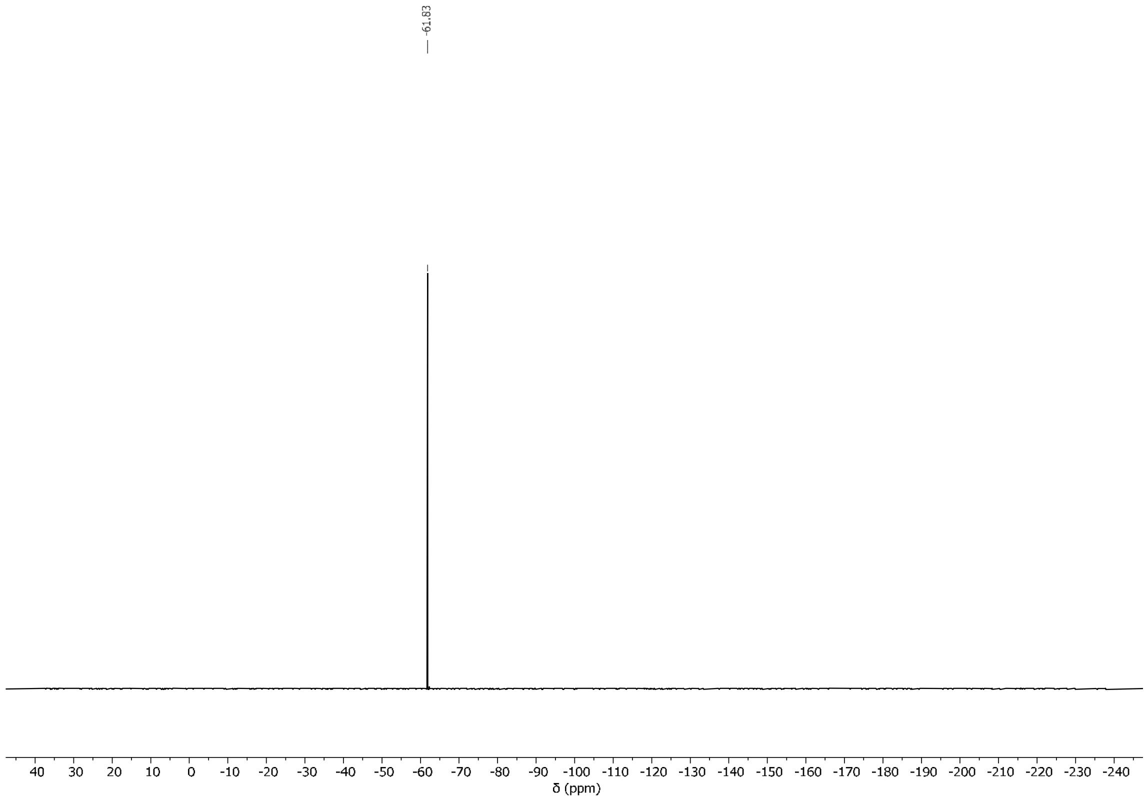
-61.83
δ (ppm)

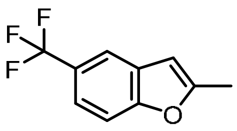
F
F
F
O

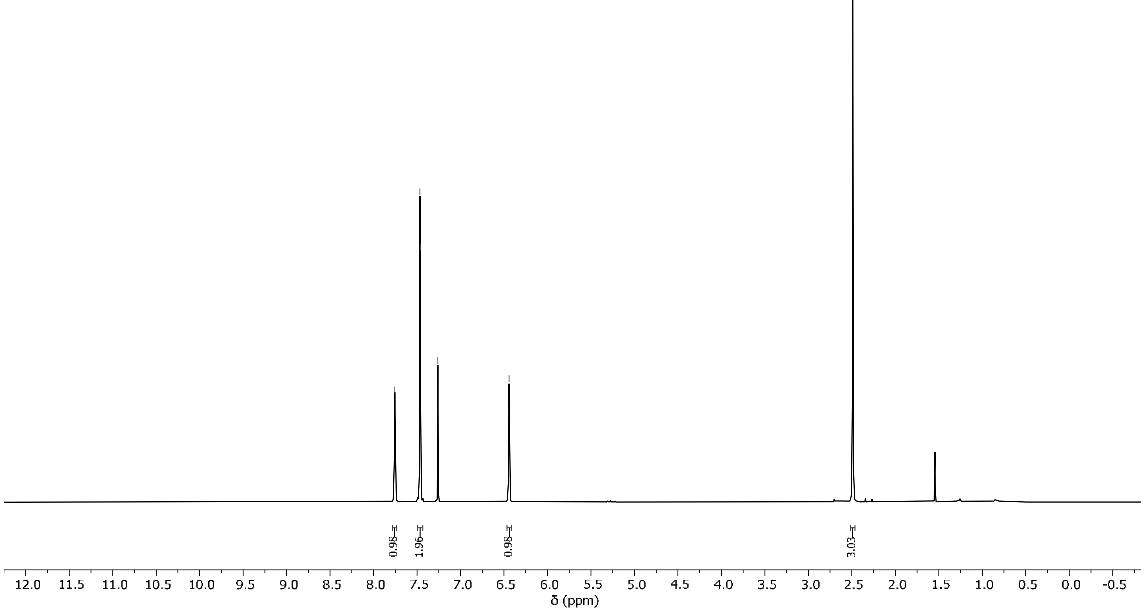
7.76
7.47
7.46
7.26 CDCl3
6.44
2.49
2.49
0.98
1.96
0.98
3.03
12.0 11.5 11.0 10.5 10.0 9.5 9.0 8.5 8.0 7.5 7.0 6.5 6.0 5.5 5.0 4.5 4.0 3.5 3.0 2.5 2.0 1.5 1.0 0.5 0.0 -0.5
δ (ppm)

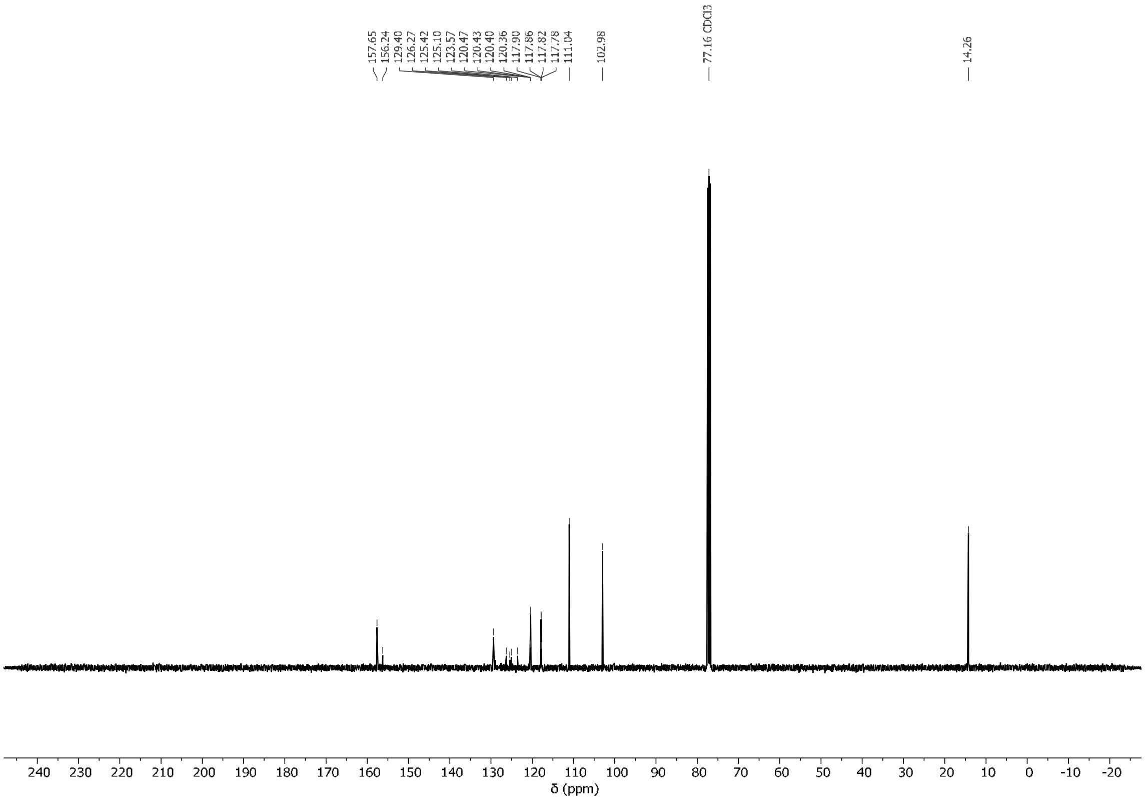
157.65
156.24
129.40
126.27
125.42
125.10
123.57
120.47
120.43
120.40
120.36
117.90
117.86
117.82
117.78
111.04
102.98
77.16 CDCl3
14.26
240 230 220 210 200 190 180 170 160 150 140 130 120 110 100 90 80 70 60 50 40 30 20 10 0 -10 -20
δ (ppm)

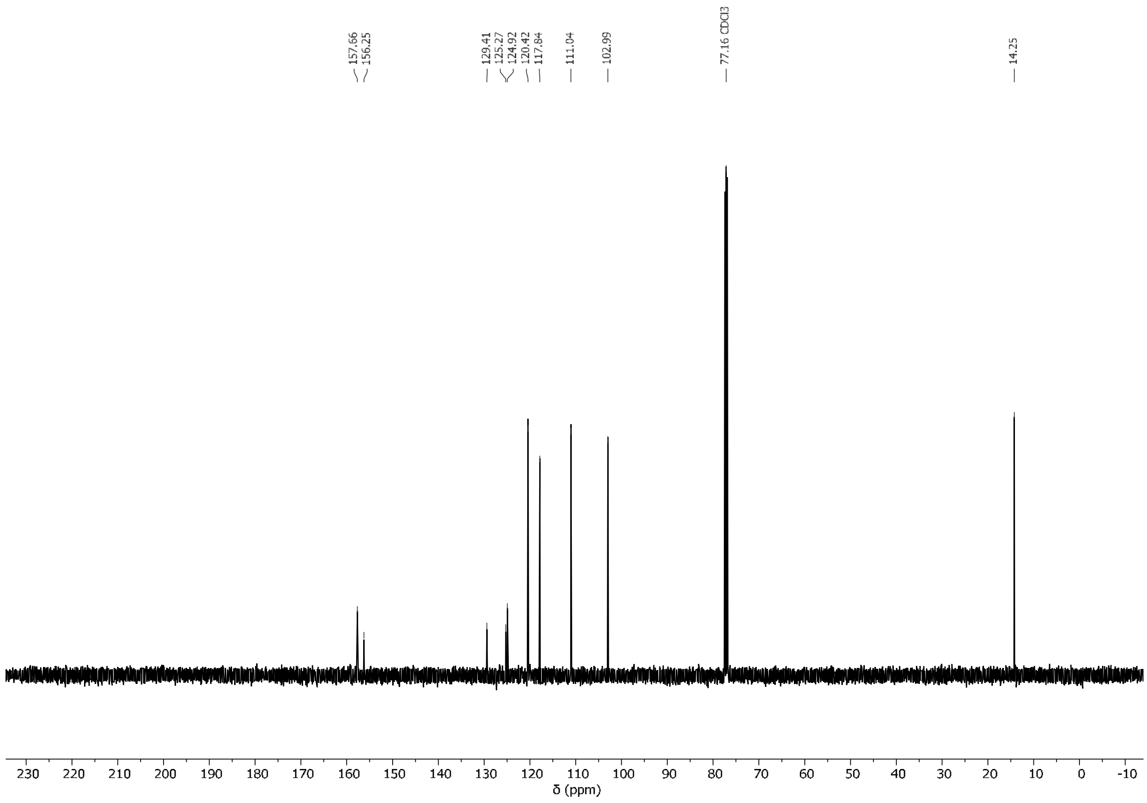

157.66
156.25
129.41
125.27
124.92
120.42
117.84
111.04
102.99
77.16 CDCl3
14.25
230 220 210 200 190 180 170 160 150 140 130 120 110 100 90 80 70 60 50 40 30 20 10 0 -10
δ (ppm)

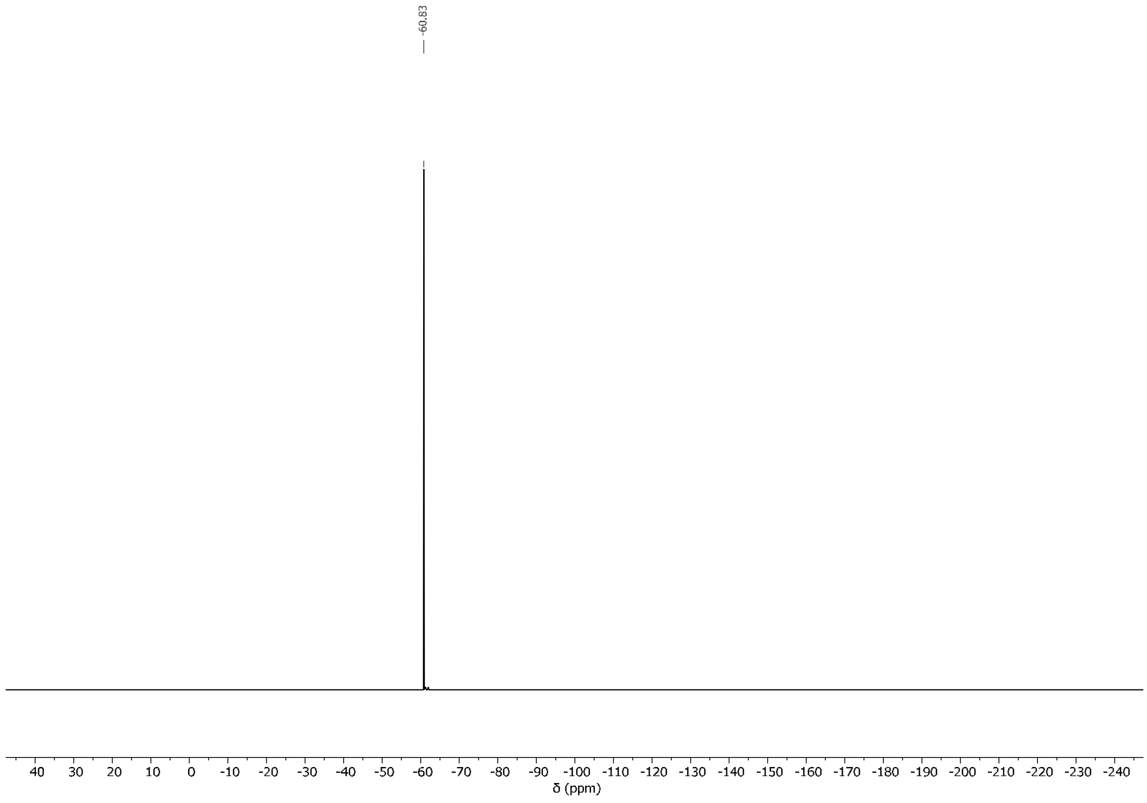

-60.83
40 30 20 10 0 -10 -20 -30 -40 -50 -60 -70 -80 -90 -100 -110 -120 -130 -140 -150 -160 -170 -180 -190 -200 -210 -220 -230 -240
δ (ppm)

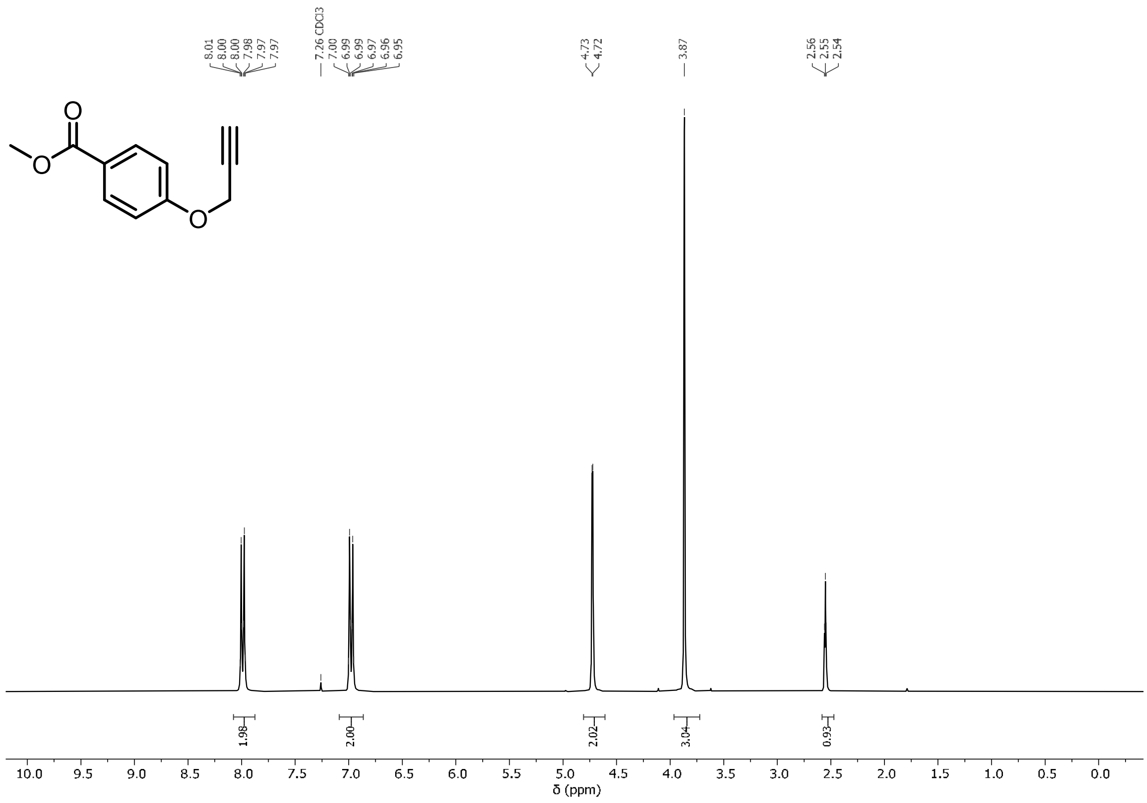
8.01
8.00
8.00
7.98
7.97
7.97
7.26 CDCl3
7.00
6.99
6.99
6.97
6.96
6.95
4.73
4.72
3.87
2.56
2.55
2.54
O
O
O
1.98
2.00
2.02
3.04
0.93
10.0 9.5 9.0 8.5 8.0 7.5 7.0 6.5 6.0 5.5 5.0 4.5 4.0 3.5 3.0 2.5 2.0 1.5 1.0 0.5 0.0
δ (ppm)

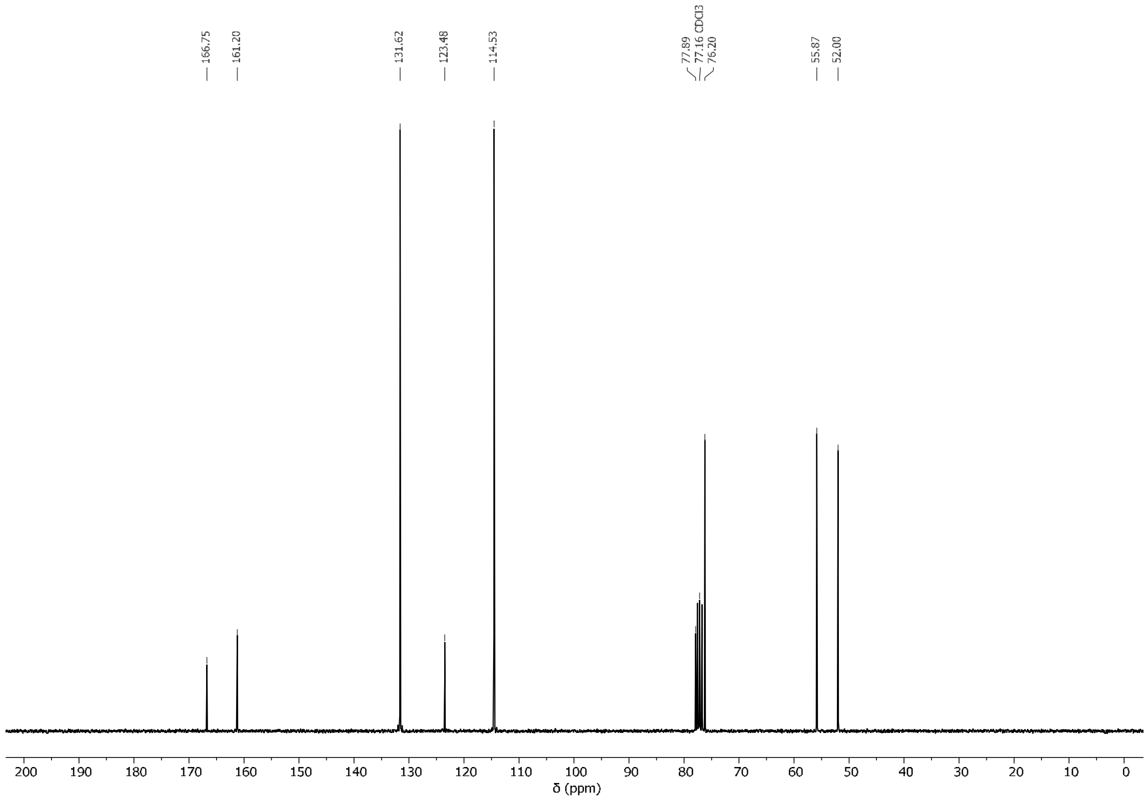
166.75
161.20
131.62
123.48
114.53
77.89
77.16 CDCl3
76.20
55.87
52.00
200 190 180 170 160 150 140 130 120 110 100 90 80 70 60 50 40 30 20 10 0
δ (ppm)

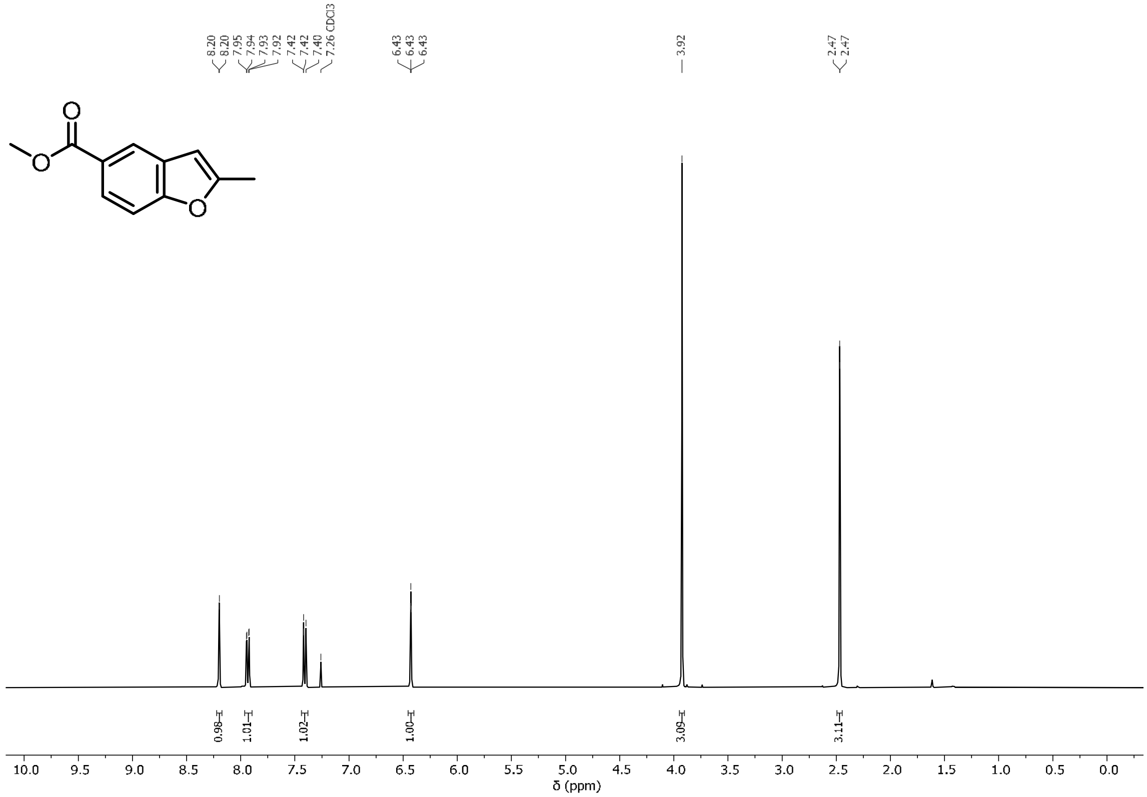
8.20
8.20
7.95
7.94
7.93
7.92
7.42
7.42
7.40
7.26 CDCl3
6.43
6.43
6.43
3.92
2.47
2.47
0.98
1.01
1.02
1.00
3.09
3.11
δ (ppm)

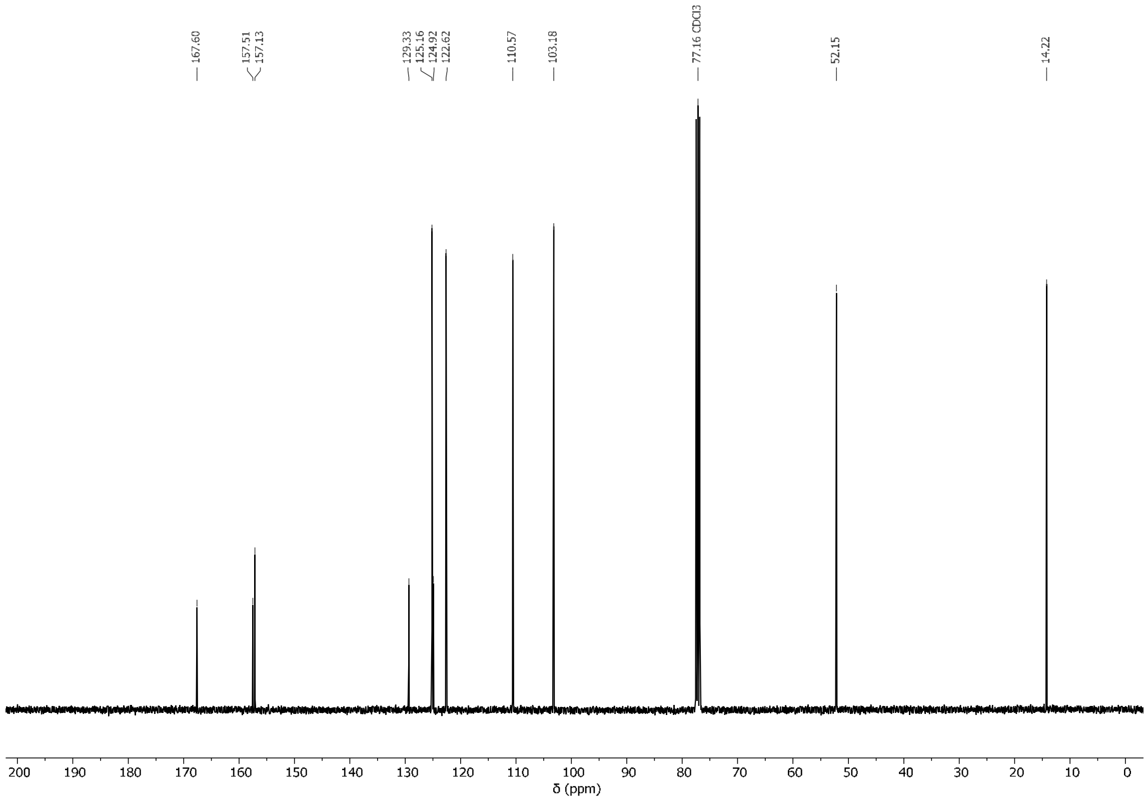
167.60
157.51
157.13
129.33
125.16
124.92
122.62
110.57
103.18
77.16 CDCl3
52.15
14.22
δ (ppm)

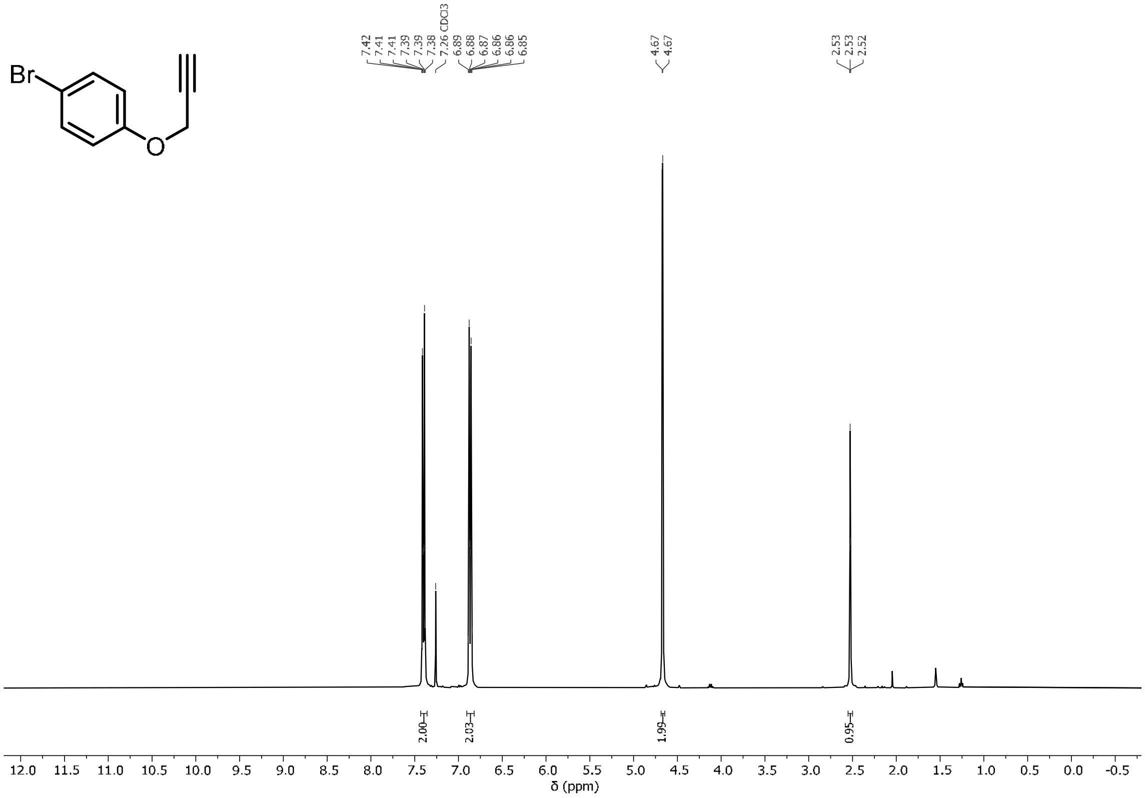
Br
O
7.42
7.41
7.41
7.39
7.39
7.38
7.26 CDCl3
6.89
6.88
6.87
6.86
6.86
6.85
4.67
4.67
2.53
2.53
2.52
2.00
2.03
1.99
0.95
12.0 11.5 11.0 10.5 10.0 9.5 9.0 8.5 8.0 7.5 7.0 6.5 6.0 5.5 5.0 4.5 4.0 3.5 3.0 2.5 2.0 1.5 1.0 0.5 0.0 -0.5
δ (ppm)

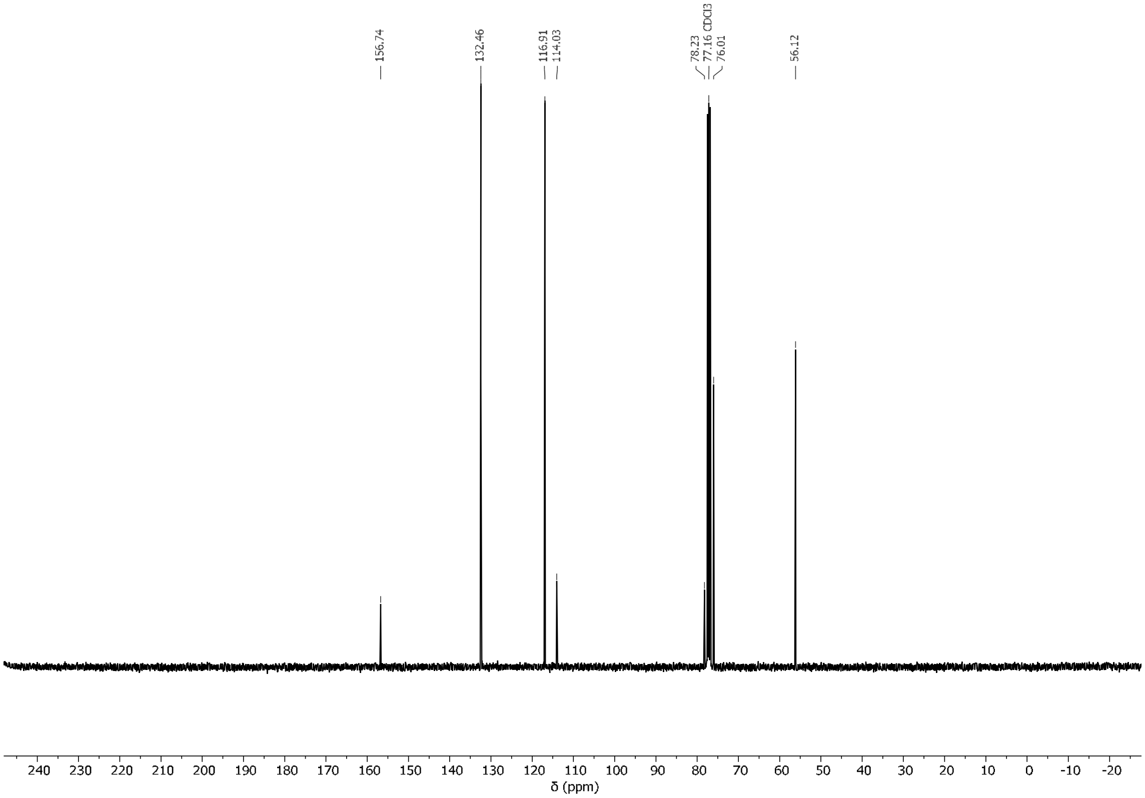
156.74
132.46
116.91
114.03
78.23
77.16 CDCl3
76.01
56.12
240 230 220 210 200 190 180 170 160 150 140 130 120 110 100 90 80 70 60 50 40 30 20 10 0 -10 -20
δ (ppm)

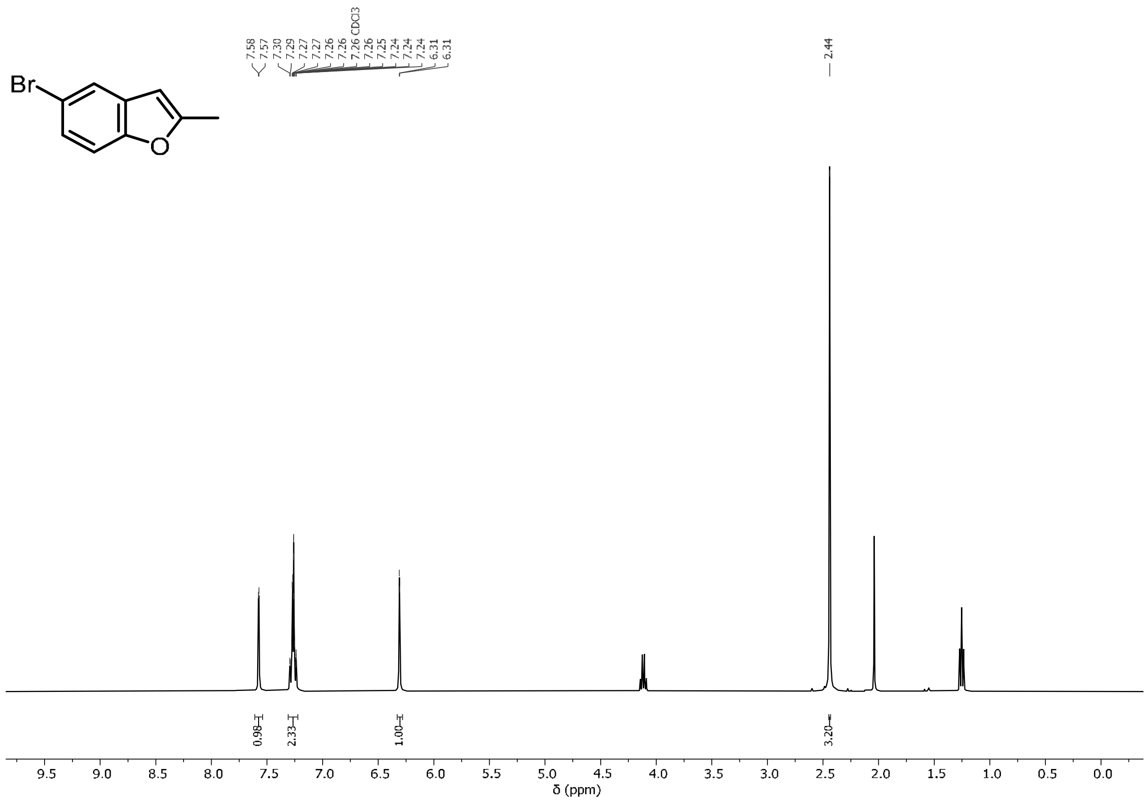
Br
O
7.58
7.57
7.30
7.29
7.27
7.27
7.26
7.26
7.26 CDCl3
7.26
7.25
7.24
7.24
7.24
7.24
6.31
6.31
2.44
0.98
2.33
1.00
3.20
9.5 9.0 8.5 8.0 7.5 7.0 6.5 6.0 5.5 5.0 4.5 4.0 3.5 3.0 2.5 2.0 1.5 1.0 0.5 0.0
δ (ppm)

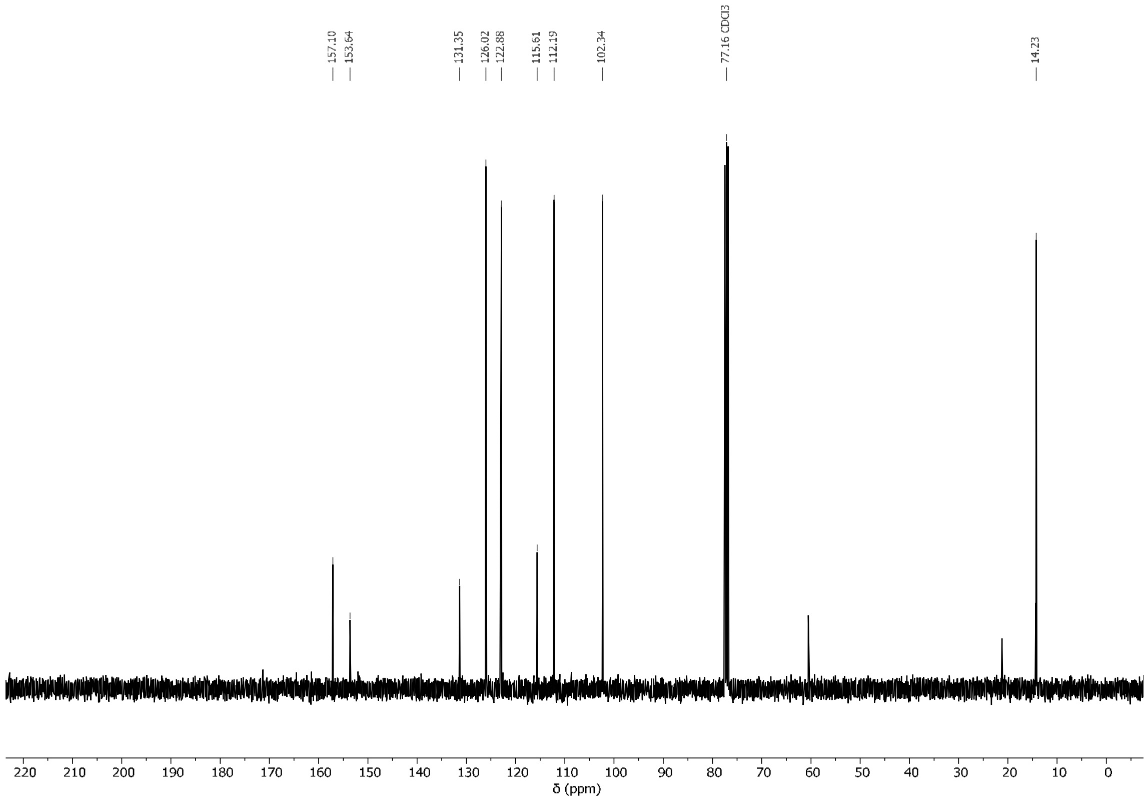
157.10
153.64
131.35
126.02
122.88
115.61
112.19
102.34
77.16 CDCl3
14.23
220 210 200 190 180 170 160 150 140 130 120 110 100 90 80 70 60 50 40 30 20 10 0
δ (ppm)

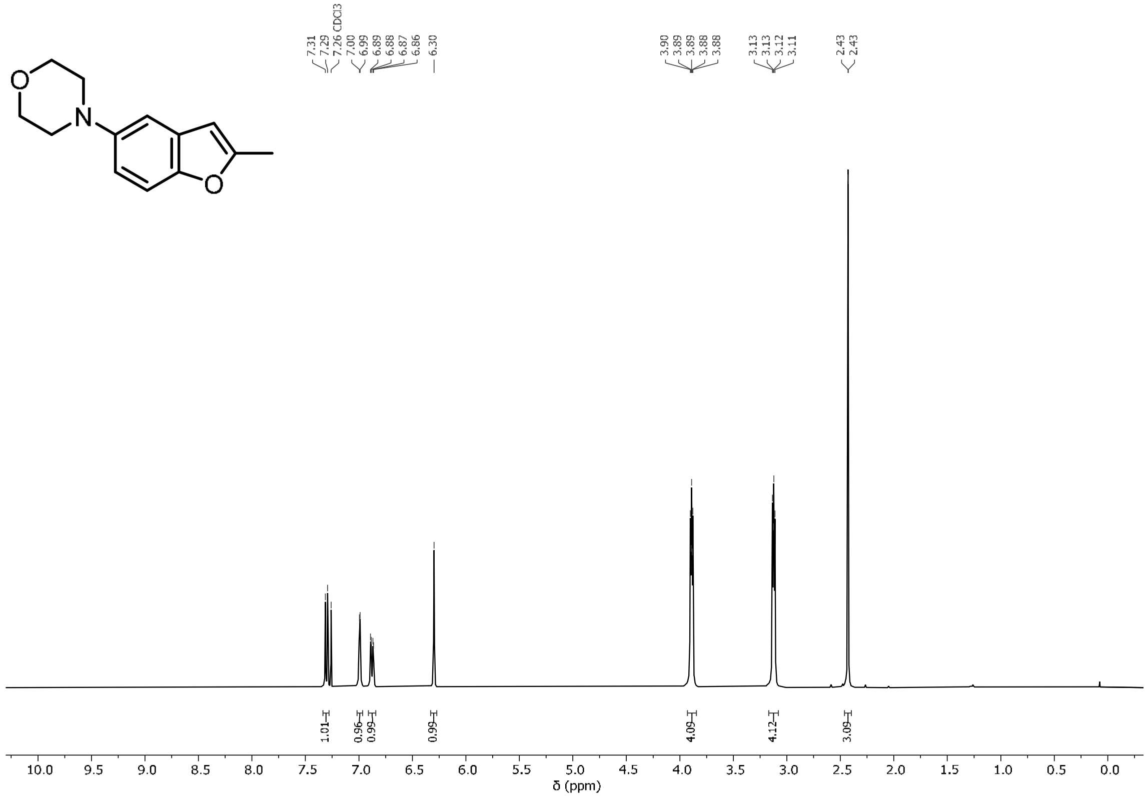

O
N
O
7.31
7.29
7.26 CDCl3
7.00
6.99
6.89
6.88
6.87
6.86
6.30
3.90
3.89
3.89
3.88
3.88
3.13
3.13
3.12
3.11
2.43
2.43
1.01
0.96
0.99
0.99
4.09
4.12
3.09
10.0 9.5 9.0 8.5 8.0 7.5 7.0 6.5 6.0 5.5 5.0 4.5 4.0 3.5 3.0 2.5 2.0 1.5 1.0 0.5 0.0
δ (ppm)

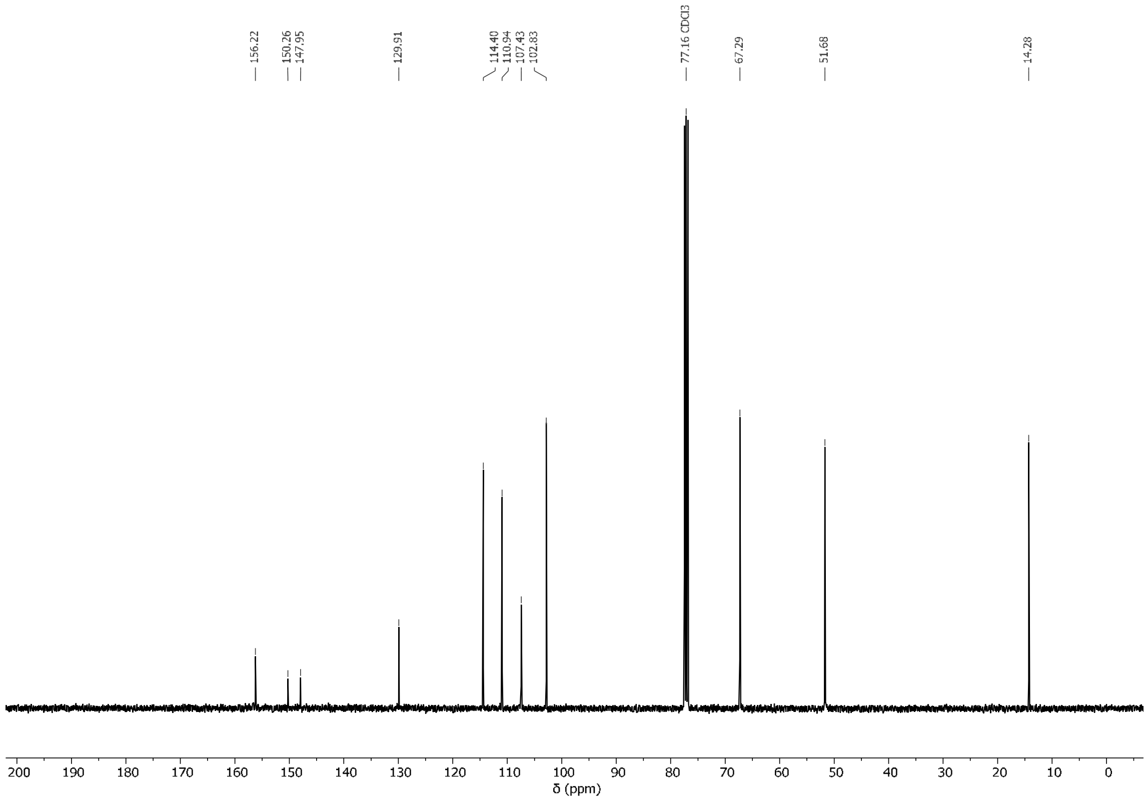

156.22
150.26
147.95
129.91
114.40
110.94
107.43
102.83
77.16 CDCl3
67.29
51.68
14.28
200 190 180 170 160 150 140 130 120 110 100 90 80 70 60 50 40 30 20 10 0
δ (ppm)

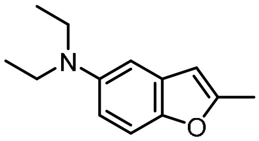

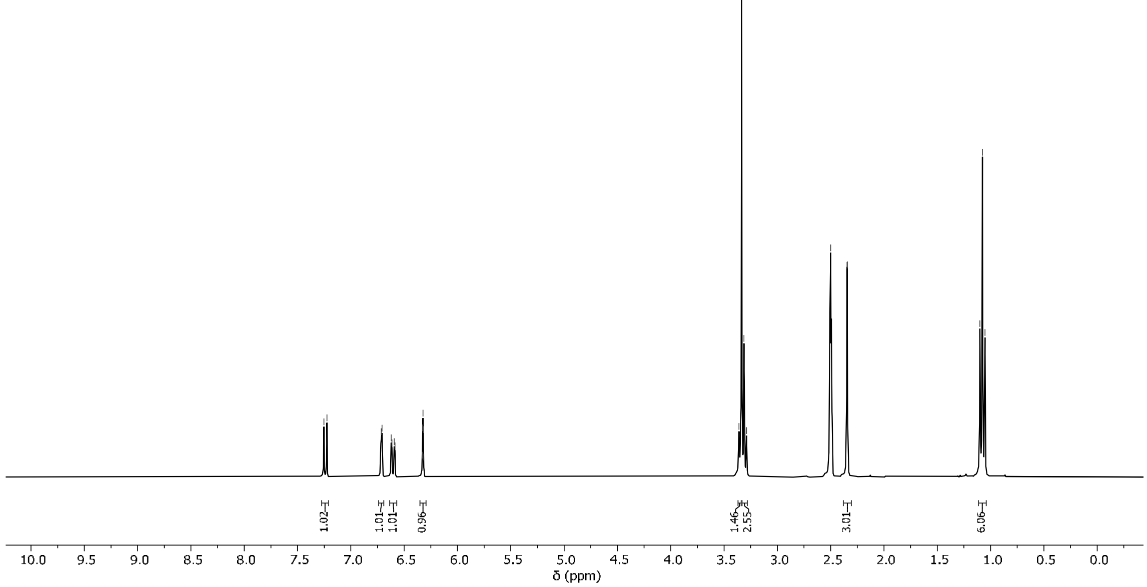
7.25
7.22
6.72
6.71
6.62
6.61
6.59
6.59
6.33
6.33
6.32
6.32
6.32
3.36
3.34
3.31
3.29
2.50 DMSO-d6
2.35
2.34
1.10
1.08
1.05
1.02
1.01
1.01
0.96
1.46
2.55
3.01
6.06
δ (ppm)

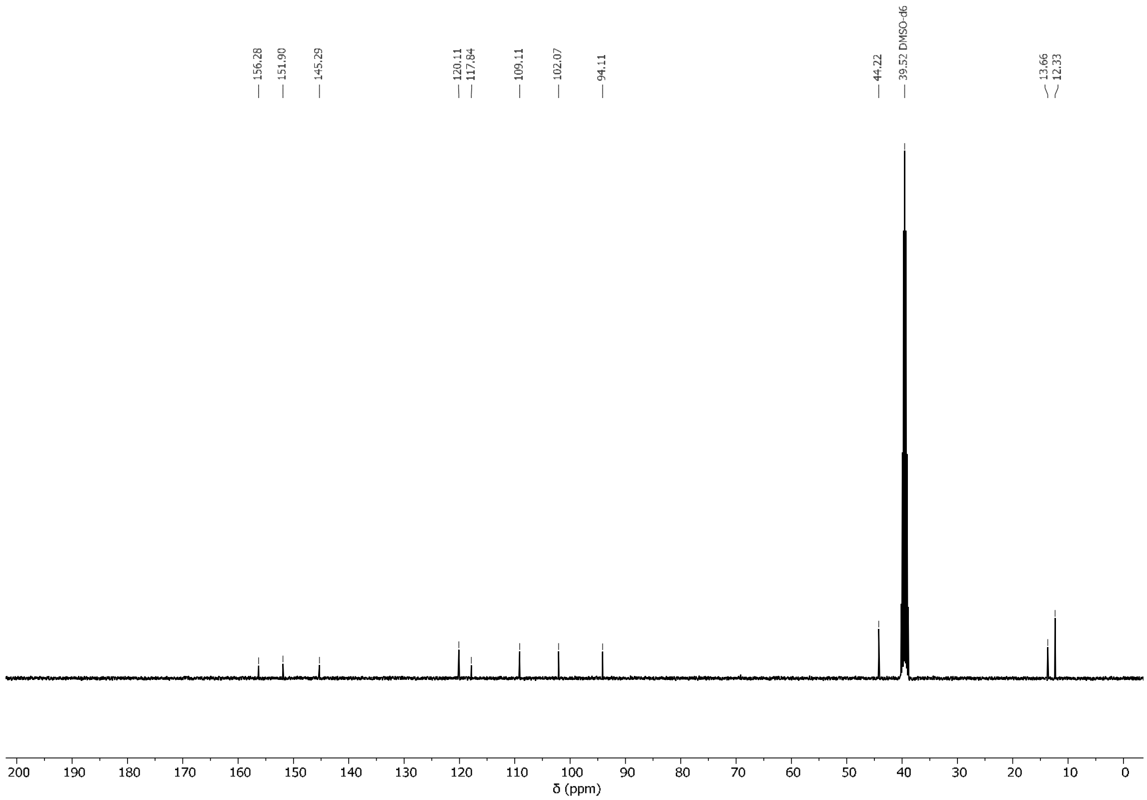
156.28
151.90
145.29
120.11
117.84
109.11
102.07
94.11
44.22
39.52 DMSO-d6
13.66
12.33
δ (ppm)

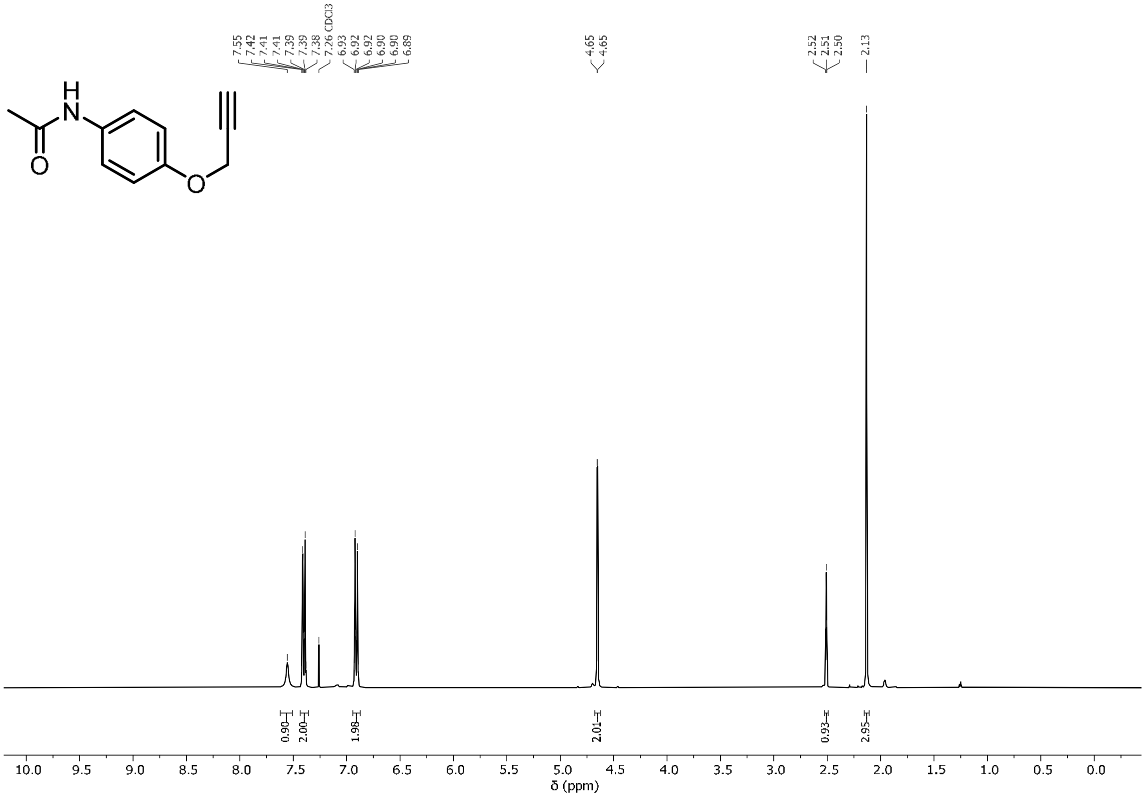
7.55
7.42
7.41
7.41
7.39
7.39
7.38
7.26 CDCl3
6.93
6.92
6.92
6.90
6.90
6.89
4.65
4.65
2.52
2.51
2.50
2.13
H
N
O
O
0.90
2.00
1.98
2.01
0.93
2.95
10.0 9.5 9.0 8.5 8.0 7.5 7.0 6.5 6.0 5.5 5.0 4.5 4.0 3.5 3.0 2.5 2.0 1.5 1.0 0.5 0.0
δ (ppm)

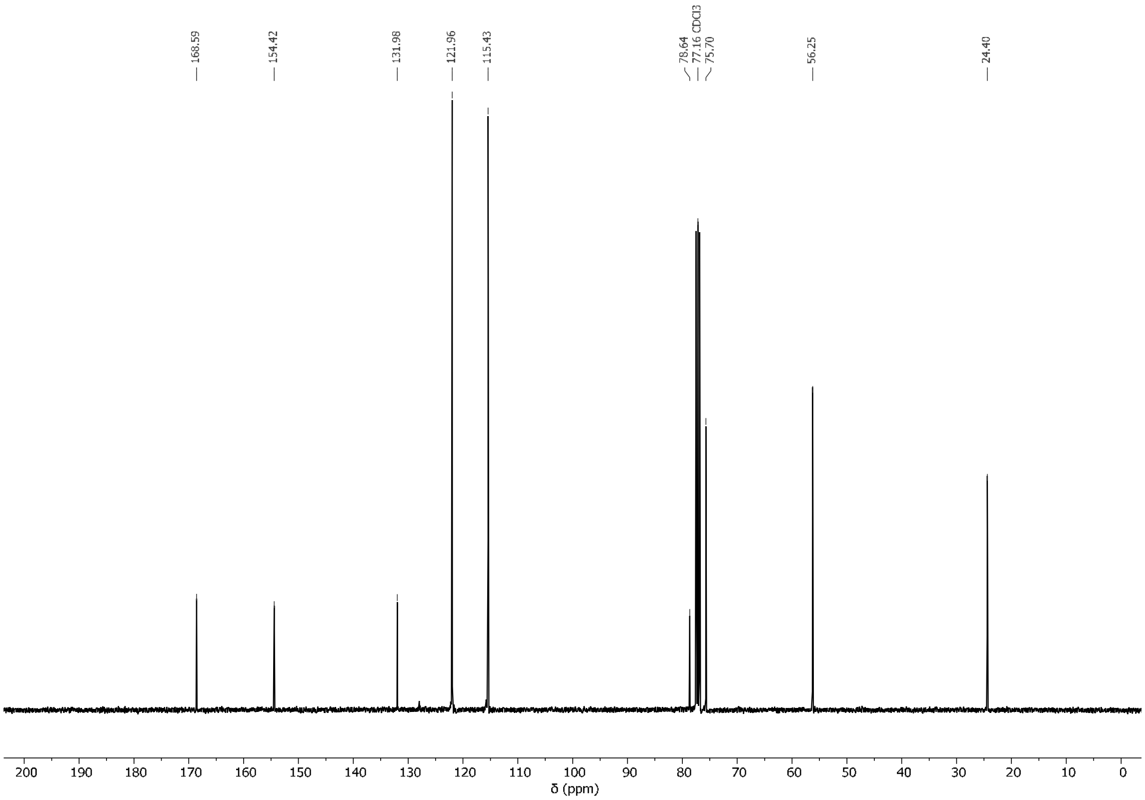
168.59
154.42
131.98
121.96
115.43
78.64
77.16 CDCl3
75.70
56.25
24.40
200 190 180 170 160 150 140 130 120 110 100 90 80 70 60 50 40 30 20 10 0
δ (ppm)

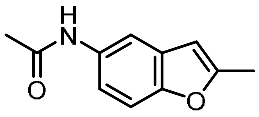

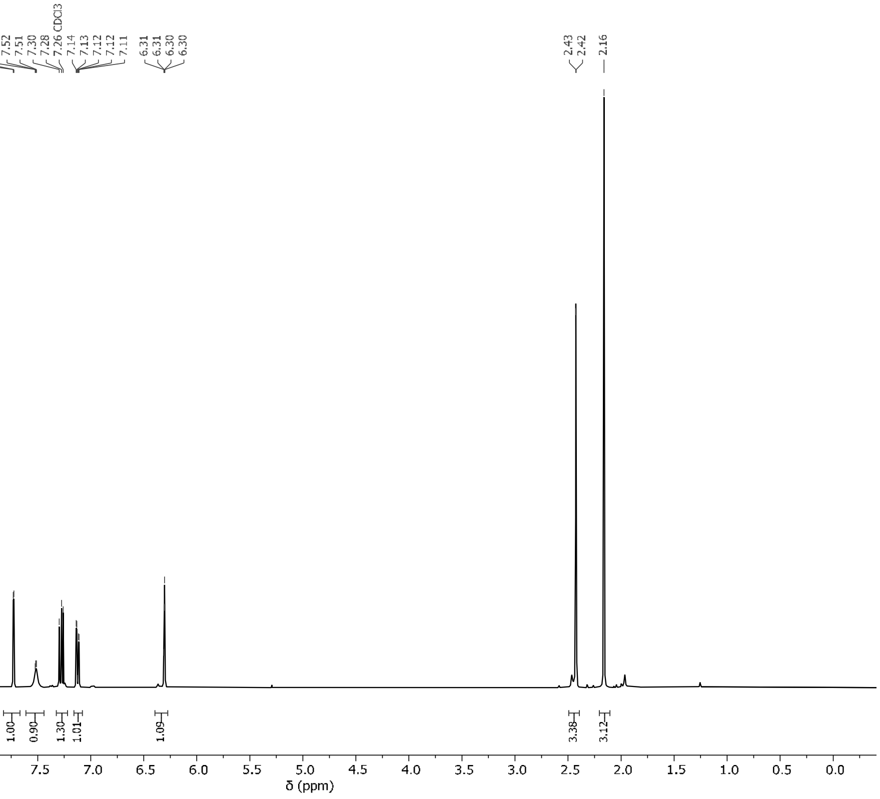
7.73
7.72
7.52
7.52
7.51
7.30
7.28
7.26 CDCl3
7.14
7.13
7.12
7.12
7.11
6.31
6.31
6.30
6.30
2.43
2.42
2.16
1.00
0.90
1.30
1.01
1.09
3.38
3.12
δ (ppm)

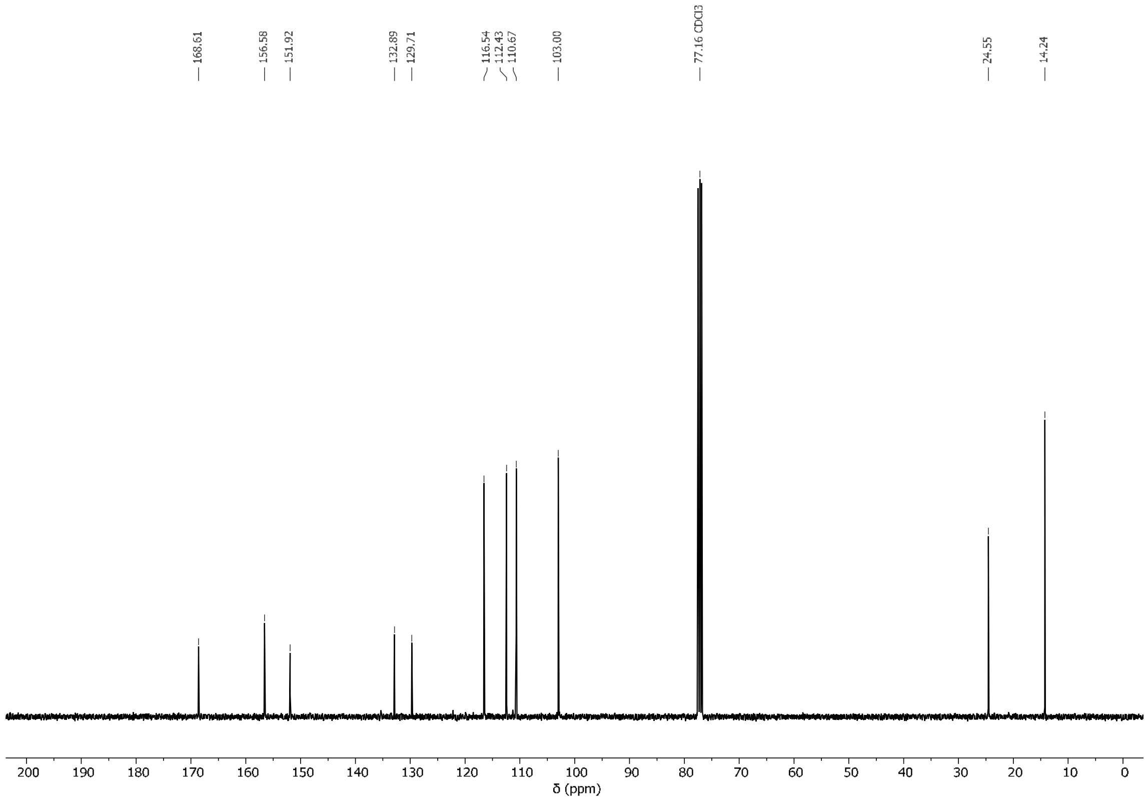
168.61
156.58
151.92
132.89
129.71
116.54
112.43
110.67
103.00
77.16 CDCl3
24.55
14.24
δ (ppm)

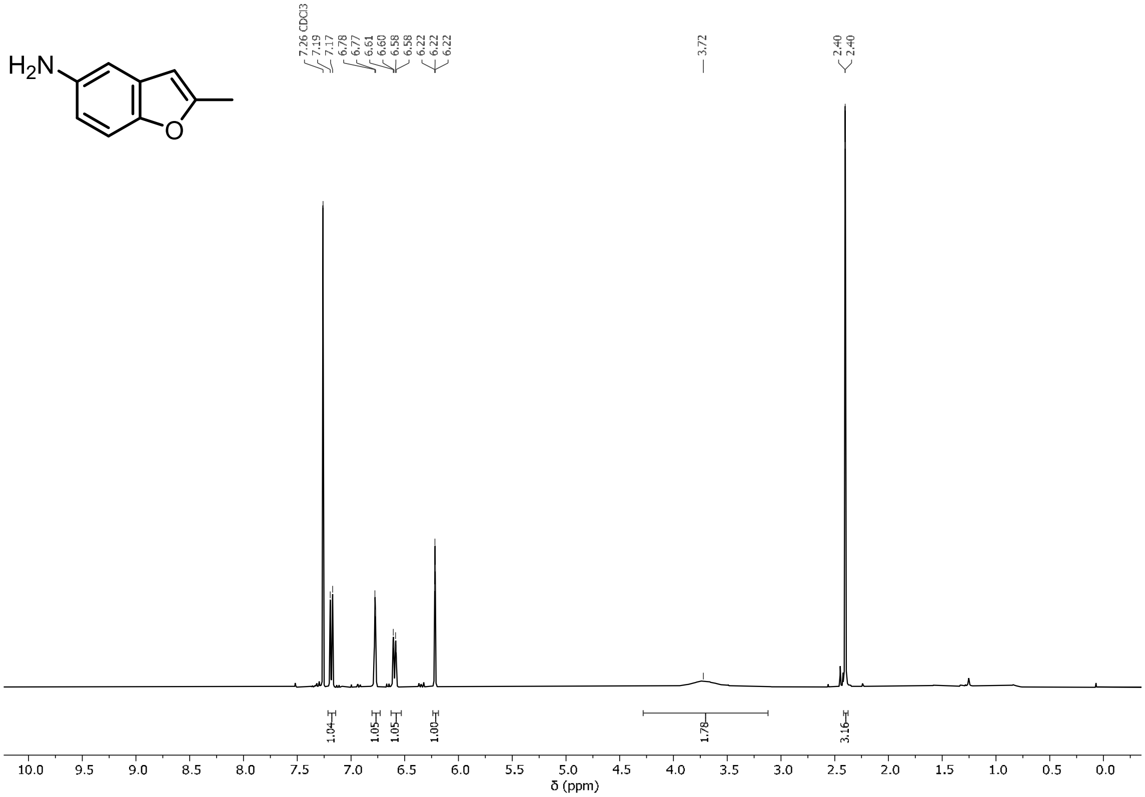
H2N
O
7.26 CDCl3
7.19
7.17
6.78
6.77
6.61
6.60
6.58
6.58
6.22
6.22
6.22
3.72
2.40
2.40
1.04
1.05
1.05
1.00
1.78
3.16
10.0 9.5 9.0 8.5 8.0 7.5 7.0 6.5 6.0 5.5 5.0 4.5 4.0 3.5 3.0 2.5 2.0 1.5 1.0 0.5 0.0
δ (ppm)

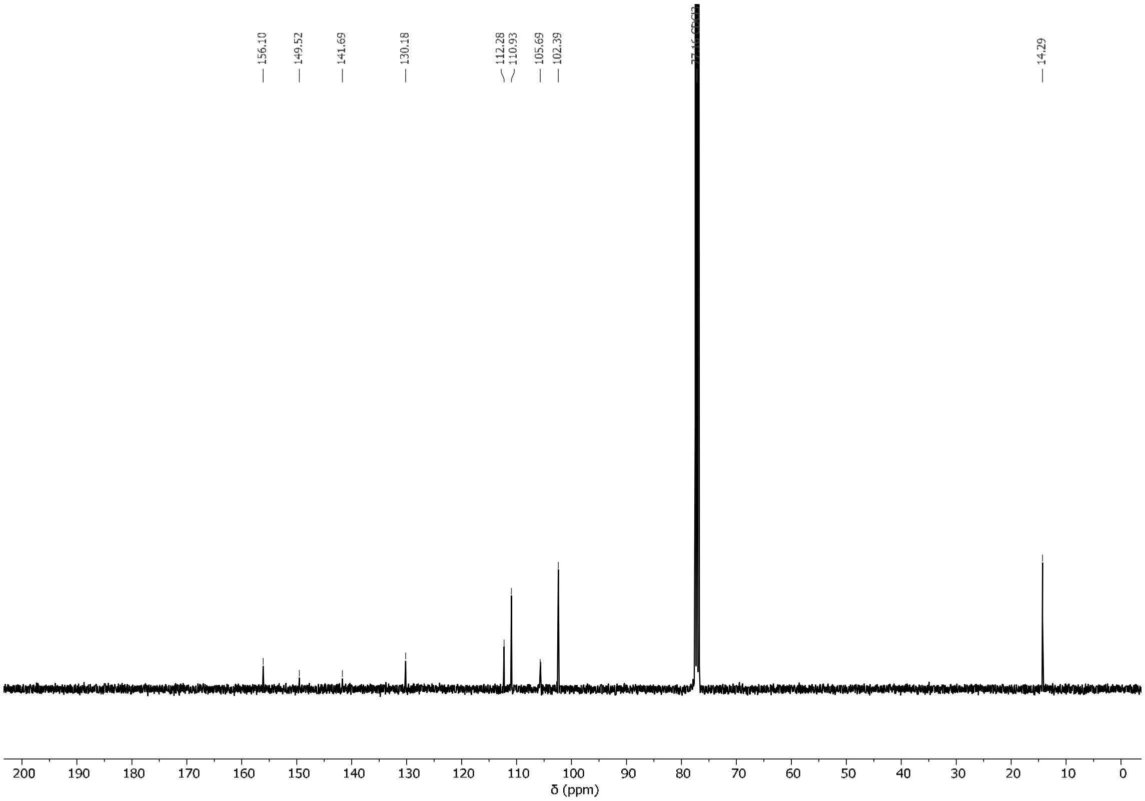
156.10
149.52
141.69
130.18
112.28
110.93
105.69
102.39
14.29
200 190 180 170 160 150 140 130 120 110 100 90 80 70 60 50 40 30 20 10 0
δ (ppm)

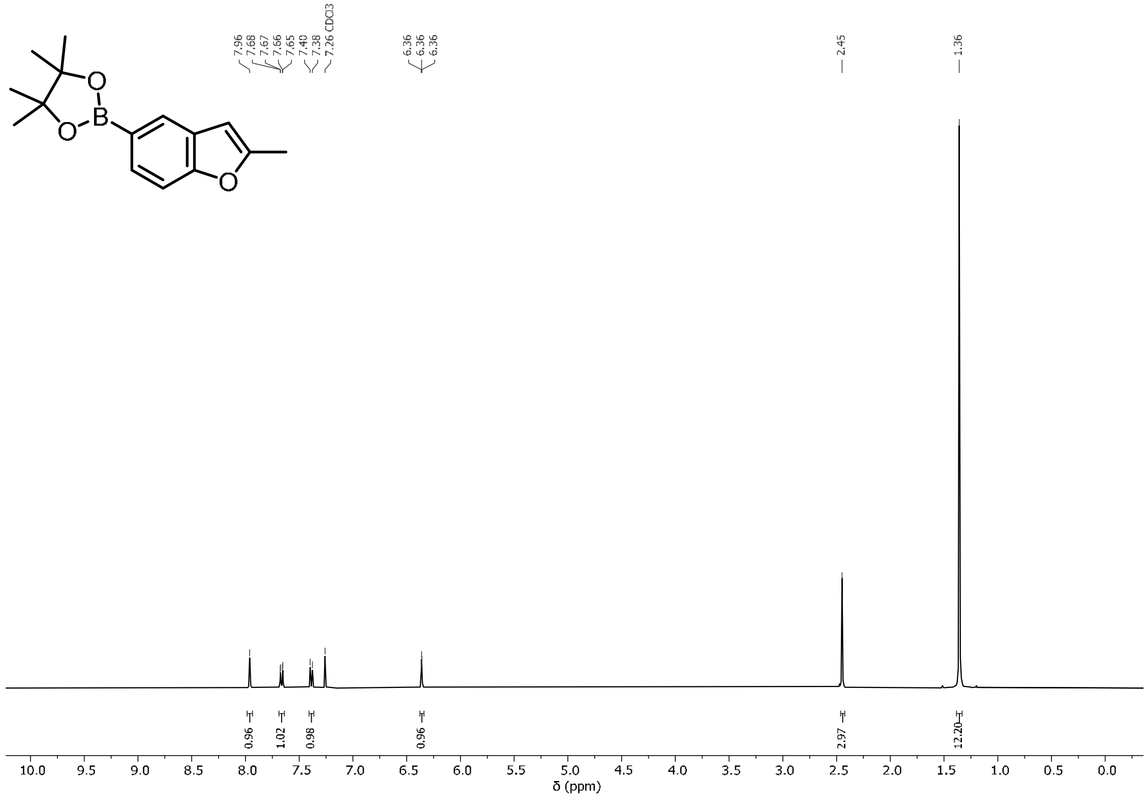
7.96
7.68
7.67
7.66
7.65
7.40
7.38
7.26 CDCl3
6.36
6.36
6.36
2.45
1.36
0.96
1.02
0.98
0.96
2.97
12.20
δ (ppm)

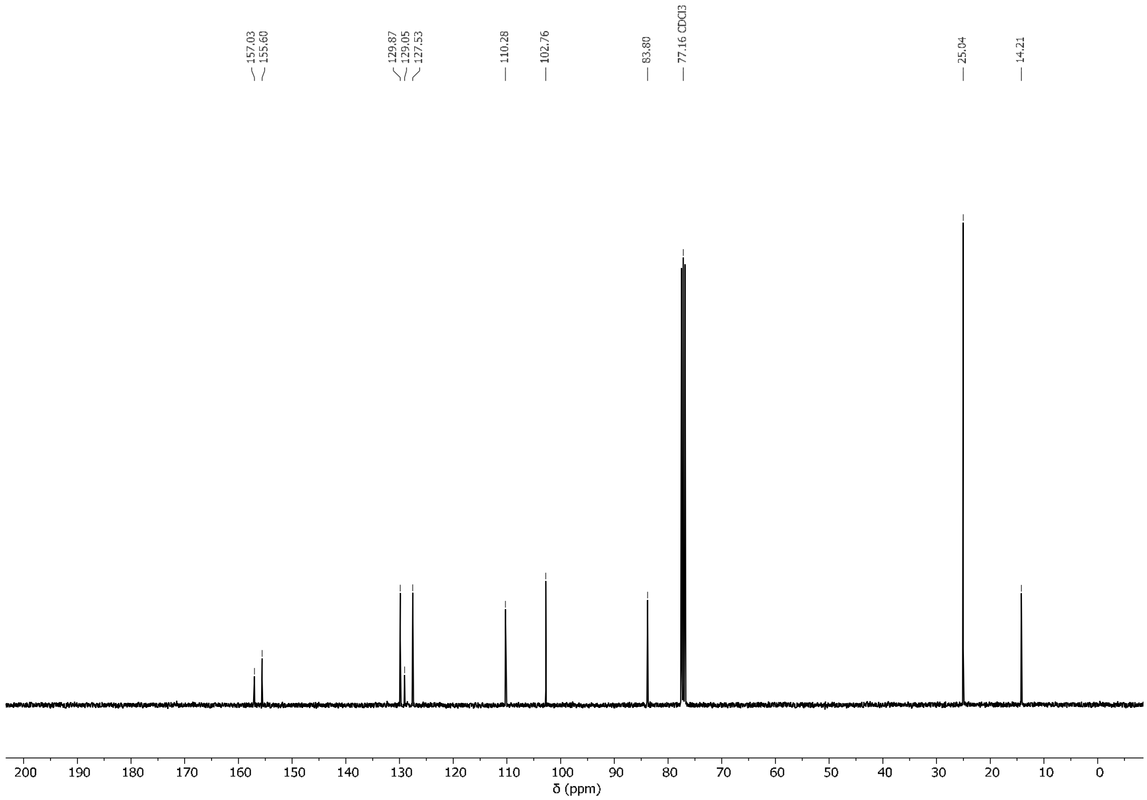
157.03
155.60
129.87
129.05
127.53
110.28
102.76
83.80
77.16 CDCl3
25.04
14.21
δ (ppm)

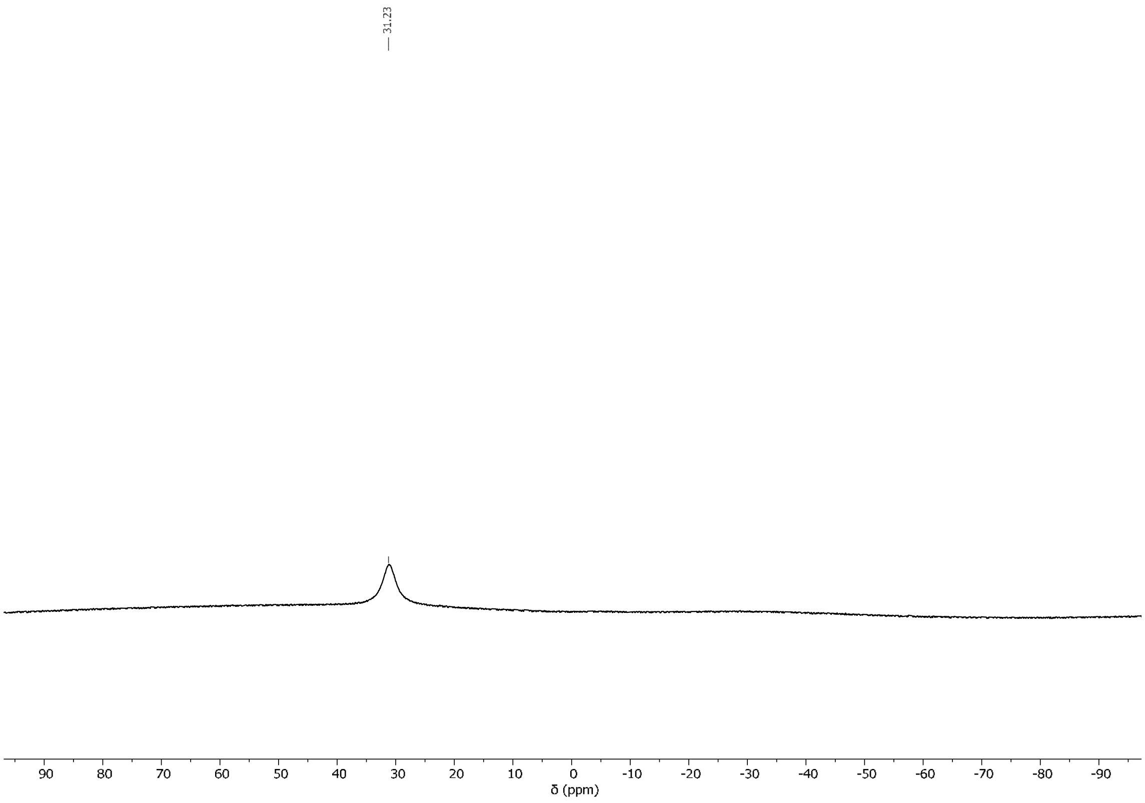

31.23
90 80 70 60 50 40 30 20 10 0 -10 -20 -30 -40 -50 -60 -70 -80 -90
δ (ppm)

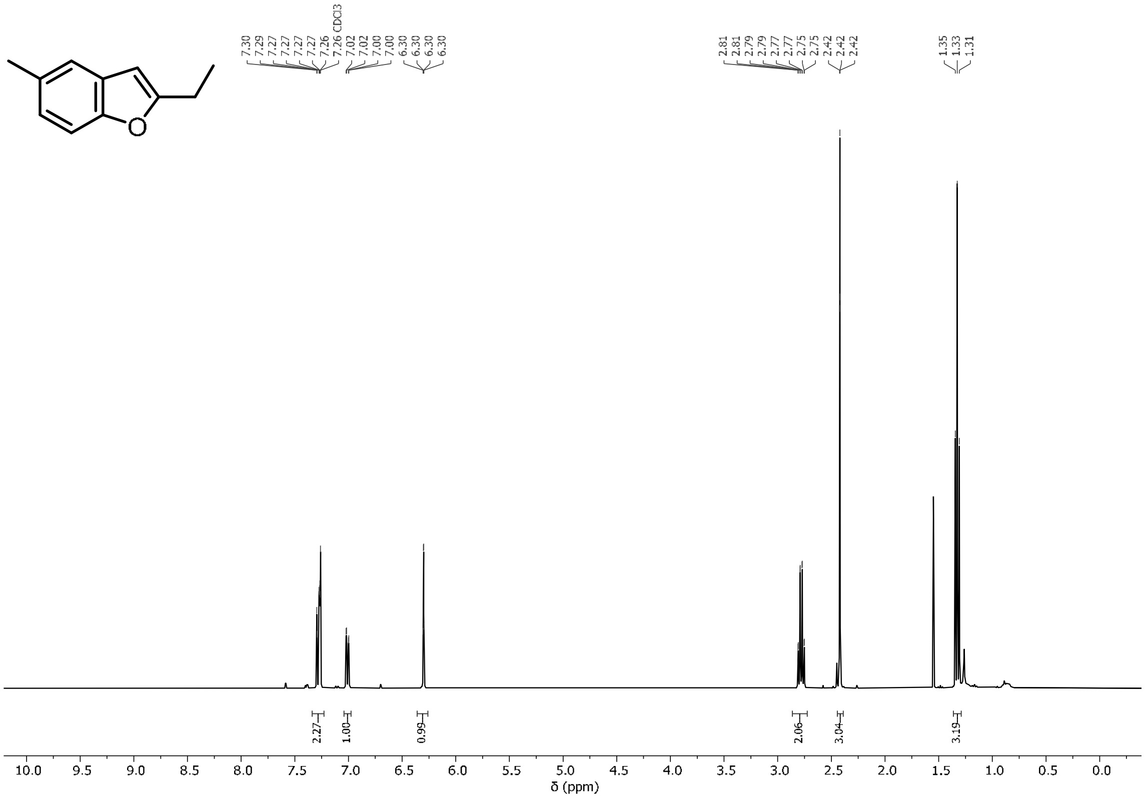

7.30 7.29 7.27 7.27 7.27 7.27 7.26 7.26 CDCl3 7.02 7.02 7.00 7.00 6.30 6.30 6.30 6.30
2.81 2.81 2.79 2.79 2.77 2.77 2.75 2.75 2.42 2.42 2.42
1.35 1.33 1.31
2.27 1.00 0.99 2.06 3.04 3.19
10.0 9.5 9.0 8.5 8.0 7.5 7.0 6.5 6.0 5.5 5.0 4.5 4.0 3.5 3.0 2.5 2.0 1.5 1.0 0.5 0.0
δ (ppm)

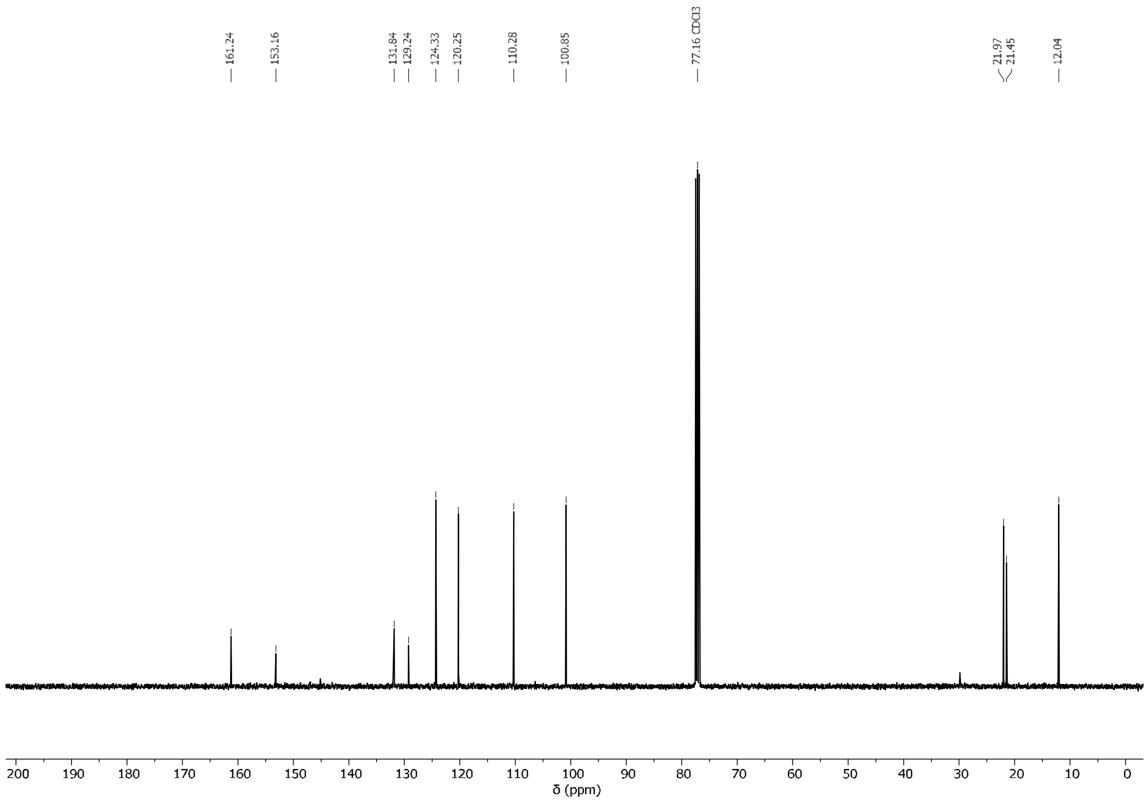
161.24
153.16
131.84
129.24
124.33
120.25
110.28
100.85
77.16 CDCl3
21.97
21.45
12.04
200 190 180 170 160 150 140 130 120 110 100 90 80 70 60 50 40 30 20 10 0
δ (ppm)

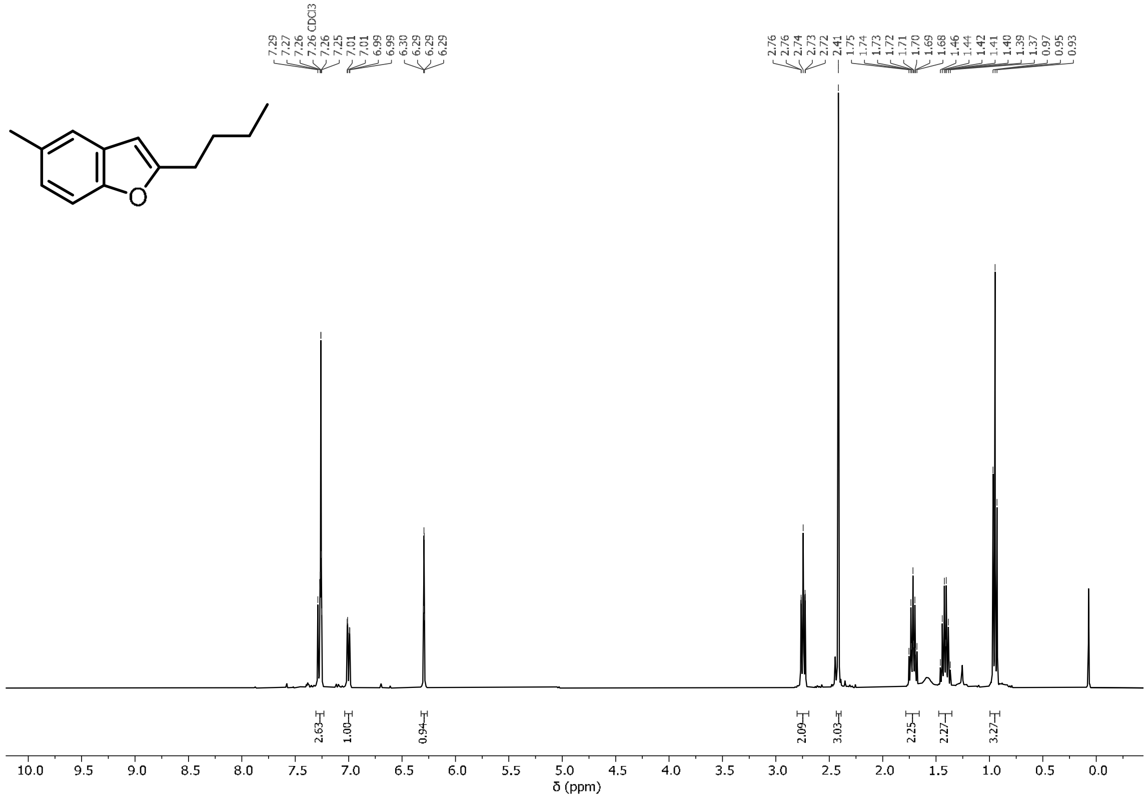
7.29
7.27
7.26
7.26 CDCl3
7.26
7.25
7.01
7.01
6.99
6.99
6.30
6.29
6.29
6.29
2.76
2.76
2.74
2.73
2.72
2.41
1.75
1.74
1.73
1.72
1.71
1.70
1.69
1.68
1.46
1.44
1.42
1.41
1.40
1.39
1.37
0.97
0.95
0.93
O
2.63
1.00
0.94
2.09
3.03
2.25
2.27
3.27
10.0 9.5 9.0 8.5 8.0 7.5 7.0 6.5 6.0 5.5 5.0 4.5 4.0 3.5 3.0 2.5 2.0 1.5 1.0 0.5 0.0
δ (ppm)

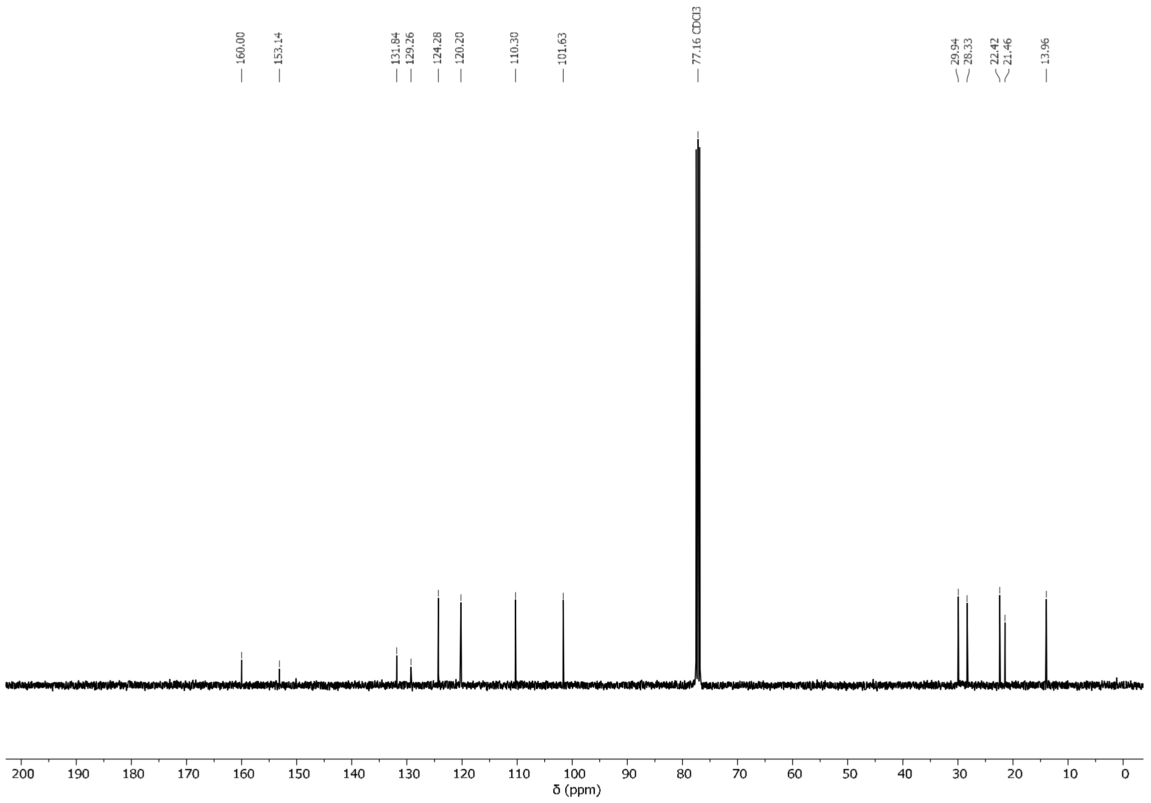
160.00
153.14
131.84
129.26
124.28
120.20
110.30
101.63
77.16 CDCl3
29.94
28.33
22.42
21.46
13.96
200 190 180 170 160 150 140 130 120 110 100 90 80 70 60 50 40 30 20 10 0
δ (ppm)

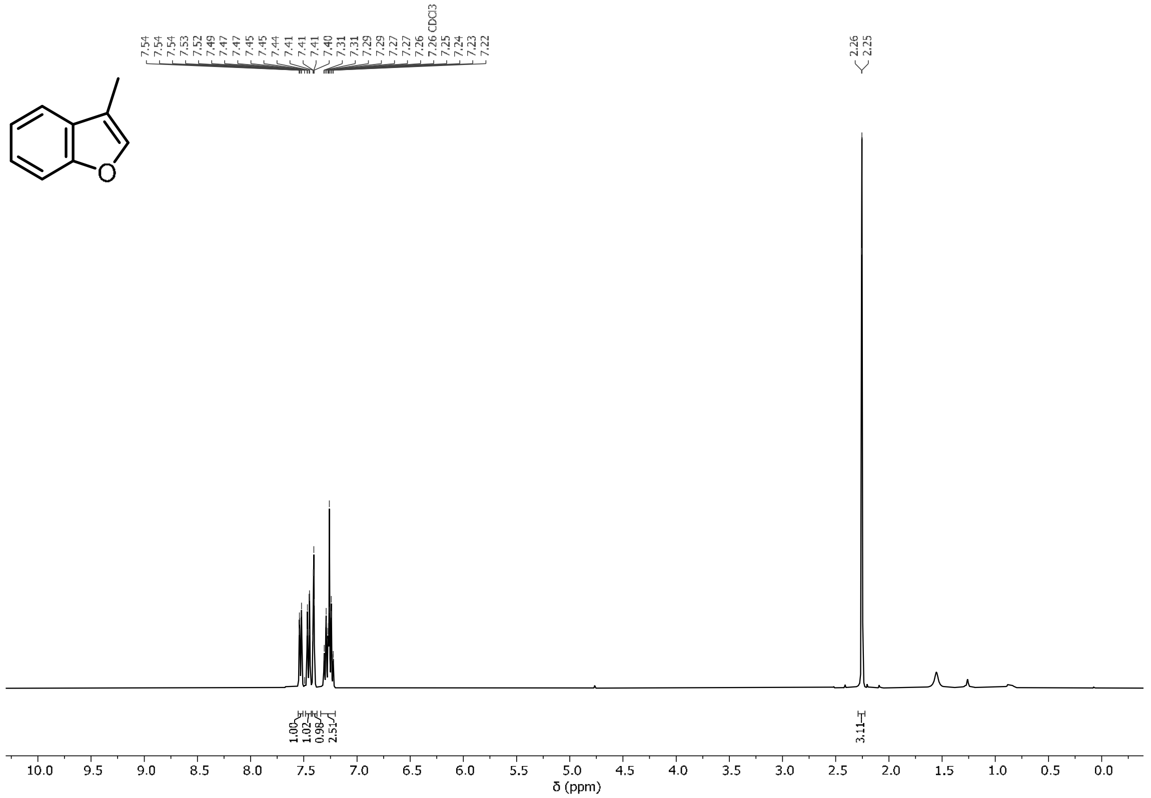
O
7.54
7.54
7.53
7.52
7.49
7.47
7.47
7.45
7.45
7.44
7.41
7.41
7.41
7.40
7.31
7.31
7.29
7.29
7.27
7.27
7.26 CDCl3
7.25
7.24
7.23
7.22
2.26
2.25
1.00
1.02
0.98
2.51
3.11
10.0 9.5 9.0 8.5 8.0 7.5 7.0 6.5 6.0 5.5 5.0 4.5 4.0 3.5 3.0 2.5 2.0 1.5 1.0 0.5 0.0
δ (ppm)

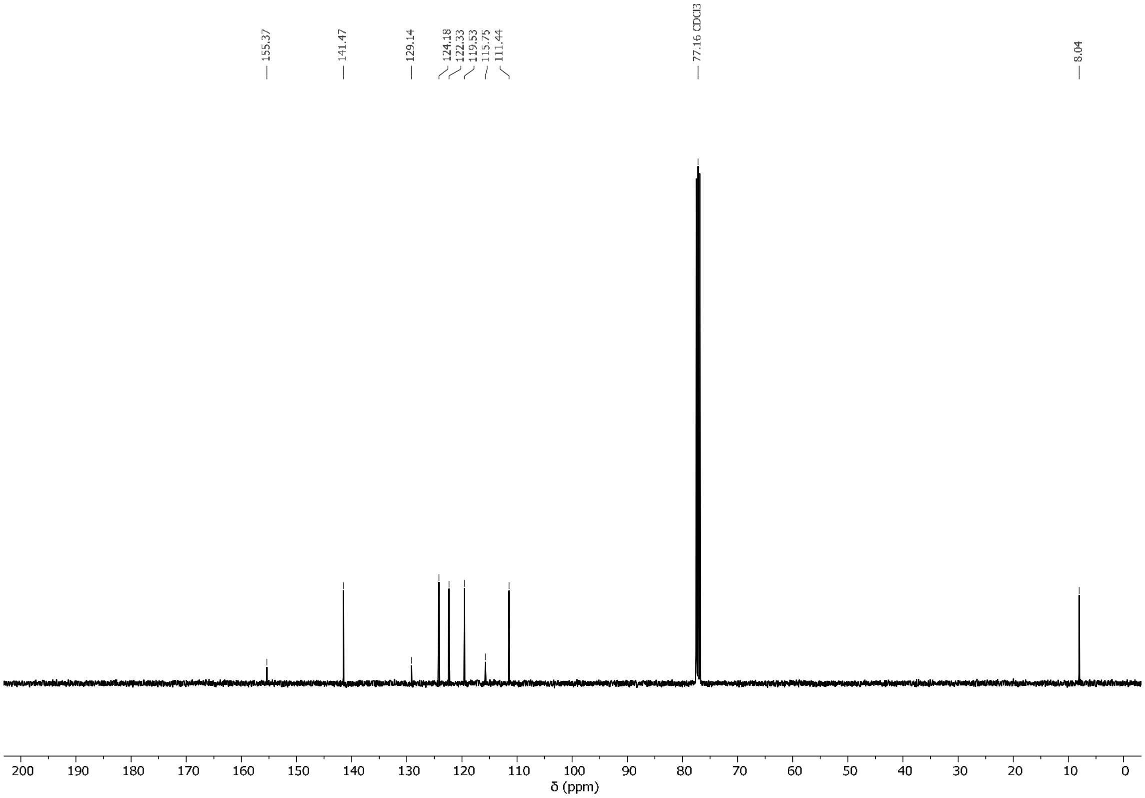
155.37
141.47
129.14
124.18
122.33
119.53
115.75
111.44
77.16 CDCl3
8.04
200 190 180 170 160 150 140 130 120 110 100 90 80 70 60 50 40 30 20 10 0
δ (ppm)

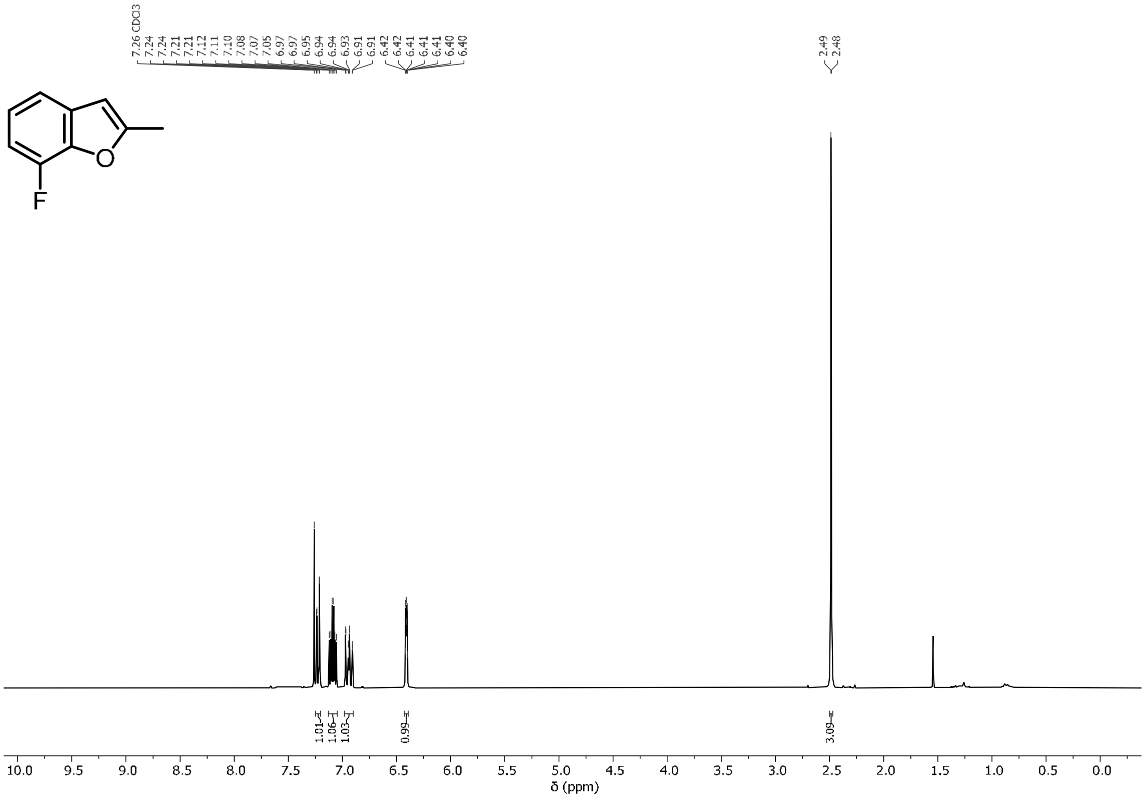
F
O
7.26 CDCl3
7.24
7.24
7.21
7.21
7.12
7.11
7.10
7.08
7.07
7.05
6.97
6.97
6.95
6.94
6.94
6.93
6.91
6.91
6.42
6.42
6.41
6.41
6.41
6.40
6.40
2.49
2.48
1.01
1.06
1.03
0.99
3.09
10.0 9.5 9.0 8.5 8.0 7.5 7.0 6.5 6.0 5.5 5.0 4.5 4.0 3.5 3.0 2.5 2.0 1.5 1.0 0.5 0.0
δ (ppm)

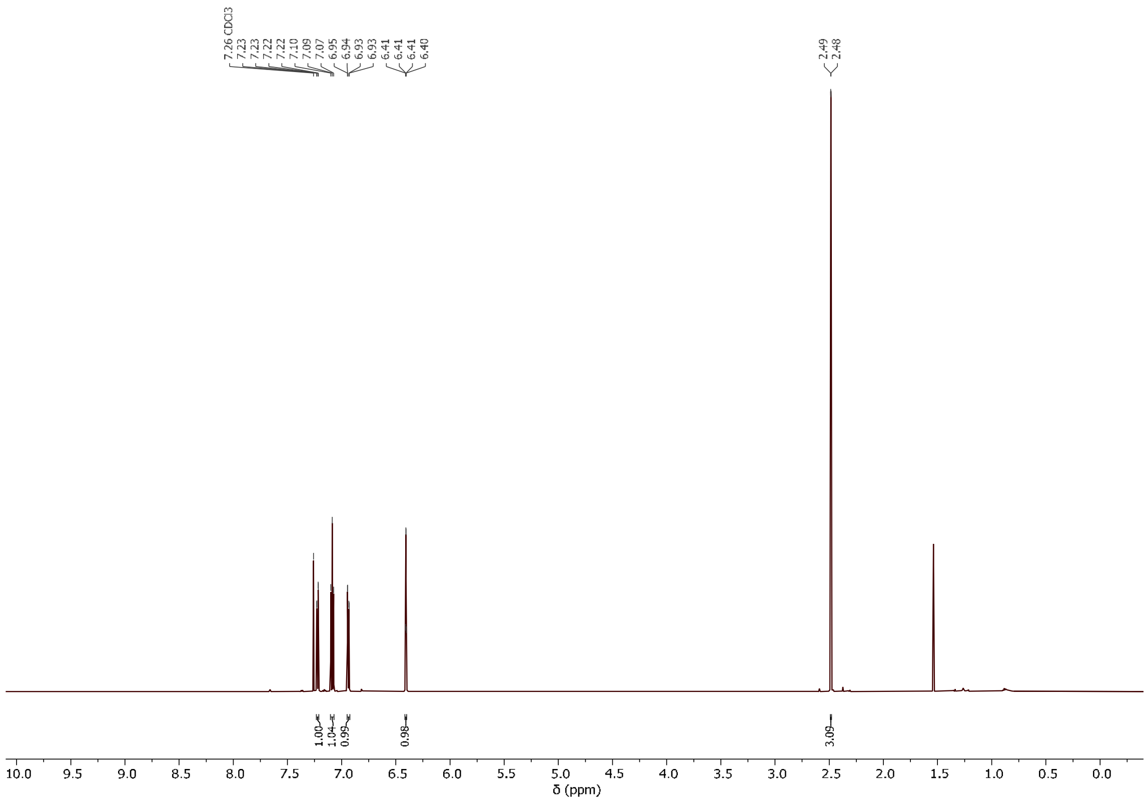
7.26 CDCl3
7.23
7.23
7.22
7.22
7.10
7.09
7.07
6.95
6.94
6.93
6.93
6.41
6.41
6.41
6.40
2.49
2.48
1.00
1.04
0.99
0.98
3.09
δ (ppm)

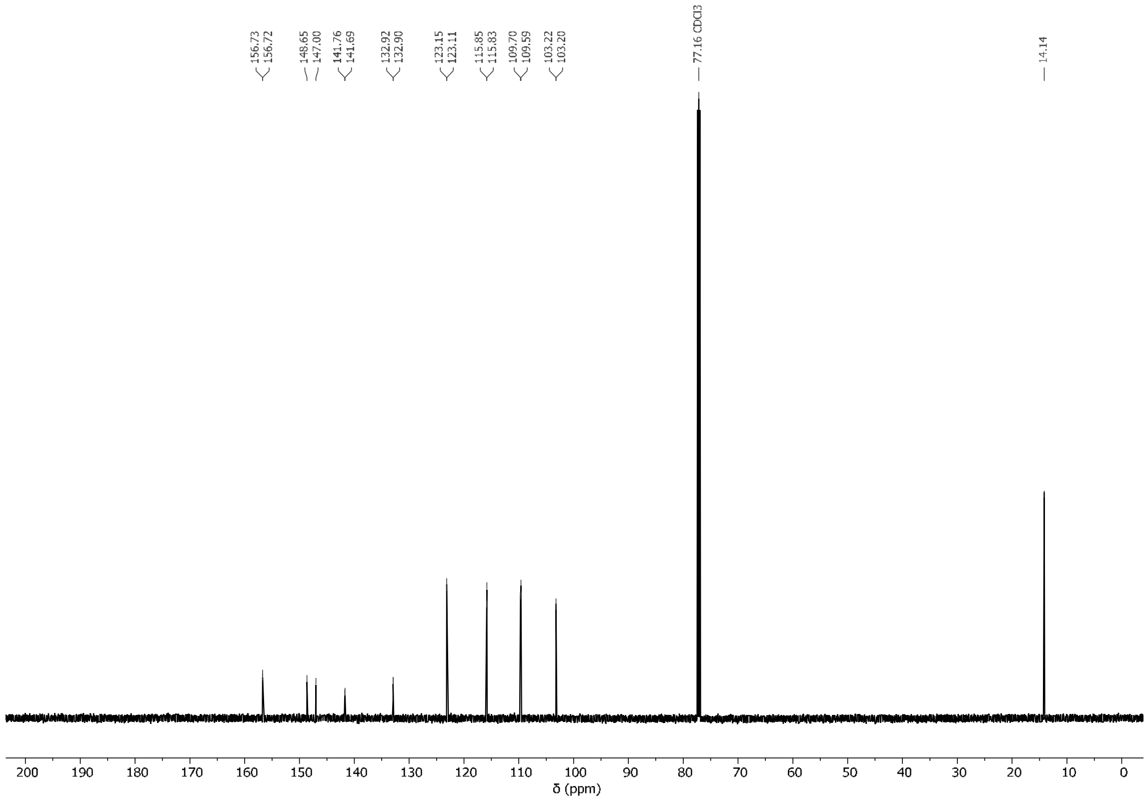
156.73
156.72
148.65
147.00
141.76
141.69
132.92
132.90
123.15
123.11
115.85
115.83
109.70
109.59
103.22
103.20
77.16 CDCl3
14.14
δ (ppm)

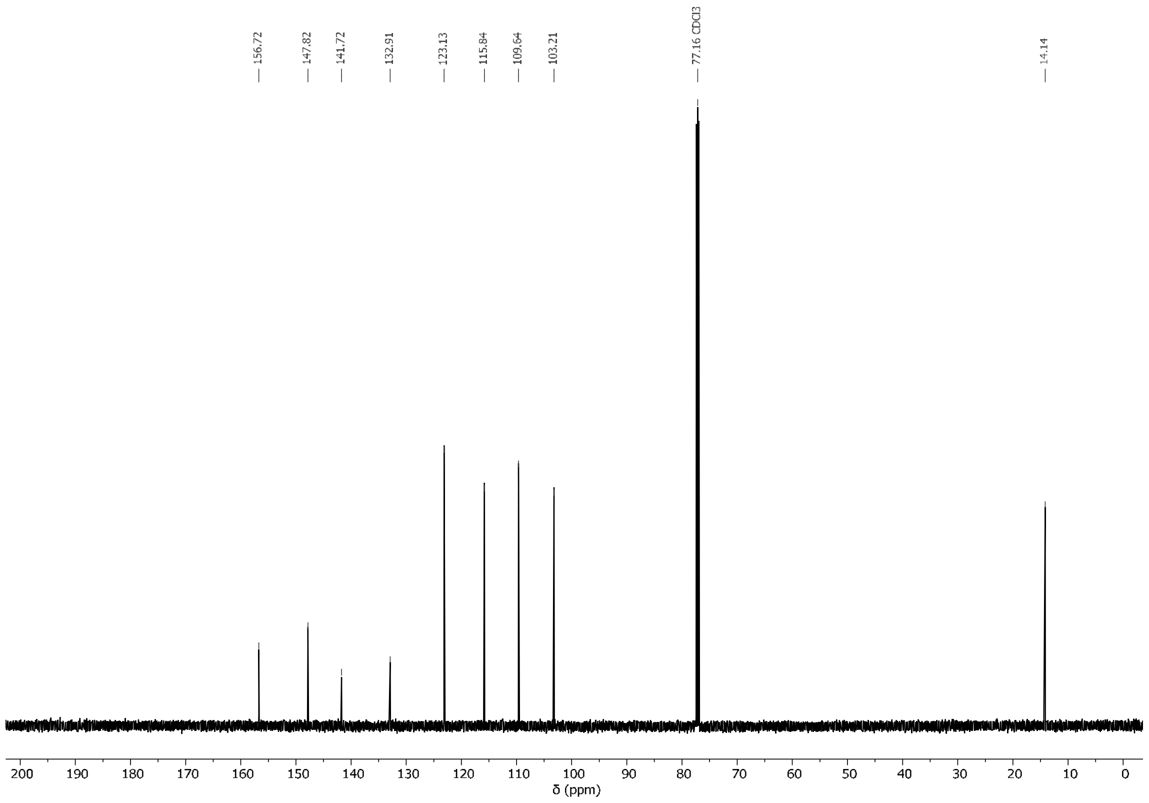
156.72
147.82
141.72
132.91
123.13
115.84
109.64
103.21
77.16 CDCl3
14.14
200 190 180 170 160 150 140 130 120 110 100 90 80 70 60 50 40 30 20 10 0
δ (ppm)

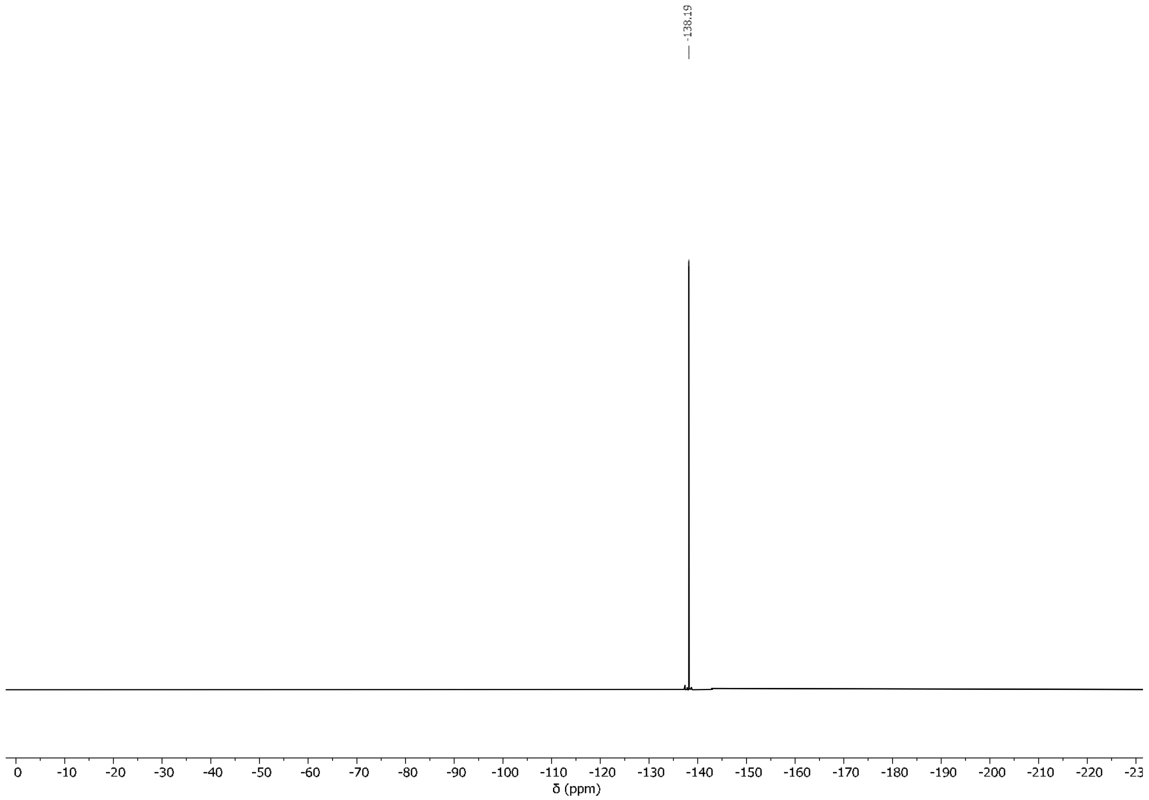
-138.19
0 -10 -20 -30 -40 -50 -60 -70 -80 -90 -100 -110 -120 -130 -140 -150 -160 -170 -180 -190 -200 -210 -220 -23
δ (ppm)

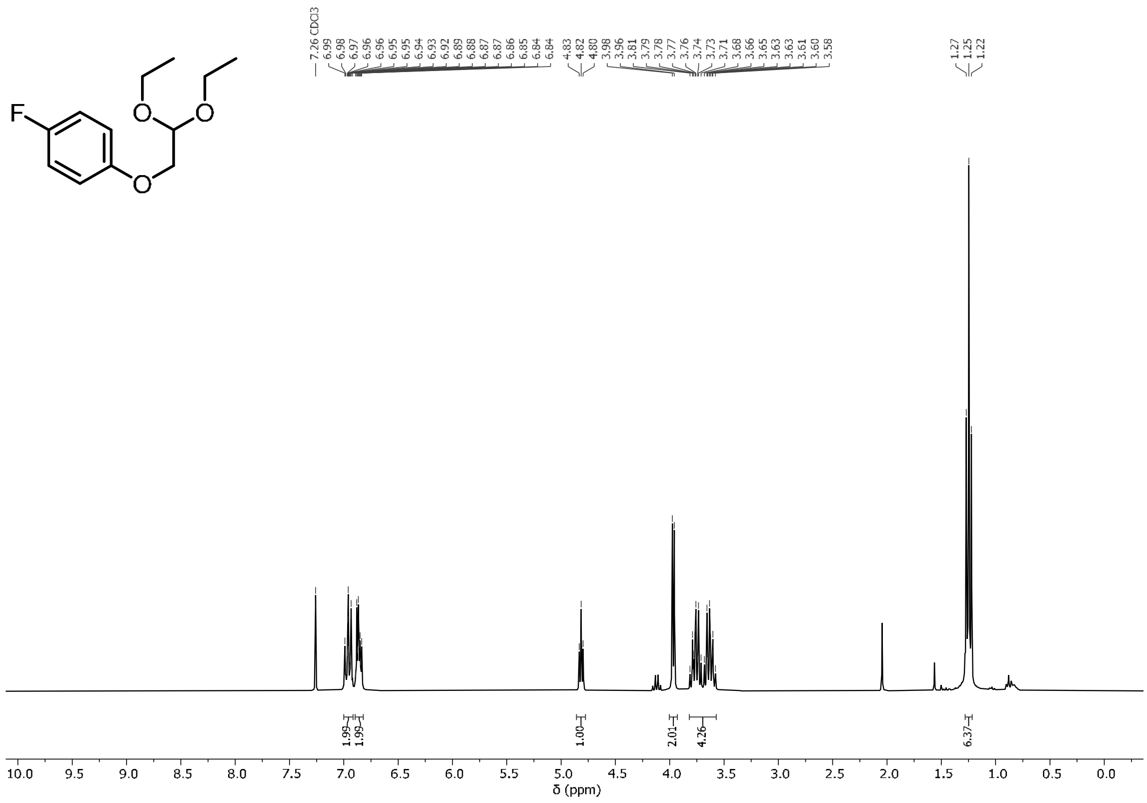
7.26 CDCl3
6.99
6.98
6.97
6.96
6.96
6.95
6.95
6.94
6.93
6.92
6.89
6.88
6.87
6.87
6.86
6.85
6.84
6.84
4.83
4.82
4.80
3.98
3.96
3.81
3.79
3.78
3.77
3.76
3.74
3.73
3.71
3.68
3.66
3.65
3.63
3.63
3.61
3.60
3.58
1.27
1.25
1.22
1.99
1.99
1.00
2.01
4.26
6.37
δ (ppm)

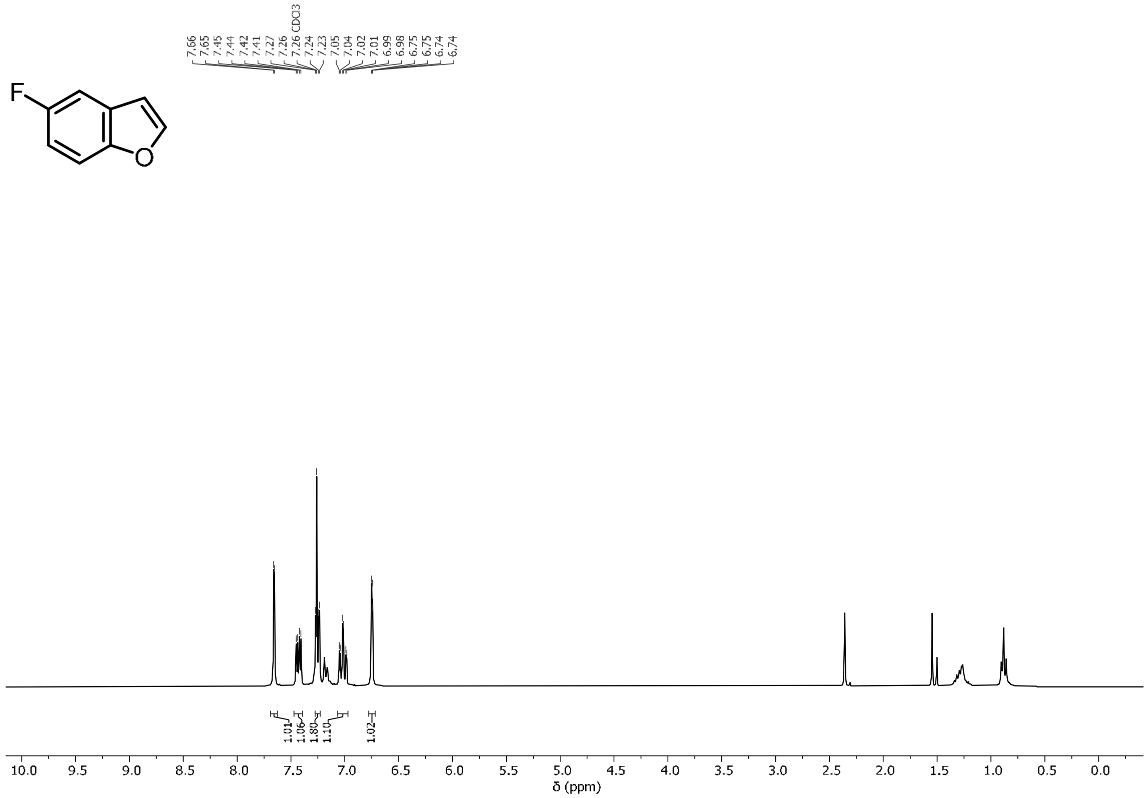
7.66
7.65
7.45
7.44
7.42
7.41
7.27
7.26 CDCl3
7.24
7.23
7.05
7.04
7.02
7.01
6.99
6.98
6.75
6.75
6.74
6.74
1.01
1.06
1.80
1.10
1.02
δ (ppm)

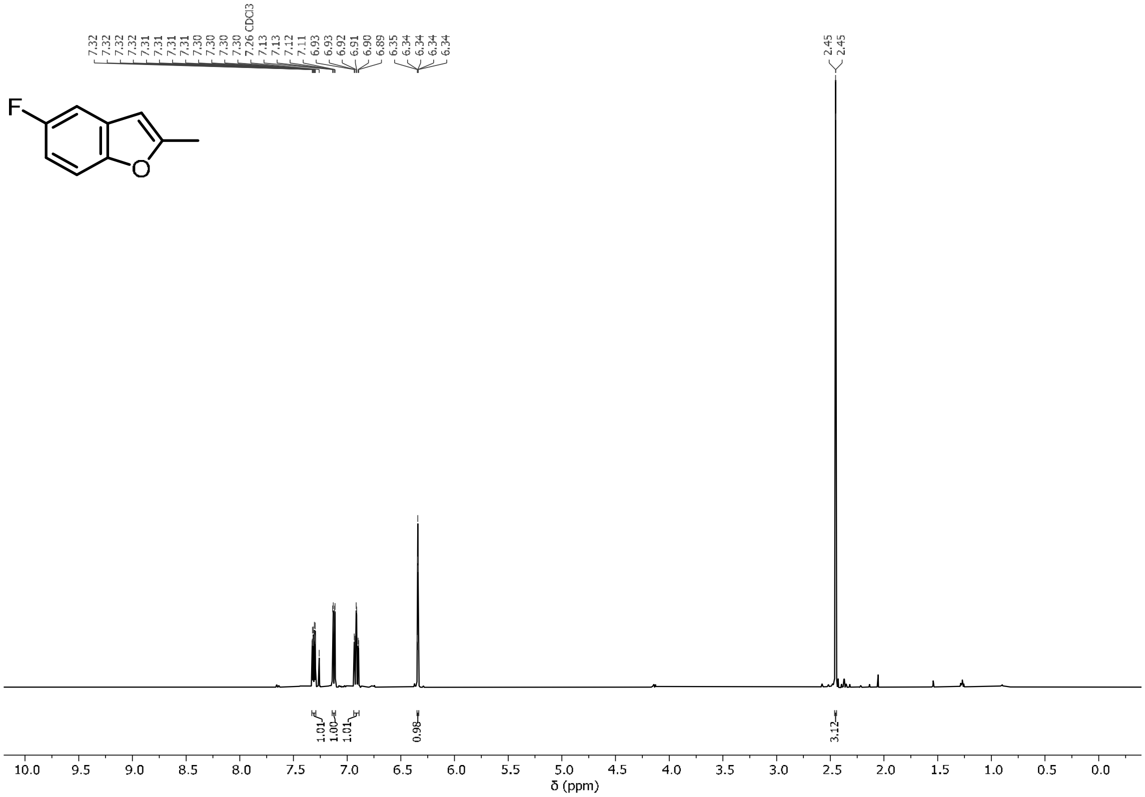

F
O
7.32
7.32
7.32
7.32
7.31
7.31
7.31
7.30
7.30
7.30
7.30
7.26 CDCl3
7.13
7.13
7.12
7.11
6.93
6.93
6.92
6.91
6.90
6.89
6.35
6.34
6.34
6.34
6.34
2.45
2.45
1.01
1.00
1.01
0.98
3.12
10.0 9.5 9.0 8.5 8.0 7.5 7.0 6.5 6.0 5.5 5.0 4.5 4.0 3.5 3.0 2.5 2.0 1.5 1.0 0.5 0.0
δ (ppm)

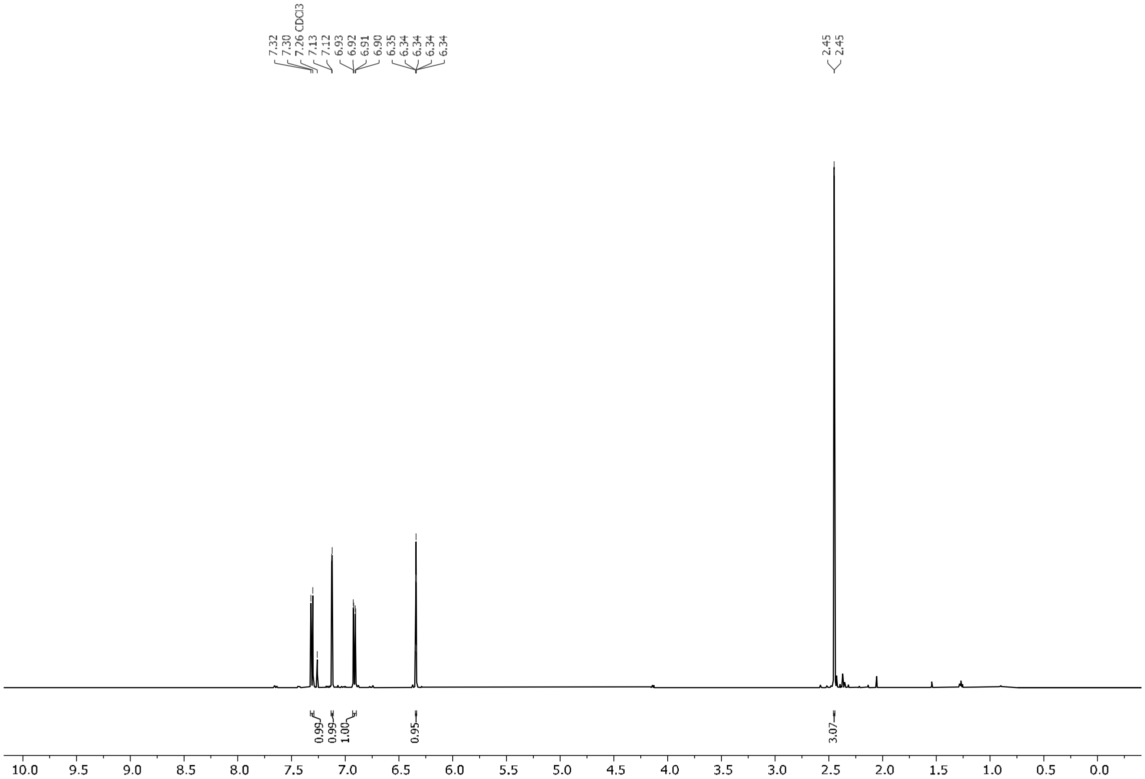

7.32
7.30
7.26 CDCl3
7.13
7.12
6.93
6.92
6.91
6.90
6.35
6.34
6.34
6.34
6.34
2.45
2.45
0.99
0.99
1.00
0.95
3.07
10.0 9.5 9.0 8.5 8.0 7.5 7.0 6.5 6.0 5.5 5.0 4.5 4.0 3.5 3.0 2.5 2.0 1.5 1.0 0.5 0.0
δ (ppm)

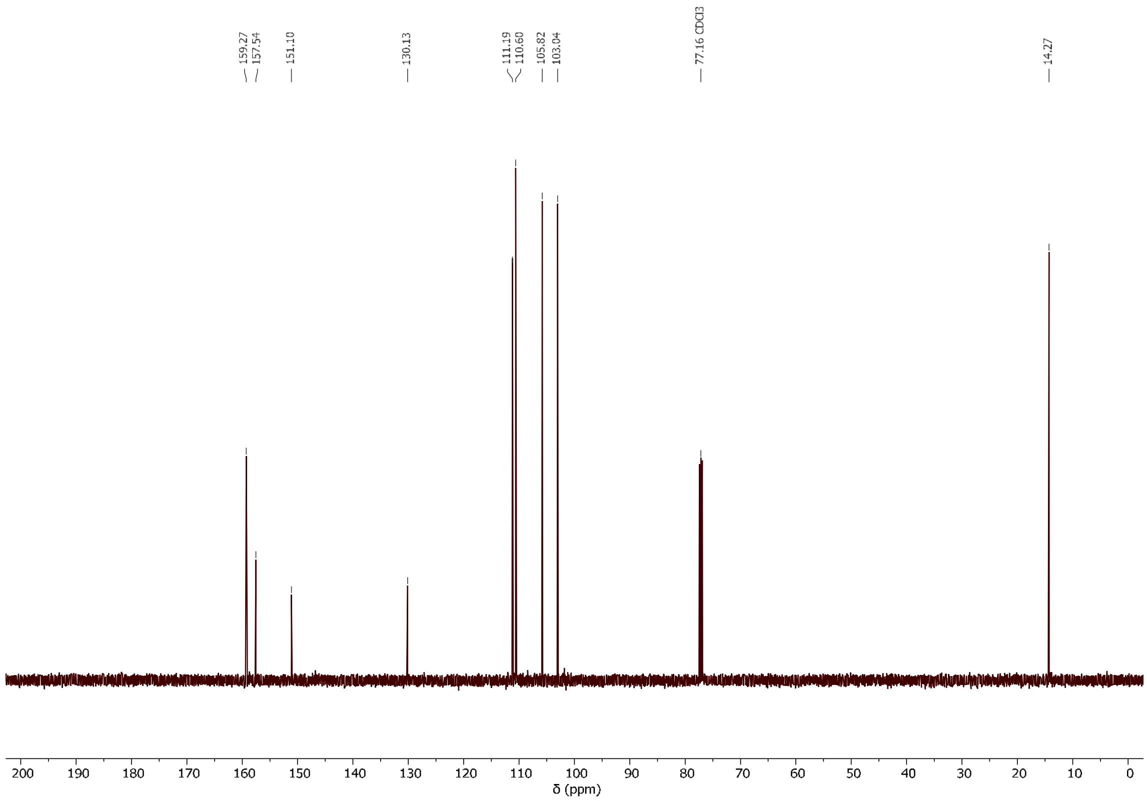
159.27
157.54
151.10
130.13
111.19
110.60
105.82
103.04
77.16 CDCl3
14.27
δ (ppm)

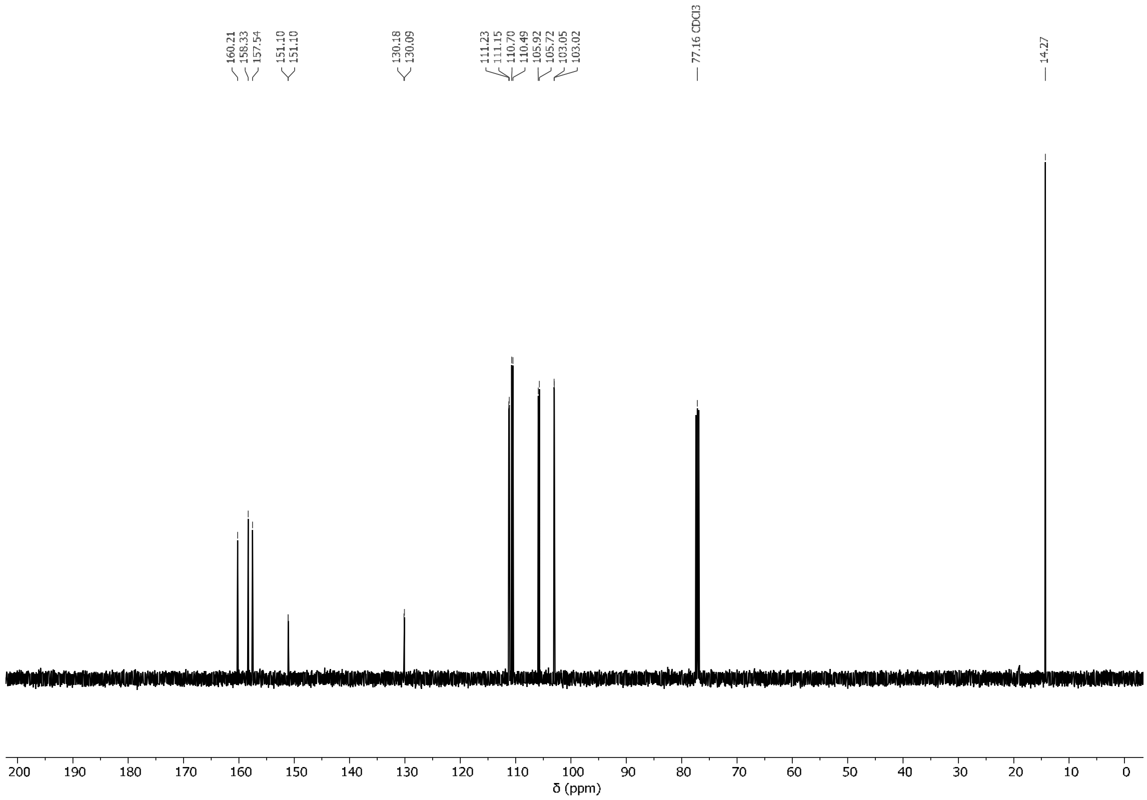
160.21
158.33
157.54
151.10
151.10
130.18
130.09
111.23
111.15
110.70
110.49
105.92
105.72
103.05
103.02
77.16 CDCl3
14.27
δ (ppm)

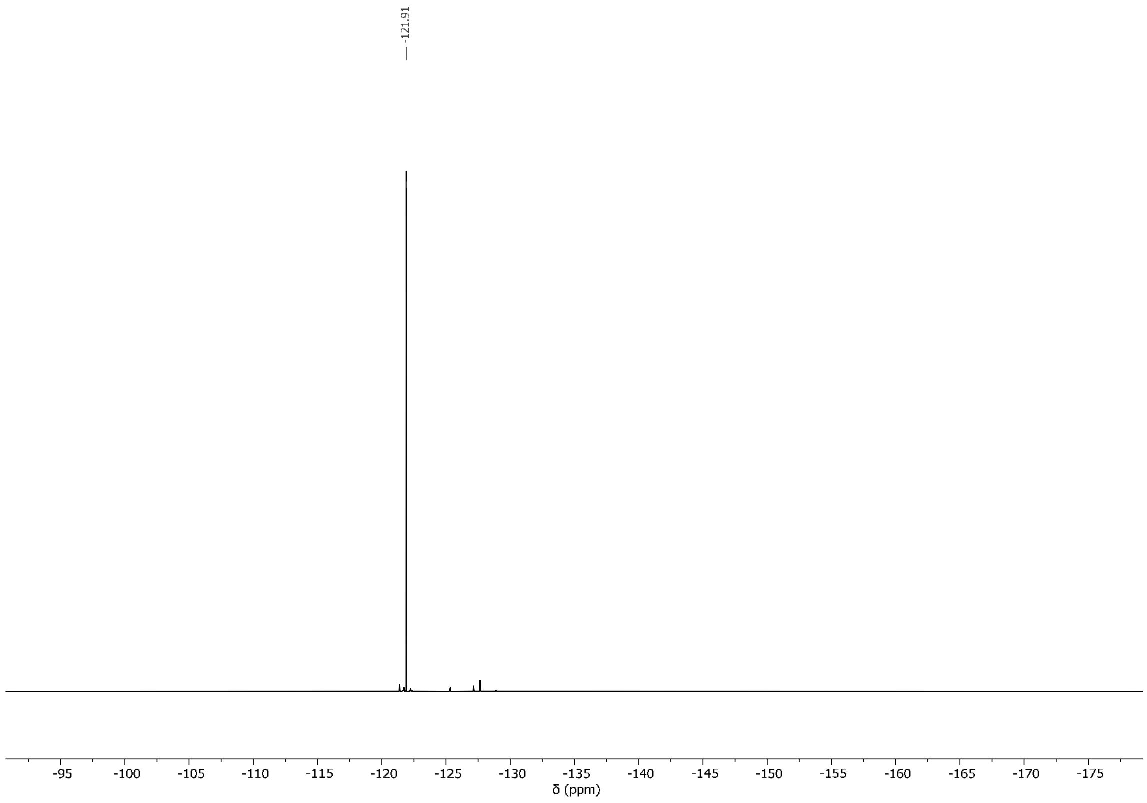

-121.91
-95
-100
-105
-110
-115
-120
-125
-130
-135
-140
-145
-150
-155
-160
-165
-170
-175
δ (ppm)

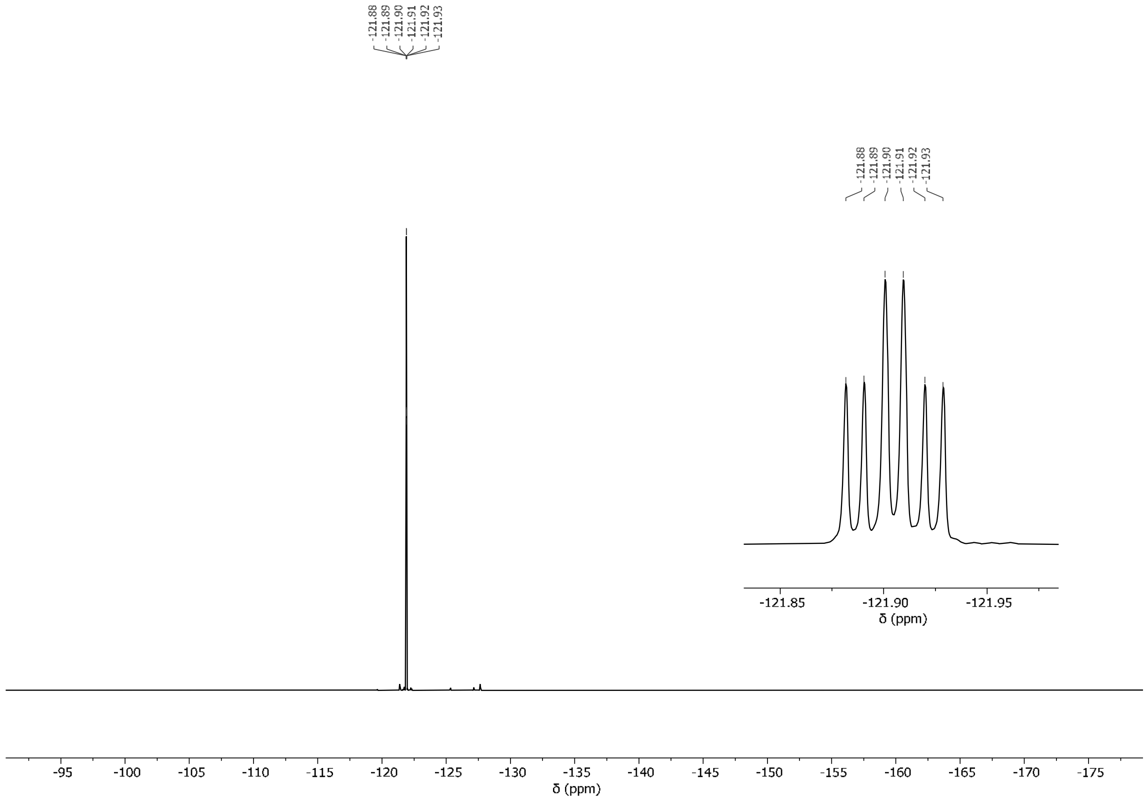

-121.88
-121.89
-121.90
-121.91
-121.92
-121.93
-121.85
-121.90
-121.95
δ (ppm)
-95
-100
-105
-110
-115
-120
-125
-130
-135
-140
-145
-150
-155
-160
-165
-170
-175
δ (ppm)

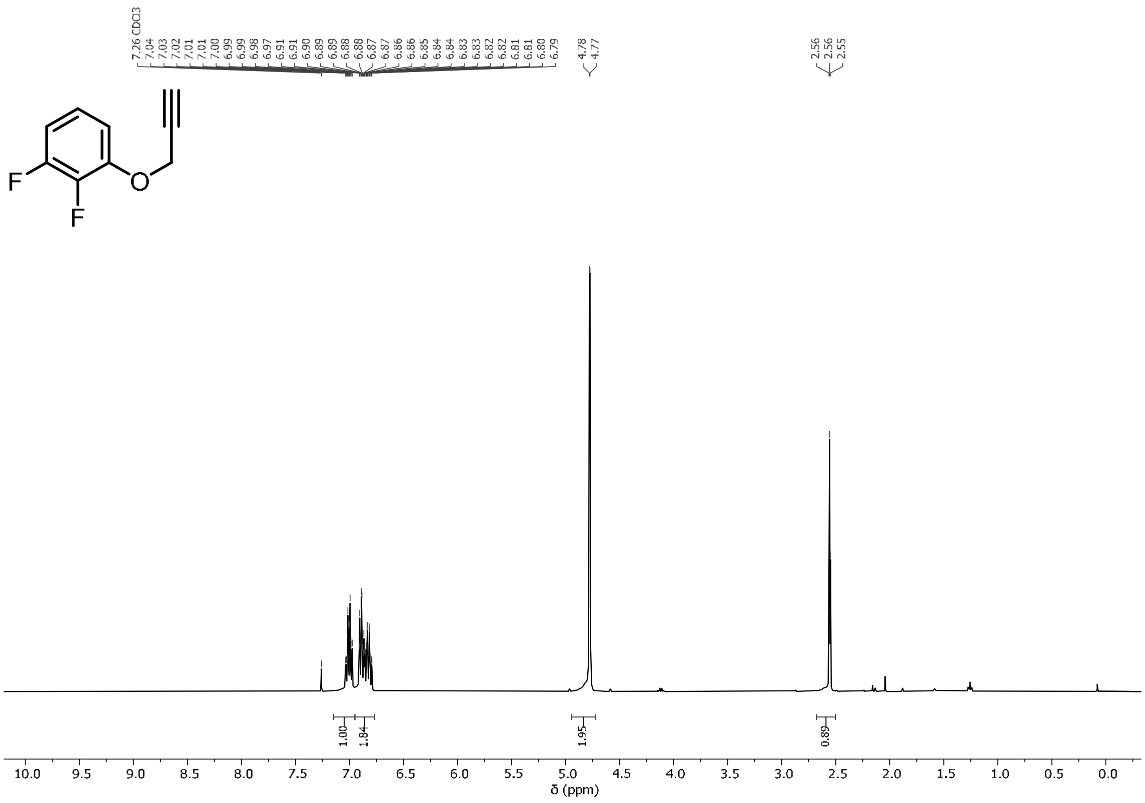

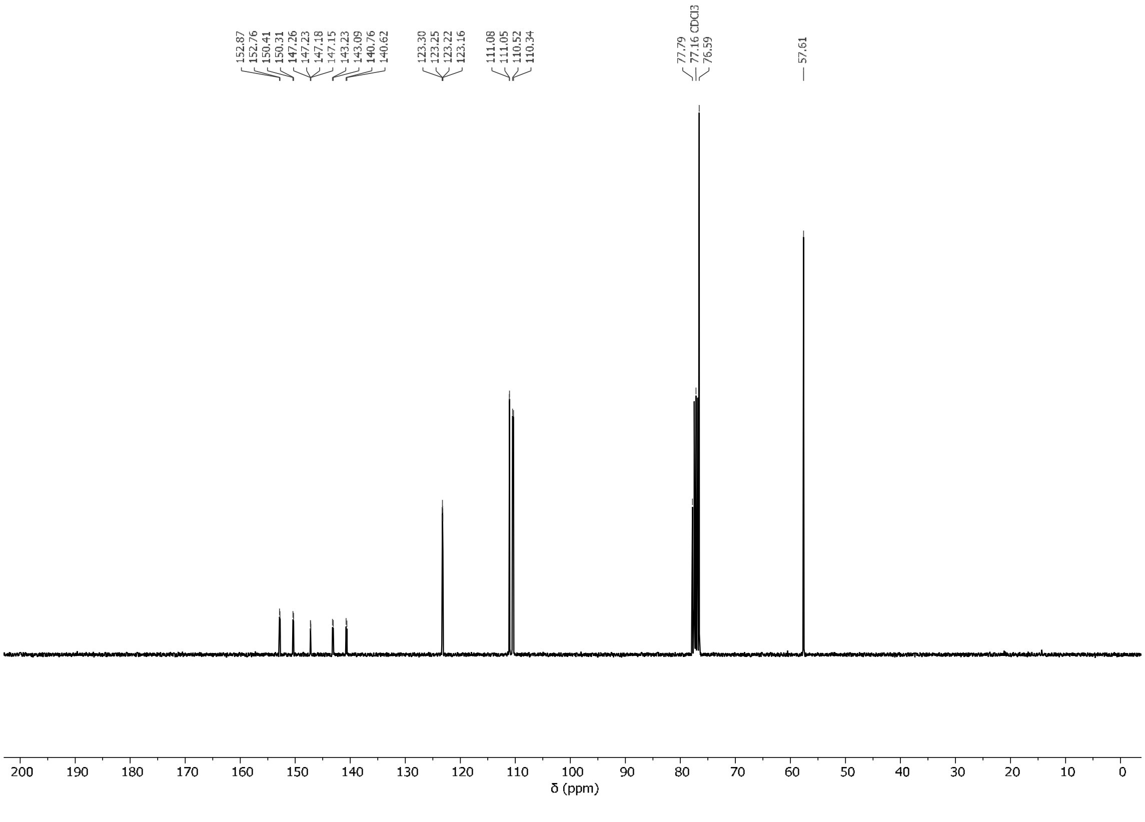

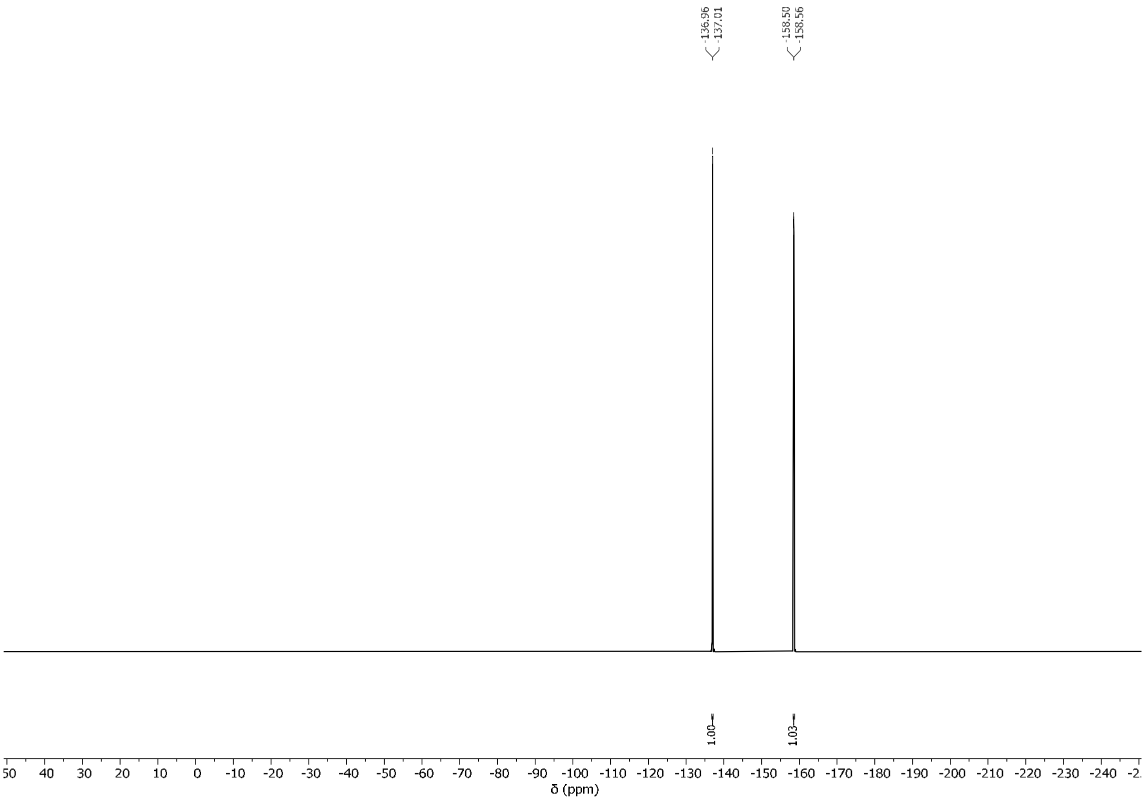
-136.96
-137.01
-158.50
-158.56
1.00
1.03
50 40 30 20 10 0 -10 -20 -30 -40 -50 -60 -70 -80 -90 -100 -110 -120 -130 -140 -150 -160 -170 -180 -190 -200 -210 -220 -230 -240
δ (ppm)

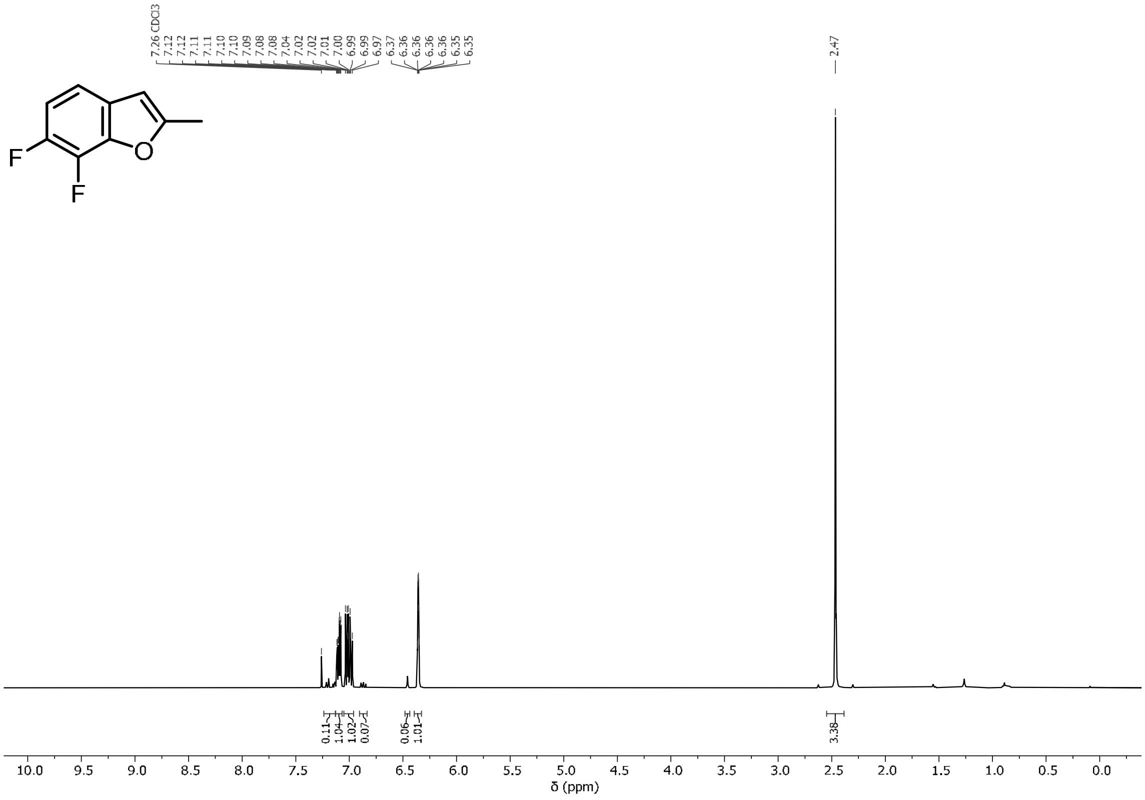
7.26 CDCl3
7.12
7.12
7.11
7.11
7.10
7.10
7.09
7.08
7.08
7.04
7.02
7.02
7.01
7.00
6.99
6.99
6.97
6.37
6.36
6.36
6.36
6.36
6.35
6.35
2.47
F
F
O
0.11
1.04
1.02
0.07
0.06
1.01
3.38
10.0 9.5 9.0 8.5 8.0 7.5 7.0 6.5 6.0 5.5 5.0 4.5 4.0 3.5 3.0 2.5 2.0 1.5 1.0 0.5 0.0
δ (ppm)

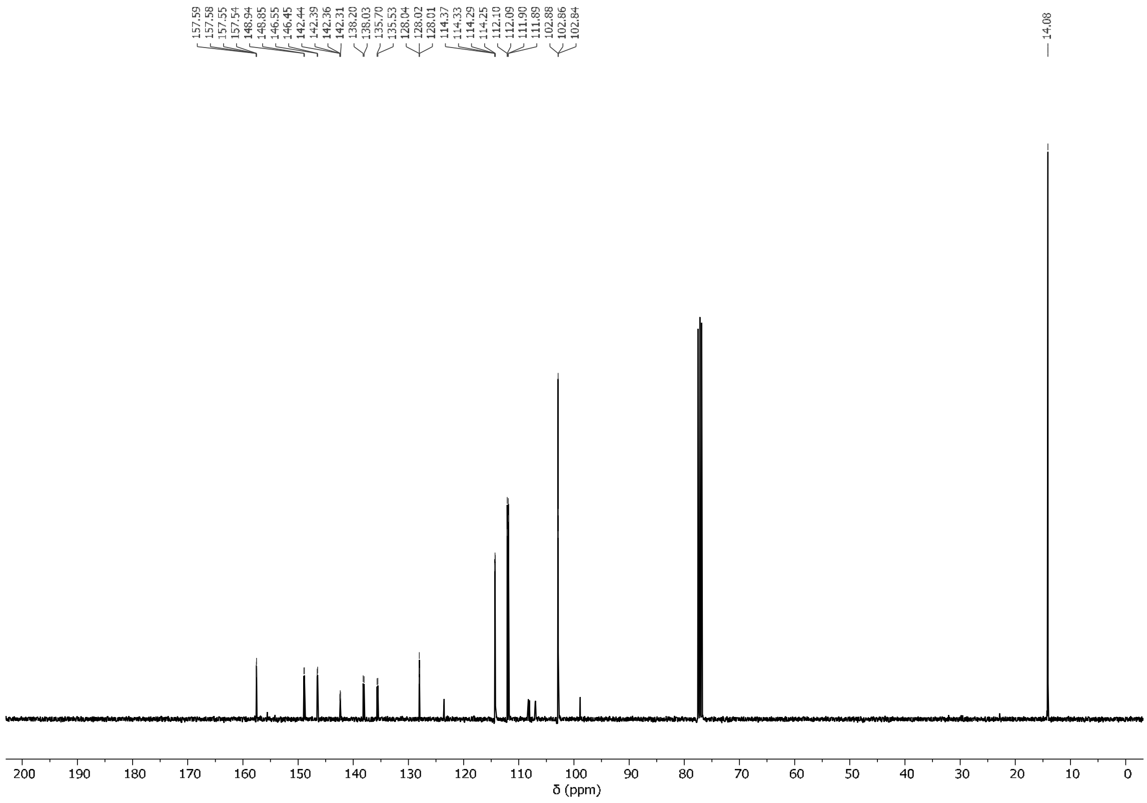
157.59
157.58
157.55
157.54
148.94
148.85
146.55
146.45
142.44
142.39
142.36
142.31
138.20
138.03
135.70
135.53
128.04
128.02
128.01
114.37
114.33
114.29
114.25
112.10
112.09
111.90
111.89
102.88
102.86
102.84
14.08
δ (ppm)

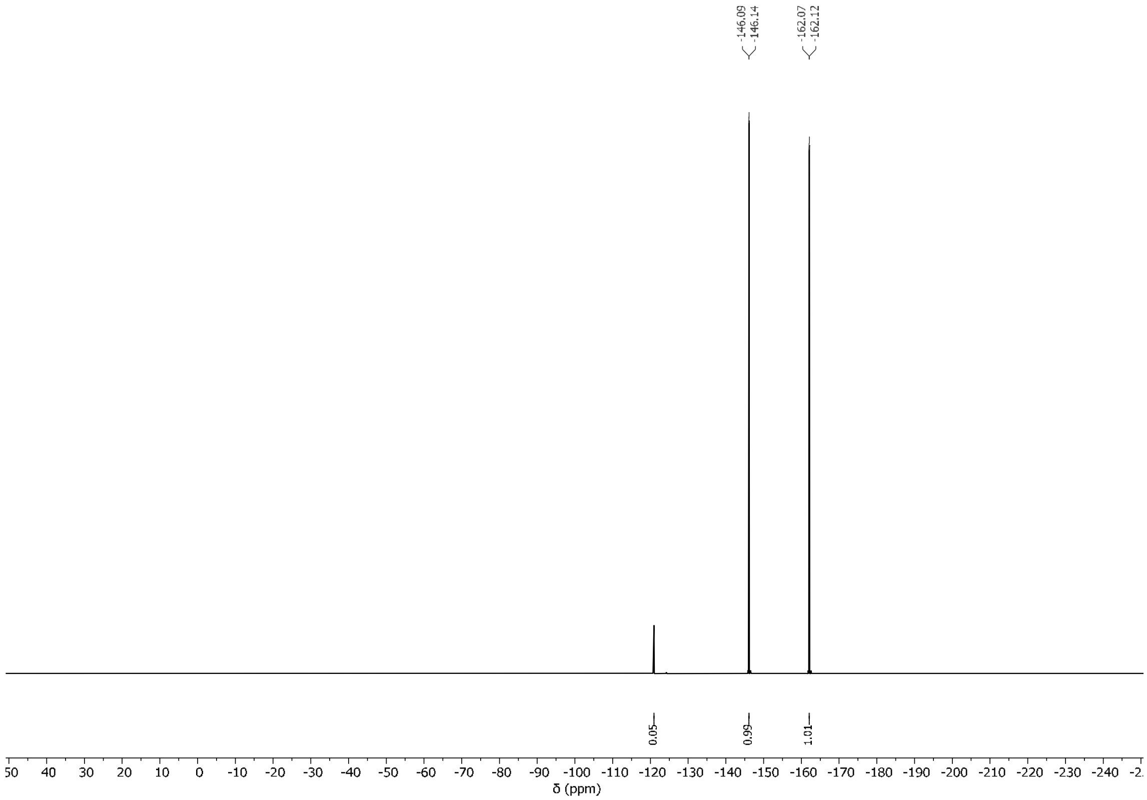
-146.09
-146.14
-162.07
-162.12
0.05
0.99
1.01
δ (ppm)

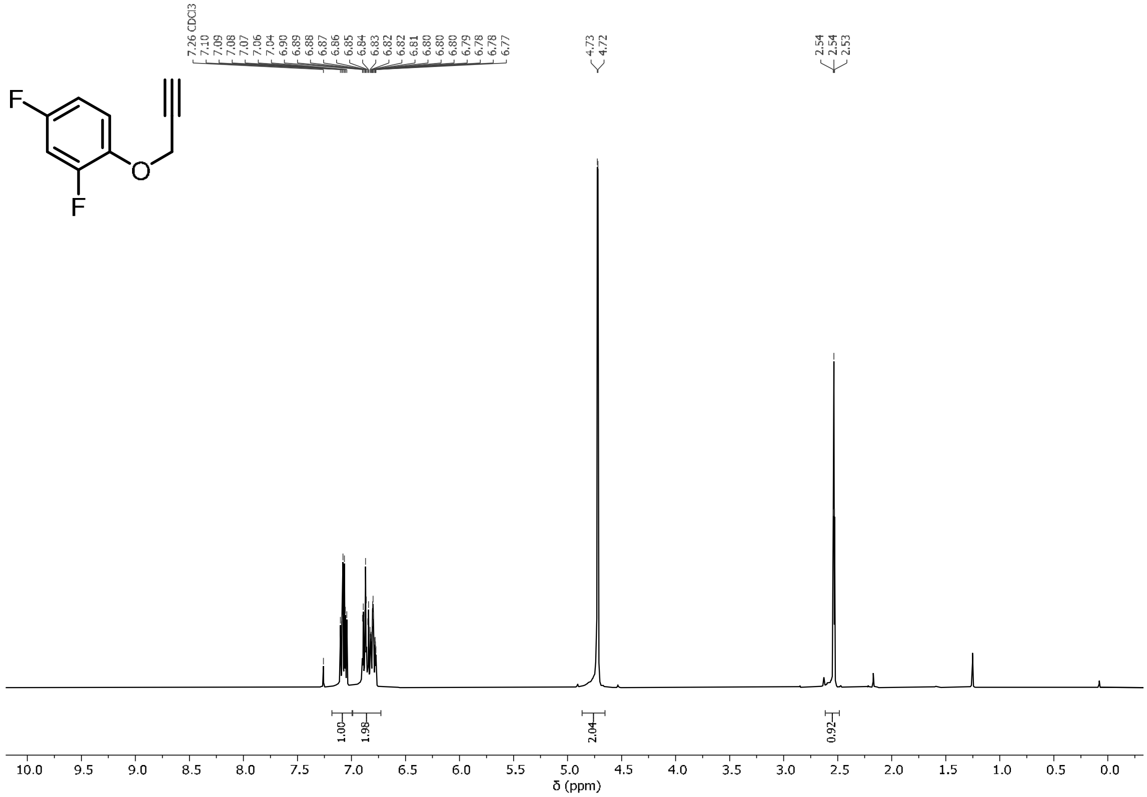
F
F
O
7.26 CDCl3
7.10
7.09
7.08
7.07
7.06
7.04
6.90
6.89
6.88
6.87
6.86
6.85
6.84
6.83
6.82
6.82
6.81
6.80
6.80
6.80
6.79
6.78
6.78
6.77
4.73
4.72
2.54
2.54
2.53
1.00
1.98
2.04
0.92
10.0 9.5 9.0 8.5 8.0 7.5 7.0 6.5 6.0 5.5 5.0 4.5 4.0 3.5 3.0 2.5 2.0 1.5 1.0 0.5 0.0
δ (ppm)

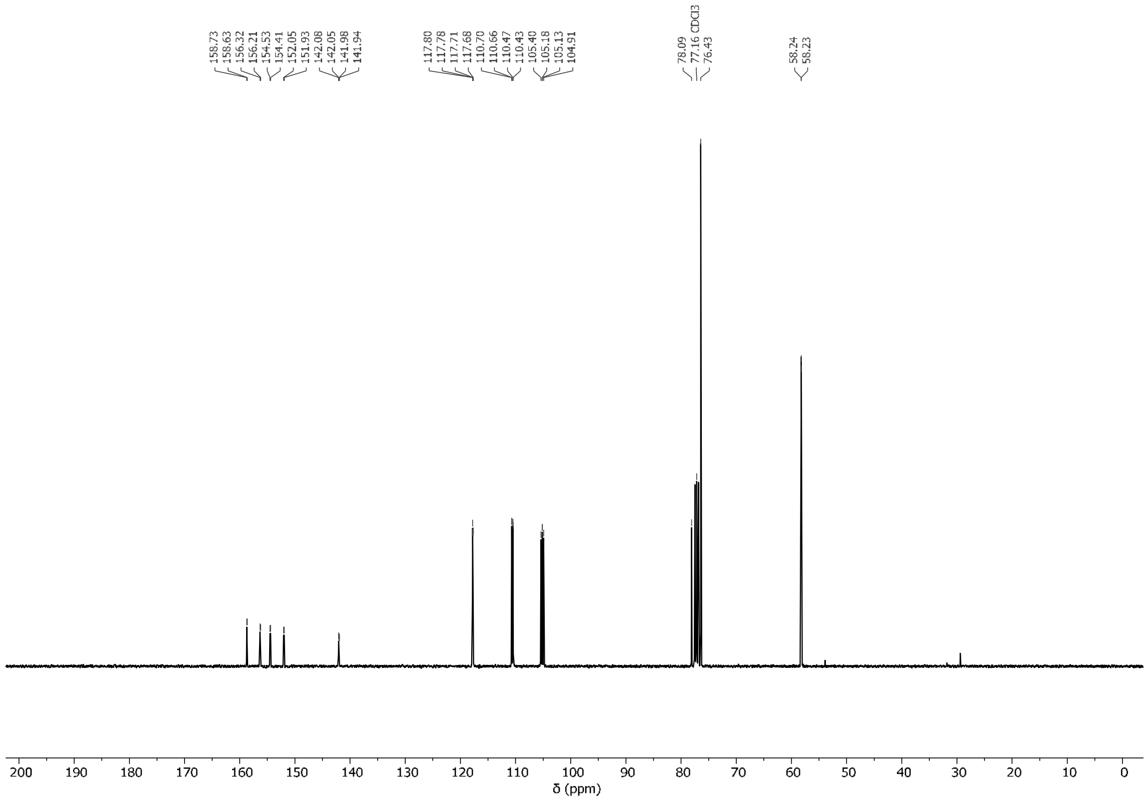
158.73
158.63
156.32
156.21
154.53
154.41
152.05
151.93
142.08
142.05
141.98
141.94
117.80
117.78
117.71
117.68
110.70
110.66
110.47
110.43
105.40
105.18
105.13
104.91
78.09
77.16 CDCl3
76.43
58.24
58.23
200 190 180 170 160 150 140 130 120 110 100 90 80 70 60 50 40 30 20 10 0
δ (ppm)

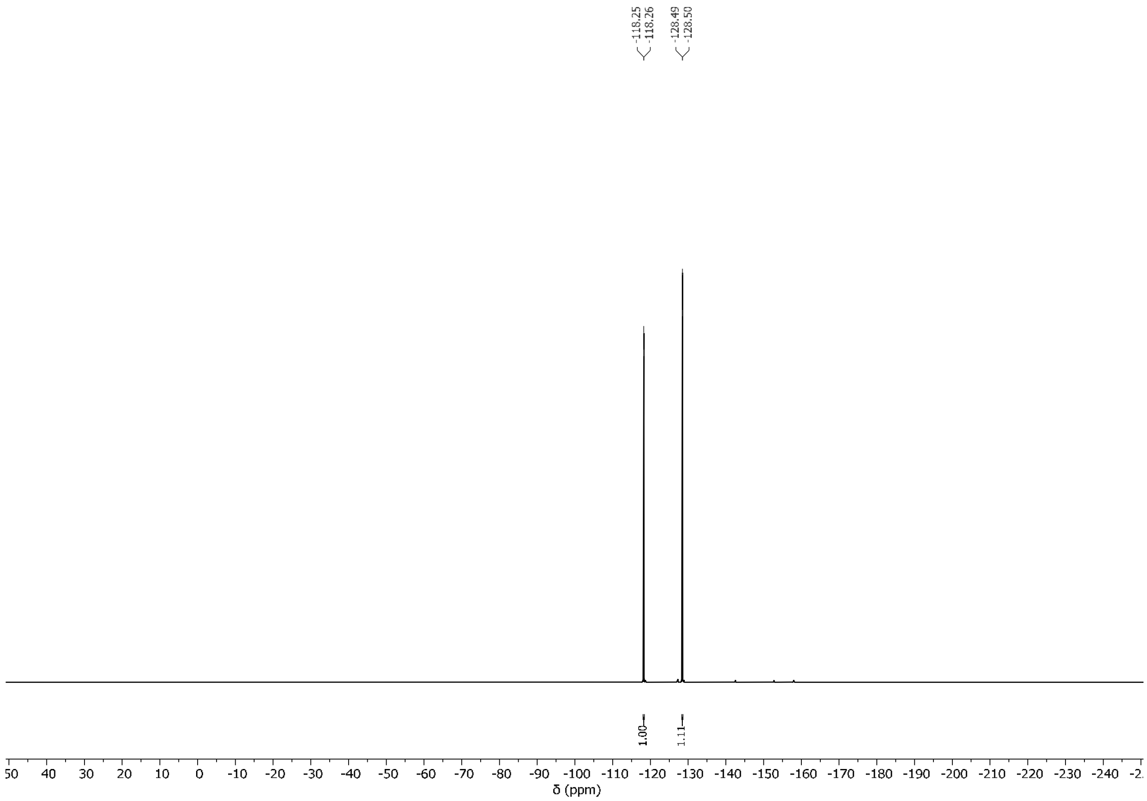
-118.25
-118.26
-128.49
-128.50
1.00
1.11
50 40 30 20 10 0 -10 -20 -30 -40 -50 -60 -70 -80 -90 -100 -110 -120 -130 -140 -150 -160 -170 -180 -190 -200 -210 -220 -230 -240
δ (ppm)

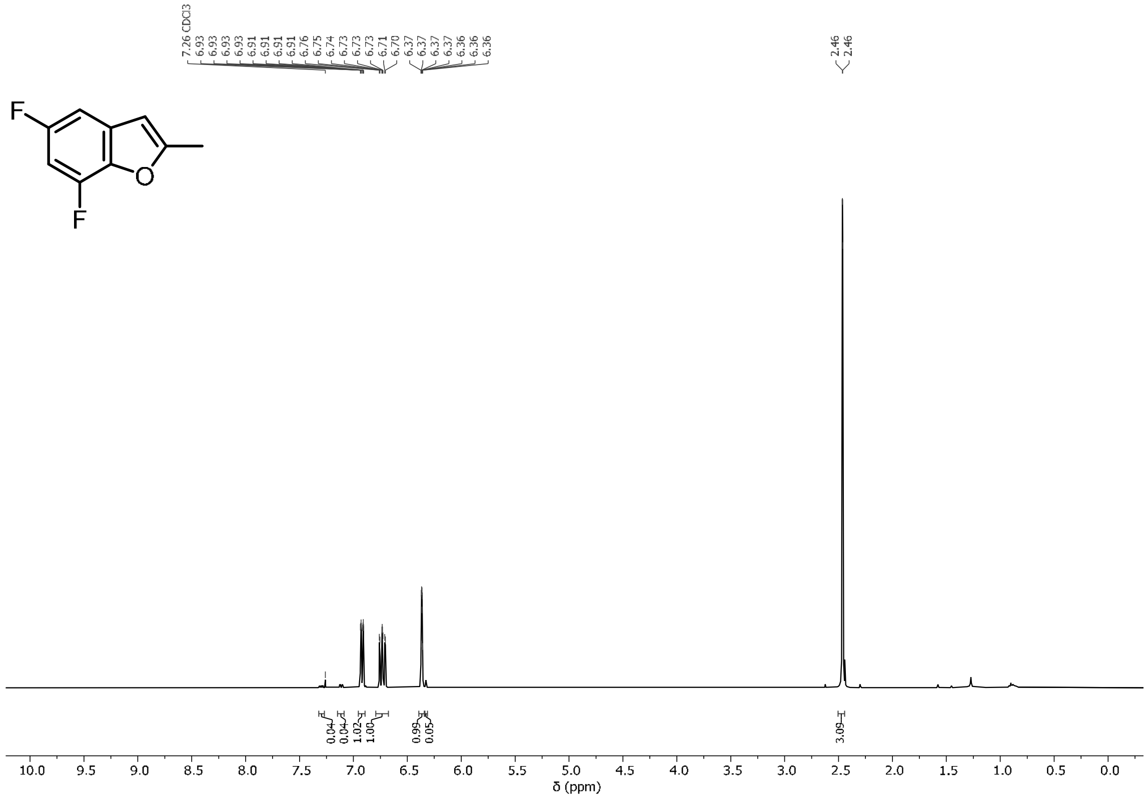
7.26 CDCl3
6.93
6.93
6.93
6.93
6.91
6.91
6.91
6.91
6.76
6.75
6.74
6.73
6.73
6.73
6.71
6.70
6.37
6.37
6.37
6.37
6.36
6.36
6.36
2.46
2.46
F
F
O
0.04
0.04
1.02
1.00
0.99
0.05
3.09
10.0 9.5 9.0 8.5 8.0 7.5 7.0 6.5 6.0 5.5 5.0 4.5 4.0 3.5 3.0 2.5 2.0 1.5 1.0 0.5 0.0
δ (ppm)

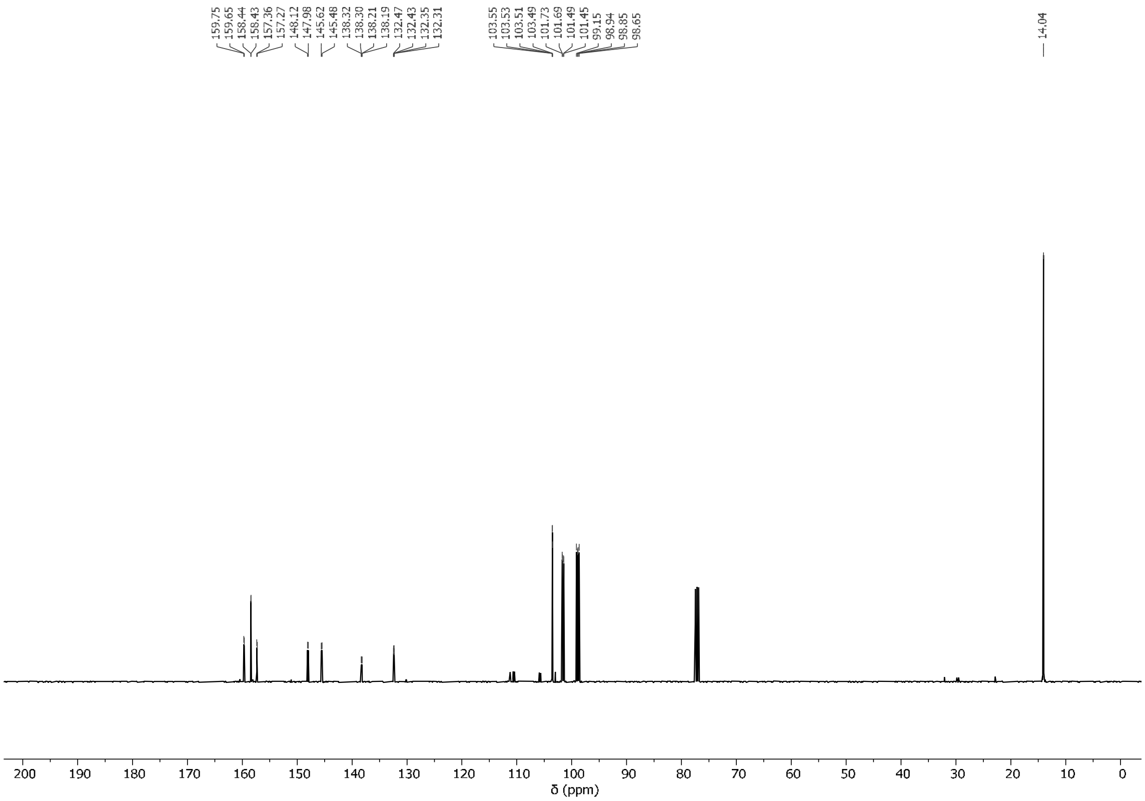
159.75
159.65
158.44
158.43
157.36
157.27
148.12
147.98
145.62
145.48
138.32
138.30
138.21
138.19
132.47
132.43
132.35
132.31
103.55
103.53
103.51
103.49
101.73
101.69
101.49
101.45
99.15
98.94
98.85
98.65
14.04
δ (ppm)

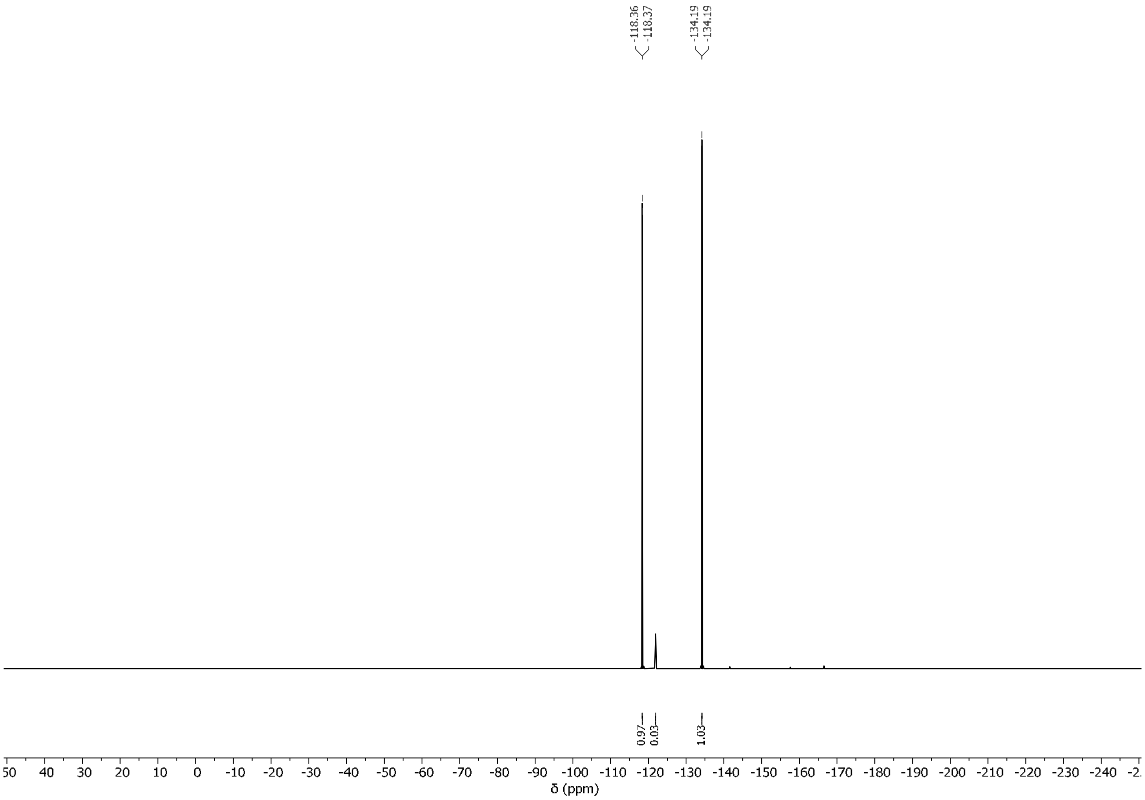
-118.36
-118.37
-134.19
-134.19
0.97
0.03
1.03
δ (ppm)

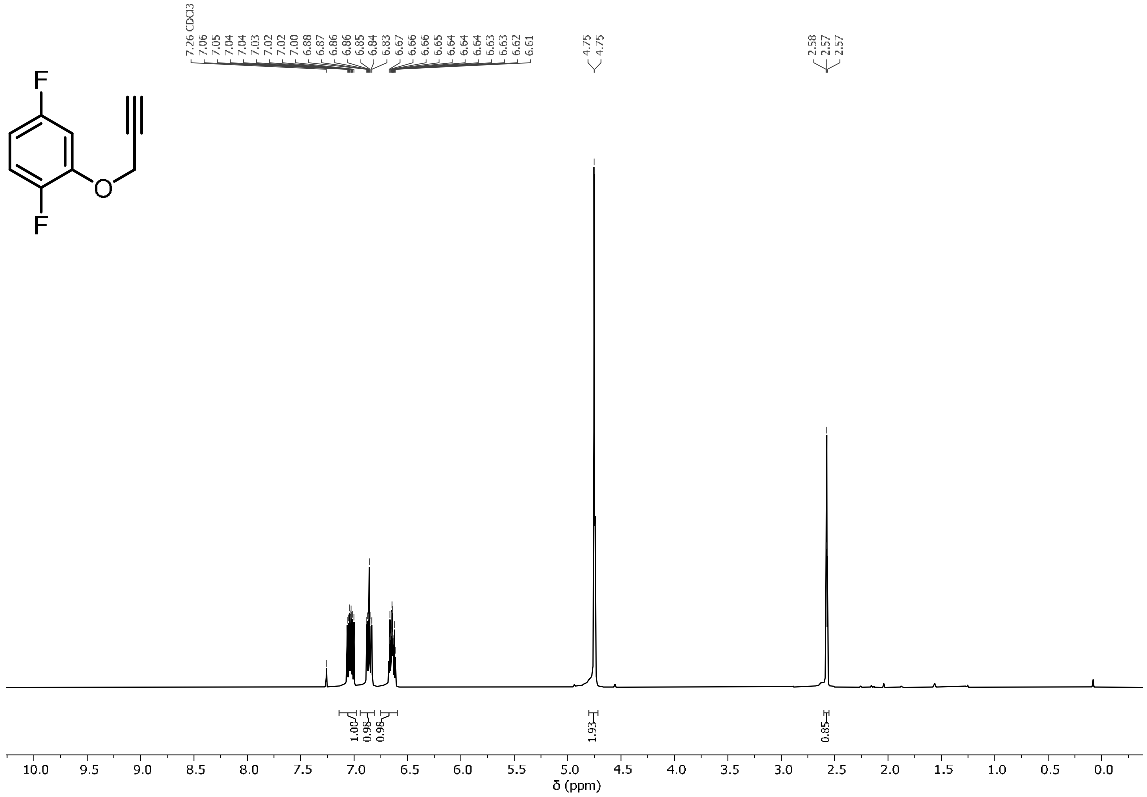
F
O
F
7.26 CDCl3
7.06
7.05
7.04
7.04
7.03
7.02
7.02
7.00
6.88
6.87
6.86
6.86
6.85
6.84
6.83
6.67
6.66
6.66
6.65
6.64
6.64
6.64
6.63
6.63
6.62
6.61
4.75
4.75
2.58
2.57
2.57
1.00
0.98
0.98
1.93
0.85
10.0 9.5 9.0 8.5 8.0 7.5 7.0 6.5 6.0 5.5 5.0 4.5 4.0 3.5 3.0 2.5 2.0 1.5 1.0 0.5 0.0
δ (ppm)

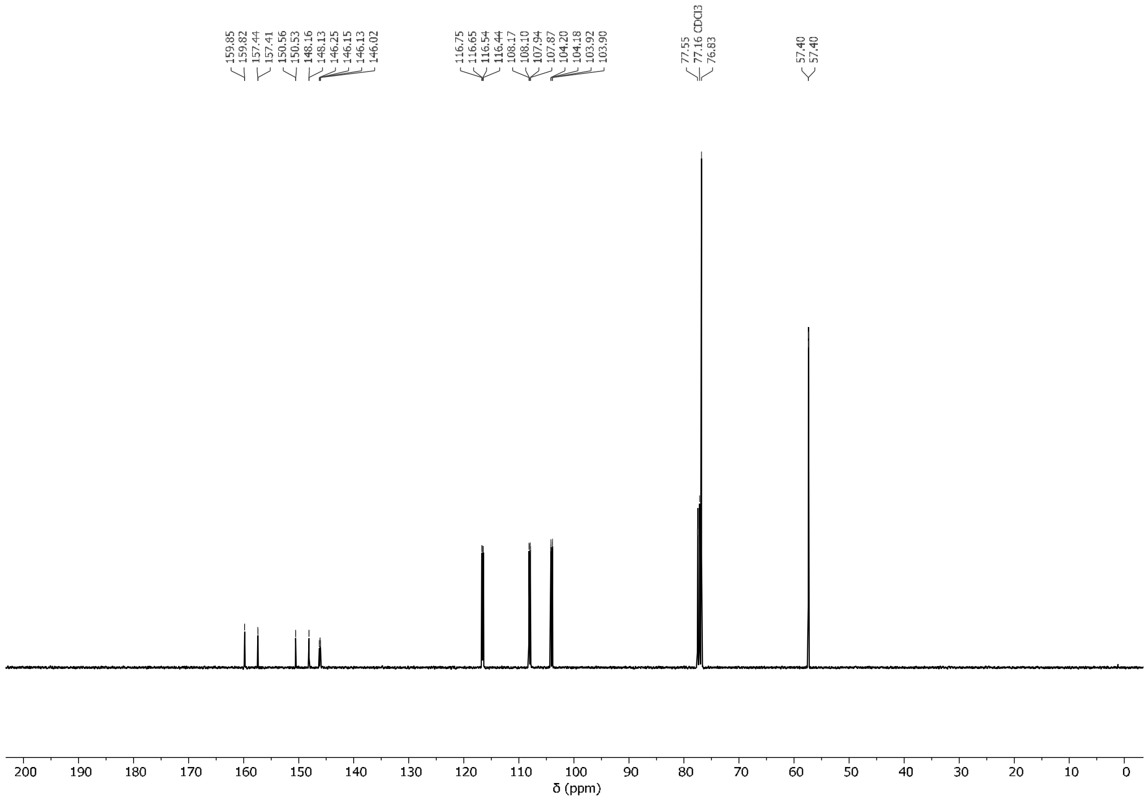
159.85
159.82
157.44
157.41
150.56
150.53
148.16
148.13
146.25
146.15
146.13
146.02
116.75
116.65
116.54
116.44
108.17
108.10
107.94
107.87
104.20
104.18
103.92
103.90
77.55
77.16 CDCl3
76.83
57.40
57.40
200 190 180 170 160 150 140 130 120 110 100 90 80 70 60 50 40 30 20 10 0
δ (ppm)

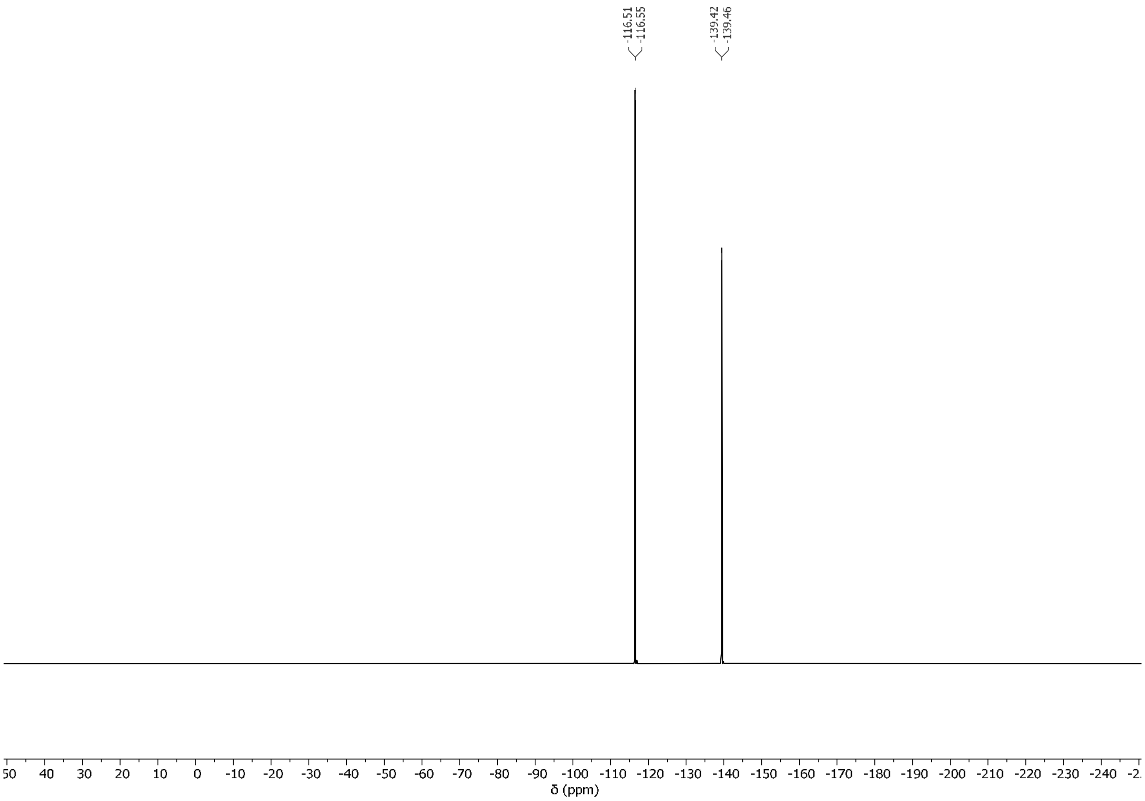
-116.51
-116.55
-139.42
-139.46
δ (ppm)

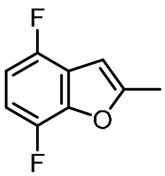
F
O
F

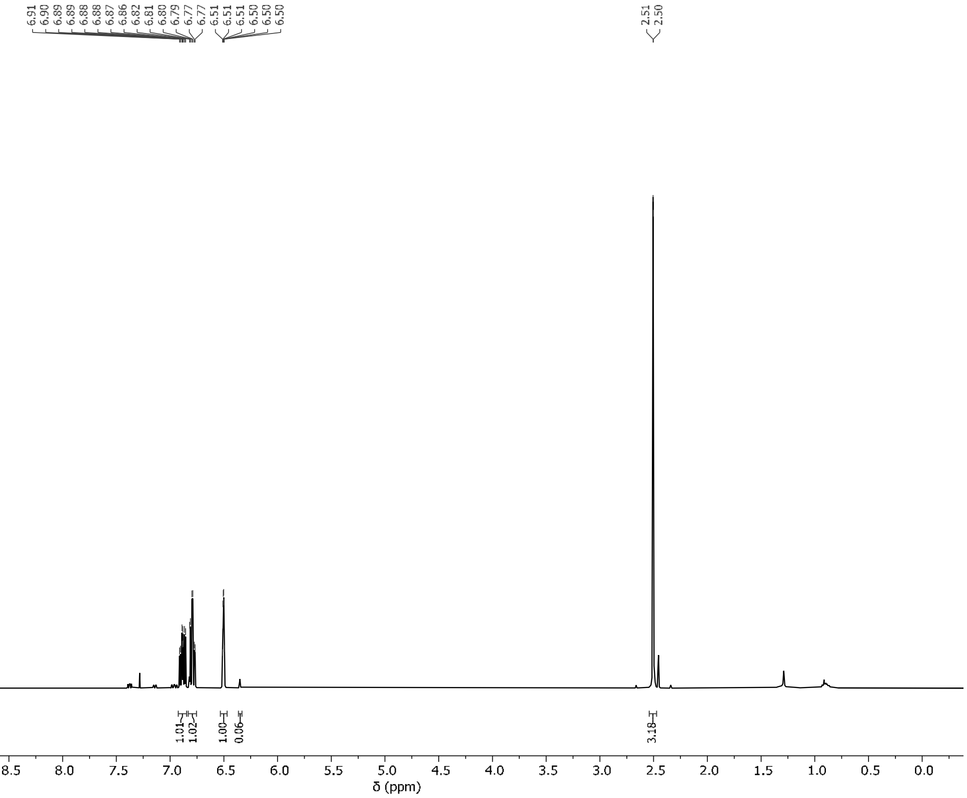
6.91
6.90
6.89
6.89
6.88
6.88
6.87
6.86
6.82
6.81
6.80
6.79
6.77
6.77
6.51
6.51
6.51
6.50
6.50
6.50
2.51
2.50
1.01
1.02
1.00
0.06
3.18
δ (ppm)

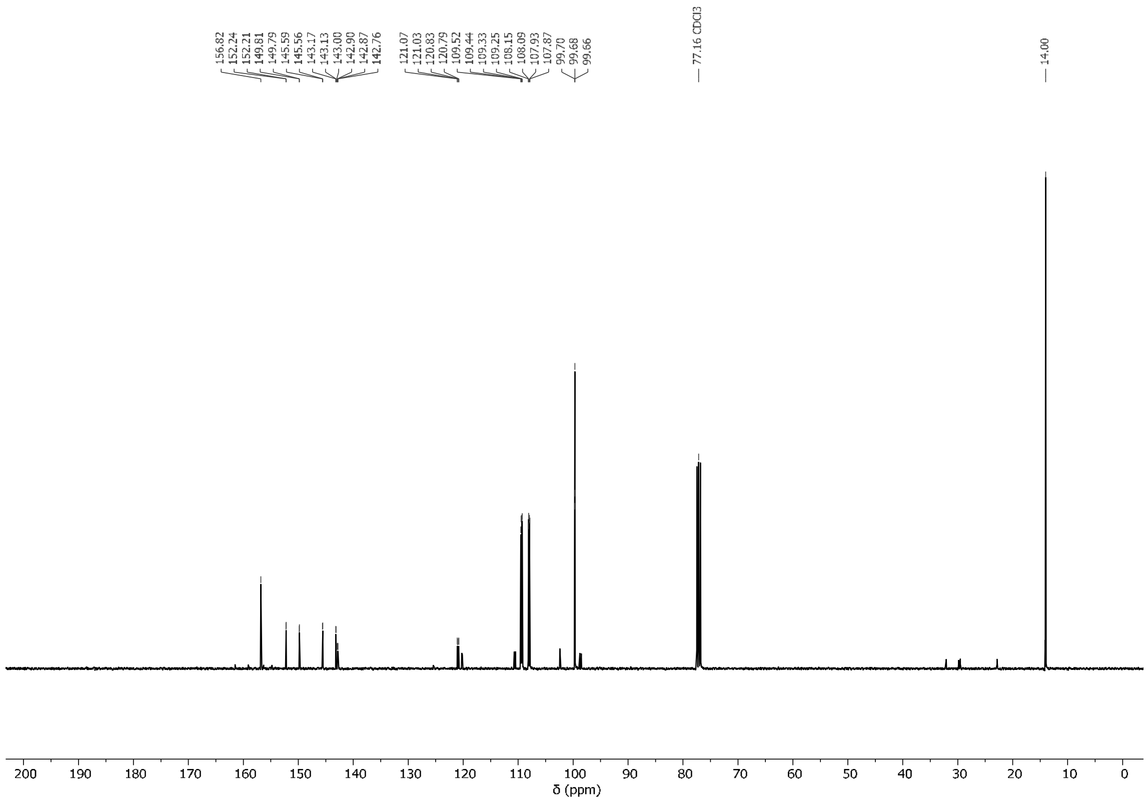

156.82
152.24
152.21
149.81
149.79
145.59
145.56
143.17
143.13
143.00
142.90
142.87
142.76
121.07
121.03
120.83
120.79
109.52
109.44
109.33
109.25
108.15
108.09
107.93
107.87
99.70
99.68
99.66
77.16 CDCl3
14.00
δ (ppm)

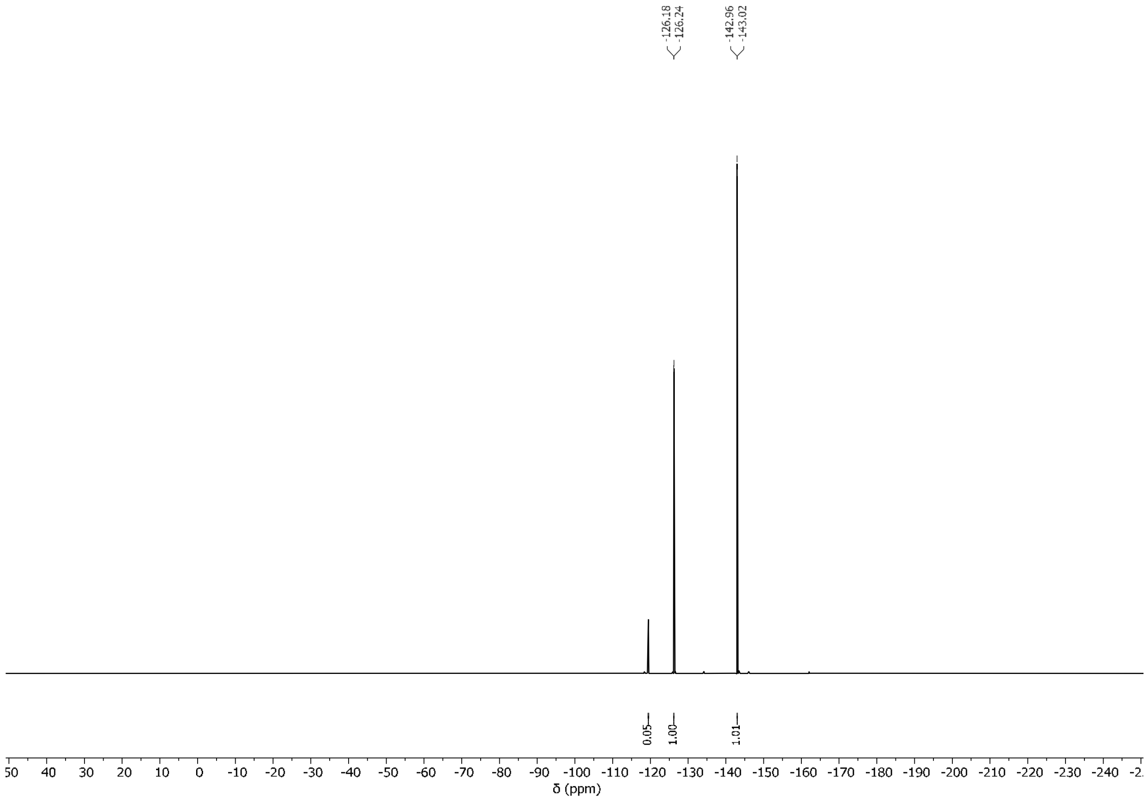

-126.18
-126.24
-142.96
-143.02
0.05
1.00
1.01
δ (ppm)

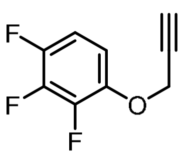
F
F
F
O

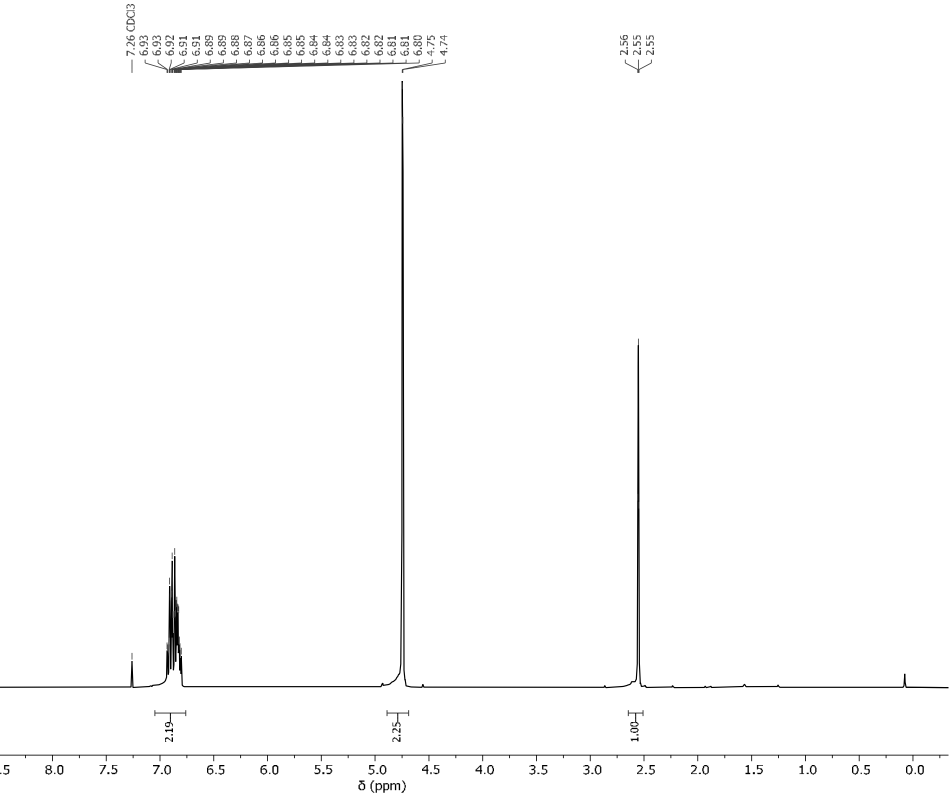
7.26 CDCl3
6.93
6.93
6.92
6.91
6.91
6.89
6.89
6.88
6.87
6.86
6.86
6.85
6.85
6.84
6.84
6.83
6.83
6.82
6.82
6.81
6.81
6.80
4.75
4.74
2.56
2.55
2.55
2.19
2.25
1.00
10.0 9.5 9.0 8.5 8.0 7.5 7.0 6.5 6.0 5.5 5.0 4.5 4.0 3.5 3.0 2.5 2.0 1.5 1.0 0.5 0.0
δ (ppm)

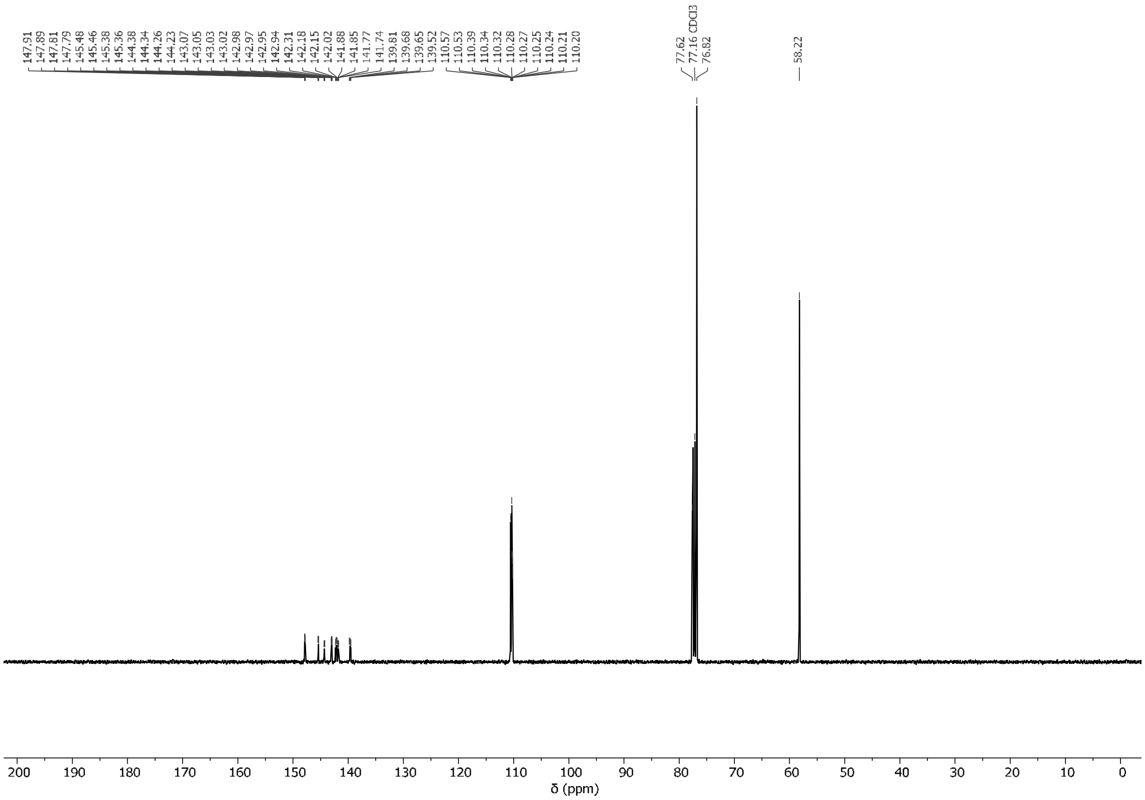
147.91
147.89
147.81
147.79
145.48
145.46
145.38
145.36
144.38
144.34
144.26
144.23
143.07
143.05
143.03
143.02
142.98
142.97
142.95
142.94
142.31
142.18
142.15
142.02
141.88
141.85
141.77
141.74
139.81
139.68
139.65
139.52
110.57
110.53
110.39
110.34
110.32
110.28
110.27
110.25
110.24
110.21
110.20
77.62
77.16 CDCl3
76.82
58.22
200 190 180 170 160 150 140 130 120 110 100 90 80 70 60 50 40 30 20 10 0
δ (ppm)

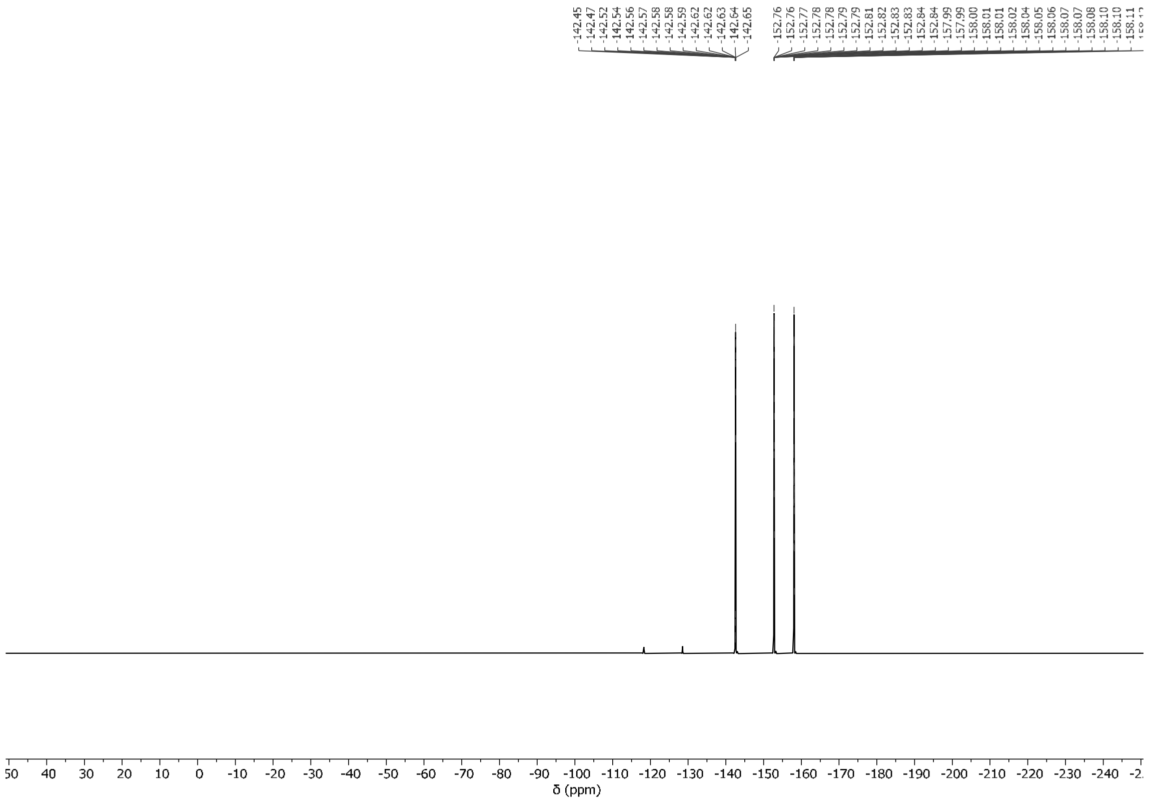

-142.45 -142.47 -142.52 -142.54 -142.56 -142.57 -142.58 -142.58 -142.59 -142.62 -142.62 -142.63 -142.64 -142.65
-152.76 -152.76 -152.77 -152.78 -152.78 -152.79 -152.79 -152.81 -152.82 -152.83 -152.84 -152.84 -157.99 -157.99 -158.00 -158.01 -158.01 -158.02 -158.04 -158.05 -158.06 -158.07 -158.07 -158.08 -158.10 -158.10 -158.11
40 30 20 10 0 -10 -20 -30 -40 -50 -60 -70 -80 -90 -100 -110 -120 -130 -140 -150 -160 -170 -180 -190 -200 -210 -220 -230 -240
δ (ppm)

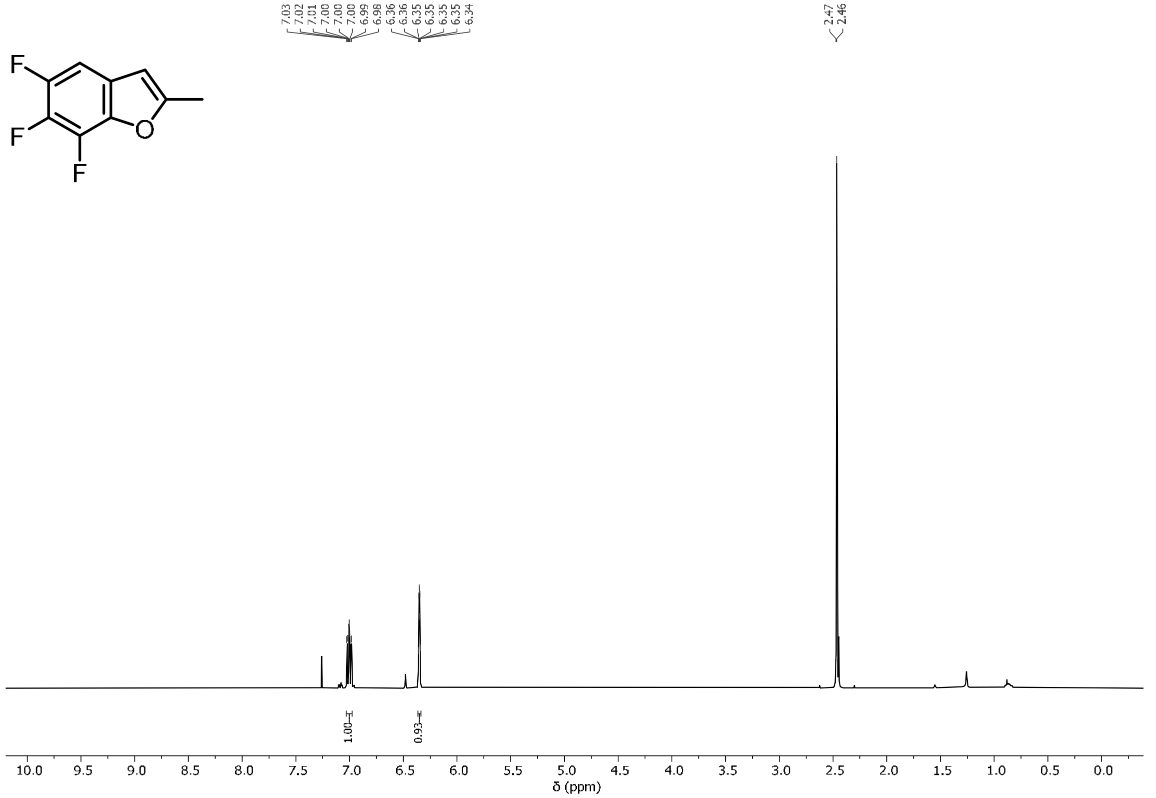

F
F
F
O
7.03 7.02 7.01 7.00 7.00 6.99 6.98 6.36 6.36 6.35 6.35 6.35 6.34
2.47 2.46
1.00
0.93
10.0 9.5 9.0 8.5 8.0 7.5 7.0 6.5 6.0 5.5 5.0 4.5 4.0 3.5 3.0 2.5 2.0 1.5 1.0 0.5 0.0
δ (ppm)

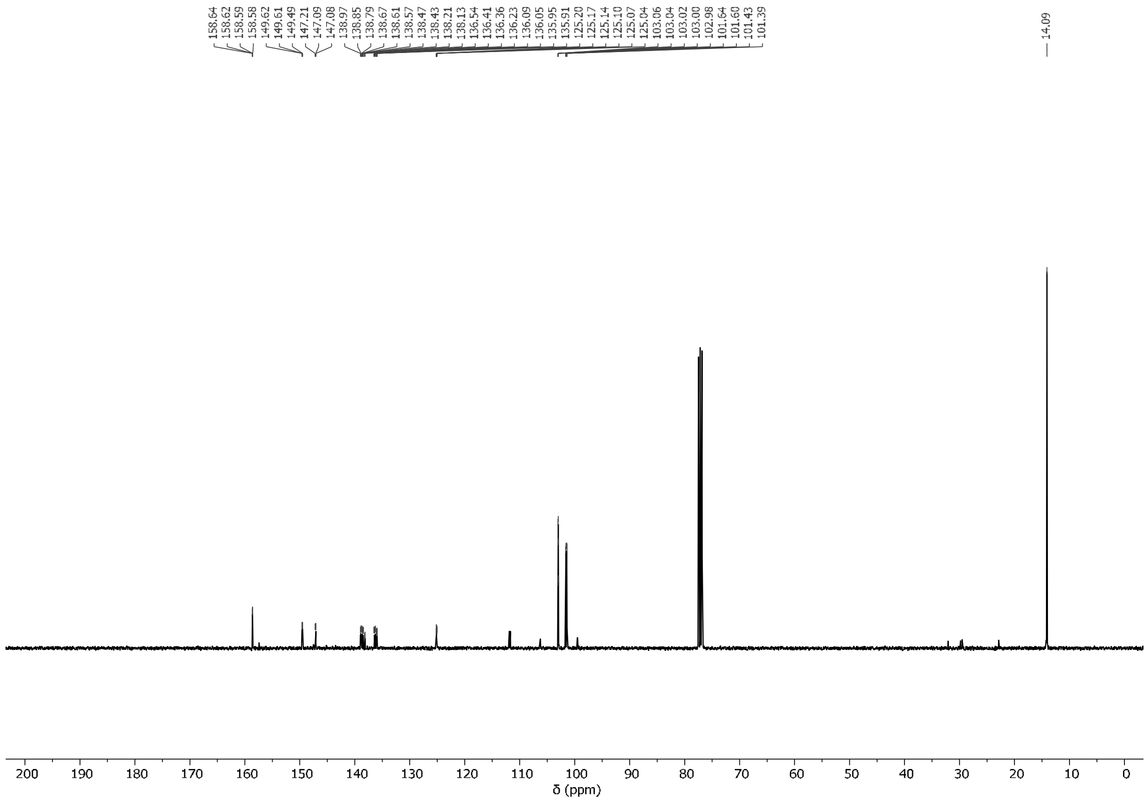
158.64
158.62
158.59
158.58
149.62
149.61
149.49
147.21
147.09
147.08
138.97
138.85
138.79
138.67
138.61
138.57
138.47
138.43
138.21
138.13
136.54
136.41
136.36
136.23
136.09
136.05
135.95
135.91
125.20
125.17
125.14
125.10
125.07
125.04
103.06
103.04
103.02
103.00
102.98
101.64
101.60
101.43
101.39
14.09
200 190 180 170 160 150 140 130 120 110 100 90 80 70 60 50 40 30 20 10 0
δ (ppm)

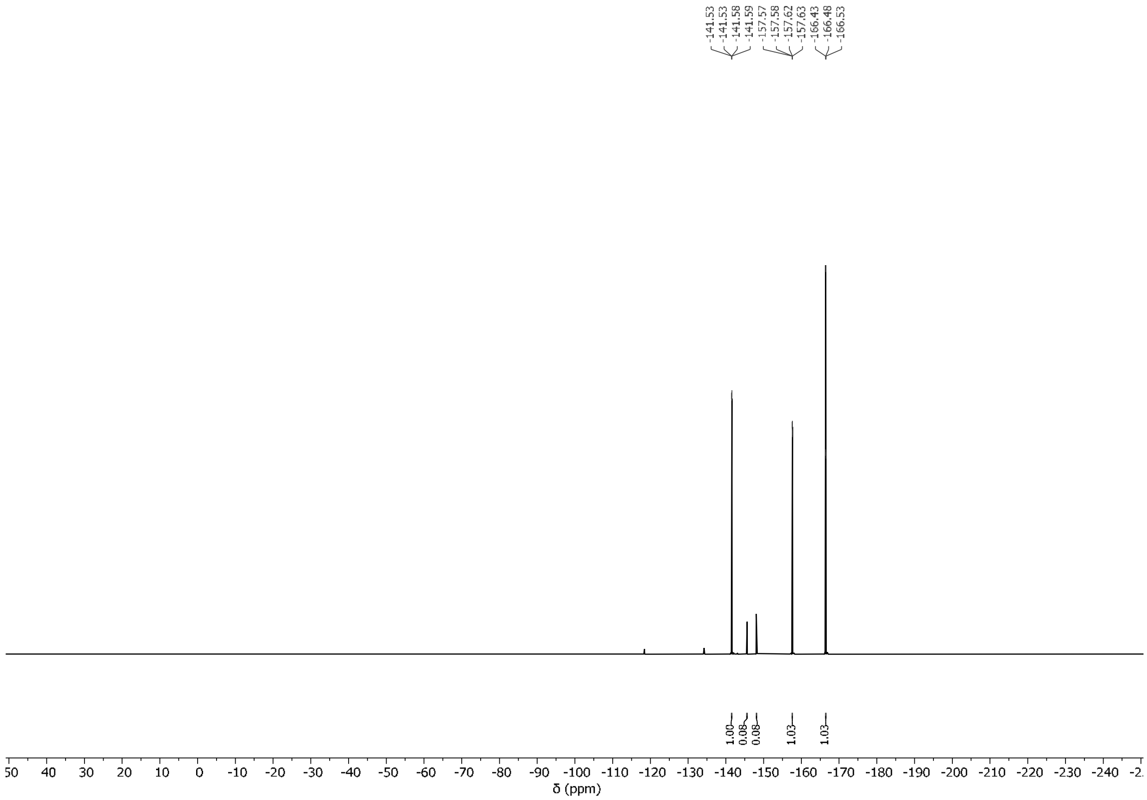
-141.53
-141.53
-141.58
-141.59
-157.57
-157.58
-157.62
-157.63
-166.43
-166.48
-166.53
1.00
0.08
0.08
1.03
1.03
50 40 30 20 10 0 -10 -20 -30 -40 -50 -60 -70 -80 -90 -100 -110 -120 -130 -140 -150 -160 -170 -180 -190 -200 -210 -220 -230 -240
δ (ppm)

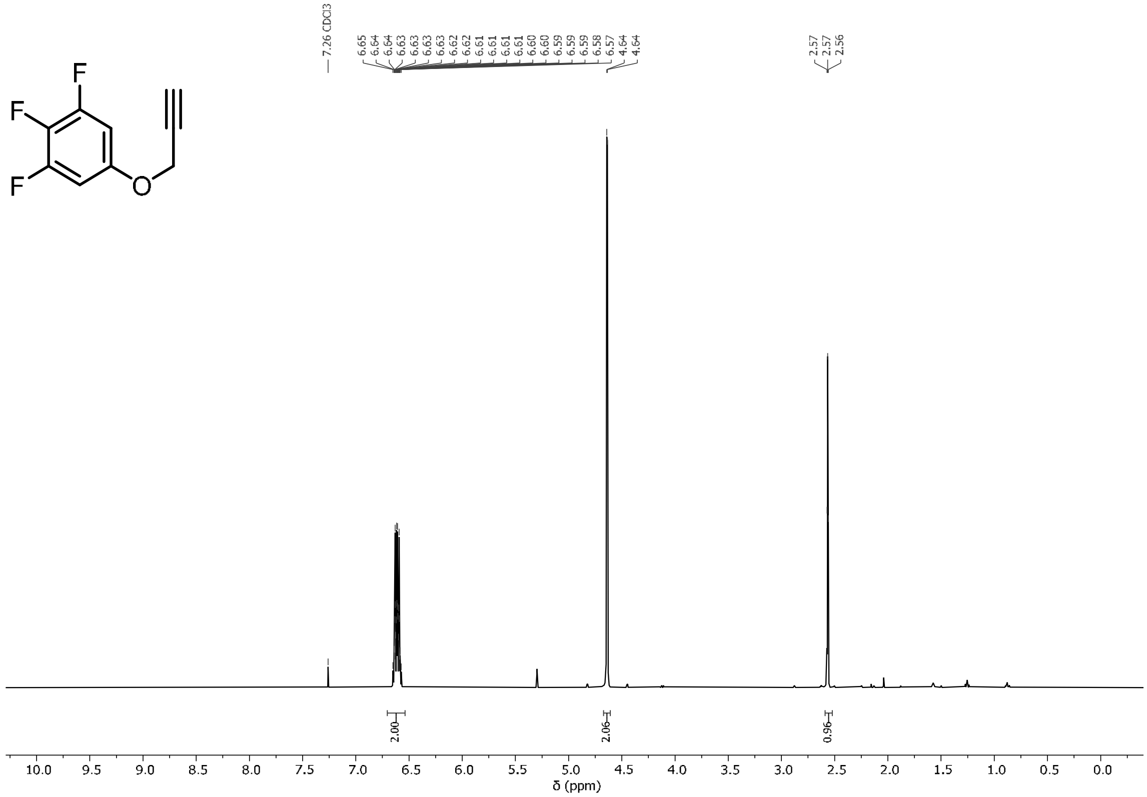
F
F
F
O
7.26 CDCl3
6.65
6.64
6.64
6.63
6.63
6.63
6.63
6.62
6.62
6.61
6.61
6.61
6.61
6.60
6.60
6.59
6.59
6.59
6.58
6.57
4.64
4.64
2.57
2.57
2.56
2.00
2.06
0.96
10.0 9.5 9.0 8.5 8.0 7.5 7.0 6.5 6.0 5.5 5.0 4.5 4.0 3.5 3.0 2.5 2.0 1.5 1.0 0.5 0.0
δ (ppm)

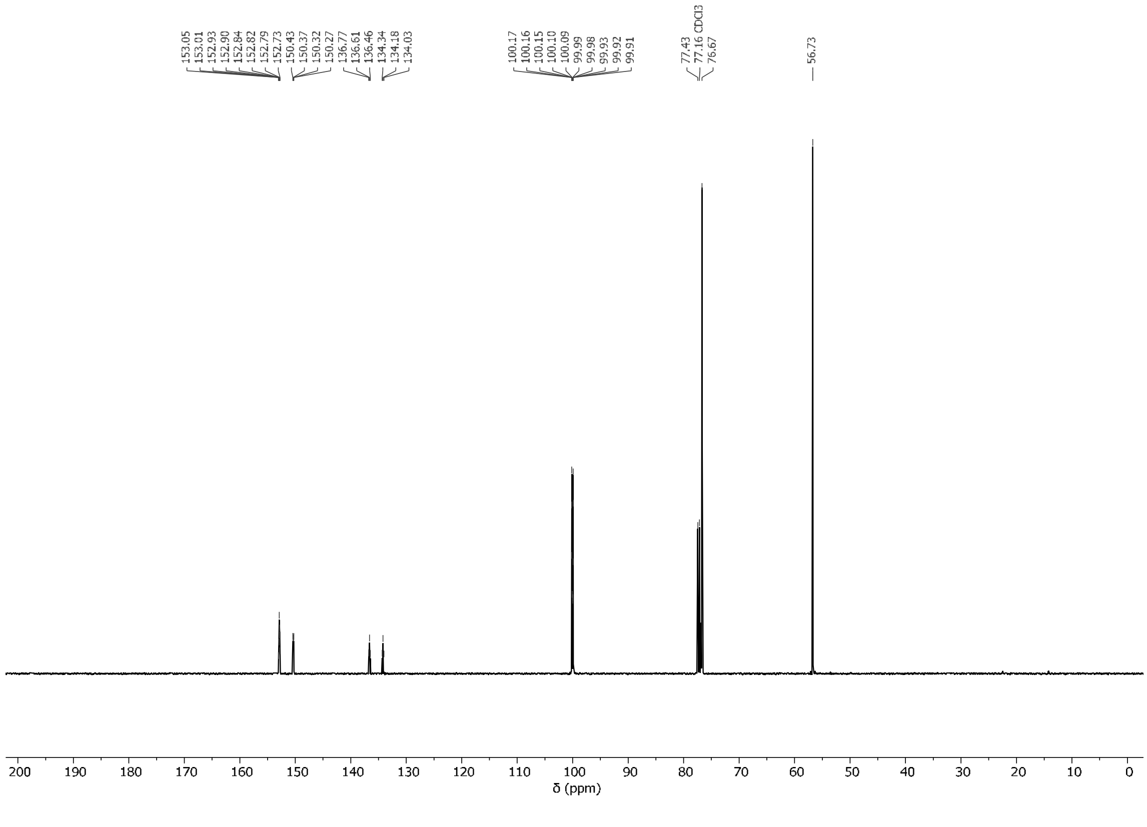
153.05
153.01
152.93
152.90
152.84
152.82
152.79
152.73
150.43
150.37
150.32
150.27
136.77
136.61
136.46
134.34
134.18
134.03
100.17
100.16
100.15
100.10
100.09
99.99
99.98
99.93
99.92
99.91
77.43
77.16 CDCl3
76.67
56.73
200 190 180 170 160 150 140 130 120 110 100 90 80 70 60 50 40 30 20 10 0
δ (ppm)

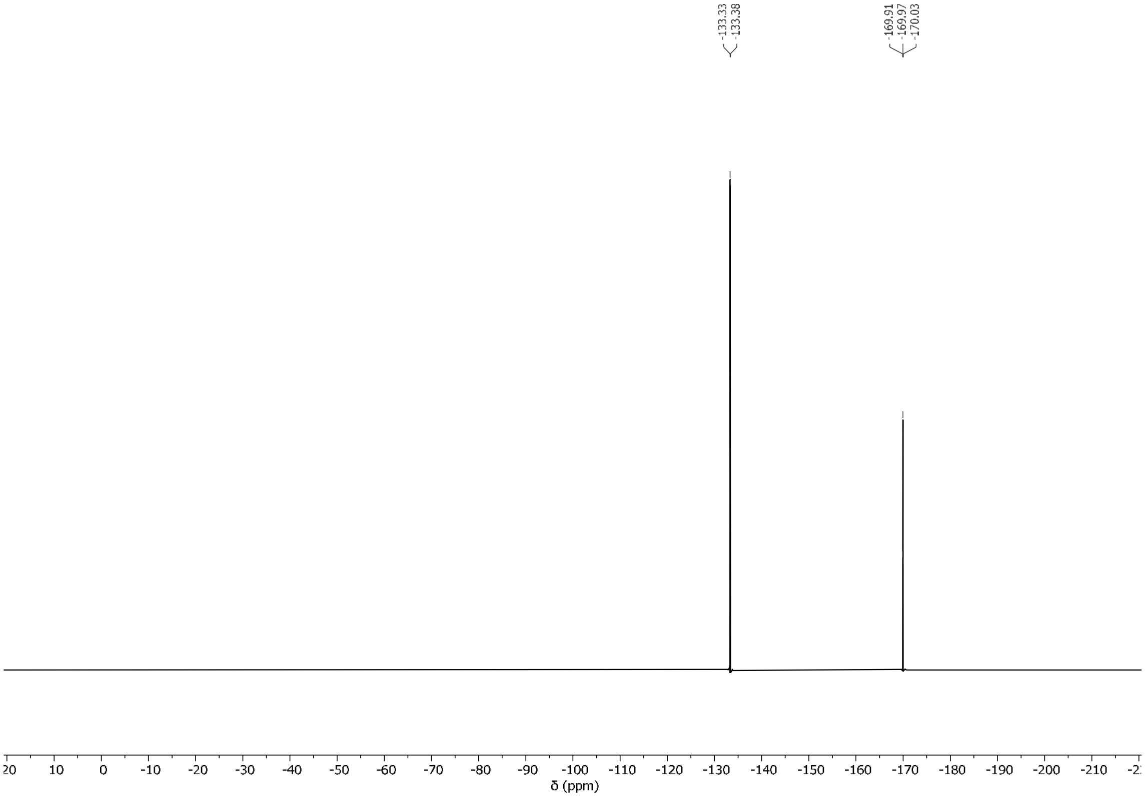

-133.33
-133.38
-169.91
-169.97
-170.03
20 10 0 -10 -20 -30 -40 -50 -60 -70 -80 -90 -100 -110 -120 -130 -140 -150 -160 -170 -180 -190 -200 -210
δ (ppm)

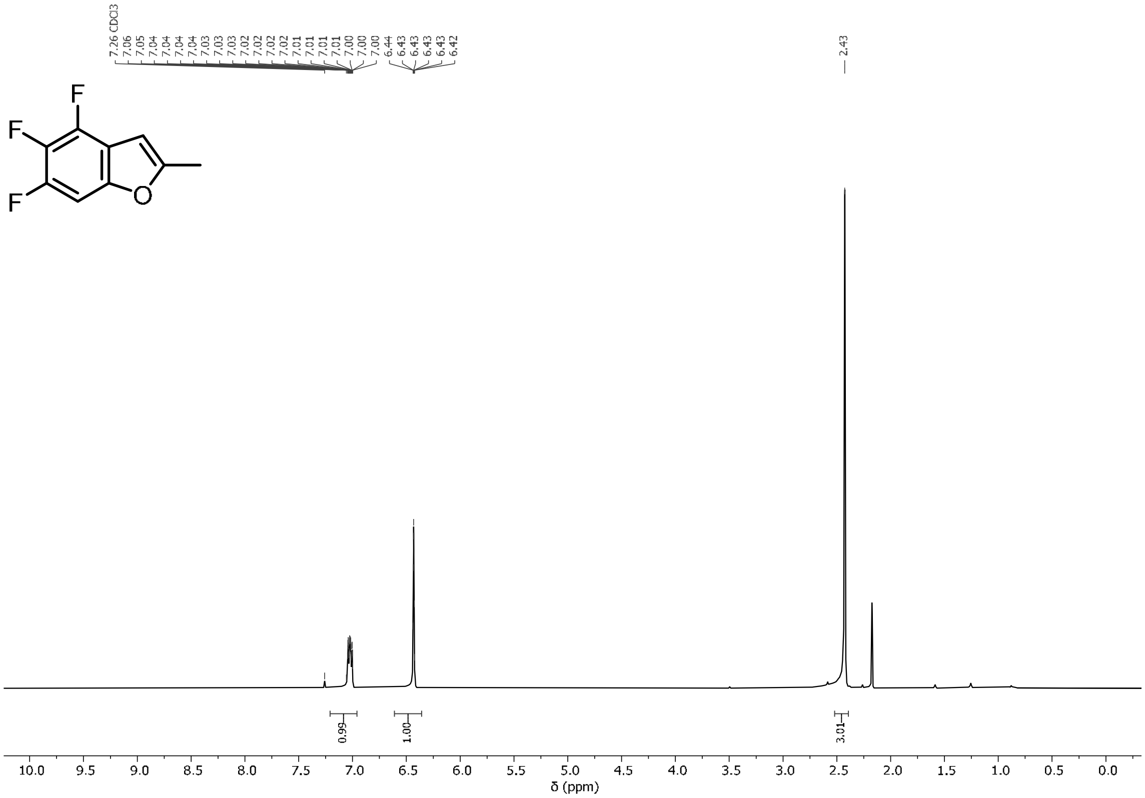

7.26 CDCl3
7.06
7.05
7.04
7.04
7.04
7.03
7.03
7.02
7.02
7.02
7.01
7.01
7.01
7.00
7.00
6.44
6.43
6.43
6.43
6.42
2.43
F
F
F
O
0.99
1.00
3.01
10.0 9.5 9.0 8.5 8.0 7.5 7.0 6.5 6.0 5.5 5.0 4.5 4.0 3.5 3.0 2.5 2.0 1.5 1.0 0.5 0.0
δ (ppm)

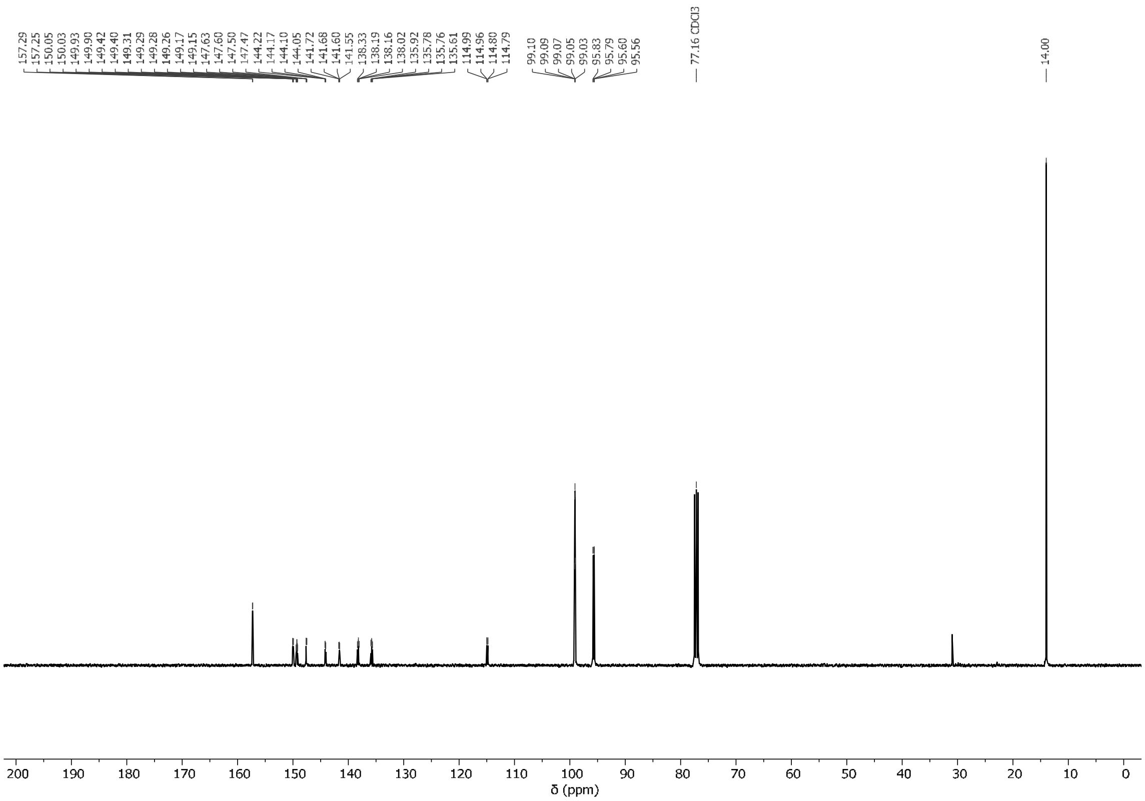
157.29
157.25
150.05
150.03
149.93
149.90
149.42
149.40
149.31
149.29
149.28
149.26
149.17
149.15
147.63
147.60
147.50
147.47
144.22
144.17
144.10
144.05
141.72
141.68
141.60
141.55
138.33
138.19
138.16
138.02
135.92
135.78
135.76
135.61
114.99
114.96
114.80
114.79
99.10
99.09
99.07
99.05
99.03
95.83
95.79
95.60
95.56
77.16 CDCl3
14.00
200 190 180 170 160 150 140 130 120 110 100 90 80 70 60 50 40 30 20 10 0
δ (ppm)

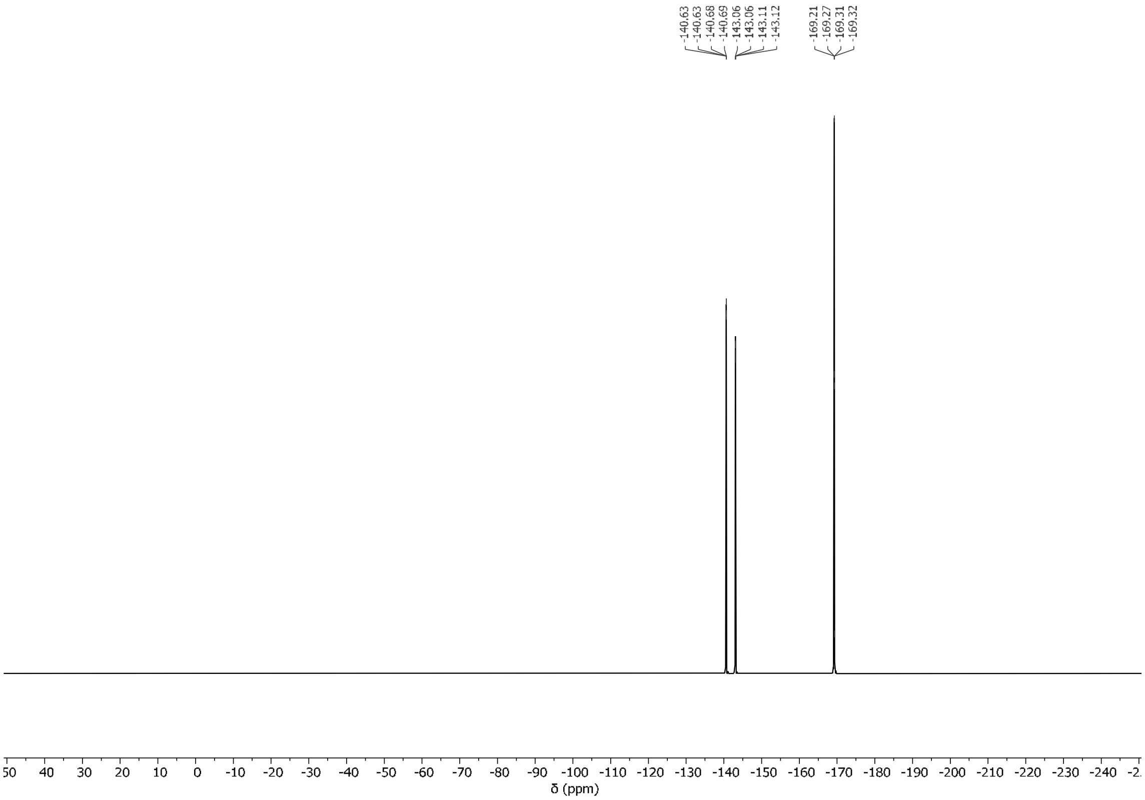
-140.63
-140.63
-140.68
-140.69
-143.06
-143.06
-143.11
-143.12
-169.21
-169.27
-169.31
-169.32
50 40 30 20 10 0 -10 -20 -30 -40 -50 -60 -70 -80 -90 -100 -110 -120 -130 -140 -150 -160 -170 -180 -190 -200 -210 -220 -230 -240
δ (ppm)

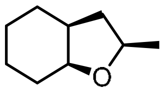

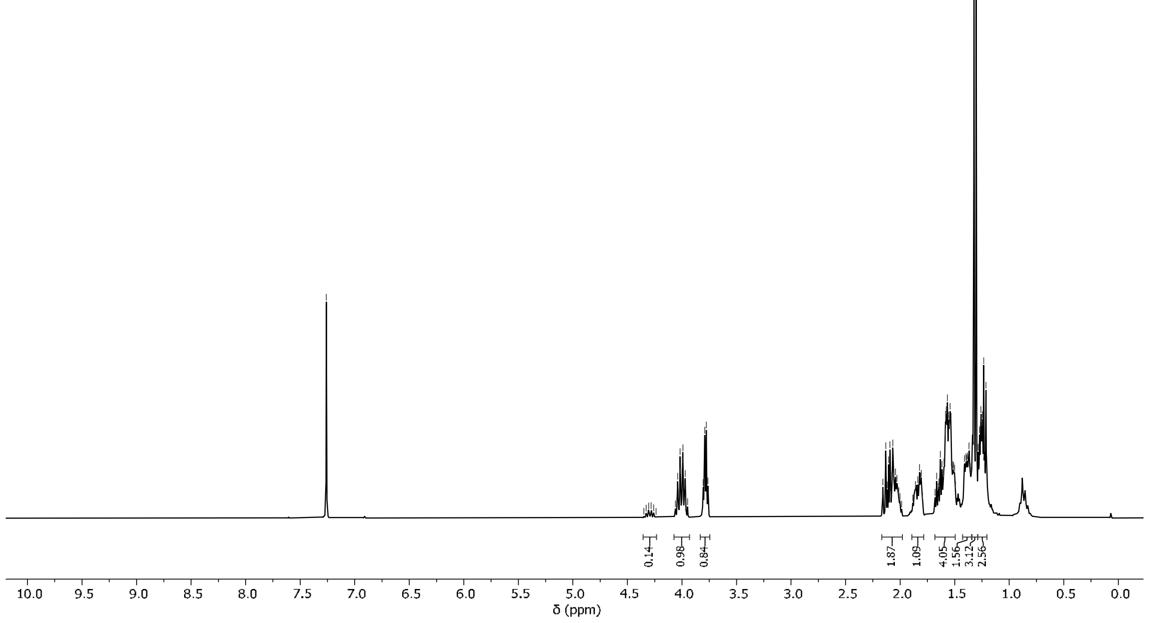
7.26 CDCl3
4.35
4.33
4.30
4.28
4.26
4.24
4.06
4.04
4.03
4.02
4.01
4.00
3.99
3.98
3.97
3.95
3.81
3.79
3.78
3.76
2.16
2.13
2.12
2.11
2.09
2.09
2.08
2.07
2.06
2.04
2.03
2.01
2.00
1.99
1.88
1.86
1.84
1.82
1.81
1.81
1.79
1.68
1.67
1.65
1.65
1.63
1.62
1.60
1.59
1.58
1.58
1.57
1.56
1.55
1.54
1.53
1.52
1.51
1.50
1.49
1.41
1.40
1.39
1.38
1.37
1.36
1.35
1.32
1.30
1.29
1.27
1.26
1.26
1.25
1.25
1.23
1.22
1.21
0.14
0.98
0.84
1.87
1.09
4.05
1.56
3.12
2.56
10.0 9.5 9.0 8.5 8.0 7.5 7.0 6.5 6.0 5.5 5.0 4.5 4.0 3.5 3.0 2.5 2.0 1.5 1.0 0.5 0.0
δ (ppm)

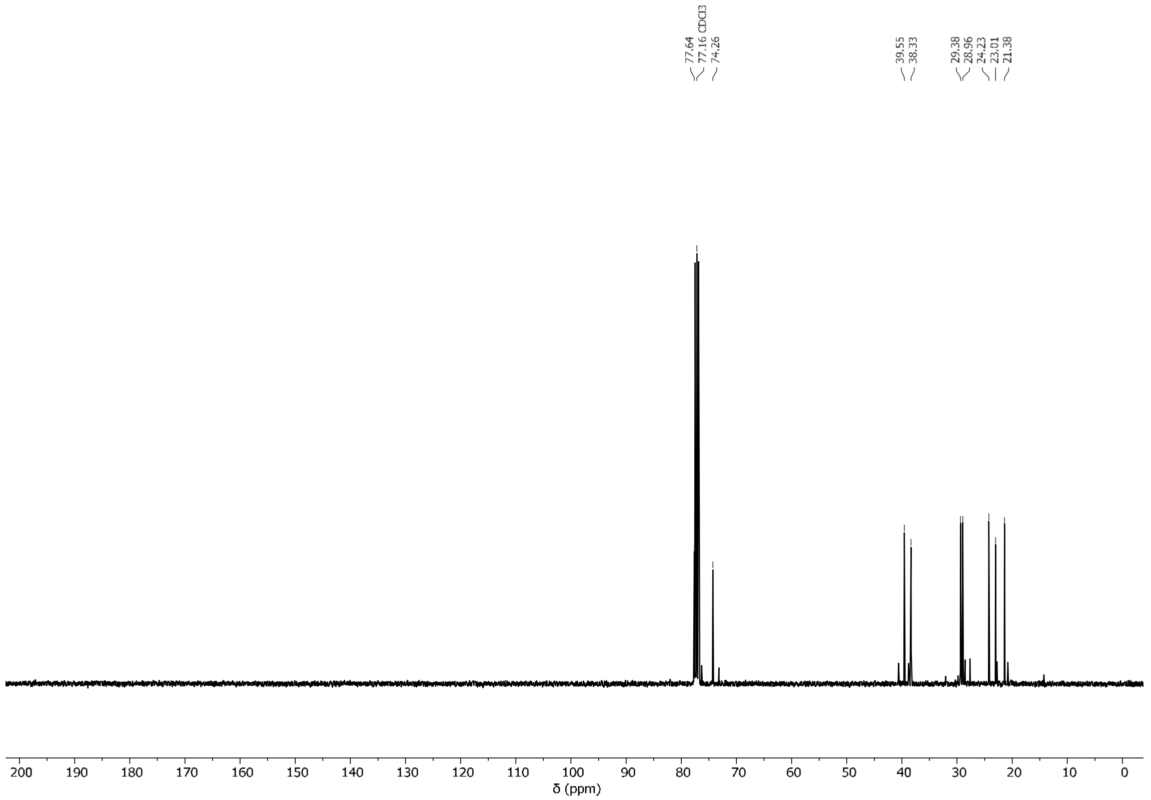
77.64
77.16 CDCl3
74.26
39.55
38.33
29.38
28.96
24.23
23.01
21.38
200 190 180 170 160 150 140 130 120 110 100 90 80 70 60 50 40 30 20 10 0
δ (ppm)

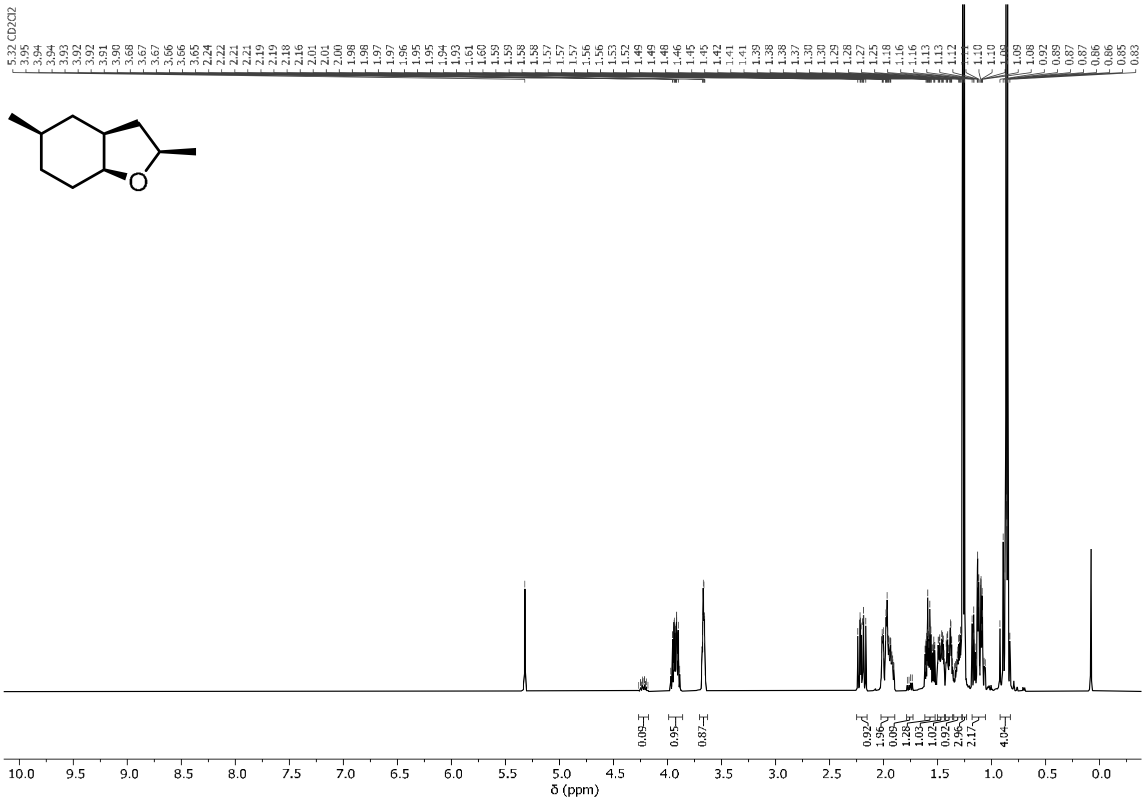
5.32 CD2Cl2
3.95 3.94 3.94 3.93 3.92 3.92 3.91 3.90 3.68 3.67 3.67 3.66 3.66 3.65 2.24 2.22 2.21 2.21 2.19 2.18 2.16 2.01 2.01 2.00 1.98 1.98 1.97 1.97 1.96 1.95 1.95 1.94 1.93 1.61 1.60 1.59 1.59 1.58 1.58 1.57 1.57 1.57 1.56 1.56 1.53 1.52 1.49 1.49 1.48 1.46 1.45 1.45 1.42 1.41 1.41 1.39 1.38 1.38 1.37 1.30 1.30 1.29 1.28 1.27 1.25 1.18 1.16 1.16 1.13 1.13 1.12 1.11 1.10 1.10 1.09 1.09 1.08 0.92 0.89 0.87 0.87 0.86 0.85 0.83
0.09
0.95
0.87
0.92
1.96
0.09
1.28
1.03
1.02
0.92
2.96
2.17
4.04
10.0 9.5 9.0 8.5 8.0 7.5 7.0 6.5 6.0 5.5 5.0 4.5 4.0 3.5 3.0 2.5 2.0 1.5 1.0 0.5 0.0
δ (ppm)

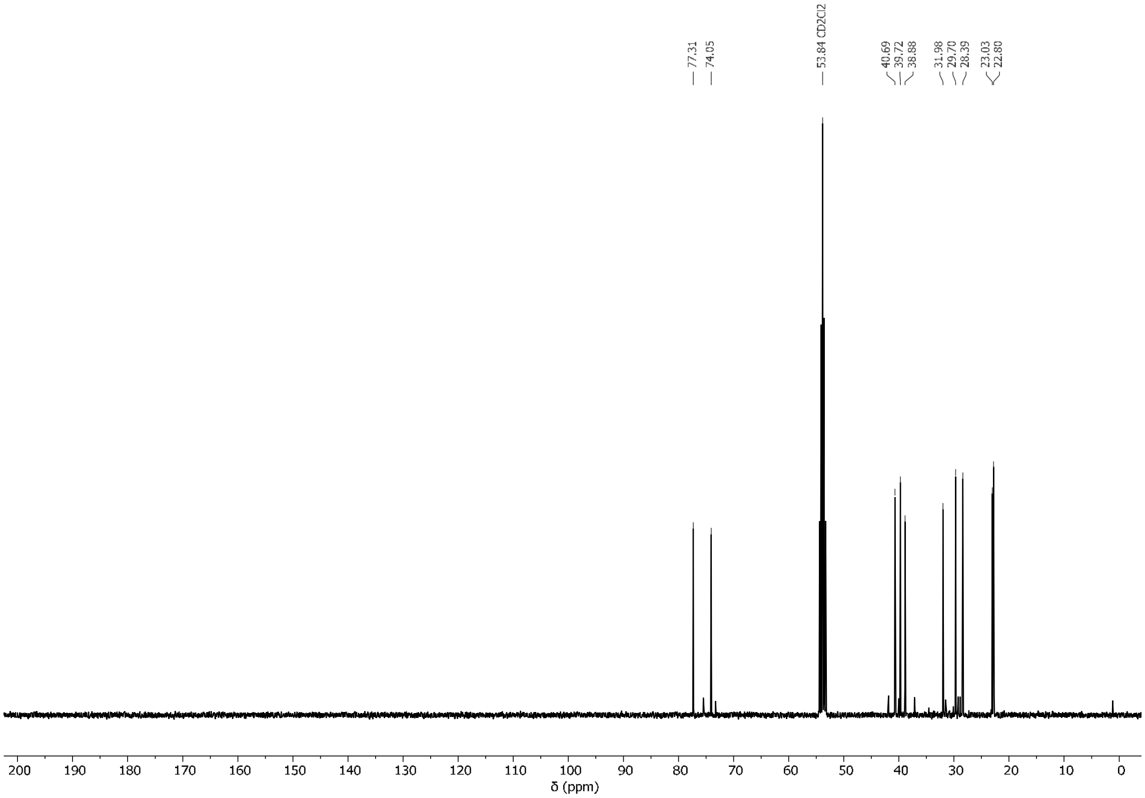
77.31
74.05
53.84 CD2Cl2
40.69
39.72
38.88
31.98
29.70
28.39
23.03
22.80
200 190 180 170 160 150 140 130 120 110 100 90 80 70 60 50 40 30 20 10 0
δ (ppm)

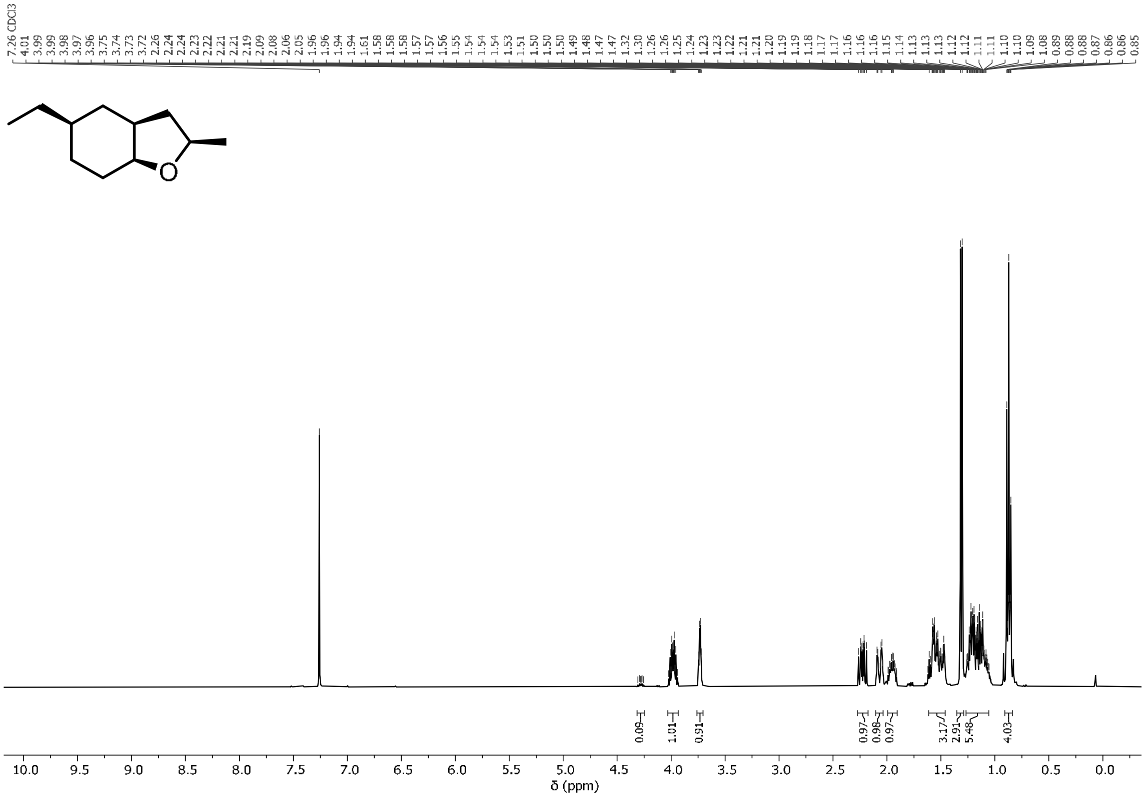
7.26 CDCl3
4.01
3.99
3.99
3.98
3.97
3.96
3.75
3.74
3.73
3.72
2.26
2.24
2.24
2.23
2.22
2.21
2.21
2.19
2.09
2.08
2.06
2.05
1.96
1.96
1.94
1.94
1.61
1.58
1.58
1.57
1.57
1.56
1.55
1.54
1.54
1.54
1.53
1.51
1.50
1.50
1.50
1.49
1.48
1.47
1.47
1.32
1.30
1.26
1.25
1.24
1.23
1.23
1.22
1.21
1.21
1.21
1.20
1.19
1.19
1.18
1.17
1.17
1.16
1.16
1.16
1.15
1.14
1.13
1.13
1.13
1.12
1.12
1.11
1.11
1.11
1.10
1.10
1.09
1.08
0.89
0.88
0.88
0.87
0.86
0.85
O
0.09
1.01
0.91
0.97
0.98
0.97
3.17
2.91
5.48
4.03
10.0
9.5
9.0
8.5
8.0
7.5
7.0
6.5
6.0
5.5
5.0
4.5
4.0
3.5
3.0
2.5
2.0
1.5
1.0
0.5
0.0
δ (ppm)

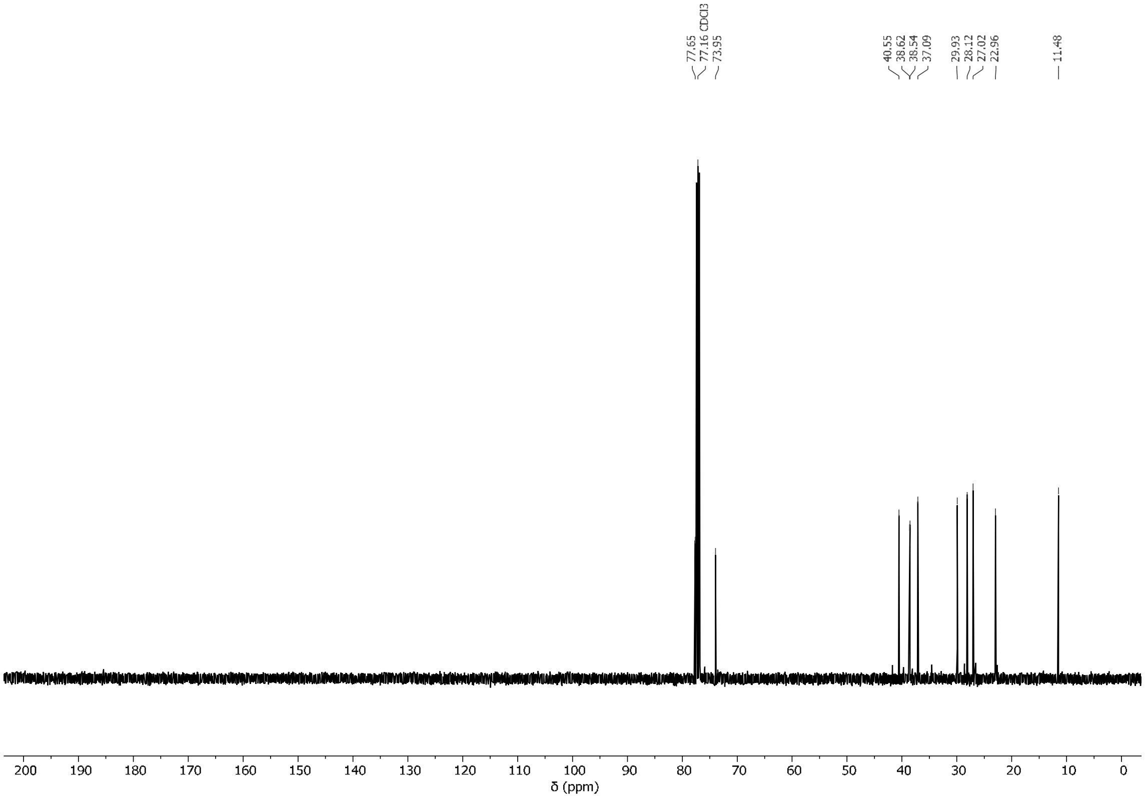
77.65
77.16 CDCl3
73.55
40.55
38.62
38.54
37.09
29.93
28.12
27.02
22.96
11.48
200
190
180
170
160
150
140
130
120
110
100
90
80
70
60
50
40
30
20
10
0
δ (ppm)

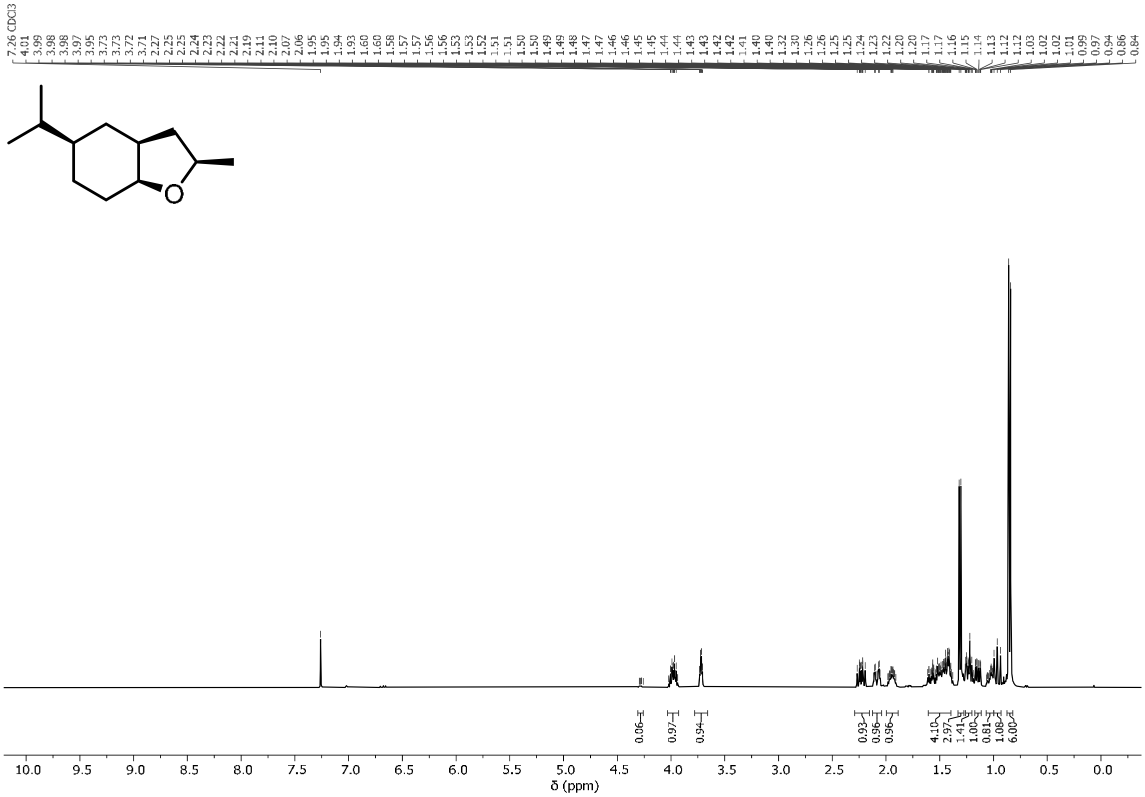
7.26 CDCl3
4.01
3.99
3.98
3.98
3.97
3.95
3.73
3.73
3.72
3.72
3.71
2.27
2.25
2.25
2.24
2.23
2.22
2.21
2.19
2.11
2.10
2.07
2.06
1.95
1.95
1.94
1.93
1.60
1.60
1.58
1.57
1.57
1.56
1.56
1.53
1.53
1.52
1.51
1.51
1.50
1.50
1.49
1.49
1.48
1.47
1.47
1.46
1.46
1.45
1.45
1.44
1.44
1.43
1.43
1.42
1.42
1.41
1.40
1.40
1.32
1.30
1.26
1.26
1.25
1.25
1.24
1.23
1.22
1.20
1.20
1.17
1.17
1.16
1.15
1.14
1.13
1.12
1.12
1.03
1.02
1.02
1.01
0.99
0.97
0.94
0.86
0.84
O
0.06
0.97
0.94
0.93
0.96
0.96
4.10
2.97
1.41
1.00
0.81
1.08
6.00
10.0 9.5 9.0 8.5 8.0 7.5 7.0 6.5 6.0 5.5 5.0 4.5 4.0 3.5 3.0 2.5 2.0 1.5 1.0 0.5 0.0
δ (ppm)

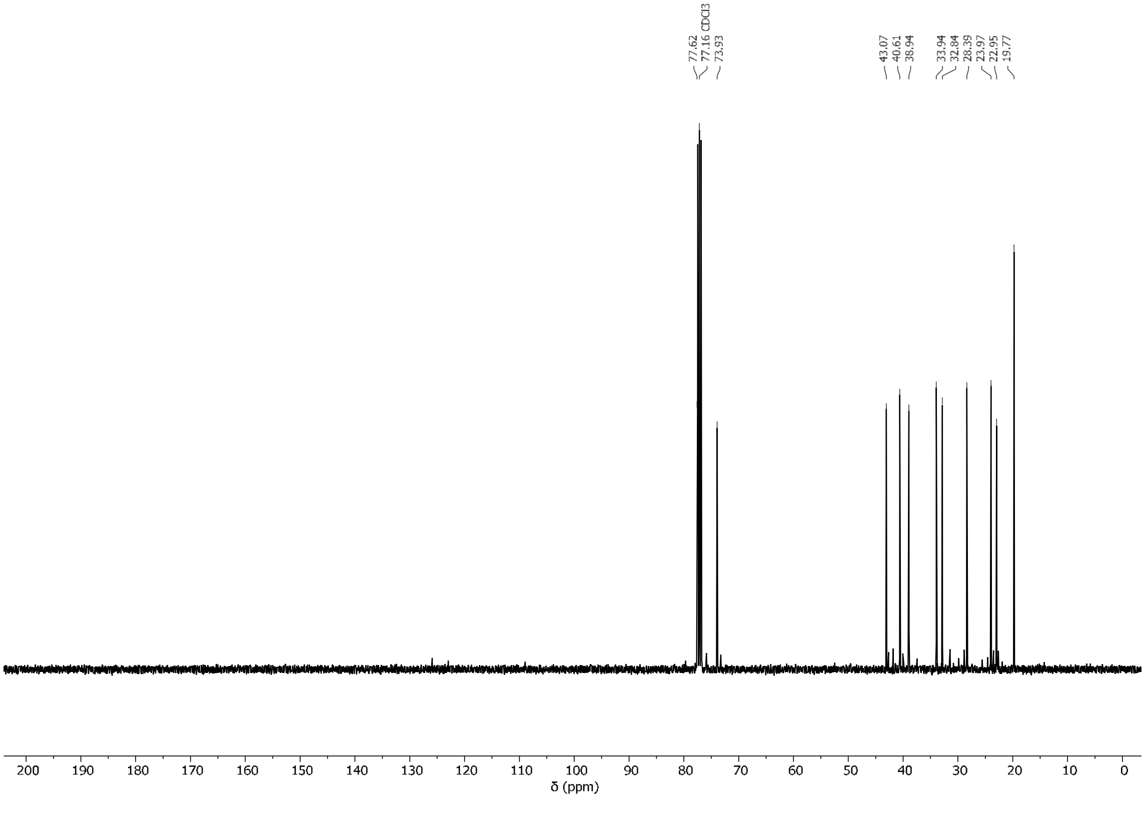
77.62
77.16 CDCl3
73.93
43.07
40.61
38.94
33.94
32.84
28.39
23.97
22.95
19.77
200 190 180 170 160 150 140 130 120 110 100 90 80 70 60 50 40 30 20 10 0
δ (ppm)

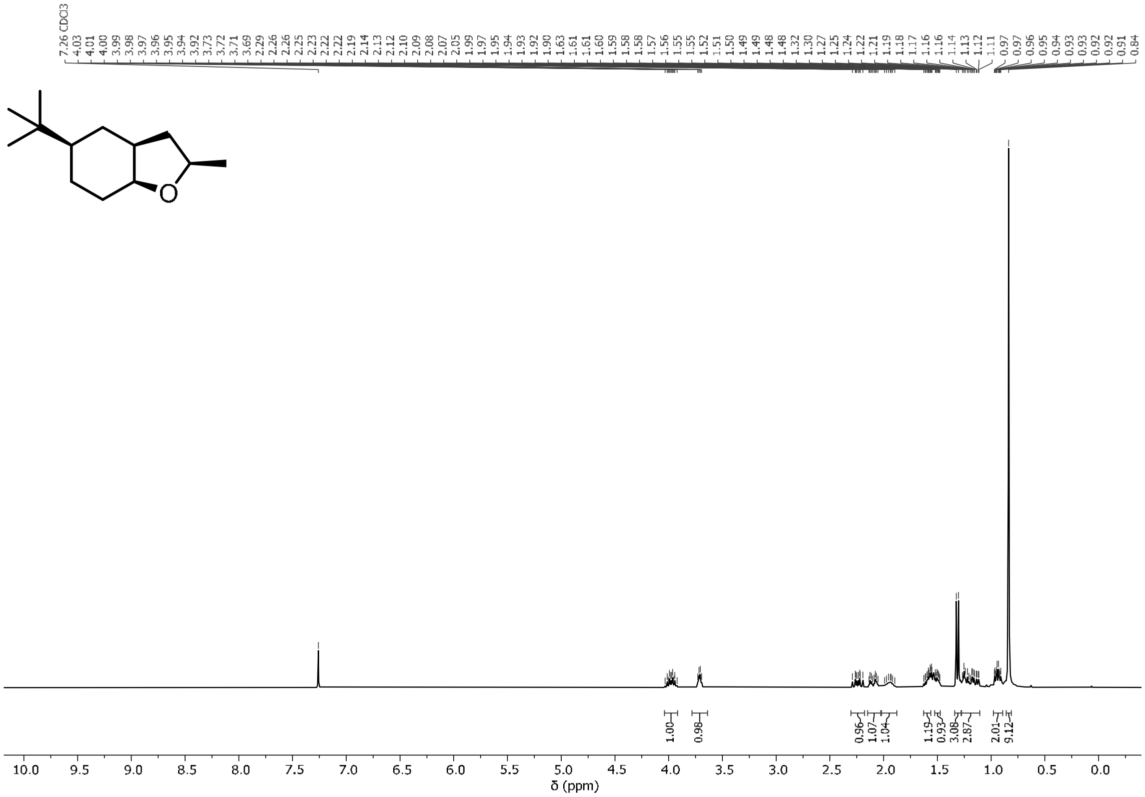
7.26 CDCl3
4.03
4.01
4.00
3.99
3.98
3.97
3.96
3.95
3.94
3.92
3.73
3.72
3.71
3.69
2.29
2.26
2.26
2.25
2.23
2.22
2.22
2.19
2.14
2.13
2.12
2.10
2.09
2.08
2.07
2.05
1.99
1.97
1.95
1.94
1.93
1.92
1.90
1.63
1.61
1.61
1.60
1.59
1.58
1.58
1.57
1.56
1.55
1.55
1.52
1.51
1.50
1.49
1.49
1.48
1.48
1.32
1.30
1.27
1.25
1.24
1.22
1.21
1.19
1.18
1.17
1.16
1.16
1.14
1.13
1.12
1.11
0.97
0.97
0.96
0.95
0.94
0.93
0.93
0.92
0.92
0.91
0.84
1.00
0.98
0.96
1.07
1.04
1.19
0.93
3.08
2.87
2.01
9.12
10.0 9.5 9.0 8.5 8.0 7.5 7.0 6.5 6.0 5.5 5.0 4.5 4.0 3.5 3.0 2.5 2.0 1.5 1.0 0.5 0.0
δ (ppm)

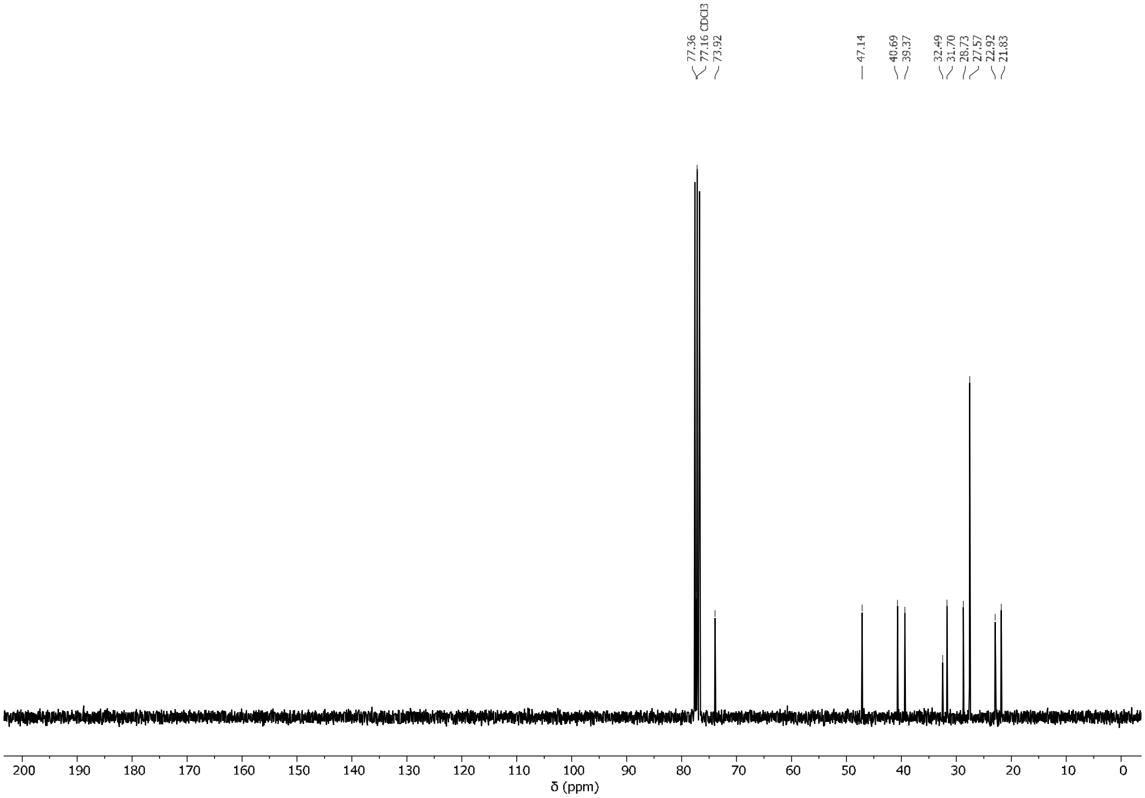
77.36
77.16 CDCl3
73.92
47.14
40.69
39.37
32.49
31.70
28.73
27.57
22.92
21.83
200 190 180 170 160 150 140 130 120 110 100 90 80 70 60 50 40 30 20 10 0
δ (ppm)

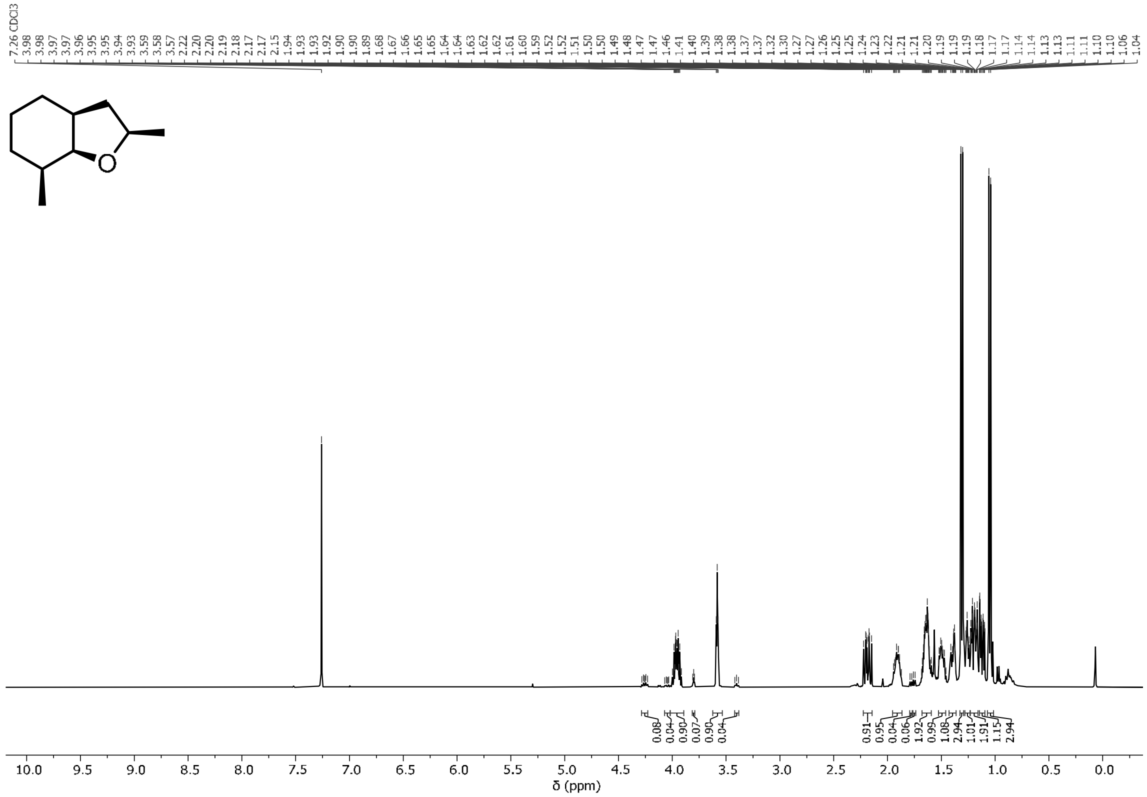
7.26 CDCl3
3.98
3.98
3.97
3.97
3.96
3.95
3.95
3.94
3.93
3.59
3.58
3.57
2.22
2.20
2.20
2.19
2.18
2.17
2.17
2.15
1.94
1.93
1.93
1.92
1.90
1.90
1.89
1.68
1.67
1.66
1.65
1.65
1.64
1.64
1.63
1.62
1.62
1.61
1.60
1.59
1.52
1.52
1.51
1.50
1.50
1.49
1.48
1.47
1.47
1.46
1.41
1.40
1.39
1.38
1.38
1.37
1.37
1.32
1.30
1.27
1.27
1.26
1.25
1.25
1.24
1.23
1.22
1.21
1.21
1.20
1.19
1.19
1.19
1.18
1.17
1.17
1.14
1.14
1.13
1.13
1.11
1.11
1.10
1.10
1.06
1.04
O
0.08
0.04
0.90
0.07
0.90
0.04
0.91
0.95
0.04
0.06
1.92
0.99
1.08
2.94
1.01
1.91
1.15
2.94
10.0 9.5 9.0 8.5 8.0 7.5 7.0 6.5 6.0 5.5 5.0 4.5 4.0 3.5 3.0 2.5 2.0 1.5 1.0 0.5 0.0
δ (ppm)

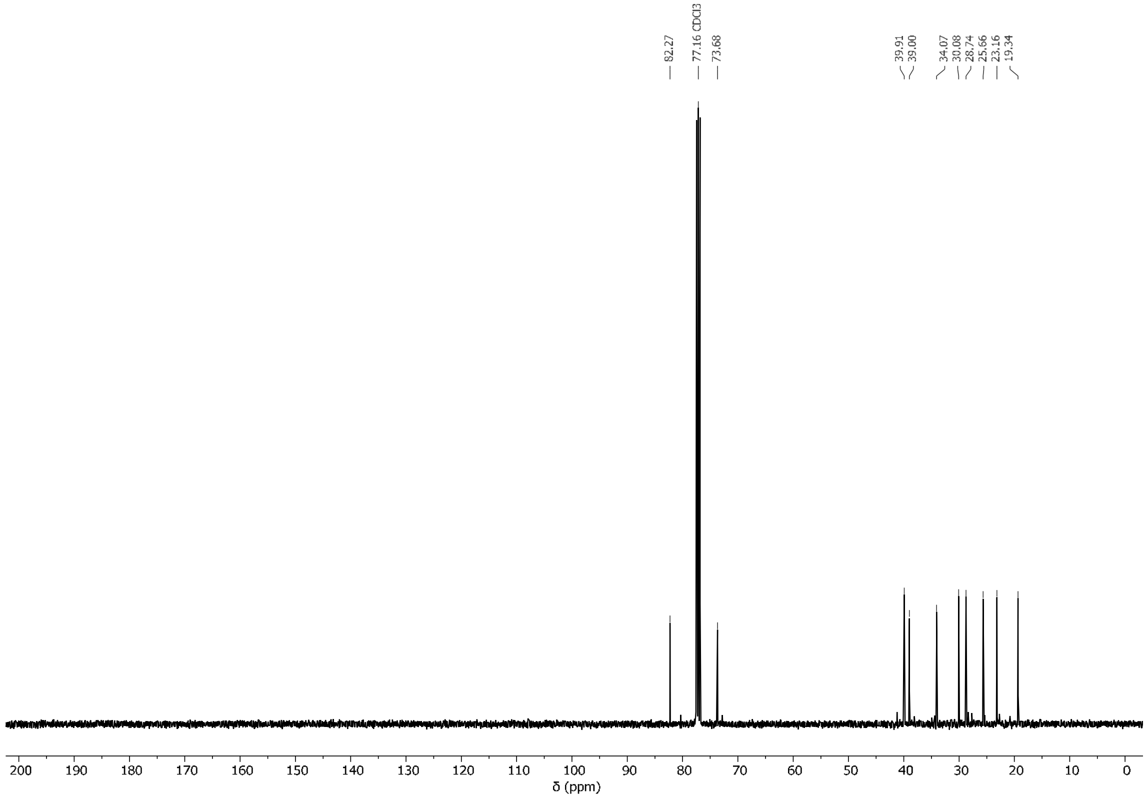
82.27
77.16 CDCl3
73.68
39.91
39.00
34.07
30.08
28.74
25.66
23.16
19.34
200 190 180 170 160 150 140 130 120 110 100 90 80 70 60 50 40 30 20 10 0
δ (ppm)

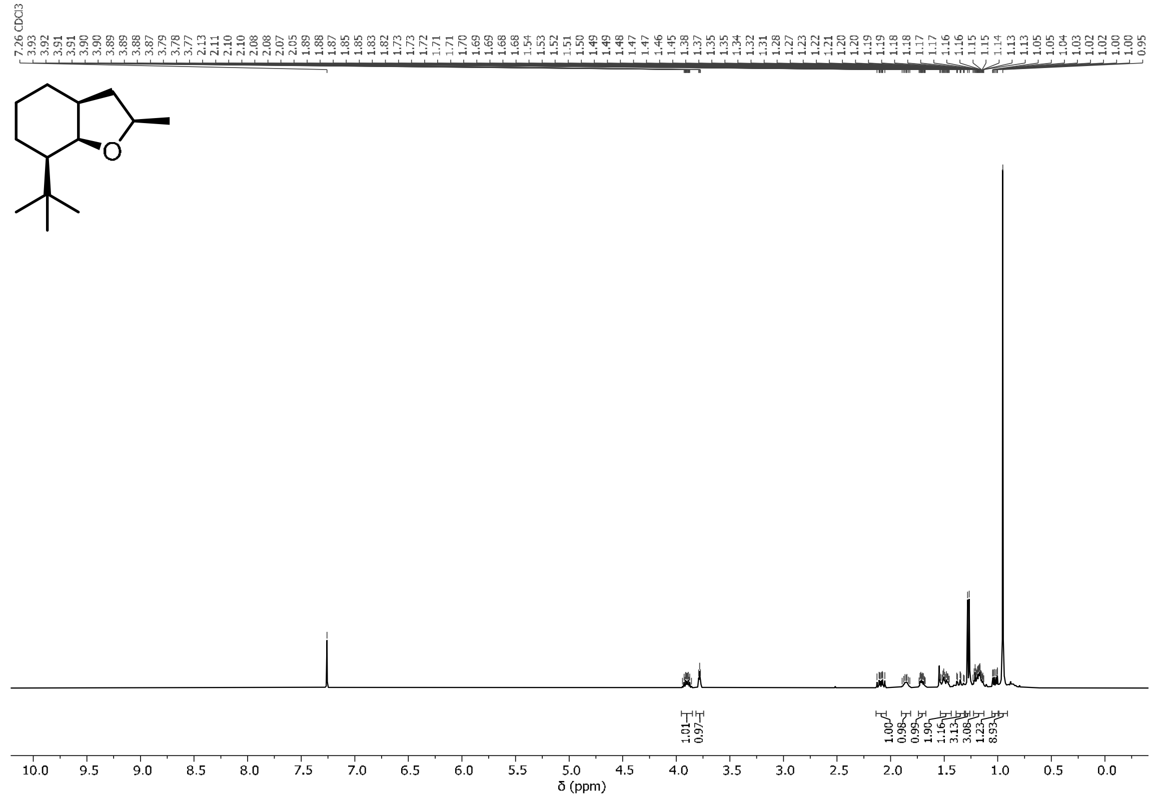
7.26 CDCl3
3.93
3.92
3.91
3.91
3.90
3.89
3.89
3.88
3.87
3.79
3.78
3.77
2.13
2.11
2.10
2.10
2.08
2.08
2.07
2.05
1.89
1.88
1.87
1.85
1.83
1.82
1.73
1.73
1.72
1.71
1.71
1.70
1.69
1.69
1.68
1.68
1.54
1.53
1.52
1.51
1.50
1.49
1.49
1.48
1.47
1.47
1.46
1.45
1.38
1.37
1.35
1.35
1.34
1.32
1.31
1.28
1.27
1.23
1.22
1.21
1.20
1.20
1.19
1.19
1.18
1.18
1.17
1.17
1.16
1.16
1.15
1.15
1.14
1.13
1.13
1.13
1.05
1.05
1.04
1.03
1.02
1.02
1.00
1.00
0.95
O
1.01
0.97
1.00
0.98
0.99
1.90
1.16
3.13
3.08
1.23
8.93
10.0 9.5 9.0 8.5 8.0 7.5 7.0 6.5 6.0 5.5 5.0 4.5 4.0 3.5 3.0 2.5 2.0 1.5 1.0 0.5 0.0
δ (ppm)

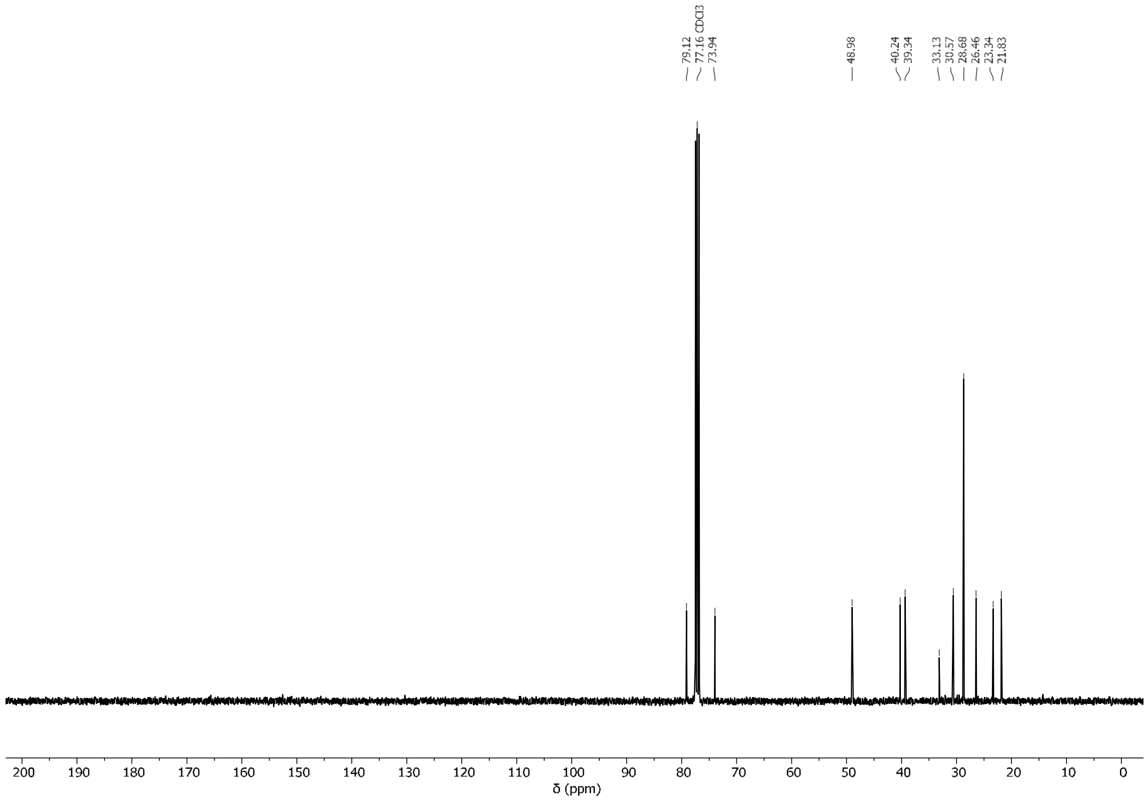
79.12
77.16 CDCl3
73.94
48.98
40.24
39.34
33.13
30.57
28.68
26.46
23.34
21.83
200 190 180 170 160 150 140 130 120 110 100 90 80 70 60 50 40 30 20 10 0
δ (ppm)

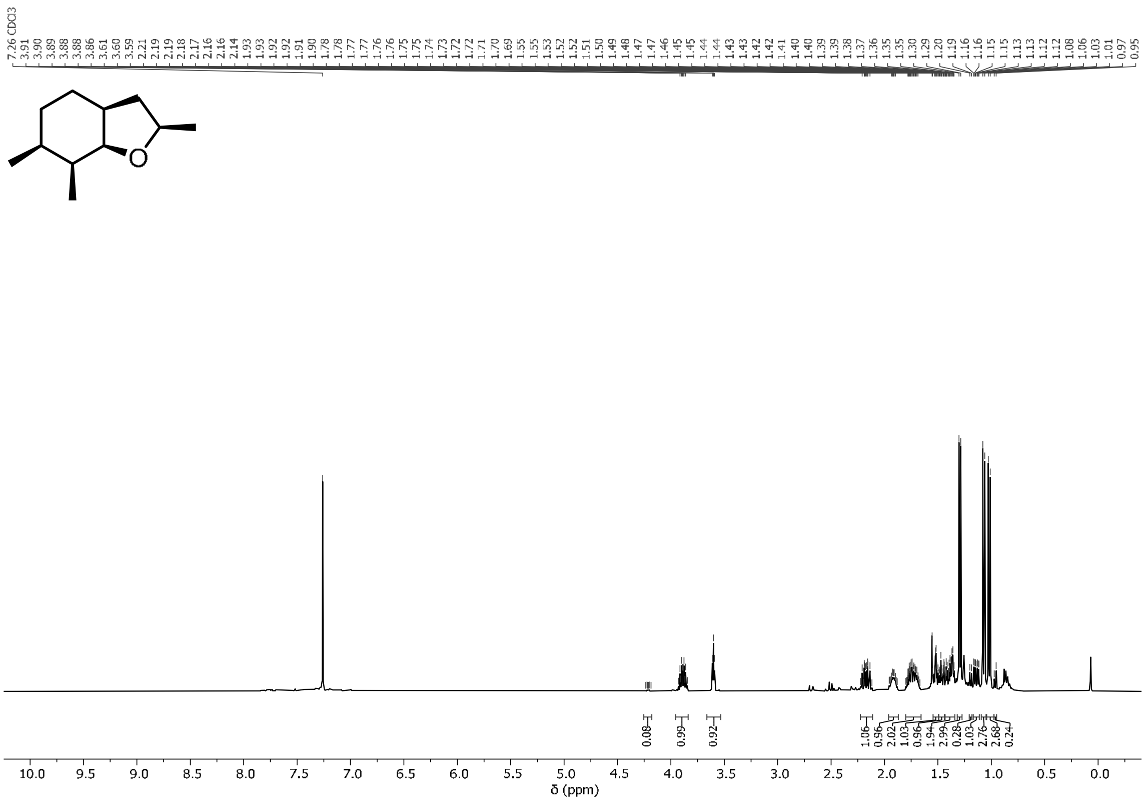
O
7.26 CDCl3
3.91
3.90
3.89
3.88
3.88
3.86
3.61
3.60
3.59
2.21
2.19
2.19
2.18
2.17
2.16
2.16
2.14
1.93
1.93
1.93
1.92
1.92
1.91
1.90
1.78
1.78
1.77
1.77
1.76
1.75
1.75
1.74
1.73
1.72
1.72
1.71
1.70
1.69
1.55
1.55
1.53
1.52
1.52
1.51
1.50
1.49
1.48
1.47
1.47
1.46
1.45
1.45
1.44
1.44
1.43
1.43
1.42
1.42
1.41
1.40
1.40
1.39
1.39
1.38
1.37
1.36
1.35
1.35
1.30
1.29
1.20
1.19
1.16
1.16
1.15
1.15
1.13
1.13
1.12
1.12
1.08
1.06
1.03
1.01
0.97
0.95
0.08
0.99
0.92
1.06
0.96
2.02
1.03
0.96
1.94
2.99
0.28
1.03
2.76
2.68
0.24
10.0 9.5 9.0 8.5 8.0 7.5 7.0 6.5 6.0 5.5 5.0 4.5 4.0 3.5 3.0 2.5 2.0 1.5 1.0 0.5 0.0
δ (ppm)

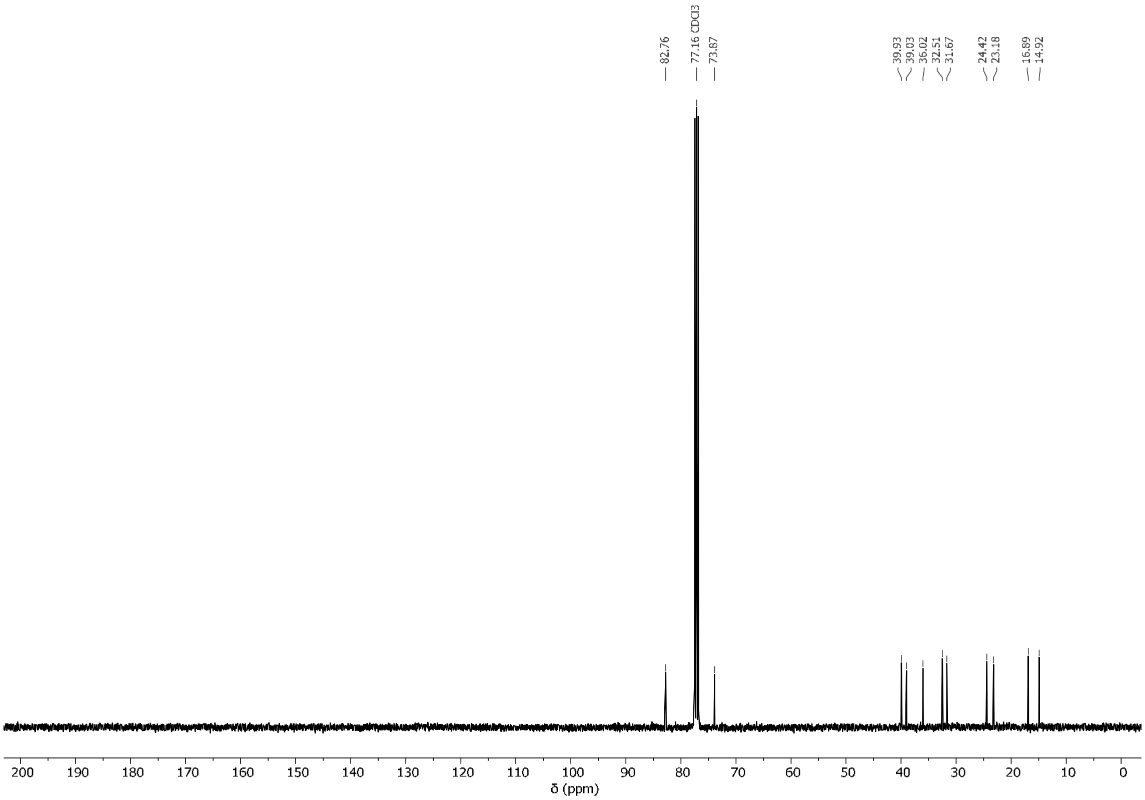
82.76
77.16 CDCl3
73.87
39.93
39.03
36.02
32.51
31.67
24.42
23.18
16.89
14.92
200 190 180 170 160 150 140 130 120 110 100 90 80 70 60 50 40 30 20 10 0
δ (ppm)

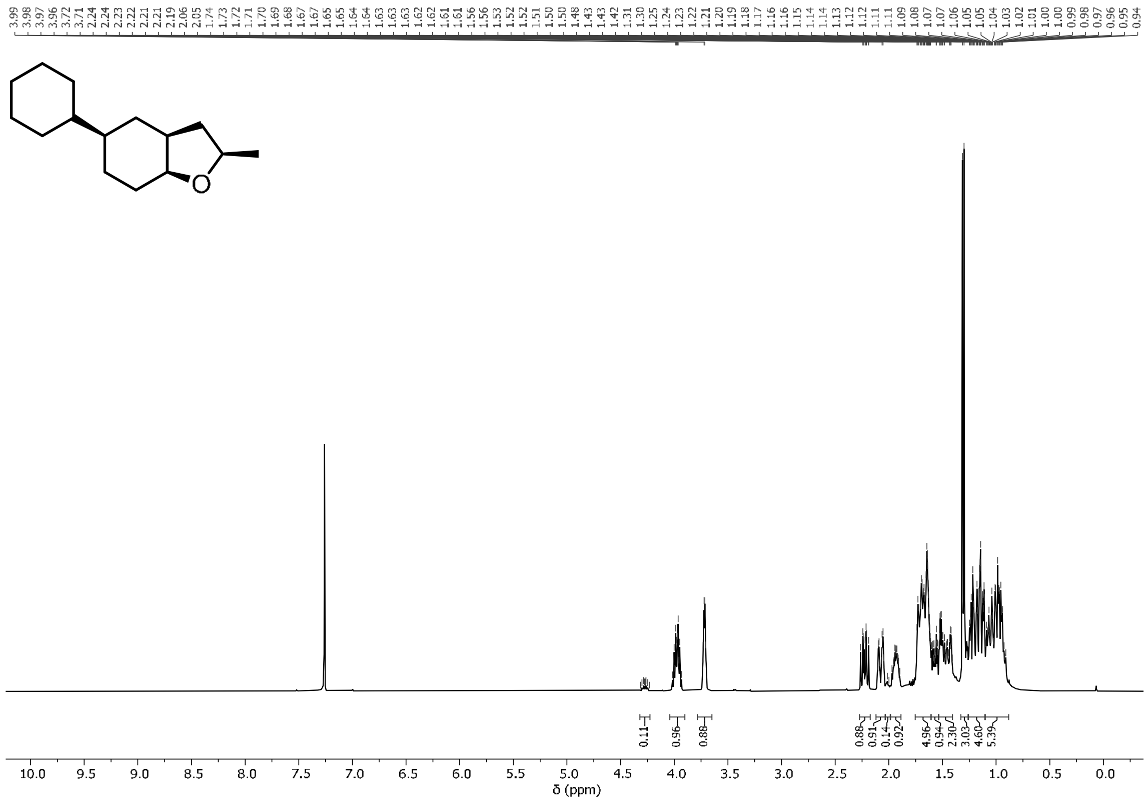
3.99 3.98 3.97 3.96 3.72 3.71 2.24 2.24 2.23 2.22 2.21 2.21 2.19 2.06 2.05 1.74 1.73 1.72 1.71 1.70 1.69 1.68 1.67 1.67 1.65 1.65 1.64 1.63 1.63 1.62 1.61 1.61 1.56 1.56 1.53 1.52 1.52 1.51 1.50 1.50 1.48 1.43 1.43 1.42 1.31 1.30 1.25 1.24 1.23 1.22 1.21 1.20 1.19 1.18 1.17 1.16 1.16 1.15 1.14 1.14 1.13 1.12 1.12 1.11 1.11 1.09 1.08 1.07 1.07 1.06 1.05 1.05 1.04 1.03 1.02 1.01 1.00 1.00 0.99 0.98 0.97 0.96 0.95 0.94
0.11 0.96 0.88 0.88 0.91 0.14 0.92 4.96 0.94 2.30 3.03 4.60 5.39
10.0 9.5 9.0 8.5 8.0 7.5 7.0 6.5 6.0 5.5 5.0 4.5 4.0 3.5 3.0 2.5 2.0 1.5 1.0 0.5 0.0
δ (ppm)

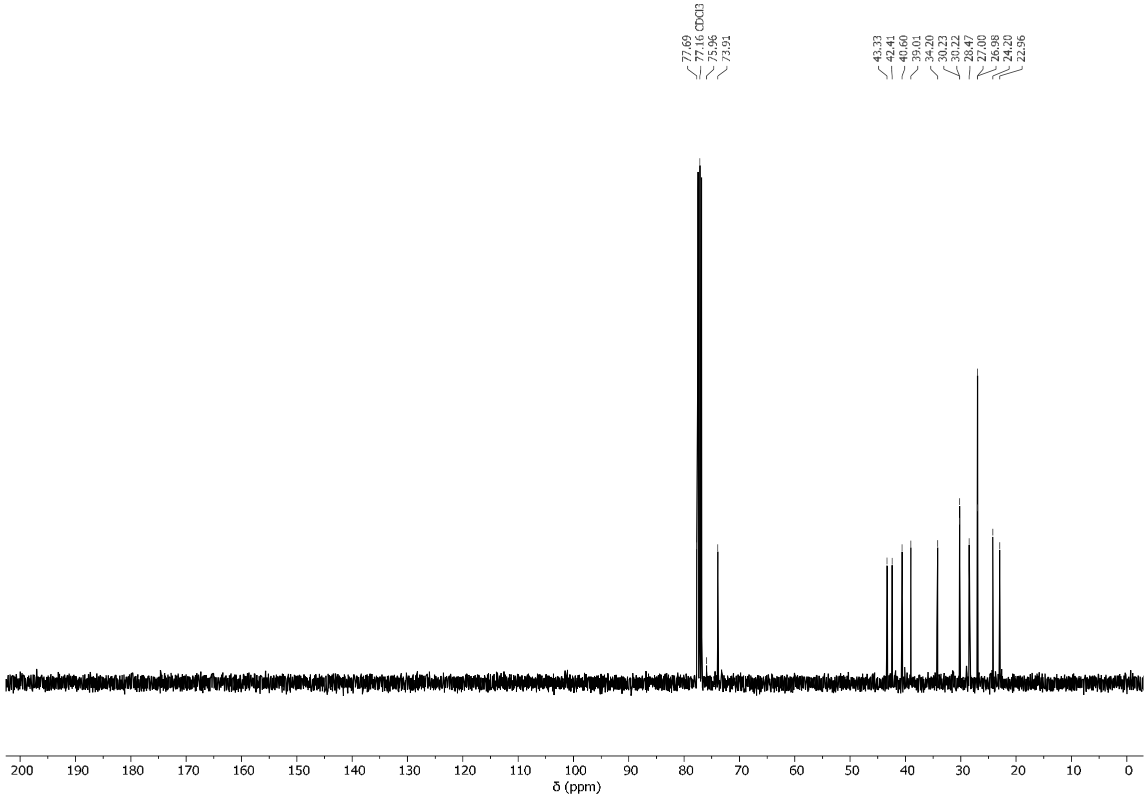
77.69 77.16 CDCl3 75.96 73.91
43.33 42.41 40.60 39.01 34.20 30.23 30.22 28.47 27.00 26.98 24.20 22.96
200 190 180 170 160 150 140 130 120 110 100 90 80 70 60 50 40 30 20 10 0
δ (ppm)

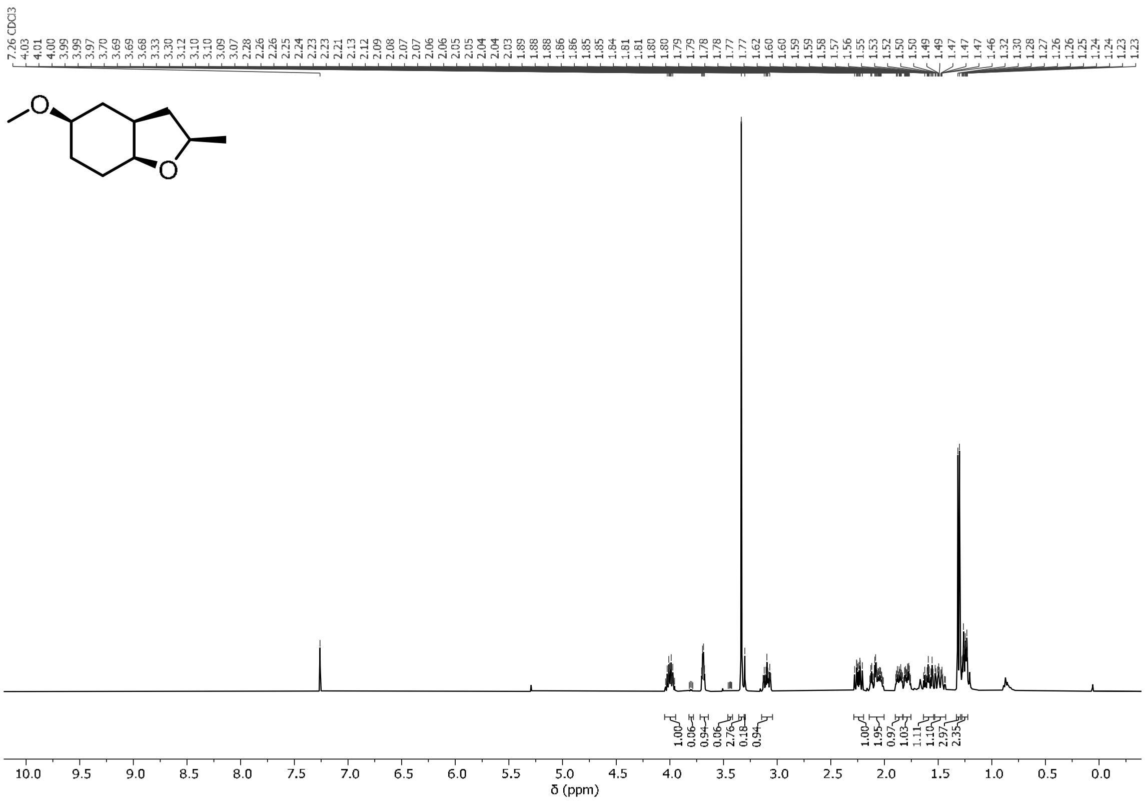
7.26 CDCl3
4.03 4.01 4.00 3.99 3.99 3.97 3.70 3.69 3.69 3.68 3.33 3.30 3.12 3.10 3.10 3.09 3.07 2.28 2.26 2.26 2.25 2.24 2.23 2.23 2.21 2.13 2.12 2.09 2.08 2.07 2.07 2.06 2.06 2.05 2.05 2.04 2.04 2.03 1.89 1.88 1.88 1.86 1.86 1.85 1.85 1.84 1.81 1.81 1.80 1.80 1.79 1.79 1.78 1.78 1.77 1.77 1.62 1.60 1.60 1.59 1.59 1.58 1.57 1.56 1.55 1.53 1.52 1.50 1.50 1.49 1.49 1.47 1.47 1.46 1.32 1.30 1.28 1.27 1.26 1.26 1.25 1.24 1.24 1.23 1.23
O
O
1.00 0.06 0.94 0.06 2.76 0.18 0.94 1.00 1.95 0.97 1.03 1.11 1.10 2.97 2.35
10.0 9.5 9.0 8.5 8.0 7.5 7.0 6.5 6.0 5.5 5.0 4.5 4.0 3.5 3.0 2.5 2.0 1.5 1.0 0.5 0.0
δ (ppm)

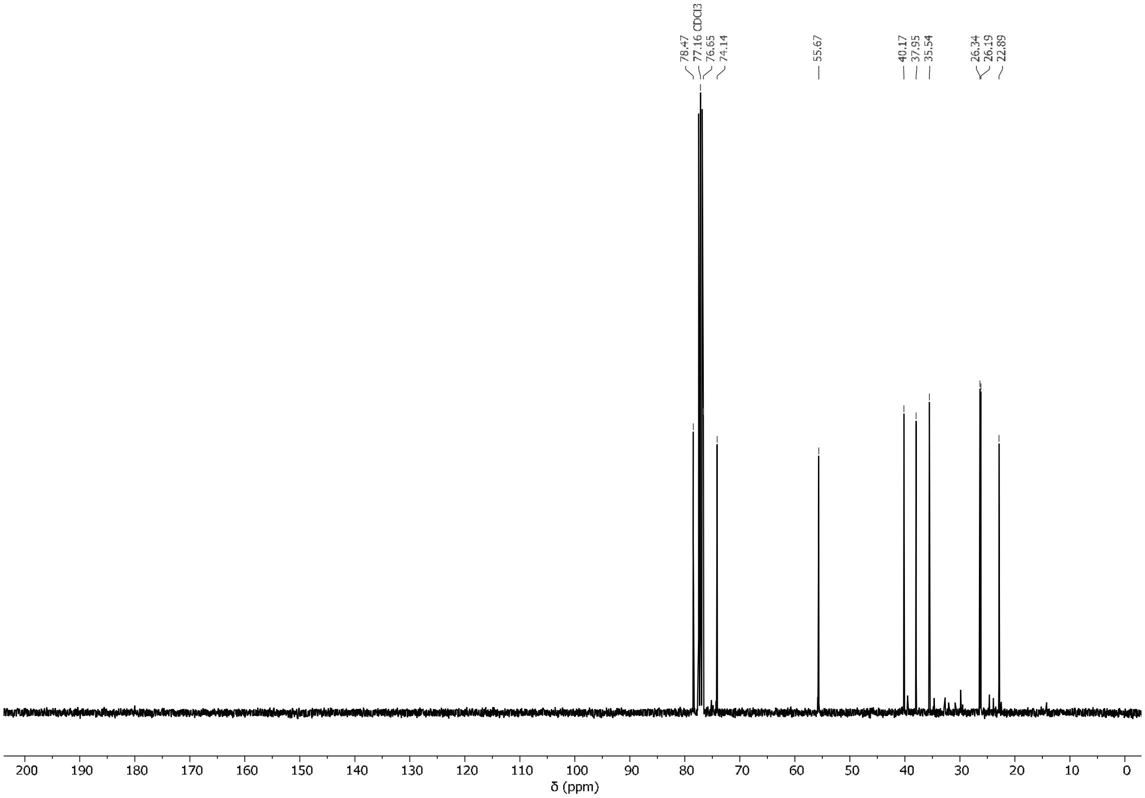
78.47 77.16 CDCl3 76.65 74.14
55.67
40.17 37.95 35.54
26.34 26.19 22.89
200 190 180 170 160 150 140 130 120 110 100 90 80 70 60 50 40 30 20 10 0
δ (ppm)

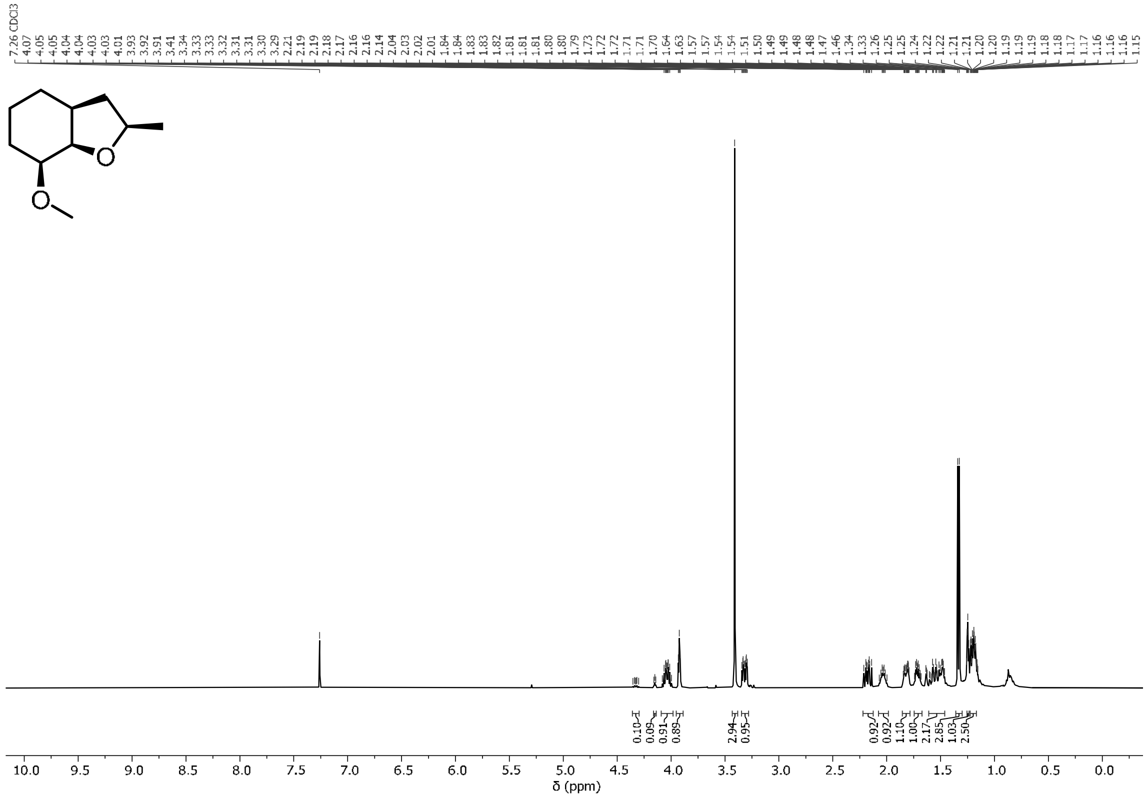
7.26 CDCl3
4.07 4.05 4.05 4.04 4.04 4.03 4.03 4.01 3.93 3.92 3.91 3.41 3.34 3.33 3.33 3.32 3.31 3.31 3.30 3.29 2.21 2.19 2.19 2.18 2.17 2.16 2.16 2.14 2.04 2.03 2.02 2.01 1.84 1.84 1.83 1.83 1.82 1.81 1.81 1.81 1.80 1.80 1.79 1.73 1.72 1.72 1.71 1.71 1.70 1.64 1.63 1.57 1.57 1.54 1.54 1.51 1.50 1.49 1.49 1.48 1.48 1.47 1.46 1.34 1.33 1.26 1.25 1.25 1.24 1.22 1.22 1.21 1.21 1.21 1.20 1.20 1.19 1.19 1.19 1.18 1.18 1.17 1.17 1.16 1.16 1.16 1.15
0.10 0.09 0.91 0.89 2.94 0.95 0.92 0.92 1.10 1.00 2.17 2.85 1.03 2.50
10.0 9.5 9.0 8.5 8.0 7.5 7.0 6.5 6.0 5.5 5.0 4.5 4.0 3.5 3.0 2.5 2.0 1.5 1.0 0.5 0.0
δ (ppm)

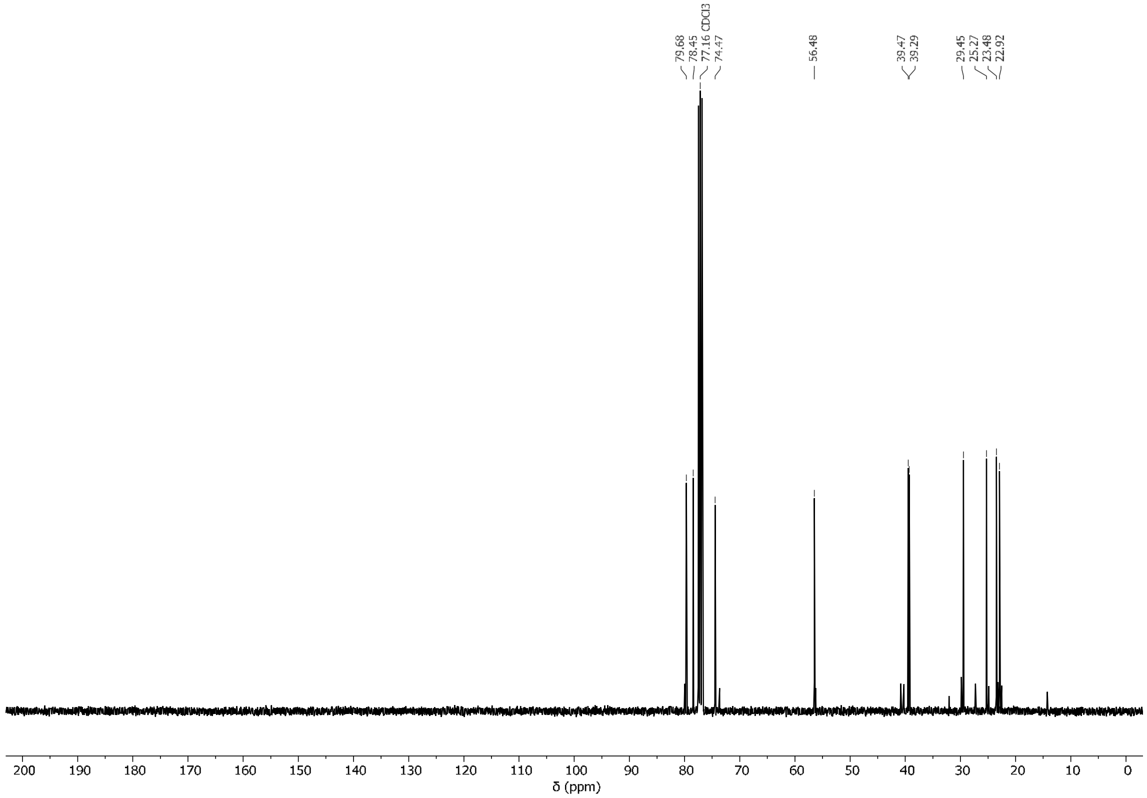
79.68 78.45 77.16 CDCl3 74.47
56.48
39.47 39.29
29.45 25.27 23.48 22.92
200 190 180 170 160 150 140 130 120 110 100 90 80 70 60 50 40 30 20 10 0
δ (ppm)

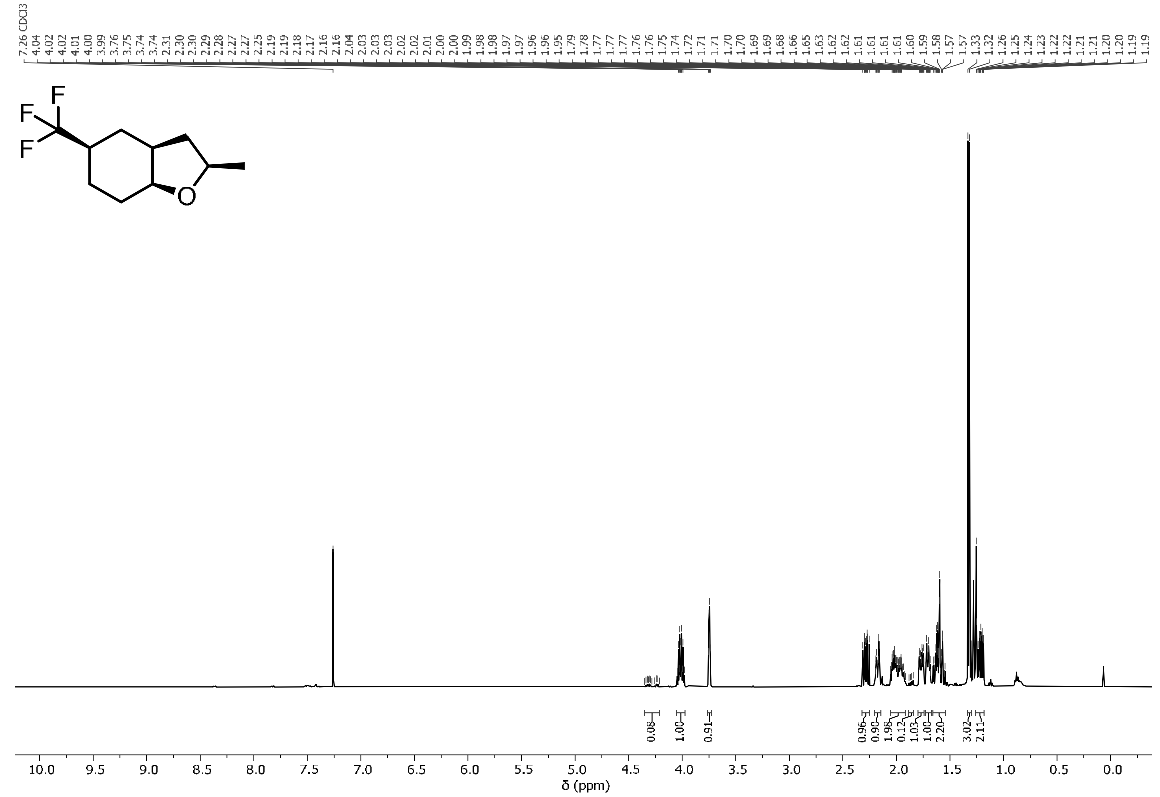

7.26 CDCl3
4.04
4.02
4.02
4.01
4.00
3.99
3.76
3.75
3.74
3.74
2.31
2.30
2.30
2.29
2.28
2.27
2.27
2.25
2.19
2.19
2.18
2.17
2.16
2.16
2.04
2.03
2.03
2.03
2.02
2.02
2.01
2.00
1.99
1.98
1.98
1.97
1.97
1.96
1.96
1.95
1.79
1.78
1.77
1.77
1.77
1.76
1.75
1.74
1.72
1.71
1.71
1.70
1.70
1.69
1.69
1.68
1.66
1.65
1.63
1.62
1.62
1.61
1.61
1.61
1.61
1.60
1.59
1.58
1.57
1.57
1.33
1.32
1.26
1.25
1.24
1.23
1.22
1.22
1.21
1.21
1.20
1.19
1.19
F
F
F
O
0.08
1.00
0.91
0.96
0.90
1.98
0.12
1.03
1.00
2.20
3.02
2.11
10.0 9.5 9.0 8.5 8.0 7.5 7.0 6.5 6.0 5.5 5.0 4.5 4.0 3.5 3.0 2.5 2.0 1.5 1.0 0.5 0.0
δ (ppm)

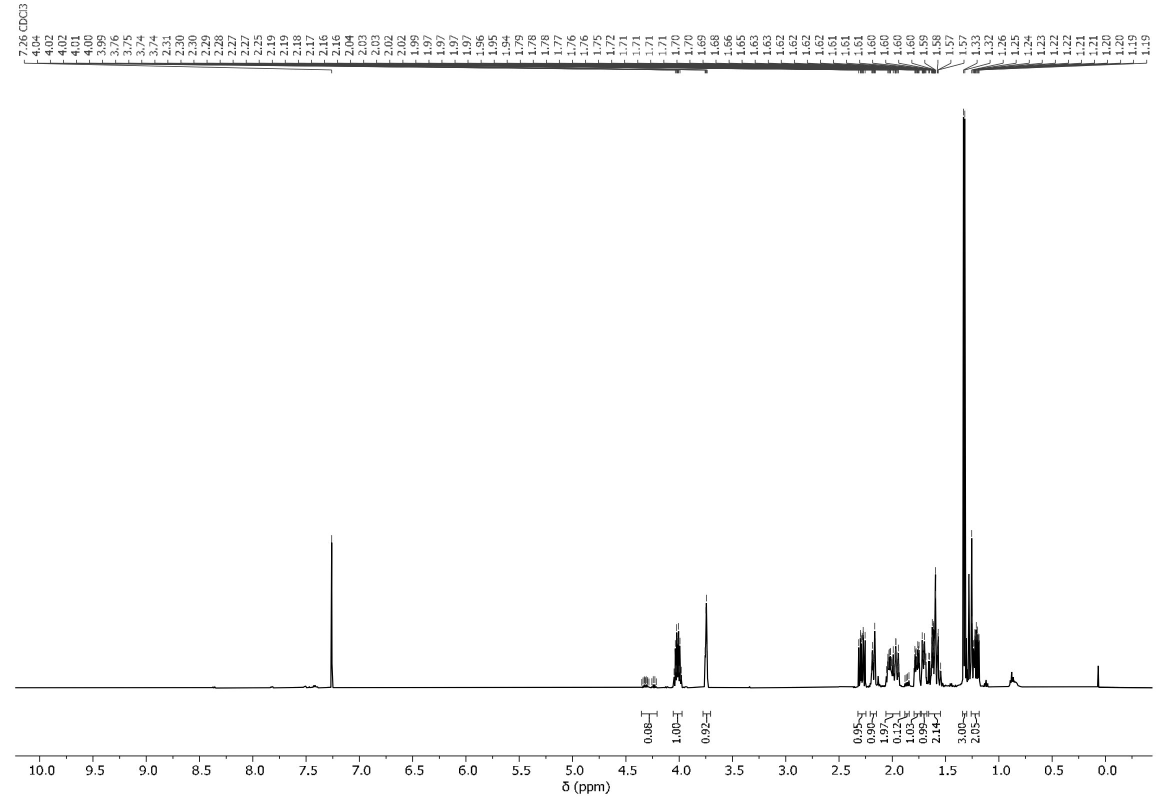

7.26 CDCl3
4.04
4.02
4.02
4.01
4.00
3.99
3.76
3.75
3.74
3.74
2.31
2.30
2.30
2.29
2.28
2.27
2.27
2.25
2.19
2.19
2.18
2.17
2.16
2.16
2.04
2.03
2.03
2.03
2.02
2.02
1.99
1.97
1.97
1.97
1.96
1.95
1.94
1.79
1.78
1.78
1.77
1.76
1.76
1.75
1.72
1.71
1.71
1.71
1.70
1.70
1.69
1.68
1.66
1.65
1.63
1.63
1.62
1.62
1.62
1.61
1.61
1.61
1.60
1.60
1.60
1.59
1.58
1.57
1.57
1.33
1.32
1.26
1.25
1.24
1.23
1.22
1.22
1.21
1.21
1.20
1.20
1.19
1.19
0.08
1.00
0.92
0.95
0.90
1.97
0.12
1.03
0.99
2.14
3.00
2.05
10.0 9.5 9.0 8.5 8.0 7.5 7.0 6.5 6.0 5.5 5.0 4.5 4.0 3.5 3.0 2.5 2.0 1.5 1.0 0.5 0.0
δ (ppm)

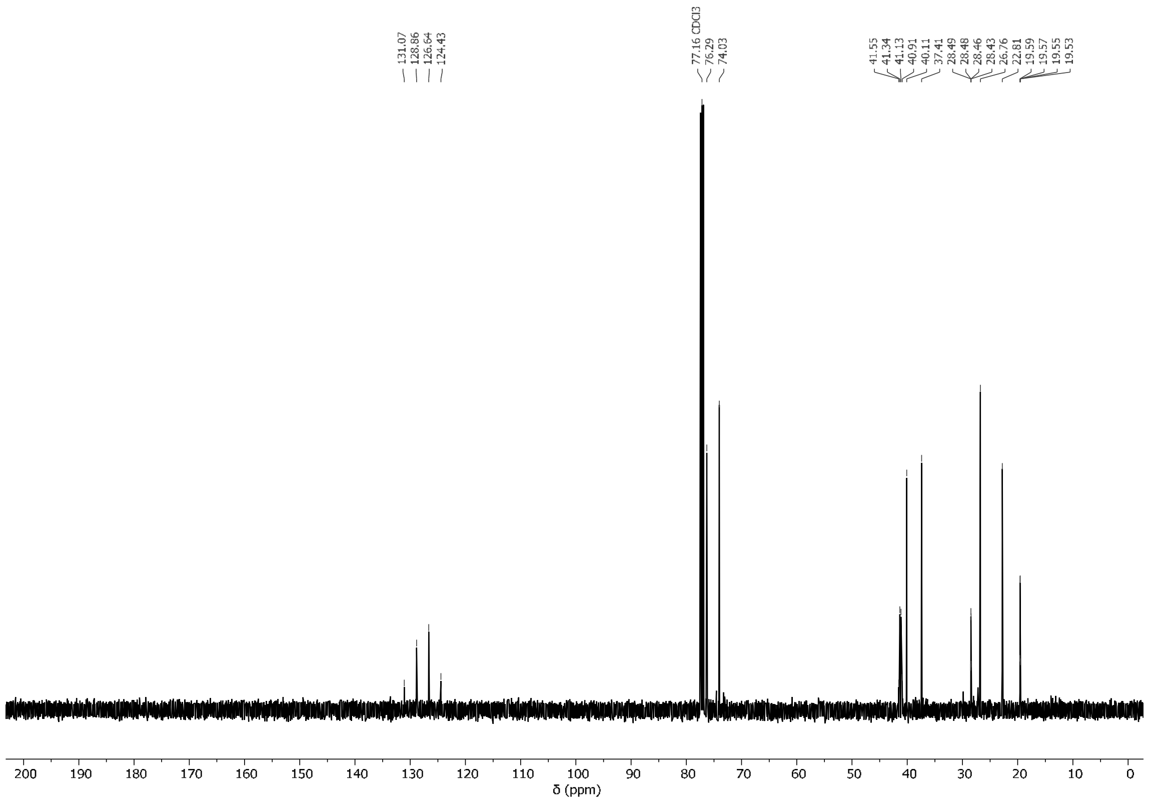
131.07
128.86
126.64
124.43
77.16 CDCl3
76.29
74.03
41.55
41.34
41.13
40.91
40.11
37.41
28.49
28.48
28.46
28.43
26.76
22.81
19.59
19.57
19.55
19.53
δ (ppm)

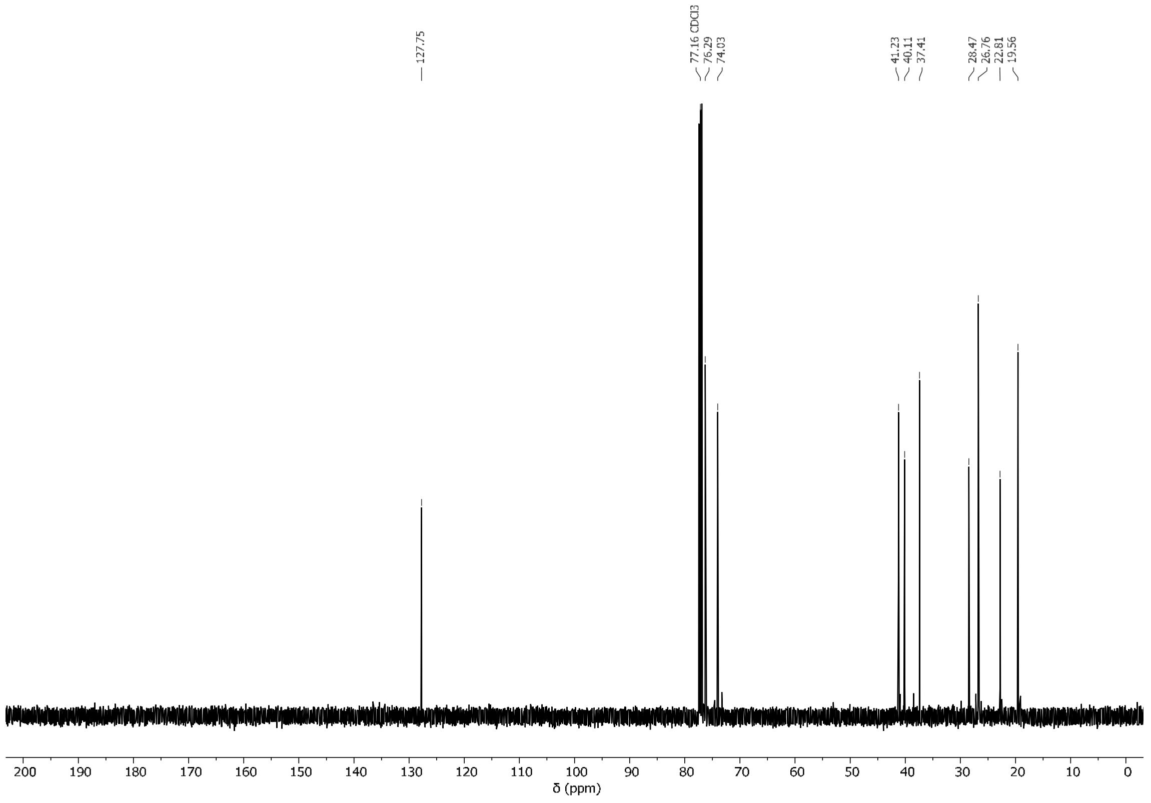
127.75
77.16 CDCl3
76.29
74.03
41.23
40.11
37.41
28.47
26.76
22.81
19.56
δ (ppm)

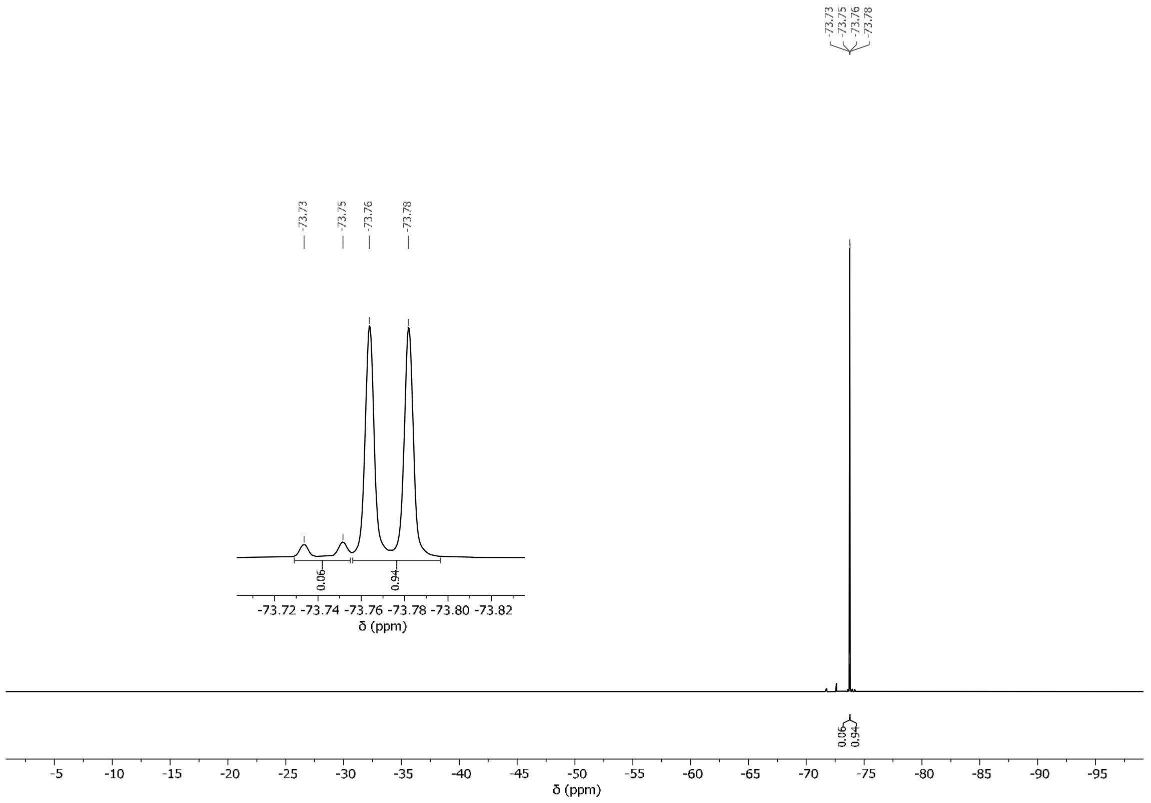

-73.73
-73.75
-73.76
-73.78
0.06
0.94
-73.72 -73.74 -73.76 -73.78 -73.80 -73.82
δ (ppm)
-5 -10 -15 -20 -25 -30 -35 -40 -45 -50 -55 -60 -65 -70 -75 -80 -85 -90 -95
δ (ppm)

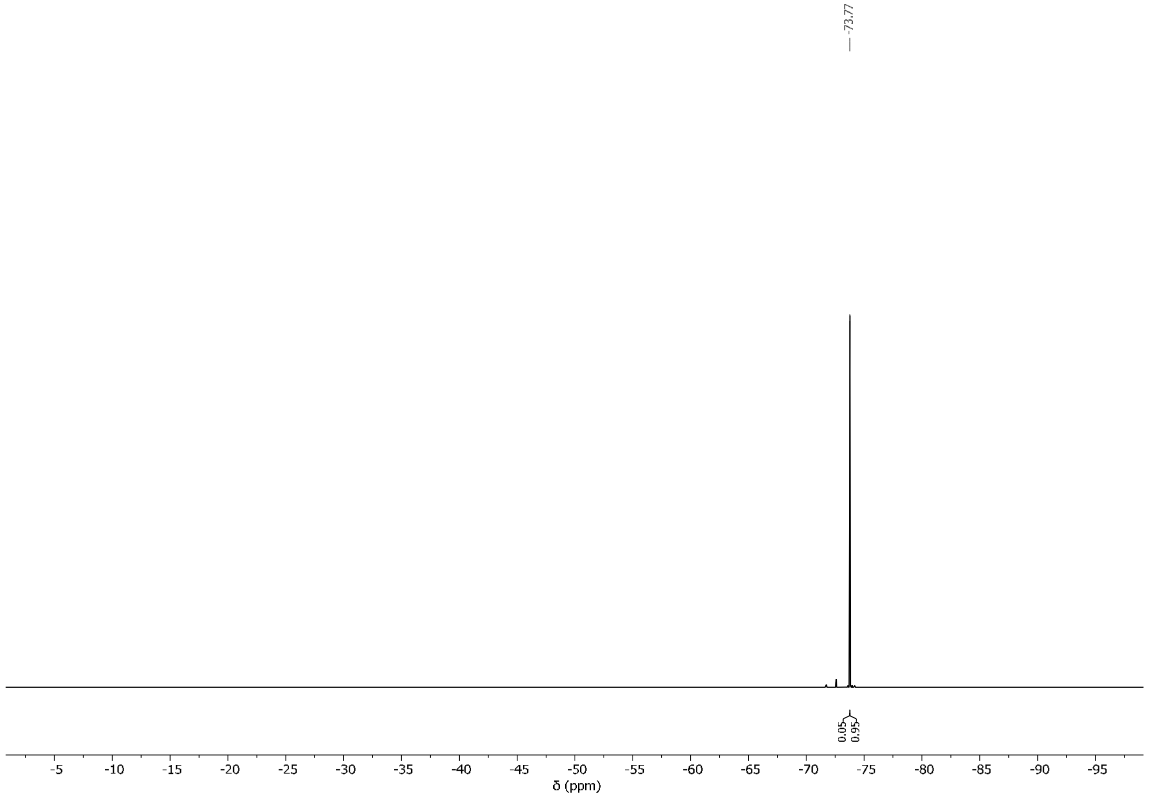

-73.77
0.05
0.95
-5 -10 -15 -20 -25 -30 -35 -40 -45 -50 -55 -60 -65 -70 -75 -80 -85 -90 -95
δ (ppm)

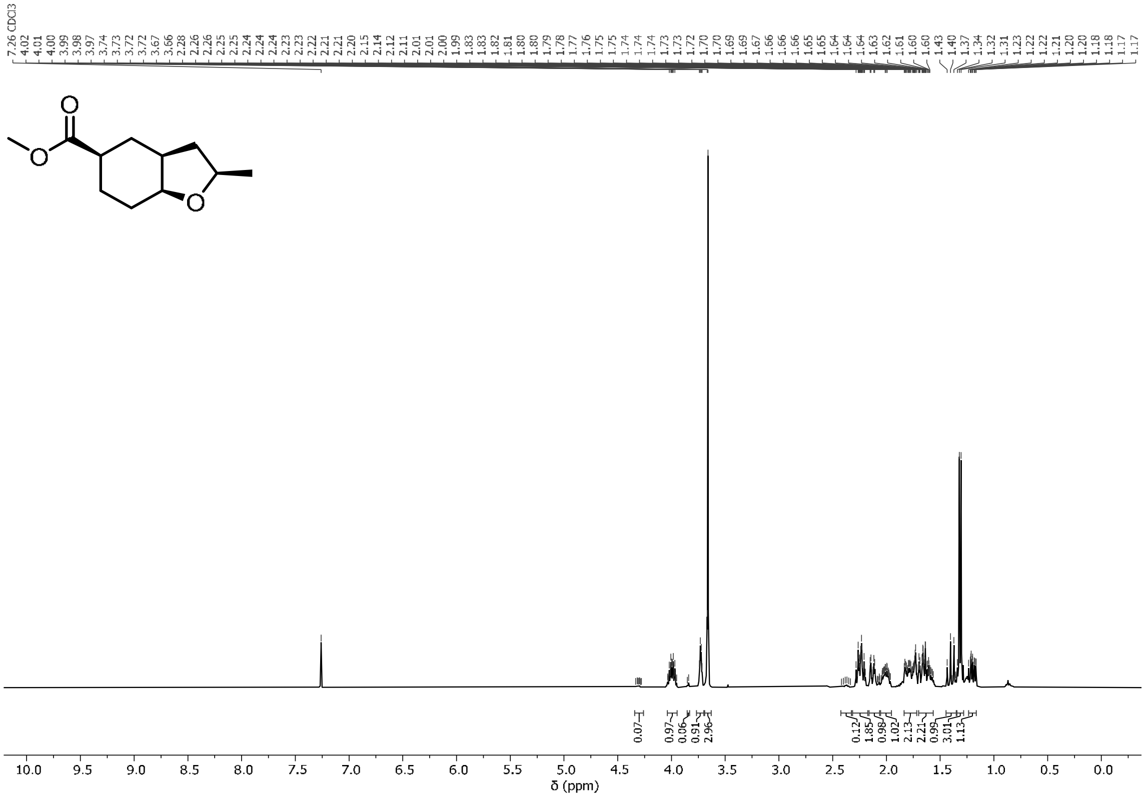
7.26 CDCl3
4.02
4.01
4.00
3.99
3.98
3.97
3.74
3.73
3.72
3.72
3.67
3.66
2.28
2.26
2.26
2.25
2.25
2.24
2.24
2.24
2.23
2.23
2.22
2.21
2.21
2.20
2.15
2.14
2.12
2.11
2.01
2.01
2.00
1.99
1.83
1.83
1.82
1.81
1.80
1.80
1.79
1.78
1.77
1.76
1.75
1.75
1.74
1.74
1.73
1.73
1.72
1.70
1.70
1.69
1.69
1.67
1.66
1.66
1.66
1.65
1.65
1.64
1.64
1.64
1.63
1.62
1.61
1.60
1.60
1.43
1.40
1.37
1.34
1.32
1.31
1.23
1.22
1.22
1.21
1.20
1.20
1.18
1.17
1.17
O
O
O
0.07
0.97
0.06
0.91
2.96
0.12
1.85
0.98
1.02
2.13
2.21
0.99
3.01
1.13
10.0 9.5 9.0 8.5 8.0 7.5 7.0 6.5 6.0 5.5 5.0 4.5 4.0 3.5 3.0 2.5 2.0 1.5 1.0 0.5 0.0
δ (ppm)

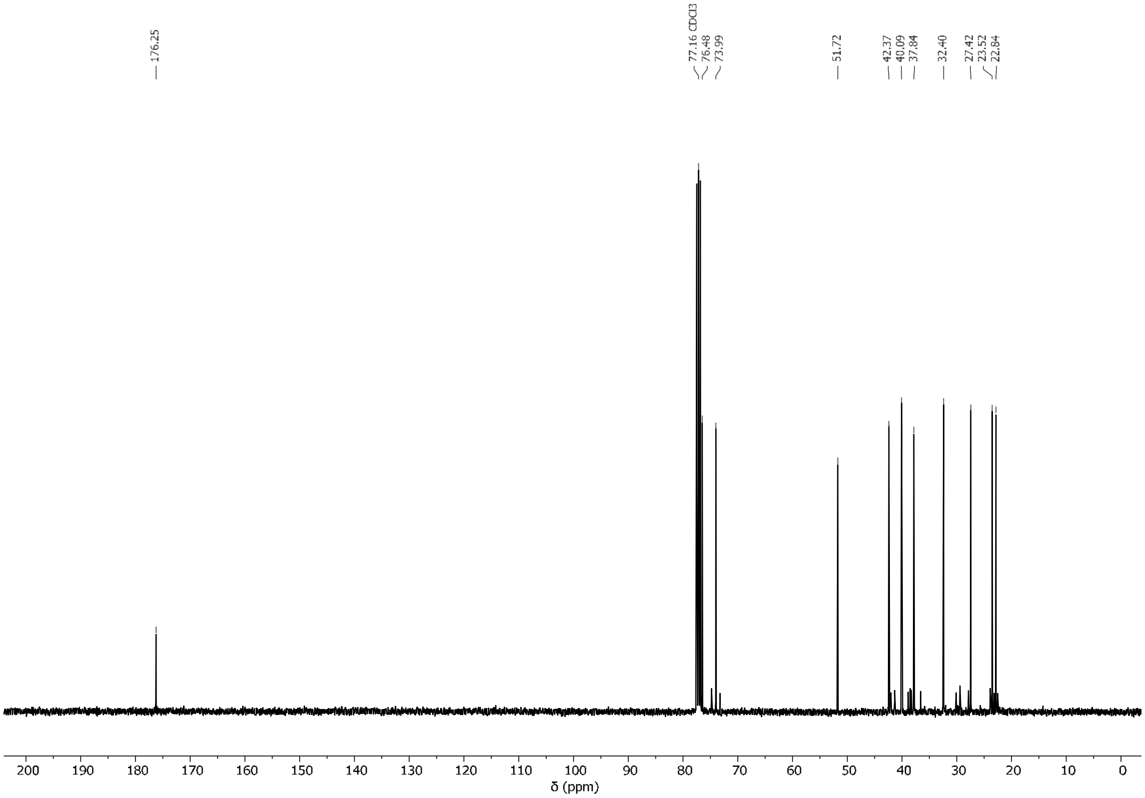
176.25
77.16 CDCl3
76.48
73.99
51.72
42.37
40.09
37.84
32.40
27.42
23.52
22.84
200 190 180 170 160 150 140 130 120 110 100 90 80 70 60 50 40 30 20 10 0
δ (ppm)

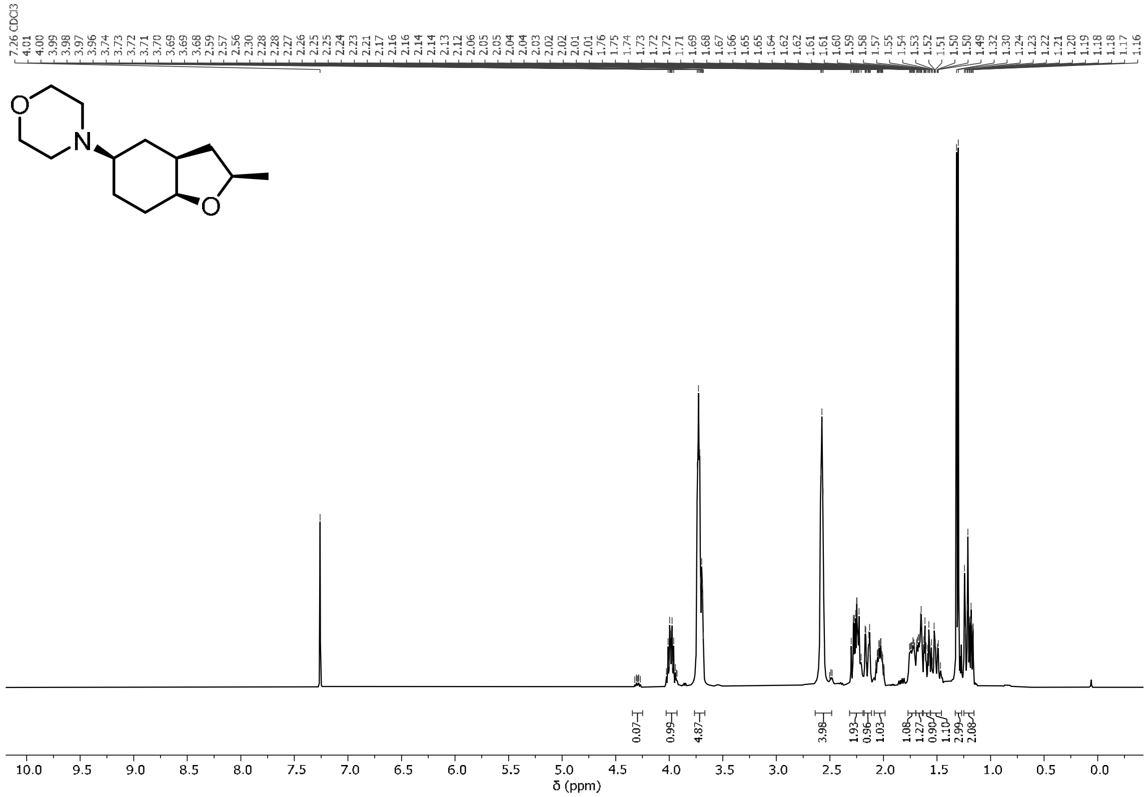
7.26 CDCl3
4.01
4.00
3.99
3.98
3.97
3.96
3.74
3.73
3.72
3.71
3.70
3.69
3.69
3.68
2.59
2.57
2.56
2.30
2.28
2.28
2.27
2.26
2.25
2.25
2.24
2.23
2.21
2.17
2.16
2.16
2.14
2.14
2.13
2.12
2.06
2.05
2.05
2.04
2.04
2.03
2.02
2.02
2.01
2.01
1.76
1.75
1.74
1.73
1.72
1.72
1.71
1.69
1.68
1.67
1.66
1.65
1.65
1.64
1.62
1.62
1.61
1.61
1.60
1.59
1.58
1.57
1.55
1.54
1.53
1.52
1.51
1.50
1.50
1.49
1.32
1.30
1.24
1.23
1.22
1.21
1.20
1.19
1.18
1.18
1.17
1.16
0.07
0.99
4.87
3.98
1.93
0.96
1.03
1.08
1.27
0.90
1.10
2.99
2.08
δ (ppm)
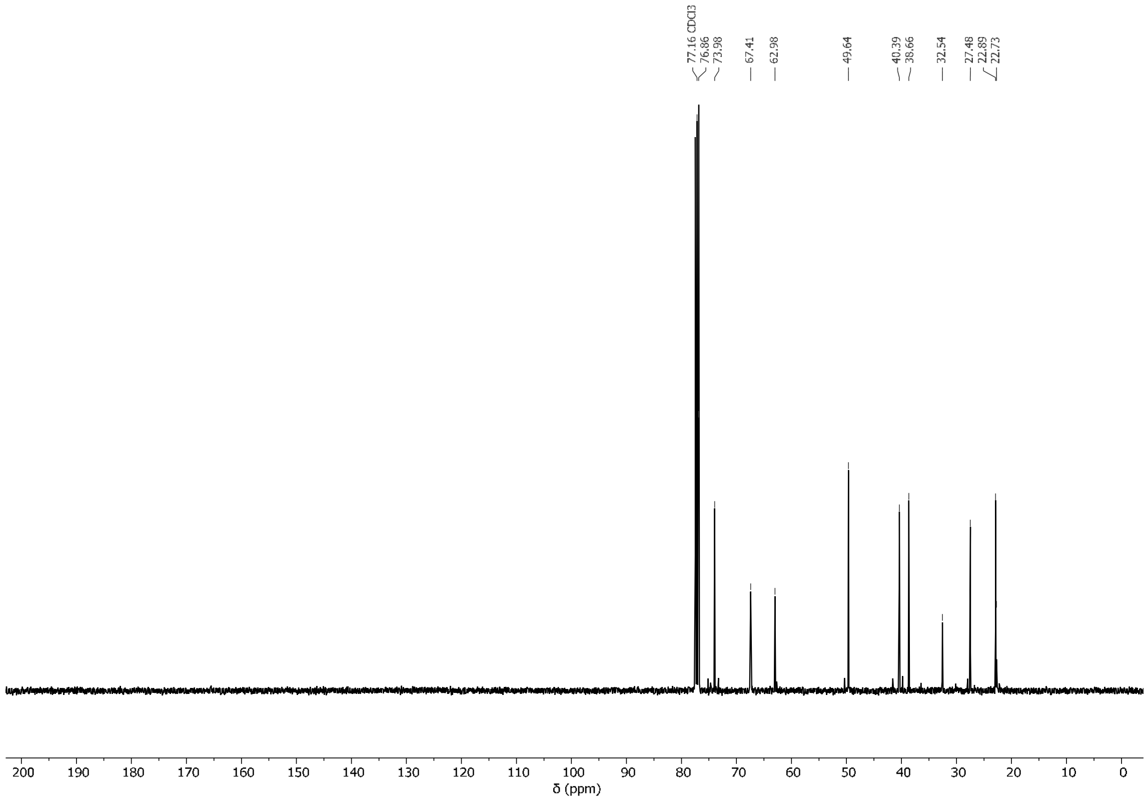
77.16 CDCl3
76.86
73.98
67.41
62.98
49.64
40.39
38.66
32.54
27.48
22.89
22.73
δ (ppm)

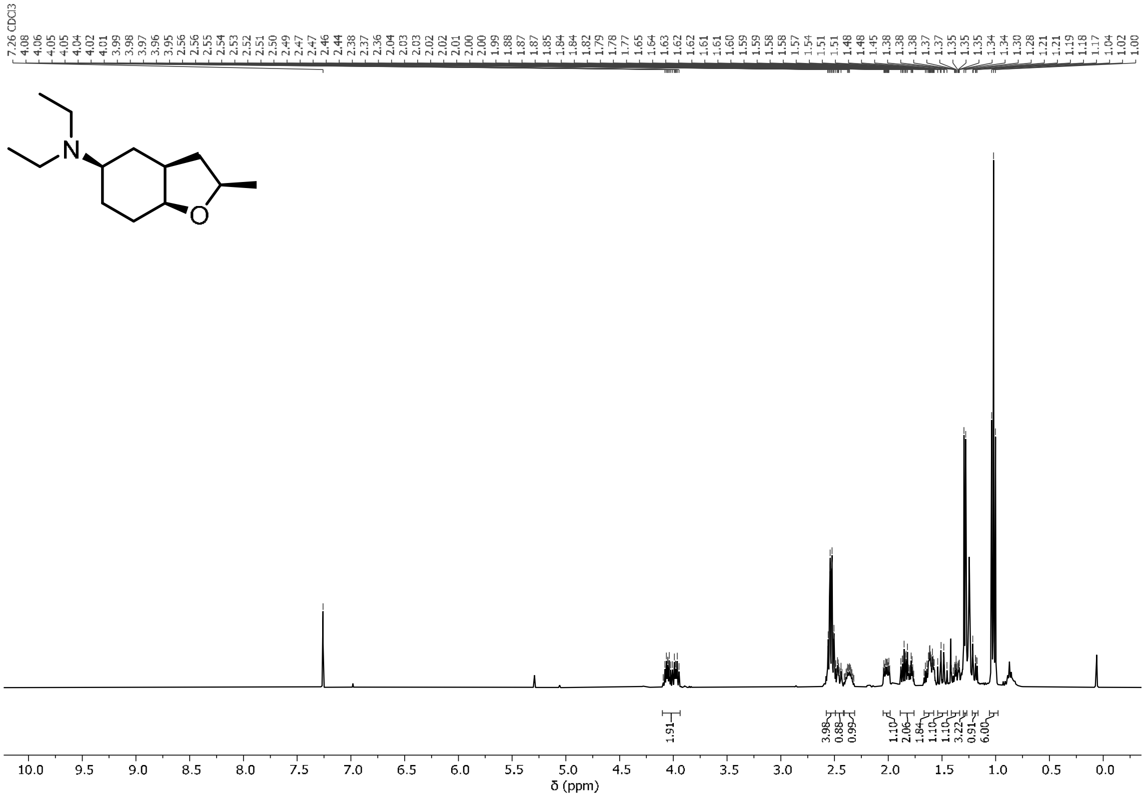
7.26 CDCl3
4.08
4.06
4.05
4.05
4.04
4.02
4.01
3.99
3.98
3.97
3.96
3.95
2.56
2.56
2.55
2.54
2.53
2.52
2.51
2.50
2.49
2.47
2.47
2.46
2.44
2.38
2.37
2.36
2.04
2.03
2.03
2.02
2.02
2.01
2.00
2.00
1.99
1.88
1.87
1.87
1.85
1.84
1.84
1.82
1.79
1.78
1.77
1.65
1.64
1.63
1.62
1.62
1.61
1.61
1.60
1.59
1.59
1.58
1.58
1.57
1.54
1.51
1.51
1.48
1.48
1.45
1.38
1.38
1.38
1.37
1.37
1.35
1.35
1.35
1.34
1.34
1.30
1.28
1.21
1.21
1.19
1.18
1.17
1.04
1.02
1.00
N
O
1.91
3.98
0.88
0.99
1.10
2.06
1.84
1.10
1.10
3.22
0.91
6.00
10.0 9.5 9.0 8.5 8.0 7.5 7.0 6.5 6.0 5.5 5.0 4.5 4.0 3.5 3.0 2.5 2.0 1.5 1.0 0.5 0.0
δ (ppm)

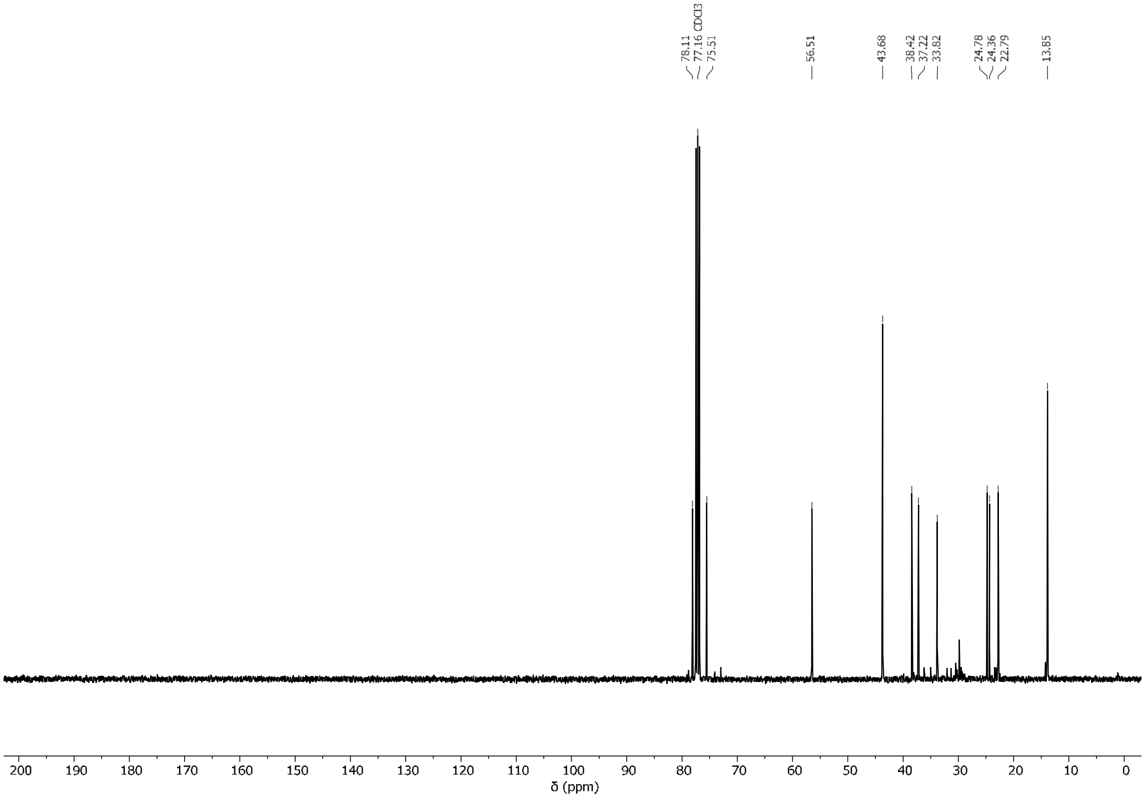
78.11
77.16 CDCl3
75.51
56.51
43.68
38.42
37.22
33.82
24.78
24.36
22.79
13.85
200 190 180 170 160 150 140 130 120 110 100 90 80 70 60 50 40 30 20 10 0
δ (ppm)

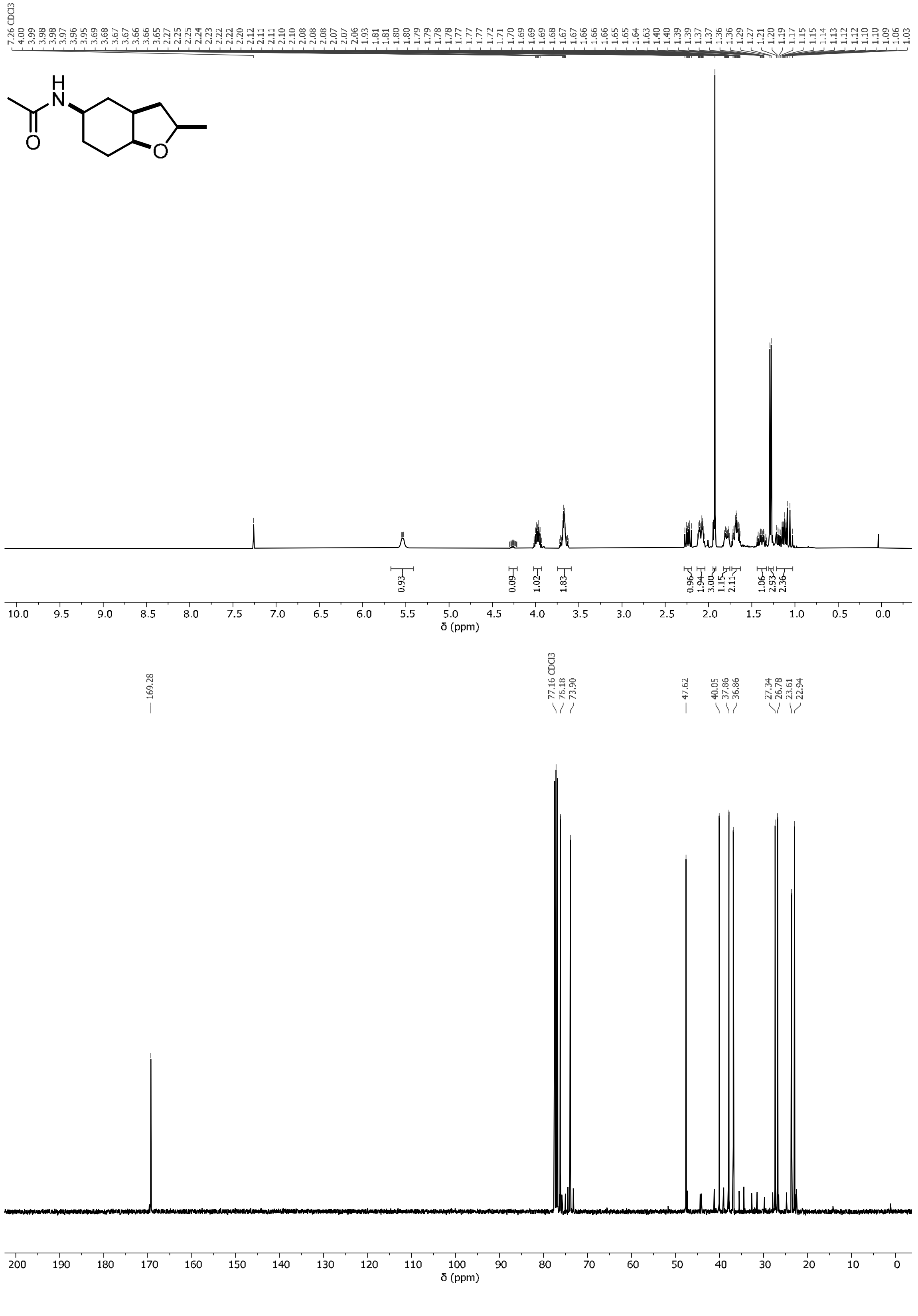

7.26 CDCl3
δ (ppm)
169.28
77.16 CDCl3
76.18
73.90
47.62
40.05
37.86
36.86
27.34
26.78
23.61
22.94
δ (ppm)

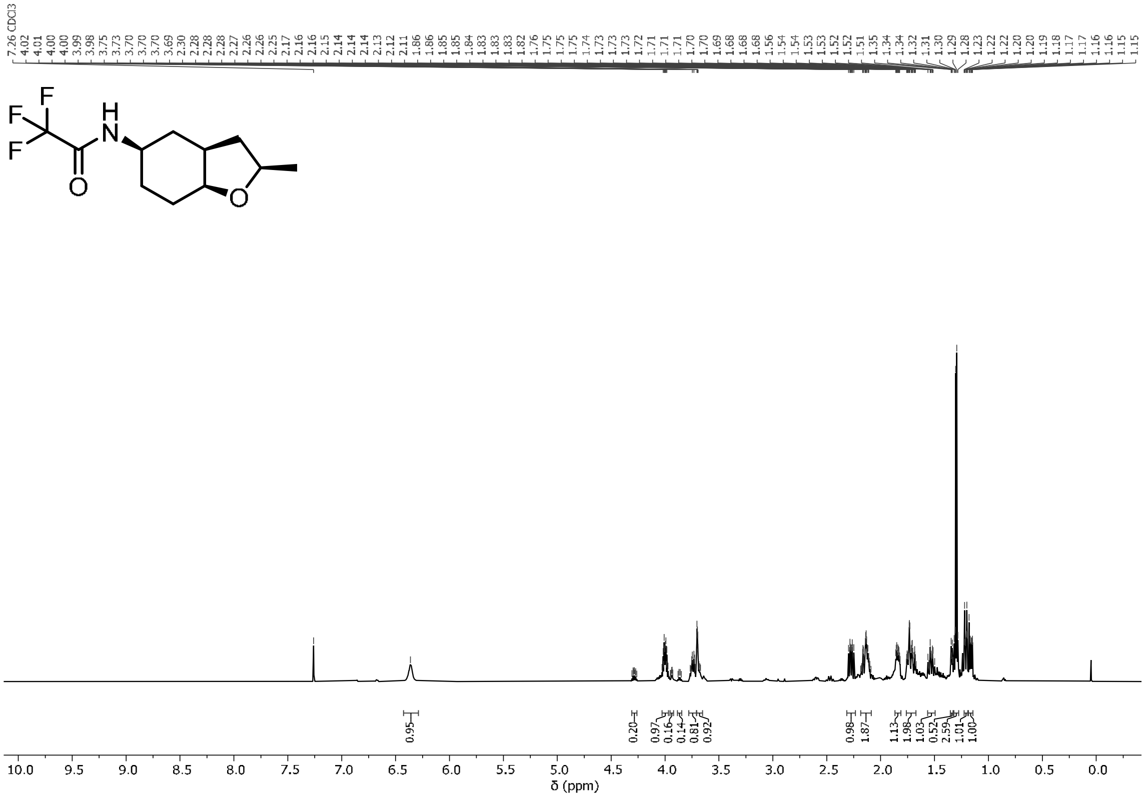
7.26 CDCl3
4.02
4.01
4.00
4.00
3.99
3.98
3.75
3.73
3.70
3.70
3.70
3.69
2.30
2.28
2.28
2.28
2.27
2.26
2.26
2.25
2.17
2.16
2.15
2.14
2.14
2.14
2.13
2.12
2.11
1.86
1.86
1.85
1.85
1.84
1.83
1.83
1.83
1.82
1.76
1.75
1.75
1.75
1.74
1.73
1.73
1.73
1.72
1.71
1.71
1.71
1.70
1.70
1.69
1.68
1.68
1.68
1.56
1.54
1.54
1.53
1.53
1.52
1.52
1.51
1.35
1.34
1.32
1.31
1.30
1.29
1.28
1.23
1.22
1.22
1.20
1.19
1.18
1.17
1.17
1.16
1.16
1.15
1.15
F
F
F
H
N
O
O
0.95
0.20
0.97
0.16
0.14
0.81
0.92
0.98
1.87
1.13
1.98
1.03
0.52
2.59
1.01
1.00
10.0
9.5
9.0
8.5
8.0
7.5
7.0
6.5
6.0
5.5
5.0
4.5
4.0
3.5
3.0
2.5
2.0
1.5
1.0
0.5
0.0
δ (ppm)

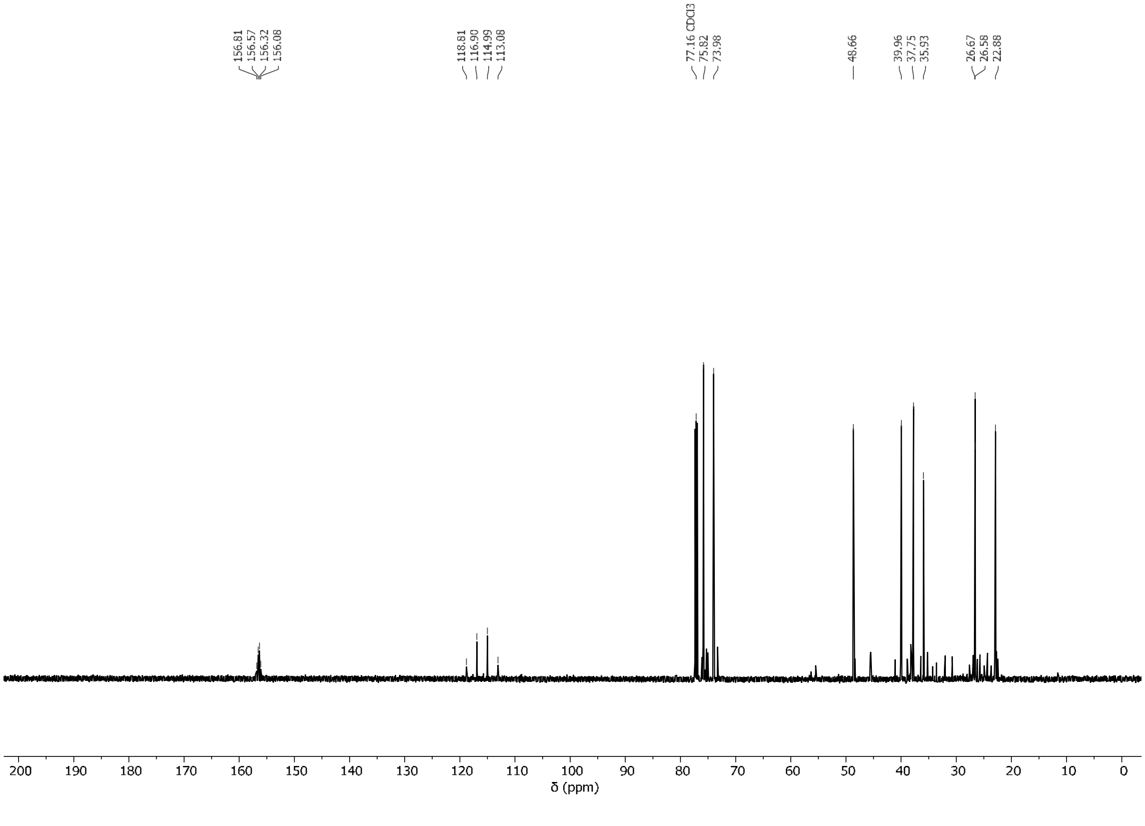
156.81
156.57
156.32
156.08
118.81
116.90
114.99
113.08
77.16 CDCl3
75.82
73.98
48.66
39.96
37.75
35.93
26.67
26.58
22.88
200
190
180
170
160
150
140
130
120
110
100
90
80
70
60
50
40
30
20
10
0
δ (ppm)

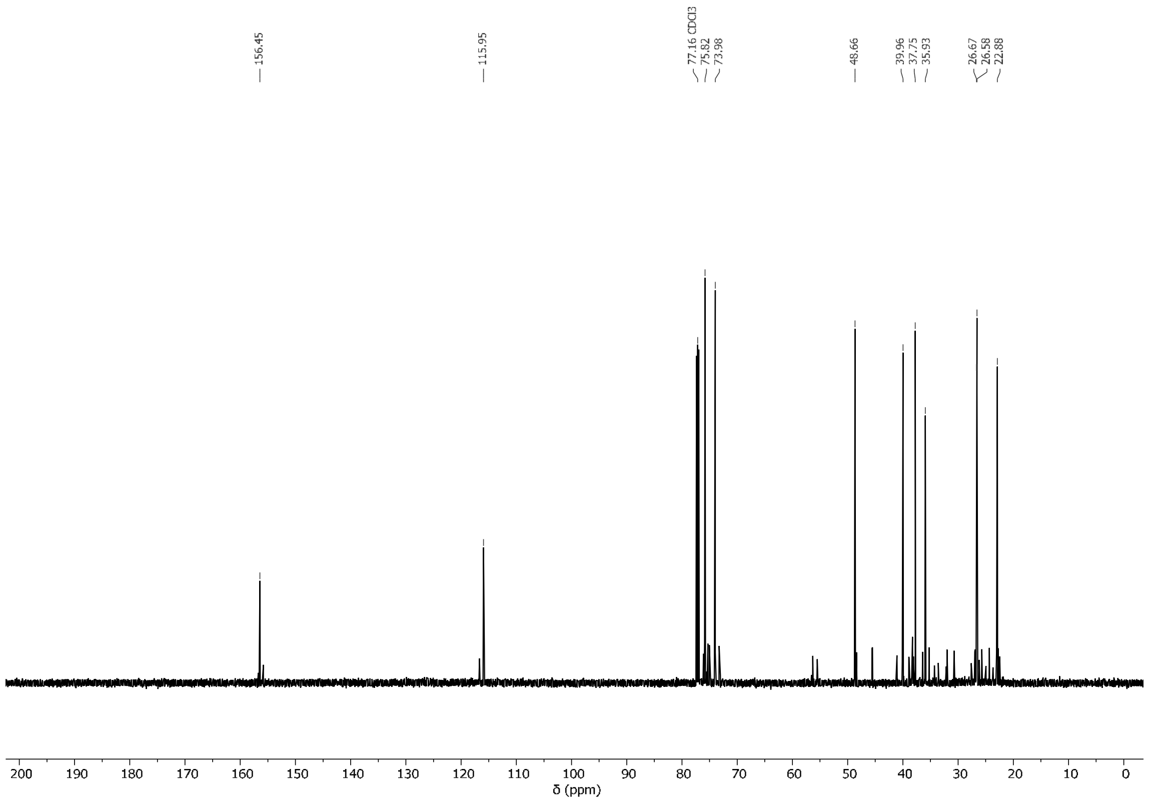
156.45
115.95
77.16 CDCl3
75.82
73.98
48.66
39.96
37.75
35.93
26.67
26.58
22.88
200 190 180 170 160 150 140 130 120 110 100 90 80 70 60 50 40 30 20 10 0
δ (ppm)

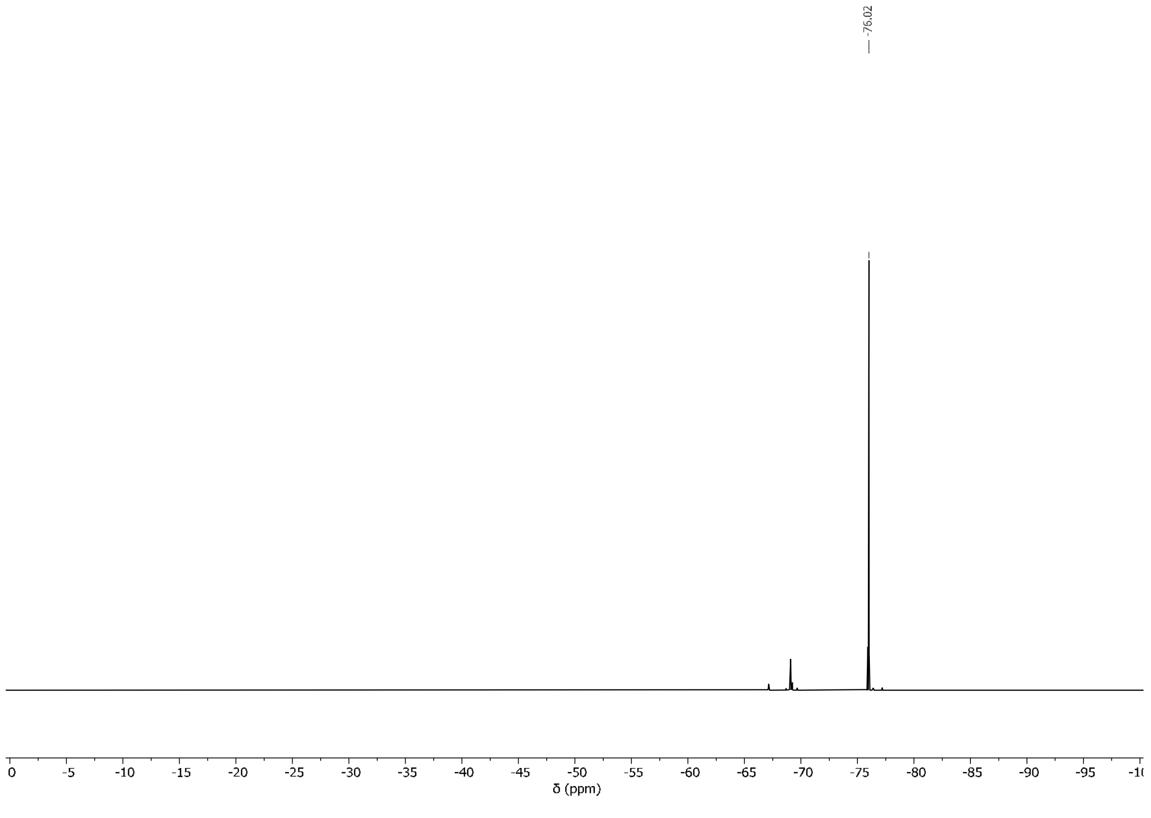
-76.02
0 -5 -10 -15 -20 -25 -30 -35 -40 -45 -50 -55 -60 -65 -70 -75 -80 -85 -90 -95
δ (ppm)

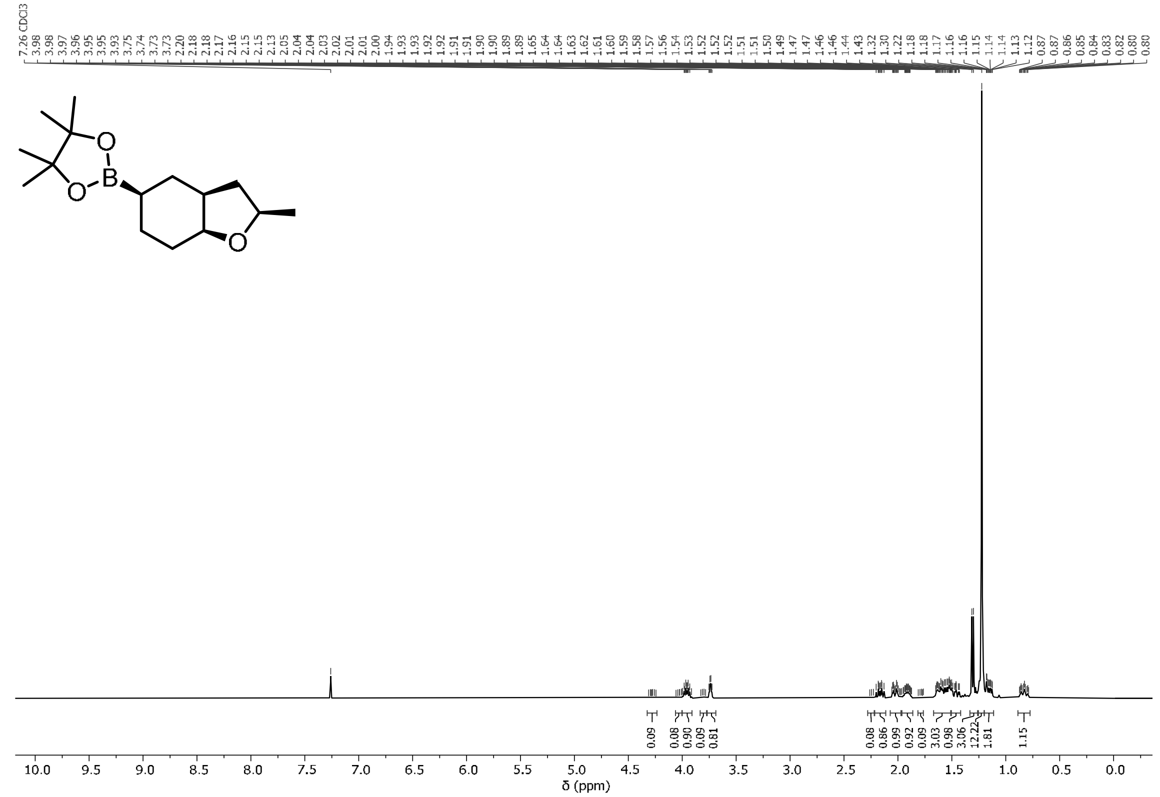
7.26 CDCl3
3.98 3.98 3.97 3.96 3.95 3.95 3.93 3.75 3.74 3.73 3.73 2.20 2.18 2.18 2.17 2.16 2.15 2.15 2.13 2.05 2.04 2.04 2.03 2.02 2.01 2.00 1.94 1.93 1.93 1.92 1.92 1.91 1.91 1.90 1.90 1.89 1.89 1.65 1.64 1.64 1.63 1.62 1.61 1.60 1.59 1.58 1.57 1.56 1.54 1.53 1.52 1.52 1.52 1.51 1.51 1.50 1.49 1.47 1.46 1.46 1.44 1.43 1.32 1.30 1.22 1.18 1.18 1.18 1.17 1.16 1.16 1.15 1.14 1.14 1.14 1.13 1.12 0.87 0.87 0.86 0.85 0.84 0.83 0.82 0.80 0.80
O
B
O
O
0.09
0.08
0.90
0.09
0.81
0.08
0.86
0.99
0.92
0.09
3.03
0.98
3.06
12.22
1.81
1.15
10.0 9.5 9.0 8.5 8.0 7.5 7.0 6.5 6.0 5.5 5.0 4.5 4.0 3.5 3.0 2.5 2.0 1.5 1.0 0.5 0.0
δ (ppm)

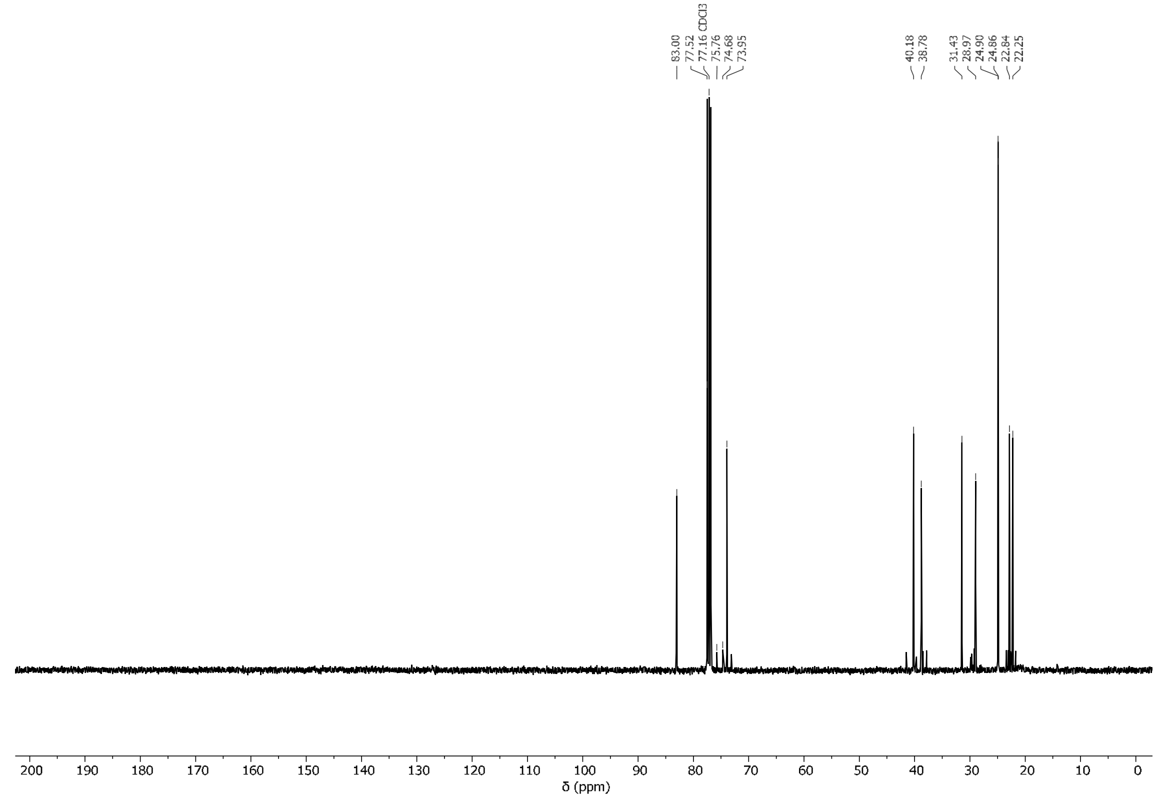
83.00
77.52
77.16 CDCl3
75.76
74.68
73.95
40.18
38.78
31.43
28.97
24.90
24.86
22.84
22.25
200 190 180 170 160 150 140 130 120 110 100 90 80 70 60 50 40 30 20 10 0
δ (ppm)

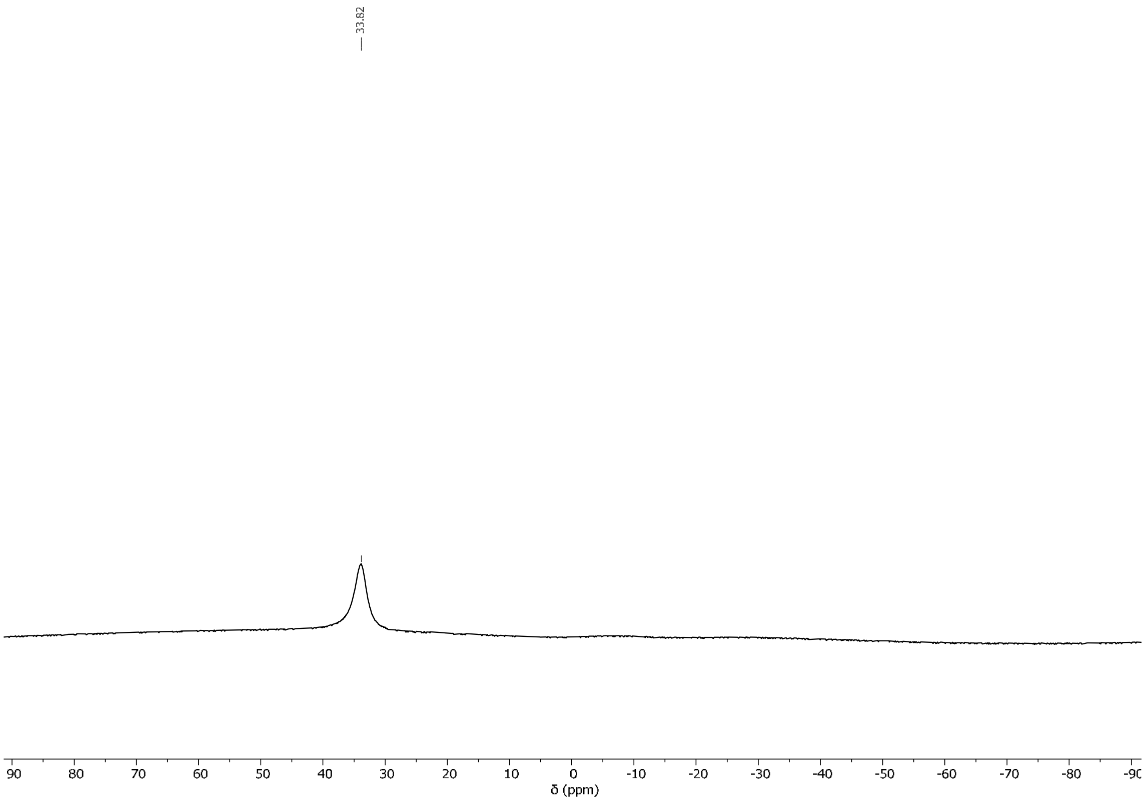
33.82
90 80 70 60 50 40 30 20 10 0 -10 -20 -30 -40 -50 -60 -70 -80 -90
δ (ppm)

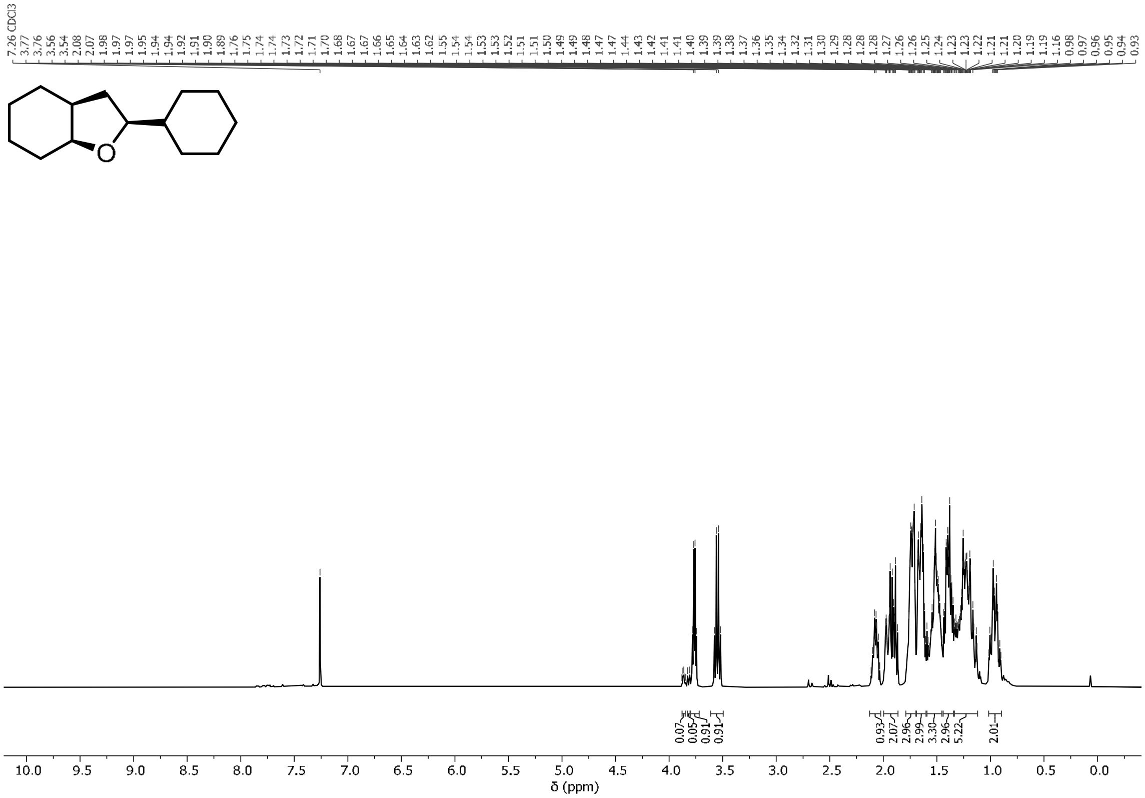
7.26 CDCl3
3.77 3.76 3.56 3.54 2.08 2.07 1.98 1.97 1.97 1.95 1.94 1.94 1.92 1.91 1.90 1.89 1.76 1.75 1.74 1.74 1.73 1.72 1.71 1.70 1.68 1.67 1.67 1.66 1.65 1.64 1.63 1.62 1.55 1.54 1.54 1.53 1.53 1.52 1.51 1.51 1.50 1.49 1.49 1.48 1.47 1.47 1.44 1.43 1.42 1.41 1.41 1.40 1.39 1.39 1.38 1.37 1.36 1.35 1.34 1.32 1.31 1.30 1.29 1.28 1.28 1.28 1.27 1.26 1.26 1.25 1.24 1.23 1.23 1.22 1.21 1.21 1.20 1.19 1.19 1.16 0.98 0.97 0.96 0.95 0.94 0.93
O
0.07 0.05 0.91 0.91
0.93 2.07 2.96 2.99 3.30 2.96 5.22
2.01
10.0 9.5 9.0 8.5 8.0 7.5 7.0 6.5 6.0 5.5 5.0 4.5 4.0 3.5 3.0 2.5 2.0 1.5 1.0 0.5 0.0
δ (ppm)

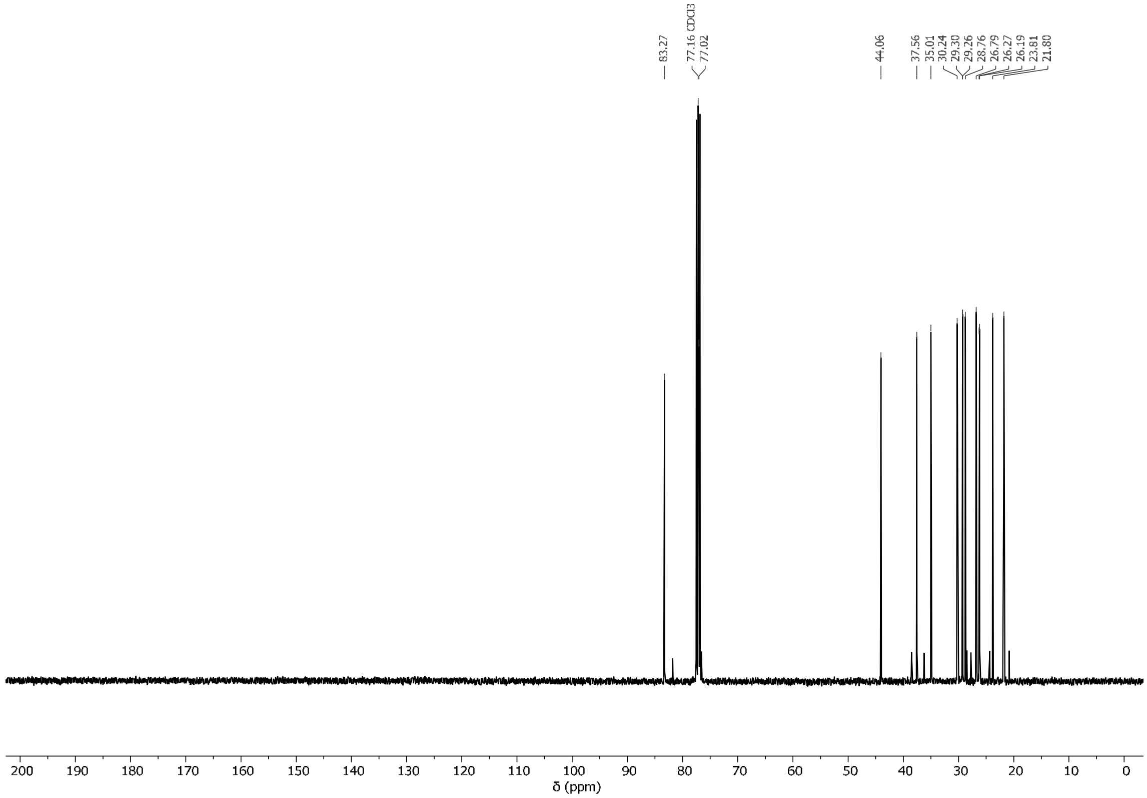

83.27
77.16 CDCl3
77.02
44.06
37.56
35.01
30.24
29.30
29.26
28.76
26.79
26.27
26.19
23.81
21.80
200 190 180 170 160 150 140 130 120 110 100 90 80 70 60 50 40 30 20 10 0
δ (ppm)

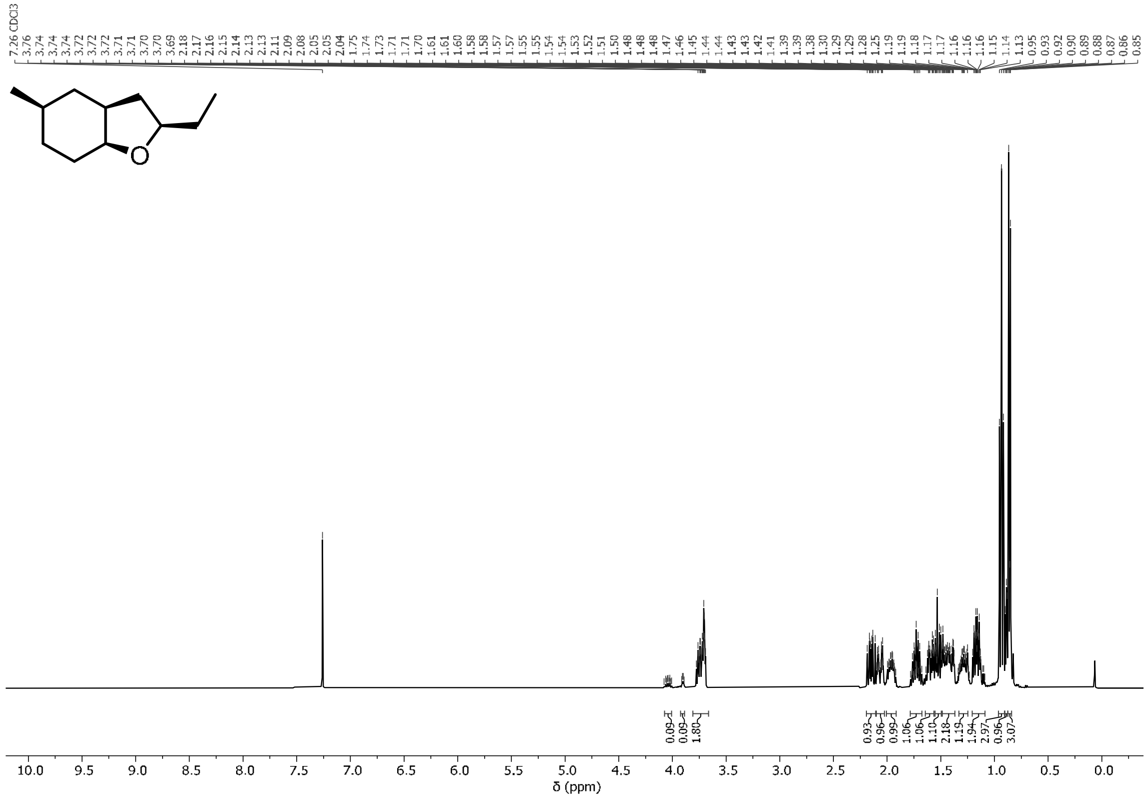

7.26 CDCl3
3.76 3.74 3.74 3.74 3.72 3.72 3.71 3.71 3.70 3.70 3.69 2.18 2.17 2.16 2.15 2.14 2.14 2.13 2.13 2.11 2.09 2.08 2.05 2.05 2.04 1.75 1.74 1.73 1.71 1.71 1.70 1.61 1.60 1.58 1.58 1.57 1.57 1.55 1.55 1.54 1.54 1.53 1.52 1.51 1.50 1.48 1.48 1.48 1.47 1.46 1.45 1.44 1.44 1.43 1.43 1.42 1.41 1.39 1.39 1.38 1.30 1.29 1.29 1.28 1.25 1.19 1.19 1.18 1.17 1.17 1.16 1.16 1.16 1.15 1.14 1.13 0.95 0.93 0.92 0.90 0.89 0.88 0.87 0.86 0.85
O
0.09 0.09 1.80
0.93 0.96 0.99 1.06 1.06 1.10 2.18 1.19 1.94 2.97 0.96 3.07
10.0 9.5 9.0 8.5 8.0 7.5 7.0 6.5 6.0 5.5 5.0 4.5 4.0 3.5 3.0 2.5 2.0 1.5 1.0 0.5 0.0
δ (ppm)

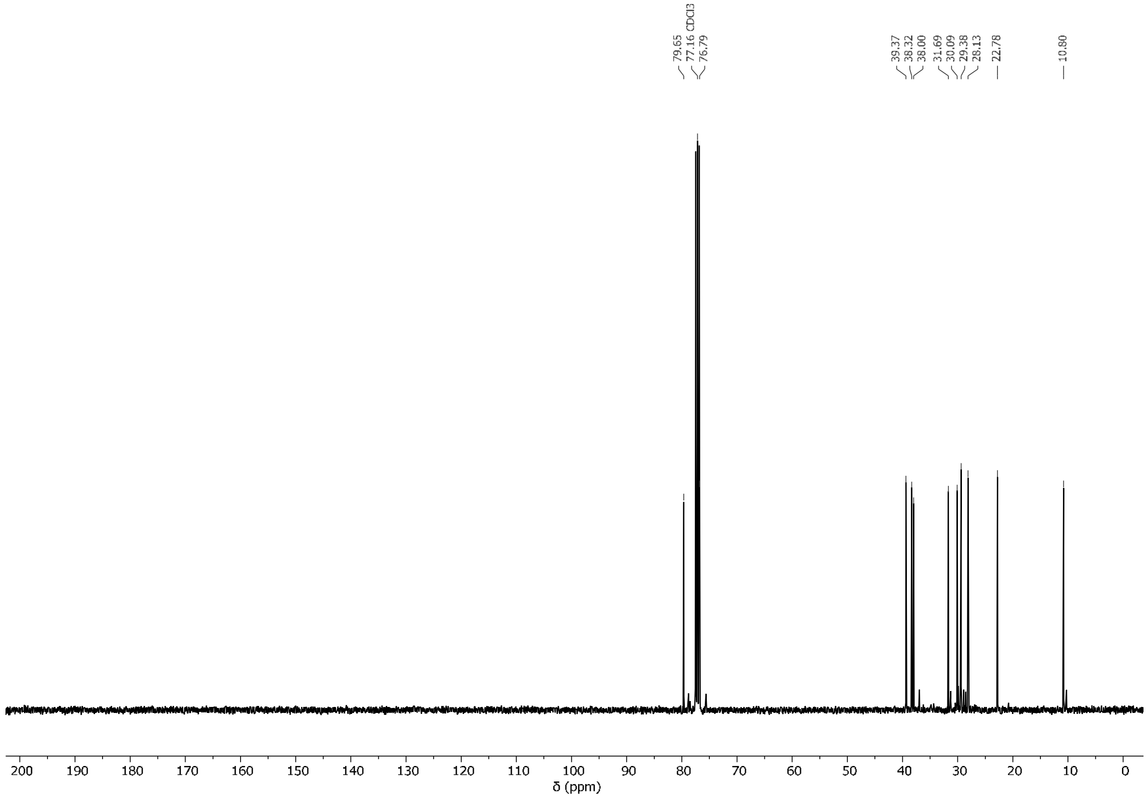
79.65
77.16 CDCl3
76.79
39.37
38.32
38.00
31.69
30.09
29.38
28.13
22.78
10.80
200 190 180 170 160 150 140 130 120 110 100 90 80 70 60 50 40 30 20 10 0
δ (ppm)

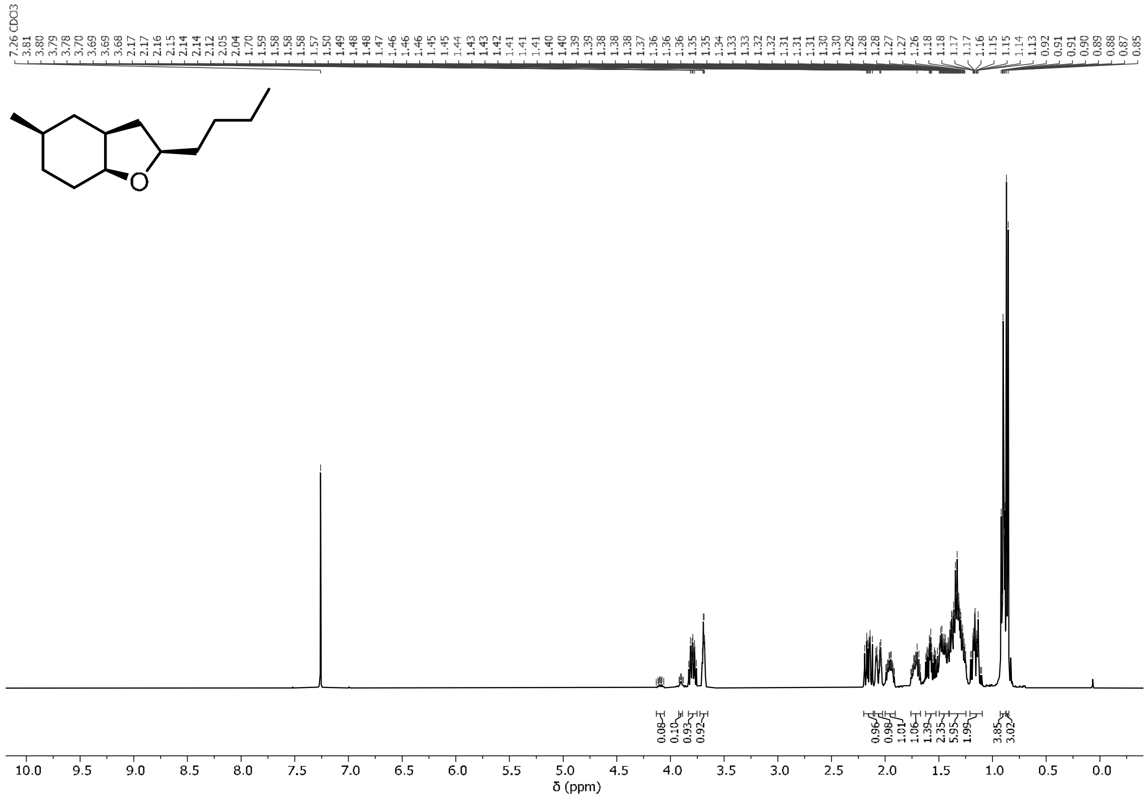
7.26 CDCl3
3.81
3.80
3.79
3.78
3.70
3.69
3.69
3.68
2.17
2.17
2.16
2.15
2.14
2.14
2.12
2.05
2.04
1.70
1.59
1.58
1.58
1.58
1.57
1.50
1.49
1.48
1.48
1.47
1.46
1.46
1.46
1.45
1.45
1.44
1.43
1.43
1.42
1.41
1.41
1.41
1.40
1.40
1.39
1.39
1.38
1.38
1.38
1.37
1.36
1.36
1.36
1.35
1.35
1.34
1.33
1.33
1.32
1.32
1.31
1.31
1.31
1.30
1.30
1.29
1.28
1.28
1.27
1.27
1.26
1.18
1.18
1.17
1.17
1.16
1.15
1.15
1.14
1.13
0.92
0.91
0.91
0.90
0.89
0.88
0.87
0.85
O
0.08
0.10
0.93
0.92
0.96
0.98
1.01
1.06
1.39
2.35
5.95
1.99
3.85
3.02
10.0 9.5 9.0 8.5 8.0 7.5 7.0 6.5 6.0 5.5 5.0 4.5 4.0 3.5 3.0 2.5 2.0 1.5 1.0 0.5 0.0
δ (ppm)

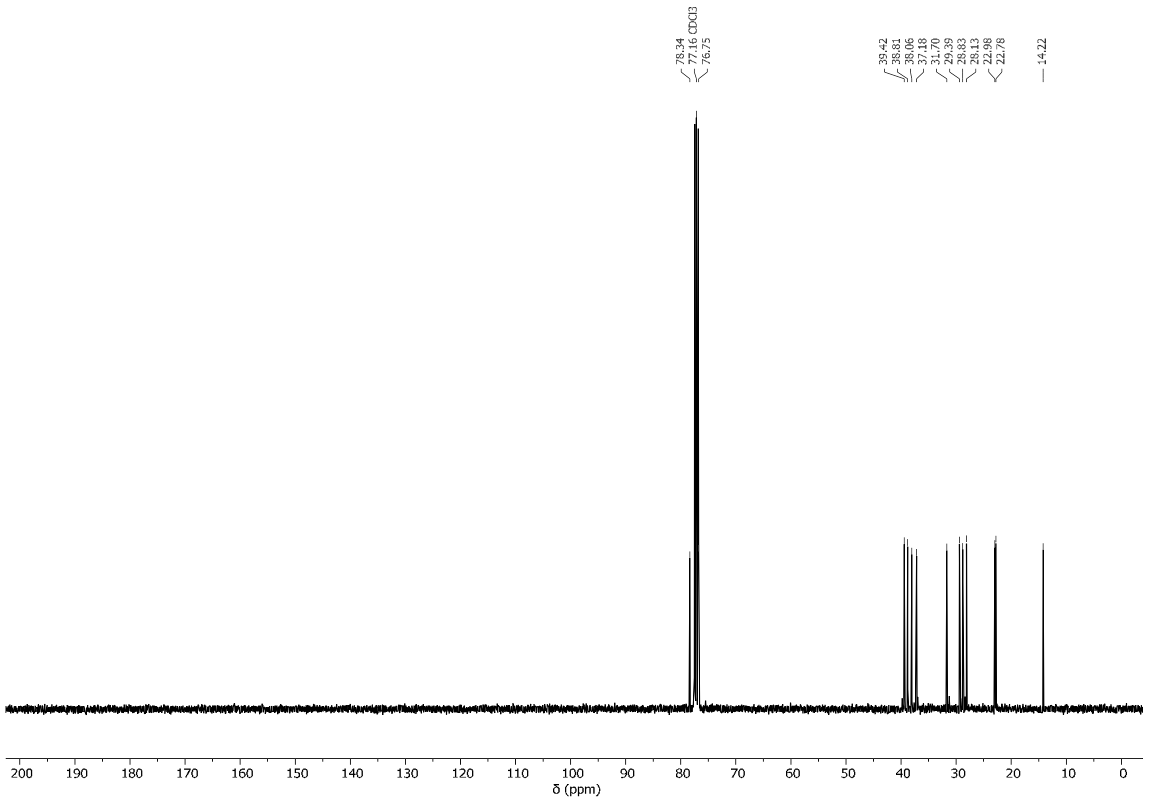
78.34
77.16 CDCl3
76.75
39.42
38.81
38.06
37.18
31.70
29.39
28.83
28.13
22.98
22.78
14.22
δ (ppm)

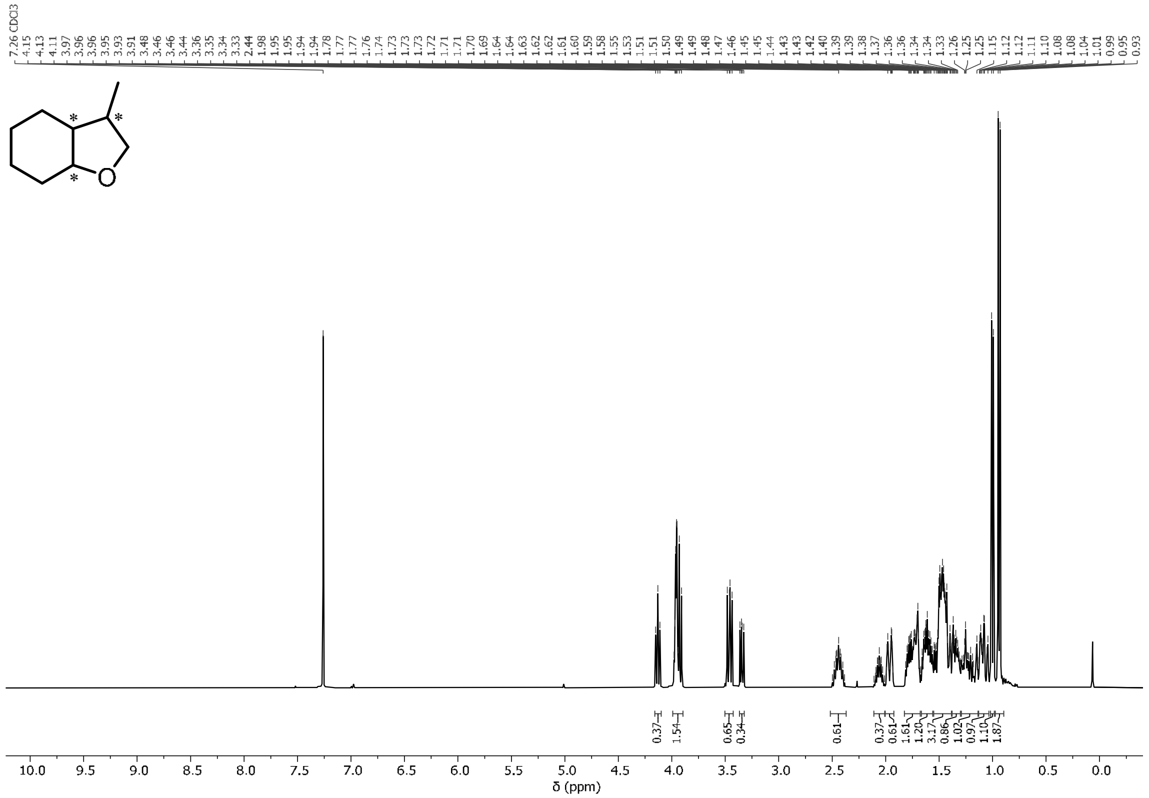
7.26 CDCl3
4.15
4.13
4.11
3.97
3.96
3.96
3.95
3.93
3.91
3.48
3.46
3.46
3.44
3.36
3.35
3.34
3.33
2.44
1.98
1.95
1.95
1.94
1.94
1.78
1.77
1.77
1.76
1.74
1.73
1.73
1.72
1.71
1.71
1.70
1.69
1.64
1.64
1.63
1.62
1.62
1.61
1.60
1.59
1.58
1.55
1.53
1.51
1.51
1.50
1.49
1.49
1.48
1.47
1.46
1.45
1.45
1.44
1.43
1.43
1.42
1.40
1.39
1.39
1.38
1.37
1.36
1.36
1.34
1.34
1.33
1.26
1.25
1.25
1.15
1.12
1.12
1.11
1.10
1.08
1.08
1.04
1.01
0.99
0.95
0.93
O
0.37
1.54
0.65
0.34
0.61
0.37
0.61
1.61
1.20
3.17
0.86
1.02
0.97
1.10
1.87
δ (ppm)

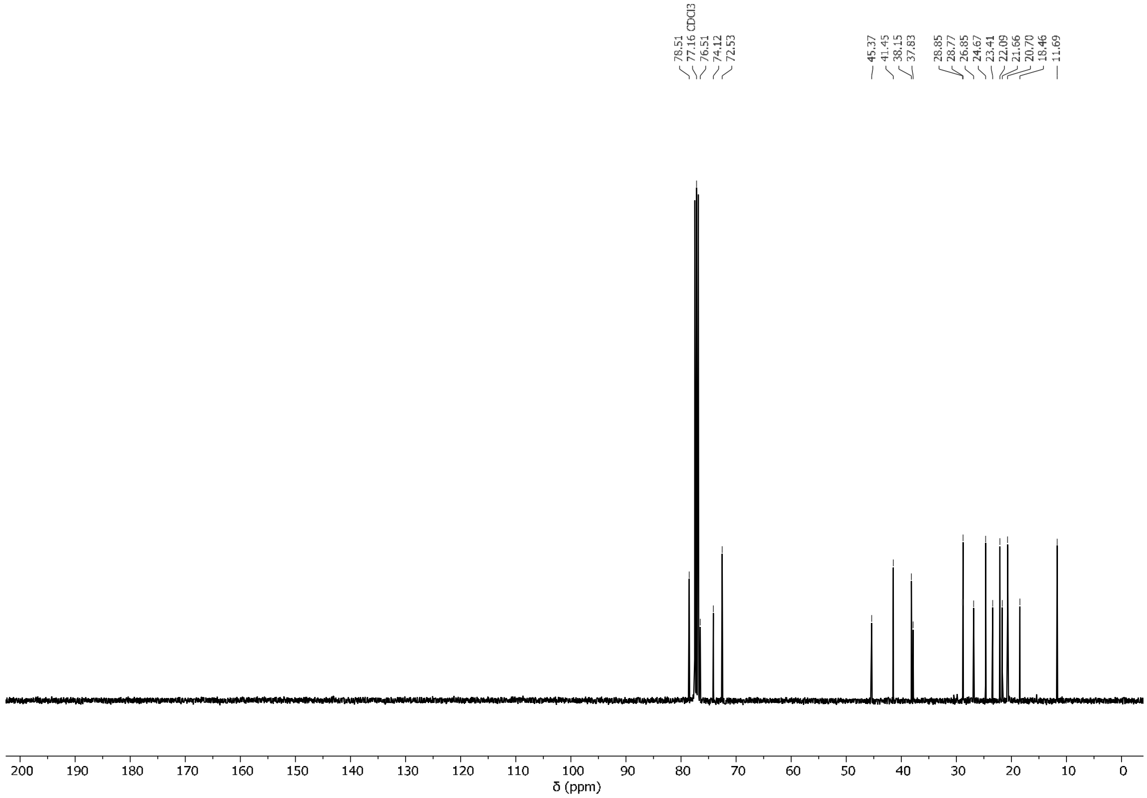
78.51
77.16 CDCl3
76.51
74.12
72.53
45.37
41.45
38.15
37.83
28.85
28.77
26.85
24.67
23.41
22.09
21.66
20.70
18.46
11.69
200 190 180 170 160 150 140 130 120 110 100 90 80 70 60 50 40 30 20 10 0
δ (ppm)

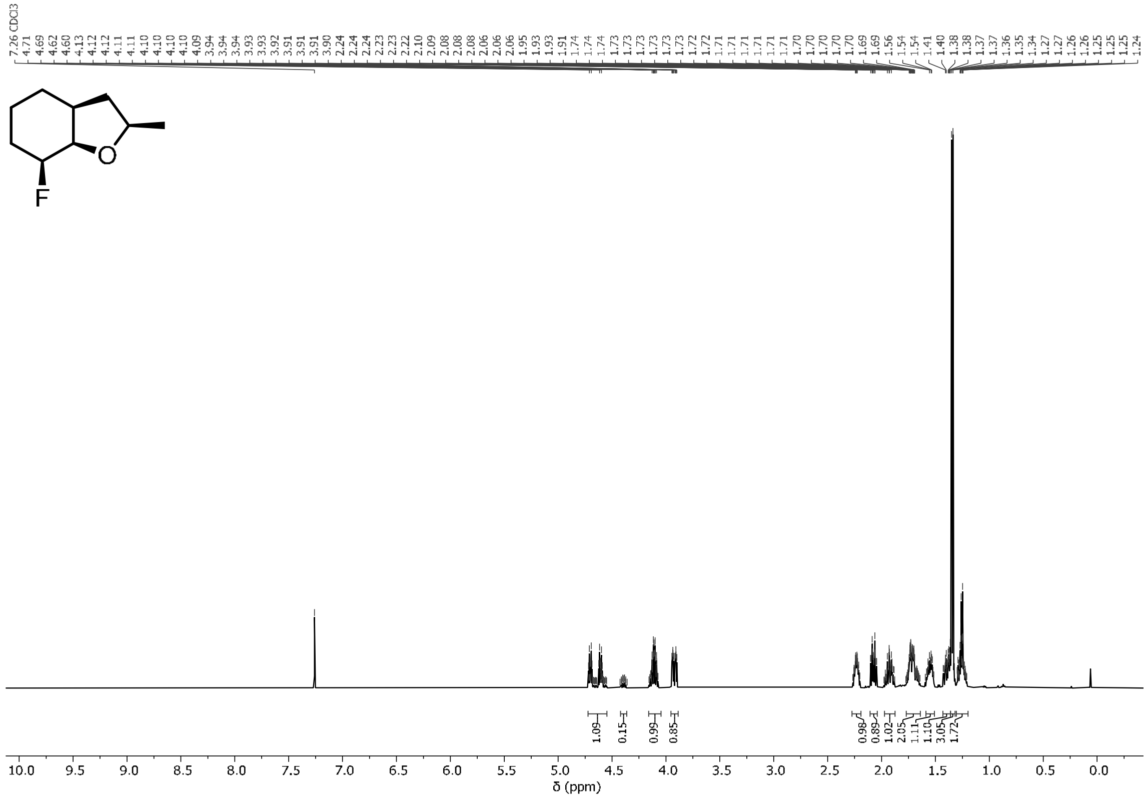
O
F
7.26 CDCl3
4.71
4.69
4.62
4.60
4.13
4.12
4.12
4.11
4.11
4.10
4.10
4.10
4.10
4.09
3.94
3.94
3.93
3.93
3.92
3.91
3.91
3.91
3.90
2.24
2.24
2.24
2.23
2.23
2.22
2.10
2.09
2.08
2.08
2.08
2.06
2.06
2.06
1.95
1.93
1.93
1.91
1.74
1.74
1.73
1.73
1.73
1.73
1.73
1.72
1.72
1.71
1.71
1.71
1.71
1.71
1.70
1.70
1.70
1.70
1.70
1.69
1.69
1.56
1.54
1.54
1.54
1.41
1.40
1.38
1.38
1.37
1.37
1.36
1.35
1.34
1.27
1.27
1.26
1.26
1.25
1.25
1.25
1.24
1.09
0.15
0.99
0.85
0.98
0.89
1.02
2.05
1.11
1.10
3.05
1.72
10.0 9.5 9.0 8.5 8.0 7.5 7.0 6.5 6.0 5.5 5.0 4.5 4.0 3.5 3.0 2.5 2.0 1.5 1.0 0.5 0.0
δ (ppm)

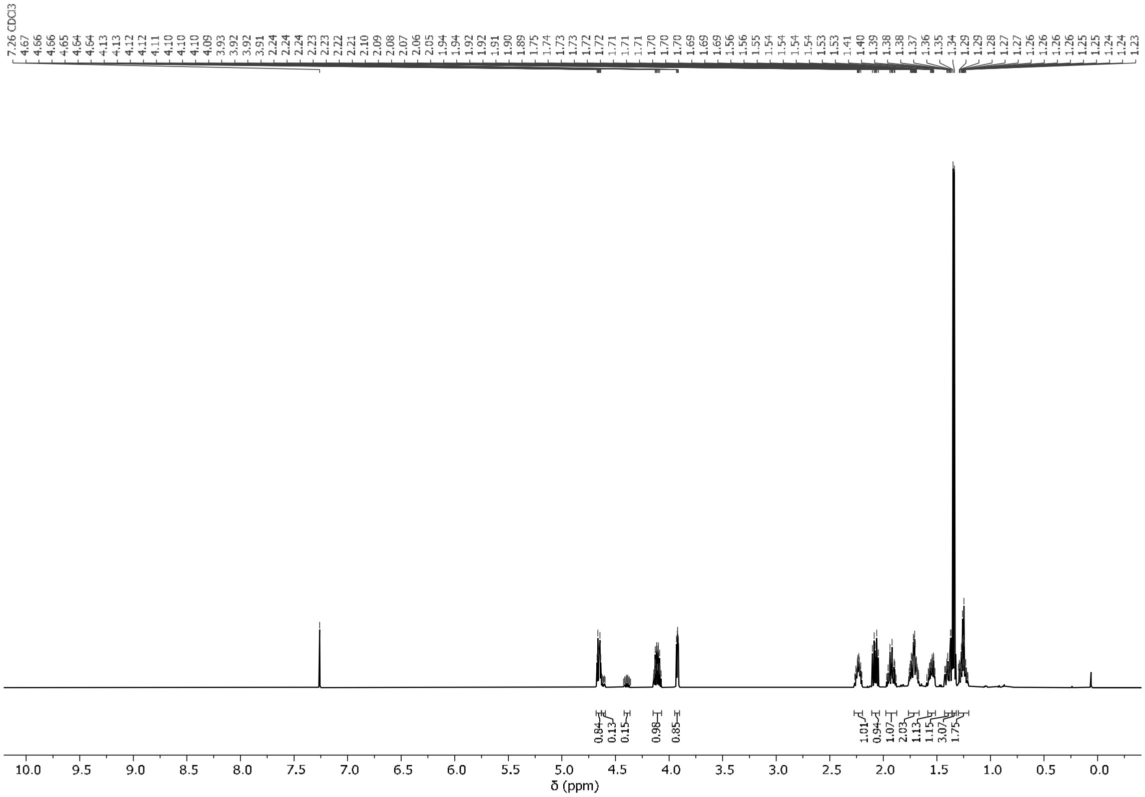
7.26 CDCl3
4.67 4.66 4.66 4.65 4.64 4.64 4.13 4.13 4.12 4.12 4.11 4.10 4.10 4.10 4.09 3.93 3.92 3.92 3.92 3.91 2.24 2.24 2.24 2.23 2.23 2.22 2.21 2.10 2.09 2.08 2.07 2.06 2.05 1.94 1.94 1.92 1.92 1.91 1.90 1.89 1.75 1.74 1.73 1.73 1.72 1.72 1.72 1.71 1.71 1.71 1.71 1.70 1.70 1.70 1.69 1.69 1.69 1.56 1.56 1.55 1.54 1.54 1.54 1.54 1.54 1.53 1.53 1.41 1.40 1.39 1.38 1.38 1.37 1.36 1.35 1.34 1.29 1.29 1.28 1.27 1.27 1.26 1.26 1.26 1.25 1.25 1.24 1.24 1.23
0.84 0.13 0.15 0.98 0.85
1.01 0.94 1.07 2.03 1.13 1.15 3.07 1.75
10.0 9.5 9.0 8.5 8.0 7.5 7.0 6.5 6.0 5.5 5.0 4.5 4.0 3.5 3.0 2.5 2.0 1.5 1.0 0.5 0.0
δ (ppm)

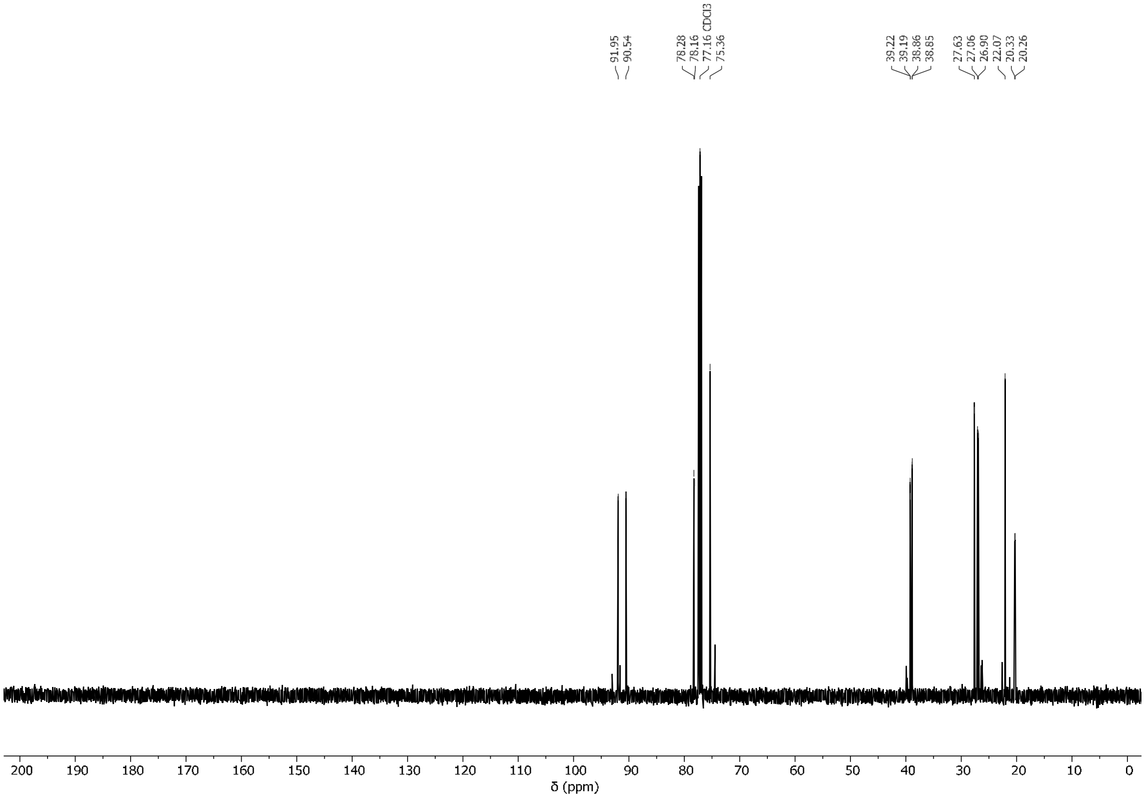
91.95
90.54
78.28
78.16
77.16 CDCl3
75.36
39.22
39.19
38.86
38.85
27.63
27.06
26.90
22.07
20.33
20.26
200 190 180 170 160 150 140 130 120 110 100 90 80 70 60 50 40 30 20 10 0
δ (ppm)

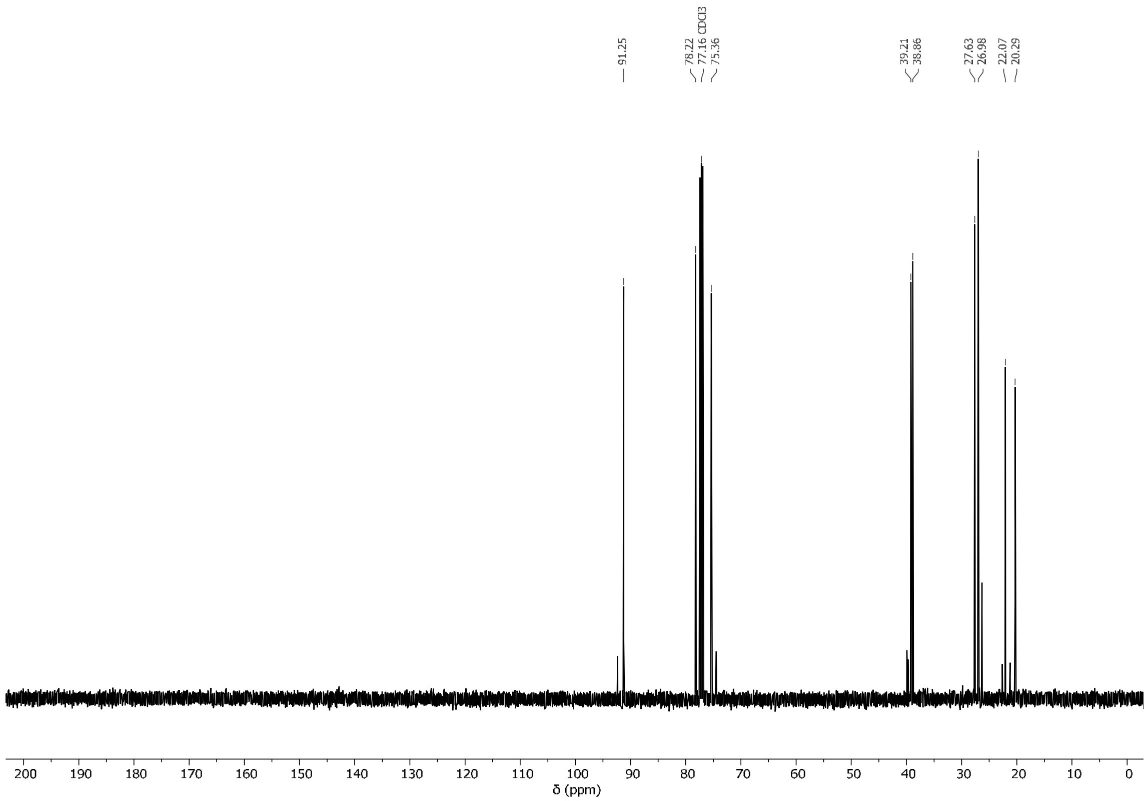
91.25
78.22
77.16 CDCl3
75.36
39.21
38.86
27.63
26.98
22.07
20.29
200 190 180 170 160 150 140 130 120 110 100 90 80 70 60 50 40 30 20 10 0
δ (ppm)

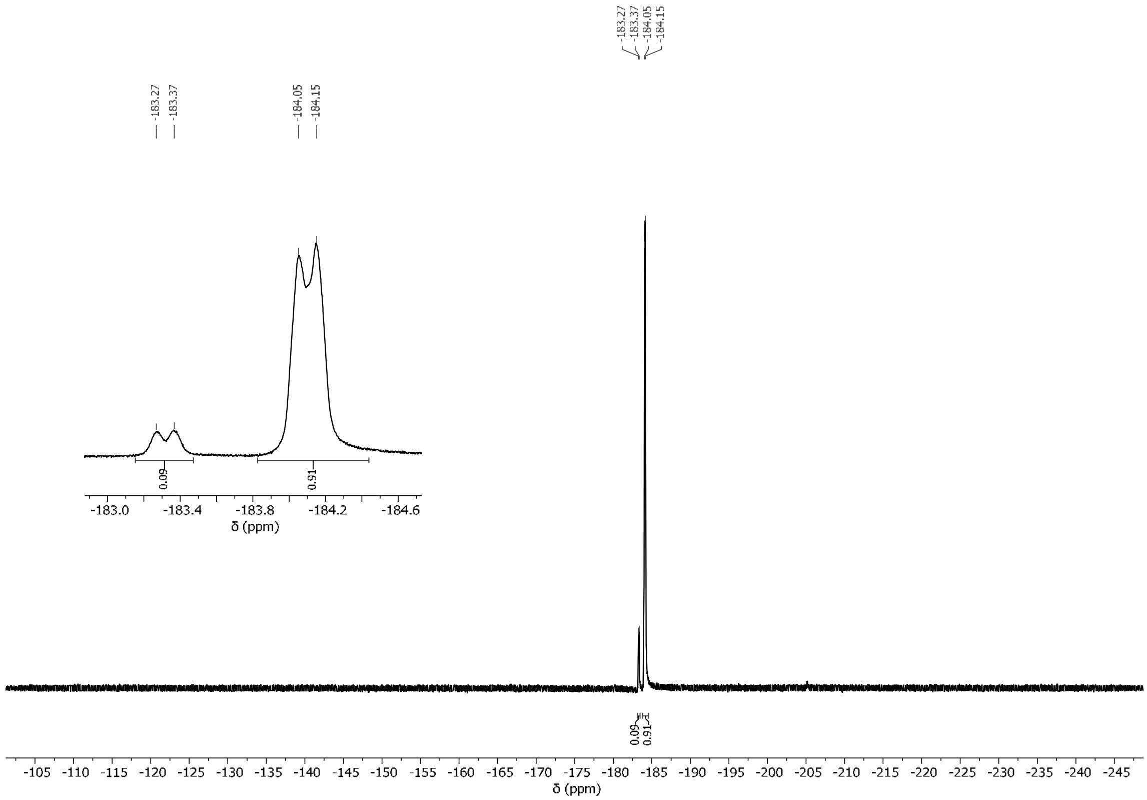
-183.27
-183.37
-184.05
-184.15
0.09
0.91
-183.0 -183.4 -183.8 -184.2 -184.6
δ (ppm)
-105 -110 -115 -120 -125 -130 -135 -140 -145 -150 -155 -160 -165 -170 -175 -180 -185 -190 -195 -200 -205 -210 -215 -220 -225 -230 -235 -240 -245
δ (ppm)

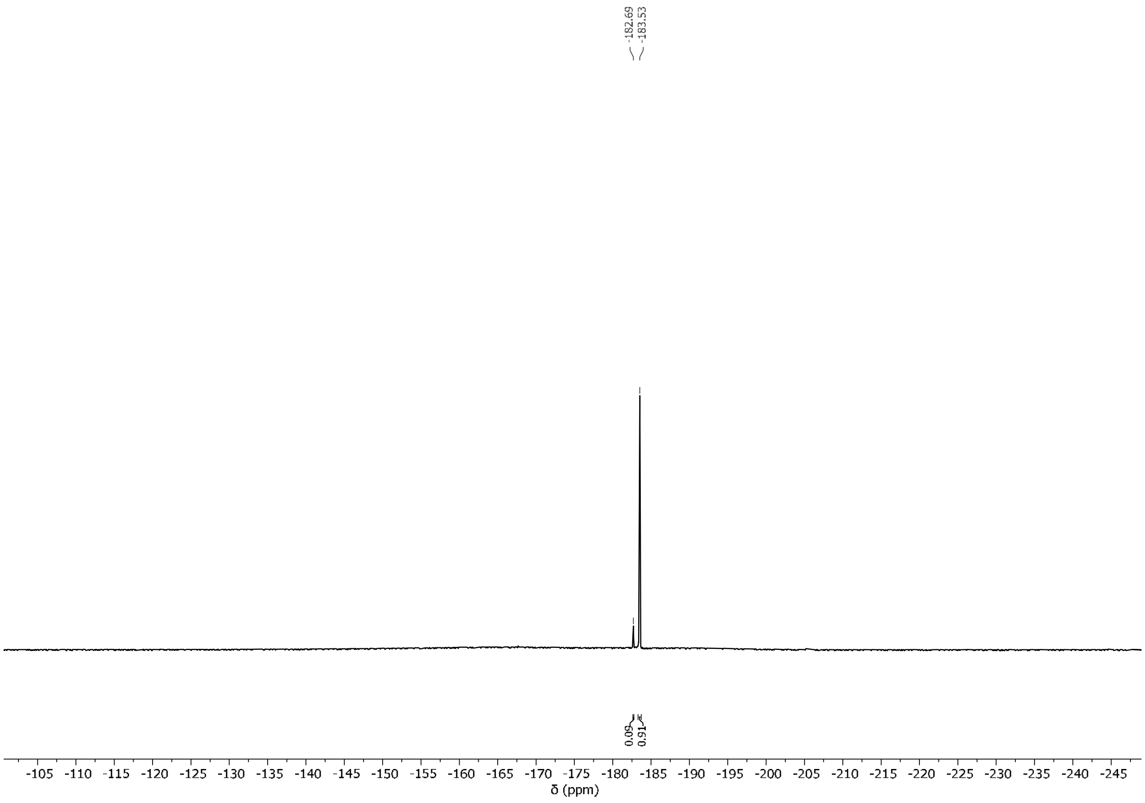

-182.69
-183.53
0.09
0.91
-105 -110 -115 -120 -125 -130 -135 -140 -145 -150 -155 -160 -165 -170 -175 -180 -185 -190 -195 -200 -205 -210 -215 -220 -225 -230 -235 -240 -245
δ (ppm)

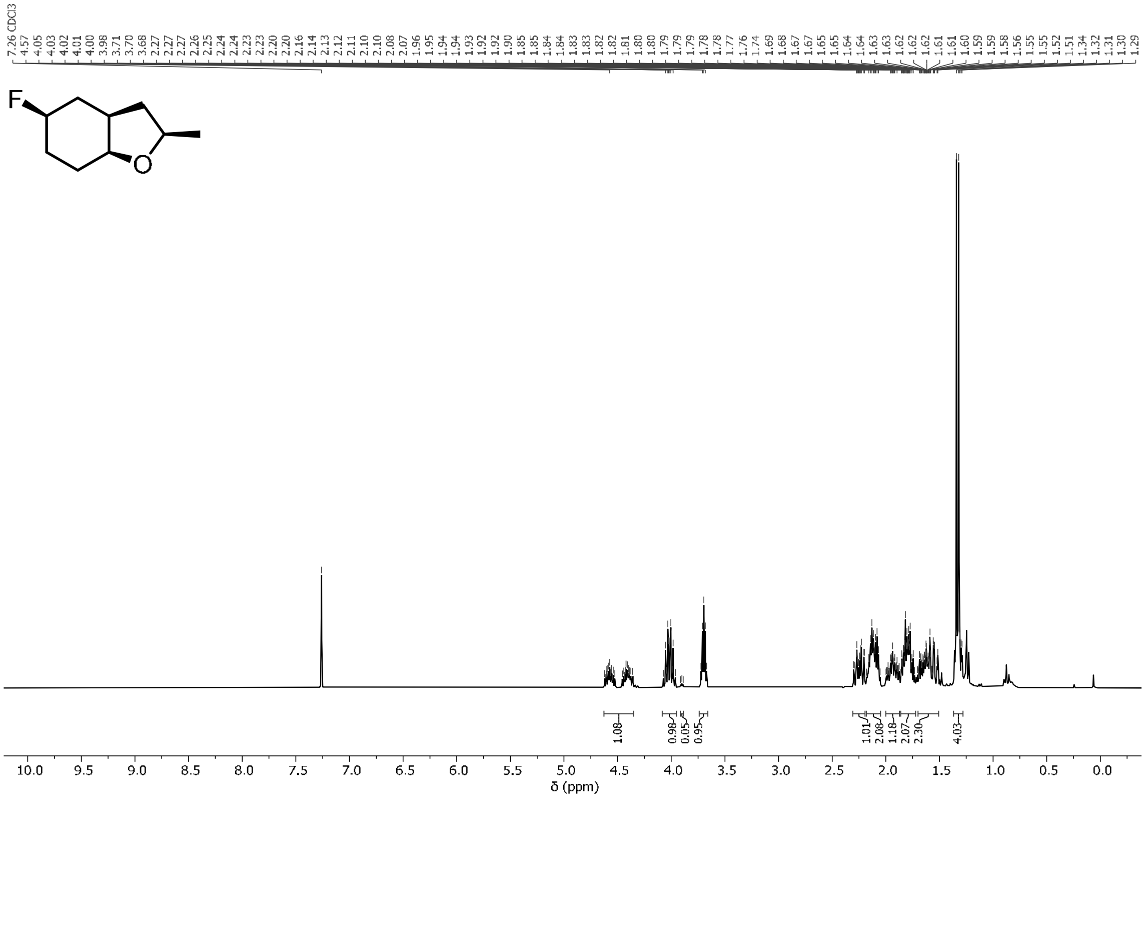

7.26 CDCl3
4.57 4.05 4.03 4.02 4.01 4.00 3.98 3.71 3.70 3.68 2.27 2.27 2.27 2.26 2.25 2.24 2.24 2.23 2.23 2.20 2.20 2.16 2.14 2.13 2.12 2.11 2.10 2.10 2.08 2.07 1.96 1.95 1.94 1.94 1.93 1.92 1.92 1.90 1.85 1.85 1.84 1.84 1.83 1.83 1.82 1.82 1.81 1.80 1.80 1.79 1.79 1.79 1.78 1.78 1.77 1.76 1.74 1.69 1.68 1.67 1.67 1.65 1.65 1.64 1.63 1.63 1.62 1.62 1.62 1.61 1.61 1.60 1.59 1.59 1.58 1.56 1.55 1.55 1.52 1.51 1.34 1.32 1.31 1.30 1.29
F
O
1.08
0.98
0.05
0.95
1.01
2.08
1.18
2.07
2.30
4.03
10.0 9.5 9.0 8.5 8.0 7.5 7.0 6.5 6.0 5.5 5.0 4.5 4.0 3.5 3.0 2.5 2.0 1.5 1.0 0.5 0.0
δ (ppm)

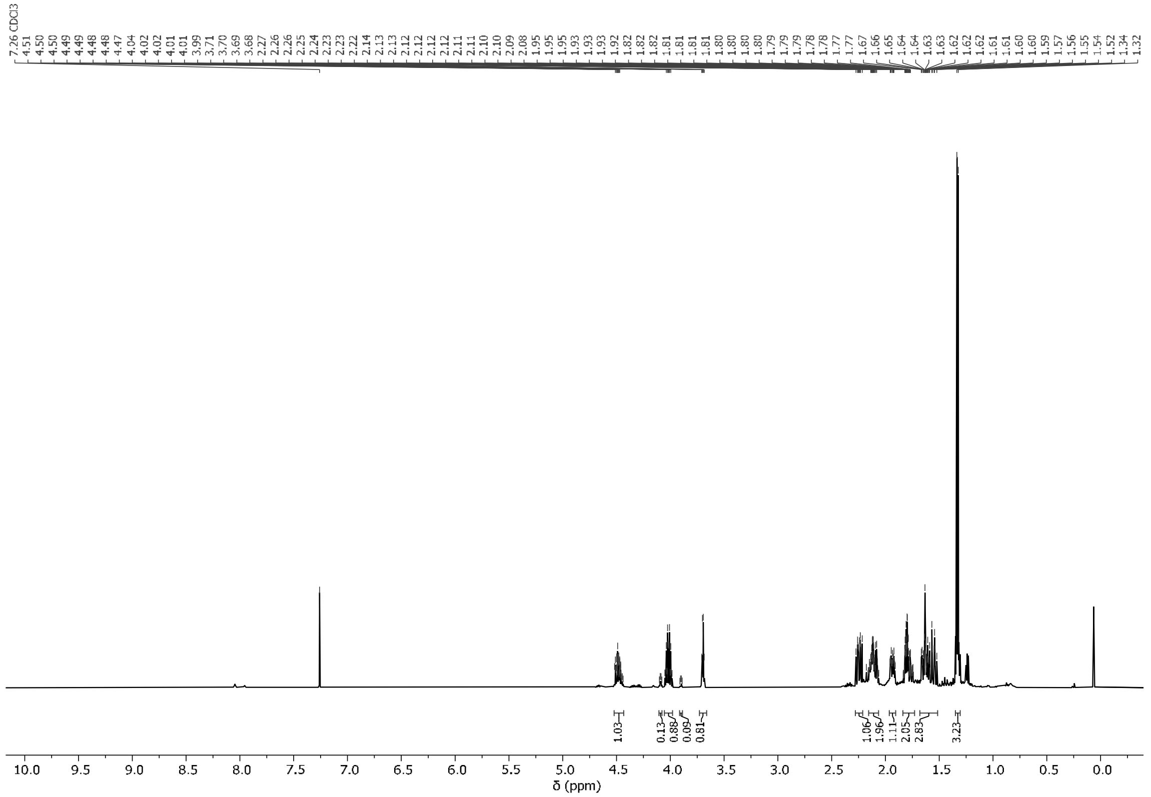
7.26 CDCl3
4.51
4.50
4.50
4.49
4.49
4.48
4.48
4.47
4.04
4.02
4.02
4.01
4.01
3.99
3.71
3.70
3.69
3.68
2.27
2.26
2.26
2.25
2.24
2.23
2.23
2.22
2.14
2.13
2.13
2.12
2.12
2.12
2.12
2.12
2.11
2.11
2.10
2.10
2.09
2.08
1.95
1.95
1.95
1.93
1.93
1.93
1.92
1.92
1.82
1.82
1.82
1.81
1.81
1.81
1.81
1.81
1.80
1.80
1.80
1.80
1.79
1.79
1.79
1.78
1.78
1.77
1.77
1.67
1.66
1.65
1.64
1.64
1.63
1.63
1.62
1.62
1.62
1.61
1.61
1.60
1.60
1.59
1.57
1.56
1.55
1.54
1.52
1.34
1.32
1.03
0.13
0.88
0.09
0.81
1.06
1.96
1.11
2.05
2.83
3.23
δ (ppm)

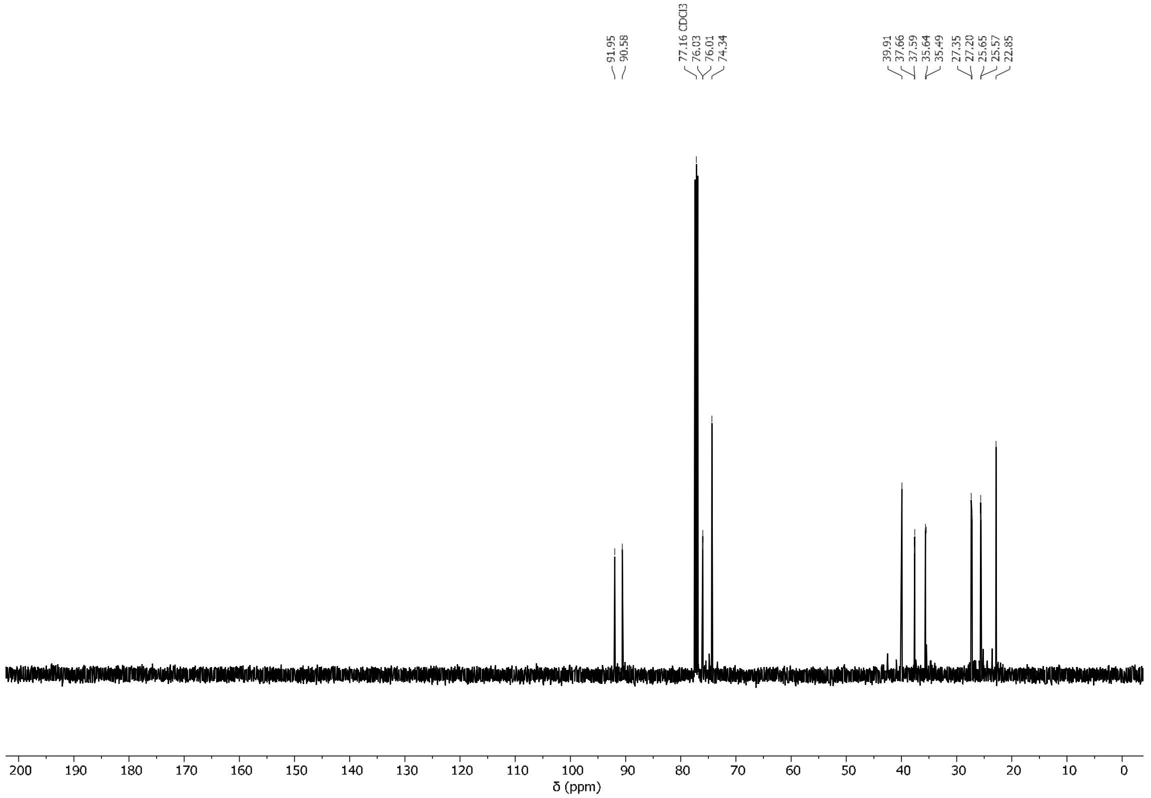
91.95
90.58
77.16 CDCl3
76.03
76.01
74.34
39.91
37.66
37.59
35.64
35.49
27.35
27.20
25.65
25.57
22.85
δ (ppm)

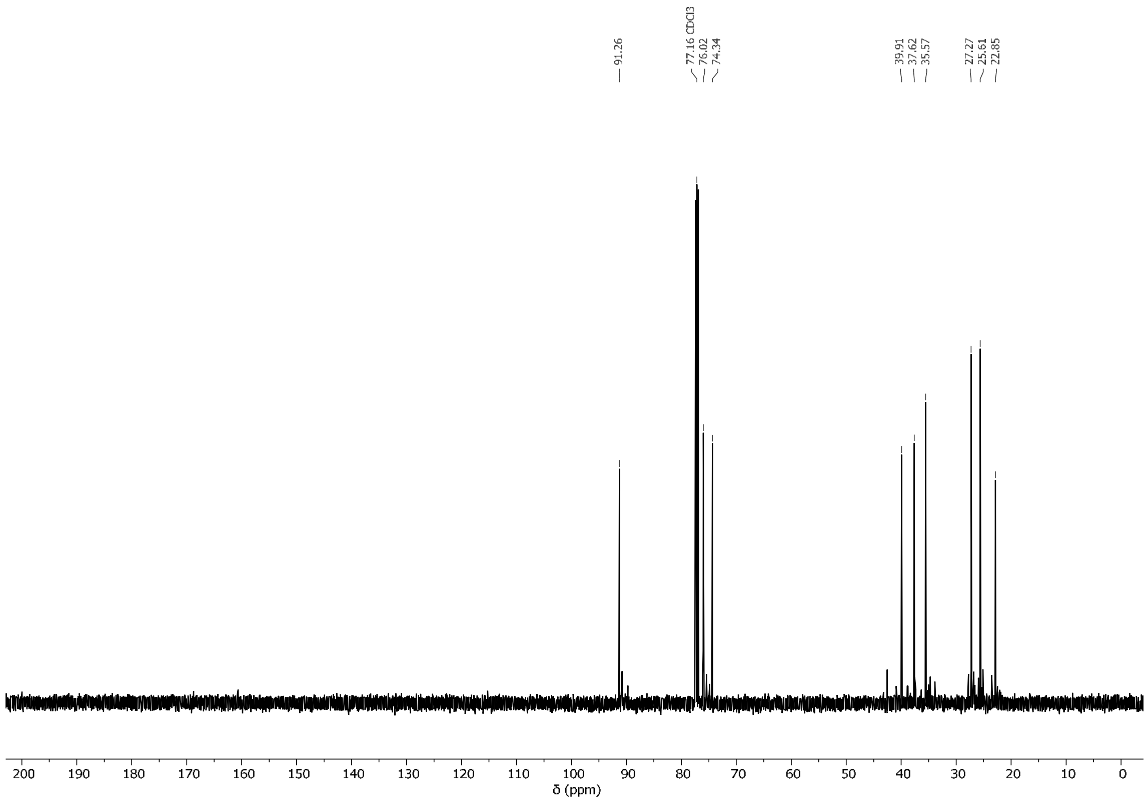
91.26
77.16 CDCl3
76.02
74.34
39.91
37.62
35.57
27.27
25.61
22.85
200 190 180 170 160 150 140 130 120 110 100 90 80 70 60 50 40 30 20 10 0
δ (ppm)

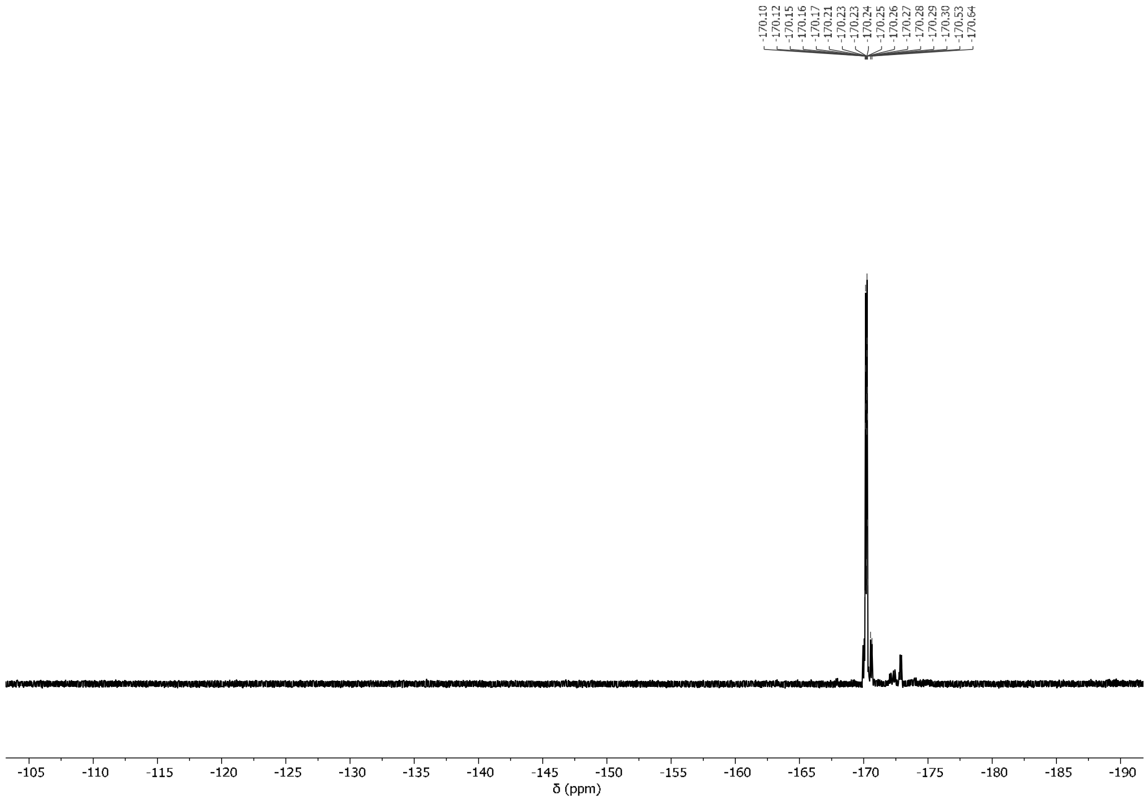
-170.10
-170.12
-170.15
-170.16
-170.17
-170.21
-170.23
-170.23
-170.24
-170.25
-170.26
-170.27
-170.28
-170.29
-170.30
-170.53
-170.64
-105 -110 -115 -120 -125 -130 -135 -140 -145 -150 -155 -160 -165 -170 -175 -180 -185 -190
δ (ppm)

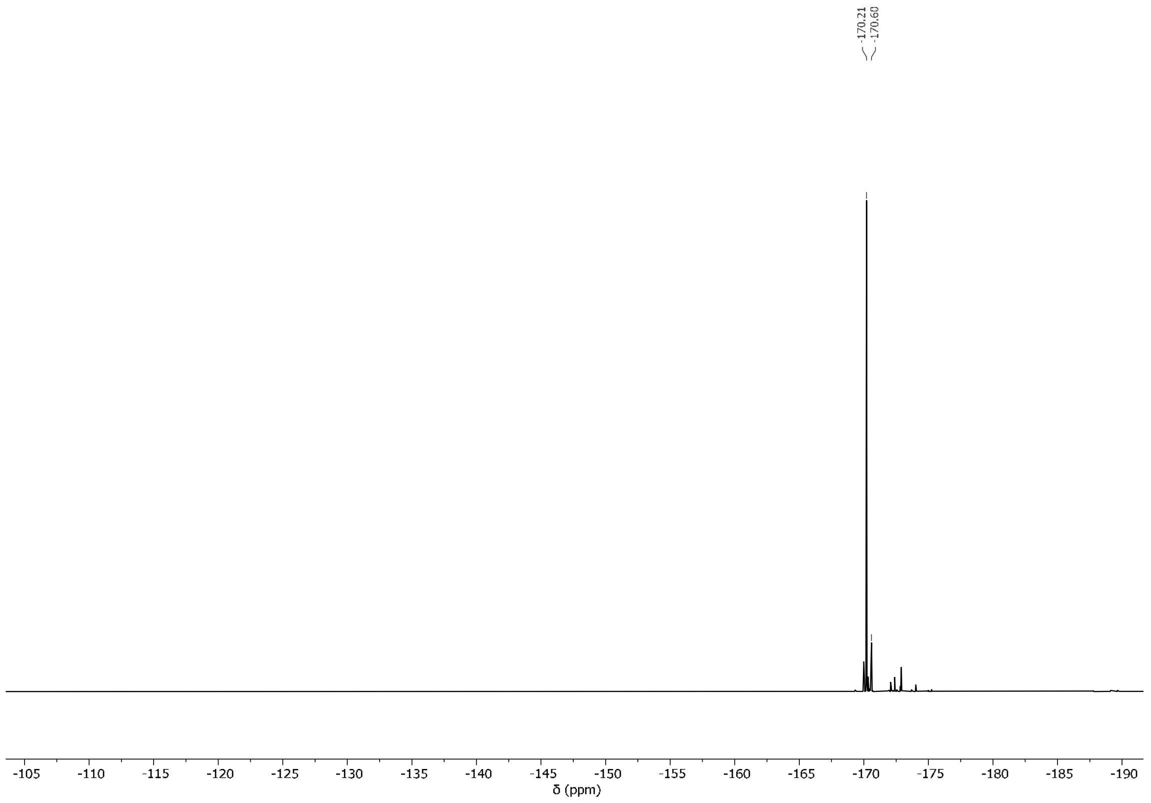
-170.21
-170.60
-105 -110 -115 -120 -125 -130 -135 -140 -145 -150 -155 -160 -165 -170 -175 -180 -185 -190
δ (ppm)

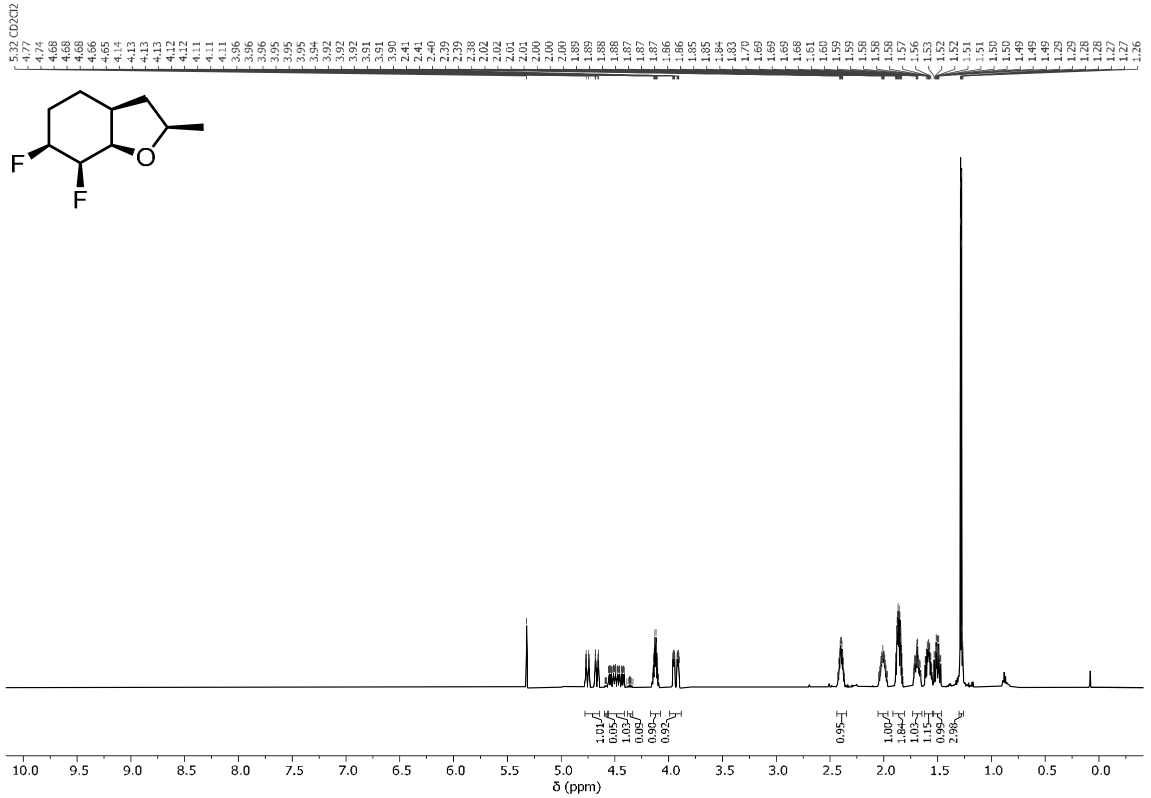
5.32 CD2Cl2
4.77 4.74 4.68 4.68 4.68 4.66 4.65 4.14 4.13 4.13 4.12 4.12 4.11 4.11 4.11 3.96 3.96 3.95 3.95 3.95 3.94 3.92 3.92 3.91 3.91 3.90 2.41 2.41 2.40 2.39 2.39 2.38 2.02 2.02 2.01 2.01 2.00 2.00 2.00 1.89 1.88 1.88 1.87 1.87 1.87 1.86 1.86 1.85 1.85 1.84 1.83 1.70 1.69 1.69 1.68 1.61 1.60 1.59 1.59 1.58 1.58 1.57 1.56 1.53 1.52 1.52 1.51 1.51 1.50 1.50 1.49 1.49 1.29 1.28 1.28 1.27 1.27 1.26
F
F
O
1.01 0.05 1.03 0.09 0.90 0.92 0.95 1.00 1.84 1.03 1.15 0.99 2.98
10.0 9.5 9.0 8.5 8.0 7.5 7.0 6.5 6.0 5.5 5.0 4.5 4.0 3.5 3.0 2.5 2.0 1.5 1.0 0.5 0.0
δ (ppm)

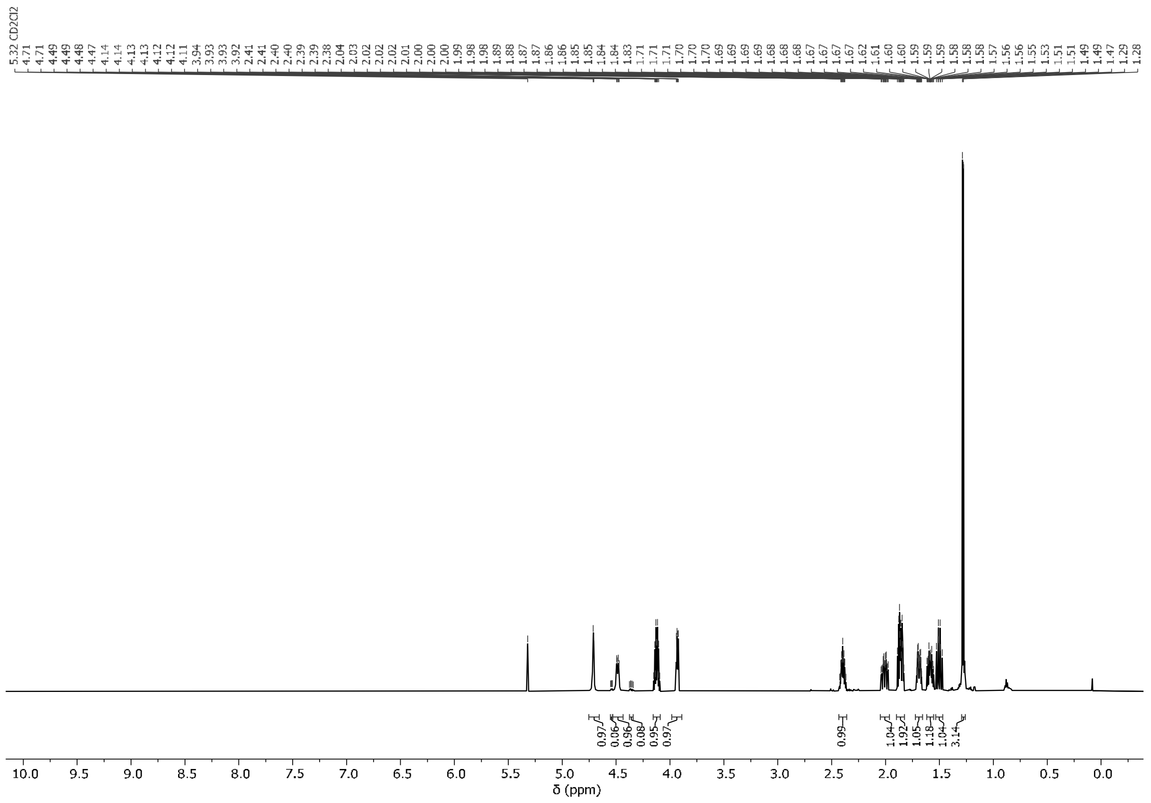
5.32 CD2Cl2
δ (ppm)

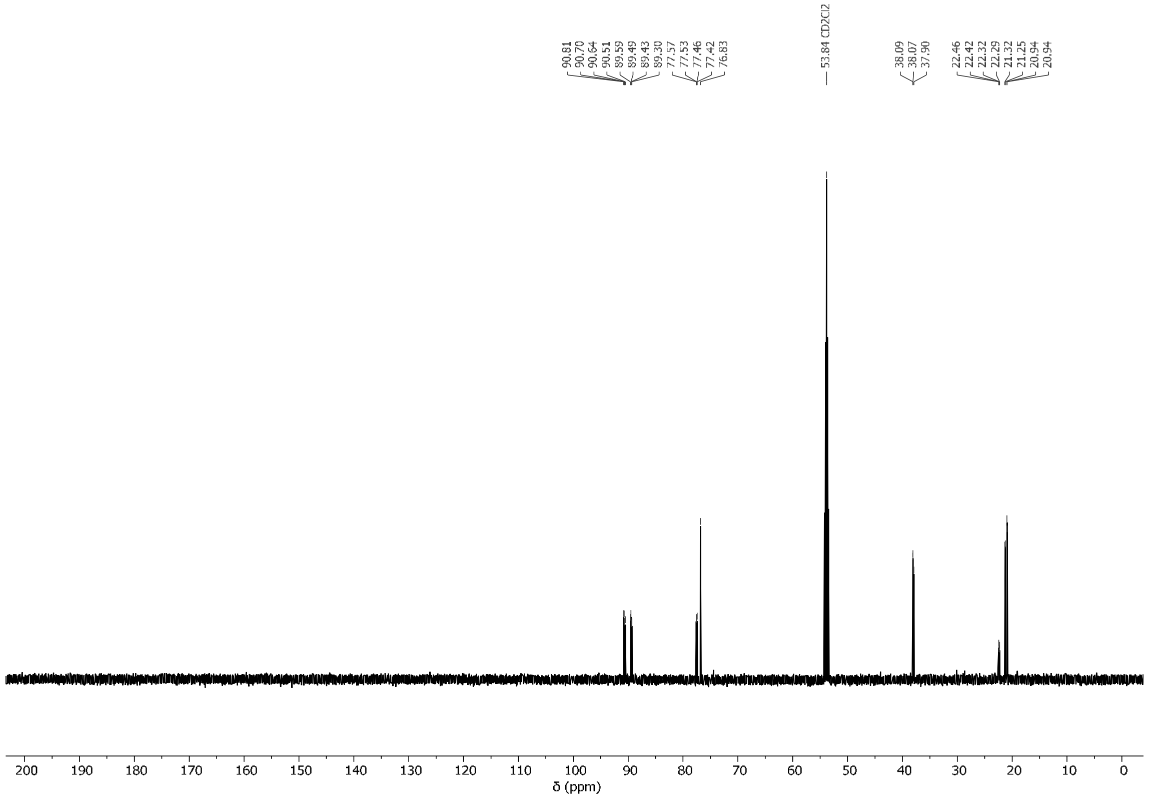
53.84 CD2Cl2
δ (ppm)

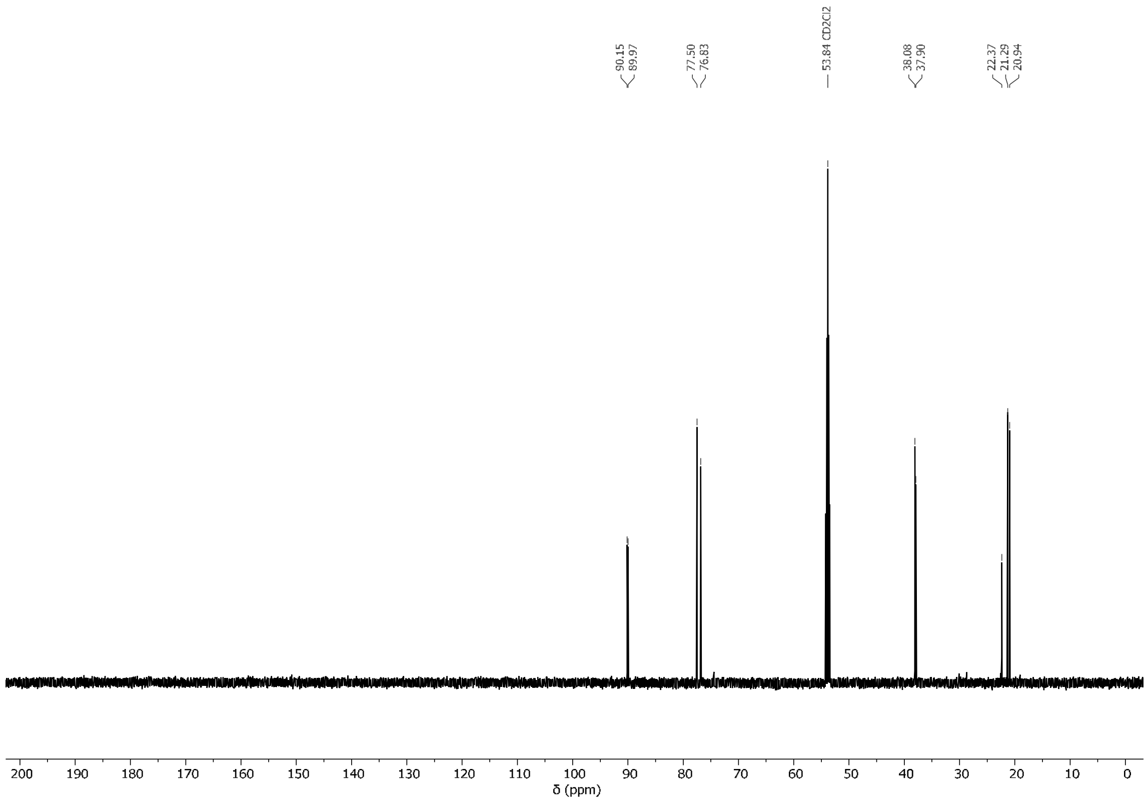
90.15
89.97
77.50
76.83
53.84 CD2Cl2
38.08
37.90
22.37
21.29
20.94
200 190 180 170 160 150 140 130 120 110 100 90 80 70 60 50 40 30 20 10 0
δ (ppm)

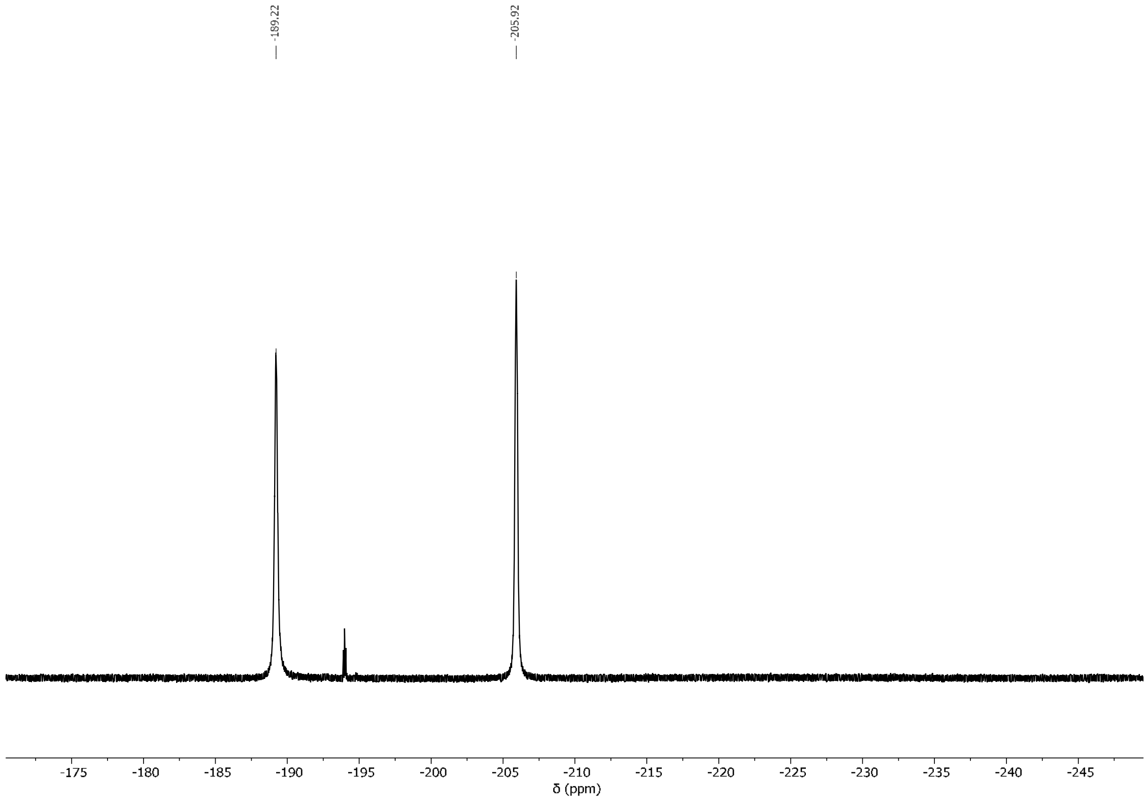
-189.22
-205.92
-175 -180 -185 -190 -195 -200 -205 -210 -215 -220 -225 -230 -235 -240 -245
δ (ppm)

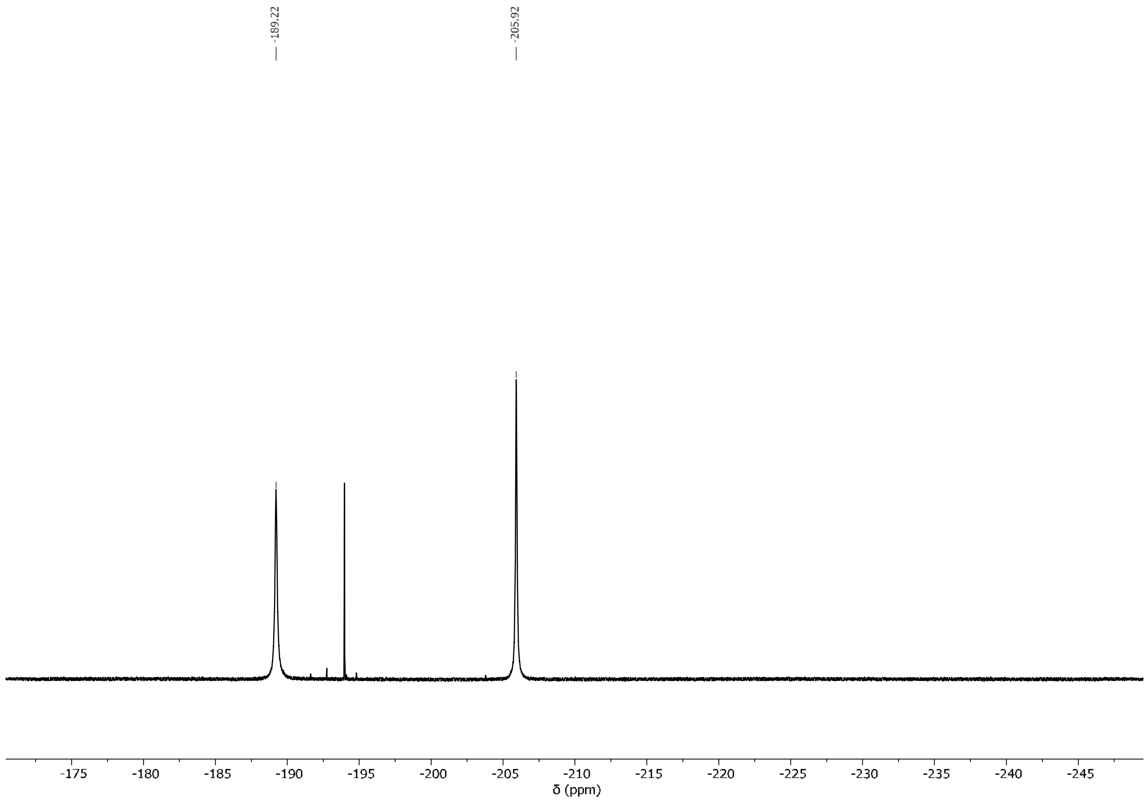
-189.22
-205.92
-175 -180 -185 -190 -195 -200 -205 -210 -215 -220 -225 -230 -235 -240 -245
δ (ppm)

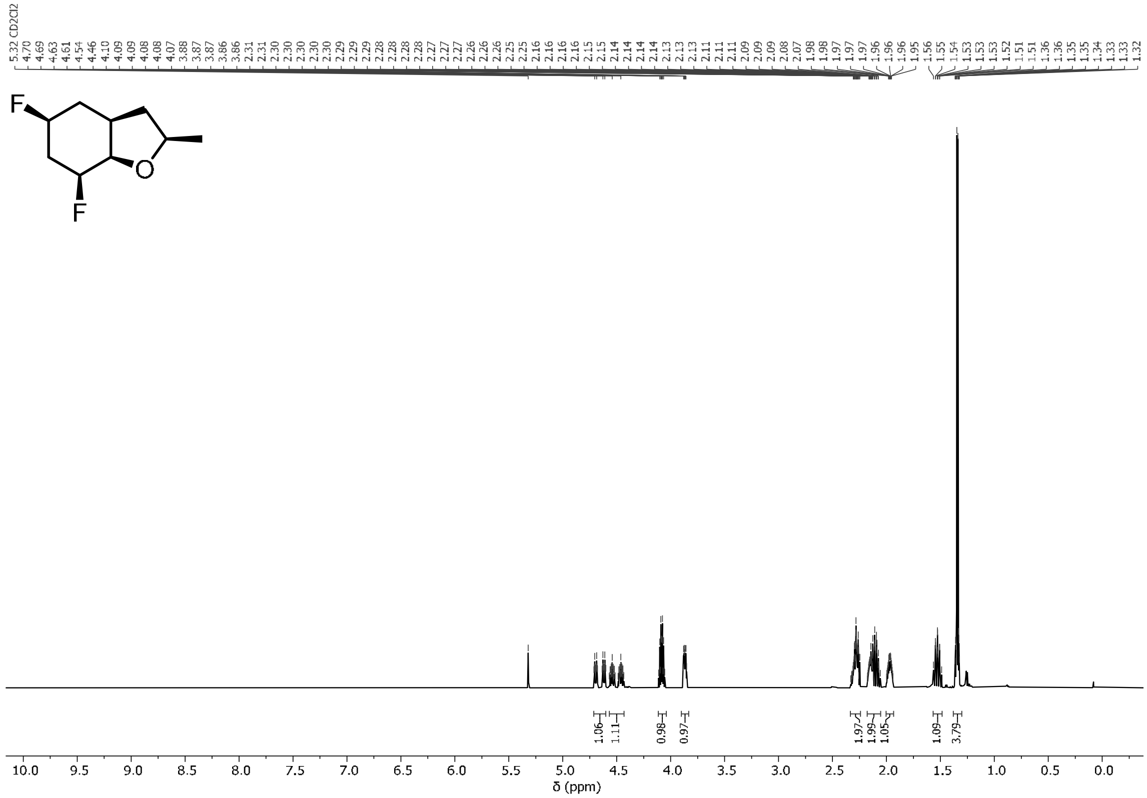
5.32 CD2Cl2
4.70 4.69 4.63 4.61 4.54 4.46 4.10 4.09 4.09 4.08 4.08 4.07 3.88 3.87 3.87 3.86 2.31 2.31 2.31 2.30 2.30 2.30 2.30 2.29 2.29 2.29 2.28 2.28 2.28 2.28 2.27 2.27 2.27 2.26 2.26 2.26 2.25 2.25 2.16 2.16 2.16 2.16 2.15 2.15 2.14 2.14 2.14 2.14 2.13 2.13 2.13 2.13 2.11 2.11 2.11 2.09 2.09 2.09 2.08 2.07 1.98 1.98 1.97 1.97 1.97 1.96 1.96 1.96 1.95 1.56 1.55 1.54 1.53 1.53 1.53 1.52 1.51 1.51 1.36 1.36 1.35 1.35 1.34 1.33 1.33 1.32
F
O
F
1.06 1.11 0.98 0.97 1.97 1.99 1.05 1.09 3.79
10.0 9.5 9.0 8.5 8.0 7.5 7.0 6.5 6.0 5.5 5.0 4.5 4.0 3.5 3.0 2.5 2.0 1.5 1.0 0.5 0.0
δ (ppm)

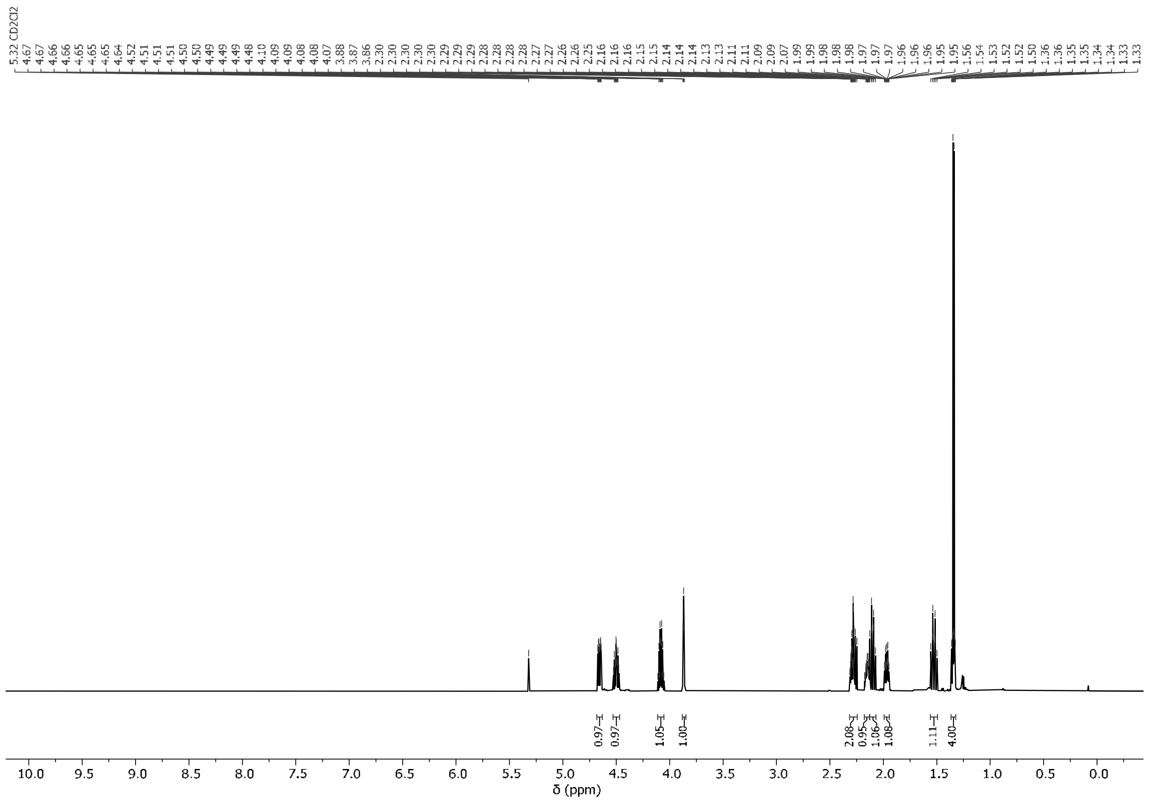
5.32 CD2Cl2
4.67 4.67 4.66 4.66 4.65 4.65 4.64 4.52 4.51 4.51 4.51 4.50 4.50 4.49 4.49 4.49 4.48 4.10 4.09 4.09 4.08 4.08 4.07 3.88 3.87 3.86 2.30 2.30 2.30 2.30 2.30 2.29 2.29 2.29 2.28 2.28 2.28 2.27 2.27 2.26 2.26 2.25 2.16 2.16 2.16 2.15 2.14 2.14 2.14 2.13 2.13 2.11 2.11 2.09 2.07 1.99 1.99 1.98 1.98 1.98 1.97 1.97 1.97 1.96 1.96 1.96 1.95 1.95 1.56 1.54 1.53 1.52 1.52 1.50 1.36 1.36 1.35 1.35 1.34 1.33 1.33
0.97 0.97 1.05 1.00 2.08 0.95 1.06 1.08 1.11 4.00
10.0 9.5 9.0 8.5 8.0 7.5 7.0 6.5 6.0 5.5 5.0 4.5 4.0 3.5 3.0 2.5 2.0 1.5 1.0 0.5 0.0
δ (ppm)

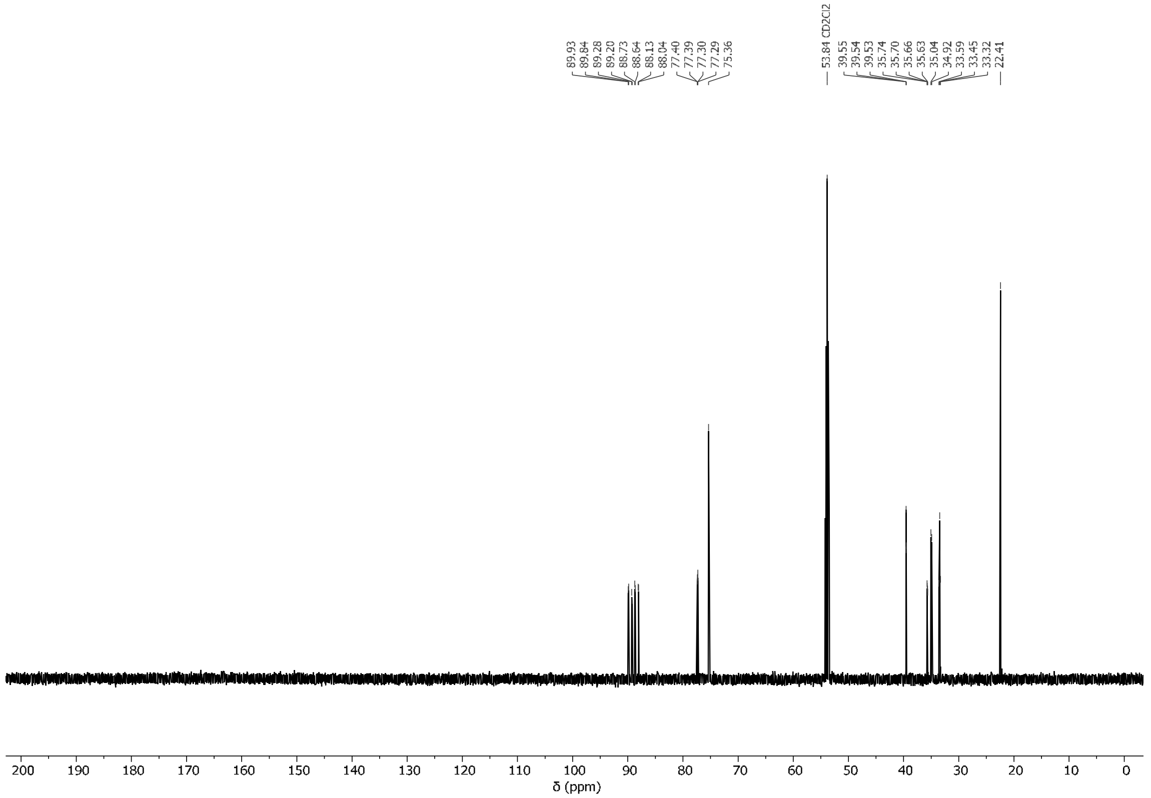
89.93 89.84 89.28 89.20 88.73 88.64 88.13 88.04 77.40 77.39 77.30 77.29 75.36
53.84 CD2Cl2
39.55 39.54 39.53 35.74 35.70 35.66 35.63 35.04 34.92 33.59 33.45 33.32 22.41
200 190 180 170 160 150 140 130 120 110 100 90 80 70 60 50 40 30 20 10 0
δ (ppm)

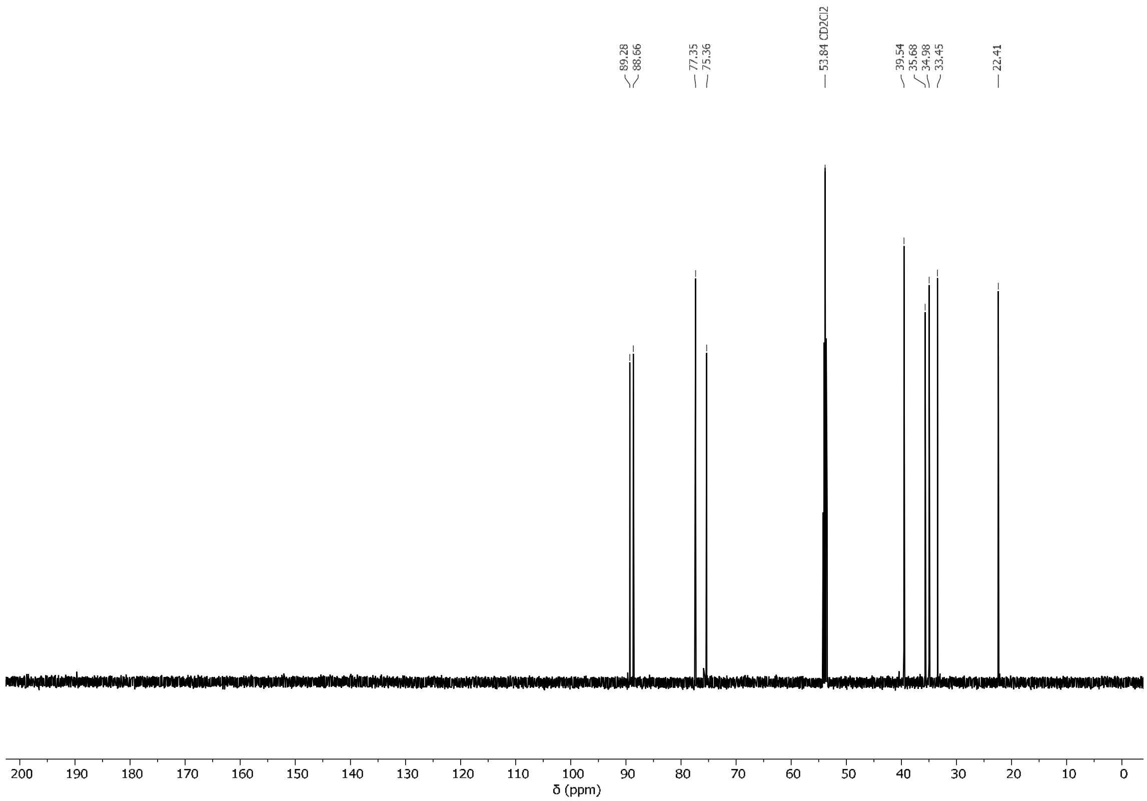
89.28
88.66
77.35
75.36
53.84 CD2Cl2
39.54
35.68
34.98
33.45
22.41
δ (ppm)

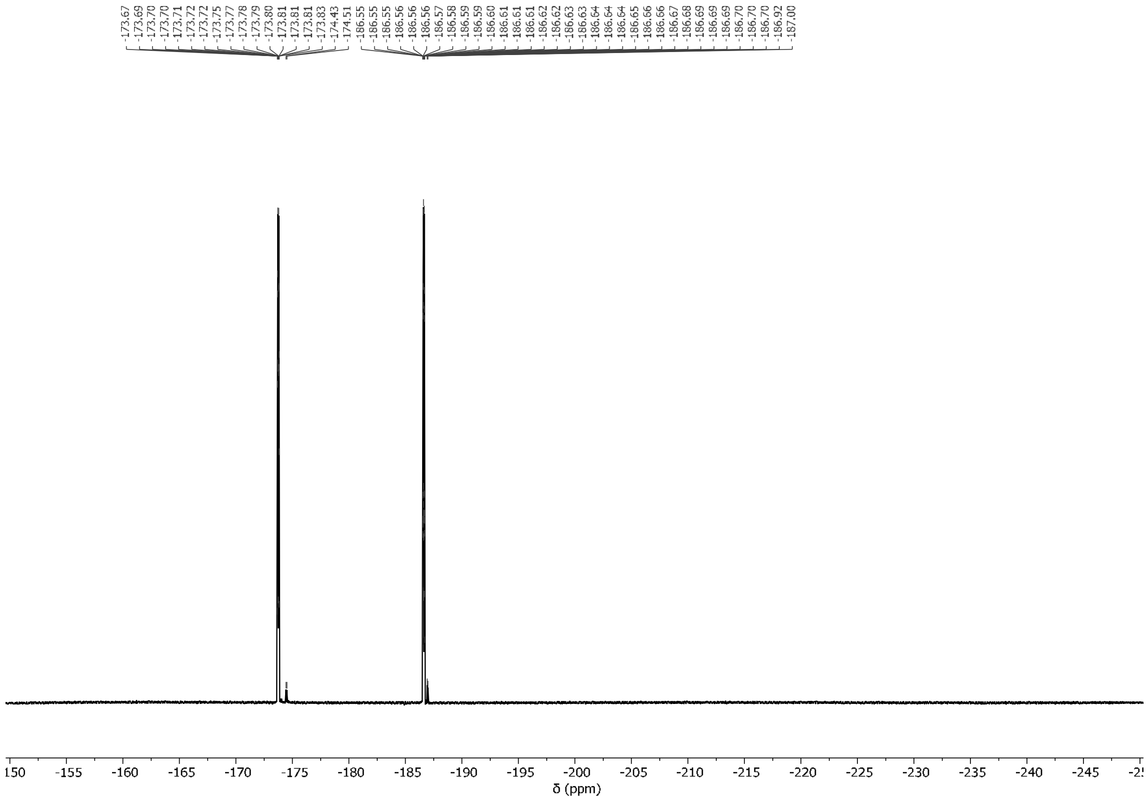
-173.67
-173.69
-173.70
-173.71
-173.72
-173.75
-173.77
-173.78
-173.79
-173.80
-173.81
-173.81
-173.81
-173.83
-174.43
-174.51
-186.55
-186.55
-186.56
-186.56
-186.56
-186.57
-186.58
-186.59
-186.59
-186.60
-186.61
-186.61
-186.62
-186.63
-186.63
-186.64
-186.64
-186.65
-186.66
-186.67
-186.68
-186.69
-186.69
-186.70
-186.70
-186.70
-186.92
-187.00
δ (ppm)

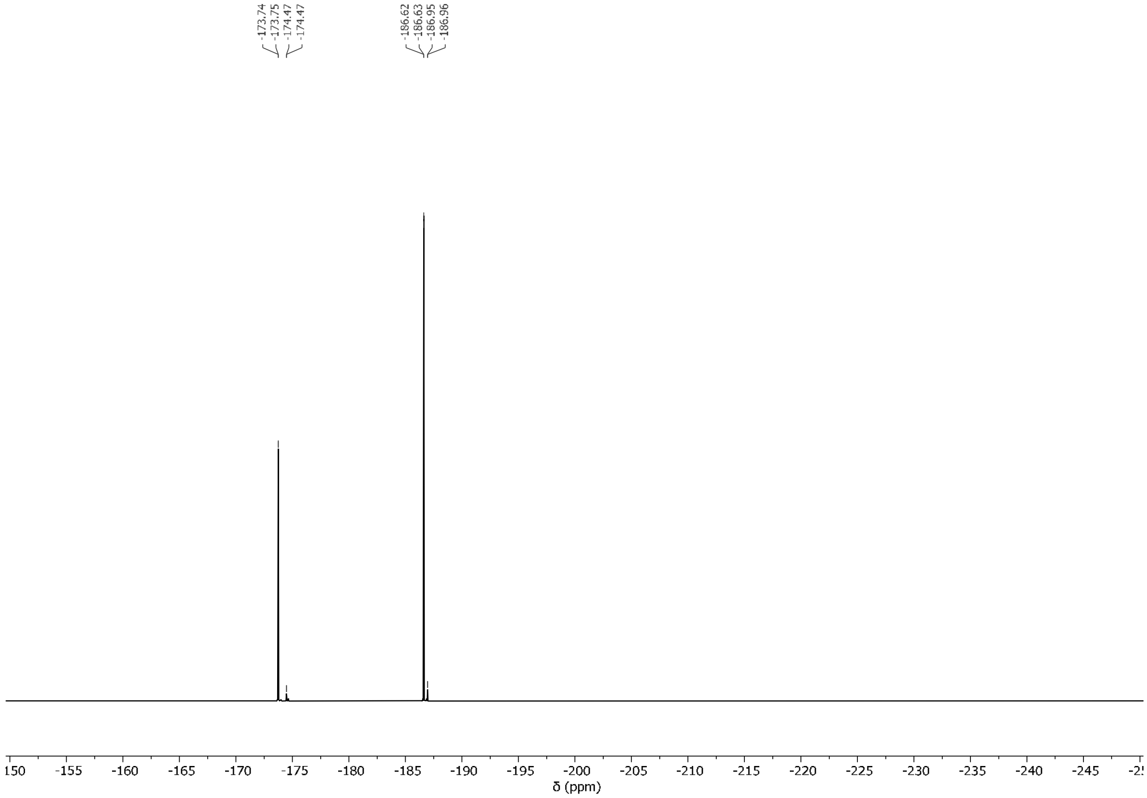

-173.74
-173.75
-174.47
-174.47
-186.62
-186.63
-186.95
-186.96
150 -155 -160 -165 -170 -175 -180 -185 -190 -195 -200 -205 -210 -215 -220 -225 -230 -235 -240 -245
δ (ppm)

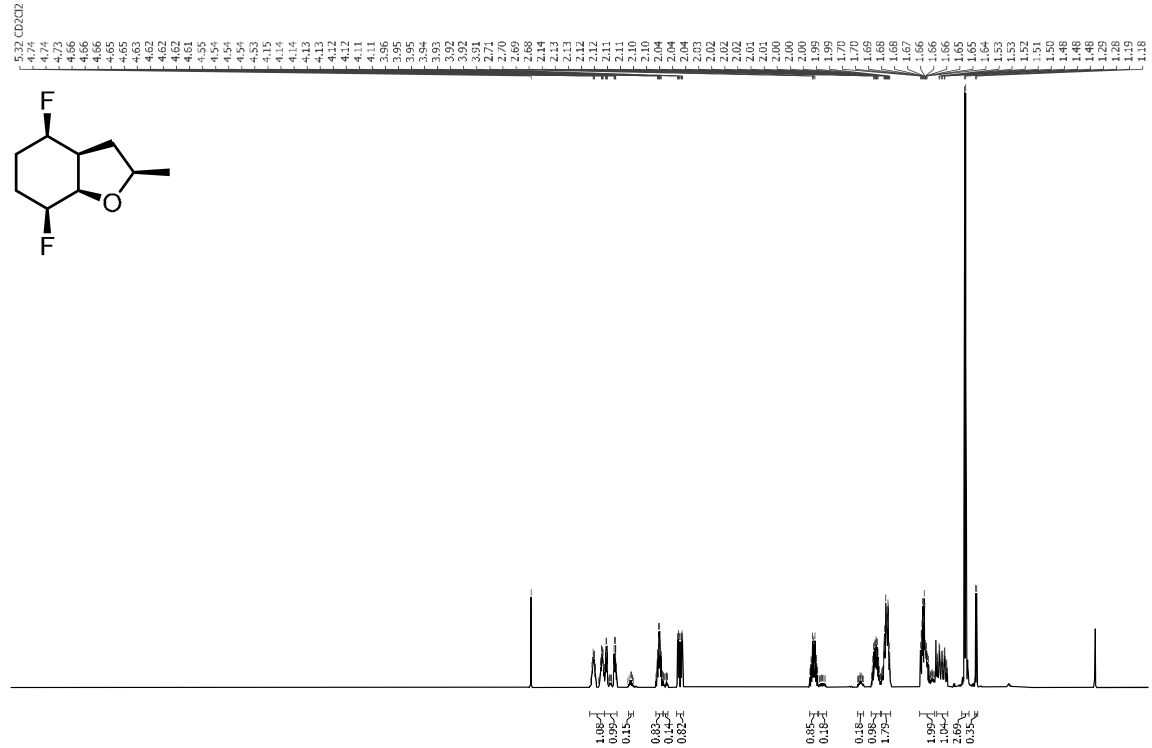

5.32 CD2Cl2
4.74 4.74 4.73 4.66 4.66 4.66 4.65 4.65 4.63 4.62 4.62 4.62 4.61 4.55 4.54 4.54 4.54 4.53 4.15 4.14 4.14 4.13 4.13 4.12 4.12 4.11 4.11 3.96 3.95 3.95 3.94 3.93 3.92 3.92 3.91 2.71 2.70 2.69 2.68 2.14 2.13 2.13 2.12 2.12 2.11 2.11 2.11 2.10 2.10 2.04 2.04 2.04 2.04 2.03 2.02 2.02 2.02 2.01 2.01 2.00 2.00 1.99 1.99 1.70 1.69 1.68 1.68 1.67 1.66 1.66 1.66 1.65 1.65 1.64 1.53 1.53 1.52 1.51 1.50 1.48 1.48 1.48 1.29 1.28 1.19 1.18
F
F
O
1.08 0.99 0.15 0.83 0.14 0.82 0.85 0.18 0.18 0.98 1.79 1.99 1.04 2.69 0.35
10.0 9.5 9.0 8.5 8.0 7.5 7.0 6.5 6.0 5.5 5.0 4.5 4.0 3.5 3.0 2.5 2.0 1.5 1.0 0.5 0.0
δ (ppm)

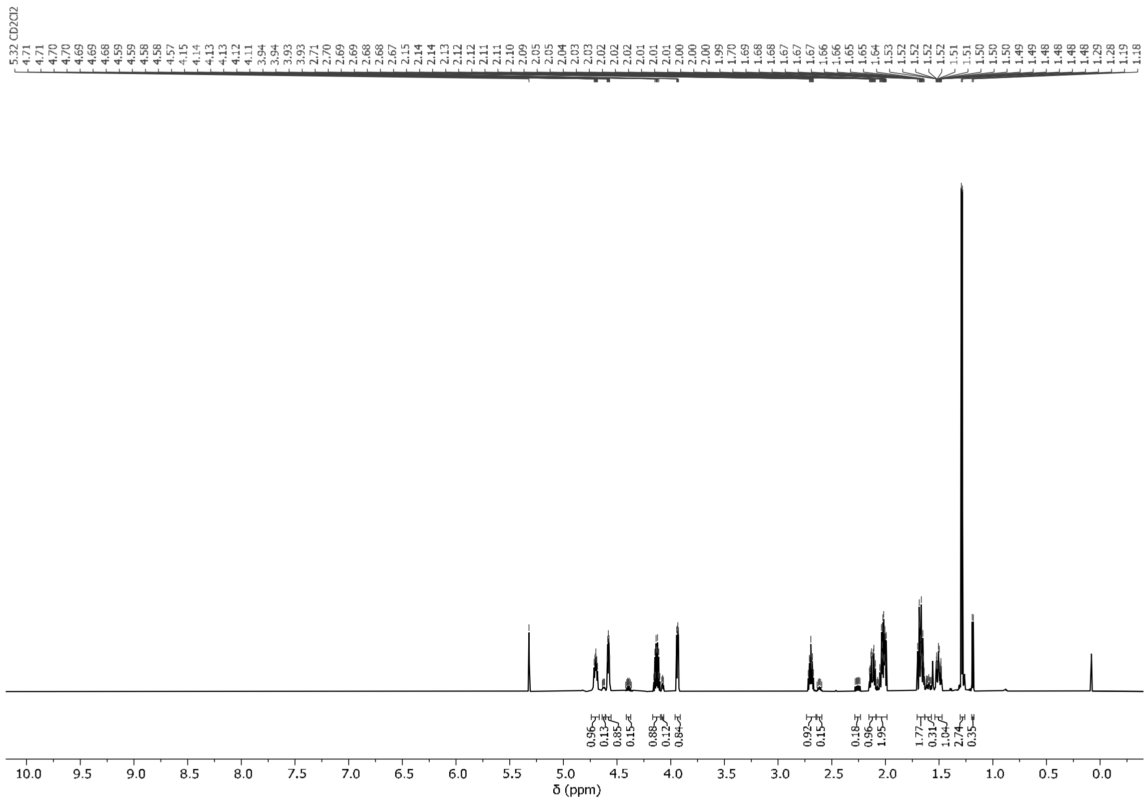
5.32 CD2Cl2
4.71 4.71 4.70 4.70 4.69 4.69 4.68 4.59 4.59 4.58 4.58 4.57 4.15 4.14 4.13 4.13 4.12 4.11 3.94 3.94 3.93 3.93 2.71 2.70 2.69 2.69 2.68 2.68 2.67 2.15 2.14 2.14 2.13 2.12 2.12 2.11 2.11 2.10 2.09 2.05 2.05 2.04 2.03 2.03 2.02 2.02 2.02 2.01 2.01 2.01 2.00 2.00 1.99 1.70 1.69 1.68 1.68 1.67 1.67 1.66 1.66 1.65 1.65 1.64 1.53 1.52 1.52 1.52 1.52 1.51 1.51 1.50 1.50 1.50 1.49 1.49 1.48 1.48 1.48 1.48 1.29 1.28 1.19 1.18
0.96 0.13 0.85 0.15 0.88 0.12 0.84 0.92 0.15 0.18 0.96 1.95 1.77 0.31 1.04 2.74 0.35
10.0 9.5 9.0 8.5 8.0 7.5 7.0 6.5 6.0 5.5 5.0 4.5 4.0 3.5 3.0 2.5 2.0 1.5 1.0 0.5 0.0
δ (ppm)

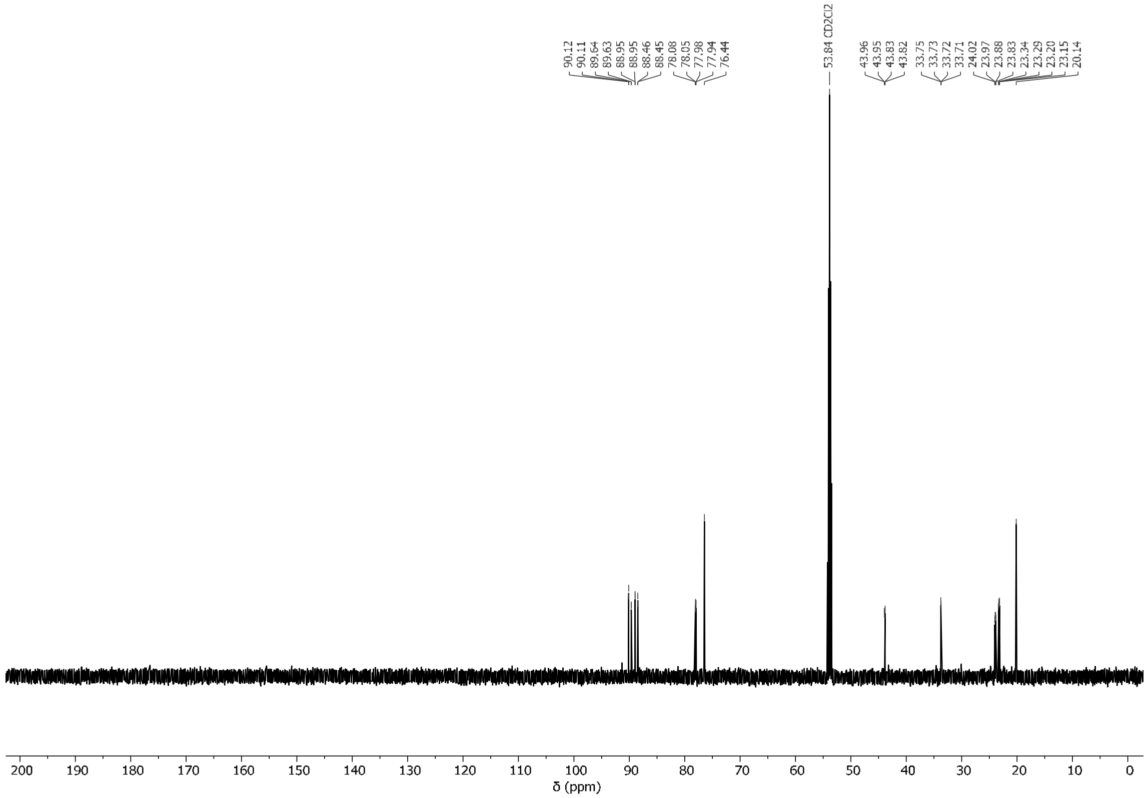
90.12 90.11 89.64 89.63 88.95 88.95 88.46 88.45 78.08 78.05 77.98 77.94 76.44
53.84 CD2Cl2
43.96 43.95 43.83 43.82 33.75 33.73 33.72 33.71 24.02 23.97 23.88 23.83 23.34 23.29 23.20 23.15 20.14
200 190 180 170 160 150 140 130 120 110 100 90 80 70 60 50 40 30 20 10 0
δ (ppm)

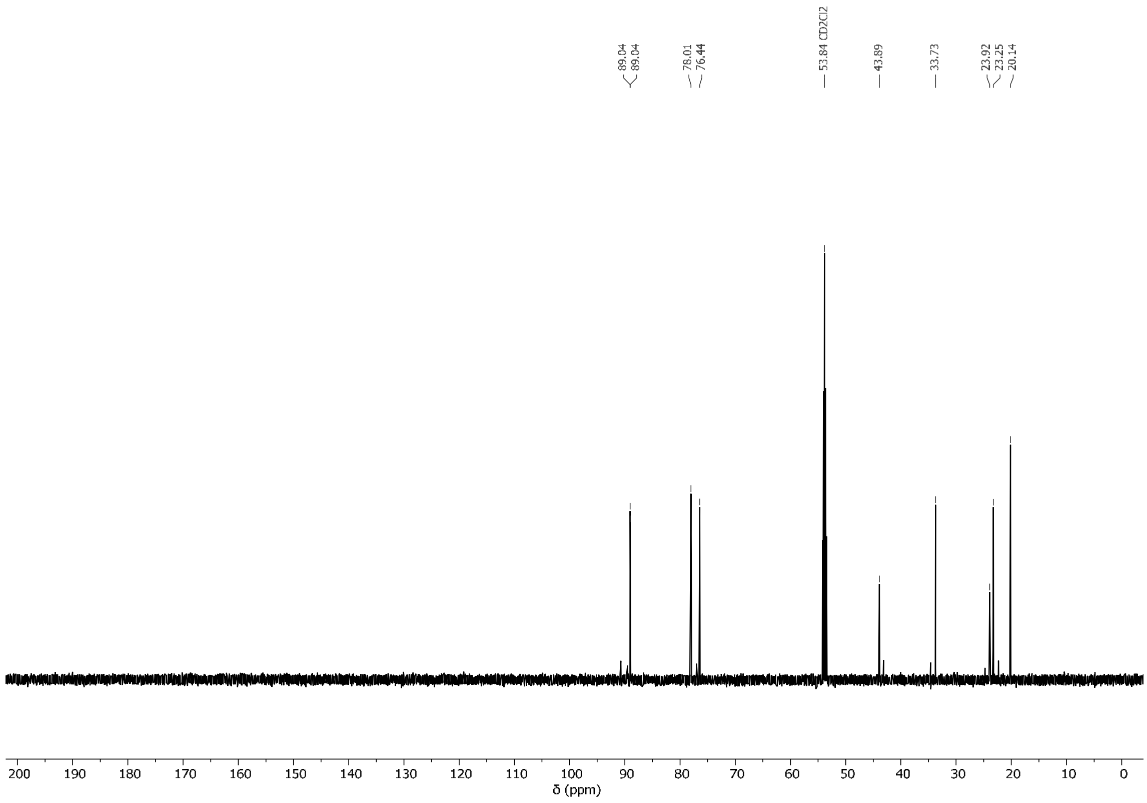
89.04
89.04
78.01
76.44
53.84 CD2Cl2
43.89
33.73
23.92
23.25
20.14
200 190 180 170 160 150 140 130 120 110 100 90 80 70 60 50 40 30 20 10 0
δ (ppm)

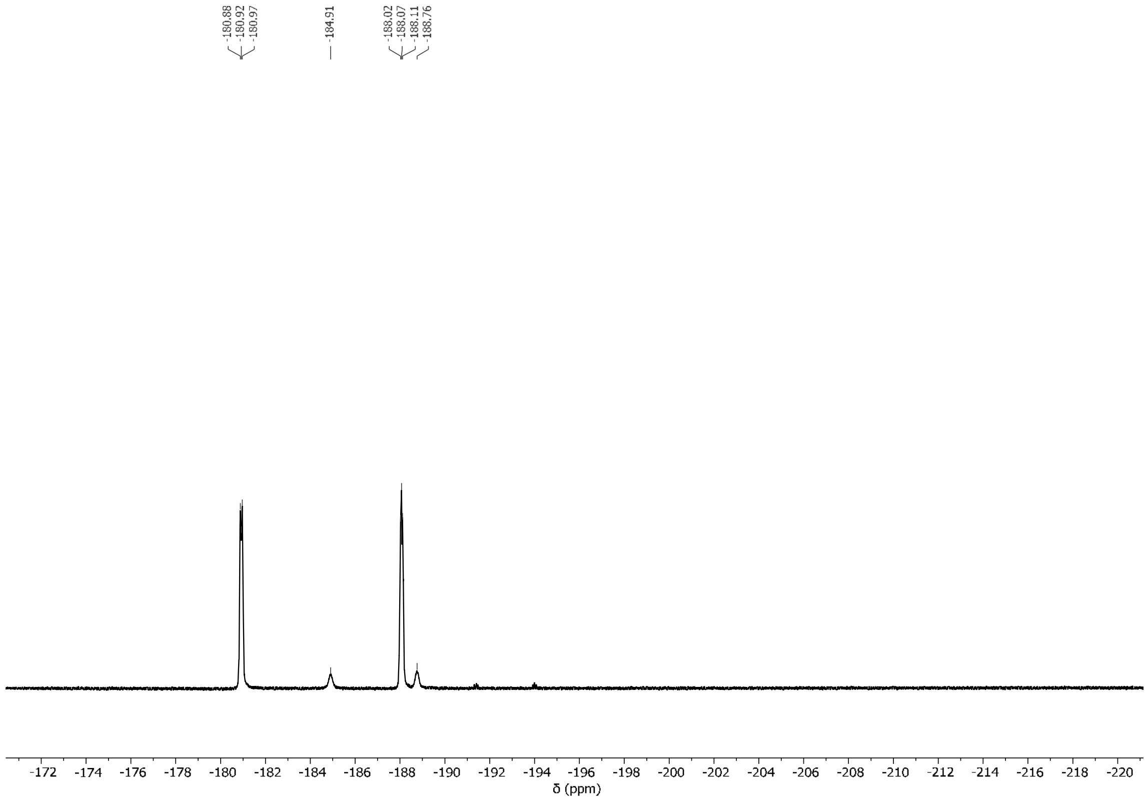
-180.88
-180.92
-180.97
-184.91
-188.02
-188.07
-188.11
-188.76
-172 -174 -176 -178 -180 -182 -184 -186 -188 -190 -192 -194 -196 -198 -200 -202 -204 -206 -208 -210 -212 -214 -216 -218 -220
δ (ppm)

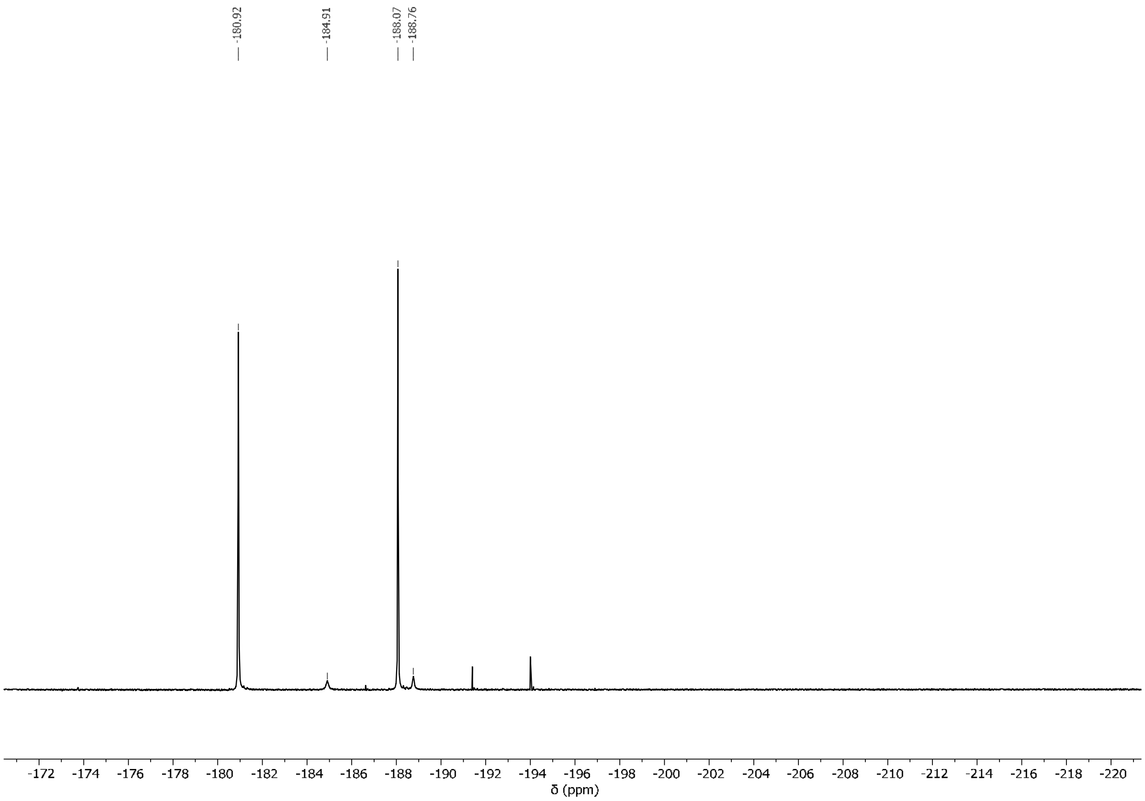

-180.92
-184.91
-188.07
-188.76
-172 -174 -176 -178 -180 -182 -184 -186 -188 -190 -192 -194 -196 -198 -200 -202 -204 -206 -208 -210 -212 -214 -216 -218 -220
δ (ppm)

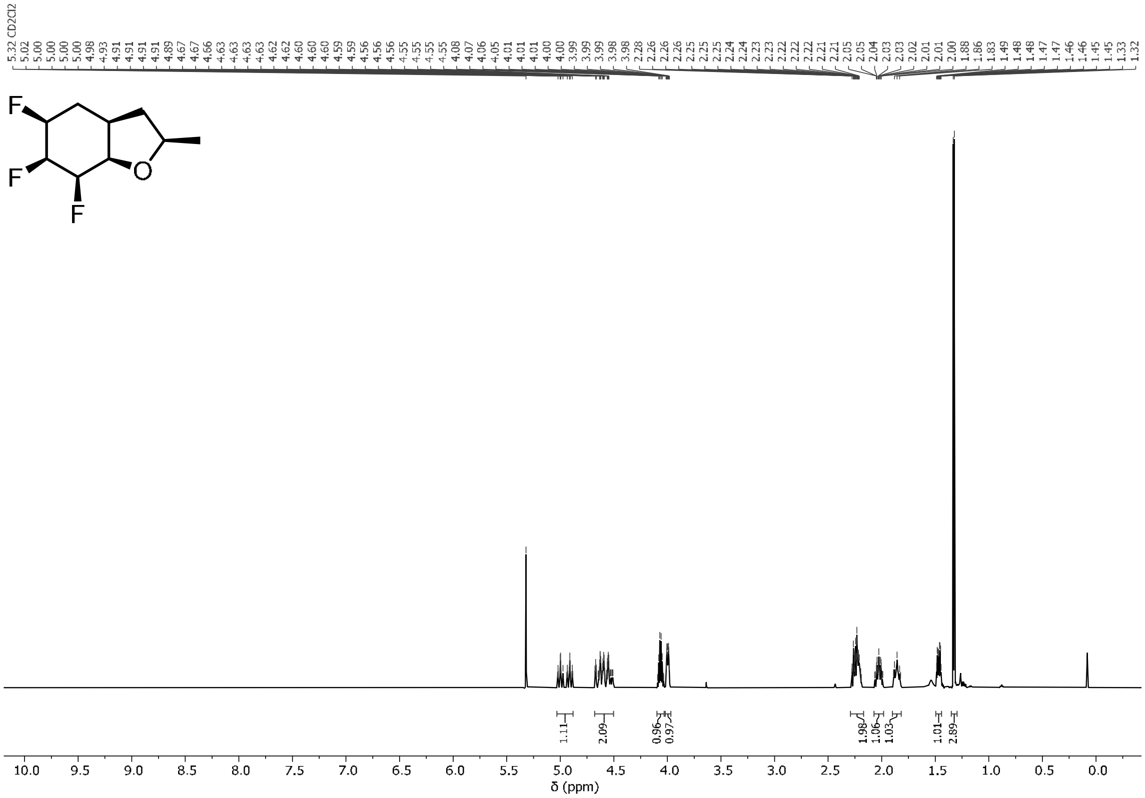

5.32 CD2Cl2
5.02 5.00 5.00 5.00 5.00 4.98 4.93 4.91 4.91 4.91 4.91 4.89 4.67 4.66 4.63 4.63 4.63 4.62 4.60 4.60 4.59 4.59 4.56 4.56 4.56 4.55 4.55 4.55 4.08 4.07 4.06 4.05 4.01 4.01 4.01 4.00 3.99 3.99 3.99 3.98 3.98 2.28 2.26 2.26 2.26 2.25 2.25 2.25 2.24 2.24 2.23 2.23 2.22 2.22 2.22 2.21 2.21 2.05 2.05 2.04 2.03 2.03 2.02 2.01 2.01 2.00 1.88 1.86 1.83 1.49 1.48 1.48 1.47 1.47 1.46 1.46 1.45 1.33 1.32
F
F
F
O
1.11
2.09
0.96
0.97
1.98
1.06
1.03
1.01
2.89
10.0 9.5 9.0 8.5 8.0 7.5 7.0 6.5 6.0 5.5 5.0 4.5 4.0 3.5 3.0 2.5 2.0 1.5 1.0 0.5 0.0
δ (ppm)

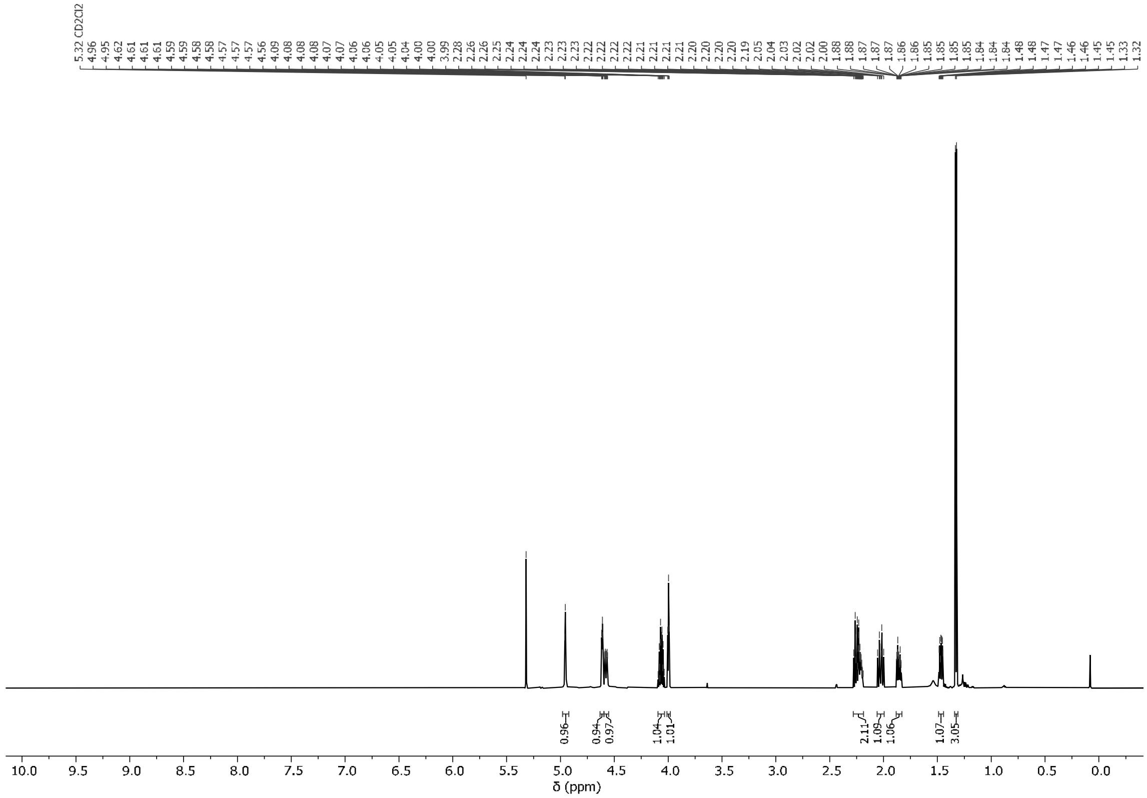
5.32 CD2Cl2
4.96
4.95
4.62
4.61
4.61
4.61
4.59
4.59
4.58
4.58
4.57
4.57
4.56
4.09
4.08
4.08
4.08
4.07
4.07
4.06
4.06
4.05
4.05
4.04
4.00
4.00
3.99
2.28
2.26
2.26
2.25
2.24
2.24
2.24
2.23
2.23
2.23
2.22
2.22
2.22
2.22
2.21
2.21
2.21
2.21
2.20
2.20
2.20
2.20
2.19
2.05
2.04
2.03
2.02
2.02
2.00
1.88
1.88
1.87
1.87
1.87
1.86
1.86
1.85
1.85
1.85
1.85
1.84
1.84
1.84
1.48
1.48
1.47
1.46
1.46
1.45
1.33
1.32
0.96
0.94
0.97
1.04
1.01
2.11
1.09
1.06
1.07
3.05
10.0 9.5 9.0 8.5 8.0 7.5 7.0 6.5 6.0 5.5 5.0 4.5 4.0 3.5 3.0 2.5 2.0 1.5 1.0 0.5 0.0
δ (ppm)

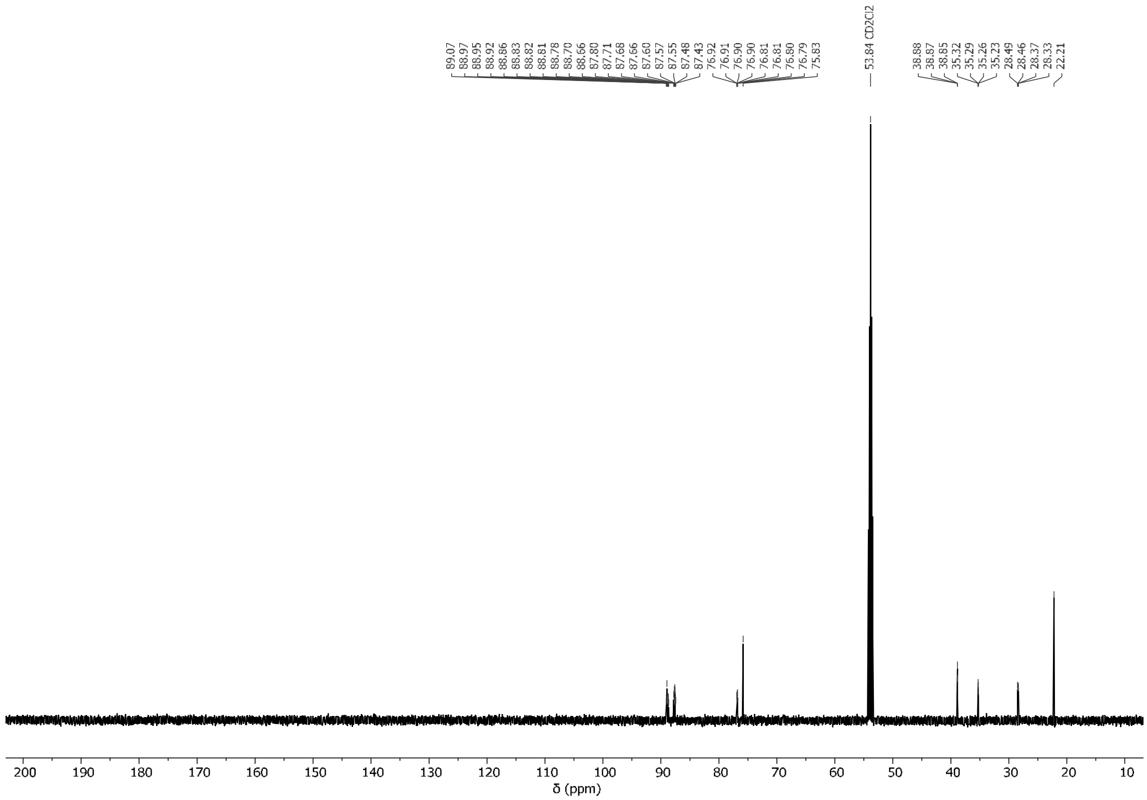
89.07
88.97
88.95
88.92
88.86
88.83
88.82
88.81
88.78
88.70
88.66
87.80
87.71
87.68
87.66
87.60
87.57
87.55
87.48
87.43
76.92
76.91
76.90
76.90
76.81
76.81
76.81
76.80
76.79
75.83
53.84 CD2Cl2
38.88
38.87
38.85
35.32
35.29
35.26
35.23
28.49
28.46
28.37
28.33
22.21
200 190 180 170 160 150 140 130 120 110 100 90 80 70 60 50 40 30 20 10
δ (ppm)

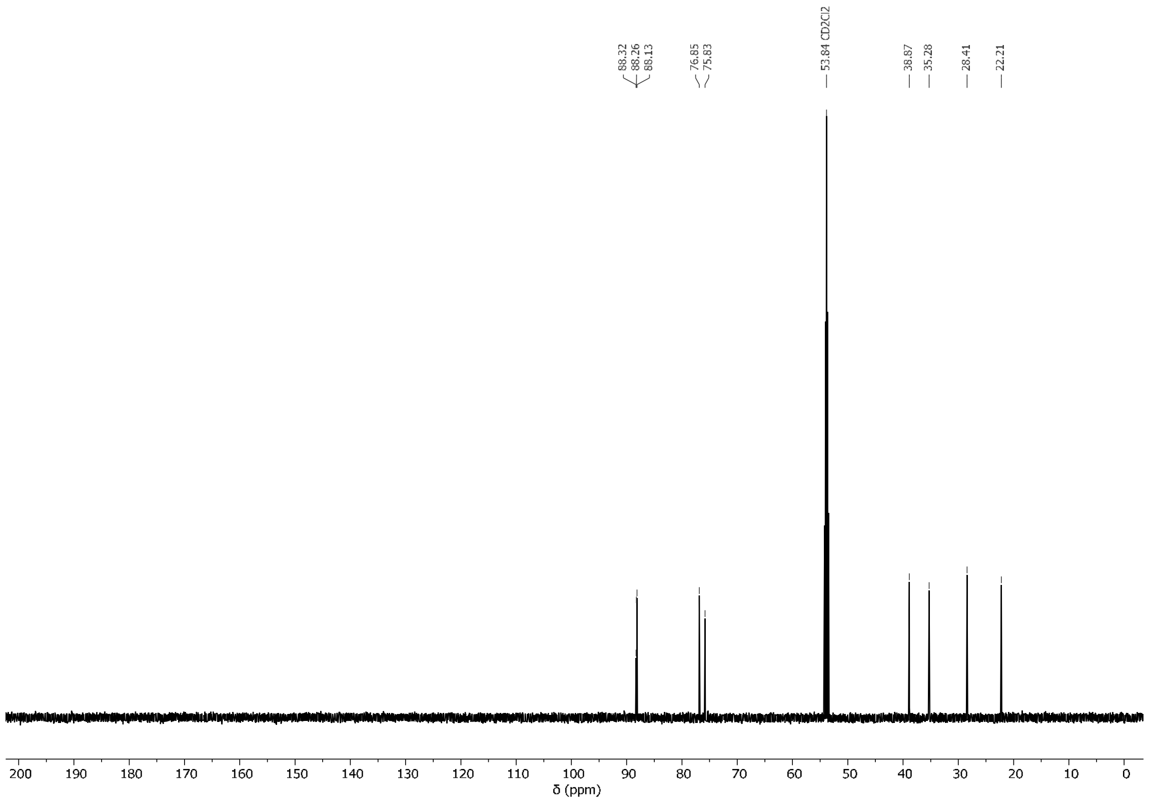
88.32
88.26
88.13
76.85
75.83
53.84 CD2Cl2
38.87
35.28
28.41
22.21
200 190 180 170 160 150 140 130 120 110 100 90 80 70 60 50 40 30 20 10 0
δ (ppm)

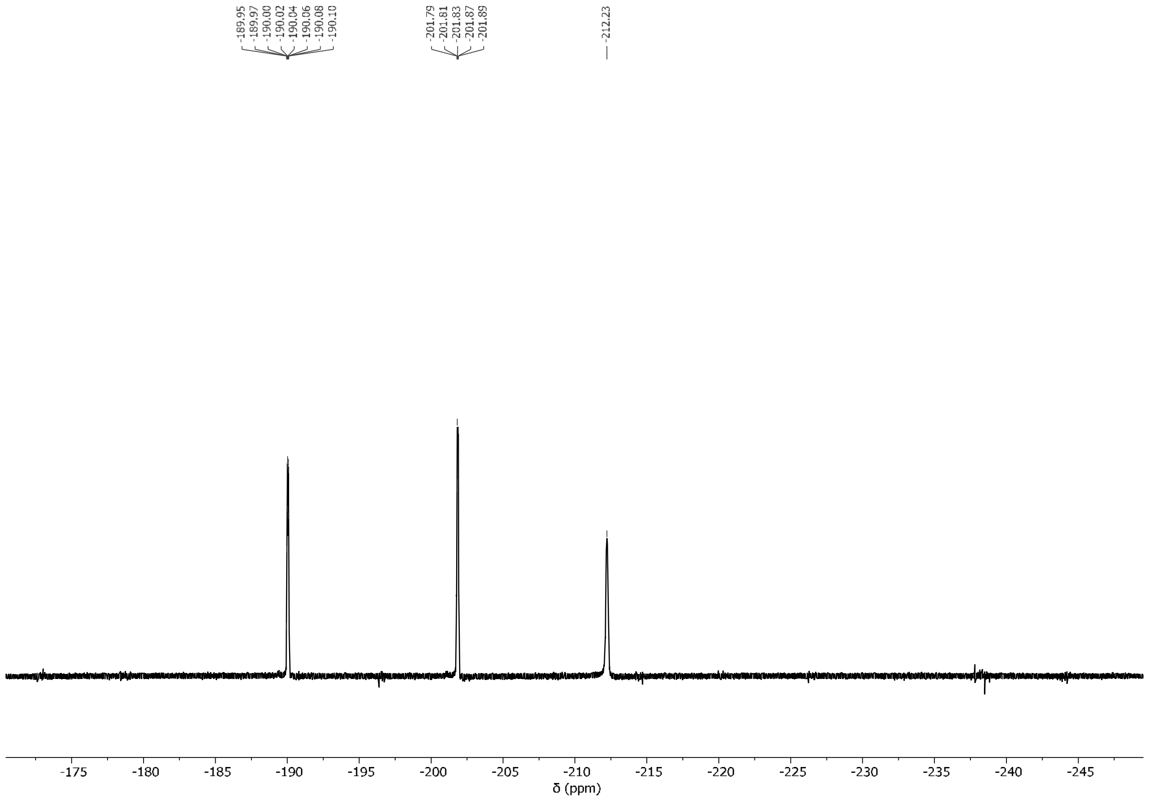
-189.95
-189.97
-190.00
-190.02
-190.04
-190.06
-190.08
-190.10
-201.79
-201.81
-201.83
-201.87
-201.89
-212.23
-175 -180 -185 -190 -195 -200 -205 -210 -215 -220 -225 -230 -235 -240 -245
δ (ppm)

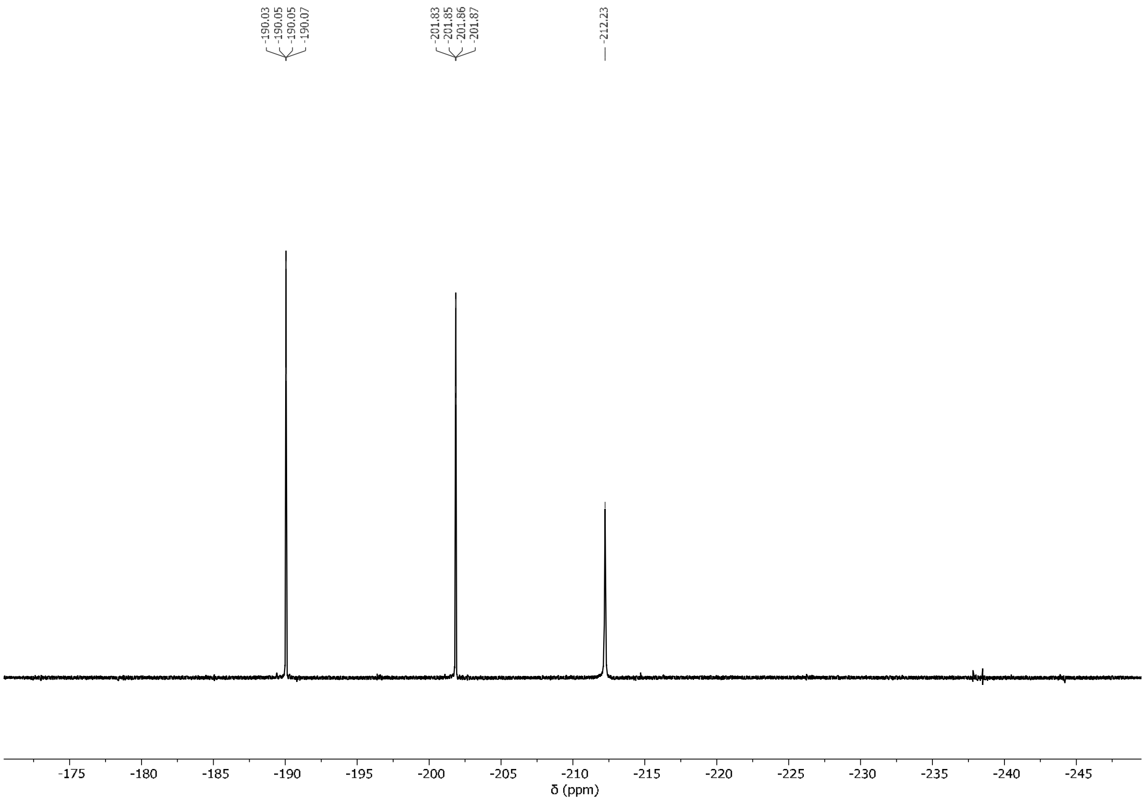
-190.03
-190.05
-190.05
-190.07
-201.83
-201.85
-201.86
-201.87
-212.23
-175 -180 -185 -190 -195 -200 -205 -210 -215 -220 -225 -230 -235 -240 -245
δ (ppm)

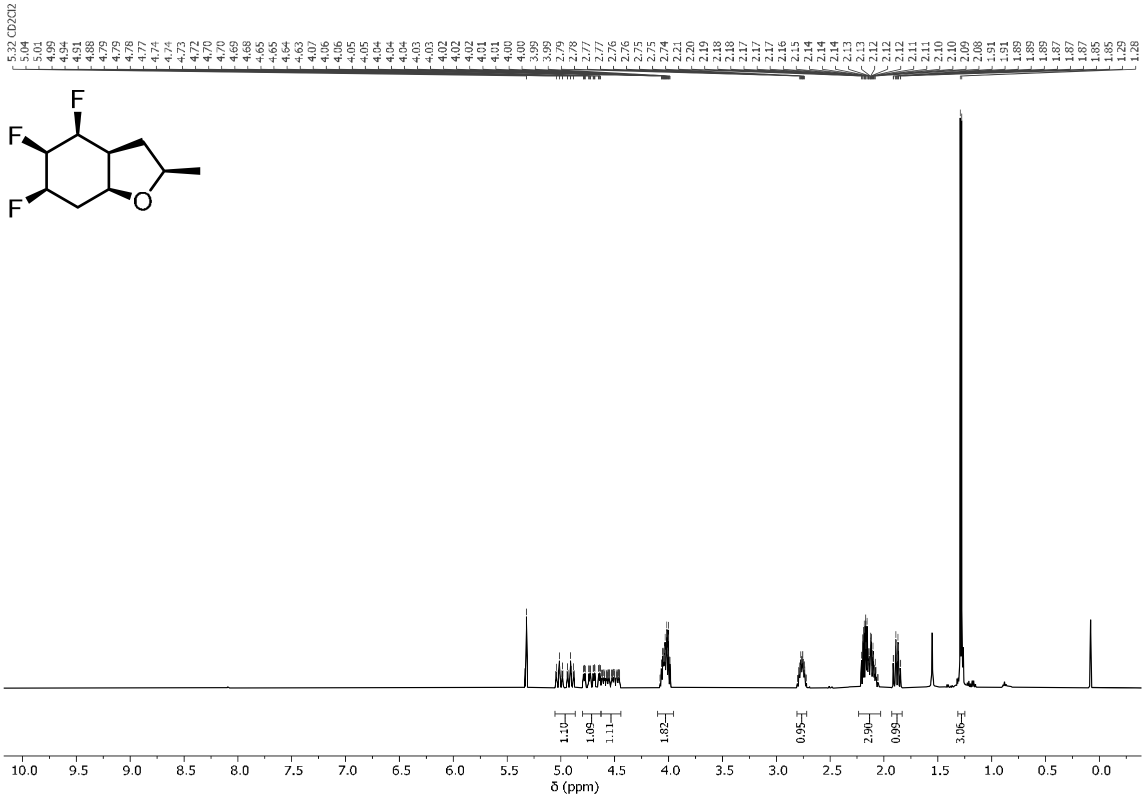
5.32 CD2Cl2
5.04 5.01 4.99 4.94 4.91 4.88 4.79 4.79 4.78 4.77 4.74 4.74 4.73 4.72 4.70 4.70 4.69 4.68 4.65 4.64 4.63 4.07 4.06 4.05 4.05 4.04 4.04 4.04 4.03 4.03 4.02 4.02 4.02 4.01 4.01 4.00 4.00 3.99 2.79 2.78 2.77 2.77 2.76 2.76 2.75 2.75 2.74 2.21 2.20 2.19 2.18 2.18 2.17 2.17 2.17 2.16 2.15 2.14 2.14 2.14 2.13 2.13 2.12 2.12 2.12 2.11 2.11 2.10 2.10 2.09 2.08 1.91 1.91 1.89 1.89 1.87 1.87 1.87 1.85 1.29 1.28
F
F
F
O
1.10
1.09
1.11
1.82
0.95
2.90
0.99
3.06
10.0 9.5 9.0 8.5 8.0 7.5 7.0 6.5 6.0 5.5 5.0 4.5 4.0 3.5 3.0 2.5 2.0 1.5 1.0 0.5 0.0
δ (ppm)

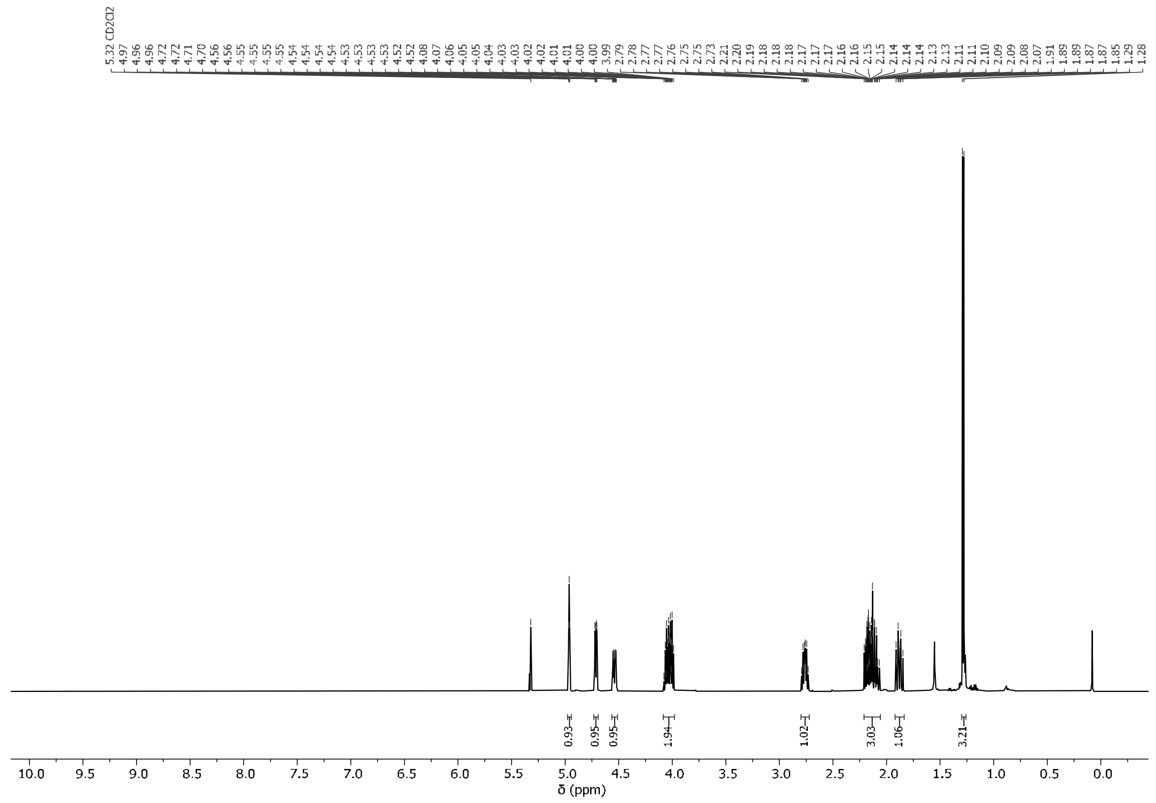
5.32 CD2Cl2
4.97
4.96
4.96
4.72
4.72
4.71
4.70
4.56
4.56
4.55
4.55
4.55
4.55
4.54
4.54
4.54
4.54
4.53
4.53
4.53
4.53
4.52
4.52
4.08
4.07
4.06
4.05
4.05
4.04
4.03
4.03
4.02
4.02
4.01
4.01
4.00
4.00
3.99
2.79
2.78
2.77
2.77
2.76
2.75
2.75
2.73
2.21
2.20
2.19
2.18
2.18
2.18
2.17
2.17
2.17
2.17
2.16
2.16
2.15
2.15
2.14
2.14
2.14
2.13
2.13
2.11
2.11
2.10
2.09
2.09
2.08
2.07
1.91
1.89
1.89
1.87
1.85
1.29
1.28
0.93
0.95
0.95
1.94
1.02
3.03
1.06
3.21
10.0 9.5 9.0 8.5 8.0 7.5 7.0 6.5 6.0 5.5 5.0 4.5 4.0 3.5 3.0 2.5 2.0 1.5 1.0 0.5 0.0
δ (ppm)

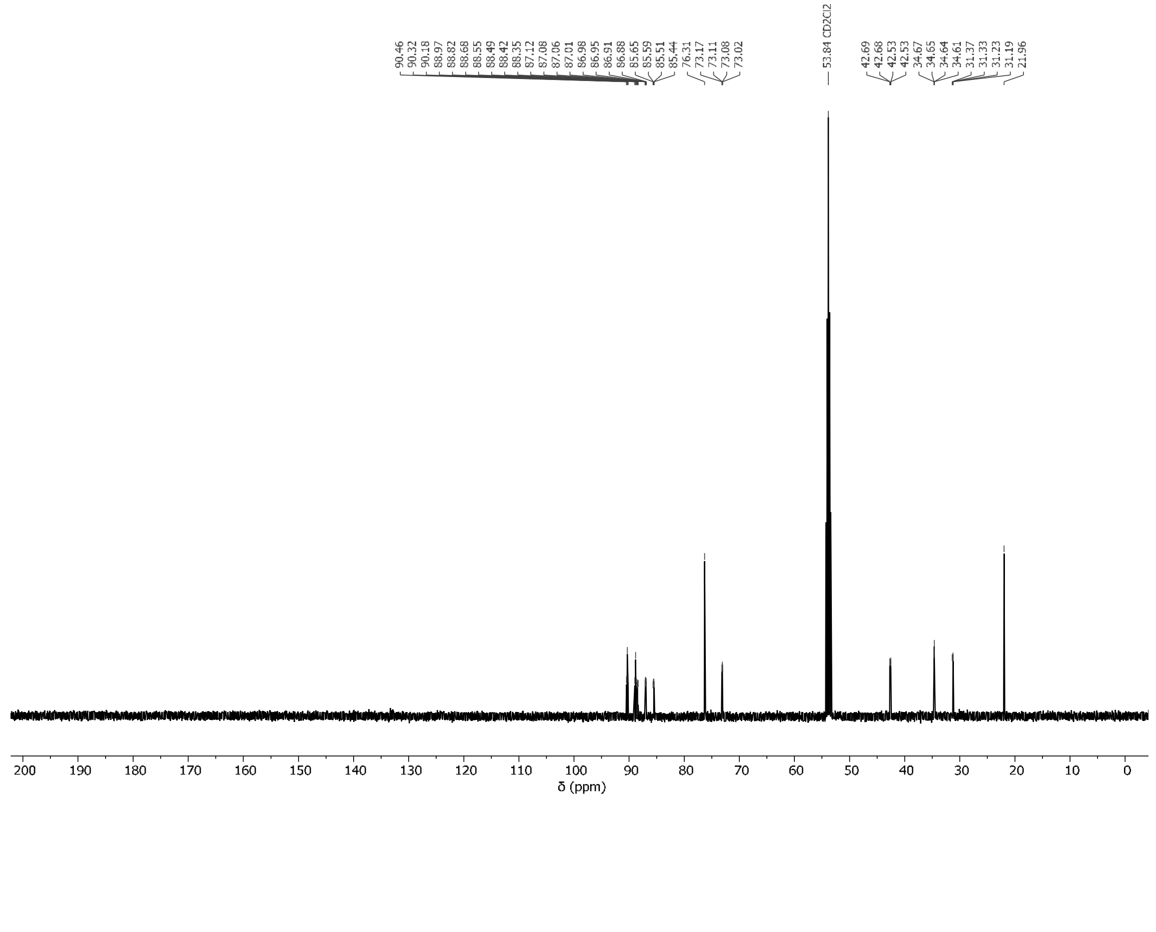
90.46
90.32
90.18
88.97
88.82
88.68
88.55
88.49
88.42
88.35
87.12
87.08
87.06
87.01
86.98
86.95
86.91
86.88
85.65
85.59
85.51
85.44
76.31
73.17
73.11
73.08
73.02
53.84 CD2Cl2
42.69
42.68
42.53
42.53
34.67
34.65
34.64
34.61
31.37
31.33
31.23
31.19
21.96
200 190 180 170 160 150 140 130 120 110 100 90 80 70 60 50 40 30 20 10 0
δ (ppm)

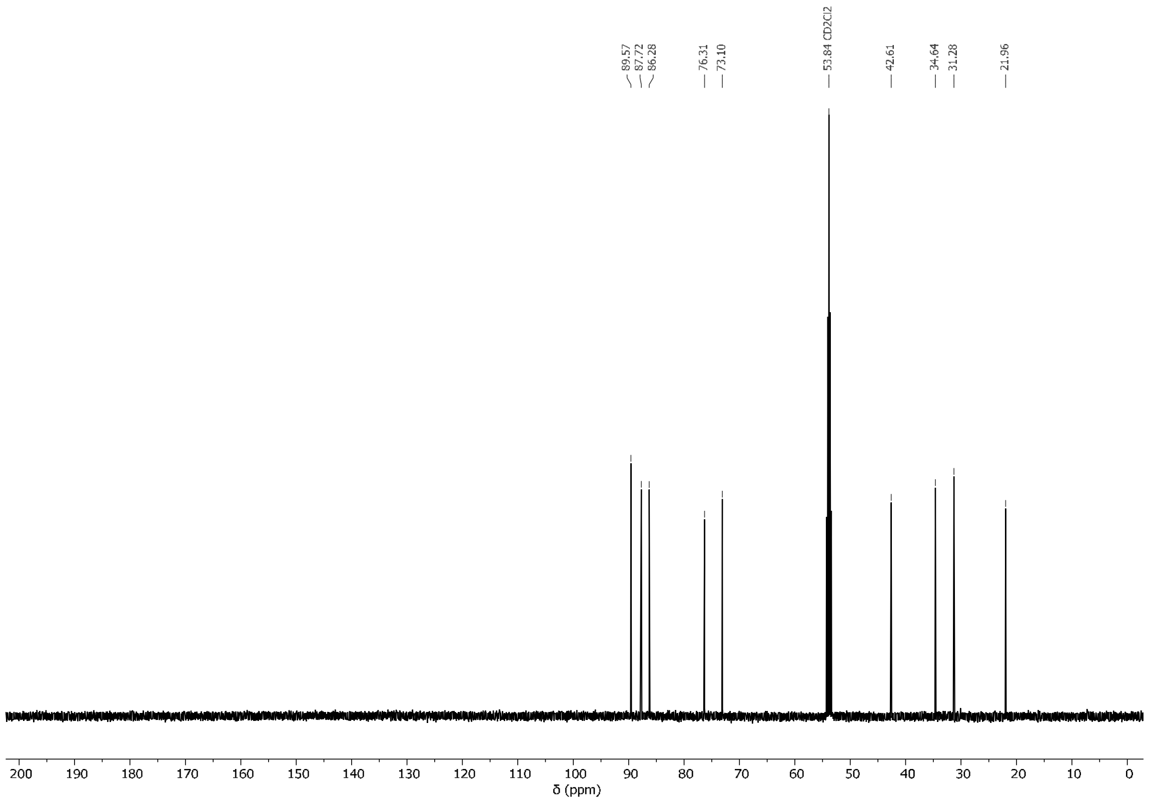
89.57
87.72
86.28
76.31
73.10
53.84 CD2Cl2
42.61
34.64
31.28
21.96
δ (ppm)

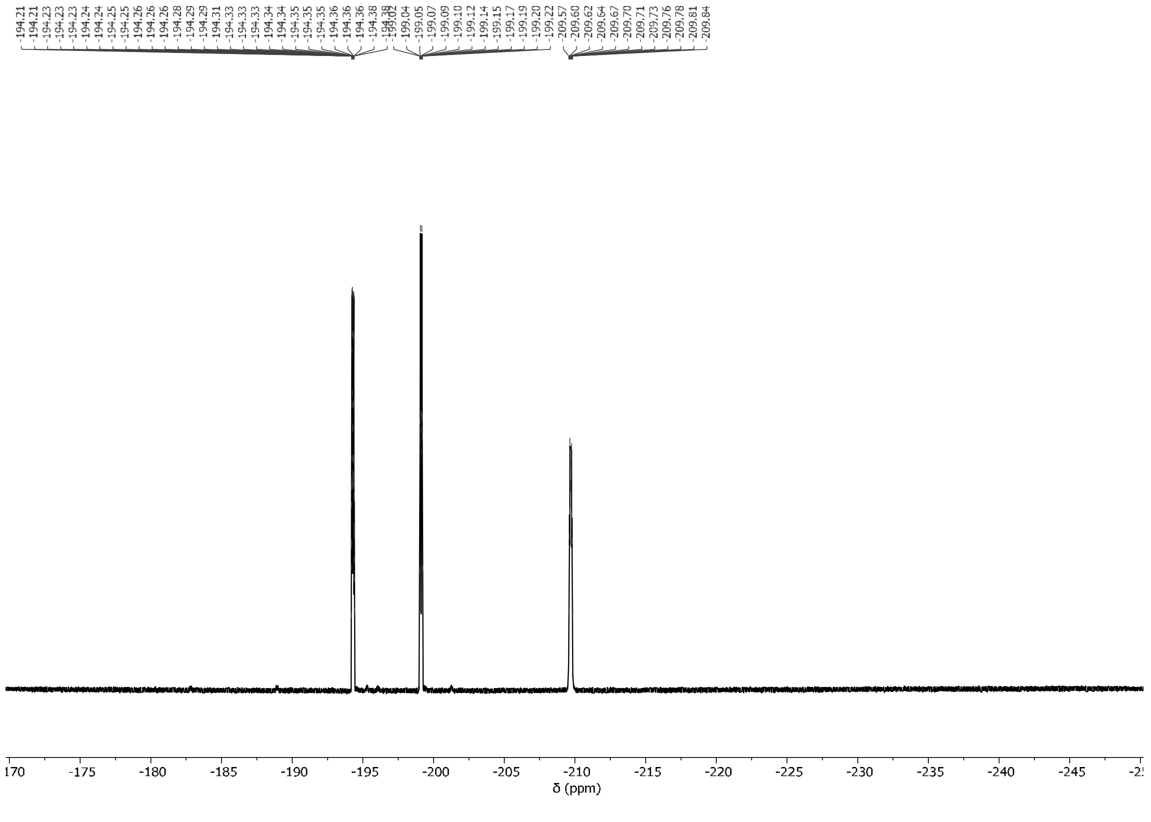
δ (ppm)

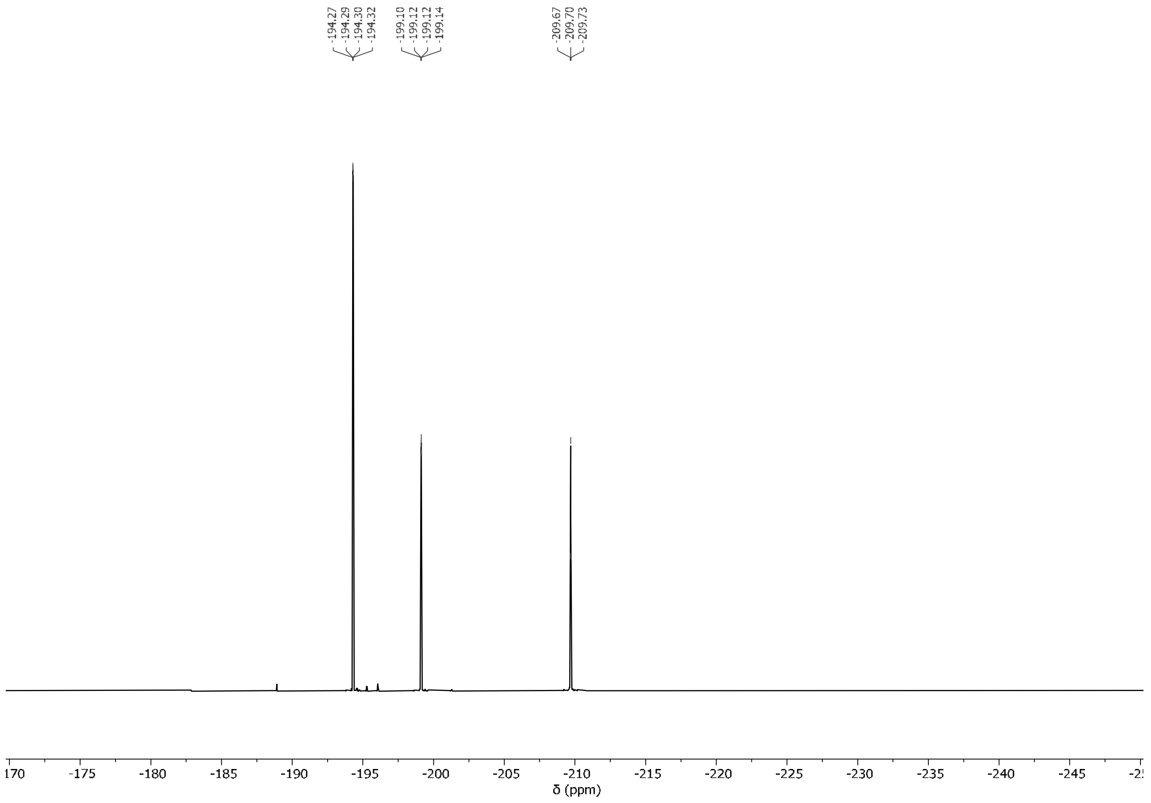
-194.27
-194.29
-194.30
-194.32
-199.10
-199.12
-199.12
-199.14
-209.67
-209.70
-209.73
δ (ppm)
